# DMFpred: Predicting protein disorder molecular functions based on protein cubic language model

**DOI:** 10.1371/journal.pcbi.1010668

**Published:** 2022-10-31

**Authors:** Yihe Pang, Bin Liu

**Affiliations:** 1 School of Computer Science and Technology, Beijing Institute of Technology, Beijing, China; 2 Advanced Research Institute of Multidisciplinary Science, Beijing Institute of Technology, Beijing, China; Georgia Institute of Technology, UNITED STATES

## Abstract

Intrinsically disordered proteins and regions (IDP/IDRs) are widespread in living organisms and perform various essential molecular functions. These functions are summarized as six general categories, including entropic chain, assembler, scavenger, effector, display site, and chaperone. The alteration of IDP functions is responsible for many human diseases. Therefore, identifying the function of disordered proteins is helpful for the studies of drug target discovery and rational drug design. Experimental identification of the molecular functions of IDP in the wet lab is an expensive and laborious procedure that is not applicable on a large scale. Some computational methods have been proposed and mainly focus on predicting the entropic chain function of IDRs, while the computational predictive methods for the remaining five important categories of disordered molecular functions are desired. Motivated by the growing numbers of experimental annotated functional sequences and the need to expand the coverage of disordered protein function predictors, we proposed DMFpred for disordered molecular functions prediction, covering disordered assembler, scavenger, effector, display site and chaperone. DMFpred employs the Protein Cubic Language Model (PCLM), which incorporates three protein language models for characterizing sequences, structural and functional features of proteins, and attention-based alignment for understanding the relationship among three captured features and generating a joint representation of proteins. The PCLM was pre-trained with large-scaled IDR sequences and fine-tuned with functional annotation sequences for molecular function prediction. The predictive performance evaluation on five categories of functional and multi-functional residues suggested that DMFpred provides high-quality predictions. The web-server of DMFpred can be freely accessed from http://bliulab.net/DMFpred/.

## Author summary

Intrinsically disordered proteins (IDPs) are proteins that are without stable three-dimensional (3D) structures in native physiologic conditions. The discovery of IDPs has disproved the idea that proteins must fold into 3D structures to accomplish their biological functions. They are prevalent in eukaryotic organisms and carry out many critical functions in cellular regulation, signaling networks, and disease pathways. These functions of IDPs can be summarized into six general categories, including entropic chain, assembler, scavenger, effector, display site, and chaperone. Experimental identification of the molecular functions of IDP in the wet lab is an expensive and laborious procedure that is not applicable on a large scale. Some computational methods have been proposed to identify the entropic chain function of IDPs, while the predictive methods for the remaining but important functions of IDPs are desired. In this study, we proposed a disordered molecular function computational predictor of proteins, namely DMFpred. The DMFpred supports high-throughput sequences as input and computationally predicts five molecular functions of IDPs, including assembler, scavenger, effector, display site, and chaperone. The evaluation results suggested that DMFpred provides high-quality predictions, and the corresponding web server of DMFpred can be freely accessed from http://bliulab.net/DMFpred/.

This is a *PLOS Computational Biology* Methods paper.

## Introduction

Proteins or regions that lack stable 3D-structures under the native physiologic conditions are known as intrinsically disordered proteins and regions (IDP/IDRs). Recent studies have suggested that IDP/IDRs are common in nature, with more than 30% of proteins in eukaryotes being disordered [1,2]. The widespread occurrence of IDP/IDRs alter the classical protein structure-function paradigm [3–5]. IDP/IDRs play essential roles in living organisms, the alteration of their functions are responsible for many human diseases such as cancer [6], Alzheimer’s [7] and Parkinson’s [8]. Exploring the molecular functional mechanism of IDP/IDRs will be helpful for a complete understanding of protein structures and functions, and will be also used to guide wet lab experiments and inform studies of rational drug design [9,10].

The functions of protein disordered regions arise from their native structural flexibility or from their ability to bind to partner molecules [4]. These disorder functions can be summarized as six categories: entropic chains, assembler, scavenger, effector, display site, and chaperone [4,11]. The disordered entropic chain benefits directly from its intrinsically disordered conformation without becoming structured, which serves as the connector between domains and structural elements making up domains [12]. Disordered assemblers bring together multiple binding partners, and promote the formation of large protein complexes [4,5,13]. Scavenger disordered regions in proteins store and neutralize small ligands, such as chromogranin, salivary glycoproteins and calcium-binding phosphoproteins [11,14,15]. Effectors interact with other partner proteins and modify their activity [16]. Some disordered regions serve as display sites, facilitating easy access and recognition of the post-translational modifications (PTMs) in proteins [17]. Disordered chaperone function makes the IDRs assisting RNA and protein molecules to reach their functionally folded states [18].

The intrinsically disordered is encoded in the protein sequence, motivating the development of computational sequence-based disorder predictors [19]. Currently, there are about 200 million disordered proteins have been identified experimentally and predictively [20]. In contrast, only thousands of disordered proteins have functional annotations [21,22]. This data suggests that it is important to develop computational predictors for filling the deepening gap between annotated and unannotated disordered sequences. In this regard, several sequence-based computational predictors are proposed for predicting specific functions of disordered proteins. For example, the DFLpred [23] and APOD [24] are computational methods developed for predicting disordered linkers that fulfill entropic chain function in proteins. Besides, there are predictors for identifying disordered regions binding to specific types of molecular partners, including protein binding predictors [25–32], DNAs and RNAs binding predictors [33,34], and lipid binding predictors [35]. However, methods for predicting the other five classes (assembler, scavenger, effector, display site and chaperone) of molecular functions of IDRs are required.

Protein representation is critical for the construction of computational predictors. Protein sequence defines structure, which in turn dictates its function [4]. The intrinsically disordered proteins reassessed the classical sequence-structure-function paradigm [36], the complex sequence, structure, and functional properties of IDP/IDRs should be explored to fully represent the disordered proteins. By modelling the language’s generative rules, the language model in natural language processing (NLP) comprehensively understands the language, and capture the semantic features of text, which is an indispensable technology in NLP. Protein sequences can be viewed as the language of genetics sharing high similarities with natural language sentences [37]. For example, the natural language sentences composed of words express their semantics, while proteins composed of residues perform various functions. Inspired by their similarities, the proteins can be represented and modelled by the language models.

In this paper, we proposed DMFpred predictor, which predicts five molecular functions of IDRs, including assembler, scavenger, effector, display site, and chaperone. DMFpred employs the Protein Cubic Language Model (PCLM) to learn protein representations, consisting of three types of protein language models and an attention-based language model alignment (ALAN) module. Three protein language models were used to capture protein sequences, structural, and functional features, respectively. The ALAN module extracts the relationship among three captured features and encodes the complementarity information. The key challenge in functional prediction is that the number of disordered sequences with functional annotations is relatively small. The transfer learning technology can transfer knowledge from tasks with plentiful training data to improve the performance of similar other tasks, which is especially useful for the task with limited training data [38]. Therefore, we first pre-trained PCLM with large IDRs sequences to capture the disordered features of proteins. Then the general disordered features were transferred separately to five different disorder functions prediction via model fine-tuning. Benefited from pre-training and function-specific fine-tuning of PCLM, DMFpred captures more relevant features of disorder molecular functions. The ablation experiment results demonstrated that each module of PCLM contributes to the predictive performance improvements. And further evaluation suggested that DMFpred provides high-quality predictions on all five categories of functional residues and multi-functional residues, whose residues carry more than one category of molecular functions. The corresponding web server of DMFpred was established and can be freely accessed from http://bliulab.net/DMFpred/.

## Materials and methods

### Benchmark datasets

The datasets used in this study were collected from DisProt [22], which is the major repository of manually curated functional annotations of intrinsically disordered proteins from literature. All sequences in the database are functionally annotated at the amino acid level. In this study, we focused on five general categories of disordered molecular functions (DMFs), including assembler, scavenger, effector, display site and chaperone. Following the intrinsically Disordered Proteins Ontology (IDPO) schema in the DisProt, each of the five categories of function terms has one or two leaf terms (see S1 **Fig**). Here, we treat all the leaf terms as the same functional class as their root terms. The sequences in the database are functionally annotated with amino acids as the basic unit, and we collected a total of 590 sequences containing residues assigned at least one class of DMFs. For each class of function, we treat the residues annotated with the functional term class in the database as functional residues, and the others as non-functional residues. Then we assign all the functional residues in the sequences as label ‘1’ and non-functional residues as label ‘0’, leading to five lines of labels corresponding to five categories of DMFs annotations.

To avoid data redundancy, we performed the similarity clustering on the 590 sequences by using PSI-BLAST [39] by setting the threshold of 25%, and filtered sequences with pairwise sequence similarity >25%. This way ensured that the sequence similarity between any two sequences in the collections was lower than 25%. The remaining 541 proteins were randomly divided into training, evaluation, and test sets in a ratio of 6:2:2. Finally, 324 sequences were used as the training set for model training, 106 sequences were used as the valuation set for model selection, and 111 sequences were used as the independent test set (TEST-1) to evaluate predictive performance (**S1 Data**). The number of functional residues for the five categories of disordered molecular functions in the DMF benchmark datasets is given in **Table 1**.

**Table 1:** The number of functional residues in the DMF benchmark datasets.

Dataset	Number of Assembler residue	Number of Chaperone residue	Number of Display-site residue	Number of Effector residue	Number of Scavenger residue
Training set	14177	2236	5041	12783	2202
Evaluation set	7764	904	932	4102	1022
TEST-1	4412	836	1001	3980	545

### Architecture of protein cubic language model

#### Sequence, structure and function language models

Sequence, structure, and function are three important aspects of proteins. Only one language model cannot fully characterize the three features. In this paper, we employed three types of language models for capturing the sequences, structural, and functional features of proteins.

#### Sequence language model

**Fig 1:**
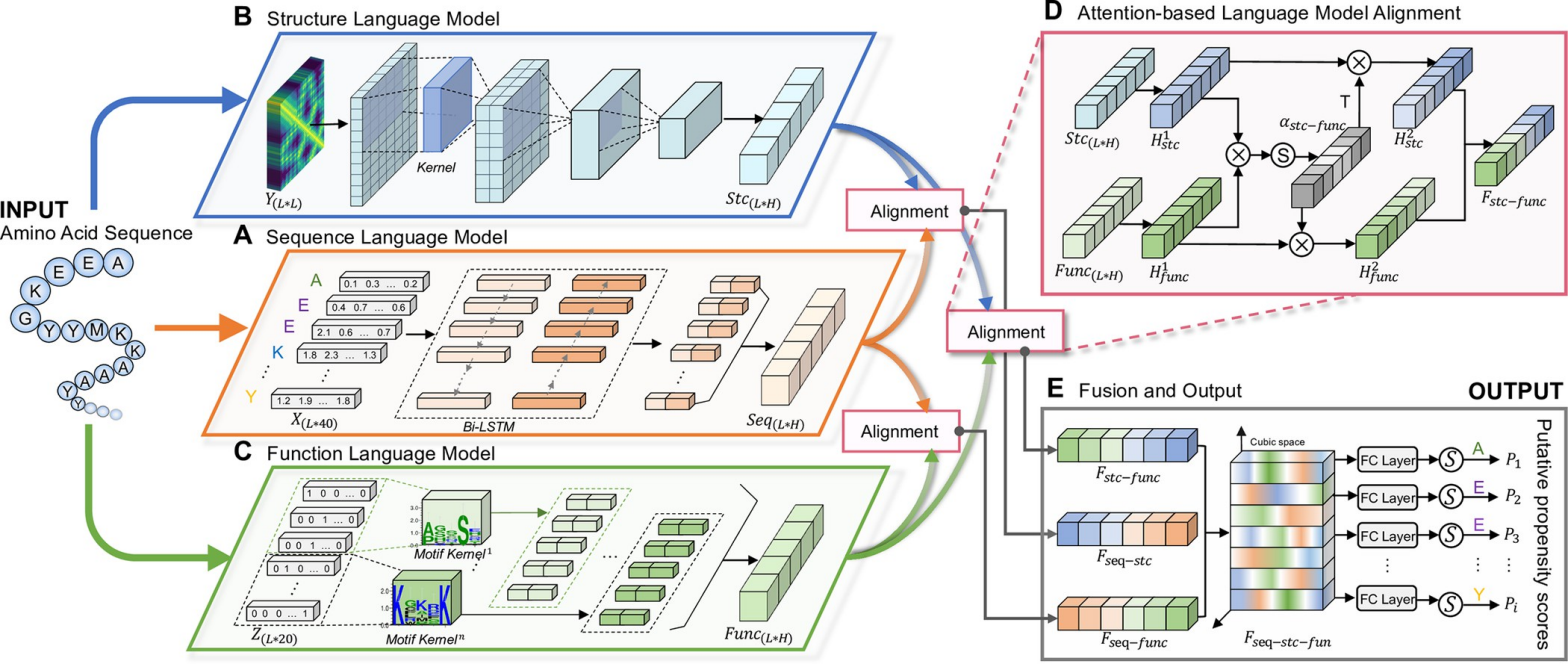
The architecture of protein cubic language model (PCLM). The PCLM contains five main modules: three protein language models (**A**. sequence, **B**. structure, and **C**. function language model), attention-based language model alignment module (**D.** ALAN), and the fusion and output layer (**E**). The input protein sequence is converted to sequence profile **X**, structure profile **Y**, and function profile **Z**, which are then fed into three protein language models to capture the sequence features **Seq**, the structure features **Stc**, and the function features **Func**. Next, three captured features are incorporated into the alignment features (**F**_*stc−func*_, **F**_*seq−stc*_ and **F**_*seq−func*_) by ALAN modules. Finally, the fusion and output layers merge the outputs of ALAN to calculate the propensity score *P*_*i*_ of disorder molecular function for each residue.

The amino acid sequence contains the evolutionary information of protein. Here, the bidirectional long short-term memory (Bi-LSTM) networks were employed as the sequence language model to capture the global correlation features of evolutionary information (see **Fig 1A**). By using the protein PSSM profile and HMM profile as the inputs of the sequence language model, the sequence features **Seq** can be calculated by [40]:

$$Seq=[h_{1},h_{2},\cdots ,h_{L}]$$ (1)

$$h_{i}=Concat[{LSTM}_{f}(X_{L\times 40}),{LSTM}_{b}(X_{L\times 40})]$$ (2)

where **X**_*L*×40_ is the combination of PSSM and HMM matrix generated by PSI-BLAST [39] and HH-suits [41] respectively, and *L* is the length of the sequence. *LSTM*_*f*_ and *LSTM*_*b*_ indicate the forward and backward recurrent neural unit respectively. *Concat* represents the combination of vectors.

### Structure language model

Protein structure reflects the results of local interaction among residues. The structure language model aims to capture structural features of the protein, and a convolutional-based model is used to capture structural local pattern features from the residue-residue contact map (CCM) (see **Fig 1B**). By taking CCMs as inputs, the structure features **Stc** can be calculated by [42]:

$$Stc=relu[Conv(Y_{L\times L},{Filter}_{stc})+b_{stc}]$$ (3)

where **Y**_*L*×*L*_ is the CCM profile generated by CCMpred [43,44], **Filter**_*stc*_ and **b**_*stc*_ are trainable variables, Conv represents convolution operator, and *relu* is the Rectified Linear Unit activation function [45].

### Function language model

Functional conservative sequence segments also known as functional motifs hold particular functionality information of proteins. Previous researches [46–48] have shown that the motif-based convolution (MotifConv) by embedding particular motifs into the convolution kernel can learn the prior biological features. Inspired by MotifConv, the functional motif-based convolution was employed as the function language model to capture proteins’ functional features (see **Fig 1C**). The 164 motifs used in this study were extracted from the Eukaryotic Linear Motif (ELM) database [49]. The letter-probability matrix of each motif is used to build the convolution kernel formulated as:

$$M_{1}=\left[\begin{array}{ccc} a_{1,1} & \cdots & a_{1,20}\\ \vdots & \ddots & \vdots \\ a_{l,1} & \cdots & a_{l,20} \end{array}\right]$$ (4)

where *l* is the length of motif, *a*_*i*,*j*_ represents the frequency of standard amino acid. Then the function features **Func** can be calculated by:

$$Func=relu[Conv(Z_{L\times 20},M)+b_{func}]$$ (5)

where **Z**_*L*×20_ is the one-hot encoding matrix of protein sequence, **M** is the combination of 164 motif convolution kernel matrix, and **b**_*func*_ is trainable variable.

### Attention based language model alignment

The primary sequences encode the disordered states of IDP/IDRs, which in turn determine functions. The potential correlations among sequence, structure and function are essential information for the protein representations. In this study, attention alignment models the correlations between protein features by calculating the attention alignment weights on two kinds of features (see **Fig 1D**). For example, given the sequence features **Seq**, structure features **Stc**, and function features **Func**, the attention-alignment weights **α**_*seq−stc*_, **α**_*seq−func*_ and **α**_*stc−func*_ are calculated by:

$$\alpha_{seq-stc}=softmax(H_{seq}^{1}Seq\times H_{stc}^{1}Stc)$$ (6)

$$\alpha_{seq-func}=softmax(H_{seq}^{1}Seq\times H_{func}^{1}Func)$$ (7)

$$\alpha_{stc-func}=softmax(H_{stc}^{1}Stc\times H_{func}^{1}Func)$$ (8)

where $H_{seq}^{1}$, $H_{stc}^{1}$ and $H_{func}^{1}$ are the trainable weight variables. The attention-alignment weights between two kinds of features reflect matching patterns between different property aspects of the proteins. Weighted by the attention-alignment weights, the sequence features **Seq**, structure features **Stc** and function features **Func** captured by three language models can be enhanced and fused into the complementary features **F**_*seq−stc*_, **F**_*seq−func*_ and **F**_*stc−func*_:

$$F_{seq-stc}=Concat(H_{seq}^{2}\alpha_{seq-stc}^{T}{Seq}^{\prime },H_{stc}^{2}\alpha_{seq-stc}{Stc}^{\prime })$$ (9)

$$F_{seq-func}=Concat(H_{seq}^{2}\alpha_{seq-func}^{T}{Seq}^{\prime },H_{func}^{2}\alpha_{seq-func}{Func}^{\prime })$$ (10)

$$F_{stc-func}=Concat(H_{func}^{2}\alpha_{stc-func}^{T}{Stc}^{\prime },H_{stc}^{2}\alpha_{stc-func}{Func}^{\prime })$$ (11)

where $H_{seq}^{2}$ $H_{stc}^{2}$ and $H_{func}^{2}$ are the trainable variables, **Seq**′, **Stc**′ and **Func**′ indicate the transformed feature matrix of **Seq**, **Stc** and **Func**, respectively. The *softmax* is the activation function. The complementary features **F**_*seq−stc*_, **F**_*seq−func*_ and **F**_*stc−func*_ learn the correlations among sequence, structure, and functional properties of proteins, and these features are fed into the cubic fusion and output layers for calculating the predictive propensity score.

### Cubic fusion and output layer

The cubic fusion module of PCLM merges the three alignment complementary features into latent cubic space, and obtains a joint representation matrix **F**_*seq−stc−func*_ of protein sequences:

$$F_{seq-stc-func}=W_{x}F_{seq-stc}+W_{y}F_{seq-func}+W_{z}F_{stc-func}$$ (12)

$${F_{seq-stc-func}=[F_{1},\cdots ,F_{L-1},F_{L}],F}_{seq-stc-func}\in R^{L\times n}$$ (13)

where *L* denotes the length of the input sequence, *n* denotes the dimension of features, **W**_*x*_, **W**_*y*_, and **W**_*z*_ are the trainable weighted variables. Each vector **F**_*i*_ in the representation matrix represents the features of each residue in the sequence. The fully connected (FC) layer captures the global and local correlations between residues in the sequence so as to calculate the propensity score *P*_*i*_ for each residue:

$$P[P_{1},\ldots ,P_{i},{\ldots ,P}_{L}]=Sigmoid(W_{f}F_{seq-stc-func}+b_{f})$$ (14)

where **W**_*f*_ *a*nd **b**_*f*_ represent the weighted and bias variables, respectively.

### Pre-training of protein cubic language model

The transfer learning involves a model training strategy, which transfers the knowledge learned from the source domain to a new and different target domain. It is especially effective when the target domain has insufficient training data [38]. In this study, although we have relatively limited number of disorder functional annotation regions for PCLM model training, the number of intrinsically disordered regions (IDRs) is sufficient. The large number of IDRs will overcome the problem that model cannot be fully trained with insufficient disorder functional data, and the generic disordered features learned from IDR dataset can be transferred to facilitate the disorder molecular function prediction. Therefore, in this study, we employed the widely used IDP/IDR prediction benchmark dataset [40] as the pre-training dataset to pre-train PCLM model for predicting disordered regions in protein. To avoid data redundancy, we excluded sequences with >25% sequence similarity to the disordered functional benchmark datasets, and obtained 2639 sequences with 38134 IDRs and 1079 sequences with 16403 IDRs for model pre-training and validation, respectively (**S2 Data**). The binary cross-entropy loss function was used to calculate the loss score for model parameters optimizing [50]:

$$loss=-\sum_{i}[y_{i}log\;p_{i}+(1-y_{i})log\;(1-p_{i})]$$ (15)

where *p*_*i*_ denotes the predictive score for residue *R*_*i*_ being disordered calculated by **Eq 14**, and *y*_*i*_ represents the actual label of disordered residue. The Adam optimizer [51] with a learning rate of 0.001 was employed to optimize the model parameters, and the model with the minimized loss score on the IDR validation set was saved as the pre-trained model.

### Fine-tuning PCLM for predicting disordered molecular functions

**Fig 2:**
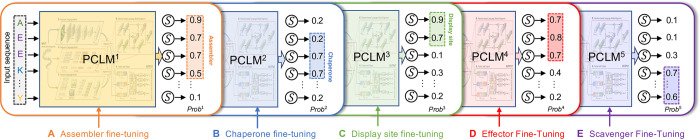
The functional specific fine-tuning of protein cubic language model (PCLM). The pretrained PCLM model was separately fine-tuned with five categories of disordered molecular functions into five corresponding PCLM^1-5^ models for predicting assembler (**A**) chaperone (**B**) display site (**C**) effector (**D**) and scavenger (**E**) functional residues from the input sequence. *Prob*_1_, *Prob*_2_, *Prob*_3_, *Prob*_4_, and *Prob*_5_ represent the predicted propensity scores for the five functions of input sequence, respectively.

In the fine-tuning stage, the pre-trained PCLM model was fine-tuned with functional specific data for predicting the disordered molecular functions in protein. Because of the differences between the five molecular functions, we separately fine-tuned PCLM with assembler, chaperone, display site, effector, and scavenger functional annotations in the DMF benchmark dataset, leading to five independent predicting PCLM models (see **Fig 2**). In the DMFpred predictor, the five functional specific fine-tuned PCLM models work in parallel to produce five disordered molecular functional predictions for each residue in the input proteins. Here, we used the same loss function and optimizer as the ones used in the pre-training stage, but different learning rates to fine-tune the model parameters for each function. Parameters of all layers in PCLM were fine-tuned for achieving better performance, and this strategy has been adopted by many transfer learning based studies [52,53]. More detailed hyper-parameters for DMFpred are given in **S1 Table**.

### Evaluation criteria

DMFpred generates two forms of outputs: the real-valued propensity score (the likelihood of residue with the given function) and binary results (residue with or without the given function). Binary predictions were converted from the propensities: one residue is predicted as functional residue if its propensity score is greater than a given threshold. Otherwise, it is predicted as the non-functional residue. The receiver operating characteristic curve (ROC) and AUC value (area under ROC curve) were utilized to evaluate the predictive performance of the real-valued propensity prediction. Sensitivity (Sn), specificity (Sp) and accuracy (ACC) were used for the evaluation of the binary results. Since the dataset is imbalanced, *i*.*e*. there are many more non-functional residues than the functional residues. Therefore, two metrics, balanced accuracy (BACC) and the Matthews Correlation Coefficient (MCC) were used to measure the predictive performance.

Disordered residues interact with multiple partners with more than one functions are called the multi-functional residues. The residue-level functional prediction of these multi-functional residues can be treated as a multi-label learning task, and five example-based metrics were utilized to evaluate the performance of DMFpred on multi-functional residues [54]:

$$\left\{ \begin{array}{c} Hammingloss=\frac{1}{p}\sum_{i=1}^{p}\frac{1}{q}|h(x_{i})∆Y_{i}|\\ {Accuracy}_{exam}=\frac{1}{p}\sum_{i=1}^{p}\frac{\left|h(x_{i})\cap Y_{i}\right|}{\left|h(x_{i})\cup Y_{i}\right|}\\ {Precision}_{exam}=\frac{1}{p}\sum_{i=1}^{p}\frac{\left|h(x_{i})\cap Y_{i}\right|}{\left|h(x_{i})\right|}\\ {Recall}_{exam}=\frac{1}{p}\sum_{i=1}^{p}\frac{\left|h(x_{i})\cap Y_{i}\right|}{\left|Y_{i}\right|}\\ {F1}_{exam}=\frac{2\times {Precision}_{exam}\times {Recall}_{exam}}{{Precision}_{exam}+{Recall}_{exam}} \end{array}\right.$$ (16)

where *p* indicates the total number of samples, *q* indicates the number of labels, *h*(*x*_*i*_) is the predicted label set and *Y*_*i*_ is the true label set. Δ represents the symmetric difference between two sets.

## Results and discussion

### Functional specific fine-tuning achieves better performance

**Fig 3:**
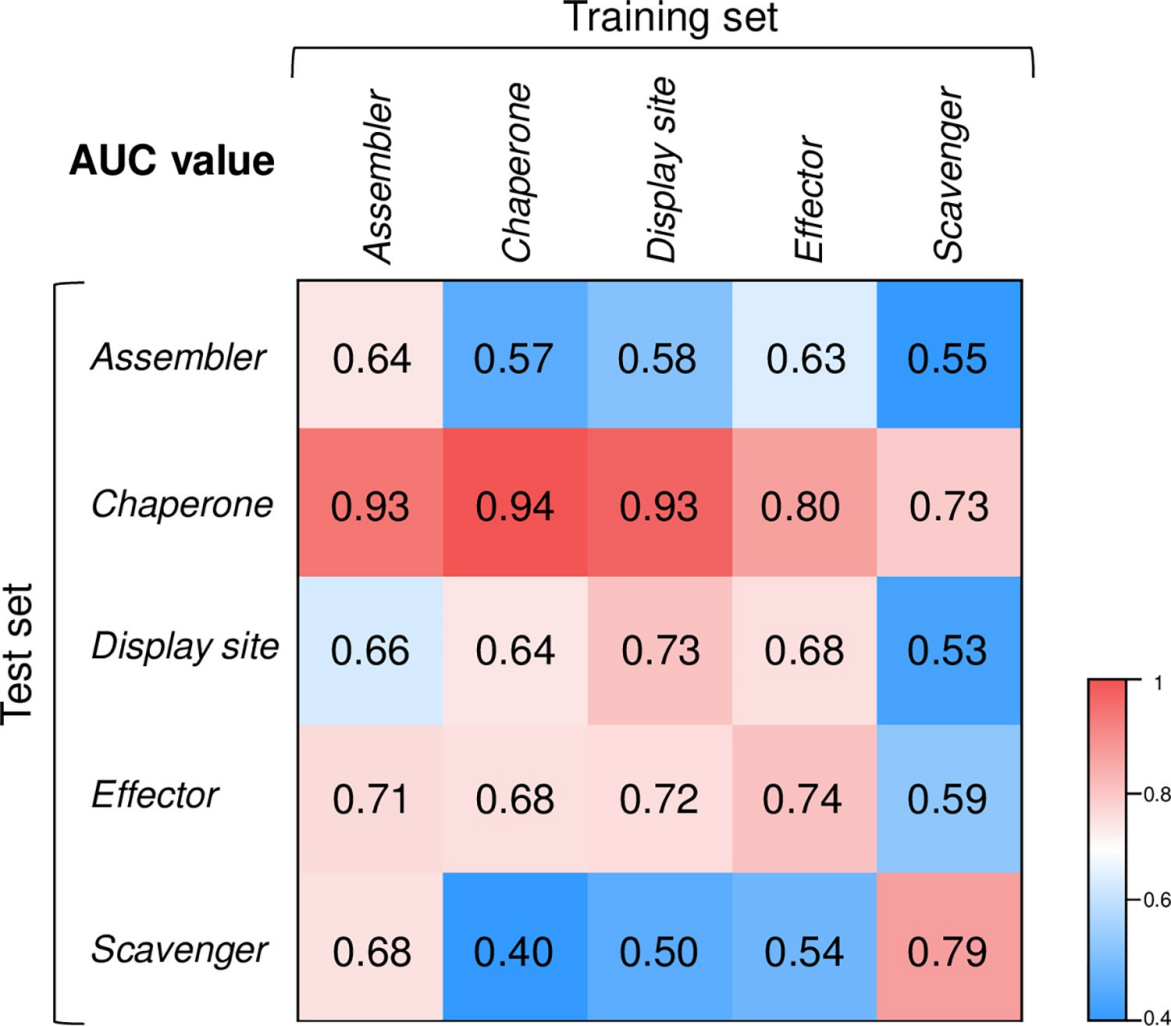
Cross-functional validation results. Functions on the x-axis were used to fine-tune PCLM model, and functions on the y-axis were used for model validation.

In order to investigate the differences among five categories of molecular functions, we performed the cross-functional validation on the benchmark datasets. To avoid the overestimation caused by the multi-functional residues, sequences that only belonging to one class function in the training and validation sets are used to fine-tune and test the PCLM model. The AUC evaluation results are shown in **Fig 3**. From **Fig 3**, we can see that model fine-tuned and tested on the same function achieves the best performance, while cross-functional predictors achieve lower performances. These predictive results suggest that specialized predictors are required for each functional category, and function-specific fine-tuning is the key to achieve better predictive performance of each disordered molecular function.

### Ablation analysis of protein cubic language models

**Fig 4:**
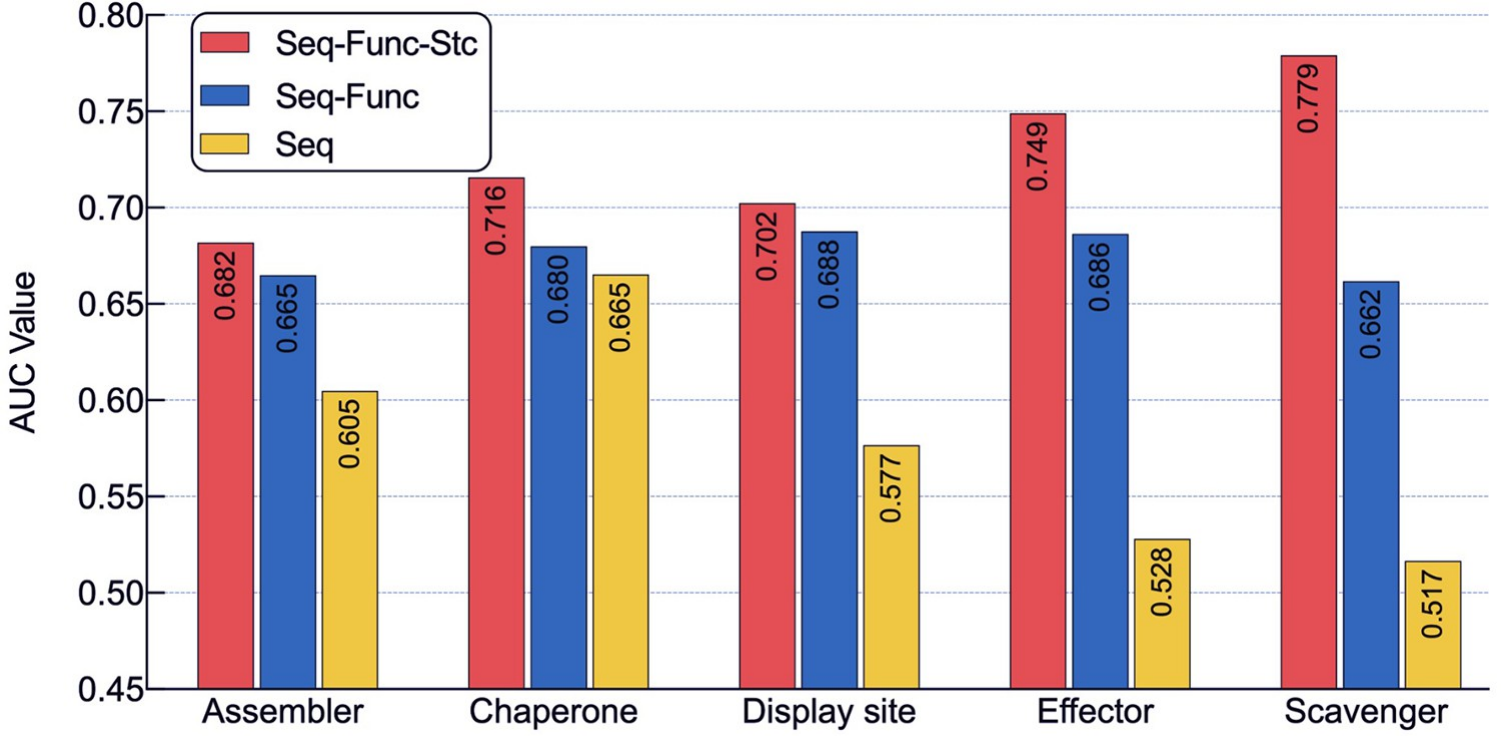
The predictive results of PCLM model in DMFpred with different language models. *Seq* represents the PCLM with only sequence language model, *Seq-Func* denotes the PCLM with the combination of sequence language model and function language model, and *Seq-Func-Stc* stands for the PCLM model, which is the combination of sequence language model, function language model and structure language model. The AUC values were calculated on the validation data set.

To verify the contribution of three language models to DMFpred, we performed an ablation analysis. The PCLM models with different combinations of three language models were individually fine-tuned on five molecular function training data, and the corresponding AUC values for each function evaluated on validation dataset are shown in **Fig 4**. We can see that (i) predictors with the combination of three language models consistently achieve the best performances for all five functions; (ii) the prediction performance of predictor decreased by dropping the structural language model. Predictors with only sequence language model performed the worst. These results are not surprising because three language models capture the sequence, structural, and functional features of proteins, and these three features are complementary, and contribute to the functional prediction. As a result, predictors incorporating the three protein language models achieve the best performance.

### Attention based language model alignment learns the correlation patterns

**Fig 5:**
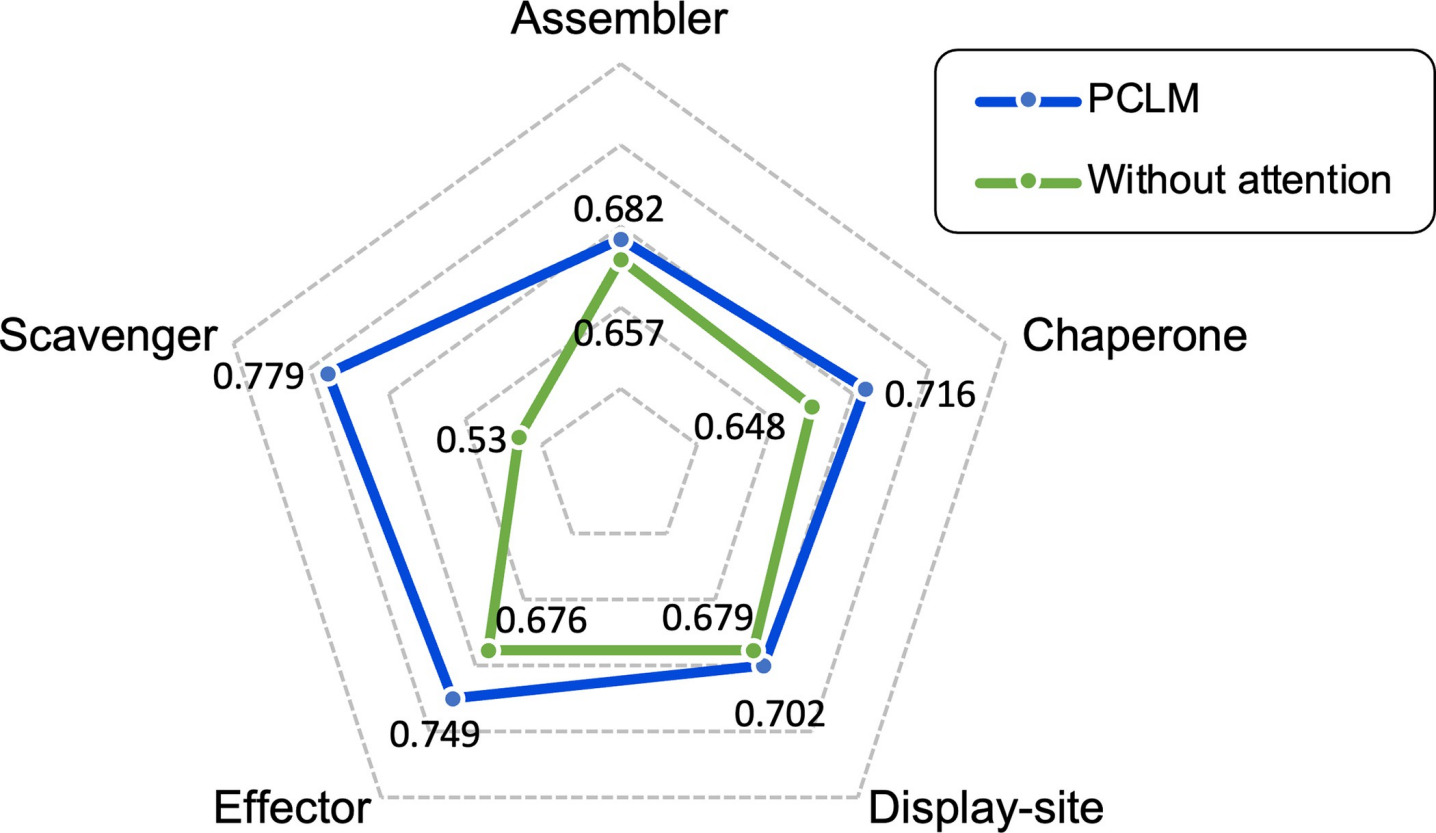
The predictive results (AUC values) of DMFpred with and without attention-based language model alignment. The *PCLM* represents the entire PCLM predictive model, while the *Without attention* denotes the PCLM model without the ALAN module. Both two models were independently fine-tuned and evaluated for the five molecular functions on the training dataset and validation dataset, respectively.

In order to investigate the performance improvement of attention-based language model alignment (ALAN) to the proposed predictor. We compared the performance of predictors for predicting five disordered molecular functions by using PCLM model with and without the ALAN module. The PCLM model without ALAN directly feed the features captured by the three language models to the fusion and output layers (see **Fig 1**) to calculate prediction results. The two types of models were independently fine-tuned with five different functions, and the results evaluated on the validation dataset are shown in **Fig 5**. From this figure, we can see that predictors with ALAN consistently outperform the predictors without ALAN on five classes of functions, demonstrating the effectiveness of the ALAN module. Furthermore, we note that the predictor for Scavenger function with an ALAN achieves better performance in terms of AUC value. These results may be caused by the fact that the complementary features captured by the ALAN module supplemented the inadequate sequence, structure and functional features learned from limited annotated sequences. This improvement is especially manifested in the Scavenger function with a relatively small number of annotated sequences. Benefitted from the features captured by ALAN, predictor can make more accurate prediction leading to better performance.

**Fig 6:**
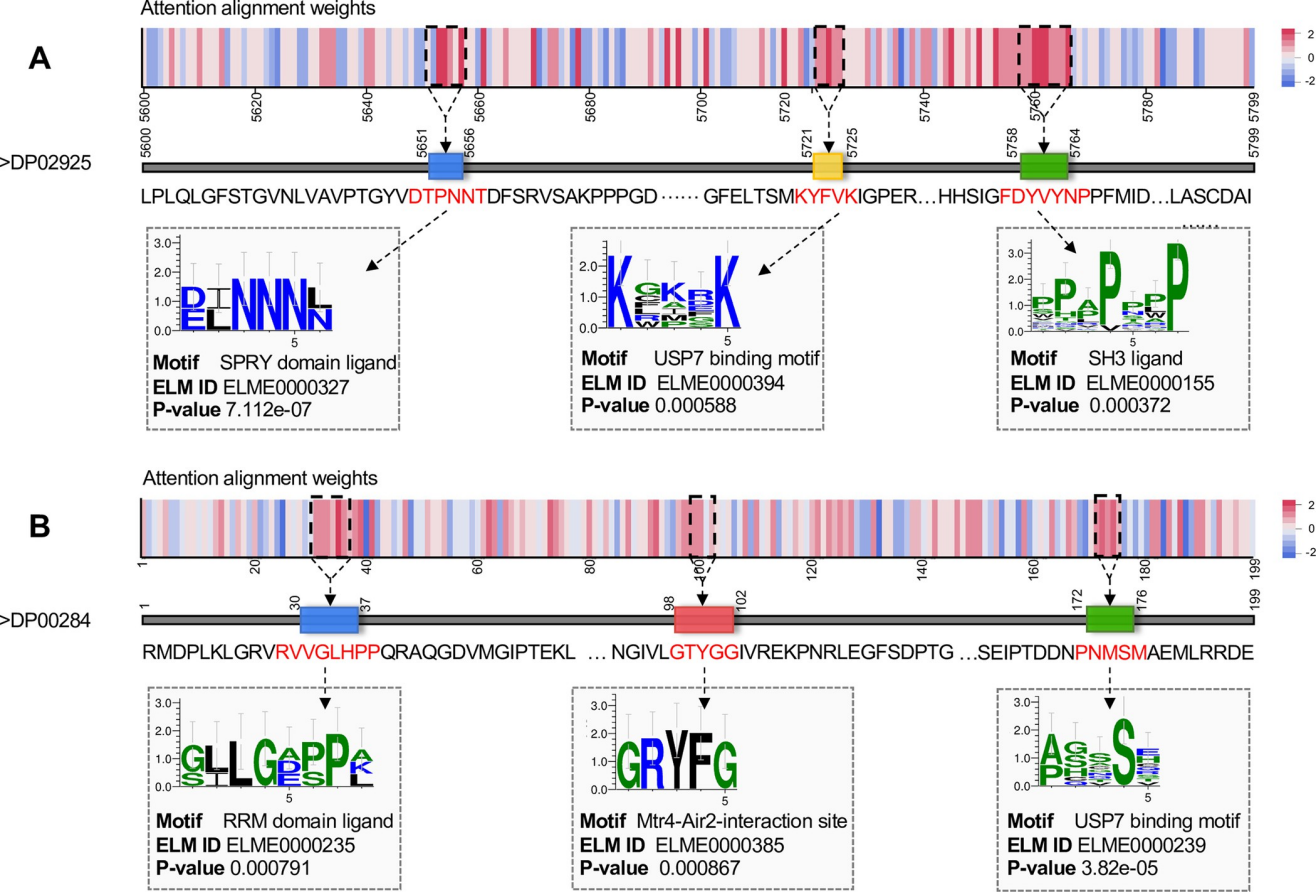
The attention alignment weight visualizations.

To further analyse the information learned by the ALAN module, we visualized the attention-alignment weights between sequence and structure features. Two protein examples (DisProt ID: DP02925 and DP00284) selected from the independent test set (TEST-1) were visualized in **Fig 6**, from which we can see that the specific segments in the sequences map with the highest attention weights, and these sequence segments corresponding to the experimentally determined functional motifs searched from the ELM database [49] by FIMO tools (https://meme-suite.org/meme/tools/fimo). These results indicate that the ALAN can capture critical correlation patterns by modelling the relationship between different protein features. This prior biological knowledge captured by ALAN complements the original sequence, structure and functional attributes of proteins, providing a powerful protein representation.

### Model pre-training facilitates feature correlation

**Fig 7:**
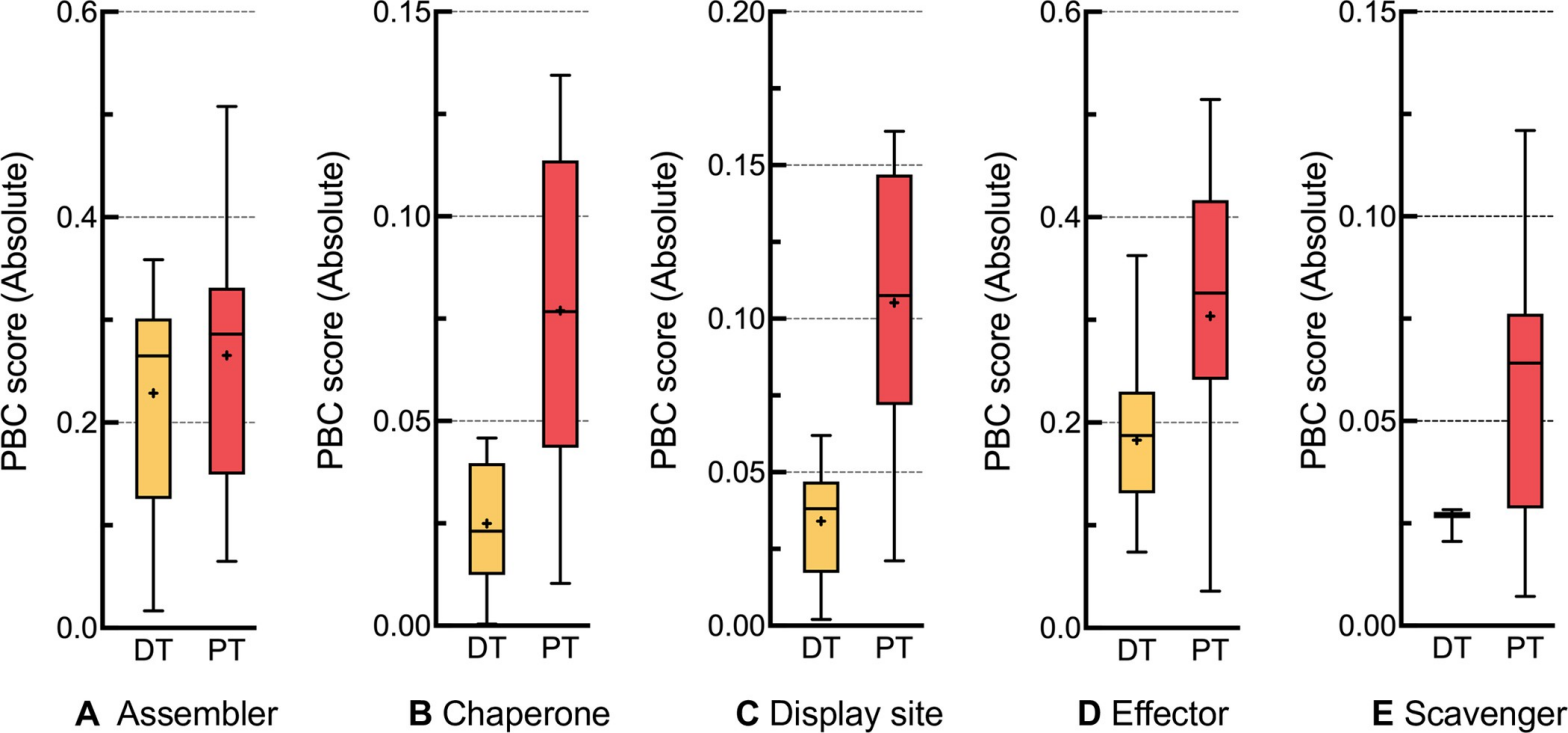
Absolute PBC score distributions on five functions. DT represents the PCLM models directly trained with molecular functional data, and PT represents the PCLM models pre-trained with disordered proteins.

In order to explore the contribution of model pre-trained with disordered proteins, we compare the predictive power of features extracted between models directly trained with molecular functional sequences (DT in **Fig 7**) and the fine-tuned model based on pre-training with IDRs (PT in **Fig 7**). Following previous studies [23, 24], the absolute point-biserial correlation (PBC) score is used to quantify the feature predictive qualities, which reflects the correlation between numeric and binary variables:

$$PBC=\frac{m_{1}-m_{0}}{s_{n}}\sqrt{\frac{n_{0}\times n_{1}}{n\times n}}$$ (17)

where *n*_0_ and *n*_1_ indicate the number of functional and non-functional residues, *m*_0_ and *m*_1_ indicate the average values of features of functional and non-functional residues, *s*_*n*_ is the standard deviation of all values of features, and *n* is the total number of residues. The PBC score results for five functions on the TEST-1 independent test set are shown in **Fig 7**. From this figure, we observe that the features captured by the pre-trained model are consistently outperformed that directly trained model on all five functions. This is because model pre-trained with IDR sequences captures more disordered features than directly trained on limited functional sequences. As the functional residues are the sub-set of disordered regions, the common disordered features captured by pre-trained model facilitate to distinguish disordered functional residues from ordered residues, leading to a robust predictive quality.

### Overall results

To our best knowledge, DMFpred is currently the only predictor for predicting the five general molecular functions of disordered proteins. There are two forms of outputs of DMFpred: real-valued propensity results and binary results. We used the ROC curve and AUC value for evaluating the real-valued predictive results. Sn, Sp, ACC and two metrics for imbalanced datasets (BACC and MCC) were used to assess binary results. The evaluation results on the TEST-1 independent test set are shown in **Table 2** (the ROC curve and thresholds settings see **S2 Fig, S2 Table**). From **Table 2**, we can see that DMFpred provides accurate predictive performance for all five functional categories in terms of AUC values. The Sn, Sp and ACC results show the ability of DMFpred to correctly predict functional and non-functional residues, demonstrating the predictive performance.

**Table 2:** The predictive performance of DMFpred for five categories of molecular functions on TEST-1 independent test set.

Function	AUC	Sn	Sp	ACC	BACC	MCC
Assembler	0.682	0.428	0.804	0.778	0.616	0.143
Chaperone	0.716	0.379	0.919	0.912	0.650	0.120
Display-site	0.702	0.291	0.962	0.952	0.627	0.155
Effector	0.749	0.663	0.741	0.736	0.703	0.215
Scavenger	0.779	0.999	0.520	0.524	0.761	0.095

In order to further evaluate the predictive performance of the predictor, we constructed a new independent test set (TEST-2) with the sequences newly added into the DisProt database during July 2021 to June 2022 by following the same dataset collection protocols. TEST-2 contains 47 proteins with 5780 functional residues, including 3753 assemblers, 218 chaperones, 855 display sites, 682 effectors and 272 scavengers. The prediction results of DMFpred on TEST-2 are shown in **S3 Table**. From these results, we can see that the predictive results achieved by DMFpred on the new independent test set TEST-2 are highly comparable with those on the independent test dataset TEST-1, indicating that the performance of DMFpred predictor is stable.

### Predictive results on the multi-functional residues

The disordered residues interacting with multiple partners with more than one functions are called multi-functional residues. In order to investigate the performance of DMFpred predictor for predicting these multi-functional residues, we collected all the residues with at least two functional annotations from TEST-1 dataset, and obtained a total number of 1352 multi-functional residues for performance evaluation. We compare DMFpred with a random baseline predictor generating the multi-functional labels for each residue with a probability of 0.5, and the evaluation results are shown in **Table 3**. From this table, we can see the followings: (i) compared with the baseline predictor, DMFpred achieves lower Hamming loss, but higher accuracy, which indicates DMFpred can accurately predict more multi-functional residues than the baseline predictor. (ii) DMFpred achieves higher performance than the baseline method in terms of precision, recall rate and F1 value. These results are not surprising because DMFpred was fine-tuned with function-specific labels on the benchmark dataset so as to learn the discriminative features of each function. Benefitting from the accurate prediction for five functions, DMFpred achieves better performance for predicting multi-functional residues.

**Table 3:** The predictive results for multi-functional residues on TEST-1 independent test set.

Predictor	Hamming loss	Accuracy_exam_	Precision_exam_	Recall_exam_	F1_exam_
DMFpred	0.413	0.404	0.504	0.671	0.576
Baseline	0.508	0.285	0.409	0.485	0.444

## Conclusion

Intrinsically disordered proteins/regions perform various molecular functions in living organisms. These functions of IDP/IDRs can be summarized as six general categories, including entropic chains, assembler, scavenger, effector, display site and chaperone. Motivated by the growing numbers of the annotated disordered sequences and the need to expand the coverage of disordered protein function predictors, we introduce the disordered molecular functional predictor called DMFpred, covering five important categories: disordered assembler, scavenger, effector, display site and chaperone. It has the following advantages: 1) DMFpred employed the protein cubic language model (PCLM) that incorporates three protein language models for characterizing sequence, structure, and functional attributes of proteins. PCLM employed attention-based language model alignment to capture the sequence-structure-function correlation and learn a joint representation of proteins. 2) Benefited from the pre-training and function-specific fine-tuning of PCLM, DMFpred captures discriminative features for five functional categories prediction. 3) The evaluation results on five categories of functional and multi-functional residues suggest that DMFpred provides high quality predictions. 4) The web-server of DMFpred is established and can be freely accessed from http://bliulab.net/DMFpred/, which will be helpful to researchers working on the related fields.

## Supporting information

S1 Fig The five disordered molecular functions and their sub-level annotations collected from the DisProt database. (TIF)

S2 Fig The ROC curves of DMFpred for predicting five disordered molecular functions. (TIF)

S1 Table The hyper-parameters of PCLM in DMFpred. (DOCX)

S2 Table The thresholds used for binary results of DMFpred. The thresholds were selected according to the highest MCC values in the validation set.
(DOCX)

S3 Table The predictive performance of DMFpred on the TEST-2 independent test set. (DOCX)

S1 Data The molecular function benchmark dataset. (DOCX)

S2 Data The IDRs pre-training dataset. (DOCX)
