# Supplementary figures and images for: DMFpred: Predicting protein disorder molecular functions based on protein cubic language model
Source: PLoS Comput Biol. 2022 Oct 31;18(10):e1010668. doi: 10.1371/journal.pcbi.1010668 (PMC9674156; doi:10.1371/journal.pcbi.1010668)

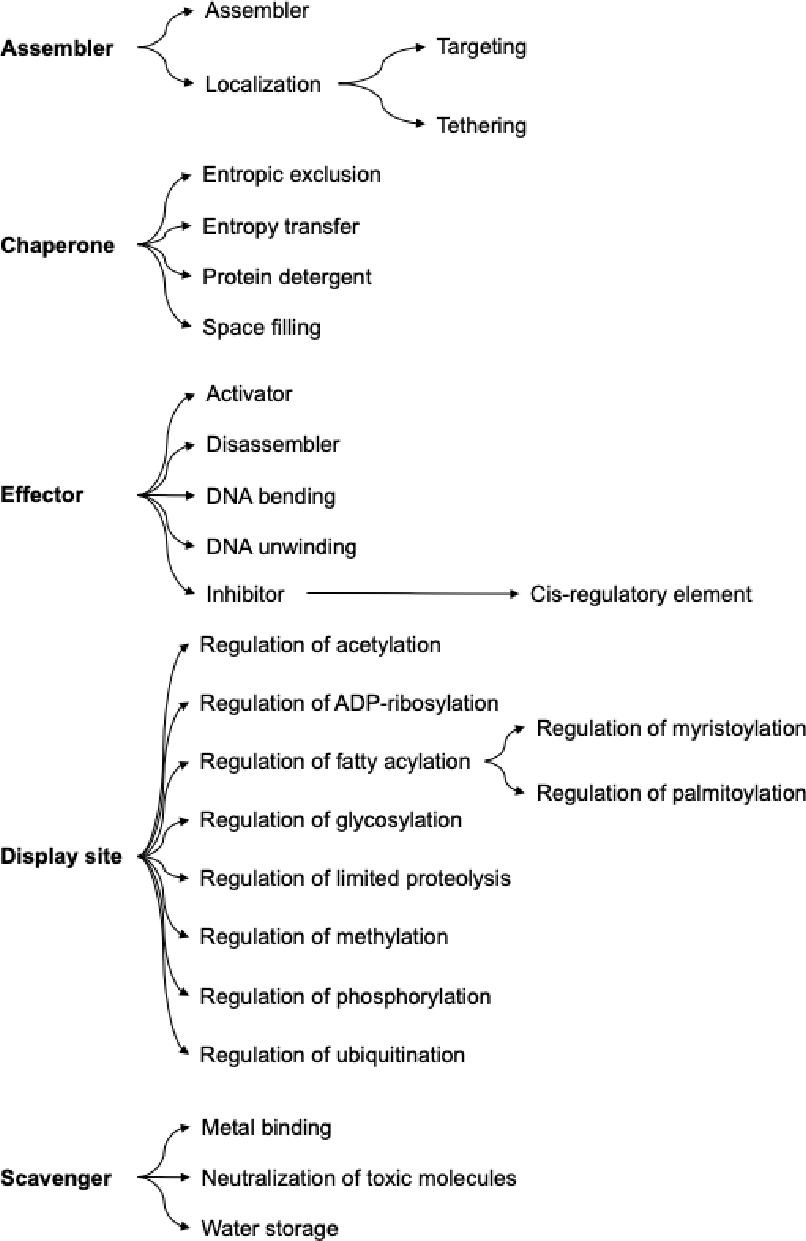

Supplement: S1 Fig — (TIF) [file pcbi.1010668.s001.tif]

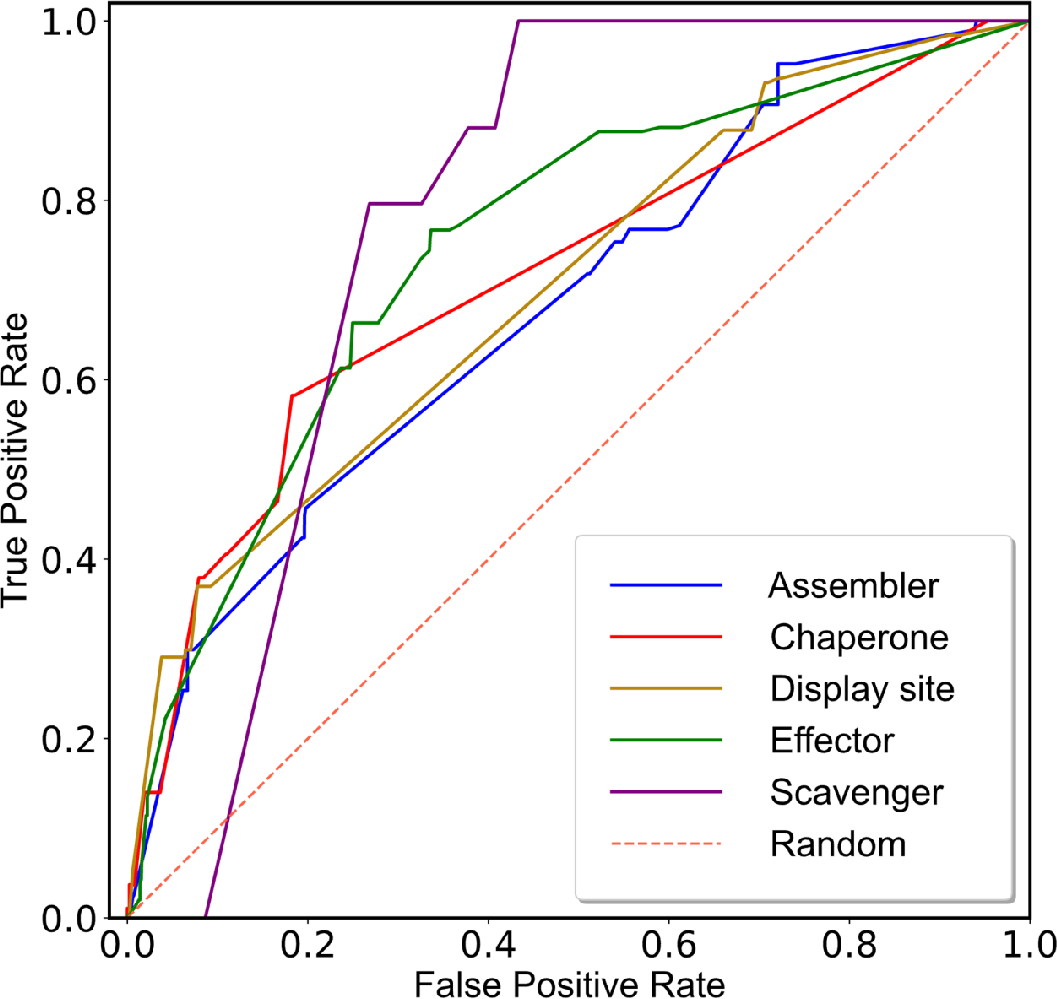

Supplement: S2 Fig — (TIF) [file pcbi.1010668.s002.tif]
