# Supplementary material for: DMFpred: Predicting protein disorder molecular functions based on protein cubic language model
Source: PLoS Comput Biol. 2022 Oct 31;18(10):e1010668. doi: 10.1371/journal.pcbi.1010668 (PMC9674156; doi:10.1371/journal.pcbi.1010668)
Supplement: S1 Table — (DOCX) [file pcbi.1010668.s003.docx]

**Table S1.** The hyper-parameters of PCLM in DMFpred.

| **Hyper-parameter** | | **Setting** | |
| --- | --- | --- | --- |
| Sequence language model | Bi-LSTM | Recurrent cell: LSTM | |
|  |  | Hidden size of ${LSTM}_{f}$: 32 | |
|  |  | Hidden size of ${LSTM}_{b}$: 32 | |
| Structure language model | CNN | Filter_size: [7,7,1,64] | |
|  |  | Stride: [1,1] | |
|  |  | Bias variable: 64 | |
| Function language model | MotifConv | Number of filter: 164 | |
|  |  | Filter_size: [*l*,20,1,1] | |
|  |  | Stride: [1,20] | |
|  |  | Bias variable: 1 | |
| Sequence-structure attention alignment | Attention weight | Hidden size of seq and stc: 64 | |
|  | Output | Hidden size of seq and stc: 64 | |
| Sequence-function attention alignment | Attention weight | Hidden size of seq and func: 64 | |
|  | Output | Hidden size of seq and func: 64 | |
| Structure-function attention alignment | Attention weight | Hidden size of stc and func: 64 | |
|  | Output | Hidden size of stc and func: 64 | |
| Output layer | Hidden layer 1 | Units: 64, Activation: tanh | |
|  | Hidden layer 2 | Units: 16, Activation: tanh | |
|  | Hidden layer 3 | Units: 1, Activation: sigmoid | |
| Batch_size | 5 | | |
| Learning_rate | Pre-training | 0.001 | |
|  | Fine-tuning | Assembler | 0.001 |
|  |  | Chaperone | 0.0005 |
|  |  | Display-site | 0.0001 |
|  |  | Effector | 0.0001 |
|  |  | Scavenger | 0.0005 |
