# Supplementary material for: DMFpred: Predicting protein disorder molecular functions based on protein cubic language model
Source: PLoS Comput Biol. 2022 Oct 31;18(10):e1010668. doi: 10.1371/journal.pcbi.1010668 (PMC9674156; doi:10.1371/journal.pcbi.1010668)
Supplement: S2 Table — The thresholds were selected according to the highest MCC values in the validation set. (DOCX) [file pcbi.1010668.s004.docx]

**Table S2.** The thresholds used for binary results of DMFpred.

| **Function** | Assembler | Chaperone | Display-site | Effector | Scavenger |
| --- | --- | --- | --- | --- | --- |
| **Threshold** | 0.085 | 0.008 | 0.082 | 0.082 | 0.01 |

* The thresholds were selected according to the highest MCC values in the validation set.
