# Supplementary material for: DMFpred: Predicting protein disorder molecular functions based on protein cubic language model
Source: PLoS Comput Biol. 2022 Oct 31;18(10):e1010668. doi: 10.1371/journal.pcbi.1010668 (PMC9674156; doi:10.1371/journal.pcbi.1010668)
Supplement: S3 Table — (DOCX) [file pcbi.1010668.s005.docx]

**Table S3.** The predictive performance of DMFpred on the TEST-2 independent test set.

| **Function** | **AUC** | **Sn** | **Sp** | **ACC** | **BACC** | **MCC** |
| --- | --- | --- | --- | --- | --- | --- |
| Assembler | 0.658 | 0.388 | 0.852 | 0.788 | 0.620 | 0.212 |
| Chaperone | 0.810 | 0.725 | 0.934 | 0.932 | 0.830 | 0.226 |
| Display-site | 0.757 | 0.247 | 0.971 | 0.949 | 0.609 | 0.203 |
| Effector | 0.779 | 0.666 | 0.745 | 0.743 | 0.706 | 0.144 |
| Scavenger | 0.743 | 0.662 | 0.645 | 0.645 | 0.654 | 0.063 |
