# Supplementary material for: DMFpred: Predicting protein disorder molecular functions based on protein cubic language model
Source: PLoS Comput Biol. 2022 Oct 31;18(10):e1010668. doi: 10.1371/journal.pcbi.1010668 (PMC9674156; doi:10.1371/journal.pcbi.1010668)
Supplement: S1 Data — (DOCX) [file pcbi.1010668.s006.docx]

# The molecular function benchmark dataset.

Training set in page 1, evaluation set in page 394, independent test set in page 513.

Line 1: >Protein ID

Line 2: Protein sequence in one-letter amino acid encoding

Line 3: Annotations of disordered assembler functional residues

Line 4: Annotations of disordered chaperone functional residues

Line 5: Annotations of disordered display site functional residues

Line 6: Annotations of disordered effector functional residues

Line 7: Annotations of disordered scavenger functional residues

# Training set (324 proteins)

>DP02656

MLALLCSCLLLAAGASDAWTGEDSAEPNSDSAEWIRDMYAKVTEIWQEVMQRRDDDGALHAACQVQPSATLDAAQPRVTGVVLFRQLAPRAKLDAFFALEGFPTEPNSSSRAIHVHQFGDLSQGCESTGPHYNPLAVPHPQHPGDFGNFAVRDGSLWRYRAGLAASLAGPHSIVGRAVVVHAGEDDLGRGGNQASVENGNAGRRLACCVVGVCGPGLWERQAREHSERKKRRRESECKAA

000000000000000000111111111111111111111111111111111111000000000000000000000000000000000000000000000000000000000000000000000000000000000000000000000000000000000000000000000000000000000000000000000000000000000000000000000000000000000000000000

000000000000000000000000000000000000000000000000000000000000000000000000000000000000000000000000000000000000000000000000000000000000000000000000000000000000000000000000000000000000000000000000000000000000000000000000000000000000000000000000

000000000000000000000000000000000000000000000000000000000000000000000000000000000000000000000000000000000000000000000000000000000000000000000000000000000000000000000000000000000000000000000000000000000000000000000000000000000000000000000000

000000000000000000000000000000000000000000000000000000000000000000000000000000000000000000000000000000000000000000000000000000000000000000000000000000000000000000000000000000000000000000000000000000000000000000000000000000000000000000000000

000000000000000000000000000000000000000000000000000000000000000000000000000000000000000000000000000000000000000000000000000000000000000000000000000000000000000000000000000000000000000000000000000000000000000000000000000000000000000000000000

>DP02635

MQRDCIMDYKESCPSVSIPSSDEHREKKKRFTVYKVLVSVGRSEWFVFRRYAEFDKLYNSLKKQFPAMALKIPAKRIFGDNFDPDFIKQRRAGLNEFIQNLVRYPELYNHPDVRAFLQMDSPRHQSDPSEDEDERSTSKPHSTSRNINLGPTGNPHAKPTDFDFLKVIGKGSFGKVLLAKRKLDGKFYAVKVLQKKIVLNRKEQKHIMAERNVLLKNVKHPFLVGLHYSFQTTEKLYFVLDFVNGGELFFHLQRERSFPEPRARFYAAEIASALGYLHSIKIVYRDLKPENILLDSMGHVVLTDFGLCKEGIAISDTTTTFCGTPEYLAPEVIRKQPYDNTVDWWCLGAVLYEMLYGLPPFYCRDVAEMYDNILHKPLNLRPGVSLTAWSILEELLEKNRQNRLGAKEDFLEIQNHPFFESLSWTDLVQKKIPPPFNPNVAGPDDIRNFDAVFTEETVPYSVCVSSDYSIVNASVLEADDAFVGFSYAPPSEDLFL

0000000000000000000000000000000000000000000000000000000000000000000000000000000000000000000000000000000000000000000000000000011111111111111111111111111111111111000000000000000000000000000000000000000000000000000000000000000000000000000000000000000000000000000000000000000000000000000000000000000000000000000000000000000000000000000000000000000000000000000000000000000000000000000000000000000000000000000000000000000000000000000000000000000000000000000000000000000000000000000000000000000000000000

0000000000000000000000000000000000000000000000000000000000000000000000000000000000000000000000000000000000000000000000000000000000000000000000000000000000000000000000000000000000000000000000000000000000000000000000000000000000000000000000000000000000000000000000000000000000000000000000000000000000000000000000000000000000000000000000000000000000000000000000000000000000000000000000000000000000000000000000000000000000000000000000000000000000000000000000000000000000000000000000000000000000000000

0000000000000000000000000000000000000000000000000000000000000000000000000000000000000000000000000000000000000000000000000000000000000000000000000000000000000000000000000000000000000000000000000000000000000000000000000000000000000000000000000000000000000000000000000000000000000000000000000000000000000000000000000000000000000000000000000000000000000000000000000000000000000000000000000000000000000000000000000000000000000000000000000000000000000000000000000000000000000000000000000000000000000000

0000000000000000000000000000000000000000000000000000000000000000000000000000000000000000000000000000000000000000000000000000000000000000000000000000000000000000000000000000000000000000000000000000000000000000000000000000000000000000000000000000000000000000000000000000000000000000000000000000000000000000000000000000000000000000000000000000000000000000000000000000000000000000000000000000000000000000000000000000000000000000000000000000000000000000000000000000000000000000000000000000000000000000

0000000000000000000000000000000000000000000000000000000000000000000000000000000000000000000000000000000000000000000000000000000000000000000000000000000000000000000000000000000000000000000000000000000000000000000000000000000000000000000000000000000000000000000000000000000000000000000000000000000000000000000000000000000000000000000000000000000000000000000000000000000000000000000000000000000000000000000000000000000000000000000000000000000000000000000000000000000000000000000000000000000000000000

>DP01076

MFGNNRPMFGGSNLSFGSNTSSFGGQQSQQPNSLFGNSNNNNNSTSNNAQSGFGGFTSAAGSNSNSLFGNNNTQNNGAFGQSMGATQNSPFGSLNSSNASNGNTFGGSSSMGSFGGNTNNAFNNNSNSTNSPFGFNKPNTGGTLFGSQNNNSAGTSSLFGGQSTSTTGTFGNTGSSFGTGLNGNGSNIFGAGNNSQSNTTGSLFGNQQSSAFGTNNQQGSLFGQQSQNTNNAFGNQNQLGGSSFGSKPVGSGSLFGQSNNTLGNTTNNRNGLFGQMNSSNQGSSNSGLFGQNSMNSSTQGVFGQNNNQMQINGNNNNSLFGKANTFSNSASGGLFGQNNQQQGSGLFGQNSQTSGSSGLFGQNNQKQPNTFTQSNTGIGLFGQNNNQQQQSTGLFGAKPAGTTGSLFGGNSSTQPNSLFGTTNVPTSNTQSQQGNSLFGATKLTNMPFGGNPTANQSGSGNSLFGTKPASTTGSLFGNNTASTTVPSTNGLFGNNANNSTSTTNTGLFGAKPDSQSKPALGGGLFGNSNSNSSTIGQNKPVFGGTTQNTGLFGATGTNSSAVGSTGKLFGQNNNTLNVGTQNVPPVNNTTQNALLGTTAVPSLQQAPVTNEQLFSKISIPNSITNPVKATTSKVNADMKRNSSLTSAYRLAPKPLFAPSSNGDAKFQKWGKTLERSDRGSSTSNSITDPESSYLNSNDLLFDPDRRYLKHLVIKNNKNLNVINHNDDEASKVKLVTFTTESASKDDQASSSIAASKLTEKAHSPQTDLKDDHDESTPDPQSKSPNGSTSIPMIENEKISSKVPGLLSNDVTFFKNNYYISPSIETLGNKSLIELRKINNLVIGHRNYGKVEFLEPVDLLNTPLDTLCGDLVTFGPKSCSIYENCSIKPEKGEGINVRCRVTLYSCFPIDKETRKPIKNITHPLLKRSIAKLKENPVYKFESYDPVTGTYSYTIDHPVLT

11111111111111111111111111111111111111111111111111111111111111111111111111111111111111111111111111111111111111111111111111111111111111111111111111111111111111111111111111111111111111111111111111111111111111111111111111111111111111111111111111111111111111111111111111111111111111111111111111111111111111111111111111111111111111111111111111111111111111111111111111111111111111111111111111111111111111111111111111111111111111111111111111111111111111111111111111111111111111111111111111111111111111111111111111111111111111111111111111111111111111111111111111111111111111111111111111111111111111111111111111111111111111111111111111111111111111110000000000000000000000000000000000000000000000000000000000000000000000000000000000000000000000000000000000000000000000000000000000000000000000000000000000000000000000000000000000000000000000000000000000000000000000000000000000000000000000000000000000000000000000000000000000000000000000000000000000000000000000000000000

00000000000000000000000000000000000000000000000000000000000000000000000000000000000000000000000000000000000000000000000000000000000000000000000000000000000000000000000000000000000000000000000000000000000000000000000000000000000000000000000000000000000000000000000000000000000000000000000000000000000000000000000000000000000000000000000000000000000000000000000000000000000000000000000000000000000000000000000000000000000000000000000000000000000000000000000000000000000000000000000000000000000000000000000000000000000000000000000000000000000000000000000000000000000000000000000000000000000000000000000000000000000000000000000000000000000000000000000000000000000000000000000000000000000000000000000000000000000000000000000000000000000000000000000000000000000000000000000000000000000000000000000000000000000000000000000000000000000000000000000000000000000000000000000000000000000000000000000000000000000000000000000000000000000000000000000000000000000000000000000

00000000000000000000000000000000000000000000000000000000000000000000000000000000000000000000000000000000000000000000000000000000000000000000000000000000000000000000000000000000000000000000000000000000000000000000000000000000000000000000000000000000000000000000000000000000000000000000000000000000000000000000000000000000000000000000000000000000000000000000000000000000000000000000000000000000000000000000000000000000000000000000000000000000000000000000000000000000000000000000000000000000000000000000000000000000000000000000000000000000000000000000000000000000000000000000000000000000000000000000000000000000000000000000000000000000000000000000000000000000000000000000000000000000000000000000000000000000000000000000000000000000000000000000000000000000000000000000000000000000000000000000000000000000000000000000000000000000000000000000000000000000000000000000000000000000000000000000000000000000000000000000000000000000000000000000000000000000000000000000000

00000000000000000000000000000000000000000000000000000000000000000000000000000000000000000000000000000000000000000000000000000000000000000000000000000000000000000000000000000000000000000000000000000000000000000000000000000000000000000000000000000000000000000000000000000000000000000000000000000000000000000000000000000000000000000000000000000000000000000000000000000000000000000000000000000000000000000000000000000000000000000000000000000000000000000000000000000000000000000000000000000000000000000000000000000000000000000000000000000000000000000000000000000000000000000000000000000000000000000000000000000000000000000000000000000000000000000000000000000000000000000000000000000000000000000000000000000000000000000000000000000000000000000000000000000000000000000000000000000000000000000000000000000000000000000000000000000000000000000000000000000000000000000000000000000000000000000000000000000000000000000000000000000000000000000000000000000000000000000000000

00000000000000000000000000000000000000000000000000000000000000000000000000000000000000000000000000000000000000000000000000000000000000000000000000000000000000000000000000000000000000000000000000000000000000000000000000000000000000000000000000000000000000000000000000000000000000000000000000000000000000000000000000000000000000000000000000000000000000000000000000000000000000000000000000000000000000000000000000000000000000000000000000000000000000000000000000000000000000000000000000000000000000000000000000000000000000000000000000000000000000000000000000000000000000000000000000000000000000000000000000000000000000000000000000000000000000000000000000000000000000000000000000000000000000000000000000000000000000000000000000000000000000000000000000000000000000000000000000000000000000000000000000000000000000000000000000000000000000000000000000000000000000000000000000000000000000000000000000000000000000000000000000000000000000000000000000000000000000000000000

>DP00953

MAESPTEEAATAGAGAAGPGASSVAGVVGVSGSGGGFGPPFLPDVWAAAAAAGGAGGPGSGLAPLPGLPPSAAAHGAALLSHWDPTLSSDWDGERTAPQCLLRIKRDIMSIYKEPPPGMFVVPDTVDMTKIHALITGPFDTPYEGGFFLFVFRCPPDYPIHPPRVKLMTTGNNTVRFNPNFYRNGKVCLSILGTWTGPAWSPAQSISSVLISIQSLMTENPYHNEPGFEQERHPGDSKNYNECIRHETIRVAVCDMMEGKCPCPEPLRGVMEKSFLEYYDFYEVACKDRLHLQGQTMQDPFGEKRGHFDYQSLLMRLGLIRQKVLERLHNENAEMDSDSSSSGTETDLHGSLRV

111111111111111111111111111111111111111111111111111111111111111111111111111111111111111111111111111000000000000000000000000000000000000000000000000000000000000000000000000000000000000000000000000000000000000000000000000000000000000000000000000000000000000000000000000000000000000000000000000000000000000000000000000000000000000000000000000000000000000000

000000000000000000000000000000000000000000000000000000000000000000000000000000000000000000000000000000000000000000000000000000000000000000000000000000000000000000000000000000000000000000000000000000000000000000000000000000000000000000000000000000000000000000000000000000000000000000000000000000000000000000000000000000000000000000000000000000000000000000

000000000000000000000000000000000000000000000000000000000000000000000000000000000000000000000000000000000000000000000000000000000000000000000000000000000000000000000000000000000000000000000000000000000000000000000000000000000000000000000000000000000000000000000000000000000000000000000000000000000000000000000000000000000000000000000000000000000000000000

000000000000000000000000000000000000000000000000000000000000000000000000000000000000000000000000000000000000000000000000000000000000000000000000000000000000000000000000000000000000000000000000000000000000000000000000000000000000000000000000000000000000000000000000000000000000000000000000000000000000000000000000000000000000000000000000000000000000000000

000000000000000000000000000000000000000000000000000000000000000000000000000000000000000000000000000000000000000000000000000000000000000000000000000000000000000000000000000000000000000000000000000000000000000000000000000000000000000000000000000000000000000000000000000000000000000000000000000000000000000000000000000000000000000000000000000000000000000000

>DP00955

MLCCMRRTKQVEKNDEDQKIEQDGIKPEDKAHKAATKIQASFRGHITRKKLKGEKKGDAPAAEAEANEKDEAAVAEGTEKKEGEGSTPAEAAPGAGPKPEEKTGKAGETPSEEKKGEGAPDAATEQAAPQAPAPSEEKAGSAETESATKASTDNSPSSKAEDAPAKEEPKQADVPAAVTAAAATAPAAEDAAAMATAQPPTETAESSQAEEKIEAVDETKPKDSARQDEGKGEEREADQEHA

11111111111111111111111111111111111111111111111111111111111111111111111111111111111111111111111111111111111111111111111111111111111111111111111111111111111111111111111111111111111111111111111111111111111111111111111111111111111111111111111111

00000000000000000000000000000000000000000000000000000000000000000000000000000000000000000000000000000000000000000000000000000000000000000000000000000000000000000000000000000000000000000000000000000000000000000000000000000000000000000000000000

00000000000000000000000000000000000000000000000000000000000000000000000000000000000000000000000000000000000000000000000000000000000000000000000000000000000000000000000000000000000000000000000000000000000000000000000000000000000000000000000000

00000000000000000000000000000000000000000000000000000000000000000000000000000000000000000000000000000000000000000000000000000000000000000000000000000000000000000000000000000000000000000000000000000000000000000000000000000000000000000000000000

00000000000000000000000000000000000000000000000000000000000000000000000000000000000000000000000000000000000000000000000000000000000000000000000000000000000000000000000000000000000000000000000000000000000000000000000000000000000000000000000000

>DP01396

MGPWGWKLRWTVALLLAAAGTAVGDRCERNEFQCQDGKCISYKWVCDGSAECQDGSDESQETCLSVTCKSGDFSCGGRVNRCIPQFWRCDGQVDCDNGSDEQGCPPKTCSQDEFRCHDGKCISRQFVCDSDRDCLDGSDEASCPVLTCGPASFQCNSSTCIPQLWACDNDPDCEDGSDEWPQRCRGLYVFQGDSSPCSAFEFHCLSGECIHSSWRCDGGPDCKDKSDEENCAVATCRPDEFQCSDGNCIHGSRQCDREYDCKDMSDEVGCVNVTLCEGPNKFKCHSGECITLDKVCNMARDCRDWSDEPIKECGTNECLDNNGGCSHVCNDLKIGYECLCPDGFQLVAQRRCEDIDECQDPDTCSQLCVNLEGGYKCQCEEGFQLDPHTKACKAVGSIAYLFFTNRHEVRKMTLDRSEYTSLIPNLRNVVALDTEVASNRIYWSDLSQRMICSTQLDRAHGVSSYDTVISRDIQAPDGLAVDWIHSNIYWTDSVLGTVSVADTKGVKRKTLFRENGSKPRAIVVDPVHGFMYWTDWGTPAKIKKGGLNGVDIYSLVTENIQWPNGITLDLLSGRLYWVDSKLHSISSIDVNGGNRKTILEDEKRLAHPFSLAVFEDKVFWTDIINEAIFSANRLTGSDVNLLAENLLSPEDMVLFHNLTQPRGVNWCERTTLSNGGCQYLCLPAPQINPHSPKFTCACPDGMLLARDMRSCLTEAEAAVATQETSTVRLKVSSTAVRTQHTTTRPVPDTSRLPGATPGLTTVEIVTMSHQALGDVAGRGNEKKPSSVRALSIVLPIVLLVFLCLGVFLLWKNWRLKNINSINFDNPVYQKTTEDEVHICHNQDGYSYPSRQMVSLEDDVA

00000000000000000000000000000000000000000000000000000000000000000000000000000000000000000000000000000000000000000000000000000000000000000000000000000000000000000011111111111110000000000000000000000000000000000000000000000000000000000000000000000000000000000000000000000000000000000000000000000000000000000000000000000000000000000000000000000000000000000000000000000000000000000000000000000000000000000000000000000000000000000000000000000000000000000000000000000000000000000000000000000000000000000000000000000000000000000000000000000000000000000000000000000000000000000000000000000000000000000000000000000000000000000000000000000000000000000000000000000000000000000000000000000000000000000000000000000000000000000000000000000000000000000000000000000000000000000000000000000000000000000000000000000000000000000000000000000000000000000000000000000000000000000000

00000000000000000000000000000000000000000000000000000000000000000000000000000000000000000000000000000000000000000000000000000000000000000000000000000000000000000000000000000000000000000000000000000000000000000000000000000000000000000000000000000000000000000000000000000000000000000000000000000000000000000000000000000000000000000000000000000000000000000000000000000000000000000000000000000000000000000000000000000000000000000000000000000000000000000000000000000000000000000000000000000000000000000000000000000000000000000000000000000000000000000000000000000000000000000000000000000000000000000000000000000000000000000000000000000000000000000000000000000000000000000000000000000000000000000000000000000000000000000000000000000000000000000000000000000000000000000000000000000000000000000000000000000000000000000000000000000000000000000000000000000000000000000000

00000000000000000000000000000000000000000000000000000000000000000000000000000000000000000000000000000000000000000000000000000000000000000000000000000000000000000000000000000000000000000000000000000000000000000000000000000000000000000000000000000000000000000000000000000000000000000000000000000000000000000000000000000000000000000000000000000000000000000000000000000000000000000000000000000000000000000000000000000000000000000000000000000000000000000000000000000000000000000000000000000000000000000000000000000000000000000000000000000000000000000000000000000000000000000000000000000000000000000000000000000000000000000000000000000000000000000000000000000000000000000000000000000000000000000000000000000000000000000000000000000000000000000000000000000000000000000000000000000000000000000000000000000000000000000000000000000000000000000000000000000000000000000000

00000000000000000000000000000000000000000000000000000000000000000000000000000000000000000000000000000000000000000000000000000000000000000000000000000000000000000000000000000000000000000000000000000000000000000000000000000000000000000000000000000000000000000000000000000000000000000000000000000000000000000000000000000000000000000000000000000000000000000000000000000000000000000000000000000000000000000000000000000000000000000000000000000000000000000000000000000000000000000000000000000000000000000000000000000000000000000000000000000000000000000000000000000000000000000000000000000000000000000000000000000000000000000000000000000000000000000000000000000000000000000000000000000000000000000000000000000000000000000000000000000000000000000000000000000000000000000000000000000000000000000000000000000000000000000000000000000000000000000000000000000000000000000000

00000000000000000000000000000000000000000000000000000000000000000000000000000000000000000000000000000000000000000000000000000000000000000000000000000000000000000000000000000000000000000000000000000000000000000000000000000000000000000000000000000000000000000000000000000000000000000000000000000000000000000000000000000000000000000000000000000000000000000000000000000000000000000000000000000000000000000000000000000000000000000000000000000000000000000000000000000000000000000000000000000000000000000000000000000000000000000000000000000000000000000000000000000000000000000000000000000000000000000000000000000000000000000000000000000000000000000000000000000000000000000000000000000000000000000000000000000000000000000000000000000000000000000000000000000000000000000000000000000000000000000000000000000000000000000000000000000000000000000000000000000000000000000000

>DP02395

MDYMKLGLKSRKTGIDVKQDIPKDEYSMENIDDFFKDDETSLISMRRKSRRKSSLFLPSTLNGDTKNVLPPFLQSYKSQDDEVVQSPSGKGDGSRRSSLLSHQSNFLSPANDFEPIEEEPEQEENDIRGNDFATPITQKLSKPTYKRKYSTRYSLDTSESPSVRLTPDRITNKNVYSDVPDLVADEDDDDRVNTSLNTSDNALLEDELEDDGFIPESEEDGDYIESDSSLDSGSDSASDSDGDNTYQEVEEEAEVNTNDNEDDYIRRQASDVVRTDSIIDRNGLRKSTRVKVAPLQYWRNEKIVYKRKSNKPVLDIDKIVTYDESEDEEEILAAQRRKKQKKKPTPTRPYNYVPTGRPRGRPKKDPNAKENLIPEDPNEDIIERIESGGIENGEWLKHGILEANVKISDTKEETKDEIIAFAPNLSQTEQVKDTKDENFALEIMFDKHKEYFASGILKLPAISGQKKLSNSFRTYITFHVIQGIVEVTVCKNKFLSVKGSTFQIPAFNEYAIANRGNDEAKMFFVQVTVSEDANDDNDKELDSTFDTFG

000000000000000000000000000000000000000000000000000000000000000000000000000000000000000000000000000000000000000000000000000000000000000000000000000000000000000000000000000000000000000000000000000000000000000000000000000000000000000000000000000000000000000000000000000000000000000000000000000000000000000000000000000000000000000000111111111111111111111111111111111111111111111111111111111111111111111111111111111111111111111111111111111111000000000000000000000000000000000000000000000000000000000000000000000000000000000000000000000000000000000000000

000000000000000000000000000000000000000000000000000000000000000000000000000000000000000000000000000000000000000000000000000000000000000000000000000000000000000000000000000000000000000000000000000000000000000000000000000000000000000000000000000000000000000000000000000000000000000000000000000000000000000000000000000000000000000000000000000000000000000000000000000000000000000000000000000000000000000000000000000000000000000000000000000000000000000000000000000000000000000000000000000000000000000000000000000000000000000000000000000000000000000000000

000000000000000000000000000000000000000000000000000000000000000000000000000000000000000000000000000000000000000000000000000000000000000000000000000000000000000000000000000000000000000000000000000000000000000000000000000000000000000000000000000000000000000000000000000000000000000000000000000000000000000000000000000000000000000000000000000000000000000000000000000000000000000000000000000000000000000000000000000000000000000000000000000000000000000000000000000000000000000000000000000000000000000000000000000000000000000000000000000000000000000000000

000000000000000000000000000000000000000000000000000000000000000000000000000000000000000000000000000000000000000000000000000000000000000000000000000000000000000000000000000000000000000000000000000000000000000000000000000000000000000000000000000000000000000000000000000000000000000000000000000000000000000000000000000000000000000000000000000000000000000000000000000000000000000000000000000000000000000000000000000000000000000000000000000000000000000000000000000000000000000000000000000000000000000000000000000000000000000000000000000000000000000000000

000000000000000000000000000000000000000000000000000000000000000000000000000000000000000000000000000000000000000000000000000000000000000000000000000000000000000000000000000000000000000000000000000000000000000000000000000000000000000000000000000000000000000000000000000000000000000000000000000000000000000000000000000000000000000000000000000000000000000000000000000000000000000000000000000000000000000000000000000000000000000000000000000000000000000000000000000000000000000000000000000000000000000000000000000000000000000000000000000000000000000000000

>DP01271

MAVNVYSTSVTSDNLSRHDMLAWINESLQLNLTKIEQLCSGAAYCQFMDMLFPGSIALKKVKFQAKLEHEYIQNFKILQAGFKRMGVDKIIPVDKLVKGKFQDNFEFVQWFKKFFDANYDGKDYDPVAARQGQETAVAPSLVAPALNKPKKPLTSSSAAPQRPISTQRTAAAPKAGPGVVRKNPGVGNGDDEAAELMQQVNVLKLTVEDLEKERDFYFGKLRNIELICQENEGENDPVLQRIVDILYATDEGFVIPDEGGPQEEQEEY

0000000000000000000000000000000000000000000000000000000000000000000000000000000000000000000000000000000000000000000000000000000000000000000000000000000000000000000000000000000000000000000000000000000000000000000000000000000000000000000000000000000001111111100000000000

0000000000000000000000000000000000000000000000000000000000000000000000000000000000000000000000000000000000000000000000000000000000000000000000000000000000000000000000000000000000000000000000000000000000000000000000000000000000000000000000000000000000000000000000000000

0000000000000000000000000000000000000000000000000000000000000000000000000000000000000000000000000000000000000000000000000000000000000000000000000000000000000000000000000000000000000000000000000000000000000000000000000000000000000000000000000000000000000000000000000000

0000000000000000000000000000000000000000000000000000000000000000000000000000000000000000000000000000000000000000000000000000000000000000000000000000000000000000000000000000000000000000000000000000000000000000000000000000000000000000000000000000000000000000000000000000

0000000000000000000000000000000000000000000000000000000000000000000000000000000000000000000000000000000000000000000000000000000000000000000000000000000000000000000000000000000000000000000000000000000000000000000000000000000000000000000000000000000000000000000000000000

>DP01078

MSSLKDEVPTETSEDFGFKFLGQKQILPSFNEKLPFASLQNLDISNSKSLFVAASGSKAVVGELQLLRDHITSDSTPLTFKWEKEIPDVIFVCFHGDQVLVSTRNALYSLDLEELSEFRTVTSFEKPVFQLKNVNNTLVILNSVNDLSALDLRTKSTKQLAQNVTSFDVTNSQLAVLLKDRSFQSFAWRNGEMEKQFEFSLPSELEELPVEEYSPLSVTILSPQDFLAVFGNVISETDDEVSYDQKMYIIKHIDGSASFQETFDITPPFGQIVRFPYMYKVTLSGLIEPDANVNVLASSCSSEVSIWDSKQVIEPSQDSERAVLPISEETDKDTNPIGVAVDVVTSGTILEPCSGVDTIERLPLVYILNNEGSLQIVGLFHVAAIKSGHYSINLESLEHEKSLSPTSEKIPIAGQEQEEKKKNNESSKALSENPFTSANTSGFTFLKTQPAAANSLQSQSSSTFGAPSFGSSAFKIDLPSVSSTSTGVASSEQDATDPASAKPVFGKPAFGAIAKEPSTSEYAFGKPSFGAPSFGSGKSSVESPASGSAFGKPSFGTPSFGSGNSSVEPPASGSAFGKPSFGTPSFGSGNSSAEPPASGSAFGKPSFGTSAFGTASSNETNSGSIFGKAAFGSSSFAPANNELFGSNFTISKPTVDSPKEVDSTSPFPSSGDQSEDESKSDVDSSSTPFGTKPNTSTKPKTNAFDFGSSSFGSGFSKALESVGSDTTFKFGTQASPFSSQLGNKSPFSSFTKDDTENGSLSKGSTSEINDDNEEHESNGPNVSGNDLTDSTVEQTSSTRLPETPSDEDGEVVEEEAQKSPIGKLTETIKKSANIDMAGLKNPVFGNHVKAKSESPFSAFATNITKPSSTTPAFSFGNSTMNKSNTSTVSPMEEADTKETSEKGPITLKSVENPFLPAKEERTGESSKKDHNDDPKDGYVSGSEISVRTSESAFDTTANEEIPKSQDVNNHEKSETDPKYSQHAVVDHDNKSKEMNETSKNNERSGQPNHGVQGDGIALKKDNEKENFDSNMAIKQFEDHQSSEEDASEKDSRQSSEVKESDDNMSLNSDRDESISESYDKLEDINTDELPHGGEAFKAREVSASADFDVQTSLEDNYAESGIQTDLSESSKENEVQTDAIPVKHNSTQTVKKEAVDNGLQTEPVETCNFSVQTFEGDENYLAEQCKPKQLKEYYTSAKVSNIPFVSQNSTLRLIESTFQTVEAEFTVLMENIRNMDTFFTDQSSIPLVKRTVRSINNLYTWRIPEAEILLNIQNNIKCEQMQITNANIQDLKEKVTDYVRKDIAQITEDVANAKEEYLFLMHFDDASSGYVKDLSTHQFRMQKTLRQKLFDVSAKINHTEELLNILKLFTVKNKRLDDNPLVAKLAKESLARDGLLKEIKLLREQVSRLQLEEKGKKASSFDASSSITKDMKGFKVVEVGLAMNTKKQIGDFFKNLNMAK

00000000000000000000000000000000000000000000000000000000000000000000000000000000000000000000000000000000000000000000000000000000000000000000000000000000000000000000000000000000000000000000000000000000000000000000000000000000000000000000000000000000000000000000000000000000000000000000000000000000000000000000000000000000000000000000000000000000000000000000000000000000000000000000000000000000000000000000000000000000000000000000000000000000111111111111111111111111111111111111111111111111111111111111111111111111111111111111111111111111111111111111111111111111111111111111111111111111111111111111111111111111111111111111111111111111111111111111111111111111111111111111111111111111111111111111111111111111111111111111111111111111111111111111111111111111111111111111111111111111111111111111111111111111111111111111111111111111111111111111111111111111111111111111111111111111111111111000000000000000000000000000000000000000000000000000000000000000000000000000000000000000000000000000000000000000000000000000000000000000000000000000000000000000000000000000000000000000000000000000000000000000000000000000000000000000000000000000000000000000000000000000000000000000000000000000000000000000000000000000000000000000000000000000000000000000000000000000000000000000000000000000000000000000000000000000000000000000000000000000000000000000000000000000000000000000000000000000000000000000000000000000000000000000000000000000000000000000000000000000000000000000000000000000

00000000000000000000000000000000000000000000000000000000000000000000000000000000000000000000000000000000000000000000000000000000000000000000000000000000000000000000000000000000000000000000000000000000000000000000000000000000000000000000000000000000000000000000000000000000000000000000000000000000000000000000000000000000000000000000000000000000000000000000000000000000000000000000000000000000000000000000000000000000000000000000000000000000000000000000000000000000000000000000000000000000000000000000000000000000000000000000000000000000000000000000000000000000000000000000000000000000000000000000000000000000000000000000000000000000000000000000000000000000000000000000000000000000000000000000000000000000000000000000000000000000000000000000000000000000000000000000000000000000000000000000000000000000000000000000000000000000000000000000000000000000000000000000000000000000000000000000000000000000000000000000000000000000000000000000000000000000000000000000000000000000000000000000000000000000000000000000000000000000000000000000000000000000000000000000000000000000000000000000000000000000000000000000000000000000000000000000000000000000000000000000000000000000000000000000000000000000000000000000000000000000000000000000000000000000000000000000000000000000000000000000000000000000000000000000000000000000000000000000000000000000000000000000000000000000000000000000000000000000000000000000000000000000000000000000000000000000000000000000000000000000000000000000

00000000000000000000000000000000000000000000000000000000000000000000000000000000000000000000000000000000000000000000000000000000000000000000000000000000000000000000000000000000000000000000000000000000000000000000000000000000000000000000000000000000000000000000000000000000000000000000000000000000000000000000000000000000000000000000000000000000000000000000000000000000000000000000000000000000000000000000000000000000000000000000000000000000000000000000000000000000000000000000000000000000000000000000000000000000000000000000000000000000000000000000000000000000000000000000000000000000000000000000000000000000000000000000000000000000000000000000000000000000000000000000000000000000000000000000000000000000000000000000000000000000000000000000000000000000000000000000000000000000000000000000000000000000000000000000000000000000000000000000000000000000000000000000000000000000000000000000000000000000000000000000000000000000000000000000000000000000000000000000000000000000000000000000000000000000000000000000000000000000000000000000000000000000000000000000000000000000000000000000000000000000000000000000000000000000000000000000000000000000000000000000000000000000000000000000000000000000000000000000000000000000000000000000000000000000000000000000000000000000000000000000000000000000000000000000000000000000000000000000000000000000000000000000000000000000000000000000000000000000000000000000000000000000000000000000000000000000000000000000000000000000000000000000

00000000000000000000000000000000000000000000000000000000000000000000000000000000000000000000000000000000000000000000000000000000000000000000000000000000000000000000000000000000000000000000000000000000000000000000000000000000000000000000000000000000000000000000000000000000000000000000000000000000000000000000000000000000000000000000000000000000000000000000000000000000000000000000000000000000000000000000000000000000000000000000000000000000000000000000000000000000000000000000000000000000000000000000000000000000000000000000000000000000000000000000000000000000000000000000000000000000000000000000000000000000000000000000000000000000000000000000000000000000000000000000000000000000000000000000000000000000000000000000000000000000000000000000000000000000000000000000000000000000000000000000000000000000000000000000000000000000000000000000000000000000000000000000000000000000000000000000000000000000000000000000000000000000000000000000000000000000000000000000000000000000000000000000000000000000000000000000000000000000000000000000000000000000000000000000000000000000000000000000000000000000000000000000000000000000000000000000000000000000000000000000000000000000000000000000000000000000000000000000000000000000000000000000000000000000000000000000000000000000000000000000000000000000000000000000000000000000000000000000000000000000000000000000000000000000000000000000000000000000000000000000000000000000000000000000000000000000000000000000000000000000000000000000

00000000000000000000000000000000000000000000000000000000000000000000000000000000000000000000000000000000000000000000000000000000000000000000000000000000000000000000000000000000000000000000000000000000000000000000000000000000000000000000000000000000000000000000000000000000000000000000000000000000000000000000000000000000000000000000000000000000000000000000000000000000000000000000000000000000000000000000000000000000000000000000000000000000000000000000000000000000000000000000000000000000000000000000000000000000000000000000000000000000000000000000000000000000000000000000000000000000000000000000000000000000000000000000000000000000000000000000000000000000000000000000000000000000000000000000000000000000000000000000000000000000000000000000000000000000000000000000000000000000000000000000000000000000000000000000000000000000000000000000000000000000000000000000000000000000000000000000000000000000000000000000000000000000000000000000000000000000000000000000000000000000000000000000000000000000000000000000000000000000000000000000000000000000000000000000000000000000000000000000000000000000000000000000000000000000000000000000000000000000000000000000000000000000000000000000000000000000000000000000000000000000000000000000000000000000000000000000000000000000000000000000000000000000000000000000000000000000000000000000000000000000000000000000000000000000000000000000000000000000000000000000000000000000000000000000000000000000000000000000000000000000000000000000

>DP02863

MKRAPVIPKHTLNTQPVEDTSLSTPAAPMVDSLIARVGVMARGNAITLPVCGRDVKFTLEVLRGDSVEKTSRVWSGNERDQELLTEDALDDLIPSFLLTGQQTPAFGRRVSGVIEIADGSRRRKAAALTESDYRVLVGELDDEQMAALSRLGNDYRPTSAYERGQRYASRLQNEFAGNISALADAENISRKIITRCINTAKLPKSVVALFSHPGELSARSGDALQKAFTDKEELLKQQASNLHEQKKAGVIFEAEEVITLLTSVLKTSSASRTSLSSRHQFAPGATVLYKGDKMVLNLDRSRVPTECIEKIEAILKELEKPAP

00000000000000000000000000000000000000000000000000000000000000000000000000000000000000000000000000000000000000000000000000000000000000000000000000000000000000000000000000000000000000000000000000000000000000000000000000000000000000000000000000000000000000000000000000000000001111111111111111111111111111111111111111111111111

00000000000000000000000000000000000000000000000000000000000000000000000000000000000000000000000000000000000000000000000000000000000000000000000000000000000000000000000000000000000000000000000000000000000000000000000000000000000000000000000000000000000000000000000000000000000000000000000000000000000000000000000000000000000

00000000000000000000000000000000000000000000000000000000000000000000000000000000000000000000000000000000000000000000000000000000000000000000000000000000000000000000000000000000000000000000000000000000000000000000000000000000000000000000000000000000000000000000000000000000000000000000000000000000000000000000000000000000000

00000000000000000000000000000000000000000000000000000000000000000000000000000000000000000000000000000000000000000000000000000000000000000000000000000000000000000000000000000000000000000000000000000000000000000000000000000000000000000000000000000000000000000000000000000000000000000000000000000000000000000000000000000000000

00000000000000000000000000000000000000000000000000000000000000000000000000000000000000000000000000000000000000000000000000000000000000000000000000000000000000000000000000000000000000000000000000000000000000000000000000000000000000000000000000000000000000000000000000000000000000000000000000000000000000000000000000000000000

>DP02761

MNRQSGVNAGVQNNPPSRVVYLGSIPYDQTEEQILDLCSNVGPVINLKMMFDPQTGRSKGYAFIEFRDLESSASAVRNLNGYQLGSRFLKCGYSSNSDISGVSQQQQQQYNNINGNNNNNGNNNNNSNGPDFQNSGNANFLSQKFPELPSGIDVNINMTTPAMMISSELAKKPKEVQLKFLQKFQEWTRAHPEDAVSLLELCPQLSFVTAELLLTNGICKVDDLIPLASRPQEEASATNNNSVNEVVDPAVLNKQKELLKQVLQLNDSQISILPDDERMAIWDLKQKALRGEFGAF

00000000000000000000000000000000000000000000000000000000000000000000000000000000000000000000000000000000000000000000000000000011111111111111111111111111111111111111111111111111111111111111111111111111111111111111111111111111111111110000000000000000000000000000000000000000000000000000000000000000

00000000000000000000000000000000000000000000000000000000000000000000000000000000000000000000000000000000000000000000000000000000000000000000000000000000000000000000000000000000000000000000000000000000000000000000000000000000000000000000000000000000000000000000000000000000000000000000000000000000

00000000000000000000000000000000000000000000000000000000000000000000000000000000000000000000000000000000000000000000000000000000000000000000000000000000000000000000000000000000000000000000000000000000000000000000000000000000000000000000000000000000000000000000000000000000000000000000000000000000

00000000000000000000000000000000000000000000000000000000000000000000000000000000000000000000000000000000000000000000000000000000000000000000000000000000000000000000000000000000000000000000000000000000000000000000000000000000000000000000000000000000000000000000000000000000000000000000000000000000

00000000000000000000000000000000000000000000000000000000000000000000000000000000000000000000000000000000000000000000000000000000000000000000000000000000000000000000000000000000000000000000000000000000000000000000000000000000000000000000000000000000000000000000000000000000000000000000000000000000

>DP00242

MTDKIRTLQGRVVSDKMEKSIVVAIERFVKHPIYGKFIKRTTKLHVHDENNECGIGDVVEIRECRPLSKTKSWTLVRVVEKAVL

111111111111111111111111111111111111111111111111111111111111111111111111111111111110

000000000000000000000000000000000000000000000000000000000000000000000000000000000000

000000000000000000000000000000000000000000000000000000000000000000000000000000000000

000000000000000000000000000000000000000000000000000000000000000000000000000000000000

000000000000000000000000000000000000000000000000000000000000000000000000000000000000

>DP02894

MPTVEELYRNYGILADATEQVGQHKDAYQVILDGVKGGTKEKRLAAQFIPKFFKHFPELADSAINAQLDLCEDEDVSIRRQAIKELPQFATGENLPRVADILTQLLQTDDSAEFNLVNNALLSIFKMDAKGTLGGLFSQILQGEDIVRERAIKFLSTKLKTLPDEVLTKEVEELILTESKKVLEDVTGEEFVLFMKILSGLKSLQTVSGRQQLVELVAEQADLEQTFNPSDPDCVDRLLQCTRQAVPLFSKNVHSTRFVTYFCEQVLPNLGTLTTPVEGLDIQLEVLKLLAEMSSFCGDMEKLETNLRKLFDKLLEYMPLPPEEAENGENAGNEEPKLQFSYVECLLYSFHQLGRKLPDFLTAKLNAEKLKDFKIRLQYFARGLQVYIRQLRLALQGKTGEALKTEENKIKVVALKITNNINVLIKDLFHIPPSYKSTVTLSWKPVQKVEIGQKRASEDTTSGSPPKKSSAGPKRDARQIYNPPSGKYSSNLGNFNYERSLQGK

000000000000000000000000000000000000000000000000000000000000000000000000000000000000000000000000000000000000000000000000000000000000000000000000000000000000000000000000000000000000000000000000000000000000000000000000000000000000000000000000000000000000000000000000000000000000000000000000000000000000000000000000000000000000000000000000000000000000000000000000000000000000000000000000000000000000000000000000000000000000000000000000000000000000000000000011111111111111111111100000000000000000000000000000

000000000000000000000000000000000000000000000000000000000000000000000000000000000000000000000000000000000000000000000000000000000000000000000000000000000000000000000000000000000000000000000000000000000000000000000000000000000000000000000000000000000000000000000000000000000000000000000000000000000000000000000000000000000000000000000000000000000000000000000000000000000000000000000000000000000000000000000000000000000000000000000000000000000000000000000000000000000000000000000000000000000000000000000000

000000000000000000000000000000000000000000000000000000000000000000000000000000000000000000000000000000000000000000000000000000000000000000000000000000000000000000000000000000000000000000000000000000000000000000000000000000000000000000000000000000000000000000000000000000000000000000000000000000000000000000000000000000000000000000000000000000000000000000000000000000000000000000000000000000000000000000000000000000000000000000000000000000000000000000000000000000000000000000000000000000000000000000000000

000000000000000000000000000000000000000000000000000000000000000000000000000000000000000000000000000000000000000000000000000000000000000000000000000000000000000000000000000000000000000000000000000000000000000000000000000000000000000000000000000000000000000000000000000000000000000000000000000000000000000000000000000000000000000000000000000000000000000000000000000000000000000000000000000000000000000000000000000000000000000000000000000000000000000000000000000000000000000000000000000000000000000000000000

000000000000000000000000000000000000000000000000000000000000000000000000000000000000000000000000000000000000000000000000000000000000000000000000000000000000000000000000000000000000000000000000000000000000000000000000000000000000000000000000000000000000000000000000000000000000000000000000000000000000000000000000000000000000000000000000000000000000000000000000000000000000000000000000000000000000000000000000000000000000000000000000000000000000000000000000000000000000000000000000000000000000000000000000

>DP02295

MTTSRKSHAKDKKAGGEQDLADLKFRYDLLTNELFHLREFVSLVDYDPTHFNDSESFQKFLRETHLSLEERGEKFTDDVAKKGTNGDLTRRRRNLRTSTVVSSETTNEKKGDIELKLESIAPLVRNKCEELKYKLSDHSNRKSIVPQKRPIQHLKKREAAKSLKFKSERKENPLPLHEHIAEERYDHIAKVEEPSEAFTIKCPSDDSSFENTSEHYSDNFYFTTSSEEEDIKKKRGRKKKKPRIKLVVHPPKQTITNPLHVVKPGYESLHEYIASFKSLEDDLTLEEYNKYIDEQRRLLSRLKKGIENGALKYDKETDSLQPITSKEIKTIITYKPDPISYFYKQQDLQIHTDHLINQGIHMSKLFRSSTKARIARAKKVSQMIEQHFKHVAGAEERKAKEEERHKKSLARFAVQAVKKRWNMAEKAYRILRKDEEEQLKRIEGKQHLSKMLEKSTQLLEAQLNQVNDDGRSSTPSSDSNDVLSESDDDMDDELSTSSDEDEEVDADVGLENSPASTEATPTDESLNLIQLKEKYGHFNGSSTVYDSRNKDEKFPTLDKHESSSSESSVMTGEESSIYSSSENESQNENDRESDDKTPSVGLSALFGKGEESDGDLDLDDSEDFTVNSSSVEGEELEKDQVDNSAATFERAGDFVHTQNENRDDIKDVEEDAETKVQEEQLSVVDVPVPSLLRGNLRTYQKQGLNWLASLYNNHTNGILADEMGLGKTIQTISLLAYLACEKENWGPHLIVVPTSVLLNWEMEFKRFAPGFKVLTYYGSPQQRKEKRKGWNKPDAFHVCIVSYQLVVQDQHSFKRKRWQYMVLDEAHNIKNFRSTRWQALLNFNTQRRLLLTGTPLQNNLAELWSLLYFLMPQTVIDGKKVSGFADLDAFQQWFGRPVDKIIETGQNFGQDKETKKTVAKLHQVLRPYLLRRLKADVEKQMPAKYEHIVYCKLSKRQRFLYDDFMSRAQTKATLASGNFMSIVNCLMQLRKVCNHPNLFEVRPILTSFVLEHCVASDYKDVERTLLKLFKKNNQVNRVDLDFLNLVFTLNDKDLTSYHAEEISKLTCVKNFVEEVNKLRETNKQLQEEFGEASFLNFQDANQYFKYSNKQKLEGTVDMLNFLKMVNKLRCDRRPIFGKNLIDLLTKDRRVKYDKSSIIDNELIKPLQTRVLDNRKIIDTFAVLTPSAVSLDMRKLALGLNDDSSVGENTRLKVMQNCFEVSNPLHQLQTKLTIAFPDKSLLQYDCGKLQKLAILLQQLKDNGHRALIFTQMTKVLDVLEQFLNYHGYLYMRLDGATKIEDRQILTERFNTDSRITVFILSSRSGGLGINLTGADTVIFYDSDWNPAMDKQCQDRCHRIGQTRDVHIYRFVSEHTIESNILKKANQKRQLDNVVIQEGDFTTDYFSKLSVRDLLGSELPENASGGDKPLIADADVAAKDPRQLERLLAQAEDEDDVKAANLAMREVEIDNDDFDESTEKKAANEEEENHAELDEYEGTAHVDEYMIRFIANGYYY

00000000000000000000000000000000000000000000000000000000000000000000000000000000000000000000000000000000000000000000000000000000000000000000000000000000000000000000000000000000000000000000000000000000000000000000000000000000000000000000000000000000000000000000000000000000000000000000000000000000000000000000000000000000000000000000000000000000000000000000000000000000000000000000000000000000000000000000000000000000000000000000000000000000000000000000000000000000000000000000000000000000000000000000000000000000000000000000000000000000000000000000000000000000000000000000000000000000000000000000001111111111111111111111111111100000000000000000000000000000000000000000000000000000000000000000000000000000000000000000000000000000000000000000000000000000000000000000000000000000000000000000000000000000000000000000000000000000000000000000000000000000000000000000000000000000000000000000000000000000000000000000000000000000000000000000000000000000000000000000000000000000000000000000000000000000000000000000000000000000000000000000000000000000000000000000000000000000000000000000000000000000000000000000000000000000000000000000000000000000000000000000000000000000000000000000000000000000000000000000000000000000000000000000000000000000000000000000000000000000000000000000000000000000000000000000000000000000000000000000000000000000000000000000000000000000000000000000000000000000000000000000000000000000000000000000000000000000000000000000000000000000000000000000000000000000000000000000000000000000000000000000000000

00000000000000000000000000000000000000000000000000000000000000000000000000000000000000000000000000000000000000000000000000000000000000000000000000000000000000000000000000000000000000000000000000000000000000000000000000000000000000000000000000000000000000000000000000000000000000000000000000000000000000000000000000000000000000000000000000000000000000000000000000000000000000000000000000000000000000000000000000000000000000000000000000000000000000000000000000000000000000000000000000000000000000000000000000000000000000000000000000000000000000000000000000000000000000000000000000000000000000000000000000000000000000000000000000000000000000000000000000000000000000000000000000000000000000000000000000000000000000000000000000000000000000000000000000000000000000000000000000000000000000000000000000000000000000000000000000000000000000000000000000000000000000000000000000000000000000000000000000000000000000000000000000000000000000000000000000000000000000000000000000000000000000000000000000000000000000000000000000000000000000000000000000000000000000000000000000000000000000000000000000000000000000000000000000000000000000000000000000000000000000000000000000000000000000000000000000000000000000000000000000000000000000000000000000000000000000000000000000000000000000000000000000000000000000000000000000000000000000000000000000000000000000000000000000000000000000000000000000000000000000000000000000000000000000000000000000000000000000000000000000000000000000000000000000000000000000000000000000000000000000000000000000

00000000000000000000000000000000000000000000000000000000000000000000000000000000000000000000000000000000000000000000000000000000000000000000000000000000000000000000000000000000000000000000000000000000000000000000000000000000000000000000000000000000000000000000000000000000000000000000000000000000000000000000000000000000000000000000000000000000000000000000000000000000000000000000000000000000000000000000000000000000000000000000000000000000000000000000000000000000000000000000000000000000000000000000000000000000000000000000000000000000000000000000000000000000000000000000000000000000000000000000000000000000000000000000000000000000000000000000000000000000000000000000000000000000000000000000000000000000000000000000000000000000000000000000000000000000000000000000000000000000000000000000000000000000000000000000000000000000000000000000000000000000000000000000000000000000000000000000000000000000000000000000000000000000000000000000000000000000000000000000000000000000000000000000000000000000000000000000000000000000000000000000000000000000000000000000000000000000000000000000000000000000000000000000000000000000000000000000000000000000000000000000000000000000000000000000000000000000000000000000000000000000000000000000000000000000000000000000000000000000000000000000000000000000000000000000000000000000000000000000000000000000000000000000000000000000000000000000000000000000000000000000000000000000000000000000000000000000000000000000000000000000000000000000000000000000000000000000000000000000000000000000000000

00000000000000000000000000000000000000000000000000000000000000000000000000000000000000000000000000000000000000000000000000000000000000000000000000000000000000000000000000000000000000000000000000000000000000000000000000000000000000000000000000000000000000000000000000000000000000000000000000000000000000000000000000000000000000000000000000000000000000000000000000000000000000000000000000000000000000000000000000000000000000000000000000000000000000000000000000000000000000000000000000000000000000000000000000000000000000000000000000000000000000000000000000000000000000000000000000000000000000000000000000000000000000000000000000000000000000000000000000000000000000000000000000000000000000000000000000000000000000000000000000000000000000000000000000000000000000000000000000000000000000000000000000000000000000000000000000000000000000000000000000000000000000000000000000000000000000000000000000000000000000000000000000000000000000000000000000000000000000000000000000000000000000000000000000000000000000000000000000000000000000000000000000000000000000000000000000000000000000000000000000000000000000000000000000000000000000000000000000000000000000000000000000000000000000000000000000000000000000000000000000000000000000000000000000000000000000000000000000000000000000000000000000000000000000000000000000000000000000000000000000000000000000000000000000000000000000000000000000000000000000000000000000000000000000000000000000000000000000000000000000000000000000000000000000000000000000000000000000000000000000000000000000

00000000000000000000000000000000000000000000000000000000000000000000000000000000000000000000000000000000000000000000000000000000000000000000000000000000000000000000000000000000000000000000000000000000000000000000000000000000000000000000000000000000000000000000000000000000000000000000000000000000000000000000000000000000000000000000000000000000000000000000000000000000000000000000000000000000000000000000000000000000000000000000000000000000000000000000000000000000000000000000000000000000000000000000000000000000000000000000000000000000000000000000000000000000000000000000000000000000000000000000000000000000000000000000000000000000000000000000000000000000000000000000000000000000000000000000000000000000000000000000000000000000000000000000000000000000000000000000000000000000000000000000000000000000000000000000000000000000000000000000000000000000000000000000000000000000000000000000000000000000000000000000000000000000000000000000000000000000000000000000000000000000000000000000000000000000000000000000000000000000000000000000000000000000000000000000000000000000000000000000000000000000000000000000000000000000000000000000000000000000000000000000000000000000000000000000000000000000000000000000000000000000000000000000000000000000000000000000000000000000000000000000000000000000000000000000000000000000000000000000000000000000000000000000000000000000000000000000000000000000000000000000000000000000000000000000000000000000000000000000000000000000000000000000000000000000000000000000000000000000000000000000000000

>DP02946

MTAIFSLAINSNFVLANNDKPDASDDKYADYVVRLGSEHPLNHTQIIELSSAVSRAVLLSYPNIIDRYTAAATEYTVIDALFHSPTFRHIVSFGLHNQQENLGHIRYTNEYEINNNREDEFSLVSEVSYDDIKSSNAQQVPLVAFYEAREDRATGTPIVNMGVAPSLFSGRYSWWQEALIHEIVHHVTGSSDTHEENKQGPTEILAQMVAAELHWAIPTFKGYSDPARVEAIQERDFHSLLNMFQRHGSELGFLFTRLATIAKGKKASPDFGTLTSFCSEGISSFPKYPDHDDDFNGGGAFFLPSASADSSVECTFDVLNRIEPVDDSIKFEGGNLLIKNDFKNLNLRVAQLSFLNAKKGSGFYRKNWDSWKSWYQASSWKNGLNSGLYGYGHDESEGNLIYSPYGITFNDGSFSIGFSSRKHINDNTKDDNFVKLNNANWSSFYYAGQMFFDKNKRPVALVITEPLNAAFGAGWSYIYKDGKWHYEAQDDWDQRLFKDSTLSLDPHAPQFIN

000000000000000000000000000000000000000000000000000000000000000000000000000000000000000000000000000000000000000000000000000000000000000000000000000000000000000000000000000000000000000000000000000000000000000000000000000000000000000000000000000000000000000000000000000000000000011111111111111111111111111111111111110000000000000000000000000000000000000000000000000000000000000000000000000000000000000000000000000000000000000000000000000000000000000000000000000000000000000000000000000000000000000000000000000000000

000000000000000000000000000000000000000000000000000000000000000000000000000000000000000000000000000000000000000000000000000000000000000000000000000000000000000000000000000000000000000000000000000000000000000000000000000000000000000000000000000000000000000000000000000000000000000000000000000000000000000000000000000000000000000000000000000000000000000000000000000000000000000000000000000000000000000000000000000000000000000000000000000000000000000000000000000000000000000000000000000000000000000000000000000000000

000000000000000000000000000000000000000000000000000000000000000000000000000000000000000000000000000000000000000000000000000000000000000000000000000000000000000000000000000000000000000000000000000000000000000000000000000000000000000000000000000000000000000000000000000000000000000000000000000000000000000000000000000000000000000000000000000000000000000000000000000000000000000000000000000000000000000000000000000000000000000000000000000000000000000000000000000000000000000000000000000000000000000000000000000000000

000000000000000000000000000000000000000000000000000000000000000000000000000000000000000000000000000000000000000000000000000000000000000000000000000000000000000000000000000000000000000000000000000000000000000000000000000000000000000000000000000000000000000000000000000000000000000000000000000000000000000000000000000000000000000000000000000000000000000000000000000000000000000000000000000000000000000000000000000000000000000000000000000000000000000000000000000000000000000000000000000000000000000000000000000000000

000000000000000000000000000000000000000000000000000000000000000000000000000000000000000000000000000000000000000000000000000000000000000000000000000000000000000000000000000000000000000000000000000000000000000000000000000000000000000000000000000000000000000000000000000000000000000000000000000000000000000000000000000000000000000000000000000000000000000000000000000000000000000000000000000000000000000000000000000000000000000000000000000000000000000000000000000000000000000000000000000000000000000000000000000000000

>DP00956

MSNPPTPLLADYEWSGYLTGIGRAFDDGVKDLNKQLQDAQANLTKNPSDPTALANYQMIMSEYNLYRNAQSSAVKSMKDIDSSIVSNFR

00000000011111111111111111110000000000000000000000000000000000000000000000000000000000000

00000000000000000000000000000000000000000000000000000000000000000000000000000000000000000

00000000000000000000000000000000000000000000000000000000000000000000000000000000000000000

00000000000000000000000000000000000000000000000000000000000000000000000000000000000000000

00000000000000000000000000000000000000000000000000000000000000000000000000000000000000000

>DP00970

MDIRKIKKLIELVEESGISELEISEGEESVRISRAAPAASFPVMQQAYAAPMMQQPAQSNAAAPATVPSMEAPAAAEISGHIVRSPMVGTFYRTPSPDAKAFIEVGQKVNVGDTLCIVEAMKMMNQIEADKSGTVKAILVESGQPVEFDEPLVVIE

111111111111111111111111111111111111111111111111111111111111111111111111111110000000000000000000000000000000000000000000000000000000000000000000000000000000

000000000000000000000000000000000000000000000000000000000000000000000000000000000000000000000000000000000000000000000000000000000000000000000000000000000000

000000000000000000000000000000000000000000000000000000000000000000000000000000000000000000000000000000000000000000000000000000000000000000000000000000000000

000000000000000000000000000000000000000000000000000000000000000000000000000000000000000000000000000000000000000000000000000000000000000000000000000000000000

000000000000000000000000000000000000000000000000000000000000000000000000000000000000000000000000000000000000000000000000000000000000000000000000000000000000

>DP02415

MAEDVSSAAPSPRGCADGRDADPTEEQMAETERNDEEQFECQELLECQVQVGAPEEEEEEEEDAGLVAEAEAVAAGWMLDFLCLSLCRAFRDGRSEDFRRTRNSAEAIIHGLSSLTACQLRTIYICQFLTRIAAGKTLDAQFENDERITPLESALMIWGSIEKEHDKLHEEIQNLIKIQAIAVCMENGNFKEAEEVFERIFGDPNSHMPFKSKLLMIISQKDTFHSFFQHFSYNHMMEKIKSYVNYVLSEKSSTFLMKAAAKVVESKRTRTITSQDKPSGNDVEMETEANLDTRKSVSDKQSAVTESSEGTVSLLRSHKNLFLSKLQHGTQQQDLNKKERRVGTPQSTKKKKESRRATESRIPVSKSQPVTPEKHRARKRQAWLWEEDKNLRSGVRKYGEGNWSKILLHYKFNNRTSVMLKDRWRTMKKLKLISSDSED

0000000000000000000000000000000000000000000000000000000000000000000000000000000000000000000000000000000000000000000000000000000000000011111111111111000000000000000000000000000000000000000000000000000000000000000000000000000000000000000000000000000000000000000000000000000000000000000000000000000000000000000000000000000000000000000000000000000000000000000000000000000000000000000000000000000000000000000000000000000000000000000000000000000

0000000000000000000000000000000000000000000000000000000000000000000000000000000000000000000000000000000000000000000000000000000000000000000000000000000000000000000000000000000000000000000000000000000000000000000000000000000000000000000000000000000000000000000000000000000000000000000000000000000000000000000000000000000000000000000000000000000000000000000000000000000000000000000000000000000000000000000000000000000000000000000000000000000

0000000000000000000000000000000000000000000000000000000000000000000000000000000000000000000000000000000000000000000000000000000000000000000000000000000000000000000000000000000000000000000000000000000000000000000000000000000000000000000000000000000000000000000000000000000000000000000000000000000000000000000000000000000000000000000000000000000000000000000000000000000000000000000000000000000000000000000000000000000000000000000000000000000

0000000000000000000000000000000000000000000000000000000000000000000000000000000000000000000000000000000000000000000000000000000000000000000000000000000000000000000000000000000000000000000000000000000000000000000000000000000000000000000000000000000000000000000000000000000000000000000000000000000000000000000000000000000000000000000000000000000000000000000000000000000000000000000000000000000000000000000000000000000000000000000000000000000

0000000000000000000000000000000000000000000000000000000000000000000000000000000000000000000000000000000000000000000000000000000000000000000000000000000000000000000000000000000000000000000000000000000000000000000000000000000000000000000000000000000000000000000000000000000000000000000000000000000000000000000000000000000000000000000000000000000000000000000000000000000000000000000000000000000000000000000000000000000000000000000000000000000

>DP00194

MDLSQLTPRRPYLLRAFYEWLLDNQLTPHLVVDVTLPGVQVPMEYARDGQIVLNIAPRAVGNLELANDEVRFNARFGGIPRQVSVPLAAVLAIYARENGAGTMFEPEAAYDEDTSIMNDEEASADNETVMSVIDGDKPDHDDDTHPDDEPPQPPRGGRPALRVVK

000000000000000000000000000000000000000000000000000000000000000000000000000000000000000000000000000000000000001111111111111111111111111111111111111111111111111111111

000000000000000000000000000000000000000000000000000000000000000000000000000000000000000000000000000000000000000000000000000000000000000000000000000000000000000000000

000000000000000000000000000000000000000000000000000000000000000000000000000000000000000000000000000000000000000000000000000000000000000000000000000000000000000000000

000000000000000000000000000000000000000000000000000000000000000000000000000000000000000000000000000000000000000000000000000000000000000000000000000000000000000000000

000000000000000000000000000000000000000000000000000000000000000000000000000000000000000000000000000000000000000000000000000000000000000000000000000000000000000000000

>DP00230

MTTSLQDGQSAASRAAARDSPLAAQVCGAAQGRGDAHDLAPAPWLHARALLPLPDGTRGCAADRRKKKDLDVPEMPSIPNPFPELCCSPFTSVLSADLFPKANSRKKQVIKVYSEDETSRALDVPSDITARDVCQLLILKNHYIDDHSWTLFEHLPHIGVERTIEDHELVIEVLSNWGIEEENKLYFRKNYAKYEFFKNPMYFFPEHMVSFATETNGEISPTQILQMFLSSSTYPEIHGFLHAKEQGKKSWKKIYFFLRRSGLYFSTKGTSKEPRHLQFFSEFGNSDIYVSLAGKKKHGAPTNYGFCFKPNKAGGPRDLKMLCAEEEQSRTCWVTAIRLLKYGMQLYQNYMHPYQGRSGCSSQSISPMRSISENSLVAMDFSGQKSRVIENPTEALSVAVEEGLAWRKKGCLRLGTHGSPTASSQSSATNMAIHRSQPWFHHKISRDEAQRLIIQQGLVDGVFLVRDSQSNPKTFVLSMSHGQKIKHFQIIPVEDDGEMFHTLDDGHTRFTDLIQLVEFYQLNKGVLPCKLKHYCARIAL

000000000000000000000000000000000000000000000000000000000000000000000000000000000000000000000000000000000000000000000000000000000000000000000000000000000000000000000000000000000000000000000000000000000000000000000000000000000000000000000000000000000000000000000000000000000000000000000000000000000000000000000000000000000000000000000000000000000000000000000000111111111111111111111111111111111111111111111111111111111111111111111111111000000000000000000000000000000000000000000000000000000000000000000000000000000000000000000000000000000000

000000000000000000000000000000000000000000000000000000000000000000000000000000000000000000000000000000000000000000000000000000000000000000000000000000000000000000000000000000000000000000000000000000000000000000000000000000000000000000000000000000000000000000000000000000000000000000000000000000000000000000000000000000000000000000000000000000000000000000000000000000000000000000000000000000000000000000000000000000000000000000000000000000000000000000000000000000000000000000000000000000000000000000000000000000000000000000000000000000000000

000000000000000000000000000000000000000000000000000000000000000000000000000000000000000000000000000000000000000000000000000000000000000000000000000000000000000000000000000000000000000000000000000000000000000000000000000000000000000000000000000000000000000000000000000000000000000000000000000000000000000000000000000000000000000000000000000000000000000000000000000000000000000000000000000000000000000000000000000000000000000000000000000000000000000000000000000000000000000000000000000000000000000000000000000000000000000000000000000000000000

000000000000000000000000000000000000000000000000000000000000000000000000000000000000000000000000000000000000000000000000000000000000000000000000000000000000000000000000000000000000000000000000000000000000000000000000000000000000000000000000000000000000000000000000000000000000000000000000000000000000000000000000000000000000000000000000000000000000000000000000000000000000000000000000000000000000000000000000000000000000000000000000000000000000000000000000000000000000000000000000000000000000000000000000000000000000000000000000000000000000

000000000000000000000000000000000000000000000000000000000000000000000000000000000000000000000000000000000000000000000000000000000000000000000000000000000000000000000000000000000000000000000000000000000000000000000000000000000000000000000000000000000000000000000000000000000000000000000000000000000000000000000000000000000000000000000000000000000000000000000000000000000000000000000000000000000000000000000000000000000000000000000000000000000000000000000000000000000000000000000000000000000000000000000000000000000000000000000000000000000000

>DP02801

MELWGAYLLLCLFSLLTQVTTEPPTQKPKKIVNAKKDVVNTKMFEELKSRLDTLAQEVALLKEQQALQTVCLKGTKVHMKCFLAFTQTKTFHEASEDCISRGGTLGTPQTGSENDALYEYLRQSVGNEAEIWLGLNDMAAEGTWVDMTGARIAYKNWETEITAQPDGGKTENCAVLSGAANGKWFDKRCRDQLPYICQFGIV

1111111111111111111111111111111111111111111111111000000000000000000000000000000000000000000000000000000000000000000000000000000000000000000000000000000000000000000000000000000000000000000000000000000000

0000000000000000000000000000000000000000000000000000000000000000000000000000000000000000000000000000000000000000000000000000000000000000000000000000000000000000000000000000000000000000000000000000000000

0000000000000000000000000000000000000000000000000000000000000000000000000000000000000000000000000000000000000000000000000000000000000000000000000000000000000000000000000000000000000000000000000000000000

0000000000000000000000000000000000000000000000000000000000000000000000000000000000000000000000000000000000000000000000000000000000000000000000000000000000000000000000000000000000000000000000000000000000

0000000000000000000000000000000000000000000000000000000000000000000000000000000000000000000000000000000000000000000000000000000000000000000000000000000000000000000000000000000000000000000000000000000000

>DP02487

MSAIVGLCLLSEKVVLSRSLTDEVSKLYKLNRGNVKEPRKYATERMSTQSKPVALQVPVSTIILDYKNEDFIKQNPTYSAMDIIGSPSNTAPQTAFQSIMPSLSALFNTPFIQGAFRHRIISSMGPEISYLVMVIGPPSGFMDTPNVSSAQSSVHTVSNADVDLNDIIAINSTMAKSTKLVSASTLQAMLVNDVYDRCMDLDGILLSQALPFFRNYVNVQSKGSLPPAVAACLNTPIKELFSMGSGKREPLALEFRKDNEGQCIGIVLPKGHEGDTLSSRYPAVFINESEPFSDKERSELSELKRTDSDAYEKLYSETISKHVSDGSYGNRVIISHKMSRLSNGGVKIIGRFKISDFNTVKKNLSSRSGEIDSAKEQWEALSGNGLVTDSNISMLHDKILDTITSNKPGVVLRDGNKKSENIVVCFKNGFPNKKHSLLQLTKNGISVVSLDELTDAGILVESTGPDRVRRSPKVLANKLSSFKGRKVTLDVDNMSTEALIQKLSTL

00000000000000000000000000000000000000000000000000000000000000000000000000000000000000000000000000000000000000000000000000000000000000000000000000000000000000000000000000000000000000000000000000000000000000000000000000000000000000000000000000000000000000000000000000000000000000000000000011111111111100000000000000000000000000000000000000000000000000000000000000000000000000000000000000000000000000000000000000000000000000000000000000000000000000000000000000000000000000000000000000000000000000000000000000

00000000000000000000000000000000000000000000000000000000000000000000000000000000000000000000000000000000000000000000000000000000000000000000000000000000000000000000000000000000000000000000000000000000000000000000000000000000000000000000000000000000000000000000000000000000000000000000000000000000000000000000000000000000000000000000000000000000000000000000000000000000000000000000000000000000000000000000000000000000000000000000000000000000000000000000000000000000000000000000000000000000000000000000000000

00000000000000000000000000000000000000000000000000000000000000000000000000000000000000000000000000000000000000000000000000000000000000000000000000000000000000000000000000000000000000000000000000000000000000000000000000000000000000000000000000000000000000000000000000000000000000000000000000000000000000000000000000000000000000000000000000000000000000000000000000000000000000000000000000000000000000000000000000000000000000000000000000000000000000000000000000000000000000000000000000000000000000000000000000

00000000000000000000000000000000000000000000000000000000000000000000000000000000000000000000000000000000000000000000000000000000000000000000000000000000000000000000000000000000000000000000000000000000000000000000000000000000000000000000000000000000000000000000000000000000000000000000000000000000000000000000000000000000000000000000000000000000000000000000000000000000000000000000000000000000000000000000000000000000000000000000000000000000000000000000000000000000000000000000000000000000000000000000000000

00000000000000000000000000000000000000000000000000000000000000000000000000000000000000000000000000000000000000000000000000000000000000000000000000000000000000000000000000000000000000000000000000000000000000000000000000000000000000000000000000000000000000000000000000000000000000000000000000000000000000000000000000000000000000000000000000000000000000000000000000000000000000000000000000000000000000000000000000000000000000000000000000000000000000000000000000000000000000000000000000000000000000000000000000

>DP01852

MPGFNYGGKGDGTGWSSERGSGPEPGGGSHGNSGGHDRGDSSNVGNESVTVMKPGDSYNTPWGKVIINAAGQPTMNGTVMTADNSSMVPYGRGFTRVLNSLVNNPVSPAGQNGGKSPVQTAVENYLMVQSGNLPPGYWLSNGKVMTEVREERTSGGGGKNGNERTWTVKVPREVPQLTASYNEGMRIRQEAADRARAEANARALAEEEARAIASGKSKAEFDAGKRVEAAQAAINTAQLNVNNLSGAVSAANQVITQKQAEMTPLKNELAAANQRVQETLKFINDPIRSRIHFNMRSGLIRAQHNVDTKQNEINAAVANRDALNSQLSQANNILQNARNEKSAADAALSAATAQRLQAEAALRAAAEAAEKARQRQAEEAERQRQAMEVAEKAKDERELLEKTSELIAGMGDKIGEHLGDKYKAIAKDIADNIKNFQGKTIRSFDDAMASLNKITANPAMKINKADRDALVNAWKHVDAQDMANKLGNLSKAFKVADVVMKVEKVREKSIEGYETGNWGPLMLEVESWVLSGIASSVALGIFSATLGAYALSLGVPAIAVGIAGILLAAVVGALIDDKFADALNNEIIRPAH

1111111111111111111111111111111111111111111111111111111111111111111111111111111111111111111111111111111111111111111111111111111111111111111111111111111111111111111111111111000000000000000000000000000000000000000000000000000000000000000000000000000000000000000000000000000000000000000000000000000000000000000000000000000000000000000000000000000000000000000000000000000000000000000000000000000000000000000000000000000000000000000000000000000000000000000000000000000000000000000000000000000000000000000000000000000000000000000000000000000000000000000000000000000000000000000000000000000000000000

0000000000000000000000000000000000000000000000000000000000000000000000000000000000000000000000000000000000000000000000000000000000000000000000000000000000000000000000000000000000000000000000000000000000000000000000000000000000000000000000000000000000000000000000000000000000000000000000000000000000000000000000000000000000000000000000000000000000000000000000000000000000000000000000000000000000000000000000000000000000000000000000000000000000000000000000000000000000000000000000000000000000000000000000000000000000000000000000000000000000000000000000000000000000000000000000000000000000000000

0000000000000000000000000000000000000000000000000000000000000000000000000000000000000000000000000000000000000000000000000000000000000000000000000000000000000000000000000000000000000000000000000000000000000000000000000000000000000000000000000000000000000000000000000000000000000000000000000000000000000000000000000000000000000000000000000000000000000000000000000000000000000000000000000000000000000000000000000000000000000000000000000000000000000000000000000000000000000000000000000000000000000000000000000000000000000000000000000000000000000000000000000000000000000000000000000000000000000000

0000000000000000000000000000000000000000000000000000000000000000000000000000000000000000000000000000000000000000000000000000000000000000000000000000000000000000000000000000000000000000000000000000000000000000000000000000000000000000000000000000000000000000000000000000000000000000000000000000000000000000000000000000000000000000000000000000000000000000000000000000000000000000000000000000000000000000000000000000000000000000000000000000000000000000000000000000000000000000000000000000000000000000000000000000000000000000000000000000000000000000000000000000000000000000000000000000000000000000

0000000000000000000000000000000000000000000000000000000000000000000000000000000000000000000000000000000000000000000000000000000000000000000000000000000000000000000000000000000000000000000000000000000000000000000000000000000000000000000000000000000000000000000000000000000000000000000000000000000000000000000000000000000000000000000000000000000000000000000000000000000000000000000000000000000000000000000000000000000000000000000000000000000000000000000000000000000000000000000000000000000000000000000000000000000000000000000000000000000000000000000000000000000000000000000000000000000000000000

>DP00025

MKNNLRYGIRKHKLGAASVFLGTMIVVGMGQDKEAAASEQKTTTVEENGNSATDNKTSETQTTATNVNHIEETQSYNATVTEQPSNATQVTTEEAPKAVQAPQTAQPANIETVKEEVVKEEAKPQVKETTQSQDNSGDQRQVDLTPKKATQNQVAETQVEVAQPRTASESKPRVTRSADVAEAKEASNAKVETGTDVTSKVTVEIGSIEGHNNTNKVEPHAGQRAVLKYKLKFENGLHQGDYFDFTLSNNVNTHGVSTARKVPEIKNGSVVMATGEVLEGGKIRYTFTNDIEDKVDVTAELEINLFIDPKTVQTNGNQTITSTLNEEQTSKELDVKYKDGIGNYYANLNGSIETFNKANNRFSHVAFIKPNNGKTTSVTVTGTLMKGSNQNGNQPKVRIFEYLGNNEDIAKSVYANTTDTSKFKEVTSNMSGNLNLQNNGSYSLNIENLDKTYVVHYDGEYLNGTDEVDFRTQMVGHPEQLYKYYYDRGYTLTWDNGLVLYSNKANGNEKNGPIIQNNKFEYKEDTIKETLTGQYDKNLVTTVEEEYDSSTLDIDYHTAIDGGGGYVDGYIETIEETDSSAIDIDYHTAVDSEAGHVGGYTESSEESNPIDFEESTHENSKHHADVVEYEEDTNPGGGQVTTESNLVEFDEESTKGIVTGAVSDHTTVEDTKEYTTESNLIELVDELPEEHGQAQGPVEEITKNNHHISHSGLGTENGHGNYDVIEEIEENSHVDIKSELGYEGGQNSGNQSFEEDTEEDKPKYEQGGNIVDIDFDSVPQIHGQNKGNQSFEEDTEKDKPKYEHGGNIIDIDFDSVPHIHGFNKHTEIIEEDTNKDKPSYQFGGHNSVDFEEDTLPKVSGQNEGQQTIEEDTTPPIVPPTPPTPEVPSEPETPTPPTPEVPSEPETPTPPTPEVPSEPETPTPPTPEVPAEPGKPVPPAKEEPKKPSKPVEQGKVVTPVIEINEKVKAVAPTKKPQSKKSELPETGGEESTNKGMLFGGLFSILGLALLRRNKKNHKA

0000000000000000000000000000000000000000000000000000000000000000000000000000000000000000000000000000000000000000000000000000000000000000000000000000000000000000000000000000000000000000000000000000000000000000000000000000000000000000000000000000000000000000000000000000000000000000000000000000000000000000000000000000000000000000000000000000000000000000000000000000000000000000000000000000000000000000000000000000000000000000000000000000000000000000000000000000000000000000000000000000000000000000000000000000000000000000000000000000000000000000000000000000000000000000000000000000000000000000000000000000000000000000000000000000000000000000000000000000000000000000000000000000000000000000000000000000000000000000000000000000000000000000000000001111111111111111111111111111111111111111111111111111111111111111111111111111111111111111111111111111111111111111111111111111111111000000000000000000000000000000000000000000000000000000000000000000000000000000000000000000000000000000000000000000000000000000000000000000000000

0000000000000000000000000000000000000000000000000000000000000000000000000000000000000000000000000000000000000000000000000000000000000000000000000000000000000000000000000000000000000000000000000000000000000000000000000000000000000000000000000000000000000000000000000000000000000000000000000000000000000000000000000000000000000000000000000000000000000000000000000000000000000000000000000000000000000000000000000000000000000000000000000000000000000000000000000000000000000000000000000000000000000000000000000000000000000000000000000000000000000000000000000000000000000000000000000000000000000000000000000000000000000000000000000000000000000000000000000000000000000000000000000000000000000000000000000000000000000000000000000000000000000000000000000000000000000000000000000000000000000000000000000000000000000000000000000000000000000000000000000000000000000000000000000000000000000000000000000000000000000000000000000000000000000000000000000000000000000000000000000000000000000000000000000000000000000000000000000000000000

0000000000000000000000000000000000000000000000000000000000000000000000000000000000000000000000000000000000000000000000000000000000000000000000000000000000000000000000000000000000000000000000000000000000000000000000000000000000000000000000000000000000000000000000000000000000000000000000000000000000000000000000000000000000000000000000000000000000000000000000000000000000000000000000000000000000000000000000000000000000000000000000000000000000000000000000000000000000000000000000000000000000000000000000000000000000000000000000000000000000000000000000000000000000000000000000000000000000000000000000000000000000000000000000000000000000000000000000000000000000000000000000000000000000000000000000000000000000000000000000000000000000000000000000000000000000000000000000000000000000000000000000000000000000000000000000000000000000000000000000000000000000000000000000000000000000000000000000000000000000000000000000000000000000000000000000000000000000000000000000000000000000000000000000000000000000000000000000000000000000

0000000000000000000000000000000000000000000000000000000000000000000000000000000000000000000000000000000000000000000000000000000000000000000000000000000000000000000000000000000000000000000000000000000000000000000000000000000000000000000000000000000000000000000000000000000000000000000000000000000000000000000000000000000000000000000000000000000000000000000000000000000000000000000000000000000000000000000000000000000000000000000000000000000000000000000000000000000000000000000000000000000000000000000000000000000000000000000000000000000000000000000000000000000000000000000000000000000000000000000000000000000000000000000000000000000000000000000000000000000000000000000000000000000000000000000000000000000000000000000000000000000000000000000000000000000000000000000000000000000000000000000000000000000000000000000000000000000000000000000000000000000000000000000000000000000000000000000000000000000000000000000000000000000000000000000000000000000000000000000000000000000000000000000000000000000000000000000000000000000000

0000000000000000000000000000000000000000000000000000000000000000000000000000000000000000000000000000000000000000000000000000000000000000000000000000000000000000000000000000000000000000000000000000000000000000000000000000000000000000000000000000000000000000000000000000000000000000000000000000000000000000000000000000000000000000000000000000000000000000000000000000000000000000000000000000000000000000000000000000000000000000000000000000000000000000000000000000000000000000000000000000000000000000000000000000000000000000000000000000000000000000000000000000000000000000000000000000000000000000000000000000000000000000000000000000000000000000000000000000000000000000000000000000000000000000000000000000000000000000000000000000000000000000000000000000000000000000000000000000000000000000000000000000000000000000000000000000000000000000000000000000000000000000000000000000000000000000000000000000000000000000000000000000000000000000000000000000000000000000000000000000000000000000000000000000000000000000000000000000000000

>DP00541

MGKRLDLSTLTDEEAEHVWAVVQRDFDLRRREEERLQGLKGKIQKESSKRELLSDTAHLNETHCARCLQPYRLLLNSRRQCLECSLFVCKSCSHAHPEEQGWLCDPCHLARVVKIGSLEWYYQHVRARFKRFGSAKVIRSLCGRLQGGGGSEPSLEEGNGDSEQTDEDGDLDTEARDQPLNSKKKKRLLSFRDVDFEEDSDHLVQPCSQTLGLSSVPESAHSLQSLSGEPYSEDTTSLEPEGLEETGARALGCRPSPEVQPCSPLPSGEDAHAELDSPAASCKSAFGTTAMPGTDDVRGKHLPSQYLADVDTSDEDSIQGPRAASQHSKRRARTVPETQILELNKRMSAVEHLLVHLENTVLPPSAQEPTVETHPSADTEEETLRRRLEELTSNISGSSTSSEDETKPDGTFLGGSPKVCTDTGHMETQERNPRSPGNPARPTKSTDEELSEMEDRVAMTASEVQQAESEISDIESRIAALRAAGLTVKPSGKPRRKSGIPIFLPRVTEKLDRIPKTPPADPDDQAKMPKATTAVPSLLRRKYSPSSQGVDSGSFDRKSVYRGSLTQRNPNGRRGTARHIFAKPVMAQQP

00000000000000000000000000000000000000000000000000000000000000000000000000000000000000000000000000000000000000000000000000000000000000000000000000111111111111111111111111111111111111111111111111111111111111111111111111111111111111111111111111111111111111111111111111111111111111111111111111111111111111111111111111111111111111111111111111111111111111111111111111111111111111111111111111111111111111111110000000000000000000000000000000000000000000000000000000000000000000000000000000000000000000000000000000000000000000000000000000000000000000000000000000000000000000000000000000000000000000

00000000000000000000000000000000000000000000000000000000000000000000000000000000000000000000000000000000000000000000000000000000000000000000000000000000000000000000000000000000000000000000000000000000000000000000000000000000000000000000000000000000000000000000000000000000000000000000000000000000000000000000000000000000000000000000000000000000000000000000000000000000000000000000000000000000000000000000000000000000000000000000000000000000000000000000000000000000000000000000000000000000000000000000000000000000000000000000000000000000000000000000000000000000000000000000000000000000000000

00000000000000000000000000000000000000000000000000000000000000000000000000000000000000000000000000000000000000000000000000000000000000000000000000000000000000000000000000000000000000000000000000000000000000000000000000000000000000000000000000000000000000000000000000000000000000000000000000000000000000000000000000000000000000000000000000000000000000000000000000000000000000000000000000000000000000000000000000000000000000000000000000000000000000000000000000000000000000000000000000000000000000000000000000000000000000000000000000000000000000000000000000000000000000000000000000000000000000

00000000000000000000000000000000000000000000000000000000000000000000000000000000000000000000000000000000000000000000000000000000000000000000000000000000000000000000000000000000000000000000000000000000000000000000000000000000000000000000000000000000000000000000000000000000000000000000000000000000000000000000000000000000000000000000000000000000000000000000000000000000000000000000000000000000000000000000000000000000000000000000000000000000000000000000000000000000000000000000000000000000000000000000000000000000000000000000000000000000000000000000000000000000000000000000000000000000000000

00000000000000000000000000000000000000000000000000000000000000000000000000000000000000000000000000000000000000000000000000000000000000000000000000000000000000000000000000000000000000000000000000000000000000000000000000000000000000000000000000000000000000000000000000000000000000000000000000000000000000000000000000000000000000000000000000000000000000000000000000000000000000000000000000000000000000000000000000000000000000000000000000000000000000000000000000000000000000000000000000000000000000000000000000000000000000000000000000000000000000000000000000000000000000000000000000000000000000

>DP02733

MSTGGDFGNPLRKFKLVFLGEQSVGKTSLITRFMYDSFDNTYQATIGIDFLSKTMYLEDRTVRLQLWDTAGQERFRSLIPSYIRDSTVAVVVYDITNVNSFQQTTKWIDDVRTERGSDVIIMLVGNKTDLADKRQVSIEEGERKAKELNVMFIETSAKAGYNVKQLFRRVAAALPGMESTQDRSREDMIDIKLEKPQEQPVSEGGCSC

0000000000000000000000000000000000000000000000000000000000000000000000000000000000000000000000000000000000000000000000000000000000000000000000000000000000000000000000000000001111111111111111111111111111111100

0000000000000000000000000000000000000000000000000000000000000000000000000000000000000000000000000000000000000000000000000000000000000000000000000000000000000000000000000000000000000000000000000000000000000000

0000000000000000000000000000000000000000000000000000000000000000000000000000000000000000000000000000000000000000000000000000000000000000000000000000000000000000000000000000000000000000000000000000000000000000

0000000000000000000000000000000000000000000000000000000000000000000000000000000000000000000000000000000000000000000000000000000000000000000000000000000000000000000000000000000000000000000000000000000000000000

0000000000000000000000000000000000000000000000000000000000000000000000000000000000000000000000000000000000000000000000000000000000000000000000000000000000000000000000000000000000000000000000000000000000000000

>DP00958

MKLPVAQYSAPDGVEKSFAPIRDDPRYMTTEGRTTGPSDHVLNAGQIDRDKPSEPERTKDGSQLTYLGQLRTQLTGLQDDINEFLTGRMELAKNKKKAGADEKRIQEEINQLLDGGDGDEDAV

011111111111111111111000000000000000000000000000000000000000000011111111111111111111111111111111111111111111111110000000000

000000000000000000000000000000000000000000000000000000000000000000000000000000000000000000000000000000000000000000000000000

000000000000000000000000000000000000000000000000000000000000000000000000000000000000000000000000000000000000000000000000000

000000000000000000000000000000000000000000000000000000000000000000000000000000000000000000000000000000000000000000000000000

000000000000000000000000000000000000000000000000000000000000000000000000000000000000000000000000000000000000000000000000000

>DP01055

MSSLPFVFGAAASSRVVTAAAAKGTAETKQEKSFVDWLLGKITKEDQFYETDPILRGGDVKSSGSTSGKKGGTTSGKKGTVSIPSKKKNGNGGVFGGLFAKKD

1111111111111111111111111111111111111111111111111111111111111111111111111111111111111111111111111111111

0000000000000000000000000000000000000000000000000000000000000000000000000000000000000000000000000000000

0000000000000000000000000000000000000000000000000000000000000000000000000000000000000000000000000000000

0000000000000000000000000000000000000000000000000000000000000000000000000000000000000000000000000000000

0000000000000000000000000000000000000000000000000000000000000000000000000000000000000000000000000000000

>DP00223

MALRVTRNSKINAENKAKINMAGAKRVPTAPAATSKPGLRPRTALGDIGNKVSEQLQAKMPMKKEAKPSATGKVIDKKLPKPLEKVPMLVPVPVSEPVPEPEPEPEPEPVKEEKLSPEPILVDTASPSPMETSGCAPAEEDLCQAFSDVILAVNDVDAEDGADPNLCSEYVKDIYAYLRQLEEEQAVRPKYLLGREVTGNMRAILIDWLVQVQMKFRLLQETMYMTVSIIDRFMQNNCVPKKMLQLVGVTAMFIASKYEEMYPPEIGDFAFVTDNTYTKHQIRQMEMKILRALNFGLGRPLPLHFLRRASKIGEVDVEQHTLAKYLMELTMLDYDMVHFPPSQIAAGAFCLALKILDNGEWTPTLQHYLSYTEESLLPVMQHLAKNVVMVNQGLTKHMTVKNKYATSKHAKISTLPQLNSALVQDLAKAVAKV

1111111111111111111111111111111111111111111111111111111111111111111111111111110000000000000000000000000000000000000000000000000000000000000000000000000000000000000000000000000000000000000000000000000000000000000000000000000000000000000000000000000000000000000000000000000000000000000000000000000000000000000000000000000000000000000000000000000000000000000000000000000000000000000000000000000000000000000000000000000000000000000000000

0000000000000000000000000000000000000000000000000000000000000000000000000000000000000000000000000000000000000000000000000000000000000000000000000000000000000000000000000000000000000000000000000000000000000000000000000000000000000000000000000000000000000000000000000000000000000000000000000000000000000000000000000000000000000000000000000000000000000000000000000000000000000000000000000000000000000000000000000000000000000000000000000

0000000000000000000000000000000000000000000000000000000000000000000000000000000000000000000000000000000000000000000000000000000000000000000000000000000000000000000000000000000000000000000000000000000000000000000000000000000000000000000000000000000000000000000000000000000000000000000000000000000000000000000000000000000000000000000000000000000000000000000000000000000000000000000000000000000000000000000000000000000000000000000000000

0000000000000000000000000000000000000000000000000000000000000000000000000000000000000000000000000000000000000000000000000000000000000000000000000000000000000000000000000000000000000000000000000000000000000000000000000000000000000000000000000000000000000000000000000000000000000000000000000000000000000000000000000000000000000000000000000000000000000000000000000000000000000000000000000000000000000000000000000000000000000000000000000

0000000000000000000000000000000000000000000000000000000000000000000000000000000000000000000000000000000000000000000000000000000000000000000000000000000000000000000000000000000000000000000000000000000000000000000000000000000000000000000000000000000000000000000000000000000000000000000000000000000000000000000000000000000000000000000000000000000000000000000000000000000000000000000000000000000000000000000000000000000000000000000000000

>DP00899

MINMTKQVSKILAGLFTALFAGSLMASDAPAVGKDLTQAAENIPPAFHNAPRQGELPALNYVNQPPMVPHSVANYQVTKNVNQCLNCHSPENSRLSGATRISPTHFMDRDGKVGSSSSPRRYFCLQCHVSQANVDPIVPNDFKPMKGYGN

000000000000000000000000000111111111111111111111111111111111110000000000000000000000000000000000000000000000000000000000000000000011111111111111111111

000000000000000000000000000000000000000000000000000000000000000000000000000000000000000000000000000000000000000000000000000000000000000000000000000000

000000000000000000000000000000000000000000000000000000000000000000000000000000000000000000000000000000000000000000000000000000000000000000000000000000

000000000000000000000000000000000000000000000000000000000000000000000000000000000000000000000000000000000000000000000000000000000000000000000000000000

000000000000000000000000000000000000000000000000000000000000000000000000000000000000000000000000000000000000000000000000000000000000000000000000000000

>DP00552

MQKEQLSALMDGETLDSELLNELAHNPEMQKTWESYHLIRDSMRGDTPEVLHFDISSRVMAAIEEEPVRQPATLIPEAQPAPHQWQKMPFWQKVRPWAAQLTQMGVAACVSLAVIVGVQHYNGQSETSQQPETPVFNTLPMMGKASPVSLGVPSEATANNGQQQQVQEQRRRINAMLQDYELQRRLHSEQLQFEQAQTQQAAVQVPGIQTLGTQSQ

000000000000000000000000000000000000000000000000000000000000000000000000000000000000000000000000000000000000000000000000111111111111111111111111111111111111111111111111111111111111111111111111111111111111111111111111

000000000000000000000000000000000000000000000000000000000000000000000000000000000000000000000000000000000000000000000000000000000000000000000000000000000000000000000000000000000000000000000000000000000000000000000000

000000000000000000000000000000000000000000000000000000000000000000000000000000000000000000000000000000000000000000000000000000000000000000000000000000000000000000000000000000000000000000000000000000000000000000000000

000000000000000000000000000000000000000000000000000000000000000000000000000000000000000000000000000000000000000000000000000000000000000000000000000000000000000000000000000000000000000000000000000000000000000000000000

000000000000000000000000000000000000000000000000000000000000000000000000000000000000000000000000000000000000000000000000000000000000000000000000000000000000000000000000000000000000000000000000000000000000000000000000

>DP00221

MSQQHTLPVTLSPALSQELLKTVPPPVNTHQEQMKQPTPLPPPCQKVPVELPVEVPSKQEEKHMTAVKGLPEQECEQQQKEPQEQELQQQHWEQHEEYQKAENPEQQLKQEKTQRDQQLNKQLEEEKKLLDQQLDQELVKRDEQLGMKKEQLLELPEQQEGHLKHLEQQEGQLKHPEQQEGQLELPEQQEGQLELPEQQEGQLELPEQQEGQLELPEQQEGQLELPEQQEGQLELPQQQEGQLELSEQQEGQLELSEQQEGQLKHLEHQEGQLEVPEEQMGQLKYLEQQEGQLKHLDQQEKQPELPEQQMGQLKHLEQQEGQPKHLEQQEGQLEQLEEQEGQLKHLEQQEGQLEHLEHQEGQLGLPEQQVLQLKQLEKQQGQPKHLEEEEGQLKHLVQQEGQLKHLVQQEGQLEQQERQVEHLEQQVGQLKHLEEQEGQLKHLEQQQGQLEVPEQQVGQPKNLEQEEKQLELPEQQEGQVKHLEKQEAQLELPEQQVGQPKHLEQQEKHLEHPEQQDGQLKHLEQQEGQLKDLEQQKGQLEQPVFAPAPGQVQDIQPALPTKGEVLLPVEHQQQKQEVQWPPKHK

111111111111111111111111111111111111111111111111111111111111111111111111111111111111111111111111111111111111111111111111111111111111111111111111111111111111111111111111111111111111111111111111111111111111111111111111111111111111111111111111111111111111111111111111111111111111111111111111111111111111111111111111111111111111111111111111111111111111111111111111111111111111111111111111111111111111111111111111111111111111111111111111111111111111111111111111111111111111111111111111111111111111111111111111111111111111111111111111111111111111111111111111111111111111111111111111111111111

000000000000000000000000000000000000000000000000000000000000000000000000000000000000000000000000000000000000000000000000000000000000000000000000000000000000000000000000000000000000000000000000000000000000000000000000000000000000000000000000000000000000000000000000000000000000000000000000000000000000000000000000000000000000000000000000000000000000000000000000000000000000000000000000000000000000000000000000000000000000000000000000000000000000000000000000000000000000000000000000000000000000000000000000000000000000000000000000000000000000000000000000000000000000000000000000000000000

000000000000000000000000000000000000000000000000000000000000000000000000000000000000000000000000000000000000000000000000000000000000000000000000000000000000000000000000000000000000000000000000000000000000000000000000000000000000000000000000000000000000000000000000000000000000000000000000000000000000000000000000000000000000000000000000000000000000000000000000000000000000000000000000000000000000000000000000000000000000000000000000000000000000000000000000000000000000000000000000000000000000000000000000000000000000000000000000000000000000000000000000000000000000000000000000000000000

000000000000000000000000000000000000000000000000000000000000000000000000000000000000000000000000000000000000000000000000000000000000000000000000000000000000000000000000000000000000000000000000000000000000000000000000000000000000000000000000000000000000000000000000000000000000000000000000000000000000000000000000000000000000000000000000000000000000000000000000000000000000000000000000000000000000000000000000000000000000000000000000000000000000000000000000000000000000000000000000000000000000000000000000000000000000000000000000000000000000000000000000000000000000000000000000000000000

000000000000000000000000000000000000000000000000000000000000000000000000000000000000000000000000000000000000000000000000000000000000000000000000000000000000000000000000000000000000000000000000000000000000000000000000000000000000000000000000000000000000000000000000000000000000000000000000000000000000000000000000000000000000000000000000000000000000000000000000000000000000000000000000000000000000000000000000000000000000000000000000000000000000000000000000000000000000000000000000000000000000000000000000000000000000000000000000000000000000000000000000000000000000000000000000000000000

>DP00062

MDTKHFLPLDFSTQVNSSLTSPTGRGSMAAPSLHPSLGPGIGSPGQLHSPISTLSSPINGMGPPFSVISSPMGPHSMSVPTTPTLGFSTGSPQLSSPMNPVSSSEDIKPPLGLNGVLKVPAHPSGNMASFTKHICAICGDRSSGKHYGVYSCEGCKGFFKRTVRKDLTYTCRDNKDCLIDKRQRNRCQYCRYQKCLAMGMKREAVQEERQRGKDRNENEVESTSSANEDMPVERILEAELAVEPKTETYVEANMGLNPSSPNDPVTNICQAADKQLFTLVEWAKRIPHFSELPLDDQVILLRAGWNELLIASFSHRSIAVKDGILLATGLHVHRNSAHSAGVGAIFDRVLTELVSKMRDMQMDKTELGCLRAIVLFNPDSKGLSNPAEVEALREKVYASLEAYCKHKYPEQPGRFAKLLLRLPALRSIGLKCLEHLFFFKLIGDTPIDTFLMEMLEAPHQMT

000000000000000000000000000000000000000000000000000000000000000000000000000000000000000000000000000000000000000000000000000000000000000000000000000000000000000000000000111111111111111111111000000000000000000000000000000000000000000000000000000000000000000000000000000000000000000000000000000000000000000000000000000000000000000000000000000000000000000000000000000000000000000000000000000000000000000000000000000000000000000000000000000000000000000000000000000000

000000000000000000000000000000000000000000000000000000000000000000000000000000000000000000000000000000000000000000000000000000000000000000000000000000000000000000000000000000000000000000000000000000000000000000000000000000000000000000000000000000000000000000000000000000000000000000000000000000000000000000000000000000000000000000000000000000000000000000000000000000000000000000000000000000000000000000000000000000000000000000000000000000000000000000000000000000

000000000000000000000000000000000000000000000000000000000000000000000000000000000000000000000000000000000000000000000000000000000000000000000000000000000000000000000000000000000000000000000000000000000000000000000000000000000000000000000000000000000000000000000000000000000000000000000000000000000000000000000000000000000000000000000000000000000000000000000000000000000000000000000000000000000000000000000000000000000000000000000000000000000000000000000000000000

000000000000000000000000000000000000000000000000000000000000000000000000000000000000000000000000000000000000000000000000000000000000000000000000000000000000000000000000000000000000000000000000000000000000000000000000000000000000000000000000000000000000000000000000000000000000000000000000000000000000000000000000000000000000000000000000000000000000000000000000000000000000000000000000000000000000000000000000000000000000000000000000000000000000000000000000000000

000000000000000000000000000000000000000000000000000000000000000000000000000000000000000000000000000000000000000000000000000000000000000000000000000000000000000000000000000000000000000000000000000000000000000000000000000000000000000000000000000000000000000000000000000000000000000000000000000000000000000000000000000000000000000000000000000000000000000000000000000000000000000000000000000000000000000000000000000000000000000000000000000000000000000000000000000000

>DP00133

MAEEQARHVKNGLECIRALKAEPIGSLAIEEAMAAWSEISDNPGQERATCREEKAGSSGLSKPCLSAIGSTEGGAPRIRGQGPGESDDDAETLGIPPRNLQASSTGLQCYYVYDHSGEAVKGIQDADSIMVQSGLDGDSTLSGGDNESENSDVDIGEPDTEGYAITDRGSAPISMGFRASDVETAEGGEIHELLRLQSRGNNFPKLGKTLNVPPPPDPGRASTSGTPIKKGTERRLASFGTEIASLLTGGATQCARKSPSEPSGPGAPAGNVPECVSNAALIQEWTPESGTTISPRSQNNEEGGDYYDDELFSDVQDIKTALAKIHEDNQKIISKLESLLLLKGEVESIKKQINRQNISISTLEGHLSSIMIAIPGLGKDPNDPTADVEINPDLKPIIGRDSGRALAEVLKKPVASRQLQGMTNGRTSSRGQLLKEFQLKPIGKKMSSAVGFVPDTGPASRSVIRSIIKSSRLEEDRKRYLMTLLDDIKGANDLAKFHQMLMKIIMK

111111111111111111111111111111111111111111111111111111111111111111111111111111111111111111111111111111111111111111111111111111111111111111111111111111111111111111111111111111111111111111111111111111111111111111111111111111111111111111111111111111111111111111111111111111111111111111111111111111111111000000000000000000000000000000000000000000000000000000000000000000000000000000000000000000000000000000000000000000000000000000000000000000000000000000000000000000000000000000000000000000000000000000000000000

000000000000000000000000000000000000000000000000000000000000000000000000000000000000000000000000000000000000000000000000000000000000000000000000000000000000000000000000000000000000000000000000000000000000000000000000000000000000000000000000000000000000000000000000000000000000000000000000000000000000000000000000000000000000000000000000000000000000000000000000000000000000000000000000000000000000000000000000000000000000000000000000000000000000000000000000000000000000000000000000000000000000000000000000000

000000000000000000000000000000000000000000000000000000000000000000000000000000000000000000000000000000000000000000000000000000000000000000000000000000000000000000000000000000000000000000000000000000000000000000000000000000000000000000000000000000000000000000000000000000000000000000000000000000000000000000000000000000000000000000000000000000000000000000000000000000000000000000000000000000000000000000000000000000000000000000000000000000000000000000000000000000000000000000000000000000000000000000000000000

000000000000000000000000000000000000000000000000000000000000000000000000000000000000000000000000000000000000000000000000000000000000000000000000000000000000000000000000000000000000000000000000000000000000000000000000000000000000000000000000000000000000000000000000000000000000000000000000000000000000000000000000000000000000000000000000000000000000000000000000000000000000000000000000000000000000000000000000000000000000000000000000000000000000000000000000000000000000000000000000000000000000000000000000000

000000000000000000000000000000000000000000000000000000000000000000000000000000000000000000000000000000000000000000000000000000000000000000000000000000000000000000000000000000000000000000000000000000000000000000000000000000000000000000000000000000000000000000000000000000000000000000000000000000000000000000000000000000000000000000000000000000000000000000000000000000000000000000000000000000000000000000000000000000000000000000000000000000000000000000000000000000000000000000000000000000000000000000000000000

>DP02848

MSEKRSLPMVDVKIDDEDTPQLEKKIKRQSIDHGVGSEPVSTIEIIPSDSFRKYNSQGFKAKDTDLMGTQLESTFEQELSQMEHDMADQEEHDLSSFERKKLPTDFDPSLYDISFQQIDAEQSVLNGIKDENTSTVVRFFGVTSEGHSVLCNVTGFKNYLYVPAPNSSDANDQEQINKFVHYLNETFDHAIDSIEVVSKQSIWGYSGDTKLPFWKIYVTYPHMVNKLRTAFERGHLSFNSWFSNGTTTYDNIAYTLRLMVDCGIVGMSWITLPKGKYSMIEPNNRVSSCQLEVSINYRNLIAHPAEGDWSHTAPLRIMSFDIECAGRIGVFPEPEYDPVIQIANVVSIAGAKKPFIRNVFTLNTCSPITGSMIFSHATEEEMLSNWRNFIIKVDPDVIIGYNTTNFDIPYLLNRAKALKVNDFPYFGRLKTVKQEIKESVFSSKAYGTRETKNVNIDGRLQLDLLQFIQREYKLRSYTLNAVSAHFLGEQKEDVHYSIISDLQNGDSETRRRLAVYCLKDAYLPLRLMEKLMALVNYTEMARVTGVPFSYLLARGQQIKVVSQLFRKCLEIDTVIPNMQSQASDDQYEGATVIEPIRGYYDVPIATLDFNSLYPSIMMAHNLCYTTLCNKATVERLNLKIDEDYVITPNGDYFVTTKRRRGILPIILDELISARKRAKKDLRDEKDPFKRDVLNGRQLALKISANSVYGFTGATVGKLPCLAISSSVTAYGRTMILKTKTAVQEKYCIKNGYKHDAVVVYGDTDSVMVKFGTTDLKEAMDLGTEAAKYVSTLFKHPINLEFEKAYFPYLLINKKRYAGLFWTNPDKFDKLDQKGLASVRRDSCSLVSIVMNKVLKKILIERNVDGALAFVRETINDILHNRVDISKLIISKTLAPNYTNPQPHAVLAERMKRREGVGPNVGDRVDYVIIGGNDKLYNRAEDPLFVLENNIQVDSRYYLTNQLQNPIISIVAPIIGDKQANGMFVVKSIKINTGSQKGGLMSFIKKVEACKSCKGPLRKGEGPLCSNCLARSGELYIKALYDVRDLEEKYSRLWTQCQRCAGNLHSEVLCSNKNCDIFYMRVKVKKELQEKVEQLSKW

00000000000000000000000000000000000000000000000000000000000000000000000000000000000000000000000000000000000000000000000000000000000000000000000000000000000000000000000000000000000000000000000000000000000000000000000000000000000000000000000000000000000000000000000000000000000000000000000000000000000000000000000000000000000000000000000000000000000000000000000000000000000000000000000000000000000000000000000000000000000000000000000000000000000000000000000000000000000000000000000000000000000000000000000000000000000000000000000000000000000000000000000000000000000000000000000000000000000000000000000000000000000000000000000000000000000000000000000000000000000000000000000000000000000000000000000000000000000000000000000000000000000000000000000000000000000000000000000000000000000000000000000000000000000000000000000000000000000000000000000000000000000000000000000000000000000000000000000000000000000000000000000000000000000000000000000000000000000000000000000000000000000000000000000000000000000000000000001111111111111111111111111111111111111111111110000000000000000000000000000000000000000000000

00000000000000000000000000000000000000000000000000000000000000000000000000000000000000000000000000000000000000000000000000000000000000000000000000000000000000000000000000000000000000000000000000000000000000000000000000000000000000000000000000000000000000000000000000000000000000000000000000000000000000000000000000000000000000000000000000000000000000000000000000000000000000000000000000000000000000000000000000000000000000000000000000000000000000000000000000000000000000000000000000000000000000000000000000000000000000000000000000000000000000000000000000000000000000000000000000000000000000000000000000000000000000000000000000000000000000000000000000000000000000000000000000000000000000000000000000000000000000000000000000000000000000000000000000000000000000000000000000000000000000000000000000000000000000000000000000000000000000000000000000000000000000000000000000000000000000000000000000000000000000000000000000000000000000000000000000000000000000000000000000000000000000000000000000000000000000000000000000000000000000000000000000000000000000000000000000000000000000000000000000000000000000000

00000000000000000000000000000000000000000000000000000000000000000000000000000000000000000000000000000000000000000000000000000000000000000000000000000000000000000000000000000000000000000000000000000000000000000000000000000000000000000000000000000000000000000000000000000000000000000000000000000000000000000000000000000000000000000000000000000000000000000000000000000000000000000000000000000000000000000000000000000000000000000000000000000000000000000000000000000000000000000000000000000000000000000000000000000000000000000000000000000000000000000000000000000000000000000000000000000000000000000000000000000000000000000000000000000000000000000000000000000000000000000000000000000000000000000000000000000000000000000000000000000000000000000000000000000000000000000000000000000000000000000000000000000000000000000000000000000000000000000000000000000000000000000000000000000000000000000000000000000000000000000000000000000000000000000000000000000000000000000000000000000000000000000000000000000000000000000000000000000000000000000000000000000000000000000000000000000000000000000000000000000000000000000

00000000000000000000000000000000000000000000000000000000000000000000000000000000000000000000000000000000000000000000000000000000000000000000000000000000000000000000000000000000000000000000000000000000000000000000000000000000000000000000000000000000000000000000000000000000000000000000000000000000000000000000000000000000000000000000000000000000000000000000000000000000000000000000000000000000000000000000000000000000000000000000000000000000000000000000000000000000000000000000000000000000000000000000000000000000000000000000000000000000000000000000000000000000000000000000000000000000000000000000000000000000000000000000000000000000000000000000000000000000000000000000000000000000000000000000000000000000000000000000000000000000000000000000000000000000000000000000000000000000000000000000000000000000000000000000000000000000000000000000000000000000000000000000000000000000000000000000000000000000000000000000000000000000000000000000000000000000000000000000000000000000000000000000000000000000000000000000000000000000000000000000000000000000000000000000000000000000000000000000000000000000000000000

00000000000000000000000000000000000000000000000000000000000000000000000000000000000000000000000000000000000000000000000000000000000000000000000000000000000000000000000000000000000000000000000000000000000000000000000000000000000000000000000000000000000000000000000000000000000000000000000000000000000000000000000000000000000000000000000000000000000000000000000000000000000000000000000000000000000000000000000000000000000000000000000000000000000000000000000000000000000000000000000000000000000000000000000000000000000000000000000000000000000000000000000000000000000000000000000000000000000000000000000000000000000000000000000000000000000000000000000000000000000000000000000000000000000000000000000000000000000000000000000000000000000000000000000000000000000000000000000000000000000000000000000000000000000000000000000000000000000000000000000000000000000000000000000000000000000000000000000000000000000000000000000000000000000000000000000000000000000000000000000000000000000000000000000000000000000000000000000000000000000000000000000000000000000000000000000000000000000000000000000000000000000000000

>DP00797

MATSELSCEVSEENCERREAFWAEWKDLTLSTRPEEGCSLHEEDTQRHETYHQQGQCQVLVQRSPWLMMRMGILGRGLQEYQLPYQRVLPLPIFTPAKMGATKEEREDTPIQLQELLALETALGGQCVDRQEVAEITKQLPPVVPVSKPGALRRSLSRSMSQEAQRG

00000000000000000000000000000000000000000000000000000000000000000000000000000000000000000011111111111111111111111111111111111111111111111111111111111111111111111111111

00000000000000000000000000000000000000000000000000000000000000000000000000000000000000000000000000000000000000000000000000000000000000000000000000000000000000000000000

00000000000000000000000000000000000000000000000000000000000000000000000000000000000000000000000000000000000000000000000000000000000000000000000000000000000000000000000

00000000000000000000000000000000000000000000000000000000000000000000000000000000000000000000000000000000000000000000000000000000000000000000000000000000000000000000000

00000000000000000000000000000000000000000000000000000000000000000000000000000000000000000000000000000000000000000000000000000000000000000000000000000000000000000000000

>DP01426

MPPKTPRKTAATAAAAAAEPPAPPPPPPPEEDPEQDSGPEDLPLVRLEFEETEEPDFTALCQKLKIPDHVRERAWLTWEKVSSVDGVLGGYIQKKKELWGICIFIAAVDLDEMSFTFTELQKNIEISVHKFFNLLKEIDTSTKVDNAMSRLLKKYDVLFALFSKLERTCELIYLTQPSSSISTEINSALVLKVSWITFLLAKGEVLQMEDDLVISFQLMLCVLDYFIKLSPPMLLKEPYKTAVIPINGSPRTPRRGQNRSARIAKQLENDTRIIEVLCKEHECNIDEVKNVYFKNFIPFMNSLGLVTSNGLPEVENLSKRYEEIYLKNKDLDARLFLDHDKTLQTDSIDSFETQRTPRKSNLDEEVNVIPPHTPVRTVMNTIQQLMMILNSASDQPSENLISYFNNCTVNPKESILKRVKDIGYIFKEKFAKAVGQGCVEIGSQRYKLGVRLYYRVMESMLKSEEERLSIQNFSKLLNDNIFHMSLLACALEVVMATYSRSTSQNLDSGTDLSFPWILNVLNLKAFDFYKVIESFIKAEGNLTREMIKHLERCEHRIMESLAWLSDSPLFDLIKQSKDREGPTDHLESACPLNLPLQNNHTAADMYLSPVRSPKKKGSTTRVNSTANAETQATSAFQTQKPLKSTSLSLFYKKVYRLAYLRLNTLCERLLSEHPELEHIIWTLFQHTLQNEYELMRDRHLDQIMMCSMYGICKVKNIDLKFKIIVTAYKDLPHAVQETFKRVLIKEEEYDSIIVFYNSVFMQRLKTNILQYASTRPPTLSPIPHIPRSPYKFPSSPLRIPGGNIYISPLKSPYKISEGLPTPTKMTPRSRILVSIGESFGTSEKFQKINQMVCNSDRVLKRSAEGSNPPKPLKKLRFDIEGSDEADGSKHLPGESKFQQKLAEMTSTRTRMQKQKMNDSMDTSNKEEK

0000000000000000000000000000000000000000000000000000000000000000000000000000000000000000000000000000000000000000000000000000000000000000000000000000000000000000000000000000000000000000000000000000000000000000000000000000000000000000000000000000000000000000000000000000000000000000000000000000000000000000000000000000000000000000000000000000000000000000000000000000000000000000000000000000000000000000000000000000000000000000000000000000000000000000000000000000000000000000000000000000000000000000000000000000000000000000000000000000000000000000000000000000000000000000000000000000000000000000000000000000000000000000000000000000000000000000000000000000000000000000000000000000000000000000000000000000000000000000000000000000000000000000000000000000000000000000000000000000000000000000000000000000000000000000000000000000000000001111111111111111111111111111111111111111111110000000000000000000000000000000000000000000000000000000

0000000000000000000000000000000000000000000000000000000000000000000000000000000000000000000000000000000000000000000000000000000000000000000000000000000000000000000000000000000000000000000000000000000000000000000000000000000000000000000000000000000000000000000000000000000000000000000000000000000000000000000000000000000000000000000000000000000000000000000000000000000000000000000000000000000000000000000000000000000000000000000000000000000000000000000000000000000000000000000000000000000000000000000000000000000000000000000000000000000000000000000000000000000000000000000000000000000000000000000000000000000000000000000000000000000000000000000000000000000000000000000000000000000000000000000000000000000000000000000000000000000000000000000000000000000000000000000000000000000000000000000000000000000000000000000000000000000000000000000000000000000000000000000000000000000000000000000000000000000000000000000000000000000000000000

0000000000000000000000000000000000000000000000000000000000000000000000000000000000000000000000000000000000000000000000000000000000000000000000000000000000000000000000000000000000000000000000000000000000000000000000000000000000000000000000000000000000000000000000000000000000000000000000000000000000000000000000000000000000000000000000000000000000000000000000000000000000000000000000000000000000000000000000000000000000000000000000000000000000000000000000000000000000000000000000000000000000000000000000000000000000000000000000000000000000000000000000000000000000000000000000000000000000000000000000000000000000000000000000000000000000000000000000000000000000000000000000000000000000000000000000000000000000000000000000000000000000000000000000000000000000000000000000000000000000000000000000000000000000000000000000000000000000000000000000000000000000000000000000000000000000000000000000000000000000000000000000000000000000000000

0000000000000000000000000000000000000000000000000000000000000000000000000000000000000000000000000000000000000000000000000000000000000000000000000000000000000000000000000000000000000000000000000000000000000000000000000000000000000000000000000000000000000000000000000000000000000000000000000000000000000000000000000000000000000000000000000000000000000000000000000000000000000000000000000000000000000000000000000000000000000000000000000000000000000000000000000000000000000000000000000000000000000000000000000000000000000000000000000000000000000000000000000000000000000000000000000000000000000000000000000000000000000000000000000000000000000000000000000000000000000000000000000000000000000000000000000000000000000000000000000000000000000000000000000000000000000000000000000000000000000000000000000000000000000000000000000000000000000000000000000000000000000000000000000000000000000000000000000000000000000000000000000000000000000000

0000000000000000000000000000000000000000000000000000000000000000000000000000000000000000000000000000000000000000000000000000000000000000000000000000000000000000000000000000000000000000000000000000000000000000000000000000000000000000000000000000000000000000000000000000000000000000000000000000000000000000000000000000000000000000000000000000000000000000000000000000000000000000000000000000000000000000000000000000000000000000000000000000000000000000000000000000000000000000000000000000000000000000000000000000000000000000000000000000000000000000000000000000000000000000000000000000000000000000000000000000000000000000000000000000000000000000000000000000000000000000000000000000000000000000000000000000000000000000000000000000000000000000000000000000000000000000000000000000000000000000000000000000000000000000000000000000000000000000000000000000000000000000000000000000000000000000000000000000000000000000000000000000000000000000

>DP00748

MAGNFDSEERSSWYWGRLSRQEAVALLQGQRHGVFLVRDSSTSPGDYVLSVSENSRVSHYIINSSGPRPPVPPSPAQPPPGVSPSRLRIGDQEFDSLPALLEFYKIHYLDTTTLIEPVSRSRQGSGVILRQEEAEYVRALFDFNGNDEEDLPFKKGDILRIRDKPEEQWWNAEDSEGKRGMIPVPYVEKYRPASASVSALIGGNQEGSHPQPLGGPEPGPYAQPSVNTPLPNLQNGPIYARVIQKRVPNAYDKTALALEVGELVKVTKINVSGQWEGECNGKRGHFPFTHVRLLDQQNPDEDFS

0000000000000000000000000000000000000000000000000000000000000000000000000000000000000000000000000000000000000000000000000000000000000000000000000000000000000000000000000000000000000000000000000000000000000000000000000000000111111111111111111111111111111111111111111111111111111111111111111111100000000000

0000000000000000000000000000000000000000000000000000000000000000000000000000000000000000000000000000000000000000000000000000000000000000000000000000000000000000000000000000000000000000000000000000000000000000000000000000000000000000000000000000000000000000000000000000000000000000000000000000000000000000

0000000000000000000000000000000000000000000000000000000000000000000000000000000000000000000000000000000000000000000000000000000000000000000000000000000000000000000000000000000000000000000000000000000000000000000000000000000000000000000000000000000000000000000000000000000000000000000000000000000000000000

0000000000000000000000000000000000000000000000000000000000000000000000000000000000000000000000000000000000000000000000000000000000000000000000000000000000000000000000000000000000000000000000000000000000000000000000000000000000000000000000000000000000000000000000000000000000000000000000000000000000000000

0000000000000000000000000000000000000000000000000000000000000000000000000000000000000000000000000000000000000000000000000000000000000000000000000000000000000000000000000000000000000000000000000000000000000000000000000000000000000000000000000000000000000000000000000000000000000000000000000000000000000000

>DP00047

AAQKRPSQRSKYLASASTMDHARHGFLPRHRDTGILDSLGRFFGSDRGAPKRGSGKDGHHAARTTHYGSLPQKAQGHRPQDENPVVHFFKNIVTPRTPPPSQGKGRGLSLSRFSWGAEGQKPGFGYGGRASDYKSAHKGLKGHDAQGTLSKIFKLGGRDSRSGSPMARR

1111111111111111111111111111111111111111111111111111111111111111111111111111111111111111111111111111111111111000000000000000000000000000000000000000000000000000000000000

0000000000000000000000000000000000000000000000000000000000000000000000000000000000000000000000000000000000000000000000000000000000000000000000000000000000000000000000000

0000000000000000000000000000000000000000000000000000000000000000000000000000000000000000000000000000000000000000000000000000000000000000000000000000000000000000000000000

0000000000000000000000000000000000000000000000000000000000000000000000000000000000000000000000000000000000000000000000000000000000000000000000000000000000000000000000000

0000000000000000000000000000000000000000000000000000000000000000000000000000000000000000000000000000000000000000000000000000000000000000000000000000000000000000000000000

>DP02583

MSAETVNNYDYSDWYENAAPTKAPVEVIPPCDPTADEGLFHICIAAISLVVMLVLAILARRQKLSDNQRGLTGLLSPVNFLDHTQHKGLAVAVYGVLFCKLVGMVLSHHPLPFTKEVANKEFWMILALLYYPALYYPLLACGTLHNKVGYVLGSLLSWTHFGILVWQKVDCPKTPQIYKYYALFGSLPQIACLAFLSFQYPLLLFKGLQNTETANASEDLSSSYYRDYVKKILKKKKPTKISSSTSKPKLFDRLRDAVKSYIYTPEDVFRFPLKLAISVVVAFIALYQMALLLISGVLPTLHIVRRGVDENIAFLLAGFNIILSNDRQEVVRIVVYYLWCVEICYVSAVTLSCLVNLLMLMRSMVLHRSNLKGLYRGDSLNVFNCHRSIRPSRPALVCWMGFTSYQAAFLCLGMAIQTLVFFICILFAVFLIIIPILWGTNLMLFHIIGNLWPFWLTLVLAALIQHVASRFLFIRKDGGTRDLNNRGSLFLLSYILFLVNVMIGVVLGIWRVVITALFNIVHLGRLDISLLNRNVEAFDPGYRCYSHYLKIEVSQSHPVMKAFCGLLLQSSGQDGLSAQRIRDAEEGIQLVQQEKKQNKVSNAKRARAHWQLLYTLVNNPSLVGSRKHFQCQSSESFINGALSRTSKEGSKKDGSVNEPSKEAESAAASN

0000000000000000000000000000000000000000000000000000000000000000000000000000000000000000000000000000000000000000000000000000000000000000000000000000000000000000000000000000000000000000000000000000000000000000000000000000000000000000000000000000000000000000000000000000000000000000000000000000000000000000000000000000000000000000000000000000000000000000000000000000000000000000000000000000000000000000000000000000000000000000000000000000000000000000000000000000000000000000000000000000000000000000000000000000000000000000000000000000000000000000000000000000000000000000000000000000000011111110000000000000000000000000000000000000000000000000000000000000000000000000000000

0000000000000000000000000000000000000000000000000000000000000000000000000000000000000000000000000000000000000000000000000000000000000000000000000000000000000000000000000000000000000000000000000000000000000000000000000000000000000000000000000000000000000000000000000000000000000000000000000000000000000000000000000000000000000000000000000000000000000000000000000000000000000000000000000000000000000000000000000000000000000000000000000000000000000000000000000000000000000000000000000000000000000000000000000000000000000000000000000000000000000000000000000000000000000000000000000000000000000000000000000000000000000000000000000000000000000000000000000000000000000000000000

0000000000000000000000000000000000000000000000000000000000000000000000000000000000000000000000000000000000000000000000000000000000000000000000000000000000000000000000000000000000000000000000000000000000000000000000000000000000000000000000000000000000000000000000000000000000000000000000000000000000000000000000000000000000000000000000000000000000000000000000000000000000000000000000000000000000000000000000000000000000000000000000000000000000000000000000000000000000000000000000000000000000000000000000000000000000000000000000000000000000000000000000000000000000000000000000000000000000000000000000000000000000000000000000000000000000000000000000000000000000000000000000

0000000000000000000000000000000000000000000000000000000000000000000000000000000000000000000000000000000000000000000000000000000000000000000000000000000000000000000000000000000000000000000000000000000000000000000000000000000000000000000000000000000000000000000000000000000000000000000000000000000000000000000000000000000000000000000000000000000000000000000000000000000000000000000000000000000000000000000000000000000000000000000000000000000000000000000000000000000000000000000000000000000000000000000000000000000000000000000000000000000000000000000000000000000000000000000000000000000000000000000000000000000000000000000000000000000000000000000000000000000000000000000000

0000000000000000000000000000000000000000000000000000000000000000000000000000000000000000000000000000000000000000000000000000000000000000000000000000000000000000000000000000000000000000000000000000000000000000000000000000000000000000000000000000000000000000000000000000000000000000000000000000000000000000000000000000000000000000000000000000000000000000000000000000000000000000000000000000000000000000000000000000000000000000000000000000000000000000000000000000000000000000000000000000000000000000000000000000000000000000000000000000000000000000000000000000000000000000000000000000000000000000000000000000000000000000000000000000000000000000000000000000000000000000000000

>DP00297

MAHAGRTGYDNREIVMKYIHYKLSQRGYEWDAGDVGAAPPGAAPAPGIFSSQPGHTPHPAASRDPVARTSPLQTPAAPGAAAGPALSPVPPVVHLTLRQAGDDFSRRYRRDFAEMSSQLHLTPFTARGRFATVVEELFRDGVNWGRIVAFFEFGGVMCVESVNREMSPLVDNIALWMTEYLNRHLHTWIQDNGGWDAFVELYGPSMRPLFDFSWLSLKTLLSLALVGACITLGAYLGHK

00000000000000000000000001111111111111111111111100000000000000000000000000000000000000000000000000000000000000000000000000000000000000000000000000000000000000000000000000000000000000000000000000000000000000000000000000000000000000000000000

00000000000000000000000000000000000000000000000000000000000000000000000000000000000000000000000000000000000000000000000000000000000000000000000000000000000000000000000000000000000000000000000000000000000000000000000000000000000000000000000

00000000000000000000000000000000000000000000000000000000000000000000000000000000000000000000000000000000000000000000000000000000000000000000000000000000000000000000000000000000000000000000000000000000000000000000000000000000000000000000000

00000000000000000000000000000000000000000000000000000000000000000000000000000000000000000000000000000000000000000000000000000000000000000000000000000000000000000000000000000000000000000000000000000000000000000000000000000000000000000000000

00000000000000000000000000000000000000000000000000000000000000000000000000000000000000000000000000000000000000000000000000000000000000000000000000000000000000000000000000000000000000000000000000000000000000000000000000000000000000000000000

>DP00969

MDLEKNYPTPRTSRTGHGGVNQLGGVFVNGRPLPDVVRQRIVELAHQGVRPCDISRQLRVSHGCVSKILGRYYETGSIKPGVIGGSKPKVATPKVVEKIAEYKRQNPTMFAWEIRDRLLAERVCDNDTVPSVSSINRIIRTKVQQPPNQPVPASSHSIVSTGSVTQVSSVSTDSAGSSYSISGILGITSPSADTNKRKRDEGIQESPVPNGHSLPGRDFLRKQMRGDLFTQQQLEVLDRVFERQHYSDIFTTTEPIKPEQTTEYSAMASLAGGLDDMKANLASPTPADIGSSVPGPQSYPIVTGRDLASTTLPGYPPHVPPAGQGSYSAPTLTGMVPGSEFSGSPYSHPQYSSYNDSWRFPNPGLLGSPYYYSAAARGAAPPAAATAYDRH

1111111111111111110000000000000000000000000000000000000000000000000000000000000000000000000000000000000000000000000000000000000000000000000000000000000000000000000000000000000000000000000000000000000000000000000000000000000000000000000000000000000000000000000000000000000000000000000000000000000000000000000000000000000000000000000000000000000000000000000000000000000000000000000000000000000

0000000000000000000000000000000000000000000000000000000000000000000000000000000000000000000000000000000000000000000000000000000000000000000000000000000000000000000000000000000000000000000000000000000000000000000000000000000000000000000000000000000000000000000000000000000000000000000000000000000000000000000000000000000000000000000000000000000000000000000000000000000000000000000000000000000

0000000000000000000000000000000000000000000000000000000000000000000000000000000000000000000000000000000000000000000000000000000000000000000000000000000000000000000000000000000000000000000000000000000000000000000000000000000000000000000000000000000000000000000000000000000000000000000000000000000000000000000000000000000000000000000000000000000000000000000000000000000000000000000000000000000

0000000000000000000000000000000000000000000000000000000000000000000000000000000000000000000000000000000000000000000000000000000000000000000000000000000000000000000000000000000000000000000000000000000000000000000000000000000000000000000000000000000000000000000000000000000000000000000000000000000000000000000000000000000000000000000000000000000000000000000000000000000000000000000000000000000

0000000000000000000000000000000000000000000000000000000000000000000000000000000000000000000000000000000000000000000000000000000000000000000000000000000000000000000000000000000000000000000000000000000000000000000000000000000000000000000000000000000000000000000000000000000000000000000000000000000000000000000000000000000000000000000000000000000000000000000000000000000000000000000000000000000

>DP02736

MSKFTWKELIQLGSPSKAYESSLACIAHIDMNAFFAQVEQMRCGLSKEDPVVCVQWNSIIAVSYAARKYGISRMDTIQEALKKCSNLIPIHTAVFKKGEDFWQYHDGCGSWVQDPAKQISVEDHKVSLEPYRRESRKALKIFKSACDLVERASIDEVFLDLGRICFNMLMFDNEYELTGDLKLKDALSNIREAFIGGNYDINSHLPLIPEKIKSLKFEGDVFNPEGRDLITDWDDVILALGSQVCKGIRDSIKDILGYTTSCGLSSTKNVCKLASNYKKPDAQTIVKNDCLLDFLDCGKFEITSFWTLGGVLGKELIDVLDLPHENSIKHIRETWPDNAGQLKEFLDAKVKQSDYDRSTSNIDPLKTADLAEKLFKLSRGRYGLPLSSRPVVKSMMSNKNLRGKSCNSIVDCISWLEVFCAELTSRIQDLEQEYNKIVIPRTVSISLKTKSYEVYRKSGPVAYKGINFQSHELLKVGIKFVTDLDIKGKNKSYYPLTKLSMTITNFDIIDLQKTVVDMFGNQVHTFKSSAGKEDEEKTTSSKADEKTPKLECCKYQVTFTDQKALQEHADYHLALKLSEGLNGAEESSKNLSFGEKRLLFSRKRPNSQHTATPQKKQVTSSKNILSFFTRKK

00000000000000000000000000000000000000000000000000000000000000000000000000000000000000000000000000000000000000000000000000000000000000000000000000000000000000000000000000000000000000000000000000000000000000000000000000000000000000000000000000000000000000000000000000000000000000000000000000000000000000000000000000000000000000000000000000000000000000000000000000000000000000000000000000000000000000000000000000000000000000000000000000000000000000000000000000000000000000000000000000000000000000000000000000000111111111111111111111111111111111111111111111111111111111111111111111111111111111111111111111111111111111111111111111111111

00000000000000000000000000000000000000000000000000000000000000000000000000000000000000000000000000000000000000000000000000000000000000000000000000000000000000000000000000000000000000000000000000000000000000000000000000000000000000000000000000000000000000000000000000000000000000000000000000000000000000000000000000000000000000000000000000000000000000000000000000000000000000000000000000000000000000000000000000000000000000000000000000000000000000000000000000000000000000000000000000000000000000000000000000000000000000000000000000000000000000000000000000000000000000000000000000000000000000000000000000000000000000000000000000000000

00000000000000000000000000000000000000000000000000000000000000000000000000000000000000000000000000000000000000000000000000000000000000000000000000000000000000000000000000000000000000000000000000000000000000000000000000000000000000000000000000000000000000000000000000000000000000000000000000000000000000000000000000000000000000000000000000000000000000000000000000000000000000000000000000000000000000000000000000000000000000000000000000000000000000000000000000000000000000000000000000000000000000000000000000000000000000000000000000000000000000000000000000000000000000000000000000000000000000000000000000000000000000000000000000000000

00000000000000000000000000000000000000000000000000000000000000000000000000000000000000000000000000000000000000000000000000000000000000000000000000000000000000000000000000000000000000000000000000000000000000000000000000000000000000000000000000000000000000000000000000000000000000000000000000000000000000000000000000000000000000000000000000000000000000000000000000000000000000000000000000000000000000000000000000000000000000000000000000000000000000000000000000000000000000000000000000000000000000000000000000000000000000000000000000000000000000000000000000000000000000000000000000000000000000000000000000000000000000000000000000000000

00000000000000000000000000000000000000000000000000000000000000000000000000000000000000000000000000000000000000000000000000000000000000000000000000000000000000000000000000000000000000000000000000000000000000000000000000000000000000000000000000000000000000000000000000000000000000000000000000000000000000000000000000000000000000000000000000000000000000000000000000000000000000000000000000000000000000000000000000000000000000000000000000000000000000000000000000000000000000000000000000000000000000000000000000000000000000000000000000000000000000000000000000000000000000000000000000000000000000000000000000000000000000000000000000000000

>DP00877

MAQEQTKRGGGGGDDDDIAGSTAAGQERREKLTEETDDLLDEIDDVLEENAEDFVRAYVQKGGQ

0000000000000000000011111111111111111111111111111110000000000000

0000000000000000000000000000000000000000000000000000000000000000

0000000000000000000000000000000000000000000000000000000000000000

0000000000000000000000000000000000000000000000000000000000000000

0000000000000000000000000000000000000000000000000000000000000000

>DP01106

MKENVASATVFTLLLFLNTCLLNGQLPPGKPEIFKCRSPNKETFTCWWRPGTDGGLPTNYSLTYHREGETLMHECPDYITGGPNSCHFGKQYTSMWRTYIMMVNATNQMGSSFSDELYVDVTYIVQPDPPLELAVEVKQPEDRKPYLWIKWSPPTLIDLKTGWFTLLYEIRLKPEKAAEWEIHFAGQQTEFKILSLHPGQKYLVQVRCKPDHGYWSAWSPATFIQIPSDFTMNDTTVWISVAVLSAVICLIIVWAVALKGYSMVTCIFPPVPGPKIKGFDAHLLEKGKSEELLSALGCQDFPPTSDYEDLLVEYLEVDDSEDQHLMSVHSKEHPSQGMKPTYLDPDTDSGRGSCDSPSLLSEKCEEPQANPSTFYDPEVIEKPENPETTHTWDPQCISMEGKIPYFHAGGSKCSTWPLPQPSQHNPRSSYHNITDVCELAVGPAGAPATLLNEAGKDALKSSQTIKSREEGKATQQREVESFHSETDQDTPWLLPQEKTPFGSAKPLDYVEIHKVNKDGALSLLPKQRENSGKPKKPGTPENNKEYAKVSGVMDNNILVLVPDPHAKNVACFEESAKEAPPSLEQNQAEKALANFTATSSKCRLQLGGLDYLDPACFTHSFH

0000000000000000000000000000000000000000000000000000000000000000000000000000000000000000000000000000000000000000000000000000000000000000000000000000000000000000000000000000000000000000000000000000000000000000000000000000000000000000000111111111111111111111111111111111111111111111111111111111111111111111111111111111111111111111111111111111111111111111111111111111111111111111111111111111111111111111111111111111111111111111111111111111111111111111111111111111111111111111111111111111111111111111111111111111111111111111111111111111111111111111111111111111111111111111111111111111111111111111111111000000000000000000000000

0000000000000000000000000000000000000000000000000000000000000000000000000000000000000000000000000000000000000000000000000000000000000000000000000000000000000000000000000000000000000000000000000000000000000000000000000000000000000000000000000000000000000000000000000000000000000000000000000000000000000000000000000000000000000000000000000000000000000000000000000000000000000000000000000000000000000000000000000000000000000000000000000000000000000000000000000000000000000000000000000000000000000000000000000000000000000000000000000000000000000000000000000000000000000000000000000000000000000000000000000000000000000000000000

0000000000000000000000000000000000000000000000000000000000000000000000000000000000000000000000000000000000000000000000000000000000000000000000000000000000000000000000000000000000000000000000000000000000000000000000000000000000000000000000000000000000000000000000000000000000000000000000000000000000000000000000000000000000000000000000000000000000000000000000000000000000000000000000000000000000000000000000000000000000000000000000000000000000000000000000000000000000000000000000000000000000000000000000000000000000000000000000000000000000000000000000000000000000000000000000000000000000000000000000000000000000000000000000

0000000000000000000000000000000000000000000000000000000000000000000000000000000000000000000000000000000000000000000000000000000000000000000000000000000000000000000000000000000000000000000000000000000000000000000000000000000000000000000000000000000000000000000000000000000000000000000000000000000000000000000000000000000000000000000000000000000000000000000000000000000000000000000000000000000000000000000000000000000000000000000000000000000000000000000000000000000000000000000000000000000000000000000000000000000000000000000000000000000000000000000000000000000000000000000000000000000000000000000000000000000000000000000000

0000000000000000000000000000000000000000000000000000000000000000000000000000000000000000000000000000000000000000000000000000000000000000000000000000000000000000000000000000000000000000000000000000000000000000000000000000000000000000000000000000000000000000000000000000000000000000000000000000000000000000000000000000000000000000000000000000000000000000000000000000000000000000000000000000000000000000000000000000000000000000000000000000000000000000000000000000000000000000000000000000000000000000000000000000000000000000000000000000000000000000000000000000000000000000000000000000000000000000000000000000000000000000000000

>DP00347

MNLEPPKAEIRSATRVMGGPVTPRKGPPKFKQRQTRQFKSKPPKKGVQGFGDDIPGMEGLGTDITVICPWEAFNHLELHELAQYGII

111111111111111111111111111111111111111111111111111111111111111111111111111111111111111

000000000000000000000000000000000000000000000000000000000000000000000000000000000000000

000000000000000000000000000000000000000000000000000000000000000000000000000000000000000

000000000000000000000000000000000000000000000000000000000000000000000000000000000000000

000000000000000000000000000000000000000000000000000000000000000000000000000000000000000

>DP01262

MAEEKKLKLSNTVLPSESMKVVAESMGIAQIQEETCQLLTDEVSYRIKEIAQDALKFMHMGKRQKLTTSDIDYALKLKNVEPLYGFHAQEFIPFRFASGGGRELYFYEEKEVDLSDIINTPLPRVPLDVCLKAHWLSIEGCQPAIPENPPPAPKEQQKAEATEPLKSAKPGQEEDGPLKGKGQGATTADGKGKEKKAPPLLEGAPLRLKPRSIHELSVEQQLYYKEITEACVGSCEAKRAEALQSIATDPGLYQMLPRFSTFISEGVRVNVVQNNLALLIYLMRMVKALMDNPTLYLEKYVHELIPAVMTCIVSRQLCLRPDVDNHWALRDFAARLVAQICKHFSTTTNNIQSRITKTFTKSWVDEKTPWTTRYGSIAGLAELGHDVIKTLILPRLQQEGERIRSVLDGPVLSNIDRIGADHVQSLLLKHCAPVLAKLRPPPDNQDAYRAEFGSLGPLLCSQVVKARAQAALQAQQVNRTTLTITQPRPTLTLSQAPQPGPRTPGLLKVPGSIALPVQTLVSARAAAPPQPSPPPTKFIVMSSSSSAPSTQQVLSLSTSAPGSGSTTTSPVTTTVPSVQPIVKLVSTATTAPPSTAPSGPGSVQKYIVVSLPPTGEGKGGPTSHPSPVPPPASSPSPLSGSALCGGKQEAGDSPPPAPGTPKANGSQPNSGSPQPAP

11111111111111111111111111111111111111111111111111111111111111111111111111111111111111111111111111111111111111111111111111111111111111111111111111111111111111111111111111111111111111111111111111111111111111111111111100000000000000000000000000000000000000000000000000000000000000000000000000000000000000000000000000000000000000000000000000000000000000000000000000000000000000000000000000000000000000000000000000000000000000000000000000000000000000000000000000000000000000000000000000000000000000000000000000000000000000000000000000000000000000000000000000000000000000000000000000000000000000000000000000000000000000000000000000000000000000000000000000000000000000000000000000000

00000000000000000000000000000000000000000000000000000000000000000000000000000000000000000000000000000000000000000000000000000000000000000000000000000000000000000000000000000000000000000000000000000000000000000000000000000000000000000000000000000000000000000000000000000000000000000000000000000000000000000000000000000000000000000000000000000000000000000000000000000000000000000000000000000000000000000000000000000000000000000000000000000000000000000000000000000000000000000000000000000000000000000000000000000000000000000000000000000000000000000000000000000000000000000000000000000000000000000000000000000000000000000000000000000000000000000000000000000000000000000000000000000

00000000000000000000000000000000000000000000000000000000000000000000000000000000000000000000000000000000000000000000000000000000000000000000000000000000000000000000000000000000000000000000000000000000000000000000000000000000000000000000000000000000000000000000000000000000000000000000000000000000000000000000000000000000000000000000000000000000000000000000000000000000000000000000000000000000000000000000000000000000000000000000000000000000000000000000000000000000000000000000000000000000000000000000000000000000000000000000000000000000000000000000000000000000000000000000000000000000000000000000000000000000000000000000000000000000000000000000000000000000000000000000000000000

00000000000000000000000000000000000000000000000000000000000000000000000000000000000000000000000000000000000000000000000000000000000000000000000000000000000000000000000000000000000000000000000000000000000000000000000000000000000000000000000000000000000000000000000000000000000000000000000000000000000000000000000000000000000000000000000000000000000000000000000000000000000000000000000000000000000000000000000000000000000000000000000000000000000000000000000000000000000000000000000000000000000000000000000000000000000000000000000000000000000000000000000000000000000000000000000000000000000000000000000000000000000000000000000000000000000000000000000000000000000000000000000000000

00000000000000000000000000000000000000000000000000000000000000000000000000000000000000000000000000000000000000000000000000000000000000000000000000000000000000000000000000000000000000000000000000000000000000000000000000000000000000000000000000000000000000000000000000000000000000000000000000000000000000000000000000000000000000000000000000000000000000000000000000000000000000000000000000000000000000000000000000000000000000000000000000000000000000000000000000000000000000000000000000000000000000000000000000000000000000000000000000000000000000000000000000000000000000000000000000000000000000000000000000000000000000000000000000000000000000000000000000000000000000000000000000000

>DP02301

MAGGKAGKDSGKAKAKAVSRSQRAGLQFPVGRIHRHLKTRTTSHGRVGATAAVYSAAILEYLTAEVLELAGNASKDLKVKRITPRHLQLAIRGDEELDSLIKATIAGGGVIPHIHKSLIGKKGQQKTA

00000000000000000000000000000000000000111111111100000000000000000000000000000000000000000000000000000000000000000000000000000000

00000000000000000000000000000000000000000000000000000000000000000000000000000000000000000000000000000000000000000000000000000000

00000000000000000000000000000000000000000000000000000000000000000000000000000000000000000000000000000000000000000000000000000000

00000000000000000000000000000000000000000000000000000000000000000000000000000000000000000000000000000000000000000000000000000000

00000000000000000000000000000000000000000000000000000000000000000000000000000000000000000000000000000000000000000000000000000000

>DP02831

MAEPLKEEDGEDGSAEPPGPVKAEPAHTAASVAAKNLALLKARSFDVTFDVGDEYEIIETIGNGAYGVVSSARRRLTGQQVAIKKIPNAFDVVTNAKRTLRELKILKHFKHDNIIAIKDILRPTVPYGEFKSVYVVLDLMESDLHQIIHSSQPLTLEHVRYFLYQLLRGLKYMHSAQVIHRDLKPSNLLVNENCELKIGDFGMARGLCTSPAEHQYFMTEYVATRWYRAPELMLSLHEYTQAIDLWSVGCIFGEMLARRQLFPGKNYVHQLQLIMMVLGTPSPAVIQAVGAERVRAYIQSLPPRQPVPWETVYPGADRQALSLLGRMLRFEPSARISAAAALRHPFLAKYHDPDDEPDCAPPFDFAFDREALTRERIKEAIVAEIEDFHARREGIRQQIRFQPSLQPVASEPGCPDVEMPSPWAPSGDCAMESPPPAPPPCPGPAPDTIDLTLQPPPPVSEPAPPKKDGAISDNTKAALKAALLKSLRSRLRDGPSAPLEAPEPRKPVTAQERQREREEKRRRRQERAKEREKRRQERERKERGAGASGGPSTDPLAGLVLSDNDRSLLERWTRMARPAAPALTSVPAPAPAPTPTPTPVQPTSPPPGPVAQPTGPQPQSAGSTSGPVPQPACPPPGPAPHPTGPPGPIPVPAPPQIATSTSLLAAQSLVPPPGLPGSSTPGVLPYFPPGLPPPDAGGAPQSSMSESPDVNLVTQQLSKSQVEDPLPPVFSGTPKGSGAGYGVGFDLEEFLNQSFDMGVADGPQDGQADSASLSASLLADWLEGHGMNPADIESLQREIQMDSPMLLADLPDLQDP

111111111111111111111111111111111111111111111100000000000000000000000000000000000000000000000000000000000000000000000000000000000000000000000000000000000000000000000000000000000000000000000000000000000000000000000000000000000000000000000000000000000000000000000000000000000000000000000000000000000000000000000000000000000000000000000000000000000000000000000000000000000000000000000000000000000000000000000000000000000000000000000000000000000000000000000000000000000000000000000000000000000000000000000000000000000000000000000000000000000000000000000000000000000000000000000000000000000000000000000000000000000000000000000000000000000000000000000000000000000000000000000000000000000000000000000000000000000000000000000000000000000000000000000000000000000000000000000000000000000000000000000000000000000000000000000000

000000000000000000000000000000000000000000000000000000000000000000000000000000000000000000000000000000000000000000000000000000000000000000000000000000000000000000000000000000000000000000000000000000000000000000000000000000000000000000000000000000000000000000000000000000000000000000000000000000000000000000000000000000000000000000000000000000000000000000000000000000000000000000000000000000000000000000000000000000000000000000000000000000000000000000000000000000000000000000000000000000000000000000000000000000000000000000000000000000000000000000000000000000000000000000000000000000000000000000000000000000000000000000000000000000000000000000000000000000000000000000000000000000000000000000000000000000000000000000000000000000000000000000000000000000000000000000000000000000000000000000000000000000000000000000000000

000000000000000000000000000000000000000000000000000000000000000000000000000000000000000000000000000000000000000000000000000000000000000000000000000000000000000000000000000000000000000000000000000000000000000000000000000000000000000000000000000000000000000000000000000000000000000000000000000000000000000000000000000000000000000000000000000000000000000000000000000000000000000000000000000000000000000000000000000000000000000000000000000000000000000000000000000000000000000000000000000000000000000000000000000000000000000000000000000000000000000000000000000000000000000000000000000000000000000000000000000000000000000000000000000000000000000000000000000000000000000000000000000000000000000000000000000000000000000000000000000000000000000000000000000000000000000000000000000000000000000000000000000000000000000000000000

000000000000000000000000000000000000000000000000000000000000000000000000000000000000000000000000000000000000000000000000000000000000000000000000000000000000000000000000000000000000000000000000000000000000000000000000000000000000000000000000000000000000000000000000000000000000000000000000000000000000000000000000000000000000000000000000000000000000000000000000000000000000000000000000000000000000000000000000000000000000000000000000000000000000000000000000000000000000000000000000000000000000000000000000000000000000000000000000000000000000000000000000000000000000000000000000000000000000000000000000000000000000000000000000000000000000000000000000000000000000000000000000000000000000000000000000000000000000000000000000000000000000000000000000000000000000000000000000000000000000000000000000000000000000000000000000

000000000000000000000000000000000000000000000000000000000000000000000000000000000000000000000000000000000000000000000000000000000000000000000000000000000000000000000000000000000000000000000000000000000000000000000000000000000000000000000000000000000000000000000000000000000000000000000000000000000000000000000000000000000000000000000000000000000000000000000000000000000000000000000000000000000000000000000000000000000000000000000000000000000000000000000000000000000000000000000000000000000000000000000000000000000000000000000000000000000000000000000000000000000000000000000000000000000000000000000000000000000000000000000000000000000000000000000000000000000000000000000000000000000000000000000000000000000000000000000000000000000000000000000000000000000000000000000000000000000000000000000000000000000000000000000000

>DP01070

MASSCAVQVKLELGHRAQVRKKPTVEGFTHDWMVFVRGPEHSNIQHFVEKVVFHLHESFPRPKRVCKDPPYKVEESGYAGFILPIEVYFKNKEEPRKVRFDYDLFLHLEGHPPVNHLRCEKLTFNNPTEDFRRKLLKAGGDPNRSIHTSSSSSSSSSSSSSSSSSSSSSSSSSSSSSSSSSSSSSSSSSSTSFSKPHKLMKEHKEKPSKDSREHKSAFKEPSRDHNKSSKESSKKPKENKPLKEEKIVPKMAFKEPKPMSKEPKPDSNLLTITSGQDKKAPSKRPPISDSEELSAKKRKKSSSEALFKSFSSAPPLILTCSADKKQIKDKSHVKMGKVKIESETSEKKKSTLPPFDDIVDPNDSDVEENISSKSDSEQPSPASSSSSSSSSFTPSQTRQQGPLRSIMKDLHSDDNEEESDEVEDNDNDSEMERPVNRGGSRSRRVSLSDGSDSESSSASSPLHHEPPPPLLKTNNNQILEVKSPIKQSKSDKQIKNGECDKAYLDELVELHRRLMTLRERHILQQIVNLIEETGHFHITNTTFDFDLCSLDKTTVRKLQSYLETSGTS

0000000000000000000000000000000000000000000000000000000000000000000000000000000000000000000000000000000000000000000000000000000000000000000000000000000000000000000000000000000000000000000000000000000000000000000000000000000000000000000000000000000000000000000000000000000000000000000000000000000000000000000000000000000000000000000000000000000000000000000000000000000000000000000000000000000000000000000000000000000000000000000000000000000000000000000000000000000000000000000000000000000001111111111111111111111111111111111111111111111111111111111111111111111111111111

0000000000000000000000000000000000000000000000000000000000000000000000000000000000000000000000000000000000000000000000000000000000000000000000000000000000000000000000000000000000000000000000000000000000000000000000000000000000000000000000000000000000000000000000000000000000000000000000000000000000000000000000000000000000000000000000000000000000000000000000000000000000000000000000000000000000000000000000000000000000000000000000000000000000000000000000000000000000000000000000000000000000000000000000000000000000000000000000000000000000000000000000000000000000000000

0000000000000000000000000000000000000000000000000000000000000000000000000000000000000000000000000000000000000000000000000000000000000000000000000000000000000000000000000000000000000000000000000000000000000000000000000000000000000000000000000000000000000000000000000000000000000000000000000000000000000000000000000000000000000000000000000000000000000000000000000000000000000000000000000000000000000000000000000000000000000000000000000000000000000000000000000000000000000000000000000000000000000000000000000000000000000000000000000000000000000000000000000000000000000000

0000000000000000000000000000000000000000000000000000000000000000000000000000000000000000000000000000000000000000000000000000000000000000000000000000000000000000000000000000000000000000000000000000000000000000000000000000000000000000000000000000000000000000000000000000000000000000000000000000000000000000000000000000000000000000000000000000000000000000000000000000000000000000000000000000000000000000000000000000000000000000000000000000000000000000000000000000000000000000000000000000000000000000000000000000000000000000000000000000000000000000000000000000000000000000

0000000000000000000000000000000000000000000000000000000000000000000000000000000000000000000000000000000000000000000000000000000000000000000000000000000000000000000000000000000000000000000000000000000000000000000000000000000000000000000000000000000000000000000000000000000000000000000000000000000000000000000000000000000000000000000000000000000000000000000000000000000000000000000000000000000000000000000000000000000000000000000000000000000000000000000000000000000000000000000000000000000000000000000000000000000000000000000000000000000000000000000000000000000000000000

>DP01052

MLDSLYGDLPPPSKDEGCPPKPTSLLGSLYDDLDDTPDTSSSSSASSSSSSSSALFASSSWTAERLQMLTPAVVRKQTLKSASGLGAASSQTAGQASSSKPPLDVAAVAAAASAAAAAVAAQLKAVKSEAFGDRARSLFGRGEREKAEALGASDEAASAPGVAGGVKRDESEKGESLRALGRLGVETAEGRRGHQGGDKETESSGFVSIGQMKDEYDPGTPNDYSAIYREKIRKQQREELERLRQQELDKQRRDRELPGSGSSLSGVAFGDSPSTPSPPAAPPAPPVLPSYIDPAKKSFAARMMEKMGWKHGEGLGVNKQGITAPLVAKKTAMSSGVIVQGAEVVQAAAQVWAQFNRPPTRVLLLLNMVGRGEVDEDLKEETEEEAAKFGNLLQVKIVEAAEAPDDEAVRIFCEYERKEEATRAFMTMNGRVFGGRTVKGRFYCEERWGKDDLMPNPEEEADKPLD

0000000000000000000000000000000000000000000000000000000000000000000000000000000000000000000000000000000000000000000000000000000000000000000000000000000000000000000000000000000000000000000000000000000000000000000000000000000000000000000000000000000000000000000000000000000000000000001111111111111111111111111111111111111111111111111111111111111000000000000000000000000000000000000000000000000000000000000000000000000000000000000000000000000000000000000000000000000000

0000000000000000000000000000000000000000000000000000000000000000000000000000000000000000000000000000000000000000000000000000000000000000000000000000000000000000000000000000000000000000000000000000000000000000000000000000000000000000000000000000000000000000000000000000000000000000000000000000000000000000000000000000000000000000000000000000000000000000000000000000000000000000000000000000000000000000000000000000000000000000000000000000000000000000000000000000000000

0000000000000000000000000000000000000000000000000000000000000000000000000000000000000000000000000000000000000000000000000000000000000000000000000000000000000000000000000000000000000000000000000000000000000000000000000000000000000000000000000000000000000000000000000000000000000000000000000000000000000000000000000000000000000000000000000000000000000000000000000000000000000000000000000000000000000000000000000000000000000000000000000000000000000000000000000000000000

0000000000000000000000000000000000000000000000000000000000000000000000000000000000000000000000000000000000000000000000000000000000000000000000000000000000000000000000000000000000000000000000000000000000000000000000000000000000000000000000000000000000000000000000000000000000000000000000000000000000000000000000000000000000000000000000000000000000000000000000000000000000000000000000000000000000000000000000000000000000000000000000000000000000000000000000000000000000

0000000000000000000000000000000000000000000000000000000000000000000000000000000000000000000000000000000000000000000000000000000000000000000000000000000000000000000000000000000000000000000000000000000000000000000000000000000000000000000000000000000000000000000000000000000000000000000000000000000000000000000000000000000000000000000000000000000000000000000000000000000000000000000000000000000000000000000000000000000000000000000000000000000000000000000000000000000000

>DP00949

MPAERKKPASMEEKDSLPNNKEKDCSERRTVSSKERPKDDIKLTAKKEVSKAPEDKKKRLEDDKRKKEDKERKKKDEEKVKAEEESKKKEEEEKKKHQEEERKKQEEQAKRQQEEEAAAQMKEKEESIQLHQEAWERHHLRKELRSKNQNAPDSRPEENFFSRLDSSLKKNTAFVKKLKTITEQQRDSLSHDFNGLNLSKYIAEAVASIVEAKLKISDVNCAVHLCSLFHQRYADFAPSLLQVWKKHFEARKEEKTPNITKLRTDLRFIAELTIVGIFTDKEGLSLIYEQLKNIINADRESHTHVSVVISFCRHCGDDIAGLVPRKVKSAAEKFNLSFPPSEIISPEKQQPFQNLLKEYFTSLTKHLKRDHRELQNTERQNRRILHSKGELSEDRHKQYEEFAMSYQKLLANSQSLADLLDENMPDLPQDKPTPEEHGPGIDIFTPGKPGEYDLEGGIWEDEDARNFYENLIDLKAFVPAILFKDNEKSCQNKESNKDDTKEAKESKENKEVSSPDDLELELENLEINDDTLELEGGDEAEDLTKKLLDEQEQEDEEASTGSHLKLIVDAFLQQLPNCVNRDLIDKAAMDFCMNMNTKANRKKLVRALFIVPRQRLDLLPFYARLVATLHPCMSDVAEDLCSMLRGDFRFHVRKKDQINIETKNKTVRFIGELTKFKMFTKNDTLHCLKMLLSDFSHHHIEMACTLLETCGRFLFRSPESHLRTSVLLEQMMRKKQAMHLDARYVTMVENAYYYCNPPPAEKTVKKKRPPLQEYVRKLLYKDLSKVTTEKVLRQMRKLPWQDQEVKDYVICCMINIWNVKYNSIHCVANLLAGLVLYQEDVGIHVVDGVLEDIRLGMEVNQPKFNQRRISSAKFLGELYNYRMVESAVIFRTLYSFTSFGVNPDGSPSSLDPPEHLFRIRLVCTILDTCGQYFDRGSSKRKLDCFLVYFQRYVWWKKSLEVWTKDHPFPIDIDYMISDTLELLRPKIKLCNSLEESIRQVQDLEREFLIKLGLVNDKDSKDSMTEGENLEEDEEEEEGGAETEEQSGNESEVNEPEEEEGSDNDDDEGEEEEEENTDYLTDSNKENETDEENTEVMIKGGGLKHVPCVEDEDFIQALDKMMLENLQQRSGESVKVHQLDVAIPLHLKSQLRKGPPLGGGEGEAESADTMPFVMLTRKGNKQQFKILNVPMSSQLAANHWNQQQAEQEERMRMKKLTLDINERQEQEDYQEMLQSLAQRPAPANTNRERRPRYQHPKGAPNADLIFKTGGRRR

000000000000000000000000000000000000000000000000000000000000000000000000000000000000000000000000000000000000000000000000000000000000000000000000000000000000000000000000000000000000000000000000000000000000000000000000000000000000000000000000000000000000000000000000000000000000000000000000000000000000000000000000000000000000000000000000000000000000000000000000000000000000000000000000000000000000000000000000000000000000000000000000000000000000000000000000000000000000000000000000000000000000000000000000000000000000000000000000000000000000000000000000000000000000000000000000000000000000000000000000000000000000000000000000000000000000000000000000000000000000000000000000000000000000000000000000000000000000000000000000000000000000000000000000000000000000000000000000000000000000000000000000000000000000000000000000000000000000000000000000000000000000000000000000000000000000000000000000000000000000000000000000000000000000000000000000000000000000000000000000000000000000000000000000000000000000000000000000000000000000000000000000000000000000000000000000000000000000000000000000000000000000000000000000111111111111111111111111100000000000000000000000000000000000001111111111111111111111111111111111111111100000000000000000000000000000000000000000000000000000000000000000

000000000000000000000000000000000000000000000000000000000000000000000000000000000000000000000000000000000000000000000000000000000000000000000000000000000000000000000000000000000000000000000000000000000000000000000000000000000000000000000000000000000000000000000000000000000000000000000000000000000000000000000000000000000000000000000000000000000000000000000000000000000000000000000000000000000000000000000000000000000000000000000000000000000000000000000000000000000000000000000000000000000000000000000000000000000000000000000000000000000000000000000000000000000000000000000000000000000000000000000000000000000000000000000000000000000000000000000000000000000000000000000000000000000000000000000000000000000000000000000000000000000000000000000000000000000000000000000000000000000000000000000000000000000000000000000000000000000000000000000000000000000000000000000000000000000000000000000000000000000000000000000000000000000000000000000000000000000000000000000000000000000000000000000000000000000000000000000000000000000000000000000000000000000000000000000000000000000000000000000000000000000000000000000000000000000000000000000000000000000000000000000000000000000000000000000000000000000000000000000000000000000000000000000000000000000000000000000000000000000000000000000000

000000000000000000000000000000000000000000000000000000000000000000000000000000000000000000000000000000000000000000000000000000000000000000000000000000000000000000000000000000000000000000000000000000000000000000000000000000000000000000000000000000000000000000000000000000000000000000000000000000000000000000000000000000000000000000000000000000000000000000000000000000000000000000000000000000000000000000000000000000000000000000000000000000000000000000000000000000000000000000000000000000000000000000000000000000000000000000000000000000000000000000000000000000000000000000000000000000000000000000000000000000000000000000000000000000000000000000000000000000000000000000000000000000000000000000000000000000000000000000000000000000000000000000000000000000000000000000000000000000000000000000000000000000000000000000000000000000000000000000000000000000000000000000000000000000000000000000000000000000000000000000000000000000000000000000000000000000000000000000000000000000000000000000000000000000000000000000000000000000000000000000000000000000000000000000000000000000000000000000000000000000000000000000000000000000000000000000000000000000000000000000000000000000000000000000000000000000000000000000000000000000000000000000000000000000000000000000000000000000000000000000000000

000000000000000000000000000000000000000000000000000000000000000000000000000000000000000000000000000000000000000000000000000000000000000000000000000000000000000000000000000000000000000000000000000000000000000000000000000000000000000000000000000000000000000000000000000000000000000000000000000000000000000000000000000000000000000000000000000000000000000000000000000000000000000000000000000000000000000000000000000000000000000000000000000000000000000000000000000000000000000000000000000000000000000000000000000000000000000000000000000000000000000000000000000000000000000000000000000000000000000000000000000000000000000000000000000000000000000000000000000000000000000000000000000000000000000000000000000000000000000000000000000000000000000000000000000000000000000000000000000000000000000000000000000000000000000000000000000000000000000000000000000000000000000000000000000000000000000000000000000000000000000000000000000000000000000000000000000000000000000000000000000000000000000000000000000000000000000000000000000000000000000000000000000000000000000000000000000000000000000000000000000000000000000000000000000000000000000000000000000000000000000000000000000000000000000000000000000000000000000000000000000000000000000000000000000000000000000000000000000000000000000000000000

000000000000000000000000000000000000000000000000000000000000000000000000000000000000000000000000000000000000000000000000000000000000000000000000000000000000000000000000000000000000000000000000000000000000000000000000000000000000000000000000000000000000000000000000000000000000000000000000000000000000000000000000000000000000000000000000000000000000000000000000000000000000000000000000000000000000000000000000000000000000000000000000000000000000000000000000000000000000000000000000000000000000000000000000000000000000000000000000000000000000000000000000000000000000000000000000000000000000000000000000000000000000000000000000000000000000000000000000000000000000000000000000000000000000000000000000000000000000000000000000000000000000000000000000000000000000000000000000000000000000000000000000000000000000000000000000000000000000000000000000000000000000000000000000000000000000000000000000000000000000000000000000000000000000000000000000000000000000000000000000000000000000000000000000000000000000000000000000000000000000000000000000000000000000000000000000000000000000000000000000000000000000000000000000000000000000000000000000000000000000000000000000000000000000000000000000000000000000000000000000000000000000000000000000000000000000000000000000000000000000000000000000

>DP00073

MPVAGSELPRRPLPPAAQERDAEPRPPHGELQYLGQIQHILRCGVRKDDRTGTGTLSVFGMQARYSLRDEFPLLTTKRVFWKGVLEELLWFIKGSTNAKELSSKGVKIWDANGSRDFLDSLGFSTREEGDLGPVYGFQWRHFGAEYRDMESDYSGQGVDQLQRVIDTIKTNPDDRRIIMCAWNPRDLPLMALPPCHALCQFYVVNSELSCQLYQRSGDMGLGVPFNIASYALLTYMIAHITGLKPGDFIHTLGDAHIYLNHIEPLKIQLQREPRPFPKLRILRKVEKIDDFKAEDFQIEGYNPHPTIKMEMAV

1111111111111111111111111000000000000000000000000000000000000000000000000000000000000000000000000000000000000000000000000000000000000000000000000000000000000000000000000000000000000000000000000000000000000000000000000000000000000000000000000000000000000000000000000000000000000000000000000000000000000000000000000

0000000000000000000000000000000000000000000000000000000000000000000000000000000000000000000000000000000000000000000000000000000000000000000000000000000000000000000000000000000000000000000000000000000000000000000000000000000000000000000000000000000000000000000000000000000000000000000000000000000000000000000000000

0000000000000000000000000000000000000000000000000000000000000000000000000000000000000000000000000000000000000000000000000000000000000000000000000000000000000000000000000000000000000000000000000000000000000000000000000000000000000000000000000000000000000000000000000000000000000000000000000000000000000000000000000

0000000000000000000000000000000000000000000000000000000000000000000000000000000000000000000000000000000000000000000000000000000000000000000000000000000000000000000000000000000000000000000000000000000000000000000000000000000000000000000000000000000000000000000000000000000000000000000000000000000000000000000000000

0000000000000000000000000000000000000000000000000000000000000000000000000000000000000000000000000000000000000000000000000000000000000000000000000000000000000000000000000000000000000000000000000000000000000000000000000000000000000000000000000000000000000000000000000000000000000000000000000000000000000000000000000

>DP00423

MAPKKSTTKTTSKGKKPATSKGKEKSTSKAAIKKTTAKKEEASSKSYRELIIEGLTALKERKGSSRPALKKFIKENYPIVGSASNFDLYFNNAIKKGVEAGDFEQPKGPAGAVKLAKKKSPEVKKEKEVSPKPKQAATSVSATASKAKAASTKLAPKKVVKKKSPTVTAKKASSPSSLTYKEMILKSMPQLNDGKGSSRIVLKKYVKDTFSSKLKTSSNFDYLFNSAIKKCVENGELVQPKGPSGIIKLNKKKVKLST

000000000000000000000000000000000000000000000000000000000000000000000000000000000000000000000000000000000000000000000000000000000000000000000000000000000000000000000000001111111111111111111111111111111111111111111111111111111111111111111111111111111111111111

000000000000000000000000000000000000000000000000000000000000000000000000000000000000000000000000000000000000000000000000000000000000000000000000000000000000000000000000000000000000000000000000000000000000000000000000000000000000000000000000000000000000000000

000000000000000000000000000000000000000000000000000000000000000000000000000000000000000000000000000000000000000000000000000000000000000000000000000000000000000000000000000000000000000000000000000000000000000000000000000000000000000000000000000000000000000000

000000000000000000000000000000000000000000000000000000000000000000000000000000000000000000000000000000000000000000000000000000000000000000000000000000000000000000000000000000000000000000000000000000000000000000000000000000000000000000000000000000000000000000

000000000000000000000000000000000000000000000000000000000000000000000000000000000000000000000000000000000000000000000000000000000000000000000000000000000000000000000000000000000000000000000000000000000000000000000000000000000000000000000000000000000000000000

>DP00140

MAHKKAGGSTRNGRDSEAKRLGVKRFGGESVLAGSIIVRQRGTKFHAGANVGCGRDHTLFAKADGKVKFEVKGPKNRKFISIEAE

1111111111111111111111111111111111111111111111111111111111111111111111111111111111111

0000000000000000000000000000000000000000000000000000000000000000000000000000000000000

0000000000000000000000000000000000000000000000000000000000000000000000000000000000000

0000000000000000000000000000000000000000000000000000000000000000000000000000000000000

0000000000000000000000000000000000000000000000000000000000000000000000000000000000000

>DP00803

MASLATLAAVQPTTLKGLAGSSIAGTKLHIKPARQSFKLNNVRSGAIVAKYGDKSVYFDLEDIANTTGQWDVYGSDAPSPYNSLQSKFFETFAAPFTKRGLLLKFLILGGGSLLTYVSANAPQDVLPITRGPQQPPKLGPRGKI

111111111111111111111111111111111111111111111111111111111100000000000000000000000000000000000000000000000000000000000000000000000000000000000000

000000000000000000000000000000000000000000000000000000000000000000000000000000000000000000000000000000000000000000000000000000000000000000000000

000000000000000000000000000000000000000000000000000000000000000000000000000000000000000000000000000000000000000000000000000000000000000000000000

000000000000000000000000000000000000000000000000000000000000000000000000000000000000000000000000000000000000000000000000000000000000000000000000

000000000000000000000000000000000000000000000000000000000000000000000000000000000000000000000000000000000000000000000000000000000000000000000000

>DP02547

MAAVGRVGSFGSSPPGLSSTYTGGPLGNEIASGNGGAAAGDDEDGQNLWSCILSEVSTRSRSKLPAGKNVLLLGEDGAGKTSLIRKIQGIEEYKKGRGLEYLYLNVHDEDRDDQTRCNVWILDGDLYHKGLLKFSLDAVSLKDTLVMLVVDMSKPWTALDSLQKWASVVREHVDKLKIPPEEMKQMEQKLIRDFQEYVEPGEDFPASPQRRNTASQEDKDDSVVLPLGADTLTHNLGIPVLVVCTKCDAISVLEKEHDYRDEHFDFIQSHIRKFCLQYGAALIYTSVKENKNIDLVYKYIVQKLYGFPYKIPAVVVEKDAVFIPAGWDNDKKIGILHENFQTLKAEDNFEDIITKPPVRKFVHEKEIMAEDDQVFLMKLQSLLAKQPPTAAGRPVDASPRVPGGSPRTPNRSVSSNVASVSPIPAGSKKIDPNMKAGATSEGVLANFFNSLLSKKTGSPGGPGVSGGSPAGGAGGGSSGLPPSTKKSGQKPVLDVHAELDRITRKPVTVSPTTPTSPTEGEAS

0000000000000000000000000000000000000000000000000000000000000000000000000000000000000000000000000000000000000000000000000000000000000000000000000000000000000000000000000000000000000000000000000000000000000000000000000000000000000000000000000000000000000000000000000000000000000000000000000000000000000000000000000000000000000000000000000000000000000000000000000000000000000000000000000001111111111111111111111111111111111111111111111111111111111111111111111111111111111111111111111111111111111111111111111111111111111111111

0000000000000000000000000000000000000000000000000000000000000000000000000000000000000000000000000000000000000000000000000000000000000000000000000000000000000000000000000000000000000000000000000000000000000000000000000000000000000000000000000000000000000000000000000000000000000000000000000000000000000000000000000000000000000000000000000000000000000000000000000000000000000000000000000000000000000000000000000000000000000000000000000000000000000000000000000000000000000000000000000000000000000000000000000000000000000000000

0000000000000000000000000000000000000000000000000000000000000000000000000000000000000000000000000000000000000000000000000000000000000000000000000000000000000000000000000000000000000000000000000000000000000000000000000000000000000000000000000000000000000000000000000000000000000000000000000000000000000000000000000000000000000000000000000000000000000000000000000000000000000000000000000000000000000000000000000000000000000000000000000000000000000000000000000000000000000000000000000000000000000000000000000000000000000000000

0000000000000000000000000000000000000000000000000000000000000000000000000000000000000000000000000000000000000000000000000000000000000000000000000000000000000000000000000000000000000000000000000000000000000000000000000000000000000000000000000000000000000000000000000000000000000000000000000000000000000000000000000000000000000000000000000000000000000000000000000000000000000000000000000000000000000000000000000000000000000000000000000000000000000000000000000000000000000000000000000000000000000000000000000000000000000000000

0000000000000000000000000000000000000000000000000000000000000000000000000000000000000000000000000000000000000000000000000000000000000000000000000000000000000000000000000000000000000000000000000000000000000000000000000000000000000000000000000000000000000000000000000000000000000000000000000000000000000000000000000000000000000000000000000000000000000000000000000000000000000000000000000000000000000000000000000000000000000000000000000000000000000000000000000000000000000000000000000000000000000000000000000000000000000000000

>DP02568

MAGRSGDSDEDLLKAVRLIKFLYQSNPPPNPEGTRQARRNRRRRWRERQRQIHSISERILSTYLGRSAEPVPLQLPPLERLTLDCNEDCGTSGTQGVGSPQILVESPTVLESGAKE

00000000000000000000000000000000000000000000000000000000000000000000000001111111111000000000000000000000000000000000

00000000000000000000000000000000000000000000000000000000000000000000000000000000000000000000000000000000000000000000

00000000000000000000000000000000000000000000000000000000000000000000000000000000000000000000000000000000000000000000

00000000000000000000000000000000000000000000000000000000000000000000000000000000000000000000000000000000000000000000

00000000000000000000000000000000000000000000000000000000000000000000000000000000000000000000000000000000000000000000

>DP00811

MGEPRSQPPVERPPTAETFLPLSRVRTIMKSSMDTGLITNEVLFLMTKCTELFVRHLAGAAYTEEFGQRPGEALKYEHLSQVVNKNKNLEFLLQIVPQKIRVHQFQEMLRLNRSAGSDDDDDDDDDDDEEESESESESDE

11111111111111111111111111110000000000000000000000000000000000000000000000000000000000000000000000001111111111111111111111111111111111111111

00000000000000000000000000000000000000000000000000000000000000000000000000000000000000000000000000000000000000000000000000000000000000000000

00000000000000000000000000000000000000000000000000000000000000000000000000000000000000000000000000000000000000000000000000000000000000000000

00000000000000000000000000000000000000000000000000000000000000000000000000000000000000000000000000000000000000000000000000000000000000000000

00000000000000000000000000000000000000000000000000000000000000000000000000000000000000000000000000000000000000000000000000000000000000000000

>DP01320

MSQYKTGLLLIHPAVTTTPELVENTKAQAASKKVKFVDQFLINKLNDGSITLENAKYETVHYLTPEAQTDIKFPKKLISVLADSLKPNGSLIGLSDIYKVDALINGFEIINEPDYCWIKMDSSKLNQTVSIPLKKKKTNNTKLQSGSKLPTFKKASSSTSNLPSFKKADHSRQPIVKETDSFKPPSFKMTTEPKVYRVVDDLIEDSDDDDFSSDSSKAQYFDQVDTSDDSIEEEELIDEDGSGKSMITMITCGKSKTKKKKACKDCTCGMKEQEENEINDIRSQQDKVVKFTEDELTEIDFTIDGKKVGGCGSCSLGDAFRCSGCPYLGLPAFKPGQPINLDSISDDL

000000000000000000000000000000000000000000000000000000000000000000000000000000000000000000000000000000000000000000000000000000000000000000000000000000000000000000000000000000000000000000000000000000000000000000000000000000000000000001111111111111111111111111111111111111111111111111111111111111111111111111111111111111111111111111111111111111111111

000000000000000000000000000000000000000000000000000000000000000000000000000000000000000000000000000000000000000000000000000000000000000000000000000000000000000000000000000000000000000000000000000000000000000000000000000000000000000000000000000000000000000000000000000000000000000000000000000000000000000000000000000000000000000000000000000000000000

000000000000000000000000000000000000000000000000000000000000000000000000000000000000000000000000000000000000000000000000000000000000000000000000000000000000000000000000000000000000000000000000000000000000000000000000000000000000000000000000000000000000000000000000000000000000000000000000000000000000000000000000000000000000000000000000000000000000

000000000000000000000000000000000000000000000000000000000000000000000000000000000000000000000000000000000000000000000000000000000000000000000000000000000000000000000000000000000000000000000000000000000000000000000000000000000000000000000000000000000000000000000000000000000000000000000000000000000000000000000000000000000000000000000000000000000000

000000000000000000000000000000000000000000000000000000000000000000000000000000000000000000000000000000000000000000000000000000000000000000000000000000000000000000000000000000000000000000000000000000000000000000000000000000000000000000000000000000000000000000000000000000000000000000000000000000000000000000000000000000000000000000000000000000000000

>DP01547

MPIMGSSVYITVELAIAVLAILGNVLVCWAVWLNSNLQNVTNYFVVSLAAADIAVGVLAIPFAITISTGFCAACHGCLFIACFVLVLTQSSIFSLLAIAIDRYIAIRIPLRYNGLVTGTRAKGIIAICWVLSFAIGLTPMLGWNNCGQPKEGKNHSQGCGEGQVACLFEDVVPMNYMVYFNFFACVLVPLLLMLGVYLRIFLAARRQLKQMESQPLPGERARSTLQKEVHAAKSLAIIVGLFALCWLPLHIINCFTFFCPDCSHAPLWLMYLAIVLSHTNSVVNPFIYAYRIREFRQTFRKIIRSHVLRQQEPFKAAGTSARVLAAHGSDGEQVSLRLNGHPPGVWANGSAPHPERRPNGYALGLVSGGSAQESQGNTGLPDVELLSHELKGVCPEPPGLDDPLAQDGAGVS

0000000000000000000000000000000000000000000000000000000000000000000000000000000000000000000000000000000000000000000000000000000000000000000000000000000000000000000000000000000000000000000000000000000000000000000000000000000000000000000000000000000000000000000000000000000000000000000000000000111111111111111111111111111111111111111111111111111111111111111111111111111111111111111111111111111111111111111111111111

0000000000000000000000000000000000000000000000000000000000000000000000000000000000000000000000000000000000000000000000000000000000000000000000000000000000000000000000000000000000000000000000000000000000000000000000000000000000000000000000000000000000000000000000000000000000000000000000000000000000000000000000000000000000000000000000000000000000000000000000000000000000000000000000000000000000000000000000000000

0000000000000000000000000000000000000000000000000000000000000000000000000000000000000000000000000000000000000000000000000000000000000000000000000000000000000000000000000000000000000000000000000000000000000000000000000000000000000000000000000000000000000000000000000000000000000000000000000000000000000000000000000000000000000000000000000000000000000000000000000000000000000000000000000000000000000000000000000000

0000000000000000000000000000000000000000000000000000000000000000000000000000000000000000000000000000000000000000000000000000000000000000000000000000000000000000000000000000000000000000000000000000000000000000000000000000000000000000000000000000000000000000000000000000000000000000000000000000000000000000000000000000000000000000000000000000000000000000000000000000000000000000000000000000000000000000000000000000

0000000000000000000000000000000000000000000000000000000000000000000000000000000000000000000000000000000000000000000000000000000000000000000000000000000000000000000000000000000000000000000000000000000000000000000000000000000000000000000000000000000000000000000000000000000000000000000000000000000000000000000000000000000000000000000000000000000000000000000000000000000000000000000000000000000000000000000000000000

>DP00332

MKTALILLSILGMACAFSMKNLHRRVKIEDSEENGVFKYRPRYYLYKHAYFYPHLKRFPVQGSSDSSEENGDDSSEEEEEEEETSNEGENNEESNEDEDSEAENTTLSATTLGYGEDATPGTGYTGLAAIQLPKKAGDITNKATKEKESDEEEEEEEEGNENEESEAEVDENEQGINGTSTNSTEAENGNGSSGGDNGEEGEEESVTGANAEDTTETGRQGKGTSKTTTSPNGGFEPTTPPQVYRTTSPPFGKTTTVEYEGEYEYTGANEYDNGYEIYESENGEPRGDNYRAYEDEYSYFKGQGYDGYDGQNYYHHQ

11111111111111111111111111111111111111111111111111111111111111111111111111111111111111111111111111111111111111111111111111111111111111111111111111111111111111111111111111111111111111111111111111111111111111111111111111111111111111111111111111111111111111111111111111111111111111111111111111111111111111111111111111111

00000000000000000000000000000000000000000000000000000000000000000000000000000000000000000000000000000000000000000000000000000000000000000000000000000000000000000000000000000000000000000000000000000000000000000000000000000000000000000000000000000000000000000000000000000000000000000000000000000000000000000000000000000

00000000000000000000000000000000000000000000000000000000000000000000000000000000000000000000000000000000000000000000000000000000000000000000000000000000000000000000000000000000000000000000000000000000000000000000000000000000000000000000000000000000000000000000000000000000000000000000000000000000000000000000000000000

00000000000000000000000000000000000000000000000000000000000000000000000000000000000000000000000000000000000000000000000000000000000000000000000000000000000000000000000000000000000000000000000000000000000000000000000000000000000000000000000000000000000000000000000000000000000000000000000000000000000000000000000000000

00000000000000000000000000000000000000000000000000000000000000000000000000000000000000000000000000000000000000000000000000000000000000000000000000000000000000000000000000000000000000000000000000000000000000000000000000000000000000000000000000000000000000000000000000000000000000000000000000000000000000000000000000000

>DP00564

MPLHVSLANGNRDLDYDSVQPYFMCDDEEEDVHHQQPPQPPAPSEDIWKKFELLPTPRPSPGHAGLYSPPCEAVAVSFAPRDHDGDSFSIADLPELPGGDAVKQSFVCDPDDETFVKNIILQDCMWNGFSASAKLVSKLDPYQAVRKEGTGVSLAADVEPATPPDCTCNT

01111111111111111111111111111111111111111111111111111111111111111111111111111111111111111111111111111111111111111111111111111111111111111111111111111111111111111111111110

00000000000000000000000000000000000000000000000000000000000000000000000000000000000000000000000000000000000000000000000000000000000000000000000000000000000000000000000000

00000000000000000000000000000000000000000000000000000000000000000000000000000000000000000000000000000000000000000000000000000000000000000000000000000000000000000000000000

00000000000000000000000000000000000000000000000000000000000000000000000000000000000000000000000000000000000000000000000000000000000000000000000000000000000000000000000000

00000000000000000000000000000000000000000000000000000000000000000000000000000000000000000000000000000000000000000000000000000000000000000000000000000000000000000000000000

>DP01023

MPRGFLVKRSKKSTPVSYRVRGGEDGDRALLLSPSCGGARAEPPAPSPVPGPLPPPPPAERAHAALAAALACAPGPQPPPQGPRAAHFGNPEAAHPAPLYSPTRPVSREHEKHKYFERSFNLGSPVSAESFPTPAALLGGGGGGGASGAGGGGTCGGDPLLFAPAELKMGTAFSAGAEAARGPGPGPPLPPAAALRPPGKRPPPPTAAEPPAKAVKAPGAKKPKAIRKLHFEDEVTTSPVLGLKIKEGPVEAPRGRAGGAARPLGEFICQLCKEEYADPFALAQHKCSRIVRVEYRCPECAKVFSCPANLASHRRWHKPRPAPAAARAPEPEAAARAEAREAPGGGSDRDTPSPGGVSESGSEDGLYECHHCAKKFRRQAYLRKHLLAHHQALQAKGAPLAPPAEDLLALYPGPDEKAPQEAAGDGEGAGVLGLSASAECHLCPVCGESFASKGAQERHLRLLHAAQVFPCKYCPATFYSSPGLTRHINKCHPSENRQVILLQVPVRPAC

111111111111111111110000000000000000000000000000000000000000000000000000000000000000000000000000000000000000000000000000000000000000000000000000000000000000000000000000000000000000000000000000000000000000000000000000000000000000000000000000000000000000000000000000000000000000000000000000000000000000000000000000000000000000000000000000000000000000000000000000000000000000000000000000000000000000000000000000000000000000000000000000000000000000000000000000000000000000000000000000000000000000000000000000000000

000000000000000000000000000000000000000000000000000000000000000000000000000000000000000000000000000000000000000000000000000000000000000000000000000000000000000000000000000000000000000000000000000000000000000000000000000000000000000000000000000000000000000000000000000000000000000000000000000000000000000000000000000000000000000000000000000000000000000000000000000000000000000000000000000000000000000000000000000000000000000000000000000000000000000000000000000000000000000000000000000000000000000000000000000000

000000000000000000000000000000000000000000000000000000000000000000000000000000000000000000000000000000000000000000000000000000000000000000000000000000000000000000000000000000000000000000000000000000000000000000000000000000000000000000000000000000000000000000000000000000000000000000000000000000000000000000000000000000000000000000000000000000000000000000000000000000000000000000000000000000000000000000000000000000000000000000000000000000000000000000000000000000000000000000000000000000000000000000000000000000

000000000000000000000000000000000000000000000000000000000000000000000000000000000000000000000000000000000000000000000000000000000000000000000000000000000000000000000000000000000000000000000000000000000000000000000000000000000000000000000000000000000000000000000000000000000000000000000000000000000000000000000000000000000000000000000000000000000000000000000000000000000000000000000000000000000000000000000000000000000000000000000000000000000000000000000000000000000000000000000000000000000000000000000000000000

000000000000000000000000000000000000000000000000000000000000000000000000000000000000000000000000000000000000000000000000000000000000000000000000000000000000000000000000000000000000000000000000000000000000000000000000000000000000000000000000000000000000000000000000000000000000000000000000000000000000000000000000000000000000000000000000000000000000000000000000000000000000000000000000000000000000000000000000000000000000000000000000000000000000000000000000000000000000000000000000000000000000000000000000000000

>DP00387

MKKRNRLKKNEDFQKVFKHGTSVANRQFVLYTLDQPENDELRVGLSVSKKIGNAVMRNRIKRLIRQAFLEEKERLKEKDYIIIARKPASQLTYEETKKSLQHLFRKSSLYKKSSSK

11111111111111111111111111111111111111111111111111111111111111111111111111111111111111111111111111111111111111111111

00000000000000000000000000000000000000000000000000000000000000000000000000000000000000000000000000000000000000000000

00000000000000000000000000000000000000000000000000000000000000000000000000000000000000000000000000000000000000000000

00000000000000000000000000000000000000000000000000000000000000000000000000000000000000000000000000000000000000000000

00000000000000000000000000000000000000000000000000000000000000000000000000000000000000000000000000000000000000000000

>DP02490

MSTVLVRRKERRQPPQMPRGEILLESPPELPEVVTNSFQNVLMYLPMAAGSAAMVFTFLNHRNTLQLVAGGMFALSMFGMMFGQLSQQSGERKTKLNSARRDYLRYLGQVRQRVRKAAKQQREALEWNNPAPGRLWSMVMSPRLWERRSSDADFAQVRIGAGPQRLAVQLIPPETKPVEDLEPMSAGALRRFLRAHSTVPDLPVAISLRSFARILPDGDPKAVYGMVRALIMQLAAFHSPDDVRITVCASRERMPQWQWMKWLPHSLHPTEYDAAGQVRLLTHSLVELESMLGPEIKDRGMFGASRAPAEPFHLVIVDGGQASYDSQIASDGIDGVCVIDLTGSVAETNEATMLRLRVTPERVYVVKRDRAGKEVLSSVGRPDQASIAEAEALARQLAPFRTSAADEPEEDVLSANMTLTSLLHIDNPYNLDPAVLWRPRPQRNRLRVPIGLDADGRPLELDIKESAQGGMGPHGLCIGATGSGKSELLRTLVLALAMTHSPEVLNFVLVDFKGGATFLGMEGLRHVSAIITNLEEELPLVDRMYDALHGEMVRRQEHLRHSGNYASLRDYEKARMEGAPLPPMPTLFIVLDEFSELLSAKPDFAELFVMIGRLGRSLGVHLLLASQRLEEGKLRGLDTHLSYRIGLRTFSAMESRVVLGVPDAYELPPSPGNGYLKFATEPLVRFKAAYVSGPVDEEPQTRSEGPQIVRQVLPYLTDYIRPQVVEQPQPEQRAEENKSSESLFDVVVRQLAGHGPEPHQIWLPPLDVPPTLDELLPPLSPSAAHGYTADGWEWRGRLHAVVGLVDRPFDQRRDPYWLDLSGGAGHVGVAGGPQTGKSTMLRTLITSLALLHTPQEVQFYCLDFGGGTLAGLAELPHVGSVATRLDADRIRRTVAEVSALLEQREQEFTERGIDSMATYRRLRATGEYAGDGFGDVFLVVDNWLTLRQDYEALEDSITQLAARGLGYGIHVVLSSNKWSEFRTSIRDLLGTKLELRLGDPYESEVDRKKAANVPENRPGRGLTRDGYHFLTALPRIDGDTSAETLTEGIATTVKTIREAWHGPTAPPVRMLPNVLPAAQLPSAAESGTRIPIGIDEDSLSPVYLDFNTDPHFLVFGDTECGKSNLLRLITAGIIERYTPQQARLIFIDYSRSLLDVATTEHQIGYAASSTAASSLVRDIKGAMEARLPPPDLTPEQLRSRSWWTGAELFLVVDDYEMVATSDNPLRPLAELLPQARDIGLHLIIARSMGGAGRALYEPIIQRIKEMASPGLVMSGNKDEGILLGNVKPHKLPQGRGYFVERRSGTRLIQTAYRES

0000000000000000000000000000000000000000000000000000000000000000000000000000000000000000000000000000000000000000000000000000000000000000000000000000000000000000000000000000000000000000000000000000000111111111111111111111111111111111111111111111111111111111111111111111111111111111111111111111111111111111111111111111111111111111111111111111111111111111111111111111111111111111111111111111111111111111111111111111100000000000000000000000000000000000000000000000000000000000000000000000000000000000000000000000000000000000000000000000000000000000000000000000000000000000000000000000000000000000000000000000000000000000000000000000000000000000000000000000000000000000000000000000000000000000000000000000000000000000000000000000000000000000000000000000000000000000000000000000000000000000000000000000000000000000000000000000000000000000000000000000000000000000000000000000000000000000000000000000000000000000000000000000000000000000000000000000000000000000000000000000000000000000000000000000000000000000000000000000000000000000000000000000000000000000000000000000000000000000000000000000000000000000000000000000000000000000000000000000000000000000000000000000000000000000000000000000000000000000000000000000000000000000000000000000000000000000000000000000000000000000000000000000000000000000000000000000000000000000000

0000000000000000000000000000000000000000000000000000000000000000000000000000000000000000000000000000000000000000000000000000000000000000000000000000000000000000000000000000000000000000000000000000000000000000000000000000000000000000000000000000000000000000000000000000000000000000000000000000000000000000000000000000000000000000000000000000000000000000000000000000000000000000000000000000000000000000000000000000000000000000000000000000000000000000000000000000000000000000000000000000000000000000000000000000000000000000000000000000000000000000000000000000000000000000000000000000000000000000000000000000000000000000000000000000000000000000000000000000000000000000000000000000000000000000000000000000000000000000000000000000000000000000000000000000000000000000000000000000000000000000000000000000000000000000000000000000000000000000000000000000000000000000000000000000000000000000000000000000000000000000000000000000000000000000000000000000000000000000000000000000000000000000000000000000000000000000000000000000000000000000000000000000000000000000000000000000000000000000000000000000000000000000000000000000000000000000000000000000000000000000000000000000000000000000000000000000000000000000000000000000000000000000000000000000000000000000000000000000000000000000000000000000000000000000000000000000000000000000000

0000000000000000000000000000000000000000000000000000000000000000000000000000000000000000000000000000000000000000000000000000000000000000000000000000000000000000000000000000000000000000000000000000000000000000000000000000000000000000000000000000000000000000000000000000000000000000000000000000000000000000000000000000000000000000000000000000000000000000000000000000000000000000000000000000000000000000000000000000000000000000000000000000000000000000000000000000000000000000000000000000000000000000000000000000000000000000000000000000000000000000000000000000000000000000000000000000000000000000000000000000000000000000000000000000000000000000000000000000000000000000000000000000000000000000000000000000000000000000000000000000000000000000000000000000000000000000000000000000000000000000000000000000000000000000000000000000000000000000000000000000000000000000000000000000000000000000000000000000000000000000000000000000000000000000000000000000000000000000000000000000000000000000000000000000000000000000000000000000000000000000000000000000000000000000000000000000000000000000000000000000000000000000000000000000000000000000000000000000000000000000000000000000000000000000000000000000000000000000000000000000000000000000000000000000000000000000000000000000000000000000000000000000000000000000000000000000000000000000000

0000000000000000000000000000000000000000000000000000000000000000000000000000000000000000000000000000000000000000000000000000000000000000000000000000000000000000000000000000000000000000000000000000000000000000000000000000000000000000000000000000000000000000000000000000000000000000000000000000000000000000000000000000000000000000000000000000000000000000000000000000000000000000000000000000000000000000000000000000000000000000000000000000000000000000000000000000000000000000000000000000000000000000000000000000000000000000000000000000000000000000000000000000000000000000000000000000000000000000000000000000000000000000000000000000000000000000000000000000000000000000000000000000000000000000000000000000000000000000000000000000000000000000000000000000000000000000000000000000000000000000000000000000000000000000000000000000000000000000000000000000000000000000000000000000000000000000000000000000000000000000000000000000000000000000000000000000000000000000000000000000000000000000000000000000000000000000000000000000000000000000000000000000000000000000000000000000000000000000000000000000000000000000000000000000000000000000000000000000000000000000000000000000000000000000000000000000000000000000000000000000000000000000000000000000000000000000000000000000000000000000000000000000000000000000000000000000000000000000000

0000000000000000000000000000000000000000000000000000000000000000000000000000000000000000000000000000000000000000000000000000000000000000000000000000000000000000000000000000000000000000000000000000000000000000000000000000000000000000000000000000000000000000000000000000000000000000000000000000000000000000000000000000000000000000000000000000000000000000000000000000000000000000000000000000000000000000000000000000000000000000000000000000000000000000000000000000000000000000000000000000000000000000000000000000000000000000000000000000000000000000000000000000000000000000000000000000000000000000000000000000000000000000000000000000000000000000000000000000000000000000000000000000000000000000000000000000000000000000000000000000000000000000000000000000000000000000000000000000000000000000000000000000000000000000000000000000000000000000000000000000000000000000000000000000000000000000000000000000000000000000000000000000000000000000000000000000000000000000000000000000000000000000000000000000000000000000000000000000000000000000000000000000000000000000000000000000000000000000000000000000000000000000000000000000000000000000000000000000000000000000000000000000000000000000000000000000000000000000000000000000000000000000000000000000000000000000000000000000000000000000000000000000000000000000000000000000000000000000000

>DP00077

MANVEKPNDCSGFPVVDLNSCFSNGFNNEKQEIEMETDDSPILLMSSSASRENSNTFSVIQRTPDGKIITTNNNMNSKINKQLDKLPENLRLNGRTPSGKLRSFVCEVCTRAFARQEHLKRHYRSHTNEKPYPCGLCNRCFTRRDLLIRHAQKIHSGNLGETISHTKKVSRTITKARKNSASSVKFQTPTYGTPDNGNFLNRTTANTRRKASPEANVKRKYLKKLTRRASFSAQSASSYALPDQSSLEQHPKDRVKFSTPELVPLDLKNPELDSSFDLNMNLDLNLNLDSNFNIALNRSDSSGSTMNLDYKLPESANNYTYSSGSPTRAYVGANTNSKNASFNDADLLSSSYWIKAYNDHLFSVSESDETSPMNSELNDTKLIVPDFKSTIHHLKDSRSSSWTVAIDNNSNNNKVSDNQPDFVDFQELLDNDTLGNDLLETTAVLKEFELLHDDSVSATATSNEIDLSHLNLSNSPISPHKLIYKNKEGTNDDMLISFGLDHPSNREDDLDKLCNMTRDVQAIFSQYLKGEESKRSLEDFLSTSNRKEKPDSGNYTFYGLDCLTLSKISRALPASTVNNNQPSHSIESKLFNEPMRNMCIKVLRYYEKFSHDSSESVMDSNPNLLSKELLMPAVSELNEYLDLFKNNFLPHFPIIHPSLLDLDLDSLQRYTNEDGYDDAENAQLFDRLSQGTDKEYDYEHYQILSISKIVCLPLFMATFGSLHKFGYKSQTIELYEMSRRILHSFLETKRRCRSTTVNDSYQNIWLMQSLILSFMFALVADYLEKIDSSLMKRQLSALCSTIRSNCLPTISANSEKSINNNNEPLTFGSPLQYIIFESKIRCTLMAYDFCQFLKCFFHIKFDLSIKEKDVETIYIPDNESKWASESIICNGHVVQKQNFYDFRNFYYSFTYGHLHSIPEFLGSSMIYYEYDLRKGTKSHVFLDRIDTKRLERSLDTSSYGNDNMAATNKNIAILIDDTIILKNNLMSMRFIKQIDRSFTEKVRKGQIAKIYDSFLNSVRLNFLKNYSVEVLCEFLVALNFSIRNISSLYVEEESDCSQRMNSPELPRIHLNNQALSVFNLQGYYYCFILIIKFLLDFEATPNFKLLRIFIELRSLANSILLPTLSRLYPQEFSGFPDVVFTQQFINKDNGMLVPGLSANEHHNGASAAVKTKLAKKINVEGLAMFINEILVNSFNDTSFLNMEDPIRNEFSFDNGDRAVTDLPRSAHFLSDTGLEGINFSGLNDSHQTVSTLNLLRYGENHSSKHKNGGKGQGFAEKYQLSLKYVTIAKLFFTNVKENYIHCHMLDKMASDFHTLENHLKGNS

000000000000000000000000000000000000000000000000000000000000000000000000001111111111111111111111111111100000000000000000000000000000000000000000000000000000000000000000000000000000000000000000000000000000000000000000000000000000000000000000000000000000000000000000000000000000000000000000000000000000000000000000000000000000000000000000000000000000000000000000000000000000000000000000000000000000000000000000000000000000000000000000000000000000000000000000000000000000000000000000000000000000000000000000000000000000000000000000000000000000000000000000000000000000000000000000000000000000000000000000000000000000000000000000000000000000000000000000000000000000000000000000000000000000000000000000000000000000000000000000000000000000000000000000000000000000000000000000000000000000000000000000000000000000000000000000000000000000000000000000000000000000000000000000000000000000000000000000000000000000000000000000000000000000000000000000000000000000000000000000000000000000000000000000000000000000000000000000000000000000000000000000000000000000000000000000000000000000000000000000000000000000000000000000000000000000000000000000000000000000000000000000000000000000000000000000000000000000000000000000000000000000000000000000000000000000000000000000000000000000000000000000000000000000000000000000000000000000000000000000000

000000000000000000000000000000000000000000000000000000000000000000000000000000000000000000000000000000000000000000000000000000000000000000000000000000000000000000000000000000000000000000000000000000000000000000000000000000000000000000000000000000000000000000000000000000000000000000000000000000000000000000000000000000000000000000000000000000000000000000000000000000000000000000000000000000000000000000000000000000000000000000000000000000000000000000000000000000000000000000000000000000000000000000000000000000000000000000000000000000000000000000000000000000000000000000000000000000000000000000000000000000000000000000000000000000000000000000000000000000000000000000000000000000000000000000000000000000000000000000000000000000000000000000000000000000000000000000000000000000000000000000000000000000000000000000000000000000000000000000000000000000000000000000000000000000000000000000000000000000000000000000000000000000000000000000000000000000000000000000000000000000000000000000000000000000000000000000000000000000000000000000000000000000000000000000000000000000000000000000000000000000000000000000000000000000000000000000000000000000000000000000000000000000000000000000000000000000000000000000000000000000000000000000000000000000000000000000000000000000000000000000000000000000000000000000000000000000000000000000000000000

000000000000000000000000000000000000000000000000000000000000000000000000000000000000000000000000000000000000000000000000000000000000000000000000000000000000000000000000000000000000000000000000000000000000000000000000000000000000000000000000000000000000000000000000000000000000000000000000000000000000000000000000000000000000000000000000000000000000000000000000000000000000000000000000000000000000000000000000000000000000000000000000000000000000000000000000000000000000000000000000000000000000000000000000000000000000000000000000000000000000000000000000000000000000000000000000000000000000000000000000000000000000000000000000000000000000000000000000000000000000000000000000000000000000000000000000000000000000000000000000000000000000000000000000000000000000000000000000000000000000000000000000000000000000000000000000000000000000000000000000000000000000000000000000000000000000000000000000000000000000000000000000000000000000000000000000000000000000000000000000000000000000000000000000000000000000000000000000000000000000000000000000000000000000000000000000000000000000000000000000000000000000000000000000000000000000000000000000000000000000000000000000000000000000000000000000000000000000000000000000000000000000000000000000000000000000000000000000000000000000000000000000000000000000000000000000000000000000000000000000000

000000000000000000000000000000000000000000000000000000000000000000000000000000000000000000000000000000000000000000000000000000000000000000000000000000000000000000000000000000000000000000000000000000000000000000000000000000000000000000000000000000000000000000000000000000000000000000000000000000000000000000000000000000000000000000000000000000000000000000000000000000000000000000000000000000000000000000000000000000000000000000000000000000000000000000000000000000000000000000000000000000000000000000000000000000000000000000000000000000000000000000000000000000000000000000000000000000000000000000000000000000000000000000000000000000000000000000000000000000000000000000000000000000000000000000000000000000000000000000000000000000000000000000000000000000000000000000000000000000000000000000000000000000000000000000000000000000000000000000000000000000000000000000000000000000000000000000000000000000000000000000000000000000000000000000000000000000000000000000000000000000000000000000000000000000000000000000000000000000000000000000000000000000000000000000000000000000000000000000000000000000000000000000000000000000000000000000000000000000000000000000000000000000000000000000000000000000000000000000000000000000000000000000000000000000000000000000000000000000000000000000000000000000000000000000000000000000000000000000000000000

000000000000000000000000000000000000000000000000000000000000000000000000000000000000000000000000000000000000000000000000000000000000000000000000000000000000000000000000000000000000000000000000000000000000000000000000000000000000000000000000000000000000000000000000000000000000000000000000000000000000000000000000000000000000000000000000000000000000000000000000000000000000000000000000000000000000000000000000000000000000000000000000000000000000000000000000000000000000000000000000000000000000000000000000000000000000000000000000000000000000000000000000000000000000000000000000000000000000000000000000000000000000000000000000000000000000000000000000000000000000000000000000000000000000000000000000000000000000000000000000000000000000000000000000000000000000000000000000000000000000000000000000000000000000000000000000000000000000000000000000000000000000000000000000000000000000000000000000000000000000000000000000000000000000000000000000000000000000000000000000000000000000000000000000000000000000000000000000000000000000000000000000000000000000000000000000000000000000000000000000000000000000000000000000000000000000000000000000000000000000000000000000000000000000000000000000000000000000000000000000000000000000000000000000000000000000000000000000000000000000000000000000000000000000000000000000000000000000000000000000000

>DP00893

MLDFFTIFSKGGLVLWCFQGVSDSCTGPVNALIRSVLLQERGGNNSFTHEALTLKYKLDNQFELVFVVGFQKILTLTYVDKLIDDVHRLFRDKYRTEIQQQSALSLLNGTFDFQNDFLRLLREAEESSKIRAPTTMKKFEDSEKAKKPVRSMIETRGEKPKEKAKNSKKKGAKKEGSDGPLATSKPVPAEKSGLPVGPENGVELSKEELIRRKREEFIQKHGRGMEKSNKSTKSDAPKEKGKKAPRVWELGGCANKEVLDYSTPTTNGTPEAALSEDINLIRGTGSGGQLQDLDCSSSDDEGAAQNSTKPSATKGTLGGMFGMLKGLVGSKSLSREDMESVLDKMRDHLIAKNVAADIAVQLCESVANKLEGKVMGTFSTVTSTVKQALQESLVQILQPQRRVDMLRDIMDAQRRQRPYVVTFCGVNGVGKSTNLAKISFWLLENGFSVLIAACDTFRAGAVEQLRTHTRRLSALHPPEKHGGRTMVQLFEKGYGKDAAGIAMEAIAFARNQGFDVVLVDTAGRMQDNAPLMTALAKLITVNTPDLVLFVGEALVGNEAVDQLVKFNRALADHSMAQTPRLIDGIVLTKFDTIDDKVGAAISMTYITSKPIVFVGTGQTYCDLRSLNAKAVVAALMKA

00000000000000000000000000000000000000000000000000000000000000000000000000000000000000000000000000000000000000000000000000000000011111111111111111111111111111111111111111111111000000000000000000000000000000000000000000000000000000000000000000000000000000000000000000000000000000000000000000000000000000000000000000000000000000000000000000000000000000000000000000000000000000000000000000000000000000000000000000000000000000000000000000000000000000000000000000000000000000000000000000000000000000000000000000000000000000000000000000000000000000000000000000000000000000000000000000000000000000000000000000000000000000000000000000000000000000

00000000000000000000000000000000000000000000000000000000000000000000000000000000000000000000000000000000000000000000000000000000000000000000000000000000000000000000000000000000000000000000000000000000000000000000000000000000000000000000000000000000000000000000000000000000000000000000000000000000000000000000000000000000000000000000000000000000000000000000000000000000000000000000000000000000000000000000000000000000000000000000000000000000000000000000000000000000000000000000000000000000000000000000000000000000000000000000000000000000000000000000000000000000000000000000000000000000000000000000000000000000000000000000000000000000000000

00000000000000000000000000000000000000000000000000000000000000000000000000000000000000000000000000000000000000000000000000000000000000000000000000000000000000000000000000000000000000000000000000000000000000000000000000000000000000000000000000000000000000000000000000000000000000000000000000000000000000000000000000000000000000000000000000000000000000000000000000000000000000000000000000000000000000000000000000000000000000000000000000000000000000000000000000000000000000000000000000000000000000000000000000000000000000000000000000000000000000000000000000000000000000000000000000000000000000000000000000000000000000000000000000000000000000

00000000000000000000000000000000000000000000000000000000000000000000000000000000000000000000000000000000000000000000000000000000000000000000000000000000000000000000000000000000000000000000000000000000000000000000000000000000000000000000000000000000000000000000000000000000000000000000000000000000000000000000000000000000000000000000000000000000000000000000000000000000000000000000000000000000000000000000000000000000000000000000000000000000000000000000000000000000000000000000000000000000000000000000000000000000000000000000000000000000000000000000000000000000000000000000000000000000000000000000000000000000000000000000000000000000000000

00000000000000000000000000000000000000000000000000000000000000000000000000000000000000000000000000000000000000000000000000000000000000000000000000000000000000000000000000000000000000000000000000000000000000000000000000000000000000000000000000000000000000000000000000000000000000000000000000000000000000000000000000000000000000000000000000000000000000000000000000000000000000000000000000000000000000000000000000000000000000000000000000000000000000000000000000000000000000000000000000000000000000000000000000000000000000000000000000000000000000000000000000000000000000000000000000000000000000000000000000000000000000000000000000000000000000

>DP00145

MATVNQLVRKPRARKVAKSNVPALEACPQKRGVCTRVYTTTPKKPNSALRKVCRVRLTNGFEVTSYIGGEGHNLQEHSVILIRGGRVKDLPGVRYHTVRGALDCSGVKDRKQARSKYGVKRPKA

1111111111111111111111111110000000000000000000000000000000000000000000000000000000000000000000000000000000000000000000000000

0000000000000000000000000000000000000000000000000000000000000000000000000000000000000000000000000000000000000000000000000000

0000000000000000000000000000000000000000000000000000000000000000000000000000000000000000000000000000000000000000000000000000

0000000000000000000000000000000000000000000000000000000000000000000000000000000000000000000000000000000000000000000000000000

0000000000000000000000000000000000000000000000000000000000000000000000000000000000000000000000000000000000000000000000000000

>DP01063

MAPRTLWSCYLCCLLTAAAGAASYPPRGFSLYTGSSGALSPGGPQAQIAPRPASRHRNWCAYVVTRTVSCVLEDGVETYVKYQPCAWGQPQCPQSIMYRRFLRPRYRVAYKTVTDMEWRCCQGYGGDDCAESPAPALGPASSTPRPLARPARPNLSGSSAGSPLSGLGGEGPGESEKVQQLEEQVQSLTKELQGLRGVLQGLSGRLAEDVQRAVETAFNGRQQPADAAARPGVHETLNEIQHQLQLLDTRVSTHDQELGHLNNHHGGSSSSGGSRAPAPASAPPGPSEELLRQLEQRLQESCSVCLAGLDGFRRQQQEDRERLRAMEKLLASVEERQRHLAGLAVGRRPPQECCSPELGRRLAELERRLDVVAGSVTVLSGRRGTELGGAAGQGGHPPGYTSLASRLSRLEDRFNSTLGPSEEQEESWPGAPGGLSHWLPAARGRLEQLGGLLANVSGELGGRLDLLEEQVAGAMQACGQLCSGAPGEQDSQVSEILSALERRVLDSEGQLRLVGSGLHTVEAAGEARQATLEGLQEVVGRLQDRVDAQDETAAEFTLRLNLTAARLGQLEGLLQAHGDEGCGACGGVQEELGRLRDGVERCSCPLLPPRGPGAGPGVGGPSRGPLDGFSVFGGSSGSALQALQGELSEVILSFSSLNDSLNELQTTVEGQGADLADLGATKDRIISEINRLQQEATEHATESEERFRGLEEGQAQAGQCPSLEGRLGRLEGVCERLDTVAGGLQGLREGLSRHVAGLWAGLRETNTTSQMQAALLEKLVGGQAGLGRRLGALNSSLQLLEDRLHQLSLKDLTGPAGEAGPPGPPGLQGPPGPAGPPGSPGKDGQEGPIGPPGPQGEQGVEGAPAAPVPQVAFSAALSLPRSEPGTVPFDRVLLNDGGYYDPETGVFTAPLAGRYLLSAVLTGHRHEKVEAVLSRSNQGVARVDSGGYEPEGLENKPVAESQPSPGTLGVFSLILPLQAGDTVCVDLVMGQLAHSEEPLTIFSGALLYGDPELEHA

00000000000000000000000000000000000000000000000000000000000000000000000000000000000000000000000000000000000000000000000000000000000000000000000000000000000000000000000000000000000000000000000000000000000000000000000000000000000000000000000000000000000000000000000000000000000000000000000000000000000000000000000000000000000000000000000000000000000000000000000000000000000000000000000000000000000000000000000000000000000000000000000000000000000000000000000000000000000000000000000000000000000000000000000000000000000000000000000000000000000000000000000000000000000000000000000000000000000000000000000000000000000000000000000000000000000000000000000000000000000000000000000000000000000000000000000000000000000000000000000000000000000000000000000000000000000000000000000000000000000000000000000000000000000000000000000000000000000000000000000000000000000000000000000000000000000000000000000000000000000000000000000000000000000000000000000000000000000000111111000000000000000000000000000000000000000000000000000000000000

00000000000000000000000000000000000000000000000000000000000000000000000000000000000000000000000000000000000000000000000000000000000000000000000000000000000000000000000000000000000000000000000000000000000000000000000000000000000000000000000000000000000000000000000000000000000000000000000000000000000000000000000000000000000000000000000000000000000000000000000000000000000000000000000000000000000000000000000000000000000000000000000000000000000000000000000000000000000000000000000000000000000000000000000000000000000000000000000000000000000000000000000000000000000000000000000000000000000000000000000000000000000000000000000000000000000000000000000000000000000000000000000000000000000000000000000000000000000000000000000000000000000000000000000000000000000000000000000000000000000000000000000000000000000000000000000000000000000000000000000000000000000000000000000000000000000000000000000000000000000000000000000000000000000000000000000000000000000000000000000000000000000000000000000000000000000000000000000000000000

00000000000000000000000000000000000000000000000000000000000000000000000000000000000000000000000000000000000000000000000000000000000000000000000000000000000000000000000000000000000000000000000000000000000000000000000000000000000000000000000000000000000000000000000000000000000000000000000000000000000000000000000000000000000000000000000000000000000000000000000000000000000000000000000000000000000000000000000000000000000000000000000000000000000000000000000000000000000000000000000000000000000000000000000000000000000000000000000000000000000000000000000000000000000000000000000000000000000000000000000000000000000000000000000000000000000000000000000000000000000000000000000000000000000000000000000000000000000000000000000000000000000000000000000000000000000000000000000000000000000000000000000000000000000000000000000000000000000000000000000000000000000000000000000000000000000000000000000000000000000000000000000000000000000000000000000000000000000000000000000000000000000000000000000000000000000000000000000000000000

00000000000000000000000000000000000000000000000000000000000000000000000000000000000000000000000000000000000000000000000000000000000000000000000000000000000000000000000000000000000000000000000000000000000000000000000000000000000000000000000000000000000000000000000000000000000000000000000000000000000000000000000000000000000000000000000000000000000000000000000000000000000000000000000000000000000000000000000000000000000000000000000000000000000000000000000000000000000000000000000000000000000000000000000000000000000000000000000000000000000000000000000000000000000000000000000000000000000000000000000000000000000000000000000000000000000000000000000000000000000000000000000000000000000000000000000000000000000000000000000000000000000000000000000000000000000000000000000000000000000000000000000000000000000000000000000000000000000000000000000000000000000000000000000000000000000000000000000000000000000000000000000000000000000000000000000000000000000000000000000000000000000000000000000000000000000000000000000000000000

00000000000000000000000000000000000000000000000000000000000000000000000000000000000000000000000000000000000000000000000000000000000000000000000000000000000000000000000000000000000000000000000000000000000000000000000000000000000000000000000000000000000000000000000000000000000000000000000000000000000000000000000000000000000000000000000000000000000000000000000000000000000000000000000000000000000000000000000000000000000000000000000000000000000000000000000000000000000000000000000000000000000000000000000000000000000000000000000000000000000000000000000000000000000000000000000000000000000000000000000000000000000000000000000000000000000000000000000000000000000000000000000000000000000000000000000000000000000000000000000000000000000000000000000000000000000000000000000000000000000000000000000000000000000000000000000000000000000000000000000000000000000000000000000000000000000000000000000000000000000000000000000000000000000000000000000000000000000000000000000000000000000000000000000000000000000000000000000000000000

>DP01475

MTNETIDQTRTPDQTQSQTAFDPQQFINNLQVAFIKVDNVVASFDPDQKPIVDKNDRDNRQAFDGISQLREEYSNKAIKNPTKKNQYFSDFIDKSNDLINKDNLIDVESSTKSFQKFGDQRYQIFTSWVSHQKDPSKINTRSIRNFMENIIQPPIPDDKEKAEFLKSAKQSFAGIIIGNQIRTDQKFMGVFDESLKERQEAEKNGGPTGGDWLDIFLSFIFNKKQSSDVKEAINQEPVPHVQPDIATTTTDIQGLPPEARDLLDERGNFSKFTLGDMEMLDVEGVADIDPNYKFNQLLIHNNALSSVLMGSHNGIEPEKVSLLYAGNGGFGDKHDWNATVGYKDQQGNNVATLINVHMKNGSGLVIAGGEKGINNPSFYLYKEDQLTGSQRALSQEEIRNKVDFMEFLAQNNTKLDNLSEKEKEKFQNEIEDFQKDSKAYLDALGNDRIAFVSKKDTKHSALITEFNNGDLSYTLKDYGKKADKALDREKNVTLQGSLKHDGVMFVDYSNFKYTNASKNPNKGVGATNGVSHLEAGFNKVAVFNLPDLNNLAITSFVRRNLENKLTAKGLSLQEANKLIKDFLSSNKELAGKALNFNKAVAEAKSTGNYDEVKKAQKDLEKSLRKREHLEKEVEKKLESKSGNKNKMEAKAQANSQKDEIFALINKEANRDARAIAYTQNLKGIKRELSDKLEKISKDLKDFSKSFDEFKNGKNKDFSKAEETLKALKGSVKDLGINPEWISKVENLNAALNEFKNGKNKDFSKVTQAKSDLENSVKDVIINQKVTDKVDNLNQAVSVAKAMGDFSRVEQVLADLKNFSKEQLAQQAQKNEDFNTGKNSELYQSVKNSVNKTLVGNGLSGIEATALAKNFSDIKKELNEKFKNFNNNNNGLKNSTEPIYAKVNKKKTGQVASPEEPIYTQVAKKVNAKIDRLNQIASGLGGVGQAAGFPLKRHDKVDDLSKVGLSASPEPIYATIDDLGGPFPLKRHDKVDDLSKVGRSRNQELAQKIDNLNQAVSEAKAGFFGNLEQTIDKLKDSTKKNVMNLYVESAKKVPASLSAKLDNYAINSHTRINSNIQNGAINEKATGMLTQKNPEWLKLVNDKIVAHNVGSVSLSEYDKIGFNQKNMKDYSDSFKFSTKLNNAVKDIKSGFTHFLANAFSTGYYCLARENAEHGIKNVNTKGGFQKS

0000000000000000000000000000000000000000000000000000000000000000000000000000000000000000000000000000000000000000000000000000000000000000000000000000000000000000000000000000000000000000000000000000000000000000000000000000000000000000000000000000000000000000000000000000000000000000000000000000000000000000000000000000000000000000000000000000000000000000000000000000000000000000000000000000000000000000000000000000000000000000000000000000000000000000000000000000000000000000000000000000000000000000000000000000000000000000000000000000000000000000000000000000000000000000000000000000000000000000000000000000000000000000000000000000000000000000000000000000000000000000000000000000000000000000000000000000000000000000000000000000000000000000000000000000000000000000000000000000000000000000000000000000000000000000000000000000000000000000000000000000000000000000000000000000000000001111111111111111111111111111111111111111111111111111111111111111111111111111111111111111111111111111111111111111111111111111111111111111111111111111111111111111111111111111111111111111111111111111111111111111111111111111111111111111111111111111111111111111111111111111111111111111111111111111111111111111111111

0000000000000000000000000000000000000000000000000000000000000000000000000000000000000000000000000000000000000000000000000000000000000000000000000000000000000000000000000000000000000000000000000000000000000000000000000000000000000000000000000000000000000000000000000000000000000000000000000000000000000000000000000000000000000000000000000000000000000000000000000000000000000000000000000000000000000000000000000000000000000000000000000000000000000000000000000000000000000000000000000000000000000000000000000000000000000000000000000000000000000000000000000000000000000000000000000000000000000000000000000000000000000000000000000000000000000000000000000000000000000000000000000000000000000000000000000000000000000000000000000000000000000000000000000000000000000000000000000000000000000000000000000000000000000000000000000000000000000000000000000000000000000000000000000000000000000000000000000000000000000000000000000000000000000000000000000000000000000000000000000000000000000000000000000000000000000000000000000000000000000000000000000000000000000000000000000000000000000000000000000000000000000000000000000000000000000000000000000000000000000000000000000000000000000000000000000000000000

0000000000000000000000000000000000000000000000000000000000000000000000000000000000000000000000000000000000000000000000000000000000000000000000000000000000000000000000000000000000000000000000000000000000000000000000000000000000000000000000000000000000000000000000000000000000000000000000000000000000000000000000000000000000000000000000000000000000000000000000000000000000000000000000000000000000000000000000000000000000000000000000000000000000000000000000000000000000000000000000000000000000000000000000000000000000000000000000000000000000000000000000000000000000000000000000000000000000000000000000000000000000000000000000000000000000000000000000000000000000000000000000000000000000000000000000000000000000000000000000000000000000000000000000000000000000000000000000000000000000000000000000000000000000000000000000000000000000000000000000000000000000000000000000000000000000000000000000000000000000000000000000000000000000000000000000000000000000000000000000000000000000000000000000000000000000000000000000000000000000000000000000000000000000000000000000000000000000000000000000000000000000000000000000000000000000000000000000000000000000000000000000000000000000000000000000000000000000

0000000000000000000000000000000000000000000000000000000000000000000000000000000000000000000000000000000000000000000000000000000000000000000000000000000000000000000000000000000000000000000000000000000000000000000000000000000000000000000000000000000000000000000000000000000000000000000000000000000000000000000000000000000000000000000000000000000000000000000000000000000000000000000000000000000000000000000000000000000000000000000000000000000000000000000000000000000000000000000000000000000000000000000000000000000000000000000000000000000000000000000000000000000000000000000000000000000000000000000000000000000000000000000000000000000000000000000000000000000000000000000000000000000000000000000000000000000000000000000000000000000000000000000000000000000000000000000000000000000000000000000000000000000000000000000000000000000000000000000000000000000000000000000000000000000000000000000000000000000000000000000000000000000000000000000000000000000000000000000000000000000000000000000000000000000000000000000000000000000000000000000000000000000000000000000000000000000000000000000000000000000000000000000000000000000000000000000000000000000000000000000000000000000000000000000000000000000000

0000000000000000000000000000000000000000000000000000000000000000000000000000000000000000000000000000000000000000000000000000000000000000000000000000000000000000000000000000000000000000000000000000000000000000000000000000000000000000000000000000000000000000000000000000000000000000000000000000000000000000000000000000000000000000000000000000000000000000000000000000000000000000000000000000000000000000000000000000000000000000000000000000000000000000000000000000000000000000000000000000000000000000000000000000000000000000000000000000000000000000000000000000000000000000000000000000000000000000000000000000000000000000000000000000000000000000000000000000000000000000000000000000000000000000000000000000000000000000000000000000000000000000000000000000000000000000000000000000000000000000000000000000000000000000000000000000000000000000000000000000000000000000000000000000000000000000000000000000000000000000000000000000000000000000000000000000000000000000000000000000000000000000000000000000000000000000000000000000000000000000000000000000000000000000000000000000000000000000000000000000000000000000000000000000000000000000000000000000000000000000000000000000000000000000000000000000000000

>DP00487

MPKETPSKAAADALSDLEIKDSKSNLNKELETLREENRVKSDMLKEKLSKDAENHKAYLKSHQVHRHKLKEMEKEEPLLNEDKERTVLFPIKYHEIWQAYKRAEASFWTAEEIDLSKDIHDWNNRMNENERFFISRVLAFFAASDGIVNENLVENFSTEVQIPEAKSFYGFQIMIENIHSETYSLLIDTYIKDPKESEFLFNAIHTIPEIGEKAEWALRWIQDADALFGERLVAFASIEGVFFSGSFASIFWLKKRGMMPGLTFSNELICRDEGLHTDFACLLFAHLKNKPDPAIVEKIVTEAVEIEQRYFLDALPVALLGMNADLMNQYVEFVADRLLVAFGNKKYYKVENPFDFMENISLAGKTNFFEKRVSDYQKAGVMSKSTKQEAGAFTFNEDF

111111111111111111111111111111111111111110000000000000000000000000000000000000000000000000000000000000000000000000000000000000000000000000000000000000000000000000000000000000000000000000000000000000000000000000000000000000000000000000000000000000000000000000000001111111111100000000000000000000000000000000000000000000000000000000000000000000000000000000000000000000000000000000000000000000000000000

000000000000000000000000000000000000000000000000000000000000000000000000000000000000000000000000000000000000000000000000000000000000000000000000000000000000000000000000000000000000000000000000000000000000000000000000000000000000000000000000000000000000000000000000000000000000000000000000000000000000000000000000000000000000000000000000000000000000000000000000000000000000000000000000000000000000000

000000000000000000000000000000000000000000000000000000000000000000000000000000000000000000000000000000000000000000000000000000000000000000000000000000000000000000000000000000000000000000000000000000000000000000000000000000000000000000000000000000000000000000000000000000000000000000000000000000000000000000000000000000000000000000000000000000000000000000000000000000000000000000000000000000000000000

000000000000000000000000000000000000000000000000000000000000000000000000000000000000000000000000000000000000000000000000000000000000000000000000000000000000000000000000000000000000000000000000000000000000000000000000000000000000000000000000000000000000000000000000000000000000000000000000000000000000000000000000000000000000000000000000000000000000000000000000000000000000000000000000000000000000000

000000000000000000000000000000000000000000000000000000000000000000000000000000000000000000000000000000000000000000000000000000000000000000000000000000000000000000000000000000000000000000000000000000000000000000000000000000000000000000000000000000000000000000000000000000000000000000000000000000000000000000000000000000000000000000000000000000000000000000000000000000000000000000000000000000000000000

>DP01069

MKRLLVLSILLTLGFSFAGTASADENANRPVNPGEDPNEAFRSTPFEATTSALGDCRECIAYRTGATTGKGSRRHDDTVSREIKGSSATPGGSEKAGTGRQ

11111111111111111111111111111111111111111111111111111111111111111111111111111111111111111111111111111

00000000000000000000000000000000000000000000000000000000000000000000000000000000000000000000000000000

00000000000000000000000000000000000000000000000000000000000000000000000000000000000000000000000000000

00000000000000000000000000000000000000000000000000000000000000000000000000000000000000000000000000000

00000000000000000000000000000000000000000000000000000000000000000000000000000000000000000000000000000

>DP00201

MILQRLFRLSSAVQSAISVSWRRNIGITAVAFNKELDPVQKLFVDKIREYRTKRQTSGGPVDAGPEYQQDLDRELFKLKQMYGKADMNTFPNFTFEDPKFEVVEKPQS

000000000000000000000000000000001111111111111111111111111111111111111111111111111111111111111111111111111111

000000000000000000000000000000000000000000000000000000000000000000000000000000000000000000000000000000000000

000000000000000000000000000000000000000000000000000000000000000000000000000000000000000000000000000000000000

000000000000000000000000000000000000000000000000000000000000000000000000000000000000000000000000000000000000

000000000000000000000000000000000000000000000000000000000000000000000000000000000000000000000000000000000000

>DP02782

MESLLQHLDRFSELLAVSSTTYVSTWDPATVRRALQWARYLRHIHRRFGRHGPIRTALERRLHNQWRQEGGFGRGPVPGLANFQALGHCDVLLSLRLLENRALGDAARYHLVQQLFPGPGVRDADEETLQESLARLARRRSAVHMLRFNGYRENPNLQEDSLMKTQAELLLERLQEVGKAEAERPARFLSSLWERLPQNNFLKVIAVALLQPPLSRRPQEELEPGIHKSPGEGSQVLVHWLLGNSEVFAAFCRALPAGLLTLVTSRHPALSPVYLGLLTDWGQRLHYDLQKGIWVGTESQDVPWEELHNRFQSLCQAPPPLKDKVLTALETCKAQDGDFEVPGLSIWTDLLLALRSGAFRKRQVLGLSAGLSSV

00000000000000000000000000000000000000000000000000000000000000000000000000000000000000000000000000000000000000000000000000000000000000000000000000000000000111111111111111111111111111111111111111111111111111111111111111111111111111111111111111111111111111111111111111111111111111111111111111111111111111111111111111111111111111111111111111111111111111111111100000000000000000

00000000000000000000000000000000000000000000000000000000000000000000000000000000000000000000000000000000000000000000000000000000000000000000000000000000000000000000000000000000000000000000000000000000000000000000000000000000000000000000000000000000000000000000000000000000000000000000000000000000000000000000000000000000000000000000000000000000000000000000000000000000000000

00000000000000000000000000000000000000000000000000000000000000000000000000000000000000000000000000000000000000000000000000000000000000000000000000000000000000000000000000000000000000000000000000000000000000000000000000000000000000000000000000000000000000000000000000000000000000000000000000000000000000000000000000000000000000000000000000000000000000000000000000000000000000

00000000000000000000000000000000000000000000000000000000000000000000000000000000000000000000000000000000000000000000000000000000000000000000000000000000000000000000000000000000000000000000000000000000000000000000000000000000000000000000000000000000000000000000000000000000000000000000000000000000000000000000000000000000000000000000000000000000000000000000000000000000000000

00000000000000000000000000000000000000000000000000000000000000000000000000000000000000000000000000000000000000000000000000000000000000000000000000000000000000000000000000000000000000000000000000000000000000000000000000000000000000000000000000000000000000000000000000000000000000000000000000000000000000000000000000000000000000000000000000000000000000000000000000000000000000

>DP00226

MASVLEELQKDLEEVKVLLEKSTRKRLRDTLTSEKSKIETELKNKMQQKSQKKPELDNEKPAAVVAPLTTGYTVKISNYGWDQSDKFVKIYITLTGVHQVPTENVQVHFTERSFDLLVKNLNGKNYSMIVNNLLKPISVESSSKKVKTDTVIILCRKKAENTRWDYLTQVEKECKEKEKPSYDTEADPSEGLMNVLKKIYEDGDDDMKRTINKAWVESREKQAREDTEF

0000000000000000000000000000000000000000000000011111111111111111111111111111100000000000000000000000000000000000000000000000000000000000000000000000000000000000000000000000000001111111111111111111111111111111111111111111111111111

0000000000000000000000000000000000000000000000000000000000000000000000000000000000000000000000000000000000000000000000000000000000000000000000000000000000000000000000000000000000000000000000000000000000000000000000000000000000000

0000000000000000000000000000000000000000000000000000000000000000000000000000000000000000000000000000000000000000000000000000000000000000000000000000000000000000000000000000000000000000000000000000000000000000000000000000000000000

0000000000000000000000000000000000000000000000000000000000000000000000000000000000000000000000000000000000000000000000000000000000000000000000000000000000000000000000000000000000000000000000000000000000000000000000000000000000000

0000000000000000000000000000000000000000000000000000000000000000000000000000000000000000000000000000000000000000000000000000000000000000000000000000000000000000000000000000000000000000000000000000000000000000000000000000000000000

>DP00187

MANLSYWLLALFVAMWTDVGLCKKRPKPGGWNTGGSRYPGQGSPGGNRYPPQGGGTWGQPHGGGWGQPHGGGWGQPHGGGWGQPHGGGWGQGGGTHNQWNKPSKPKTNMKHMAGAAAAGAVVGGLGGYMLGSAMSRPMMHFGNDWEDRYYRENMNRYPNQVYYRPVDQYNNQNNFVHDCVNITIKQHTVTTTTKGENFTETDIKIMERVVEQMCTTQYQKESQAYYDGRRSSAVLFSSPPVILLISFLIFLMVG

00000000000000000000000000000000000000000000000000000000000000000000000000000000000000000111111111111111111111111111111111111100000000000000000000000000000000000000000000000000000000000000000000000000000000000000000000000000000000000000000000000000000000

00000000000000000000000000000000000000000000000000000000000000000000000000000000000000000000000000000000000000000000000000000000000000000000000000000000000000000000000000000000000000000000000000000000000000000000000000000000000000000000000000000000000000

00000000000000000000000000000000000000000000000000000000000000000000000000000000000000000000000000000000000000000000000000000000000000000000000000000000000000000000000000000000000000000000000000000000000000000000000000000000000000000000000000000000000000

00000000000000000000000000000000000000000000000000000000000000000000000000000000000000000000000000000000000000000000000000000000000000000000000000000000000000000000000000000000000000000000000000000000000000000000000000000000000000000000000000000000000000

00000000000000000000000000000000000000000000000000000000000000000000000000000000000000000000000000000000000000000000000000000000000000000000000000000000000000000000000000000000000000000000000000000000000000000000000000000000000000000000000000000000000000

>DP01014

MSTDVAAAQAQSKIDLTKKKNEEINKKSLEEDDEFEDFPIDTWANGETIKSNAVTQTNIWEENWDDVEVDDDFTNELKAELDRYKRENQ

11111111111111111111111111111111111111111111111111111111111111111111111111111111111111111

00000000000000000000000000000000000000000000000000000000000000000000000000000000000000000

00000000000000000000000000000000000000000000000000000000000000000000000000000000000000000

00000000000000000000000000000000000000000000000000000000000000000000000000000000000000000

00000000000000000000000000000000000000000000000000000000000000000000000000000000000000000

>DP00348

MAENLLDGPPNPKRAKLSSPGFSANDNTDFGSLFDLENDLPDELIPNGELSLLNSGNLVPDAASKHKQLSELLRGGSGSSINPGIGNVSASSPVQQGLGGQAQGQPNSTNMASLGAMGKSPLNQGDSSTPNLPKQAASTSGPTPPASQALNPQAQKQVGLVTSSPATSQTGPGICMNANFNQTHPGLLNSNSGHSLMNQAQQGQAQVMNGSLGAAGRGRGAGMPYPAPAMQGATSSVLAETLTQVSPQMAGHAGLNTAQAGGMTKMGMTGTTSPFGQPFSQTGGQQMGATGVNPQLASKQSMVNSLPAFPTDIKNTSVTTVPNMSQLQTSVGIVPTQAIATGPTADPEKRKLIQQQLVLLLHAHKCQRREQANGEVRACSLPHCRTMKNVLNHMTHCQAGKACQVAHCASSRQIISHWKNCTRHDCPVCLPLKNASDKRNQQTILGSPASGIQNTIGSVGAGQQNATSLSNPNPIDPSSMQRAYAALGLPYMNQPQTQLQPQVPGQQPAQPPAHQQMRTLNALGNNPMSIPAGGITTDQQPPNLISESALPTSLGATNPLMNDGSNSGNIGSLSTIPTAAPPSSTGVRKGWHEHVTQDLRSHLVHKLVQAIFPTPDPAALKDRRMENLVAYAKKVEGDMYESANSRDEYYHLLAEKIYKIQKELEEKRRSRLHKQGILGNQPALPASGAQPPVIPPAQSVRPPNGPLPLPVNRMQVSQGMNSFNPMSLGNVQLPQAPMGPRAASPMNHSVQMNSMASVPGMAISPSRMPQPPNMMGTHANNIMAQAPTQNQFLPQNQFPSSSGAMSVNSVGMGQPAAQAGVSQGQVPGAALPNPLNMLAPQASQLPCPPVTQSPLHPTPPPASTAAGMPSLQHPTAPGMTPPQPAAPTQPSTPVSSGQTPTPTPGSVPSAAQTQSTPTVQAAAQAQVTPQPQTPVQPPSVATPQSSQQQPTPVHTQPPGTPLSQAAASIDNRVPTPSSVTSAETSSQQPGPDVPMLEMKTEVQTDDAEPEPTESKGEPRSEMMEEDLQGSSQVKEETDTTEQKSEPMEVEEKKPEVKVEAKEEEENSSNDTASQSTSPSQPRKKIFKPEELRQALMPTLEALYRQDPESLPFRQPVDPQLLGIPDYFDIVKNPMDLSTIKRKLDTGQYQEPWQYVDDVWLMFNNAWLYNRKTSRVYKFCSKLAEVFEQEIDPVMQSLGYCCGRKYEFSPQTLCCYGKQLCTIPRDAAYYSYQNRYHFCEKCFTEIQGENVTLGDDPSQPQTTISKDQFEKKKNDTLDPEPFVDCKECGRKMHQICVLHYDIIWPSGFVCDNCLKKTGRPRKENKFSAKRLQTTRLGNHLEDRVNKFLRRQNHPEAGEVFVRVVASSDKTVEVKPGMKSRFVDSGEMSESFPYRTKALFAFEEIDGVDVCFFGMHVQNTALIAPHQIQGRVYISYLDSIHFFRPRCLRTAVYHEILIGYLEYVKKLGYVTGHIWACPPSEGDDYIFHCHPPDQKIPKPKRLQEWYKKMLDKAFAERIINDYKDIFKQANEDRLTSAKELPYFEGDFWPNVLEESIKELEQEEEERKKEESTAASETPEGSQGDSKNAKKKNNKKTNKNKSSISRANKKKPSMPNVSNDLSQKLYATMEKHKEVFFVIHLHAGPVISTQPPIVDPDPLLSCDLMDGRDAFLTLARDKHWEFSSLRRSKWSTLCMLVELHTQGQDRFVYTCNECKHHVETRWHCTVCEDYDLCINCYNTKSHTHKMVKWGLGLDDEGSSQGEPQSKSPQESRRLSIQRCIQSLVHACQCRNANCSLPSCQKMKRVVQHTKGCKRKTNGGCPVCKQLIALCCYHAKHCQENKCPVPFCLNIKHKLRQQQIQHRLQQAQLMRRRMATMNTRNVPQQSLPSPTSAPPGTPTQQPSTPQTPQPPAQPQPSPVNMSPAGFPNVARTQPPTIVSAGKPTNQVPAPPPPAQPPPAAVEAARQIEREAQQQQHLYRANINNGMPPGRAGMGTPGSQMTPVGLNVPRPNQVSGPVMSSMPPGQWQQAPIPQQQPMPGMPRPVMSMQAQAAVAGPRMPNVQPPRSISPSALQDLLRTLKSPSSPQQQQQVLNILKSNPQLMAAFIKQRTAKYVANQPGMQPQPGLQSQPGMQPQPGMHQQPSLQNLNAMQAGVPRPGVPPPQPAMGGLNPQGQALNIMNPGHNPNMTNMNPQYREMVRRQLLQHQQQQQQQQQQQQQQQNSASLAGGMAGHSQFQQPQGPGGYAPAMQQQRMQQHLPIQGSSMGQMAAPMGQLGQMGQPGLGADSTPNIQQALQQRILQQQQMKQQIGSPGQPNPMSPQQHMLSGQPQASHLPGQQIATSLSNQVRSPAPVQSPRPQSQPPHSSPSPRIQPQPSPHHVSPQTGSPHPGLAVTMASSMDQGHLGNPEQSAMLPQLNTPNRSALSSELSLVGDTTGDTLEKFVEGL

00000000000000000000000000000000000000000000000000000000000000000000000000000000000000000000000000000000000000000000000000000000000000000000000000000000000000000000000000000000000000000000000000000000000000000000000000000000000000000000000000000000000000000000000000000000000000000000000000000000000000000000000000000000000000000000000000000000000000000000000000000000000000000000000000000000000000000000000000000000000000000000000000000000000000000000000000000000000000000000000000000000000000000000000000000000000000000000000000000000000000000000000000000000000000000000000000000000000000000000000000000000000000000000000000000000000000000000000000000000000000000000000000000000000000000000000000000000000000000000000000000000000000000000000000000000000000000000000000000000000000000000000000000000000000000000000000000000000000000000000000000000000000000000000000000000000000000000000000000000000000000000000000000000000000000000000000000000000000000000000000000000000000000000000000000000000000000000000000000000000000000000000000000000000000000000000000000000000000000000000000000000000000000000000000000000000000000000000000000000000000000000000000000000000000000000000000000000000000000000000000000000000000000000000000000000000000000000000000000000000000000000000000000000000000000000000000000000000000000000000000000000000000000000000000000000000000000000000000000000000000000000000000000000000000000000000000000000000000000000000000000000000000000000000000000000000000000000000000000000000000000000000000000000000000000000000000000000000000000000000000000000000000000000000000000000000000000000000000000000000000000000000000000000000000000000000000000000000000000000000000000000000000000000000000000000000000000000000000000000000000000000000000000000000000000000000000000000000000000000000000000000000000000000000000000000000000000000000000000000000000000000000000000000000000000000000000000000000000000000000000000000000000000000000000000000000000000000000000000000000000000000000000000000000000000000000000000000000000000000000000000000000000000011111111111111111111111111111111111111111111111111111111111111111111111111111111111111111111110000000000000000000000000000000000000000000000000000000000000000000000000000000000000000000000000000000000000000000000000000000000000000000000000000000000000000000000000000000000000000000000000000000000000000000000000000000000000000000000000000000000000000000000000000000000000000000000000

00000000000000000000000000000000000000000000000000000000000000000000000000000000000000000000000000000000000000000000000000000000000000000000000000000000000000000000000000000000000000000000000000000000000000000000000000000000000000000000000000000000000000000000000000000000000000000000000000000000000000000000000000000000000000000000000000000000000000000000000000000000000000000000000000000000000000000000000000000000000000000000000000000000000000000000000000000000000000000000000000000000000000000000000000000000000000000000000000000000000000000000000000000000000000000000000000000000000000000000000000000000000000000000000000000000000000000000000000000000000000000000000000000000000000000000000000000000000000000000000000000000000000000000000000000000000000000000000000000000000000000000000000000000000000000000000000000000000000000000000000000000000000000000000000000000000000000000000000000000000000000000000000000000000000000000000000000000000000000000000000000000000000000000000000000000000000000000000000000000000000000000000000000000000000000000000000000000000000000000000000000000000000000000000000000000000000000000000000000000000000000000000000000000000000000000000000000000000000000000000000000000000000000000000000000000000000000000000000000000000000000000000000000000000000000000000000000000000000000000000000000000000000000000000000000000000000000000000000000000000000000000000000000000000000000000000000000000000000000000000000000000000000000000000000000000000000000000000000000000000000000000000000000000000000000000000000000000000000000000000000000000000000000000000000000000000000000000000000000000000000000000000000000000000000000000000000000000000000000000000000000000000000000000000000000000000000000000000000000000000000000000000000000000000000000000000000000000000000000000000000000000000000000000000000000000000000000000000000000000000000000000000000000000000000000000000000000000000000000000000000000000000000000000000000000000000000000000000000000000000000000000000000000000000000000000000000000000000000000000000000000000000000000000000000000000000000000000000000000000000000000000000000000000000000000000000000000000000000000000000000000000000000000000000000000000000000000000000000000000000000000000000000000000000000000000000000000000000000000000000000000000000000000000000000000000000000000000000000000000000000000000000000000000000000000000000000000000000000000000000000000000000000000000000000000000000000000

00000000000000000000000000000000000000000000000000000000000000000000000000000000000000000000000000000000000000000000000000000000000000000000000000000000000000000000000000000000000000000000000000000000000000000000000000000000000000000000000000000000000000000000000000000000000000000000000000000000000000000000000000000000000000000000000000000000000000000000000000000000000000000000000000000000000000000000000000000000000000000000000000000000000000000000000000000000000000000000000000000000000000000000000000000000000000000000000000000000000000000000000000000000000000000000000000000000000000000000000000000000000000000000000000000000000000000000000000000000000000000000000000000000000000000000000000000000000000000000000000000000000000000000000000000000000000000000000000000000000000000000000000000000000000000000000000000000000000000000000000000000000000000000000000000000000000000000000000000000000000000000000000000000000000000000000000000000000000000000000000000000000000000000000000000000000000000000000000000000000000000000000000000000000000000000000000000000000000000000000000000000000000000000000000000000000000000000000000000000000000000000000000000000000000000000000000000000000000000000000000000000000000000000000000000000000000000000000000000000000000000000000000000000000000000000000000000000000000000000000000000000000000000000000000000000000000000000000000000000000000000000000000000000000000000000000000000000000000000000000000000000000000000000000000000000000000000000000000000000000000000000000000000000000000000000000000000000000000000000000000000000000000000000000000000000000000000000000000000000000000000000000000000000000000000000000000000000000000000000000000000000000000000000000000000000000000000000000000000000000000000000000000000000000000000000000000000000000000000000000000000000000000000000000000000000000000000000000000000000000000000000000000000000000000000000000000000000000000000000000000000000000000000000000000000000000000000000000000000000000000000000000000000000000000000000000000000000000000000000000000000000000000000000000000000000000000000000000000000000000000000000000000000000000000000000000000000000000000000000000000000000000000000000000000000000000000000000000000000000000000000000000000000000000000000000000000000000000000000000000000000000000000000000000000000000000000000000000000000000000000000000000000000000000000000000000000000000000000000000000000000000000000000000000000000000000000000

00000000000000000000000000000000000000000000000000000000000000000000000000000000000000000000000000000000000000000000000000000000000000000000000000000000000000000000000000000000000000000000000000000000000000000000000000000000000000000000000000000000000000000000000000000000000000000000000000000000000000000000000000000000000000000000000000000000000000000000000000000000000000000000000000000000000000000000000000000000000000000000000000000000000000000000000000000000000000000000000000000000000000000000000000000000000000000000000000000000000000000000000000000000000000000000000000000000000000000000000000000000000000000000000000000000000000000000000000000000000000000000000000000000000000000000000000000000000000000000000000000000000000000000000000000000000000000000000000000000000000000000000000000000000000000000000000000000000000000000000000000000000000000000000000000000000000000000000000000000000000000000000000000000000000000000000000000000000000000000000000000000000000000000000000000000000000000000000000000000000000000000000000000000000000000000000000000000000000000000000000000000000000000000000000000000000000000000000000000000000000000000000000000000000000000000000000000000000000000000000000000000000000000000000000000000000000000000000000000000000000000000000000000000000000000000000000000000000000000000000000000000000000000000000000000000000000000000000000000000000000000000000000000000000000000000000000000000000000000000000000000000000000000000000000000000000000000000000000000000000000000000000000000000000000000000000000000000000000000000000000000000000000000000000000000000000000000000000000000000000000000000000000000000000000000000000000000000000000000000000000000000000000000000000000000000000000000000000000000000000000000000000000000000000000000000000000000000000000000000000000000000000000000000000000000000000000000000000000000000000000000000000000000000000000000000000000000000000000000000000000000000000000000000000000000000000000000000000000000000000000000000000000000000000000000000000000000000000000000000000000000000000000000000000000000000000000000000000000000000000000000000000000000000000000000000000000000000000000000000000000000000000000000000000000000000000000000000000000000000000000000000000000000000000000000000000000000000000000000000000000000000000000000000000000000000000000000000000000000000000000000000000000000000000000000000000000000000000000000000000000000000000000000000000000000000000000000

00000000000000000000000000000000000000000000000000000000000000000000000000000000000000000000000000000000000000000000000000000000000000000000000000000000000000000000000000000000000000000000000000000000000000000000000000000000000000000000000000000000000000000000000000000000000000000000000000000000000000000000000000000000000000000000000000000000000000000000000000000000000000000000000000000000000000000000000000000000000000000000000000000000000000000000000000000000000000000000000000000000000000000000000000000000000000000000000000000000000000000000000000000000000000000000000000000000000000000000000000000000000000000000000000000000000000000000000000000000000000000000000000000000000000000000000000000000000000000000000000000000000000000000000000000000000000000000000000000000000000000000000000000000000000000000000000000000000000000000000000000000000000000000000000000000000000000000000000000000000000000000000000000000000000000000000000000000000000000000000000000000000000000000000000000000000000000000000000000000000000000000000000000000000000000000000000000000000000000000000000000000000000000000000000000000000000000000000000000000000000000000000000000000000000000000000000000000000000000000000000000000000000000000000000000000000000000000000000000000000000000000000000000000000000000000000000000000000000000000000000000000000000000000000000000000000000000000000000000000000000000000000000000000000000000000000000000000000000000000000000000000000000000000000000000000000000000000000000000000000000000000000000000000000000000000000000000000000000000000000000000000000000000000000000000000000000000000000000000000000000000000000000000000000000000000000000000000000000000000000000000000000000000000000000000000000000000000000000000000000000000000000000000000000000000000000000000000000000000000000000000000000000000000000000000000000000000000000000000000000000000000000000000000000000000000000000000000000000000000000000000000000000000000000000000000000000000000000000000000000000000000000000000000000000000000000000000000000000000000000000000000000000000000000000000000000000000000000000000000000000000000000000000000000000000000000000000000000000000000000000000000000000000000000000000000000000000000000000000000000000000000000000000000000000000000000000000000000000000000000000000000000000000000000000000000000000000000000000000000000000000000000000000000000000000000000000000000000000000000000000000000000000000000000000000000000000000

>DP00897

MLDKLGENLNKALNKLKAAAFVDKKLIKEVIKDIQRALIQADVNVKLVLKMSKEIERRALEEKTPKGLSKKEHIIKIVYEELVKLLGEEAKKLELNPKKQNVILLVGIQGSGKTTTAAKLARYIQKRGLKPALIAADTYRPAAYEQLKQLAEKIHVPIYGDETRTKSPVDIVKEGMEKFKKADVLIIDTAGRHKEEKGLLEEMKQIKEITNPDEIILVIDGTIGQQAGIQAKAFKEAVGEIGSIIVTKLDGSAKGGGALSAVAETKAPIKFIGIGEGIDDLEPFDPKKFISRLLGMGDLESLLEKAEDMVDEKTEESIDAIMRGKFTLNELMTQLEAIENMGSMKKILSMIPGFGGAMPKELSHLTEAKIKKYKVIISSMTKEERENPKIIKASRIRRIARGSGTTENDVREVLRYYETTKNAIDKLRKGKMLRIGGPLGQIMRQLMFKEG

0000000000000000000000000000000000000000000000000000000000000000000000000000000000000000000000000000000000000000000000000000000000000000000000000000000000000000000000000000000000000000000000000000000000000000000000000000000000000000000000000000000000000000000000000000000000000000000000000000000000000011111111111111110000000000000000000000000011111111111111111111000000000000000000000000000000000000000000000000000000000000000001111111111111111111111

0000000000000000000000000000000000000000000000000000000000000000000000000000000000000000000000000000000000000000000000000000000000000000000000000000000000000000000000000000000000000000000000000000000000000000000000000000000000000000000000000000000000000000000000000000000000000000000000000000000000000000000000000000000000000000000000000000000000000000000000000000000000000000000000000000000000000000000000000000000000000000000000000000000000000000000

0000000000000000000000000000000000000000000000000000000000000000000000000000000000000000000000000000000000000000000000000000000000000000000000000000000000000000000000000000000000000000000000000000000000000000000000000000000000000000000000000000000000000000000000000000000000000000000000000000000000000000000000000000000000000000000000000000000000000000000000000000000000000000000000000000000000000000000000000000000000000000000000000000000000000000000

0000000000000000000000000000000000000000000000000000000000000000000000000000000000000000000000000000000000000000000000000000000000000000000000000000000000000000000000000000000000000000000000000000000000000000000000000000000000000000000000000000000000000000000000000000000000000000000000000000000000000000000000000000000000000000000000000000000000000000000000000000000000000000000000000000000000000000000000000000000000000000000000000000000000000000000

0000000000000000000000000000000000000000000000000000000000000000000000000000000000000000000000000000000000000000000000000000000000000000000000000000000000000000000000000000000000000000000000000000000000000000000000000000000000000000000000000000000000000000000000000000000000000000000000000000000000000000000000000000000000000000000000000000000000000000000000000000000000000000000000000000000000000000000000000000000000000000000000000000000000000000000

>DP01249

MEGISIYTSDNYTEEMGSGDYDSMKEPCFREENANFNKIFLPTIYSIIFLTGIVGNGLVILVMGYQKKLRSMTDKYRLHLSVADLLFVITLPFWAVDAVANWYFGNFLCKAVHVIYTVNLYSSVLILAFISLDRYLAIVHATNSQRPRKLLAEKVVYVGVWIPALLLTIPDFIFANVSEADDRYICDRFYPNDLWVVVFQFQHIMVGLILPGIVILSCYCIIISKLSHSKGHQKRKALKTTVILILAFFACWLPYYIGISIDSFILLEIIKQGCEFENTVHKWISITEALAFFHCCLNPILYAFLGAKFKTSAQHALTSVSRGSSLKILSKGKRGGHSSVSTESESSSFHSS

1111111111111111111111111100000000000000000000000000000000000000000000000000000000000000000000000000000000000000000000000000000000000000000000000000000000000000000000000000000000000000000000000000000000000000000000000000000000000000000000000000000000000000000000000000000000000000000000000000000000000000000000000000000000000000000000000000000000000000

0000000000000000000000000000000000000000000000000000000000000000000000000000000000000000000000000000000000000000000000000000000000000000000000000000000000000000000000000000000000000000000000000000000000000000000000000000000000000000000000000000000000000000000000000000000000000000000000000000000000000000000000000000000000000000000000000000000000000000

0000000000000000000000000000000000000000000000000000000000000000000000000000000000000000000000000000000000000000000000000000000000000000000000000000000000000000000000000000000000000000000000000000000000000000000000000000000000000000000000000000000000000000000000000000000000000000000000000000000000000000000000000000000000000000000000000000000000000000

0000000000000000000000000000000000000000000000000000000000000000000000000000000000000000000000000000000000000000000000000000000000000000000000000000000000000000000000000000000000000000000000000000000000000000000000000000000000000000000000000000000000000000000000000000000000000000000000000000000000000000000000000000000000000000000000000000000000000000

0000000000000000000000000000000000000000000000000000000000000000000000000000000000000000000000000000000000000000000000000000000000000000000000000000000000000000000000000000000000000000000000000000000000000000000000000000000000000000000000000000000000000000000000000000000000000000000000000000000000000000000000000000000000000000000000000000000000000000

>DP00141

MENYFQAEAYNLDKVLDEFEQNEDETVSSTLLDTKWNKILDPPSHRLSFNPTLASVNESAVSNESQPQLKVFSLAHSAPLTTEEEDHCANGQDCNLNPEIATMWIDENAVAEDQLIKRNYSWDDQCSAVEVGEKKCGNLACLPDEKNVLVVAVMHNCDKRTLQNDLQDCNNYNSQSLMDAFSCSLDNENRQTDQFSFSINESTEKDMNSEKQMDPLNRPKTEGRSVNHLCPTSSDSLASVCSPSQLKDDGSIGRDPSMSAITSLTVDSVISSQGTDGCPAVKKQENYIPDEDLTGKISSPRTDLGSPNSFSHMSEGILMKKEPAEESTTEESLRSGLPLLLKPDMPNGSGRNNDCERCSDCLVPNEVRADENEGYEHEETLGTTEFLNMTEHFSESQDMTNWKLTKLNEMNDSQVNEEKEKFLQISQPEDTNGDSGGQCVGLADAGLDLKGTCISESEECDFSTVIDTPAANYLSNGCDSYGMQDPGVSFVPKTLPSKEDSVTEEKEIEESKSECYSNIYEQRGNEATEGSGLLLNSTGDLMKKNYLHNFCSQVPSVLGQSSPKVVASLPSISVPFGGARPKQPSNLKLQIPKPLSDHLQNDFPANSGNNTKNKNDILGKAKLGENSATNVCSPSLGNISNVDTNGEHLESYEAEISTRPCLALAPDSPDNDLRAGQFGISARKPFTTLGEVAPVWVPDSQAPNCMKCEARFTFTKRRHHCRACGKVFCASCCSLKCKLLYMDRKEARVCVICHSVLMNAQAWENMMSASSQSPNPNNPAEYCSTIPPLQQAQASGALSSPPPTVMVPVGVLKHPGAEVAQPREQRRVWFADGILPNGEVADAAKLTMNGTSSAGTLAVSHDPVKPVTTSPLPAETDICLFSGSITQVGSPVGSAMNLIPEDGLPPILISTGVKGDYAVEEKPSQISVMQQLEDGGPDPLVFVLNANLLSMVKIVNYVNRKCWCFTTKGMHAVGQSEIVILLQCLPDEKCLPKDIFNHFVQLYRDALAGNVVSNLGHSFFSQSFLGSKEHGGFLYVTSTYQSLQDLVLPTPPYLFGILIQKWETPWAKVFPIRLMLRLGAEYRLYPCPLFSVRFRKPLFGETGHTIMNLLADFRNYQYTLPVVQGLVVDMEVRKTSIKIPSNRYNEMMKAMNKSNEHVLAGGACFNEKADSHLVCVQNDDGNYQTQAISIHNQPRKVTGASFFVFSGALKSSSGYLAKSSIVEDGVMVQITAENMDSLRQALREMKDFTITCGKADAEEPQEHIHIQWVDDDKNVSKGVVSPIDGKSMETITNVKIFHGSEYKANGKVIRWTEVFFLENDDQHNCLSDPADHSRLTEHVAKAFCLALCPHLKLLKEDGMTKLGLRVTLDSDQVGYQAGSNGQPLPSQYMNDLDSALVPVIHGGACQLSEGPVVMELIFYILENIV

000000000000000000000000000000000000000000000000000000000000000000000000000000000000000000000000000000000000000000000000000000000000000000000000000000000000000000000000000000000000000000000000000000000000000000000000000000000000000000000000000000000000000000000000000000000000000000000000000000000000000000000000000000000000000000000000000000000000000000000000000000000000000000000000000000000000000000000000000000000000000000000000000000000000000000000000000000000000000000000000000000000000000000000000000000000000000000000000000000000000000000000000000000000000000000000000000000000000000000000000000000000000000000000000000000000000000000000000000000000000001111111111111111111111111111111111111111111111111111111111111111111111111111111111111111100000000000000000000000000000000000000000000000000000000000000000000000000000000000000000000000000000000000000000000000000000000000000000000000000000000000000000000000000000000000000000000000000000000000000000000000000000000000000000000000000000000000000000000000000000000000000000000000000000000000000000000000000000000000000000000000000000000000000000000000000000000000000000000000000000000000000000000000000000000000000000000000000000000000000000000000000000000000000000000000000000000000000000000000000000000000000000000000000000000000000000000000000000000000000000000000000000000000000000000000000000000000000000000000000000000000000000000000000000000000000000000000000

000000000000000000000000000000000000000000000000000000000000000000000000000000000000000000000000000000000000000000000000000000000000000000000000000000000000000000000000000000000000000000000000000000000000000000000000000000000000000000000000000000000000000000000000000000000000000000000000000000000000000000000000000000000000000000000000000000000000000000000000000000000000000000000000000000000000000000000000000000000000000000000000000000000000000000000000000000000000000000000000000000000000000000000000000000000000000000000000000000000000000000000000000000000000000000000000000000000000000000000000000000000000000000000000000000000000000000000000000000000000000000000000000000000000000000000000000000000000000000000000000000000000000000000000000000000000000000000000000000000000000000000000000000000000000000000000000000000000000000000000000000000000000000000000000000000000000000000000000000000000000000000000000000000000000000000000000000000000000000000000000000000000000000000000000000000000000000000000000000000000000000000000000000000000000000000000000000000000000000000000000000000000000000000000000000000000000000000000000000000000000000000000000000000000000000000000000000000000000000000000000000000000000000000000000000000000000000000000000000000000000000000000000000000000000000000000000000000000000000000000000000000000000000000000000000000000000000000000000000000000000000000000000000000000000000000000000000000

000000000000000000000000000000000000000000000000000000000000000000000000000000000000000000000000000000000000000000000000000000000000000000000000000000000000000000000000000000000000000000000000000000000000000000000000000000000000000000000000000000000000000000000000000000000000000000000000000000000000000000000000000000000000000000000000000000000000000000000000000000000000000000000000000000000000000000000000000000000000000000000000000000000000000000000000000000000000000000000000000000000000000000000000000000000000000000000000000000000000000000000000000000000000000000000000000000000000000000000000000000000000000000000000000000000000000000000000000000000000000000000000000000000000000000000000000000000000000000000000000000000000000000000000000000000000000000000000000000000000000000000000000000000000000000000000000000000000000000000000000000000000000000000000000000000000000000000000000000000000000000000000000000000000000000000000000000000000000000000000000000000000000000000000000000000000000000000000000000000000000000000000000000000000000000000000000000000000000000000000000000000000000000000000000000000000000000000000000000000000000000000000000000000000000000000000000000000000000000000000000000000000000000000000000000000000000000000000000000000000000000000000000000000000000000000000000000000000000000000000000000000000000000000000000000000000000000000000000000000000000000000000000000000000000000000000000000000

000000000000000000000000000000000000000000000000000000000000000000000000000000000000000000000000000000000000000000000000000000000000000000000000000000000000000000000000000000000000000000000000000000000000000000000000000000000000000000000000000000000000000000000000000000000000000000000000000000000000000000000000000000000000000000000000000000000000000000000000000000000000000000000000000000000000000000000000000000000000000000000000000000000000000000000000000000000000000000000000000000000000000000000000000000000000000000000000000000000000000000000000000000000000000000000000000000000000000000000000000000000000000000000000000000000000000000000000000000000000000000000000000000000000000000000000000000000000000000000000000000000000000000000000000000000000000000000000000000000000000000000000000000000000000000000000000000000000000000000000000000000000000000000000000000000000000000000000000000000000000000000000000000000000000000000000000000000000000000000000000000000000000000000000000000000000000000000000000000000000000000000000000000000000000000000000000000000000000000000000000000000000000000000000000000000000000000000000000000000000000000000000000000000000000000000000000000000000000000000000000000000000000000000000000000000000000000000000000000000000000000000000000000000000000000000000000000000000000000000000000000000000000000000000000000000000000000000000000000000000000000000000000000000000000000000000000000000

000000000000000000000000000000000000000000000000000000000000000000000000000000000000000000000000000000000000000000000000000000000000000000000000000000000000000000000000000000000000000000000000000000000000000000000000000000000000000000000000000000000000000000000000000000000000000000000000000000000000000000000000000000000000000000000000000000000000000000000000000000000000000000000000000000000000000000000000000000000000000000000000000000000000000000000000000000000000000000000000000000000000000000000000000000000000000000000000000000000000000000000000000000000000000000000000000000000000000000000000000000000000000000000000000000000000000000000000000000000000000000000000000000000000000000000000000000000000000000000000000000000000000000000000000000000000000000000000000000000000000000000000000000000000000000000000000000000000000000000000000000000000000000000000000000000000000000000000000000000000000000000000000000000000000000000000000000000000000000000000000000000000000000000000000000000000000000000000000000000000000000000000000000000000000000000000000000000000000000000000000000000000000000000000000000000000000000000000000000000000000000000000000000000000000000000000000000000000000000000000000000000000000000000000000000000000000000000000000000000000000000000000000000000000000000000000000000000000000000000000000000000000000000000000000000000000000000000000000000000000000000000000000000000000000000000000000000000

>DP01079

MSETNGGNAARENSEVKQTAVENPIDKLDGTPKRPREKDQDEQAEETSDKSEAPNKNDEEKKEEGKKDQEPSHKKIKVDDGKTVESGIVEDDKKEDKFVFGAASKFGTGFGVAKKDTKDGDATTSTESLPASDSKTKKPFAFGSGLSFGSGFNILKNKTENNSESEKKATDVDKDKVHSGSEQLANASEDTKDKPKPLKLQKQEVKSGEESEECIYQVNAKLYQLSNIKEGWKERGVGIIKINKSKDDVEKTRIVMRSRGILKVILNIQLVKGFTVQKGFTGSLQSEKFIRLLAVDDNGDPAQYAIKTGKKETTDELYNIIVKSVPK

111111111111111111111111111111111111111111111111111111111111111111111111111111111111111111111111111111111111111111111111111111111111111111111111111111111111111111111111111111111111111111111111111111111110000000000000000000000000000000000000000000000000000000000000000000000000000000000000000000000000000000000000000000000000000

000000000000000000000000000000000000000000000000000000000000000000000000000000000000000000000000000000000000000000000000000000000000000000000000000000000000000000000000000000000000000000000000000000000000000000000000000000000000000000000000000000000000000000000000000000000000000000000000000000000000000000000000000000000000000

000000000000000000000000000000000000000000000000000000000000000000000000000000000000000000000000000000000000000000000000000000000000000000000000000000000000000000000000000000000000000000000000000000000000000000000000000000000000000000000000000000000000000000000000000000000000000000000000000000000000000000000000000000000000000

000000000000000000000000000000000000000000000000000000000000000000000000000000000000000000000000000000000000000000000000000000000000000000000000000000000000000000000000000000000000000000000000000000000000000000000000000000000000000000000000000000000000000000000000000000000000000000000000000000000000000000000000000000000000000

000000000000000000000000000000000000000000000000000000000000000000000000000000000000000000000000000000000000000000000000000000000000000000000000000000000000000000000000000000000000000000000000000000000000000000000000000000000000000000000000000000000000000000000000000000000000000000000000000000000000000000000000000000000000000

>DP02839

MWIIEAEGDILKGKSRILFPGTYIVGRNVSDDSSHIQVISKSISKRHARFTILTPSEKDYFTGGPCEFEVKDLDTKFGTKVNEKVVGQNGDSYKEKDLKIQLGKCPFTINAYWRSMCIQFDNPEMLSQWASNLNLLGIPTGLRDSDATTHFVMNRQAGSSITVGTMYAFLKKTVIIDDSYLQYLSTVKESVIEDASLMPDALECFKNIIKNNDQFPSSPEDCINSLEGFSCAMLNTSSESHHLLELLGLRISTFMSLGDIDKELISKTDFVVLNNAVYDSEKISFPEGIFCLTIEQLWKIIIERNSRELISKEIERLKYATASNSTPQKIIQPQRHIQKNIVDDLFSVKKPLPCSPKSKRVKTLENLSIMDFVQPKQMFGKEPEGYLSNQSNNGSAQNKKSGDNSEKTKNSLKSSSKKSANTGSGQGKTKVEYVSYNSVDKGNSSPFKPLELNVVGEKKANAEVDSLPSENVQESEDDKAFEENRRLRNLGSVEYIRIMSSEKSNANSRHTSKYYSGRKNFKKFQKKASQKAPLQAFLSLSEHKKTEVFDQDDTDLEPVPRLMSKVESIPAGASSDKSGKSSISKKSSNSFKELSPKTNNDEDDEFNDLKFHF

0000000000000000000000000000000000000000000000000000000000000000000000000000000000000000000000000000000000000000000000000000000000000000000000000000000000000000000000000000000000000000000000000000000000000000000000000000000000000000000000000000000000000000000000000000000000000000000000000000000000000000000000000000000000000000011111111111111111111111111111111111111111111111111111111111111111111111111111111111111111111111111111111111111111111111111111111111111111111111111111111111111111111111111111111111111111111111111111111111111111111111111111111000000000000000000000000000000000000000000000000000000000000

0000000000000000000000000000000000000000000000000000000000000000000000000000000000000000000000000000000000000000000000000000000000000000000000000000000000000000000000000000000000000000000000000000000000000000000000000000000000000000000000000000000000000000000000000000000000000000000000000000000000000000000000000000000000000000000000000000000000000000000000000000000000000000000000000000000000000000000000000000000000000000000000000000000000000000000000000000000000000000000000000000000000000000000000000000000000000000000000000000000000000000000000000000000000000000000000000000000000000000000000000000000000000

0000000000000000000000000000000000000000000000000000000000000000000000000000000000000000000000000000000000000000000000000000000000000000000000000000000000000000000000000000000000000000000000000000000000000000000000000000000000000000000000000000000000000000000000000000000000000000000000000000000000000000000000000000000000000000000000000000000000000000000000000000000000000000000000000000000000000000000000000000000000000000000000000000000000000000000000000000000000000000000000000000000000000000000000000000000000000000000000000000000000000000000000000000000000000000000000000000000000000000000000000000000000000

0000000000000000000000000000000000000000000000000000000000000000000000000000000000000000000000000000000000000000000000000000000000000000000000000000000000000000000000000000000000000000000000000000000000000000000000000000000000000000000000000000000000000000000000000000000000000000000000000000000000000000000000000000000000000000000000000000000000000000000000000000000000000000000000000000000000000000000000000000000000000000000000000000000000000000000000000000000000000000000000000000000000000000000000000000000000000000000000000000000000000000000000000000000000000000000000000000000000000000000000000000000000000

0000000000000000000000000000000000000000000000000000000000000000000000000000000000000000000000000000000000000000000000000000000000000000000000000000000000000000000000000000000000000000000000000000000000000000000000000000000000000000000000000000000000000000000000000000000000000000000000000000000000000000000000000000000000000000000000000000000000000000000000000000000000000000000000000000000000000000000000000000000000000000000000000000000000000000000000000000000000000000000000000000000000000000000000000000000000000000000000000000000000000000000000000000000000000000000000000000000000000000000000000000000000000

>DP00173

MEVTGDAGVPESGEIRTLKPCLLRRNYSREQHGVAASCLEDLRSKACDILAIDKSLTPVTLVLAEDGTIVDDDDYFLCLPSNTKFVALASNEKWAYNNSDGGTAWISQESFDVDETDSGAGLKWKNVARQLKEDLSSIILLSEEDLQMLVDAPCSDLAQELRQSCATVQRLQHTLQQVLDQREEVRQSKQLLQLYLQALEKEGSLLSKQEESKAAFGEEVDAVDTGISRETSSDVALASHILTALREKQAPELSLSSQDLELVTKEDPKALAVALNWDIKKTETVQEACERELALRLQQTQSLHSLRSISASKASPPGDLQNPKRARQDPT

1111111111111111111111111111111111111111111111111111111111111111111111111111111111111111111111111111111111111111111100000000000000000000000000000000000000000000000000000000000000000000000000000000000000000000000000000000000000000000000000000000000000000000000000000000000000000000000000000000000000000000000000000000000000000000000

0000000000000000000000000000000000000000000000000000000000000000000000000000000000000000000000000000000000000000000000000000000000000000000000000000000000000000000000000000000000000000000000000000000000000000000000000000000000000000000000000000000000000000000000000000000000000000000000000000000000000000000000000000000000000000000

0000000000000000000000000000000000000000000000000000000000000000000000000000000000000000000000000000000000000000000000000000000000000000000000000000000000000000000000000000000000000000000000000000000000000000000000000000000000000000000000000000000000000000000000000000000000000000000000000000000000000000000000000000000000000000000

0000000000000000000000000000000000000000000000000000000000000000000000000000000000000000000000000000000000000000000000000000000000000000000000000000000000000000000000000000000000000000000000000000000000000000000000000000000000000000000000000000000000000000000000000000000000000000000000000000000000000000000000000000000000000000000

0000000000000000000000000000000000000000000000000000000000000000000000000000000000000000000000000000000000000000000000000000000000000000000000000000000000000000000000000000000000000000000000000000000000000000000000000000000000000000000000000000000000000000000000000000000000000000000000000000000000000000000000000000000000000000000

>DP00628

MRAAPLLLARAASLSLGFLFLLFFWLDRSVLAKELKFVTLVFRHGDRSPIDTFPTDPIKESSWPQGFGQLTQLGMEQHYELGEYIRKRYRKFLNESYKHEQVYIRSTDVDRTLMSAMTNLAALFPPEGVSIWNPILLWQPIPVHTVPLSEDQLLYLPFRNCPRFQELESETLKSEEFQKRLHPYKDFIATLGKLSGLHGQDLFGIWSKVYDPLYCESVHNFTLPSWATEDTMTKLRELSELSLLSLYGIHKQKEKSRLQGGVLVNEILNHMKRATQIPSYKKLIMYSAHDTTVSGLQMALDVYNGLLPPYASCHLTELYFEKGEYFVEMYYRNETQHEPYPLMLPGCSPSCPLERFAELVGPVIPQDWSTECMTTNSHQGTEDSTD

00000000000000000000000000000000000000000000000000000000000000000000000000000000000000000000000000000000000000000000000000000000000000000000000000000000000000000000000000000000000000000000000000000000000000000000000000000000000000000000000000000001111111111111111111111111111111111111110000000000000000000000000000000000000000000000000000000000000000000000000000000000000000000000000000

00000000000000000000000000000000000000000000000000000000000000000000000000000000000000000000000000000000000000000000000000000000000000000000000000000000000000000000000000000000000000000000000000000000000000000000000000000000000000000000000000000000000000000000000000000000000000000000000000000000000000000000000000000000000000000000000000000000000000000000000000000000000000000000000000

00000000000000000000000000000000000000000000000000000000000000000000000000000000000000000000000000000000000000000000000000000000000000000000000000000000000000000000000000000000000000000000000000000000000000000000000000000000000000000000000000000000000000000000000000000000000000000000000000000000000000000000000000000000000000000000000000000000000000000000000000000000000000000000000000

00000000000000000000000000000000000000000000000000000000000000000000000000000000000000000000000000000000000000000000000000000000000000000000000000000000000000000000000000000000000000000000000000000000000000000000000000000000000000000000000000000000000000000000000000000000000000000000000000000000000000000000000000000000000000000000000000000000000000000000000000000000000000000000000000

00000000000000000000000000000000000000000000000000000000000000000000000000000000000000000000000000000000000000000000000000000000000000000000000000000000000000000000000000000000000000000000000000000000000000000000000000000000000000000000000000000000000000000000000000000000000000000000000000000000000000000000000000000000000000000000000000000000000000000000000000000000000000000000000000

>DP00241

MSEETVPEAASPPPPQGQPYFDRFSEDDPEYMRLRNRAADLRQDFNLMEQKKRVTMILQSPSFREELEGLIQEQMKKGNNSSNIWALRQIADFMASTSHAVFPTSSMNVSMMTPINDLHTADSLNLAKGERLMRCKISSVYRLLDLYGWAQLSDTYVTLRVSKEQDHFLISPKGVSCSEVTASSLIKVNILGEVVEKGSSCFPVDTTGFCLHSAIYAARPDVRCIIHLHTPATAAVSAMKWGLLPVSHNALLVGDMAYYDFNGEMEQEADRINLQKCLGPTCKILVLRNHGVVALGDTVEEAFYKIFHLQAACEIQVSALSSAGGVENLILLEQEKHRPHEVGSVQWAGSTFGPMQKSRLGEHEFEALMRMLDNLGYRTGYTYRHPFVQEKTKHKSEVEIPATVTAFVFEEDGAPVPALRQHAQKQQKEKTRWLNTPNTYLRVNVADEVQRSMGSPRPKTTWMKADEVEKSSSGMPIRIENPNQFVPLYTDPQEVLEMRNKIREQNRQDVKSAGPQSQLLASVIAEKSRSPSTESQLMSKGDEDTKDDSEETVPNPFSQLTDQELEEYKKEVERKKLELDGEKETAPEEPGSPAKSAPASPVQSPAKEAETKSPLVSPSKSLEEGTKKTETSKAATTEPETTQPEGVVVNGREEEQTAEEILSKGLSQMTTSADTDVDTSKDKTESVTSGPMSPEGSPSKSPSKKKKKFRTPSFLKKSKKKEKVES

000000000000000000000000000000000000000000000000000000000000000000000000000000000000000000000000000000000000000000000000000000000000000000000000000000000000000000000000000000000000000000000000000000000000000000000000000000000000000000000000000000000000000000000000000000000000000000000000000000000000000000000000000000000000000000000000000000000000000000000000000000000000000000000000000000000000000000000000111111111111111111111111111111111111111111111111111111111111111111111111111111111111111111111111111111111111111111111111111111111111111111111111111111111111111111111111111111111111111111111111111111111111111111111111111111111111111111111111111111111111111111111111111111111111111111111111111111111111111111111111111111

000000000000000000000000000000000000000000000000000000000000000000000000000000000000000000000000000000000000000000000000000000000000000000000000000000000000000000000000000000000000000000000000000000000000000000000000000000000000000000000000000000000000000000000000000000000000000000000000000000000000000000000000000000000000000000000000000000000000000000000000000000000000000000000000000000000000000000000000000000000000000000000000000000000000000000000000000000000000000000000000000000000000000000000000000000000000000000000000000000000000000000000000000000000000000000000000000000000000000000000000000000000000000000000000000000000000000000000000000000000000000000000000000000000000000000000000000000000000000000000000000000

000000000000000000000000000000000000000000000000000000000000000000000000000000000000000000000000000000000000000000000000000000000000000000000000000000000000000000000000000000000000000000000000000000000000000000000000000000000000000000000000000000000000000000000000000000000000000000000000000000000000000000000000000000000000000000000000000000000000000000000000000000000000000000000000000000000000000000000000000000000000000000000000000000000000000000000000000000000000000000000000000000000000000000000000000000000000000000000000000000000000000000000000000000000000000000000000000000000000000000000000000000000000000000000000000000000000000000000000000000000000000000000000000000000000000000000000000000000000000000000000000000

000000000000000000000000000000000000000000000000000000000000000000000000000000000000000000000000000000000000000000000000000000000000000000000000000000000000000000000000000000000000000000000000000000000000000000000000000000000000000000000000000000000000000000000000000000000000000000000000000000000000000000000000000000000000000000000000000000000000000000000000000000000000000000000000000000000000000000000000000000000000000000000000000000000000000000000000000000000000000000000000000000000000000000000000000000000000000000000000000000000000000000000000000000000000000000000000000000000000000000000000000000000000000000000000000000000000000000000000000000000000000000000000000000000000000000000000000000000000000000000000000000

000000000000000000000000000000000000000000000000000000000000000000000000000000000000000000000000000000000000000000000000000000000000000000000000000000000000000000000000000000000000000000000000000000000000000000000000000000000000000000000000000000000000000000000000000000000000000000000000000000000000000000000000000000000000000000000000000000000000000000000000000000000000000000000000000000000000000000000000000000000000000000000000000000000000000000000000000000000000000000000000000000000000000000000000000000000000000000000000000000000000000000000000000000000000000000000000000000000000000000000000000000000000000000000000000000000000000000000000000000000000000000000000000000000000000000000000000000000000000000000000000000

>DP00935

MSHSVKIYDTCIGCTQCVRACPLDVLEMVPWDGCKAGQIASSPRTEDCVGCKRCETACPTDFLSIRVYLGAETTRSMGLAY

000000000000000000000000000000000000000000000011111111111100000000011111111111110

000000000000000000000000000000000000000000000000000000000000000000000000000000000

000000000000000000000000000000000000000000000000000000000000000000000000000000000

000000000000000000000000000000000000000000000000000000000000000000000000000000000

000000000000000000000000000000000000000000000000000000000000000000000000000000000

>DP00378

MSDQDHSMDEMTAVVKIEKGVGGNNGGNGNGGGAFSQARSSSTGSSSSTGGGGQESQPSPLALLAATCSRIESPNENSNNSQGPSQSGGTGELDLTATQLSQGANGWQIISSSSGATPTSKEQSGSSTNGSNGSESSKNRTVSGGQYVVAAAPNLQNQQVLTGLPGVMPNIQYQVIPQFQTVDGQQLQFAATGAQVQQDGSGQIQIIPGANQQIITNRGSGGNIIAAMPNLLQQAVPLQGLANNVLSGQTQYVTNVPVALNGNITLLPVNSVSAATLTPSSQAVTISSSGSQESGSQPVTSGTTISSASLVSSQASSSSFFTNANSYSTTTTTSNMGIMNFTTSGSSGTNSQGQTPQRVSGLQGSDALNIQQNQTSGGSLQAGQQKEGEQNQQTQQQQILIQPQLVQGGQALQALQAAPLSGQTFTTQAISQETLQNLQLQAVPNSGPIIIRTPTVGPNGQVSWQTLQLQNLQVQNPQAQTITLAPMQGVSLGQTSSSNTTLTPIASAASIPAGTVTVNAAQLSSMPGLQTINLSALGTSGIQVHPIQGLPLAIANAPGDHGAQLGLHGAGGDGIHDDTAGGEEGENSPDAQPQAGRRTRREACTCPYCKDSEGRGSGDPGKKKQHICHIQGCGKVYGKTSHLRAHLRWHTGERPFMCTWSYCGKRFTRSDELQRHKRTHTGEKKFACPECPKRFMRSDHLSKHIKTHQNKKGGPGVALSVGTLPLDSGAGSEGSGTATPSALITTNMVAMEAICPEGIARLANSGINVMQVADLQSINISGNGF

00000000000000000000000000000000000000000000000000000000000000000000000000000000000000000000000000000000000000000000000000000000000000000000000000000000000000000000000000000000000000000000000000000000000000000000000000000000000000000000000000000000000000000000000000000000000000000000000000000000000000000000000000000000000000000000000000000111111111111111111111111111111111111111111111111111111111111111111111111111111111111111111111111111111111111111111111111111111111111111111111111111000000000000000000000000000000000000000000000000000000000000000000000000000000000000000000000000000000000000000000000000000000000000000000000000000000000000000000000000000000000000000000000000000000000000000000000000000000000000000000000000000000000000000000000000000000000000000000000000000000000

00000000000000000000000000000000000000000000000000000000000000000000000000000000000000000000000000000000000000000000000000000000000000000000000000000000000000000000000000000000000000000000000000000000000000000000000000000000000000000000000000000000000000000000000000000000000000000000000000000000000000000000000000000000000000000000000000000000000000000000000000000000000000000000000000000000000000000000000000000000000000000000000000000000000000000000000000000000000000000000000000000000000000000000000000000000000000000000000000000000000000000000000000000000000000000000000000000000000000000000000000000000000000000000000000000000000000000000000000000000000000000000000000000000000000000000000000000000000000000000000000000000000000000000000000000000000000000000000000000000000000000

00000000000000000000000000000000000000000000000000000000000000000000000000000000000000000000000000000000000000000000000000000000000000000000000000000000000000000000000000000000000000000000000000000000000000000000000000000000000000000000000000000000000000000000000000000000000000000000000000000000000000000000000000000000000000000000000000000000000000000000000000000000000000000000000000000000000000000000000000000000000000000000000000000000000000000000000000000000000000000000000000000000000000000000000000000000000000000000000000000000000000000000000000000000000000000000000000000000000000000000000000000000000000000000000000000000000000000000000000000000000000000000000000000000000000000000000000000000000000000000000000000000000000000000000000000000000000000000000000000000000000000

00000000000000000000000000000000000000000000000000000000000000000000000000000000000000000000000000000000000000000000000000000000000000000000000000000000000000000000000000000000000000000000000000000000000000000000000000000000000000000000000000000000000000000000000000000000000000000000000000000000000000000000000000000000000000000000000000000000000000000000000000000000000000000000000000000000000000000000000000000000000000000000000000000000000000000000000000000000000000000000000000000000000000000000000000000000000000000000000000000000000000000000000000000000000000000000000000000000000000000000000000000000000000000000000000000000000000000000000000000000000000000000000000000000000000000000000000000000000000000000000000000000000000000000000000000000000000000000000000000000000000000

00000000000000000000000000000000000000000000000000000000000000000000000000000000000000000000000000000000000000000000000000000000000000000000000000000000000000000000000000000000000000000000000000000000000000000000000000000000000000000000000000000000000000000000000000000000000000000000000000000000000000000000000000000000000000000000000000000000000000000000000000000000000000000000000000000000000000000000000000000000000000000000000000000000000000000000000000000000000000000000000000000000000000000000000000000000000000000000000000000000000000000000000000000000000000000000000000000000000000000000000000000000000000000000000000000000000000000000000000000000000000000000000000000000000000000000000000000000000000000000000000000000000000000000000000000000000000000000000000000000000000000

>DP01050

MAKGQSLQDPFLNALRRERVPVSIYLVNGIKLQGQIESFDQFVILLKNTVSQMVYKHAISTVVPSRPVSHHSNNAGGGTSSNYHHGSSAQNTSAQQDSEETE

000000000000000000000000000000000000000000000000000000000000000000000000000000000000000000000000000000

000000000000000000000000000000000000000000000000000000000000000000000000001111111111111111111111111111

000000000000000000000000000000000000000000000000000000000000000000000000000000000000000000000000000000

000000000000000000000000000000000000000000000000000000000000000000000000000000000000000000000000000000

000000000000000000000000000000000000000000000000000000000000000000000000000000000000000000000000000000

>DP00675

MELITNELLYKTYKQKPVGVEEPVYDQAGDPLFGERGAVHPQSTLKLPHKRGERDVPTNLASLPKRGDCRSGNSRGPVSGIYLKPGPLFYQDYKGPVYHRAPLELFEEGSMCETTKRIGRVTGSDGKLYHIYVCIDGCIIIKSATRSYQRVFRWVHNRLDCPLWVTTCSDTKEEGATKKKTQKPDRLERGKMKIVPKESEKDSKTKPPDATIVVEGVKYQVRKKGKTKSKNTQDGLYHNKNKPQESRKKLEKALLAWAIIAIVLFQVTMGENITQWNLQDNGTEGIQRAMFQRGVNRSLHGIWPEKICTGVPSHLATDIELKTIHGMMDASEKTNYTCCRLQRHEWNKHGWCNWYNIEPWILVMNRTQANLTEGQPPRECAVTCRYDRASDLNVVTQARDSPTPLTGCKKGKNFSFAGILMRGPCNFEIAASDVLFKEHERISMFQDTTLYLVDGLTNSLEGARQGTAKLTTWLGKQLGILGKKLENKSKTWFGAYAASPYCDVDRKIGYIWYTKNCTPACLPKNTKIVGPGKFGTNAEDGKILHEMGGHLSEVLLLSLVVLSDFAPETASVMYLILHFSIPQSHVDVMDCDKTQLNLTVELTTAEVIPGSVWNLGKYVCIRPNWWPYETTVVLAFEEVSQVVKLVLRALRDLTRIWNAATTTAFLVCLVKIVRGQMVQGILWLLLITGVQGHLDCKPEFSYAIAKDERIGQLGAEGLTTTWKEYSPGMKLEDTMVIAWCEDGKLMYLQRCTRETRYLAILHTRALPTSVVFKKLFDGRKQEDVVEMNDNFEFGLCPCDAKPIVRGKFNTTLLNGPAFQMVCPIGWTGTVSCTSFNMDTLATTVVRTYRRSKPFPHRQGCITQKNLGEDLHNCILGGNWTCVPGDQLLYKGGSIESCKWCGYQFKESEGLPHYPIGKCKLENETGYRLVDSTSCNREGVAIVPQGTLKCKIGKTTVQVIAMDTKLGPMPCRPYEIISSEGPVEKTACTFNYTKTLKNKYFEPRDSYFQQYMLKGEYQYWFDLEVTDHHRDYFAESILVVVVALLGGRYVLWLLVTYMVLSEQKALGIQYGSGEVVMMGNLLTHNNIEVVTYFLLLYLLLREESVKKWVLLLYHILVVHPIKSVIVILLMIGDVVKADSGGQEYLGKIDLCFTTVVLIVIGLIIARRDPTIVPLVTIMAALRVTELTHQPGVDIAVAVMTITLLMVSYVTDYFRYKKWLQCILSLVSAVFLIRSLIYLGRIEMPEVTIPNWRPLTLILLYLISTTIVTRWKVDVAGLLLQCVPILLLVTTLWADFLTLILILPTYELVKLYYLKTVRTDTERSWLGGIDYTRVDSIYDVDESGEGVYLFPSRQKAQGNFSILLPLIKATLISCVSSKWQLIYMSYLTLDFMYYMHRKVIEEISGGTNIISRLVAALIELNWSMEEEESKGLKKFYLLSGRLRNLIIKHKVRNETVASWYGEEEVYGMPKIMTIIKASTLSKSRHCIICTVCEGREWKGGTCPKCGRHGKPITCGMSLADFEERHYKRIFIREGNFEGMCSRCQGKHRRFEMDREPKSARYCAECNRLHPAEEGDFWAESSMLGLKITYFALMDGKVYDITEWAGCQRVGISPDTHRVPCHISFGSRMPFRQEYNGFVQYTARGQLFLRNLPVLATKVKMLMVGNLGEEIGNLEHLGWILRGPAVCKKITEHEKCHINILDKLTAFFGIMPRGTTPRAPVRFPTSLLKVRRGLETAWAYTHQGGISSVDHVTAGKDLLVCDSMGRTRVVCQSNNRLTDETEYGVKTDSGCPDGARCYVLNPEAVNISGSKGAVVHLQKTGGEFTCVTASGTPAFFDLKNLKGWSGLPIFEASSGRVVGRVKVGKNEESKPTKIMSGIQTVSKNRADLTEMVKKITSMNRGDFKQITLATGAGKTTELPKAVIEEIGRHKRVLVLIPLRAAAESVYQYMRLKHPSISFNLRIGDMKEGDMATGITYASYGYFCQMPQPKLRAAMVEYSYIFLDEYHCATPEQLAIIGKIHRFSESIRVVAMTATPAGSVTTTGQKHPIEEFIAPEVMKGEDLGSQFLDIAGLKIPVDEMKGNMLVFVPTRNMAVEVAKKLKAKGYNSGYYYSGEDPANLRVVTSQSPYVIVATNAIESGVTLPDLDTVIDTGLKCEKRVRVSSKIPFIVTGLKRMAVTVGEQAQRRGRVGRVKPGRYYRSQETATGSKDYHYDLLQAQRYGIEDGINVTKSFREMNYDWSLYEEDSLLITQLEILNNLLISEDLPAAVKNIMARTDHPEPIQLAYNSYEVQVPVLFPKIRNGEVTDTYENYSFLNARKLGEDVPVYIYATEDEDLAVDLLGLDWPDPGNQQVVETGKALKQVTGLSSAENALLVALFGYVGYQALSKRHVPMITDIYTIEDQRLEDTTHLQYAPNAIKTDGTETELKELASGDVEKIMGAISDYAAGGLEFVKSQAEKIKTAPLFKENAEAAKGYVQKFIDSLIENKEEIIRYGLWGTHTALYKSIAARLGHETAFATLVLKWLAFGGESVSDHVKQAAVDLVVYYVMNKPSFPGDSETQQEGRRFVASLFISALATYTYKTWNYHNLSKVVEPALAYLPYATSALKMFTPTRLESVVILSTTIYKTYLSIRKGKSDGLLGTGISAAMEILSQNPVSVGISVMLGVGAIAAHNAIESSEQKRTLLMKVFVKNFLDQAATDELVKENPEKIIMALFEAVQTIGNPLRLIYHLYGVYYKGWEAKELSERTAGRNLFTLIMFEAFELLGMDSQGKIRNLSGNYILDLIYGLHKQINRGLKKMVLGWAPAPFSCDWTPSDERIRLPTDNYLRVETRCPCGYEMKAFKNVGGKLTKVEESGPFLCRNRPGRGPVNYRVTKYYDDNLREIKPVAKLEGQVEHYYKGVTAKIDYSKGKMLLATDKWEVEHGVITRLAKRYTGVGFNGAYLGDEPNHRALVERDCATITKNTVQFLKMKKGCAFTYDLTISNLTRLIELVHRNNLEEKEIPTATVTTWLAYTFVNEDVGTIKPVLGERVIPDPVVDINLQPEVQVDTSEVGITIIGRETLMTTGVTPVLEKVEPDASDNQNSVKIGLDEGNYPGPGIQTHTLTEEIHNRDARPFIMILGSRNSISNRAKTARNINLYTGNDPREIRDLMAAGRMLVVALRDVDPELSEMVDFKGTFLDREALEALSLGQPKPKQVTKEAVRNLIEQKKDVEIPNWFASDDPVFLEVALKNDKYYLVGDVGELKDQAKALGATDQTRIIKEVGSRTYAMKLSSWFLKASNKQMSLTPLFEELLLRCPPATKSNKGHMASAYQLAQGNWEPLGCGVHLGTIPARRVKIHPYEAYLKLKDFIEEEEKKPRVKDTVIREHNKWILKKIRFQGNLNTKKMLNPGKLSEQLDREGRKRNIYNHQIGTIMSSAGIRLEKLPIVRAQTDTKTFHEAIRDKIDKSENRQNPELHNKLLEIFHTIAQPTLKHTYGEVTWEQLEAGVNRKGAAGFLEKKNIGEVLDSEKHLVEQLVRDLKAGRKIKYYETAIPKNEKRDVSDDWQAGDLVVEKRPRVIQYPEAKTRLAITKVMYNWVKQQPVVIPGYEGKTPLFNIFDKVRKEWDSFNEPVAVSFDTKAWDTQVTSKDLQLIGEIQKYYYKKEWHKFIDTITDHMTEVPVITADGEVYIRNGQRGSGQPDTSAGNSMLNVLTMMYGFCESTGVPYKSFNRVARIHVCGDDGFLITEKGLGLKFANKGMQILHEAGKPQKITEGEKMKVAYRFEDIEFCSHTPVPVRWSDNTSSHMAGRDTAVILSKMATRLDSSGERGTTAYEKAVAFSFLLMYSWNPLVRRICLLVLSQQPETDPSKHATYYYKGDPIGAYKDVIGRNLSELKRTGFEKLANLNLSLSTLGVWTKHTSKRIIQDCVAIGKEEGNWLVKPDRLISSKTGHLYIPDKGFTLQGKHYEQLQLRTETNPVMGVGTERYKLGPIVNLLLRRLKILLMTAVGVSS

0000000000000000000000000000000000000000000000000000000000000000000000000000000000000000000000000000000000000000000000000000000000000000000000000000000000000000000000000000000000000000000000000000000000000000000000000000000000000000000000000000000000000000000000000000000000000000000000000000000000000000000000000000000000000000000000000000000000000000000000000000000000000000000000000000000000000000000000000000000000000000000000000000000000000000000000000000000000000000000000000000000000000000000000000000000000000000000000000000000000000000000000000000000000000000000000000000000000000000000000000000000000000000000000000000000000000000000000000000000000000000000000000000000000000000000000000000000000000000000000000000000000000000000000000000000000000000000000000000000000000000000000000000000000000000000000000000000000000000000000000000000000000000000000000000000000000000000000000000000000000000000000000000000000000000000000000000000000000000000000000000000000000000000000000000000000000000000000000000000000000000000000000000000000000000000000000000000000000000000000000000000000000000000000000000000000000000000000000000000000000000000000000000000000000000000000000000000000000000000000000000000000000000000000000000000000000000000000000000000000000000000000000000000000000000000000000000000000000000000000000000000000000000000000000000000000000000000000000000000000000000000000000000000000000000000000000000000000000000000000000000000000000000000000000000000000000000000000000000000000000000000000000000000000000000000000000000000000000000000000000000000000000000000000000000000000000000000000000000000000000000000000000000000000000000000000000000000000000000000000000000000000000000000000000000000000000000000000000000000000000000000000000000000000000000000000000000000000000000000000000000000000000000000000000000000000000000000000000000000000000000000000000000000000000000000000000000000000000000000000000000000000000000000000000000000000000000000000000000000000000000000000000000000000000000000000000000000000000000000000000000000000000000000000000000000000000000000000000000000000000000000000000000000000000000000000000000000000000000000000000000000000000000000000000000000000000000000000000000000000000000000000000000000000000000000000000000000000000000000000000000000000000000000000000000000000000000000000000000000000000000000000000000000000000000000000000000000000000000000000000000000000000000000000000000000000000000000000000000000000000000000000000000000000000000000000000000000000000000000000000000000000000000000000000000000000000000000000000000000000000000000000000000000000000000000000000000000000000000000000000000000000000000000000000000000000000000000000000000000000000000000000000000000000000000000000000000000000000000000000000000000000000000000000000000000000000000000000000000000000000000000000000000000000000000000000000000000000000000000000000000000000000000000000000000000000000000000000000000000000000000000000000000000000000000000000000000000000000000000000000000000000000000000000000000000000000000000000000000000000000000000000000000000000000000000000000000000000000000000000000000000000000000000000000000000000000000000000000000000000000000000000000000000000000000000000000000000000000000000000000000000000000000000000000000000000000000000000000000000000000000000000000000000000000000000000000000000000000000000000000000000000000000000000000000000000000000000000000000000000000000000000000000000000000000000000000000000000000000000000000000000000000000000000000000000000000000000000000000000000000000000000000000000000000000000000000000000000000000000000000000000000000000000000000000000000000000000000000000000000000000000000000000000000000000000000000000000000000000000000000000000000000000000000000000000000000000000000000000000000000000000000000000000000000000000000000000000000000000000000000000000000000000000000000000000000000000000000000000000000000000000000000000000000000000000000000000000000000000000000000000000000000000000000000000

0000000000000000000000000000000000000000000000000000000000000000000000000000000000000000000000000000000000000000000000000000000000000000000000000000000000000000000000001111111111111111111111111111111111111111111111111111111111111111111111111111111111111111111111111111110000000000000000000000000000000000000000000000000000000000000000000000000000000000000000000000000000000000000000000000000000000000000000000000000000000000000000000000000000000000000000000000000000000000000000000000000000000000000000000000000000000000000000000000000000000000000000000000000000000000000000000000000000000000000000000000000000000000000000000000000000000000000000000000000000000000000000000000000000000000000000000000000000000000000000000000000000000000000000000000000000000000000000000000000000000000000000000000000000000000000000000000000000000000000000000000000000000000000000000000000000000000000000000000000000000000000000000000000000000000000000000000000000000000000000000000000000000000000000000000000000000000000000000000000000000000000000000000000000000000000000000000000000000000000000000000000000000000000000000000000000000000000000000000000000000000000000000000000000000000000000000000000000000000000000000000000000000000000000000000000000000000000000000000000000000000000000000000000000000000000000000000000000000000000000000000000000000000000000000000000000000000000000000000000000000000000000000000000000000000000000000000000000000000000000000000000000000000000000000000000000000000000000000000000000000000000000000000000000000000000000000000000000000000000000000000000000000000000000000000000000000000000000000000000000000000000000000000000000000000000000000000000000000000000000000000000000000000000000000000000000000000000000000000000000000000000000000000000000000000000000000000000000000000000000000000000000000000000000000000000000000000000000000000000000000000000000000000000000000000000000000000000000000000000000000000000000000000000000000000000000000000000000000000000000000000000000000000000000000000000000000000000000000000000000000000000000000000000000000000000000000000000000000000000000000000000000000000000000000000000000000000000000000000000000000000000000000000000000000000000000000000000000000000000000000000000000000000000000000000000000000000000000000000000000000000000000000000000000000000000000000000000000000000000000000000000000000000000000000000000000000000000000000000000000000000000000000000000000000000000000000000000000000000000000000000000000000000000000000000000000000000000000000000000000000000000000000000000000000000000000000000000000000000000000000000000000000000000000000000000000000000000000000000000000000000000000000000000000000000000000000000000000000000000000000000000000000000000000000000000000000000000000000000000000000000000000000000000000000000000000000000000000000000000000000000000000000000000000000000000000000000000000000000000000000000000000000000000000000000000000000000000000000000000000000000000000000000000000000000000000000000000000000000000000000000000000000000000000000000000000000000000000000000000000000000000000000000000000000000000000000000000000000000000000000000000000000000000000000000000000000000000000000000000000000000000000000000000000000000000000000000000000000000000000000000000000000000000000000000000000000000000000000000000000000000000000000000000000000000000000000000000000000000000000000000000000000000000000000000000000000000000000000000000000000000000000000000000000000000000000000000000000000000000000000000000000000000000000000000000000000000000000000000000000000000000000000000000000000000000000000000000000000000000000000000000000000000000000000000000000000000000000000000000000000000000000000000000000000000000000000000000000000000000000000000000000000000000000000000000000000000000000000000000000000000000000000000000000000000000000000000000000000000000000000000000000000000000000000000000000000000000000000000000000000000000000000000000000000000000000000000000000000000000000000000000000000000000000000000

0000000000000000000000000000000000000000000000000000000000000000000000000000000000000000000000000000000000000000000000000000000000000000000000000000000000000000000000000000000000000000000000000000000000000000000000000000000000000000000000000000000000000000000000000000000000000000000000000000000000000000000000000000000000000000000000000000000000000000000000000000000000000000000000000000000000000000000000000000000000000000000000000000000000000000000000000000000000000000000000000000000000000000000000000000000000000000000000000000000000000000000000000000000000000000000000000000000000000000000000000000000000000000000000000000000000000000000000000000000000000000000000000000000000000000000000000000000000000000000000000000000000000000000000000000000000000000000000000000000000000000000000000000000000000000000000000000000000000000000000000000000000000000000000000000000000000000000000000000000000000000000000000000000000000000000000000000000000000000000000000000000000000000000000000000000000000000000000000000000000000000000000000000000000000000000000000000000000000000000000000000000000000000000000000000000000000000000000000000000000000000000000000000000000000000000000000000000000000000000000000000000000000000000000000000000000000000000000000000000000000000000000000000000000000000000000000000000000000000000000000000000000000000000000000000000000000000000000000000000000000000000000000000000000000000000000000000000000000000000000000000000000000000000000000000000000000000000000000000000000000000000000000000000000000000000000000000000000000000000000000000000000000000000000000000000000000000000000000000000000000000000000000000000000000000000000000000000000000000000000000000000000000000000000000000000000000000000000000000000000000000000000000000000000000000000000000000000000000000000000000000000000000000000000000000000000000000000000000000000000000000000000000000000000000000000000000000000000000000000000000000000000000000000000000000000000000000000000000000000000000000000000000000000000000000000000000000000000000000000000000000000000000000000000000000000000000000000000000000000000000000000000000000000000000000000000000000000000000000000000000000000000000000000000000000000000000000000000000000000000000000000000000000000000000000000000000000000000000000000000000000000000000000000000000000000000000000000000000000000000000000000000000000000000000000000000000000000000000000000000000000000000000000000000000000000000000000000000000000000000000000000000000000000000000000000000000000000000000000000000000000000000000000000000000000000000000000000000000000000000000000000000000000000000000000000000000000000000000000000000000000000000000000000000000000000000000000000000000000000000000000000000000000000000000000000000000000000000000000000000000000000000000000000000000000000000000000000000000000000000000000000000000000000000000000000000000000000000000000000000000000000000000000000000000000000000000000000000000000000000000000000000000000000000000000000000000000000000000000000000000000000000000000000000000000000000000000000000000000000000000000000000000000000000000000000000000000000000000000000000000000000000000000000000000000000000000000000000000000000000000000000000000000000000000000000000000000000000000000000000000000000000000000000000000000000000000000000000000000000000000000000000000000000000000000000000000000000000000000000000000000000000000000000000000000000000000000000000000000000000000000000000000000000000000000000000000000000000000000000000000000000000000000000000000000000000000000000000000000000000000000000000000000000000000000000000000000000000000000000000000000000000000000000000000000000000000000000000000000000000000000000000000000000000000000000000000000000000000000000000000000000000000000000000000000000000000000000000000000000000000000000000000000000000000000000000000000000000000000000000000000000000000000000000000000000000000000000000000000000000000000000000000000000000000000000000000000000000000000000000000000000000000000000000000000

0000000000000000000000000000000000000000000000000000000000000000000000000000000000000000000000000000000000000000000000000000000000000000000000000000000000000000000000000000000000000000000000000000000000000000000000000000000000000000000000000000000000000000000000000000000000000000000000000000000000000000000000000000000000000000000000000000000000000000000000000000000000000000000000000000000000000000000000000000000000000000000000000000000000000000000000000000000000000000000000000000000000000000000000000000000000000000000000000000000000000000000000000000000000000000000000000000000000000000000000000000000000000000000000000000000000000000000000000000000000000000000000000000000000000000000000000000000000000000000000000000000000000000000000000000000000000000000000000000000000000000000000000000000000000000000000000000000000000000000000000000000000000000000000000000000000000000000000000000000000000000000000000000000000000000000000000000000000000000000000000000000000000000000000000000000000000000000000000000000000000000000000000000000000000000000000000000000000000000000000000000000000000000000000000000000000000000000000000000000000000000000000000000000000000000000000000000000000000000000000000000000000000000000000000000000000000000000000000000000000000000000000000000000000000000000000000000000000000000000000000000000000000000000000000000000000000000000000000000000000000000000000000000000000000000000000000000000000000000000000000000000000000000000000000000000000000000000000000000000000000000000000000000000000000000000000000000000000000000000000000000000000000000000000000000000000000000000000000000000000000000000000000000000000000000000000000000000000000000000000000000000000000000000000000000000000000000000000000000000000000000000000000000000000000000000000000000000000000000000000000000000000000000000000000000000000000000000000000000000000000000000000000000000000000000000000000000000000000000000000000000000000000000000000000000000000000000000000000000000000000000000000000000000000000000000000000000000000000000000000000000000000000000000000000000000000000000000000000000000000000000000000000000000000000000000000000000000000000000000000000000000000000000000000000000000000000000000000000000000000000000000000000000000000000000000000000000000000000000000000000000000000000000000000000000000000000000000000000000000000000000000000000000000000000000000000000000000000000000000000000000000000000000000000000000000000000000000000000000000000000000000000000000000000000000000000000000000000000000000000000000000000000000000000000000000000000000000000000000000000000000000000000000000000000000000000000000000000000000000000000000000000000000000000000000000000000000000000000000000000000000000000000000000000000000000000000000000000000000000000000000000000000000000000000000000000000000000000000000000000000000000000000000000000000000000000000000000000000000000000000000000000000000000000000000000000000000000000000000000000000000000000000000000000000000000000000000000000000000000000000000000000000000000000000000000000000000000000000000000000000000000000000000000000000000000000000000000000000000000000000000000000000000000000000000000000000000000000000000000000000000000000000000000000000000000000000000000000000000000000000000000000000000000000000000000000000000000000000000000000000000000000000000000000000000000000000000000000000000000000000000000000000000000000000000000000000000000000000000000000000000000000000000000000000000000000000000000000000000000000000000000000000000000000000000000000000000000000000000000000000000000000000000000000000000000000000000000000000000000000000000000000000000000000000000000000000000000000000000000000000000000000000000000000000000000000000000000000000000000000000000000000000000000000000000000000000000000000000000000000000000000000000000000000000000000000000000000000000000000000000000000000000000000000000000000000000000000000000000000000000000000000000000000000000000000000000000000000000000000000000000000000000000000000000000

0000000000000000000000000000000000000000000000000000000000000000000000000000000000000000000000000000000000000000000000000000000000000000000000000000000000000000000000000000000000000000000000000000000000000000000000000000000000000000000000000000000000000000000000000000000000000000000000000000000000000000000000000000000000000000000000000000000000000000000000000000000000000000000000000000000000000000000000000000000000000000000000000000000000000000000000000000000000000000000000000000000000000000000000000000000000000000000000000000000000000000000000000000000000000000000000000000000000000000000000000000000000000000000000000000000000000000000000000000000000000000000000000000000000000000000000000000000000000000000000000000000000000000000000000000000000000000000000000000000000000000000000000000000000000000000000000000000000000000000000000000000000000000000000000000000000000000000000000000000000000000000000000000000000000000000000000000000000000000000000000000000000000000000000000000000000000000000000000000000000000000000000000000000000000000000000000000000000000000000000000000000000000000000000000000000000000000000000000000000000000000000000000000000000000000000000000000000000000000000000000000000000000000000000000000000000000000000000000000000000000000000000000000000000000000000000000000000000000000000000000000000000000000000000000000000000000000000000000000000000000000000000000000000000000000000000000000000000000000000000000000000000000000000000000000000000000000000000000000000000000000000000000000000000000000000000000000000000000000000000000000000000000000000000000000000000000000000000000000000000000000000000000000000000000000000000000000000000000000000000000000000000000000000000000000000000000000000000000000000000000000000000000000000000000000000000000000000000000000000000000000000000000000000000000000000000000000000000000000000000000000000000000000000000000000000000000000000000000000000000000000000000000000000000000000000000000000000000000000000000000000000000000000000000000000000000000000000000000000000000000000000000000000000000000000000000000000000000000000000000000000000000000000000000000000000000000000000000000000000000000000000000000000000000000000000000000000000000000000000000000000000000000000000000000000000000000000000000000000000000000000000000000000000000000000000000000000000000000000000000000000000000000000000000000000000000000000000000000000000000000000000000000000000000000000000000000000000000000000000000000000000000000000000000000000000000000000000000000000000000000000000000000000000000000000000000000000000000000000000000000000000000000000000000000000000000000000000000000000000000000000000000000000000000000000000000000000000000000000000000000000000000000000000000000000000000000000000000000000000000000000000000000000000000000000000000000000000000000000000000000000000000000000000000000000000000000000000000000000000000000000000000000000000000000000000000000000000000000000000000000000000000000000000000000000000000000000000000000000000000000000000000000000000000000000000000000000000000000000000000000000000000000000000000000000000000000000000000000000000000000000000000000000000000000000000000000000000000000000000000000000000000000000000000000000000000000000000000000000000000000000000000000000000000000000000000000000000000000000000000000000000000000000000000000000000000000000000000000000000000000000000000000000000000000000000000000000000000000000000000000000000000000000000000000000000000000000000000000000000000000000000000000000000000000000000000000000000000000000000000000000000000000000000000000000000000000000000000000000000000000000000000000000000000000000000000000000000000000000000000000000000000000000000000000000000000000000000000000000000000000000000000000000000000000000000000000000000000000000000000000000000000000000000000000000000000000000000000000000000000000000000000000000000000000000000000000000000000000000000000000000000000000000000000000000000000000000000000000000000000000000000000000000000000000000

>DP01228

MSDAAVDTSSEITTEDLKEKKEVVEEAENGRDAPADEENGEQEADNEVDEEQEEGGEEEGDGEEEDGDEDEGAESATDKRAAEDDEDNDVDTKKQKTDEDD

00000000000000000000000000000000000000000000000000000000000000000000000000000000000000000000000000000

11111111111111111111111111111111111111111111111111111111111111111111111111111111111111111111111111111

00000000000000000000000000000000000000000000000000000000000000000000000000000000000000000000000000000

00000000000000000000000000000000000000000000000000000000000000000000000000000000000000000000000000000

00000000000000000000000000000000000000000000000000000000000000000000000000000000000000000000000000000

>DP01131

MEIPVPVQPSWLRRASAPLPGLSAPGRLFDQRFGEGLLEAELAALCPTTLAPYYLRAPSVALPVAQVPTDPGHFSVLLDVKHFSPEEIAVKVVGEHVEVHARHEERPDEHGFVAREFHRRYRLPPGVDPAAVTSALSPEGVLSIQAAPASAQAPPPAAAK

0000000000000000000000000000000000000000000000000000000000000000000000000000000000000000000000000000000000000000000000000000000000000000000000000000000000000000

1111111111111111111111111111111111111111111111111111111100000000000000000000000000000000000000000000000000000000000000000000000000000000000000000000000000000000

0000000000000000000000000000000000000000000000000000000000000000000000000000000000000000000000000000000000000000000000000000000000000000000000000000000000000000

0000000000000000000000000000000000000000000000000000000000000000000000000000000000000000000000000000000000000000000000000000000000000000000000000000000000000000

0000000000000000000000000000000000000000000000000000000000000000000000000000000000000000000000000000000000000000000000000000000000000000000000000000000000000000

>DP00412

MNIRPLHDRVIVKRKEVETKSAGGIVLTGSAAAKSTRGEVLAVGNGRILENGEVKPLDVKVGDIVIFNDGYGVKSEKIDNEEVLIMSESDILAIVEA

0000000000000000000000000000000000000000000000000000000000000000000000000000000000000000000000000

0000000000000000111111111111111000000000000000000000000000000000000000000000000000000000000000000

0000000000000000000000000000000000000000000000000000000000000000000000000000000000000000000000000

0000000000000000000000000000000000000000000000000000000000000000000000000000000000000000000000000

0000000000000000000000000000000000000000000000000000000000000000000000000000000000000000000000000

>DP02715

MNQNLLVTKRDGSTERINLDKIHRVLDWAAEGLHNVSISQVELRSHIQFYDGIKTSDIHETIIKAAADLISRDAPDYQYLAARLAIFHLRKKAYGQFEPPALYDHVVKMVEMGKYDNHLLEDYTEEEFKQMDTFIDHDRDMTFSYAAVKQLEGKYLVQNRVTGEIYESAQFLYILVAACLFSNYPRETRLQYVKRFYDAVSTFKISLPTPIMSGVRTPTRQFSSCVLIECGDSLDSINATSSAIVKYVSQRAGIGINAGRIRALGSPIRGGEAFHTGCIPFYKHFQTAVKSCSQGGVRGGAATLFYPMWHLEVESLLVLKNNRGVEGNRVRHMDYGVQINKLMYTRLLKGEDITLFSPSDVPGLYDAFFADQEEFERLYTKYEKDDSIRKQRVKAVELFSLMMQERASTGRIYIQNVDHCNTHSPFDPAIAPVRQSNLCLEIALPTKPLNDVNDENGEIALCTLSAFNLGAINNLDELEELAILAVRALDALLDYQDYPIPAAKRGAMGRRTLGIGVINFAYYLAKHGKRYSDGSANNLTHKTFEAIQYYLLKASNELAKEQGACPWFNETTYAKGILPIDTYKKDLDTIANEPLHYDWEALRESIKTHGLRNSTLSALMPSETSSQISNATNGIEPPRGYVSIKASKDGILRQVVPDYEHLHDAYELLWEMPGNDGYLQLVGIMQKFIDQSISANTNYDPSRFPSGKVPMQQLLKDLLTAYKFGVKTLYYQNTRDGAEDAQDDLVPSIQDDGCESGACKI

00000000000000000000000000000000000000000000000000000000000000000000000000000000000000000000000000000000000000000000000000000000000000000000000000000000000000000000000000000000000000000000000000000000000000000000000000000000000000000000000000000000000000000000000000000000000000000000000000000000000000000000000000000000000000000000000000000000000000000000000000000000000000000000000000000000000000000000000000000000000000000000000000000000000000000000000000000000000000000000000000000000000000000000000000000000000000000000000000000000000000000000000000000000000000000000000000000000000000000000000000000000000000000000000000000000000000000000000000000000000000000000000000000000000000000000000000000000000000000000000000000000000000000000000000000000000000000

00000000000000000000000000000000000000000000000000000000000000000000000000000000000000000000000000000000000000000000000000000000000000000000000000000000000000000000000000000000000000000000000000000000000000000000000000000000000000000000000000000000000000000000000000000000000000000000000000000000000000000000000000000000000000000000000000000000000000000000000000000000000000000000000000000000000000000000000000000000000000000000000000000000000000000000000000000000000000000000000000000000000000000000000000000000000000000000000000000000000000000000000000000000000000000000000000000000000000000000000000000000000000000000000000000000000000000000000000000000000000000000000000000000000000000000000000000000000000000000000000000000000000001111111111111111111111111

00000000000000000000000000000000000000000000000000000000000000000000000000000000000000000000000000000000000000000000000000000000000000000000000000000000000000000000000000000000000000000000000000000000000000000000000000000000000000000000000000000000000000000000000000000000000000000000000000000000000000000000000000000000000000000000000000000000000000000000000000000000000000000000000000000000000000000000000000000000000000000000000000000000000000000000000000000000000000000000000000000000000000000000000000000000000000000000000000000000000000000000000000000000000000000000000000000000000000000000000000000000000000000000000000000000000000000000000000000000000000000000000000000000000000000000000000000000000000000000000000000000000000000000000000000000000000000

00000000000000000000000000000000000000000000000000000000000000000000000000000000000000000000000000000000000000000000000000000000000000000000000000000000000000000000000000000000000000000000000000000000000000000000000000000000000000000000000000000000000000000000000000000000000000000000000000000000000000000000000000000000000000000000000000000000000000000000000000000000000000000000000000000000000000000000000000000000000000000000000000000000000000000000000000000000000000000000000000000000000000000000000000000000000000000000000000000000000000000000000000000000000000000000000000000000000000000000000000000000000000000000000000000000000000000000000000000000000000000000000000000000000000000000000000000000000000000000000000000000000000000000000000000000000000000

00000000000000000000000000000000000000000000000000000000000000000000000000000000000000000000000000000000000000000000000000000000000000000000000000000000000000000000000000000000000000000000000000000000000000000000000000000000000000000000000000000000000000000000000000000000000000000000000000000000000000000000000000000000000000000000000000000000000000000000000000000000000000000000000000000000000000000000000000000000000000000000000000000000000000000000000000000000000000000000000000000000000000000000000000000000000000000000000000000000000000000000000000000000000000000000000000000000000000000000000000000000000000000000000000000000000000000000000000000000000000000000000000000000000000000000000000000000000000000000000000000000000000000000000000000000000000000

>DP02787

MKSLFKVTLLATTMAVALHAPITFAAEAAKPATAADSKAAFKNDDQKSAYALGASLGRYMENSLKEQEKLGIKLDKDQLIAGVQDAFADKSKLSDQEIEQTLQAFEARVKSSAQAKMEKDAADNEAKGKEYREKFAKEKGVKTSSTGLVYQVVEAGKGEAPKDSDTVVVNYKGTLIDGKEFDNSYTRGEPLSFRLDGVIPGWTEGLKNIKKGGKIKLVIPPELAYGKAGVPGIPPNSTLVFDVELLDVKPAPKADAKPEADAKAADSAKK

000000000000000000000000000000000000000000000000000000000000000000000000000000000000000000000000000000000000000000000000000000000000000000000000000000000000000000000000000000000000000000000000000000000000000000000000000000000000000000000000000000000000000000000000000000

000000000000001111111111111111111111111111111111111111111111111111111111111111111111111111111111111111111111111111000000000000000000000000000000000000000000000000000000000000000000000000000000000000000000000000000000000000000000000000000000000000000000000000000000000000

000000000000000000000000000000000000000000000000000000000000000000000000000000000000000000000000000000000000000000000000000000000000000000000000000000000000000000000000000000000000000000000000000000000000000000000000000000000000000000000000000000000000000000000000000000

000000000000000000000000000000000000000000000000000000000000000000000000000000000000000000000000000000000000000000000000000000000000000000000000000000000000000000000000000000000000000000000000000000000000000000000000000000000000000000000000000000000000000000000000000000

000000000000000000000000000000000000000000000000000000000000000000000000000000000000000000000000000000000000000000000000000000000000000000000000000000000000000000000000000000000000000000000000000000000000000000000000000000000000000000000000000000000000000000000000000000

>DP02908

MMSDQENENEHAKAFLGLAKCEEEVDAIEREVELYRLNKMKPVYEKRDAYIDEIAEFWKIVLSQHVSFANYIRASDFKYIDTIDKIKVEWLALESEMYDTRDFSITFHFHGIEGDFKEQQVTKVFQIKKGKDDQEDGILTSEPVPIEWPQSYDSINPDLIKDKRSPEGKKKYRQGMKTIFGWFRWTGLKPGKEFPHGDSLASLFSEEIYPFCVKYYAEAQRDLEDEEGESGLSADGDSEDDDGSLGEVDLPLSDEEPSSKKRKV

000000000000000000000000000000000000000000000000000000000000000000000000000000000000000000000000000000000000000000000000000000000000000000000000000000000000000000000000000000000000000000000000000000000000000000000000000000000000000000000000000000000000000000000000

000000000000000000000000000000000000000000000000000000000000000000000000000000000000000000000000000000000000000000000000000000000000000000000000000000000000000000000000000000000000000000000000000000000000000000000000000000000111111111111111111111111111111111111111

000000000000000000000000000000000000000000000000000000000000000000000000000000000000000000000000000000000000000000000000000000000000000000000000000000000000000000000000000000000000000000000000000000000000000000000000000000000000000000000000000000000000000000000000

000000000000000000000000000000000000000000000000000000000000000000000000000000000000000000000000000000000000000000000000000000000000000000000000000000000000000000000000000000000000000000000000000000000000000000000000000000000000000000000000000000000000000000000000

000000000000000000000000000000000000000000000000000000000000000000000000000000000000000000000000000000000000000000000000000000000000000000000000000000000000000000000000000000000000000000000000000000000000000000000000000000000000000000000000000000000000000000000000

>DP02341

MNLFERFSRVVKSYANALISSFEDPEKILEQTVIEMNSDLTKMRQATAQVLASQKQLQNKYKAAQQSSDDWYKRAQLALAKGDEDLAREALKRRKSFADNATALKTQLDQQKGVVDNLVSNTRLLESKIQEAKAKKDTLLARARTAKTATKVQEMIGTVNTSGALSAFEKMEEKVMAMESEADALTQIGTDELEGKFQMLETSSVDDDLADLKKELSGSSKKGELPPGRSTVAASTRYPFKDSEIENELNELRRKANDF

0000000000000000000000000000000000000000000000000000000000000000000000000000000000000000000000000000000000000000000000000000000000000000000000000000000000000000000000000000000000000000000000000000000000000000000000000000000000000000000000000000000000000000000

0000000000000000000000000000000000000000000000000000000000000000000000000000000000000000000000000000000000000000000000000000000000000000000000000000000000000000000000000000000000000000000000000000000000000000000000000000111111111111111111111111111111111111111

0000000000000000000000000000000000000000000000000000000000000000000000000000000000000000000000000000000000000000000000000000000000000000000000000000000000000000000000000000000000000000000000000000000000000000000000000000000000000000000000000000000000000000000

0000000000000000000000000000000000000000000000000000000000000000000000000000000000000000000000000000000000000000000000000000000000000000000000000000000000000000000000000000000000000000000000000000000000000000000000000000000000000000000000000000000000000000000

0000000000000000000000000000000000000000000000000000000000000000000000000000000000000000000000000000000000000000000000000000000000000000000000000000000000000000000000000000000000000000000000000000000000000000000000000000000000000000000000000000000000000000000

>DP00445

MDIAIHHPWIRRPFFPFHSPSRLFDQFFGEHLLESDLFPTSTSLSPFYLRPPSFLRAPSWFDTGLSEMRLEKDRFSVNLDVKHFSPEELKVKVLGDVIEVHGKHEERQDEHGFISREFHRKYRIPADVDPLTITSSLSSDGVLTVNGPRKQVSGPERTIPITREEKPAVTAAPKK

0000000000000000000000000000000000000000000000000000000000000000000000000000000000000000000000000000000000000000000000000000000000000000000000000000000000000000000000000000000

0000000000000000000000000000000000000000000000000000000000000000000000000000000000000000000000000000000000000000000000000000000000000000000000000000000000000000000001111111111

0000000000000000000000000000000000000000000000000000000000000000000000000000000000000000000000000000000000000000000000000000000000000000000000000000000000000000000000000000000

0000000000000000000000000000000000000000000000000000000000000000000000000000000000000000000000000000000000000000000000000000000000000000000000000000000000000000000000000000000

0000000000000000000000000000000000000000000000000000000000000000000000000000000000000000000000000000000000000000000000000000000000000000000000000000000000000000000000000000000

>DP00613

MPKTERRRDRDRDRDRERRNRRKRDDSYDDYDEEGGISGWKLGLVFGVIVVCFAMLYPTLFHPMLMGFLGRSPPSSPSINQQRPPIHPAMGGGSGQRHPGGGADVHPAMRMAQAQAESQSGGSKGMFTWMLPVYTIGVVLFLLYTLFKSKGKKSKRKKRNYFDSEDDDDESESETKYGGKFGKKKLEGLQKRLRETESAMSKILEQLESVQAGANPVDLDAADKRSEQLEEDPSVKEAVGLTETNEQYIKDLEVALKEFQSLSKEYDKAKMKKLKRKDSSSDEDEEDEEENSSELSEIEEEEEEVKPVKKSKSSSQSVGKRKNRPKSTSEEEDEGEEESRKVAEDAEEEGIDIDSEIREHAEKEKKDKNVRRRRPKKT

000000000000000000000000000000000000000000000000000000000000000000000000000000000000000000000000000000000000000000000000000000000000000000000000000000000000000000000000000000000000000000000000000000000000000000000000000000000000000000000000000000000000000000000000000000000000000000000000000000000000000000000000000000000000000000000000000000000000000000000000000000000000000000

111111111111111111111111111111111111111111111111111111111111111111111111111111111111111111111111111111111111111111111111111111111111111111111111111111111111111111111111111111111111111111111111111111111111111111111111111111111111111111111111111111111111111111111111111111111111111111111111111111111111111111111111111111111111111111111111111111111111111111111111111111111111111111

000000000000000000000000000000000000000000000000000000000000000000000000000000000000000000000000000000000000000000000000000000000000000000000000000000000000000000000000000000000000000000000000000000000000000000000000000000000000000000000000000000000000000000000000000000000000000000000000000000000000000000000000000000000000000000000000000000000000000000000000000000000000000000

000000000000000000000000000000000000000000000000000000000000000000000000000000000000000000000000000000000000000000000000000000000000000000000000000000000000000000000000000000000000000000000000000000000000000000000000000000000000000000000000000000000000000000000000000000000000000000000000000000000000000000000000000000000000000000000000000000000000000000000000000000000000000000

000000000000000000000000000000000000000000000000000000000000000000000000000000000000000000000000000000000000000000000000000000000000000000000000000000000000000000000000000000000000000000000000000000000000000000000000000000000000000000000000000000000000000000000000000000000000000000000000000000000000000000000000000000000000000000000000000000000000000000000000000000000000000000

>DP00546

MRRRGEIDMATEGDVELELETETSGPERPPEKPRKHDSGAADLERVTDYAEEKEIQSSNLETAMSVIGDRRSREQKAKQEREKELAKVTIKKEDLELIMTEMEISRAAAERSLREHMGNVVEALIALTN

000000000000000000000000000000000000000000000000000000000000000000000000000000000000000000000000000000000000000000000000000000000

111111111111111111111111111111111111111111111111111111111111111111111111111111111111111111111111111111111111111111111111111111111

000000000000000000000000000000000000000000000000000000000000000000000000000000000000000000000000000000000000000000000000000000000

000000000000000000000000000000000000000000000000000000000000000000000000000000000000000000000000000000000000000000000000000000000

000000000000000000000000000000000000000000000000000000000000000000000000000000000000000000000000000000000000000000000000000000000

>DP01465

MSGGFELQPRDGGPRVALAPGETVIGRGPLLGITDKRVSRRHAILEVAGGQLRIKPIHTNPCFYQSSEKSQLLPLKPNLWCYLNPGDSFSLLVDKYIFRILSIPSEVEMQCTLRNSQVLDEDNILNETPKSPVINLPHETTGASQLEGSTEIAKTQMTPTNSVSFLGENRDCNKQQPILAERKRILPTWMLAEHLSDQNLSVPAISGGNVIQGSGKEEICKDKSQLNTTQQGRRQLISSGSSENTSAEQDTGEECKNTDQEESTISSKEMPQSFSAITLSNTEMNNIKTNAQRNKLPIEELGKVSKHKIATKRTPHKEDEAMSCSENCSSAQGDSLQDESQGSHSESSSNPSNPETLHAKATDSVLQGSEGNKVKRTSCMYGANCYRKNPVHFQHFSHPGDSDYGGVQIVGQDETDDRPECPYGPSCYRKNPQHKIEYRHNTLPVRNVLDEDNDNVGQPNEYDLNDSFLDDEEEDYEPTDEDSDWEPGKEDEEKEDVEELLKEAKRFMKRK

0000000000000000000000000000000000000000000000000000000000000000000000000000000000000000000000000000000000000000000000000000000000000000000000000000000000000000000000000000000000000000000000000000000000000000000000000000000000000000000000000000000000000000000000000000000000000000000000000000000000000000000000000000000000000000000000000000000000000000000000000000000000000000000000000000000000000000000000000000000000000000000000000000000000000000000000000000000000000000000000000000000000000000000000000000000

0000000000000000000000000000000000000000000000000000000000000000000000000000000000000000000000000000000000000000000000000000000000000000000000000000000000000000000000000000000000000000000000000000000000000000000000000000000000000000000000000000000000000000000000000000000000000000000000000000000000000000000000000000000000000000000000000000000000000000000000000000000000000000000000000000000000000000000000000000000000000000000000000000000000000000011111111111111111111111111111111111111111111111111111111111111

0000000000000000000000000000000000000000000000000000000000000000000000000000000000000000000000000000000000000000000000000000000000000000000000000000000000000000000000000000000000000000000000000000000000000000000000000000000000000000000000000000000000000000000000000000000000000000000000000000000000000000000000000000000000000000000000000000000000000000000000000000000000000000000000000000000000000000000000000000000000000000000000000000000000000000000000000000000000000000000000000000000000000000000000000000000

0000000000000000000000000000000000000000000000000000000000000000000000000000000000000000000000000000000000000000000000000000000000000000000000000000000000000000000000000000000000000000000000000000000000000000000000000000000000000000000000000000000000000000000000000000000000000000000000000000000000000000000000000000000000000000000000000000000000000000000000000000000000000000000000000000000000000000000000000000000000000000000000000000000000000000000000000000000000000000000000000000000000000000000000000000000

0000000000000000000000000000000000000000000000000000000000000000000000000000000000000000000000000000000000000000000000000000000000000000000000000000000000000000000000000000000000000000000000000000000000000000000000000000000000000000000000000000000000000000000000000000000000000000000000000000000000000000000000000000000000000000000000000000000000000000000000000000000000000000000000000000000000000000000000000000000000000000000000000000000000000000000000000000000000000000000000000000000000000000000000000000000

>DP00674

MPVISTQTSPVPAPRTRKNKQTQASYPVSIKTSVERGQRAKRKVQRDARPRNYKIAGIHDGLQTLAQAALPAHGWGRQDPRHKSRNLGILLDYPLGWIGDVTTHTPLVGPLVAGAVVRPVCQIVRLLEDGVNWATGWFGVHLFVVCLLSLACPCSGARVTDPDTNTTILTNCCQRNQVIYCSPSTCLHEPGCVICADECWVPANPYISHPSNWTGTDSFLADHIDFVMGALVTCDALDIGELCGACVLVGDWLVRHWLIHIDLNETGTCYLEVPTGIDPGFLGFIGWMAGKVEAVIFLTKLASQVPYAIATMFSSVHYLAVGALIYYASRGKWYQLLLALMLYIEATSGNPIRVPTGCSIAEFCSPLMIPCPCHSYLSENVSEVICYSPKWTRPVTLEYNNSISWYPYTIPGARGCMVKFKNNTWGCCRIRNVPSYCTMGTDAVWNDTRNTYEACGVTPWLTTAWHNGSALKLAILQYPGSKEMFKPHNWMSGHLYFEGSDTPIVYFYDPVNSTLLPPERWARLPGTPPVVRGSWLQVPQGFYSDVKDLATGLITKDKAWKNYQVLYSATGALSLTGVTTKAVVLILLGLCGSKYLILAYLCYLSLCFGRASGYPLRPVLPSQSYLQAGWDVLSKAQVAPFALIFFICCYLRCRLRYAALLGFVPMAAGLPLTFFVAAAAAQPDYDWWVRLLVAGLVLWAGRDRGPRIALLVGPWPLVALLTLLHLATPASAFDTEIIGGLTIPPVVALVVMSRFGFFAHLLPRCALVNSYLWQRWENWFWNVTLRPERFLLVLVCFPGATYDTLVTFCVCHVALLCLTSSAASFFGTDSRVRAHRMLVRLGKCHAWYSHYVLKFFLLVFGENGVFFYKHLHGDVLPNDFASKLPLQEPFFPFEGKARVYRNEGRRLACGDTVDGLPVVARLGDLVFAGLAMPPDGWAITAPFTLQCLSERGTLSAMAVVMTGIDPRTWTGTIFRLGSLATSYMGFVCDNVLYTAHHGSKGRRLAHPTGSIHPITVDAANDQDIYQPPCGAGSLTRCSCGETKGYLVTRLGSLVEVNKSDDPYWCVCGALPMAVAKGSSGAPILCSSGHVIGMFTAARNSGGSVSQIRVRPLVCAGYHPQYTAHATLDTKPTVPNEYSVQILIAPTGSGKSTKLPLSYMQEKYEVLVLNPSVATTASMPKYMHATYGVNPNCYFNGKCTNTGASLTYSTYGMYLTGACSRNYDVIICDECHATDATTVLGIGKVLTEAPSKNVRLVVLATATPPGVIPTPHANITEIQLTDEGTIPFHGKKIKEENLKKGRHLIFEATKKHCDELANELARKGITAVSYYRGCDISKIPEGDCVVVATDALCTGYTGDFDSVYDCSLMVEGTCHVDLDPTFTMGVRVCGVSAIVKGQRRGRTGRGRAGIYYYVDGSCTPSGMVPECNIVEAFDAAKAWYGLSSTEAQTILDTYRTQPGLPAIGANLDEWADLFSMVNPEPSFVNTAKRTADNYVLLTAAQLQLCHQYGYAAPNDAPRWQGARLGKKPCGVLWRLDGADACPGPEPSEVTRYQMCFTEVNTSGTAALAVGVGVAMAYLAIDTFGATCVRRCWSITSVPTGATVAPVVDEEEIVEECASFIPLEAMVAAIDKLKSTITTTSPFTLETALEKLNTFLGPHAATILAIIEYCCGLVTLPDNPFASCVFAFIAGITTPLPHKIKMFLSLFGGAIASKLTDARGALAFMMAGAAGTALGTWTSVGFVFDMLGGYAAASSTACLTFKCLMGEWPTMDQLAGLVYSAFNPAAGVVGVLSACAMFALTTAGPDHWPNRLLTMLARSNTVCNEYFIATRDIRRKILGILEASTPWSVISACIRWLHTPTEDDCGLIAWGLEIWQYVCNFFVICFNVLKAGVQSMVNIPGCPFYSCQKGYKGPWIGSGMLQARCPCGAELIFSVENGFAKLYKGPRTCSNYWRGAVPVNARLCGSARPDPTDWTSLVVNYGVRDYCKYEKLGDHIFVTAVSSPNVCFTQVPPTLRAAVAVDGVQVQCYLGEPKTPWTTSACCYGPDGKGKTVKLPFRVDGHTPGVRMQLNLRDALETNDCNSINNTPSDEAAVSALVFKQELRRTNQLLEAISAGVDTTKLPAPSIEEVVVRKRQFRARTGSLTLPPPPRSVPGVSCPESLQRSDPLEGPSNLPSSPPVLQLAMPMPLLGAGECNPFTAIGCAMTETGGGPDDLPSYPPKKEVSEWSDGSWSTTTTASSYVTGPPYPKIRGKDSTQSAPAKRPTKKKLGKSEFSCSMSYTWTDVISFKTASKVLSATRAITSGFLKQRSLVYVTEPRDAELRKQKVTINRQPLFPPSYHKQVRLAKEKASKVVGVMWDYDEVAAHTPSKSAKSHITGLRGTDVRSGAARKAVLDLQKCVEAGEIPSHYRQTVIVPKEEVFVKTPQKPTKKPPRLISYPHLEMRCVEKMYYGQVAPDVVKAVMGDAYGFVDPRTRVKRLLSMWSPDAVGATCDTVCFDSTITPEDIMVETDIYSAAKLSDQHRAGIHTIARQLYAGGPMIAYDGREIGYRRCRSSGVYTTSSSNSLTCWLKVNAAAEQAGMKNPRFLICGDDCTVIWKSAGADADKQAMRVFASWMKVMGAPQDCVPQPKYSLEELTSCSSNVTSGITKSGKPYYFLTRDPRIPLGRCSAEGLGYNPSAAWIGYLIHHYPCLWVSRVLAVHFMEQMLFEDKLPETVTFDWYGKNYTVPVEDLPSIIAGVHGIEAFSVVRYTNAEILRVSQSLTDMTMPPLRAWRKKARAVLASAKRRGGAHAKLARFLLWHATSRPLPDLDKTSVARYTTFNYCDVYSPEGDVFVTPQRRLQKFLVKYLAVIVFALGLIAVGLAIS

00000000000000000000000000000000000000000000000000000000000000000000000000000000000000000000000000000000000000000000000000000000000000000000000000000000000000000000000000000000000000000000000000000000000000000000000000000000000000000000000000000000000000000000000000000000000000000000000000000000000000000000000000000000000000000000000000000000000000000000000000000000000000000000000000000000000000000000000000000000000000000000000000000000000000000000000000000000000000000000000000000000000000000000000000000000000000000000000000000000000000000000000000000000000000000000000000000000000000000000000000000000000000000000000000000000000000000000000000000000000000000000000000000000000000000000000000000000000000000000000000000000000000000000000000000000000000000000000000000000000000000000000000000000000000000000000000000000000000000000000000000000000000000000000000000000000000000000000000000000000000000000000000000000000000000000000000000000000000000000000000000000000000000000000000000000000000000000000000000000000000000000000000000000000000000000000000000000000000000000000000000000000000000000000000000000000000000000000000000000000000000000000000000000000000000000000000000000000000000000000000000000000000000000000000000000000000000000000000000000000000000000000000000000000000000000000000000000000000000000000000000000000000000000000000000000000000000000000000000000000000000000000000000000000000000000000000000000000000000000000000000000000000000000000000000000000000000000000000000000000000000000000000000000000000000000000000000000000000000000000000000000000000000000000000000000000000000000000000000000000000000000000000000000000000000000000000000000000000000000000000000000000000000000000000000000000000000000000000000000000000000000000000000000000000000000000000000000000000000000000000000000000000000000000000000000000000000000000000000000000000000000000000000000000000000000000000000000000000000000000000000000000000000000000000000000000000000000000000000000000000000000000000000000000000000000000000000000000000000000000000000000000000000000000000000000000000000000000000000000000000000000000000000000000000000000000000000000000000000000000000000000000000000000000000000000000000000000000000000000000000000000000000000000000000000000000000000000000000000000000000000000000000000000000000000000000000000000000000000000000000000000000000000000000000000000000000000000000000000000000000000000000000000000000000000000000000000000000000000000000000000000000000000000000000000000000000000000000000000000000000000000000000000000000000000000000000000000000000000000000000000000000000000000000000000000000000000000000000000000000000000000000000000000000000000000000000000000000000000000000000000000000000000000000000000000000000000000000000000000000000000000000000000000000000000000000000000000000000000000000000000000000000000000000000000000000000000000

01111111111111111111111111111111111111111111111111111111111111111111111111111111111111111111111111111111111111111111111111111111111111111111111111111111111000000000000000000000000000000000000000000000000000000000000000000000000000000000000000000000000000000000000000000000000000000000000000000000000000000000000000000000000000000000000000000000000000000000000000000000000000000000000000000000000000000000000000000000000000000000000000000000000000000000000000000000000000000000000000000000000000000000000000000000000000000000000000000000000000000000000000000000000000000000000000000000000000000000000000000000000000000000000000000000000000000000000000000000000000000000000000000000000000000000000000000000000000000000000000000000000000000000000000000000000000000000000000000000000000000000000000000000000000000000000000000000000000000000000000000000000000000000000000000000000000000000000000000000000000000000000000000000000000000000000000000000000000000000000000000000000000000000000000000000000000000000000000000000000000000000000000000000000000000000000000000000000000000000000000000000000000000000000000000000000000000000000000000000000000000000000000000000000000000000000000000000000000000000000000000000000000000000000000000000000000000000000000000000000000000000000000000000000000000000000000000000000000000000000000000000000000000000000000000000000000000000000000000000000000000000000000000000000000000000000000000000000000000000000000000000000000000000000000000000000000000000000000000000000000000000000000000000000000000000000000000000000000000000000000000000000000000000000000000000000000000000000000000000000000000000000000000000000000000000000000000000000000000000000000000000000000000000000000000000000000000000000000000000000000000000000000000000000000000000000000000000000000000000000000000000000000000000000000000000000000000000000000000000000000000000000000000000000000000000000000000000000000000000000000000000000000000000000000000000000000000000000000000000000000000000000000000000000000000000000000000000000000000000000000000000000000000000000000000000000000000000000000000000000000000000000000000000000000000000000000000000000000000000000000000000000000000000000000000000000000000000000000000000000000000000000000000000000000000000000000000000000000000000000000000000000000000000000000000000000000000000000000000000000000000000000000000000000000000000000000000000000000000000000000000000000000000000000000000000000000000000000000000000000000000000000000000000000000000000000000000000000000000000000000000000000000000000000000000000000000000000000000000000000000000000000000000000000000000000000000000000000000000000000000000000000000000000000000000000000000000000000000000000000000000000000000000000000000000000000000000000000000000000000000000000000000000000000000000000000000000000000000000000000000000000000000000000000000000000000000000000

00000000000000000000000000000000000000000000000000000000000000000000000000000000000000000000000000000000000000000000000000000000000000000000000000000000000000000000000000000000000000000000000000000000000000000000000000000000000000000000000000000000000000000000000000000000000000000000000000000000000000000000000000000000000000000000000000000000000000000000000000000000000000000000000000000000000000000000000000000000000000000000000000000000000000000000000000000000000000000000000000000000000000000000000000000000000000000000000000000000000000000000000000000000000000000000000000000000000000000000000000000000000000000000000000000000000000000000000000000000000000000000000000000000000000000000000000000000000000000000000000000000000000000000000000000000000000000000000000000000000000000000000000000000000000000000000000000000000000000000000000000000000000000000000000000000000000000000000000000000000000000000000000000000000000000000000000000000000000000000000000000000000000000000000000000000000000000000000000000000000000000000000000000000000000000000000000000000000000000000000000000000000000000000000000000000000000000000000000000000000000000000000000000000000000000000000000000000000000000000000000000000000000000000000000000000000000000000000000000000000000000000000000000000000000000000000000000000000000000000000000000000000000000000000000000000000000000000000000000000000000000000000000000000000000000000000000000000000000000000000000000000000000000000000000000000000000000000000000000000000000000000000000000000000000000000000000000000000000000000000000000000000000000000000000000000000000000000000000000000000000000000000000000000000000000000000000000000000000000000000000000000000000000000000000000000000000000000000000000000000000000000000000000000000000000000000000000000000000000000000000000000000000000000000000000000000000000000000000000000000000000000000000000000000000000000000000000000000000000000000000000000000000000000000000000000000000000000000000000000000000000000000000000000000000000000000000000000000000000000000000000000000000000000000000000000000000000000000000000000000000000000000000000000000000000000000000000000000000000000000000000000000000000000000000000000000000000000000000000000000000000000000000000000000000000000000000000000000000000000000000000000000000000000000000000000000000000000000000000000000000000000000000000000000000000000000000000000000000000000000000000000000000000000000000000000000000000000000000000000000000000000000000000000000000000000000000000000000000000000000000000000000000000000000000000000000000000000000000000000000000000000000000000000000000000000000000000000000000000000000000000000000000000000000000000000000000000000000000000000000000000000000000000000000000000000000000000000000000000000000000000000000000000000000000000000000000000000000000000000000000000000000000000000000000000000000000000000

00000000000000000000000000000000000000000000000000000000000000000000000000000000000000000000000000000000000000000000000000000000000000000000000000000000000000000000000000000000000000000000000000000000000000000000000000000000000000000000000000000000000000000000000000000000000000000000000000000000000000000000000000000000000000000000000000000000000000000000000000000000000000000000000000000000000000000000000000000000000000000000000000000000000000000000000000000000000000000000000000000000000000000000000000000000000000000000000000000000000000000000000000000000000000000000000000000000000000000000000000000000000000000000000000000000000000000000000000000000000000000000000000000000000000000000000000000000000000000000000000000000000000000000000000000000000000000000000000000000000000000000000000000000000000000000000000000000000000000000000000000000000000000000000000000000000000000000000000000000000000000000000000000000000000000000000000000000000000000000000000000000000000000000000000000000000000000000000000000000000000000000000000000000000000000000000000000000000000000000000000000000000000000000000000000000000000000000000000000000000000000000000000000000000000000000000000000000000000000000000000000000000000000000000000000000000000000000000000000000000000000000000000000000000000000000000000000000000000000000000000000000000000000000000000000000000000000000000000000000000000000000000000000000000000000000000000000000000000000000000000000000000000000000000000000000000000000000000000000000000000000000000000000000000000000000000000000000000000000000000000000000000000000000000000000000000000000000000000000000000000000000000000000000000000000000000000000000000000000000000000000000000000000000000000000000000000000000000000000000000000000000000000000000000000000000000000000000000000000000000000000000000000000000000000000000000000000000000000000000000000000000000000000000000000000000000000000000000000000000000000000000000000000000000000000000000000000000000000000000000000000000000000000000000000000000000000000000000000000000000000000000000000000000000000000000000000000000000000000000000000000000000000000000000000000000000000000000000000000000000000000000000000000000000000000000000000000000000000000000000000000000000000000000000000000000000000000000000000000000000000000000000000000000000000000000000000000000000000000000000000000000000000000000000000000000000000000000000000000000000000000000000000000000000000000000000000000000000000000000000000000000000000000000000000000000000000000000000000000000000000000000000000000000000000000000000000000000000000000000000000000000000000000000000000000000000000000000000000000000000000000000000000000000000000000000000000000000000000000000000000000000000000000000000000000000000000000000000000000000000000000000000000000000000000000000000000000000000000000000000000000000000000000000000000000000000000000000

00000000000000000000000000000000000000000000000000000000000000000000000000000000000000000000000000000000000000000000000000000000000000000000000000000000000000000000000000000000000000000000000000000000000000000000000000000000000000000000000000000000000000000000000000000000000000000000000000000000000000000000000000000000000000000000000000000000000000000000000000000000000000000000000000000000000000000000000000000000000000000000000000000000000000000000000000000000000000000000000000000000000000000000000000000000000000000000000000000000000000000000000000000000000000000000000000000000000000000000000000000000000000000000000000000000000000000000000000000000000000000000000000000000000000000000000000000000000000000000000000000000000000000000000000000000000000000000000000000000000000000000000000000000000000000000000000000000000000000000000000000000000000000000000000000000000000000000000000000000000000000000000000000000000000000000000000000000000000000000000000000000000000000000000000000000000000000000000000000000000000000000000000000000000000000000000000000000000000000000000000000000000000000000000000000000000000000000000000000000000000000000000000000000000000000000000000000000000000000000000000000000000000000000000000000000000000000000000000000000000000000000000000000000000000000000000000000000000000000000000000000000000000000000000000000000000000000000000000000000000000000000000000000000000000000000000000000000000000000000000000000000000000000000000000000000000000000000000000000000000000000000000000000000000000000000000000000000000000000000000000000000000000000000000000000000000000000000000000000000000000000000000000000000000000000000000000000000000000000000000000000000000000000000000000000000000000000000000000000000000000000000000000000000000000000000000000000000000000000000000000000000000000000000000000000000000000000000000000000000000000000000000000000000000000000000000000000000000000000000000000000000000000000000000000000000000000000000000000000000000000000000000000000000000000000000000000000000000000000000000000000000000000000000000000000000000000000000000000000000000000000000000000000000000000000000000000000000000000000000000000000000000000000000000000000000000000000000000000000000000000000000000000000000000000000000000000000000000000000000000000000000000000000000000000000000000000000000000000000000000000000000000000000000000000000000000000000000000000000000000000000000000000000000000000000000000000000000000000000000000000000000000000000000000000000000000000000000000000000000000000000000000000000000000000000000000000000000000000000000000000000000000000000000000000000000000000000000000000000000000000000000000000000000000000000000000000000000000000000000000000000000000000000000000000000000000000000000000000000000000000000000000000000000000000000000000000000000000000000000000000000000000000000000000000000000000000000000

>DP01590

MSDYSTGGPPPGPPPPAGGGGGAGGAGGGPPPGPPGAGDRGGGGPGGGGPGGGSAGGPSQPPGGGGPGIRKDAFADAVQRARQIAAKIGGDAATTVNNSTPDFGFGGQKRQLEDGDQPESKKLASQGDSISSQLGPIHPPPRTSMTEEYRVPDGMVGLIIGRGGEQINKIQQDSGCKVQISPDSGGLPERSVSLTGAPESVQKAKMMLDDIVSRGRGGPPGQFHDNANGGQNGTVQEIMIPAGKAGLVIGKGGETIKQLQERAGVKMILIQDGSQNTNVDKPLRIIGDPYKVQQACEMVMDILRERDQGGFGDRNEYGSRIGGGIDVPVPRHSVGVVIGRSGEMIKKIQNDAGVRIQFKQDDGTGPEKIAHIMGPPDRCEHAARIINDLLQSLRSGPPGPPGGPGMPPGGRGRGRGQGNWGPPGGEMTFSIPTHKCGLVIGRGGENVKAINQQTGAFVEISRQLPPNGDPNFKLFIIRGSPQQIDHAKQLIEEKIEGPLCPVGPGPGGPGPAGPMGPFNPGPFNQGPPGAPPHAGGPPPHQYPPQGWGNTYPQWQPPAPHDPSKAAAAAADPNAAWAAYYSHYYQQPPGPVPGPAPAPAAPPAQGEPPQPPPTGQSDYTKAWEEYYKKIGQQPQQPGAPPQQDYTKAWEEYYKKQAQVATGGGPGAPPGSQPDYSAAWAEYYRQQAAYYGQTPGPGGPQPPPTQQGQQQAQ

000000000000000000000000000000000000000000000000000000000000000000000000000000000000000000000000000000000000000000000000000000000000000000000000000000000000000000000000000000000000000000000000000000000000000000000000000000000000000000000000000000000000000000000000000000000000000000000000000000000000000000000000000000000000000000000000000000000000000000000000000000000000000000000000000000000000000000000000000000000000000000000000000000000000000000000000000000000000000000000000000000000000000000000000000000000000000000000000000000000000000000000000000000000000000000000000000000000000000000000000000000000000000000000000000000000000000000000000000000000000000000000000000000000000000000000000000000000000000

000000000000000000000000000000000000000000000000000000000000000000000000000000000000000000000000000000000000000000000000000000000111111111111111111111111111111111111111111111111111111111111111111111111111111111111111110000000000000000000000000000000000000000000000000000000000000000000000000000000000000000000000000000000000000000000000000000000000000000000000000000000000000000000000000000000000000000000000000000000000000000000000000000000000000000000000000000000000000000000000000000000000000000000000000000000000000000000000000000000000000000000000000000000000000000000000000000000000000000000000000000000000000000000000000000000000000000000000000000000000000000000000000000000000000000000000000000000000000

000000000000000000000000000000000000000000000000000000000000000000000000000000000000000000000000000000000000000000000000000000000000000000000000000000000000000000000000000000000000000000000000000000000000000000000000000000000000000000000000000000000000000000000000000000000000000000000000000000000000000000000000000000000000000000000000000000000000000000000000000000000000000000000000000000000000000000000000000000000000000000000000000000000000000000000000000000000000000000000000000000000000000000000000000000000000000000000000000000000000000000000000000000000000000000000000000000000000000000000000000000000000000000000000000000000000000000000000000000000000000000000000000000000000000000000000000000000000000

000000000000000000000000000000000000000000000000000000000000000000000000000000000000000000000000000000000000000000000000000000000000000000000000000000000000000000000000000000000000000000000000000000000000000000000000000000000000000000000000000000000000000000000000000000000000000000000000000000000000000000000000000000000000000000000000000000000000000000000000000000000000000000000000000000000000000000000000000000000000000000000000000000000000000000000000000000000000000000000000000000000000000000000000000000000000000000000000000000000000000000000000000000000000000000000000000000000000000000000000000000000000000000000000000000000000000000000000000000000000000000000000000000000000000000000000000000000000000

000000000000000000000000000000000000000000000000000000000000000000000000000000000000000000000000000000000000000000000000000000000000000000000000000000000000000000000000000000000000000000000000000000000000000000000000000000000000000000000000000000000000000000000000000000000000000000000000000000000000000000000000000000000000000000000000000000000000000000000000000000000000000000000000000000000000000000000000000000000000000000000000000000000000000000000000000000000000000000000000000000000000000000000000000000000000000000000000000000000000000000000000000000000000000000000000000000000000000000000000000000000000000000000000000000000000000000000000000000000000000000000000000000000000000000000000000000000000000

>DP01088

MAKSKEDITYATSQARLSEDEAVRVAYEHGSPLEGGKIADSQPVDLFSSAHNMPKSGQTTMDSNTSDQSQMQRDTQEGGSKEFTTGAPG

00000000000000000000000000000000000000000000000000000000000000000000000000000000000000000

11111111111111111111111111111111111111111111111111111111111111111111111111111111111111111

00000000000000000000000000000000000000000000000000000000000000000000000000000000000000000

00000000000000000000000000000000000000000000000000000000000000000000000000000000000000000

00000000000000000000000000000000000000000000000000000000000000000000000000000000000000000

>DP00472

MAMRELVEAECGGANPLMKLAGHFTQDKALRQEGLRPGPWPPGAPASEAASKPLGVASEDELVAEFLQDQNAPLVSRAPQTFKMDDLLAEMQQIEQSNFRQAPQRAPGVADLALSENWAQEFLAAGDAVDVTQDYNETDWSQEFISEVTDPLSVSPARWAEEYLEQSEEKLWLGEPEGTATDRWYDEYHPEEDLQHTASDFVAKVDDPKLANSEFLKFVRQIGEGQVSLESGAGSGRAQAEQWAAEFIQQQGTSDAWVDQFTRPVNTSALDMEFERAKSAIESDVDFWDKLQAELEEMAKRDAEAHPWLSDYDDLTSATYDKGYQFEEENPLRDHPQPFEEGLRRLQEGDLPNAVLLFEAAVQQDPKHMEAWQYLGTTQAENEQELLAISALRRCLELKPDNQTALMALAVSFTNESLQRQACETLRDWLRYTPAYAHLVTPAEEGAGGAGLGPSKRILGSLLSDSLFLEVKELFLAAVRLDPTSIDPDVQCGLGVLFNLSGEYDKAVDCFTAALSVRPNDYLLWNKLGATLANGNQSEEAVAAYRRALELQPGYIRSRYNLGISCINLGAHREAVEHFLEALNMQRKSRGPRGEGGAMSENIWSTLRLALSMLGQSDAYGAADARDLSTLLTMFGLPQ

000000000000000000000000000000000000000000000000000000000000000000000000000000000000000000000000000000000000000000000000000000000000000000000000000000000000000000000000000000000000000000000000000000000000000000000000000000000000000000000000000000000000000000000000000000000000000000000000000000000000000000000000000000000000000000000000000000000000000000000000000000000000000000000000000000000000000000000000000000000000000000000000000000000000000000000000000000000000000000000000000000000000000000000000000000000000000000000000000000000000000000000000000000000000000000000000000000000000000000000000000000000000000000000000000000000000000

111111111111111111111111111111111111111111111111111111111111111111111111111111111111111111111111111111111111111111111111111111111111111111111111111111111111111111111111111111111111111111111111111111111111111111111111111111111111111111111111111111111111111111111111111111111111111111111111111111111111111111111111111111111111000000000000000000000000000000000000000000000000000000000000000000000000000000000000000000000000000000000000000000000000000000000000000000000000000000000000000000000000000000000000000000000000000000000000000000000000000000000000000000000000000000000000000000000000000000000000000000000000000000000000000000000000000

000000000000000000000000000000000000000000000000000000000000000000000000000000000000000000000000000000000000000000000000000000000000000000000000000000000000000000000000000000000000000000000000000000000000000000000000000000000000000000000000000000000000000000000000000000000000000000000000000000000000000000000000000000000000000000000000000000000000000000000000000000000000000000000000000000000000000000000000000000000000000000000000000000000000000000000000000000000000000000000000000000000000000000000000000000000000000000000000000000000000000000000000000000000000000000000000000000000000000000000000000000000000000000000000000000000000000

000000000000000000000000000000000000000000000000000000000000000000000000000000000000000000000000000000000000000000000000000000000000000000000000000000000000000000000000000000000000000000000000000000000000000000000000000000000000000000000000000000000000000000000000000000000000000000000000000000000000000000000000000000000000000000000000000000000000000000000000000000000000000000000000000000000000000000000000000000000000000000000000000000000000000000000000000000000000000000000000000000000000000000000000000000000000000000000000000000000000000000000000000000000000000000000000000000000000000000000000000000000000000000000000000000000000000

000000000000000000000000000000000000000000000000000000000000000000000000000000000000000000000000000000000000000000000000000000000000000000000000000000000000000000000000000000000000000000000000000000000000000000000000000000000000000000000000000000000000000000000000000000000000000000000000000000000000000000000000000000000000000000000000000000000000000000000000000000000000000000000000000000000000000000000000000000000000000000000000000000000000000000000000000000000000000000000000000000000000000000000000000000000000000000000000000000000000000000000000000000000000000000000000000000000000000000000000000000000000000000000000000000000000000

>DP01651

MWIQVRTMDGRQTHTVDSLSRLTKVEELRRKIQELFHVEPGLQRLFYRGKQMEDGHTLFDYEVRLNDTIQLLVRQSLVLPHSTKERDSELSDTDSGCCLGQSESDKSSTHGEAAAETDSRPADEDMWDETELGLYKVNEYVDARDTNMGAWFEAQVVRVTRKAPSRDEPCSSTSRPALEEDVIYHVKYDDYPENGVVQMNSRDVRARARTIIKWQDLEVGQVVMLNYNPDNPKERGFWYDAEISRKRETRTARELYANVVLGDDSLNDCRIIFVDEVFKIERPGEGSPMVDNPMRRKSGPSCKHCKDDVNRLCRVCACHLCGGRQDPDKQLMCDECDMAFHIYCLDPPLSSVPSEDEWYCPECRNDASEVVLAGERLRESKKKAKMASATSSSQRDWGKGMACVGRTKECTIVPSNHYGPIPGIPVGTMWRFRVQVSESGVHRPHVAGIHGRSNDGAYSLVLAGGYEDDVDHGNFFTYTGSGGRDLSGNKRTAEQSCDQKLTNTNRALALNCFAPINDQEGAEAKDWRSGKPVRVVRNVKGGKNSKYAPAEGNRYDGIYKVVKYWPEKGKSGFLVWRYLLRRDDDEPGPWTKEGKDRIKKLGLTMQYPEGYLEALANREREKENSKREEEEQQEGGFASPRTGKGKWKRKSAGGGPSRAGSPRRTSKKTKVEPYSLTAQQSSLIREDKSNAKLWNEVLASLKDRPASGSPFQLFLSKVEETFQCICCQELVFRPITTVCQHNVCKDCLDRSFRAQVFSCPACRYDLGRSYAMQVNQPLQTVLNQLFPGYGNGR

0000000000000000000000000000000000000000000000000000000000000000000000000000000000000000000000000000000000000000000000000000000000000000000000000000000000000000000000000000000000000000000000000000000000000000000000000000000000000000000000000000000000000000000000000000000000000000000000000000000000000000000000000000000000000000000000000000000000000000000000000000000000000000000000000000000000000000000000000000000000000000000000000000000000000000000000000000000000000000000000000000000000000000000000000000000000000000000000000000000000000000000000000000000000000000000000000000000000000000000000000000000000000000000000000000000000000000000000000000000000000000000000000000000000000000000000000000000000000000000000000000000000000000000000000000000000000000000000000000000000000000000000000

0000000000000000000000000000000000000000000000000000000000000000000000000000000000000000000000000000000000000000000000000000000000000000000000000000000000000000000000000000000000000000000000000000000000000000000000000000000000000000000000000000000000000000000000000000000000000000000000000000000000000000000000000000000000000000000000000000000000000000000000000000000000000000000000000000000000000000000000000000000000000000000000000000000000000000000000000000000000000000000000000000000000000000000000000000000000000000000000000000000000000000000000000000000000000000000000000000000000000000000000000000000000000000000000000000000000000000000000000000000000000000000000000000000000000000000000000000000000000000000000000000000000000000000000000000000000000000000000000000000000000000000000000

0000000000000000000000000000000000000000000000000000000000000000000000000000000000000000000000000000000000000000000000000000000000000000000000000000000000000000000000000000000000000000000000000000000000000000000000000000000000000000000000000000000000000000000000000000000000000000000001111111111111111000000000000000000000000000000000000000000000000000000000000000000000000000000000000000000000000000000000000000000000000000000000000000000000000000000000000000000000000000000000000000000000000000000000000000000000000000000000000000000000000000000000000000000000000000000000000000000000000000000000000000000000000000000000000000000000000000000000000000000000000000000000000000000000000000000000000000000000000000000000000000000000000000000000000000000000000000000000000000000000000000000000000

0000000000000000000000000000000000000000000000000000000000000000000000000000000000000000000000000000000000000000000000000000000000000000000000000000000000000000000000000000000000000000000000000000000000000000000000000000000000000000000000000000000000000000000000000000000000000000000000000000000000000000000000000000000000000000000000000000000000000000000000000000000000000000000000000000000000000000000000000000000000000000000000000000000000000000000000000000000000000000000000000000000000000000000000000000000000000000000000000000000000000000000000000000000000000000000000000000000000000000000000000000000000000000000000000000000000000000000000000000000000000000000000000000000000000000000000000000000000000000000000000000000000000000000000000000000000000000000000000000000000000000000000000

0000000000000000000000000000000000000000000000000000000000000000000000000000000000000000000000000000000000000000000000000000000000000000000000000000000000000000000000000000000000000000000000000000000000000000000000000000000000000000000000000000000000000000000000000000000000000000000000000000000000000000000000000000000000000000000000000000000000000000000000000000000000000000000000000000000000000000000000000000000000000000000000000000000000000000000000000000000000000000000000000000000000000000000000000000000000000000000000000000000000000000000000000000000000000000000000000000000000000000000000000000000000000000000000000000000000000000000000000000000000000000000000000000000000000000000000000000000000000000000000000000000000000000000000000000000000000000000000000000000000000000000000000

>DP01104

MPPLLAPLLCLALLPALAARGPRCSQPGETCLNGGKCEAANGTEACVCGGAFVGPRCQDPNPCLSTPCKNAGTCHVVDRRGVADYACSCALGFSGPLCLTPLDNACLTNPCRNGGTCDLLTLTEYKCRCPPGWSGKSCQQADPCASNPCANGGQCLPFEASYICHCPPSFHGPTCRQDVNECGQKPGLCRHGGTCHNEVGSYRCVCRATHTGPNCERPYVPCSPSPCQNGGTCRPTGDVTHECACLPGFTGQNCEENIDDCPGNNCKNGGACVDGVNTYNCRCPPEWTGQYCTEDVDECQLMPNACQNGGTCHNTHGGYNCVCVNGWTGEDCSENIDDCASAACFHGATCHDRVASFYCECPHGRTGLLCHLNDACISNPCNEGSNCDTNPVNGKAICTCPSGYTGPACSQDVDECSLGANPCEHAGKCINTLGSFECQCLQGYTGPRCEIDVNECVSNPCQNDATCLDQIGEFQCICMPGYEGVHCEVNTDECASSPCLHNGRCLDKINEFQCECPTGFTGHLCQYDVDECASTPCKNGAKCLDGPNTYTCVCTEGYTGTHCEVDIDECDPDPCHYGSCKDGVATFTCLCRPGYTGHHCETNINECSSQPCRHGGTCQDRDNAYLCFCLKGTTGPNCEINLDDCASSPCDSGTCLDKIDGYECACEPGYTGSMCNINIDECAGNPCHNGGTCEDGINGFTCRCPEGYHDPTCLSEVNECNSNPCVHGACRDSLNGYKCDCDPGWSGTNCDINNNECESNPCVNGGTCKDMTSGYVCTCREGFSGPNCQTNINECASNPCLNQGTCIDDVAGYKCNCLLPYTGATCEVVLAPCAPSPCRNGGECRQSEDYESFSCVCPTGWQGQTCEVDINECVLSPCRHGASCQNTHGGYRCHCQAGYSGRNCETDIDDCRPNPCHNGGSCTDGINTAFCDCLPGFRGTFCEEDINECASDPCRNGANCTDCVDSYTCTCPAGFSGIHCENNTPDCTESSCFNGGTCVDGINSFTCLCPPGFTGSYCQHDVNECDSQPCLHGGTCQDGCGSYRCTCPQGYTGPNCQNLVHWCDSSPCKNGGKCWQTHTQYRCECPSGWTGLYCDVPSVSCEVAAQRQGVDVARLCQHGGLCVDAGNTHHCRCQAGYTGSYCEDLVDECSPSPCQNGATCTDYLGGYSCKCVAGYHGVNCSEEIDECLSHPCQNGGTCLDLPNTYKCSCPRGTQGVHCEINVDDCNPPVDPVSRSPKCFNNGTCVDQVGGYSCTCPPGFVGERCEGDVNECLSNPCDARGTQNCVQRVNDFHCECRAGHTGRRCESVINGCKGKPCKNGGTCAVASNTARGFICKCPAGFEGATCENDARTCGSLRCLNGGTCISGPRSPTCLCLGPFTGPECQFPASSPCLGGNPCYNQGTCEPTSESPFYRCLCPAKFNGLLCHILDYSFGGGAGRDIPPPLIEEACELPECQEDAGNKVCSLQCNNHACGWDGGDCSLNFNDPWKNCTQSLQCWKYFSDGHCDSQCNSAGCLFDGFDCQRAEGQCNPLYDQYCKDHFSDGHCDQGCNSAECEWDGLDCAEHVPERLAAGTLVVVVLMPPEQLRNSSFHFLRELSRVLHTNVVFKRDAHGQQMIFPYYGREEELRKHPIKRAAEGWAAPDALLGQVKASLLPGGSEGGRRRRELDPMDVRGSIVYLEIDNRQCVQASSQCFQSATDVAAFLGALASLGSLNIPYKIEAVQSETVEPPPPAQLHFMYVAAAAFVLLFFVGCGVLLSRKRRRQHGQLWFPEGFKVSEASKKKRREPLGEDSVGLKPLKNASDGALMDDNQNEWGDEDLETKKFRFEEPVVLPDLDDQTDHRQWTQQHLDAADLRMSAMAPTPPQGEVDADCMDVNVRGPDGFTPLMIASCSGGGLETGNSEEEEDAPAVISDFIYQGASLHNQTDRTGETALHLAARYSRSDAAKRLLEASADANIQDNMGRTPLHAAVSADAQGVFQILIRNRATDLDARMHDGTTPLILAARLAVEGMLEDLINSHADVNAVDDLGKSALHWAAAVNNVDAAVVLLKNGANKDMQNNREETPLFLAAREGSYETAKVLLDHFANRDITDHMDRLPRDIAQERMHHDIVRLLDEYNLVRSPQLHGAPLGGTPTLSPPLCSPNGYLGSLKPGVQGKKVRKPSSKGLACGSKEAKDLKARRKKSQDGKGCLLDSSGMLSPVDSLESPHGYLSDVASPPLLPSPFQQSPSVPLNHLPGMPDTHLGIGHLNVAAKPEMAALGGGGRLAFETGPPRLSHLPVASGTSTVLGSSSGGALNFTVGGSTSLNGQCEWLSRLQSGMVPNQYNPLRGSVAPGPLSTQAPSLQHGMVGPLHSSLAASALSQMMSYQGLPSTRLATQPHLVQTQQVQPQNLQMQQQNLQPANIQQQQSLQPPPPPPQPHLGVSSAASGHLGRSFLSGEPSQADVQPLGPSSLAVHTILPQESPALPTSLPSSLVPPVTAAQFLTPPSQHSYSSPVDNTPSHQLQVPEHPFLTPSPESPDQWSSSSPHSNVSDWSEGVSSPPTSMQSQIARIPEAFK

00000000000000000000000000000000000000000000000000000000000000000000000000000000000000000000000000000000000000000000000000000000000000000000000000000000000000000000000000000000000000000000000000000000000000000000000000000000000000000000000000000000000000000000000000000000000000000000000000000000000000000000000000000000000000000000000000000000000000000000000000000000000000000000000000000000000000000000000000000000000000000000000000000000000000000000000000000000000000000000000000000000000000000000000000000000000000000000000000000000000000000000000000000000000000000000000000000000000000000000000000000000000000000000000000000000000000000000000000000000000000000000000000000000000000000000000000000000000000000000000000000000000000000000000000000000000000000000000000000000000000000000000000000000000000000000000000000000000000000000000000000000000000000000000000000000000000000000000000000000000000000000000000000000000000000000000000000000000000000000000000000000000000000000000000000000000000000000000000000000000000000000000000000000000000000000000000000000000000000000000000000000000000000000000000000000000000000000000000000000000000000000000000000000000000000000000000000000000000000000000000000000000000000000000000000000000000000000000000000000000000000000000000000000000000000000000000000000000000000000000000000000000000000000000000000000000000000000000000000000000000000000000000000000000000000000000000000000000000000000000000000000000000000000000000000000000000000000000000000000000000000000000000000000000000000000000000000000000000000000000000000000000000000000000000000000000000000000000000000000000000000000000000000000000000000000000000000000000000000000000000000000000000000000000000000000000000000000000000000000000000000000000000000000000000000000000000000000000000000000000000000000000000000000000000000000000000000000000000000000000000000000000000000000000000000000000000000000000000000000000000000000000000000000000000000000000000000000000000000000000000000000000000000000000000000000000000000000000000000000000000000000000000000000000000000000000000000000000000000000000000000000000000000000000000000000000000000000000000000000000000000000000000000000000000000000000000000000000000000000000000000000000000000000000000000000000000000000000000000000000000000000000000000000000000000000000000000000000000000000000000000000000000000000000000000000000000000000000000000000000000000000000000000000000000000000000000000000000000000000000000000000000000000000000000000000000000000000000000000000000000000000000000000000000000000000000

00000000000000000000000000000000000000000000000000000000000000000000000000000000000000000000000000000000000000000000000000000000000000000000000000000000000000000000000000000000000000000000000000000000000000000000000000000000000000000000000000000000000000000000000000000000000000000000000000000000000000000000000000000000000000000000000000000000000000000000000000000000000000000000000000000000000000000000000000000000000000000000000000000000000000000000000000000000000000000000000000000000000000000000000000000000000000000000000000000000000000000000000000000000000000000000000000000000000000000000000000000000000000000000000000000000000000000000000000000000000000000000000000000000000000000000000000000000000000000000000000000000000000000000000000000000000000000000000000000000000000000000000000000000000000000000000000000000000000000000000000000000000000000000000000000000000000000000000000000000000000000000000000000000000000000000000000000000000000000000000000000000000000000000000000000000000000000000000000000000000000000000000000000000000000000000000000000000000000000000000000000000000000000000000000000000000000000000000000000000000000000000000000000000000000000000000000000000000000000000000000000000000000000000000000000000000000000000000000000000000000000000000000000000000000000000000000000000000000000000000000000000000000000000000000000000000000000000000000000000000000000000000000000000000000000000000000000000000000000000000000000000000000000000000000000000000000000000000000000000000000000000000000000000000000000000000000000000000000000000000000000000000000000000000000000000000000000000000000000000000000000000000000000000000000000000000000000000000000000000000000000000000000000000000000000000000000000000000000000000000000000000000000000000000000000000000000000000000000000000000000000000000000000000000000000000000000000000000000000000000000000000000000000000000000000000000000000000000000000000000000000000000000000000000000000000000000000000000000000000000000000000000000000000000000000000000000000000000000000000000000000000000000000000000000000000000000000000000000000000000000000000000000000000000000000000000000000000000000000000000000000000000000000000000000000000000000000000000000000000000000000000000000000000000000000000000000000000000000000000000000000000000000000000000000000000000000000000000000000000000000000000000000000000000000000000000000000000000000000000000000000000000000000000000000000000000000000000000000000000000000000000000000000000000000000000000000000000000000000000000000000000000000000000000000000000000

00000000000000000000000000000000000000000000000000000000000000000000000000000000000000000000000000000000000000000000000000000000000000000000000000000000000000000000000000000000000000000000000000000000000000000000000000000000000000000000000000000000000000000000000000000000000000000000000000000000000000000000000000000000000000000000000000000000000000000000000000000000000000000000000000000000000000000000000000000000000000000000000000000000000000000000000000000000000000000000000000000000000000000000000000000000000000000000000000000000000000000000000000000000000000000000000000000000000000000000000000000000000000000000000000000000000000000000000000000000000000000000000000000000000000000000000000000000000000000000000000000000000000000000000000000000000000000000000000000000000000000000000000000000000000000000000000000000000000000000000000000000000000000000000000000000000000000000000000000000000000000000000000000000000000000000000000000000000000000000000000000000000000000000000000000000000000000000000000000000000000000000000000000000000000000000000000000000000000000000000000000000000000000000000000000000000000000000000000000000000000000000000000000000000000000000000000000000000000000000000000000000000000000000000000000000000000000000000000000000000000000000000000000000000000000000000000000000000000000000000000000000000000000000000000000000000000000000000000000000000000000000000000000000000000000000000000000000000000000000000000000000000000000000000000000000000000000000000000000000000000000000000000000000000000000000000000000000000000000000000000000000000000000000000000000000000000000000000000000000000000000000000000000000000000000000000000000000000000000000000000000000000000000000000000000000000000000000000000000000000000000000000000000111111111111111111111111111111111111111111111111111111111111111111111111111111111111111111111111111111111111111111111111111111111110000000000000000000000000000000000000000000000000000000000000000000000000000000000000000000000000000000000000000000000000000000000000000000000000000000000000000000000000000000000000000000000000000000000000000000000000000000000000000000000000000000000000000000000000000000000000000000000000000000000000000000000000000000000000000000000000000000000000000000000000000000000000000000000000000000000000000000000000000000000000000000000000000000000000000000000000000000000000000000000000000000000000000000000000000000000000000000000000000000000000000000000000000000000000000000000000000000000000000000000000000000000000000000000000000000000000000000000000000000000000000000

00000000000000000000000000000000000000000000000000000000000000000000000000000000000000000000000000000000000000000000000000000000000000000000000000000000000000000000000000000000000000000000000000000000000000000000000000000000000000000000000000000000000000000000000000000000000000000000000000000000000000000000000000000000000000000000000000000000000000000000000000000000000000000000000000000000000000000000000000000000000000000000000000000000000000000000000000000000000000000000000000000000000000000000000000000000000000000000000000000000000000000000000000000000000000000000000000000000000000000000000000000000000000000000000000000000000000000000000000000000000000000000000000000000000000000000000000000000000000000000000000000000000000000000000000000000000000000000000000000000000000000000000000000000000000000000000000000000000000000000000000000000000000000000000000000000000000000000000000000000000000000000000000000000000000000000000000000000000000000000000000000000000000000000000000000000000000000000000000000000000000000000000000000000000000000000000000000000000000000000000000000000000000000000000000000000000000000000000000000000000000000000000000000000000000000000000000000000000000000000000000000000000000000000000000000000000000000000000000000000000000000000000000000000000000000000000000000000000000000000000000000000000000000000000000000000000000000000000000000000000000000000000000000000000000000000000000000000000000000000000000000000000000000000000000000000000000000000000000000000000000000000000000000000000000000000000000000000000000000000000000000000000000000000000000000000000000000000000000000000000000000000000000000000000000000000000000000000000000000000000000000000000000000000000000000000000000000000000000000000000000000000000000000000000000000000000000000000000000000000000000000000000000000000000000000000000000000000000000000000000000000000000000000000000000000000000000000000000000000000000000000000000000000000000000000000000000000000000000000000000000000000000000000000000000000000000000000000000000000000000000000000000000000000000000000000000000000000000000000000000000000000000000000000000000000000000000000000000000000000000000000000000000000000000000000000000000000000000000000000000000000000000000000000000000000000000000000000000000000000000000000000000000000000000000000000000000000000000000000000000000000000000000000000000000000000000000000000000000000000000000000000000000000000000000000000000000000000000000000000000000000000000000000000000000000000000000000000000000000000000000000000000000000000000000000000000

00000000000000000000000000000000000000000000000000000000000000000000000000000000000000000000000000000000000000000000000000000000000000000000000000000000000000000000000000000000000000000000000000000000000000000000000000000000000000000000000000000000000000000000000000000000000000000000000000000000000000000000000000000000000000000000000000000000000000000000000000000000000000000000000000000000000000000000000000000000000000000000000000000000000000000000000000000000000000000000000000000000000000000000000000000000000000000000000000000000000000000000000000000000000000000000000000000000000000000000000000000000000000000000000000000000000000000000000000000000000000000000000000000000000000000000000000000000000000000000000000000000000000000000000000000000000000000000000000000000000000000000000000000000000000000000000000000000000000000000000000000000000000000000000000000000000000000000000000000000000000000000000000000000000000000000000000000000000000000000000000000000000000000000000000000000000000000000000000000000000000000000000000000000000000000000000000000000000000000000000000000000000000000000000000000000000000000000000000000000000000000000000000000000000000000000000000000000000000000000000000000000000000000000000000000000000000000000000000000000000000000000000000000000000000000000000000000000000000000000000000000000000000000000000000000000000000000000000000000000000000000000000000000000000000000000000000000000000000000000000000000000000000000000000000000000000000000000000000000000000000000000000000000000000000000000000000000000000000000000000000000000000000000000000000000000000000000000000000000000000000000000000000000000000000000000000000000000000000000000000000000000000000000000000000000000000000000000000000000000000000000000000000000000000000000000000000000000000000000000000000000000000000000000000000000000000000000000000000000000000000000000000000000000000000000000000000000000000000000000000000000000000000000000000000000000000000000000000000000000000000000000000000000000000000000000000000000000000000000000000000000000000000000000000000000000000000000000000000000000000000000000000000000000000000000000000000000000000000000000000000000000000000000000000000000000000000000000000000000000000000000000000000000000000000000000000000000000000000000000000000000000000000000000000000000000000000000000000000000000000000000000000000000000000000000000000000000000000000000000000000000000000000000000000000000000000000000000000000000000000000000000000000000000000000000000000000000000000000000000000000000000000000000000000000000000000

>DP01158

MPEPAKSAPAPKKGSKKAVTKAQKKDGKKRKRSRKESYSIYVYKVLKQVHPDTGISSKAMGIMNSFVNDIFERIAGEASRLAHYNKRSTITSREIQTAVRLLLPGELAKHAVSEGTKAVTKYTSAK

000000000000000000000000000000000000000000000000000000000000000000000000000000000000000000000000000000000000000000000000000000

000000000000000000000000000000000000000000000000000000000000000000000000000000000000000000000000000000000000000000000000000000

111111111111111111111111111111111111110000000000000000000000000000000000000000000000000000000000000000000000000000000000000000

000000000000000000000000000000000000000000000000000000000000000000000000000000000000000000000000000000000000000000000000000000

000000000000000000000000000000000000000000000000000000000000000000000000000000000000000000000000000000000000000000000000000000

>DP01175

MGDWGFLEKLLDQVQEHSTVVGKIWLTVLFIFRILILGLAGESVWGDEQSDFECNTAQPGCTNVCYDQAFPISHIRYWVLQFLFVSTPTLIYLGHVIYLSRREERLRQKEGELRALPSKDLHVERALAAIEHQMAKISVAEDGRLRIRGALMGTYVVSVLCKSVLEAGFLYGQWRLYGWTMEPVFVCQRAPCPHIVDCYVSRPTEKTIFIIFMLVVGVISLVLNLLELVHLLCRCVSREIKARRDHDARPAQGSASDPYPEQVFFYLPMGEGPSSPPCPTYNGLSSTEQNWANLTTEERLTSSRPPPFVNTAPQGGRKSPSRPNSSASKKQYV

000000000000000000000000000000000000000000000000000000000000000000000000000000000000000000000000000000000000000000000000000000000000000000000000000000000000000000000000000000000000000000000000000000000000000000000000000000000000000000000000000000000000000000000000000000000000000000000000000000000000000000000000000000000000000000000

000000000000000000000000000000000000000000000000000000000000000000000000000000000000000000000000000000000000000000000000000000000000000000000000000000000000000000000000000000000000000000000000000000000000000000000000000000000000000000000000000000000000000000000000000000000000000000000000000000000000000000000000000000000000000000000

000000000000000000000000000000000000000000000000000000000000000000000000000000000000000000000000000000000000000000000000000000000000000000000000000000000000000000000000000000000000000000000000000000000000000000000000000000000000000011111111111111111111111111111111111111111111111111111111111111111111111111111111111111111111111111111

000000000000000000000000000000000000000000000000000000000000000000000000000000000000000000000000000000000000000000000000000000000000000000000000000000000000000000000000000000000000000000000000000000000000000000000000000000000000000000000000000000000000000000000000000000000000000000000000000000000000000000000000000000000000000000000

000000000000000000000000000000000000000000000000000000000000000000000000000000000000000000000000000000000000000000000000000000000000000000000000000000000000000000000000000000000000000000000000000000000000000000000000000000000000000000000000000000000000000000000000000000000000000000000000000000000000000000000000000000000000000000000

>DP00013

MEMFQGLLLLLLLSMGGTWASKEPLRPRCRPINATLAVEKEGCPVCITVNTTICAGYCPTMTRVLQGVLPALPQVVCNYRDVRFESIRLPGCPRGVNPVVSYAVALSCQCALCRRSTTDCGGPKDHPLTCDDPRFQDSSSSKAPPPSLPSPSRLPGPSDTPILPQ

000000000000000000000000000000000000000000000000000000000000000000000000000000000000000000000000000000000000000000000000000000000000000000000000000000000000000000000

000000000000000000000000000000000000000000000000000000000000000000000000000000000000000000000000000000000000000000000000000000000000000000000000000000000000000000000

000000000000000000000000000000000000000000000000000000000000000000000000000000000000000000000000000000000000000000000000000000000000000000000011111111111111111111111

000000000000000000000000000000000000000000000000000000000000000000000000000000000000000000000000000000000000000000000000000000000000000000000000000000000000000000000

000000000000000000000000000000000000000000000000000000000000000000000000000000000000000000000000000000000000000000000000000000000000000000000000000000000000000000000

>DP02822

MNPGFDLSRRNPQEDFELIQRIGSGTYGDVYKARNVNTGELAAIKVIKLEPGEDFAVVQQEIIMMKDCKHPNIVAYFGSYLRRDKLWICMEFCGGGSLQDIYHVTGPLSELQIAYVSRETLQGLYYLHSKGKMHRDIKGANILLTDNGHVKLADFGVSAQITATIAKRKSFIGTPYWMAPEVAAVERKGGYNQLCDLWAVGITAIELAELQPPMFDLHPMRALFLMTKSNFQPPKLKDKMKWSNSFHHFVKMALTKNPKKRPTAEKLLQHPFVTQHLTRSLAIELLDKVNNPDHSTYHDFDDDDPEPLVAVPHRIHSTSRNVREEKTRSEITFGQVKFDPPLRKETEPHHELPDSDGFLDSSEEIYYTARSNLDLQLEYGQGHQGGYFLGANKSLLKSVEEELHQRGHVAHLEDDEGDDDESKHSTLKAKIPPPLPPKPKSIFIPQEMHSTEDENQGTIKRCPMSGSPAKPSQVPPRPPPPRLPPHKPVALGNGMSSFQLNGERDGSLCQQQNEHRGTNLSRKEKKDVPKPISNGLPPTPKVHMGACFSKVFNGCPLKIHCASSWINPDTRDQYLIFGAEEGIYTLNLNELHETSMEQLFPRRCTWLYVMNNCLLSISGKASQLYSHNLPGLFDYARQMQKLPVAIPAHKLPDRILPRKFSVSAKIPETKWCQKCCVVRNPYTGHKYLCGALQTSIVLLEWVEPMQKFMLIKHIDFPIPCPLRMFEMLVVPEQEYPLVCVGVSRGRDFNQVVRFETVNPNSTSSWFTESDTPQTNVTHVTQLERDTILVCLDCCIKIVNLQGRLKSSRKLSSELTFDFQIESIVCLQDSVLAFWKHGMQGRSFRSNEVTQEISDSTRIFRLLGSDRVVVLESRPTDNPTANSNLYILAGHENSY

000000000000000000000000000000000000000000000000000000000000000000000000000000000000000000000000000000000000000000000000000000000000000000000000000000000000000000000000000000000000000000000000000000000000000000000000000000000000000000000000000000000000000000000000000000000000000000000000000000000000000000000000000000000000000000000000000000000000000000000000000000000000000000000000000000000000000000000000000000000000000000000000000000000000000000000000000000000000000000000000000000000000000000000000000000000000000000000000000000000000000000000000000000000000000000000000000000000000000000000000000000000000000000000000000000000000000000000000000000000000000000000000000000000000000000000000000000000000000000000000000000000000000000000000000000000000000000000000000000000000000000000000000000000000000000000000000000000000000000000000000000000000000000000000000000000000000000000000000000

000000000000000000000000000000000000000000000000000000000000000000000000000000000000000000000000000000000000000000000000000000000000000000000000000000000000000000000000000000000000000000000000000000000000000000000000000000000000000000000000000000000000000000000000000000000000000000000000000000000000000000000000000000000000000000000000000000000000000000000000000000000000000000000000000000000000000000000000000000000000000000000000000000000000000000000000000000000000000000000000000000000000000000000000000000000000000000000000000000000000000000000000000000000000000000000000000000000000000000000000000000000000000000000000000000000000000000000000000000000000000000000000000000000000000000000000000000000000000000000000000000000000000000000000000000000000000000000000000000000000000000000000000000000000000000000000000000000000000000000000000000000000000000000000000000000000000000000000000000

000000000000000000000000000000000000000000000000000000000000000000000000000000000000000000000000000000000000000000000000000000000000000000000000000000000000000000000000000000000000000000000000000000000000000000000000000000000000000000000000000000000000000000000000000000000000000000000000000000000000000000000000001111111111111111111111111111111111111111111111111111111111111111110000000000000000000000000000000000000000000000000000000000000000000000000000000000000000000000000000000000000000000000000000000000000000000000000000000000000000000000000000000000000000000000000000000000000000000000000000000000000000000000000000000000000000000000000000000000000000000000000000000000000000000000000000000000000000000000000000000000000000000000000000000000000000000000000000000000000000000000000000000000000000000000000000000000000000000000000000000000000000000000000000000000000000000000000000000000

000000000000000000000000000000000000000000000000000000000000000000000000000000000000000000000000000000000000000000000000000000000000000000000000000000000000000000000000000000000000000000000000000000000000000000000000000000000000000000000000000000000000000000000000000000000000000000000000000000000000000000000000000000000000000000000000000000000000000000000000000000000000000000000000000000000000000000000000000000000000000000000000000000000000000000000000000000000000000000000000000000000000000000000000000000000000000000000000000000000000000000000000000000000000000000000000000000000000000000000000000000000000000000000000000000000000000000000000000000000000000000000000000000000000000000000000000000000000000000000000000000000000000000000000000000000000000000000000000000000000000000000000000000000000000000000000000000000000000000000000000000000000000000000000000000000000000000000000000000

000000000000000000000000000000000000000000000000000000000000000000000000000000000000000000000000000000000000000000000000000000000000000000000000000000000000000000000000000000000000000000000000000000000000000000000000000000000000000000000000000000000000000000000000000000000000000000000000000000000000000000000000000000000000000000000000000000000000000000000000000000000000000000000000000000000000000000000000000000000000000000000000000000000000000000000000000000000000000000000000000000000000000000000000000000000000000000000000000000000000000000000000000000000000000000000000000000000000000000000000000000000000000000000000000000000000000000000000000000000000000000000000000000000000000000000000000000000000000000000000000000000000000000000000000000000000000000000000000000000000000000000000000000000000000000000000000000000000000000000000000000000000000000000000000000000000000000000000000000

>DP00144

MLCEDQHMSVENTPQKGSGSLNSSASSISIDVKPTMQSWAQEVRAEFGHSDEASSSLNSSAASCGSLAKKETADGNLESKDGEGREMAFEFLDGVNEVKFERLVKEEKLKTPYKRRHSFTPPSNENSRSNSPNSSNSSANGDAAAPKGGNNPHSRNSKKSGNFRAHKEEKRVRHNSYTSSTSSSSSYTEADPAILSRRQKQIDYGKNTAAYERYVEMVPKDERTRDHPRTPNKYGKYSRRAFDGLVKIWRKSLHIYDPPTQARDTAKDSNSDSDSD

000000000000000000000000000000000000000000000000000000000000000000000000000000000000000000000000000000000000000000000000000000000000000000000000000000000000000000000000000000000000000000000000000000000000000000000000000000000000000000000000000000000000000000000000000000000000

000000000000000000000000000000000000000000000000000000000000000000000000000000000000000000000000000000000000000000000000000000000000000000000000000000000000000000000000000000000000000000000000000000000000000000000000000000000000000000000000000000000000000000000000000000000000

111111111111111111111111111000000000000000000000000000000000000000000000000000000000000000000000000000000000000000000000000000000000000000000000000000000000000000000000000000000000000000000000000000000000000000000000000000000000000000000000000000000000000000000000000000000000

000000000000000000000000000000000000000000000000000000000000000000000000000000000000000000000000000000000000000000000000000000000000000000000000000000000000000000000000000000000000000000000000000000000000000000000000000000000000000000000000000000000000000000000000000000000000

000000000000000000000000000000000000000000000000000000000000000000000000000000000000000000000000000000000000000000000000000000000000000000000000000000000000000000000000000000000000000000000000000000000000000000000000000000000000000000000000000000000000000000000000000000000000

>DP00669

MAPLVGLFLIWAGASVFQQLHPVNGGDIPDPGSKPTPPGMADELPTETYDLPPEIYTTTFLPRTIYPQEEMPYDDKPFPSLLSKANDLNAVFEGPACAFPFTYKGKKYYMCTRKNSVLLWCSLDTEYQGNWKFCTERDEPECVFPFIYRKKSYESCTRVHSFFWRRWCSLTSNYDRDKAWKYC

000000000000000000000000000000000000000000000000000000000000000000000000000000000000000000000000000000000000000000000000000000000000000000000000000000000000000000000000000000000000000

000000000000000000000000000000000000000000000000000000000000000000000000000000000000000000000000000000000000000000000000000000000000000000000000000000000000000000000000000000000000000

000000000000000000000000011111111111111111111111111111111111111111111111111111111111111111111111000000000000000000000000000000000000000000000000000000000000000000000000000000000000000

000000000000000000000000000000000000000000000000000000000000000000000000000000000000000000000000000000000000000000000000000000000000000000000000000000000000000000000000000000000000000

000000000000000000000000000000000000000000000000000000000000000000000000000000000000000000000000000000000000000000000000000000000000000000000000000000000000000000000000000000000000000

>DP03279

MAALGVQSINWQTAFNRQAHHTDKFSSQELILRRGQNFQVLMIMNKGLGSNERLEFIVSTGPYPSESAMTKAVFPLSNGSSGGWSAVLQASNGNTLTISISSPASAPIGRYTMALQIFSQGGISSVKLGTFILLFNPWLNVDSVFMGNHAEREEYVQEDAGIIFVGSTNRIGMIGWNFGQFEEDILSICLSILDRSLNFRRDAATDVASRNDPKYVGRVLSAMINSNDDNGVLAGNWSGTYTGGRDPRSWNGSVEILKNWKKSGFSPVRYGQCWVFAGTLNTALRSLGIPSRVITNFNSAHDTDRNLSVDVYYDPMGNPLDKGSDSVWNFHVWNEGWFVRSDLGPSYGGWQVLDATPQERSQGVFQCGPASVIGVREGDVQLNFDMPFIFAEVNADRITWLYDNTTGKQWKNSVNSHTIGRYISTKAVGSNARMDVTDKYKYPEGSDQERQVFQKALGKLKPNTPFAATSSMGLETEEQEPSIIGKLKVAGMLAVGKEVNLVLLLKNLSRDTKTVTVNMTAWTIIYNGTLVHEVWKDSATMSLDPEEEAEHPIKISYAQYEKYLKSDNMIRITAVCKVPDESEVVVERDIILDNPTLTLEVLNEARVRKPVNVQMLFSNPLDEPVRDCVLMVEGSGLLLGNLKIDVPTLGPKEGSRVRFDILPSRSGTKQLLADFSCNKFPAIKAMLSIDVAE

000000000000000000000000000000000000000000000000000000000000000000000000000000000000000000000000000000000000000000000000000000000000000000000000000000000000000000000000000000000000000000000000000000000000000000000000000000000000000000000000000000000000000000000000000000000000000000000000000000000000000000000000000000000000000000000000000000000000000000000000000000000000000000000000000000000000000000000000000000000000000000000000000000000000000000000000000000000000000000000000000000000000000000000000000000000000000000000000000000000000000000000000000000000000000000000000000000000000000000000000000000000000000000000000000000000000000000000000000000000000000000000000000000000000000000000

000000000000000000000000000000000000000000000000000000000000000000000000000000000000000000000000000000000000000000000000000000000000000000000000000000000000000000000000000000000000000000000000000000000000000000000000000000000000000000000000000000000000000000000000000000000000000000000000000000000000000000000000000000000000000000000000000000000000000000000000000000000000000000000000000000000000000000000000000000000000000000000000000000000000000000000000000000000000000000000000000000000000000000000000000000000000000000000000000000000000000000000000000000000000000000000000000000000000000000000000000000000000000000000000000000000000000000000000000000000000000000000000000000000000000000000

000000000000000000000000000000000000000000000000000000000000000000000000000000000000000000000000000000000000000000000000000000000000000000000000000000000000000000000000000000000000000000000000000000000000000000000000000000000000000000000000000000000000000000000000000000000000000000000000000000000000000000000000000000000000000000000000000000000000000000000000000000000000000000000000000000000000000000000000000000000000000000000000000000000000000000000000000000000001111100000000000000000000000000000000000000000000000000000000000000000000000000000000000000000000000000000000000000000000000000000000000000000000000000000000000000000000000000000000000000000000000000000000000000000000000000000

000000000000000000000000000000000000000000000000000000000000000000000000000000000000000000000000000000000000000000000000000000000000000000000000000000000000000000000000000000000000000000000000000000000000000000000000000000000000000000000000000000000000000000000000000000000000000000000000000000000000000000000000000000000000000000000000000000000000000000000000000000000000000000000000000000000000000000000000000000000000000000000000000000000000000000000000000000000000000000000000000000000000000000000000000000000000000000000000000000000000000000000000000000000000000000000000000000000000000000000000000000000000000000000000000000000000000000000000000000000000000000000000000000000000000000000

000000000000000000000000000000000000000000000000000000000000000000000000000000000000000000000000000000000000000000000000000000000000000000000000000000000000000000000000000000000000000000000000000000000000000000000000000000000000000000000000000000000000000000000000000000000000000000000000000000000000000000000000000000000000000000000000000000000000000000000000000000000000000000000000000000000000000000000000000000000000000000000000000000000000000000000000000000000000000000000000000000000000000000000000000000000000000000000000000000000000000000000000000000000000000000000000000000000000000000000000000000000000000000000000000000000000000000000000000000000000000000000000000000000000000000000

>DP00012

MQRSPLEKASVVSKLFFSWTRPILRKGYRQRLELSDIYQIPSVDSADNLSEKLEREWDRELASKKNPKLINALRRCFFWRFMFYGIFLYLGEVTKAVQPLLLGRIIASYDPDNKEERSIAIYLGIGLCLLFIVRTLLLHPAIFGLHHIGMQMRIAMFSLIYKKTLKLSSRVLDKISIGQLVSLLSNNLNKFDEGLALAHFVWIAPLQVALLMGLIWELLQASAFCGLGFLIVLALFQAGLGRMMMKYRDQRAGKISERLVITSEMIENIQSVKAYCWEEAMEKMIENLRQTELKLTRKAAYVRYFNSSAFFFSGFFVVFLSVLPYALIKGIILRKIFTTISFCIVLRMAVTRQFPWAVQTWYDSLGAINKIQDFLQKQEYKTLEYNLTTTEVVMENVTAFWEEGFGELFEKAKQNNNNRKTSNGDDSLFFSNFSLLGTPVLKDINFKIERGQLLAVAGSTGAGKTSLLMVIMGELEPSEGKIKHSGRISFCSQFSWIMPGTIKENIIFGVSYDEYRYRSVIKACQLEEDISKFAEKDNIVLGEGGITLSGGQRARISLARAVYKDADLYLLDSPFGYLDVLTEKEIFESCVCKLMANKTRILVTSKMEHLKKADKILILHEGSSYFYGTFSELQNLQPDFSSKLMGCDSFDQFSAERRNSILTETLHRFSLEGDAPVSWTETKKQSFKQTGEFGEKRKNSILNPINSIRKFSIVQKTPLQMNGIEEDSDEPLERRLSLVPDSEQGEAILPRISVISTGPTLQARRRQSVLNLMTHSVNQGQNIHRKTTASTRKVSLAPQANLTELDIYSRRLSQETGLEISEEINEEDLKECFFDDMESIPAVTTWNTYLRYITVHKSLIFVLIWCLVIFLAEVAASLVVLWLLGNTPLQDKGNSTHSRNNSYAVIITSTSSYYVFYIYVGVADTLLAMGFFRGLPLVHTLITVSKILHHKMLHSVLQAPMSTLNTLKAGGILNRFSKDIAILDDLLPLTIFDFIQLLLIVIGAIAVVAVLQPYIFVATVPVIVAFIMLRAYFLQTSQQLKQLESEGRSPIFTHLVTSLKGLWTLRAFGRQPYFETLFHKALNLHTANWFLYLSTLRWFQMRIEMIFVIFFIAVTFISILTTGEGEGRVGIILTLAMNIMSTLQWAVNSSIDVDSLMRSVSRVFKFIDMPTEGKPTKSTKPYKNGQLSKVMIIENSHVKKDDIWPSGGQMTVKDLTAKYTEGGNAILENISFSISPGQRVGLLGRTGSGKSTLLSAFLRLLNTEGEIQIDGVSWDSITLQQWRKAFGVIPQKVFIFSGTFRKNLDPYEQWSDQEIWKVADEVGLRSVIEQFPGKLDFVLVDGGCVLSHGHKQLMCLARSVLSKAKILLLDEPSAHLDPVTYQIIRRTLKQAFADCTVILCEHRIEAMLECQQFLVIEENKVRQYDSIQKLLNERSLFRQAISPSDRVKLFPHRNSSKCKSKPQIAALKEETEEEVQDTRL

0000000000000000000000000000000000000000000000000000000000000000000000000000000000000000000000000000000000000000000000000000000000000000000000000000000000000000000000000000000000000000000000000000000000000000000000000000000000000000000000000000000000000000000000000000000000000000000000000000000000000000000000000000000000000000000000000000000000000000000000000000000000000000000000000000000000000000000000000000000000000000000000000000000000000000000000000000000000000000000000000000000000000000000000000000000000000000000000000000000000000000000000000000000000000000000000000000000000000000000000000000000000000000000000000000000000000000000000000000000000000000000000000000000000000000000000000000000000000000000000000000000000000000000000000000000000000000000000000000000000000000000000000000000000000000000000000000000000000000000000000000000000000000000000000000000000000000000000000000000000000000000000000000000000000000000000000000000000000000000000000000000000000000000000000000000000000000000000000000000000000000000000000000000000000000000000000000000000000000000000000000000000000000000000000000000000000000000000000000000000000000000000000000000000000000000000000000000000000000000000000000000000000000000000000000000000000000000000000000000000000000000000000000000000000000000000000000000000000000000000000000000000000000000000000000000000000000000000000000000000000000000000000000000000000000000000000000000000000000000000000000000000000000000000000000000000000000

0000000000000000000000000000000000000000000000000000000000000000000000000000000000000000000000000000000000000000000000000000000000000000000000000000000000000000000000000000000000000000000000000000000000000000000000000000000000000000000000000000000000000000000000000000000000000000000000000000000000000000000000000000000000000000000000000000000000000000000000000000000000000000000000000000000000000000000000000000000000000000000000000000000000000000000000000000000000000000000000000000000000000000000000000000000000000000000000000000000000000000000000000000000000000000000000000000000000000000000000000000000000000000000000000000000000000000000000000000000000000000000000000000000000000000000000000000000000000000000000000000000000000000000000000000000000000000000000000000000000000000000000000000000000000000000000000000000000000000000000000000000000000000000000000000000000000000000000000000000000000000000000000000000000000000000000000000000000000000000000000000000000000000000000000000000000000000000000000000000000000000000000000000000000000000000000000000000000000000000000000000000000000000000000000000000000000000000000000000000000000000000000000000000000000000000000000000000000000000000000000000000000000000000000000000000000000000000000000000000000000000000000000000000000000000000000000000000000000000000000000000000000000000000000000000000000000000000000000000000000000000000000000000000000000000000000000000000000000000000000000000000000000000000000000000000000000000

0000000000000000000000000000000000000000000000000000000000000000000000000000000000000000000000000000000000000000000000000000000000000000000000000000000000000000000000000000000000000000000000000000000000000000000000000000000000000000000000000000000000000000000000000000000000000000000000000000000000000000000000000000000000000000000000000000000000000000000000000000000000000000000000000000000000000000000000000000000000000000000000000000000000000000000000000000000000000000000000000000000000000000000000000000000000000000000000000000000000000000000000000000000000000000000000000000000000000000000000000000000000000000000000000000000000000000000000000000011111111111111111111111111111111111111111111111111111111111111111111111111111111111111111111111111111111111111111111111111111111111111111111111111111111111111111111111111111111111111111000000000000000000000000000000000000000000000000000000000000000000000000000000000000000000000000000000000000000000000000000000000000000000000000000000000000000000000000000000000000000000000000000000000000000000000000000000000000000000000000000000000000000000000000000000000000000000000000000000000000000000000000000000000000000000000000000000000000000000000000000000000000000000000000000000000000000000000000000000000000000000000000000000000000000000000000000000000000000000000000000000000000000000000000000000000000000000000000000000000000000000000000000000000000000000000000000000000000000000000000000000000000000000000000000000000000000000

0000000000000000000000000000000000000000000000000000000000000000000000000000000000000000000000000000000000000000000000000000000000000000000000000000000000000000000000000000000000000000000000000000000000000000000000000000000000000000000000000000000000000000000000000000000000000000000000000000000000000000000000000000000000000000000000000000000000000000000000000000000000000000000000000000000000000000000000000000000000000000000000000000000000000000000000000000000000000000000000000000000000000000000000000000000000000000000000000000000000000000000000000000000000000000000000000000000000000000000000000000000000000000000000000000000000000000000000000000000000000000000000000000000000000000000000000000000000000000000000000000000000000000000000000000000000000000000000000000000000000000000000000000000000000000000000000000000000000000000000000000000000000000000000000000000000000000000000000000000000000000000000000000000000000000000000000000000000000000000000000000000000000000000000000000000000000000000000000000000000000000000000000000000000000000000000000000000000000000000000000000000000000000000000000000000000000000000000000000000000000000000000000000000000000000000000000000000000000000000000000000000000000000000000000000000000000000000000000000000000000000000000000000000000000000000000000000000000000000000000000000000000000000000000000000000000000000000000000000000000000000000000000000000000000000000000000000000000000000000000000000000000000000000000000000000000000000

0000000000000000000000000000000000000000000000000000000000000000000000000000000000000000000000000000000000000000000000000000000000000000000000000000000000000000000000000000000000000000000000000000000000000000000000000000000000000000000000000000000000000000000000000000000000000000000000000000000000000000000000000000000000000000000000000000000000000000000000000000000000000000000000000000000000000000000000000000000000000000000000000000000000000000000000000000000000000000000000000000000000000000000000000000000000000000000000000000000000000000000000000000000000000000000000000000000000000000000000000000000000000000000000000000000000000000000000000000000000000000000000000000000000000000000000000000000000000000000000000000000000000000000000000000000000000000000000000000000000000000000000000000000000000000000000000000000000000000000000000000000000000000000000000000000000000000000000000000000000000000000000000000000000000000000000000000000000000000000000000000000000000000000000000000000000000000000000000000000000000000000000000000000000000000000000000000000000000000000000000000000000000000000000000000000000000000000000000000000000000000000000000000000000000000000000000000000000000000000000000000000000000000000000000000000000000000000000000000000000000000000000000000000000000000000000000000000000000000000000000000000000000000000000000000000000000000000000000000000000000000000000000000000000000000000000000000000000000000000000000000000000000000000000000000000000000000

>DP00801

MDKVQYLTRSAIRRASTIEMPQQARQNLQNLFINFCLILICLLLICIIVMLL

0000000000000000000000000000000000000000000000000000

0000000000000000000000000000000000000000000000000000

1111111111111111111100000000000000000000000000000000

0000000000000000000000000000000000000000000000000000

0000000000000000000000000000000000000000000000000000

>DP01581

MSDNNGTPEPQVETTSVFRADLLKEMESSTGTAPASTGAENLPAGSALLVVKRGPNAGARFLLDQPTTTAGRHPESDIFLDDVTVSRRHAEFRINEGEFEVVDVGSLNGTYVNREPRNAQVMQTGDEIQIGKFRLVFLAGPAE

00000000000000000000000000000000000000000000000000000000000000000000000000000000000000000000000000000000000000000000000000000000000000000000000

00000000000000000000000000000000000000000000000000000000000000000000000000000000000000000000000000000000000000000000000000000000000000000000000

11111111111111111111111111111111111111110000000000000000000000000000000000000000000000000000000000000000000000000000000000000000000000000000000

00000000000000000000000000000000000000000000000000000000000000000000000000000000000000000000000000000000000000000000000000000000000000000000000

00000000000000000000000000000000000000000000000000000000000000000000000000000000000000000000000000000000000000000000000000000000000000000000000

>DP02812

MAPWSHPSAQLQPVGGDAVSPALMVLLCLGLSLGPRTHVQAGNLSKATLWAEPGSVISRGNSVTIRCQGTLEAQEYRLVKEGSPEPWDTQNPLEPKNKARFSIPSMTEHHAGRYRCYYYSPAGWSEPSDPLELVVTGFYNKPTLSALPSPVVTSGENVTLQCGSRLRFDRFILTEEGDHKLSWTLDSQLTPSGQFQALFPVGPVTPSHRWMLRCYGSRRHILQVWSEPSDLLEIPVSGAADNLSPSQNKSDSGTASHLQDYAVENLIRMGMAGLILVVLGILIFQDWHSQRSPQAAAGR

00000000000000000000000000000000000000000000000000000000000000000000000000000000000000000000000000000000000000000000000000000000000000000000000000000000000000000000000000000000000000000000000000000000000000000000000000000000000000000000000000000000000000000000000000000000000000000000000000000000000

00000000000000000000000000000000000000000000000000000000000000000000000000000000000000000000000000000000000000000000000000000000000000000000000000000000000000000000000000000000000000000000000000000000000000000000000000000000000000000000000000000000000000000000000000000000000000000000000000000000000

00000000000000000000000000000000000000000000000000000000000000000000000000000000000000000000000000000000000000000000000000000000000000000000000000111111111110000000000000000000000000000000000000000000000000000000000000000000000000000000000000000000000000000000000000000000000000000000000000000000000

00000000000000000000000000000000000000000000000000000000000000000000000000000000000000000000000000000000000000000000000000000000000000000000000000000000000000000000000000000000000000000000000000000000000000000000000000000000000000000000000000000000000000000000000000000000000000000000000000000000000

00000000000000000000000000000000000000000000000000000000000000000000000000000000000000000000000000000000000000000000000000000000000000000000000000000000000000000000000000000000000000000000000000000000000000000000000000000000000000000000000000000000000000000000000000000000000000000000000000000000000

>DP00385

MSGRPRTTSFAESCKPVQQPSAFGSMKVSRDKDGSKVTTVVATPGQGPDRPQEVSYTDTKVIGNGSFGVVYQAKLCDSGELVAIKKVLQDKRFKNRELQIMRKLDHCNIVRLRYFFYSSGEKKDEVYLNLVLDYVPETVYRVARHYSRAKQTLPVIYVKLYMYQLFRSLAYIHSFGICHRDIKPQNLLLDPDTAVLKLCDFGSAKQLVRGEPNVSYICSRYYRAPELIFGATDYTSSIDVWSAGCVLAELLLGQPIFPGDSGVDQLVEIIKVLGTPTREQIREMNPNYTEFKFPQIKAHPWTKVFRPRTPPEAIALCSRLLEYTPTARLTPLEACAHSFFDELRDPNVKLPNGRDTPALFNFTTQELSSNPPLATILIPPHARIQAAASTPTNATAASDANTGDRGQTNNAASASASNST

000000000000000000000000000000000000000000000000000000000000000000000000000000000000000000000000000000000000000000000000000000000000000000000000000000000000000000000000000000000000000000000000000000000000000000000000000000000000000000000000000000000000000000000000000000000000000000000000000000000000000000000000000000000000000000000000000000000000000000000000000000000000000000000000000000000000000000000000000000000000

000000000000000000000000000000000000000000000000000000000000000000000000000000000000000000000000000000000000000000000000000000000000000000000000000000000000000000000000000000000000000000000000000000000000000000000000000000000000000000000000000000000000000000000000000000000000000000000000000000000000000000000000000000000000000000000000000000000000000000000000000000000000000000000000000000000000000000000000000000000000

000000000000000000000000000000000000000000000000000000000000000000000000000000000000000000000000000000000000000000000000000000000000000000000000000000000000000000000000000000000000000000000000000000000000000000000000000000000000000000000000000000000000000000000000000000000000000000001111111111111110000000000000000000000000000000000000000000000000000000000000000000000000000000000000000000000000000000000000000000000000

000000000000000000000000000000000000000000000000000000000000000000000000000000000000000000000000000000000000000000000000000000000000000000000000000000000000000000000000000000000000000000000000000000000000000000000000000000000000000000000000000000000000000000000000000000000000000000000000000000000000000000000000000000000000000000000000000000000000000000000000000000000000000000000000000000000000000000000000000000000000

000000000000000000000000000000000000000000000000000000000000000000000000000000000000000000000000000000000000000000000000000000000000000000000000000000000000000000000000000000000000000000000000000000000000000000000000000000000000000000000000000000000000000000000000000000000000000000000000000000000000000000000000000000000000000000000000000000000000000000000000000000000000000000000000000000000000000000000000000000000000

>DP01776

MARSNVPKFGNWEAEENVPYTAYFDKARKTRAPGSKIMNPNDPEYNSDSQSQAPPHPPSSRTKPEQVDTVRRSREHMRSREESELKQFGDAGGSSNEAANKRQGRASQNNSYDNKSPLHKNSYDGTGKSRPKPTNLRADESPEKVTVVPKFGDWDENNPSSADGYTHIFNKVREERSSGANVSGSSRTPTHQSSRNPNNTSSCCCFGFGGK

0000000000000000000000000000000000000000000000000000000000000000000000000000000000000000000000000000000000000000000000000000000000000000000000000000000000000000000000000000000000000000000000000000000000000000000

0000000000000000000000000000000000000000000000000000000000000000000000000000000000000000000000000000000000000000000000000000000000000000000000000000000000000000000000000000000000000000000000000000000000000000000

1111111111111111111111111111111111111111111111111111111111111111111111111111111111111111111111111111111111111111111111111111111111111111111111111111111111111111111111111111111111111111111111111111111111111111111

0000000000000000000000000000000000000000000000000000000000000000000000000000000000000000000000000000000000000000000000000000000000000000000000000000000000000000000000000000000000000000000000000000000000000000000

0000000000000000000000000000000000000000000000000000000000000000000000000000000000000000000000000000000000000000000000000000000000000000000000000000000000000000000000000000000000000000000000000000000000000000000

>DP01788

MDPGNENSATEAAAIIDLDPDFEPQSRPRSCTWPLPRPEIANQPSEPPEVEPDLGEKVHTEGRSEPILLPSRLPEPAGGPQPGILGAVTGPRKGGSRRNAWGNQSYAELISQAIESAPEKRLTLAQIYEWMVRTVPYFKDKGDSNSSAGWKNSIRHNLSLHSKFIKVHNEATGKSSWWMLNPEGGKSGKAPRRRAASMDSSSKLLRGRSKAPKKKPSVLPAPPEGATPTSPVGHFAKWSGSPCSRNREEADMWTTFRPRSSSNASSVSTRLSPLRPESEVLAEEIPASVSSYAGGVPPTLNEGLELLDGLNLTSSHSLLSRSGLSGFSLQHPGVTGPLHTYSSSLFSPAEGPLSAGEGCFSSSQALEALLTSDTPPPPADVLMTQVDPILSQAPTLLLLGGLPSSSKLATGVGLCPKPLEAPGPSSLVPTLSMIAPPPVMASAPIPKALGTPVLTPPTEAASQDRMPQDLDLDMYMENLECDMDNIISDLMDEGEGLDFNFEPDP

0000000000000000000000000000000000000000000000000000000000000000000000000000000000000000000000000000000000000000000000000000000000000000000000000000000000000000000000000000000000000000000000000000000000000000000000000000000000000000000000000000000000000000000000000000000000000000000000000000000000000000000000000000000000000000000000000000000000000000000000000000000000000000000000000000000000000000000000000000000000000000000000000000000000000000000000000000000000000000000000000000000000000000000000000

0000000000000000000000000000000000000000000000000000000000000000000000000000000000000000000000000000000000000000000000000000000000000000000000000000000000000000000000000000000000000000000000000000000000000000000000000000000000000000000000000000000000000000000000000000000000000000000000000000000000000000000000000000000000000000000000000000000000000000000000000000000000000000000000000000000000000000000000000000000000000000000000000000000000000000000000000000000000000000000000000000000000000000000000000

0000000000000000000000000000000000000000000000000000000000000000000000000000000000000000000000000000000000000000000000000000000000000000000000000000000000000000000000000000000000000111111111111111111111110000000000000000000000000000000000000000000000000000000000000000000000000000000000000000000000000000000000000000000000000000000000000000000000000000000000000000000000000000000000000000000000000000000000000000000000000000000000000000000000000000000000000000000000000000000000000000000000000000000000000

0000000000000000000000000000000000000000000000000000000000000000000000000000000000000000000000000000000000000000000000000000000000000000000000000000000000000000000000000000000000000000000000000000000000000000000000000000000000000000000000000000000000000000000000000000000000000000000000000000000000000000000000000000000000000000000000000000000000000000000000000000000000000000000000000000000000000000000000000000000000000000000000000000000000000000000000000000000000000000000000000000000000000000000000000

0000000000000000000000000000000000000000000000000000000000000000000000000000000000000000000000000000000000000000000000000000000000000000000000000000000000000000000000000000000000000000000000000000000000000000000000000000000000000000000000000000000000000000000000000000000000000000000000000000000000000000000000000000000000000000000000000000000000000000000000000000000000000000000000000000000000000000000000000000000000000000000000000000000000000000000000000000000000000000000000000000000000000000000000000

>DP02791

MAGAEGFQYRAVYPFRRERPEDLELLPGDLLVVSRVALQALGVADGGERCPHNVGWMPGFNERTRQRGDFPGTYVEFLGPVALARPGPRPRGPRPLPARPLDGSSESGHILPDLAEQFSPPDPAPPILVKLVEAIEQAELDSECYSKPELPATRTDWSLSDLEQWDRTALYDAVKGFLLALPAAVVTPEAAAEAYRALREVAGPVGLVLEPPTLPLHQALTLRFLLQHLGRVARRAPSPDTAVHALASAFGPLLLRIPPSGGEGDGSEPVPDFPVLLLERLVQEHVEEQDAAPPALPPKPSKAKPAPTALANGGSPPSLQDAEWYWGDISREEVNERLRDTPDGTFLVRDASSKIQGEYTLTLRKGGNNKLIKVFHRDGHYGFSEPLTFCSVVELISHYRHESLAQYNAKLDTRLLYPVSKYQQDQVVKEDSIEAVGAQLKVYHQQYQDKSREYDQLYEEYTRTSQELQMKRTAIEAFNETIKIFEEQGQTQEKCSKEYLERFRREGNEKEMQRILLNSERLKSRIAEIHESRTKLEQDLRAQASDNREIDKRMNSLKPDLMQLRKIRDQYLVWLTQKGARQRKINEWLGIKNETEDQYSLMEDEDALPHHEERTWYVGKINRTQAEEMLSGKRDGTFLIRESSQRGCYACSVVVDGDTKHCVIYRTATGFGFAEPYNLYGSLKELVLHYQHASLVQHNDALTVTLAHPVRAPGPGPPSAAR

00000000000000000000000000000000000000000000000000000000000000000000000000000000000000000000000000000000000000000000000000000000000000000000000000000000000000000000000000000000000000000000000000000000000000000000000000000000000000000000000000000000000000000000000000000000000000000000000000000000000000000000000000000000000000000000000000000000000000000000000000000000000000000000000000000000000000000000000000000000000000000000000000000000000000000000000000000000000000000000000000000000000000000000000000000000000000000000000000000000000000000000000000000000000000000000000000000000000000000000000000000000000000000000000000000000000000000000000000000000000000000000000000000000000000000000000000000000000000000000000000

00000000000000000000000000000000000000000000000000000000000000000000000000000000000000000000000000000000000000000000000000000000000000000000000000000000000000000000000000000000000000000000000000000000000000000000000000000000000000000000000000000000000000000000000000000000000000000000000000000000000000000000000000000000000000000000000000000000000000000000000000000000000000000000000000000000000000000000000000000000000000000000000000000000000000000000000000000000000000000000000000000000000000000000000000000000000000000000000000000000000000000000000000000000000000000000000000000000000000000000000000000000000000000000000000000000000000000000000000000000000000000000000000000000000000000000000000000000000000000000000000

00000000000000000000000000000000000000000000000000000000000000000000000000000000000000000000000000000000000000000000000000000000000000000000000000000000000000000000000000000000000000000000000000000000000000000000000000000000000000000000000000000000000000000000000000000000000000000000000000000000000000000000000000000000000000000000000000000000000000000000000000000000000000000000000000000000000000000000000000000000000000000000000000000000000000000000000000000000000000000000000000000000000000000000000000000000000000000000000000000000000000000000000000000000000000000000000111111111111111111111111111111110000000000000000000000000000000000000000000000000000000000000000000000000000000000000000000000000000000000000000000

00000000000000000000000000000000000000000000000000000000000000000000000000000000000000000000000000000000000000000000000000000000000000000000000000000000000000000000000000000000000000000000000000000000000000000000000000000000000000000000000000000000000000000000000000000000000000000000000000000000000000000000000000000000000000000000000000000000000000000000000000000000000000000000000000000000000000000000000000000000000000000000000000000000000000000000000000000000000000000000000000000000000000000000000000000000000000000000000000000000000000000000000000000000000000000000000000000000000000000000000000000000000000000000000000000000000000000000000000000000000000000000000000000000000000000000000000000000000000000000000000

00000000000000000000000000000000000000000000000000000000000000000000000000000000000000000000000000000000000000000000000000000000000000000000000000000000000000000000000000000000000000000000000000000000000000000000000000000000000000000000000000000000000000000000000000000000000000000000000000000000000000000000000000000000000000000000000000000000000000000000000000000000000000000000000000000000000000000000000000000000000000000000000000000000000000000000000000000000000000000000000000000000000000000000000000000000000000000000000000000000000000000000000000000000000000000000000000000000000000000000000000000000000000000000000000000000000000000000000000000000000000000000000000000000000000000000000000000000000000000000000000

>DP02419

MEFPFDVDALFPERITVLDQHLRPPARRPGTTTPARVDLQQQIMTIIDELGKASAKAQNLSAPITSASRMQSNRHVVYILKDSSARPAGKGAIIGFIKVGYKKLFVLDDREAHNEVEPLCILDFYIHESVQRHGHGRELFQYMLQKERVEPHQLAIDRPSQKLLKFLNKHYNLETTVPQVNNFVIFEGFFAHQHRPPAPSLRATRHSRAAAVDPTPAAPARKLPPKRAEGDIKPYSSSDREFLKVAVEPPWPLNRAPRRATPPAHPPPRSSSLGNSPERGPLRPFVPEQELLRSLRLCPPHPTARLLLAADPGGSPAQRRRTRGTPPGLVAQSCCYSRHGGVNSSSPNTGNQDSKQGEQETKNRSASEEQALSQDGSGEKPMHTAPPQAPAPPAQSWTVGGDILNARFIRNLQERRSTRPW

0000000000000000000000000000000000000000000000000000000000000000000000000000000000000000000000000000000000000000000000000000000000000000000000000000000000000000000000000000000000000000000000000000000000000000000000000000000000000000000000000000000000000000000000000000000000000000000000000000000000000000000000000000000000000000000000000000000000000000000000000000000000000000000000000000000000000000000000000000000000000

0000000000000000000000000000000000000000000000000000000000000000000000000000000000000000000000000000000000000000000000000000000000000000000000000000000000000000000000000000000000000000000000000000000000000000000000000000000000000000000000000000000000000000000000000000000000000000000000000000000000000000000000000000000000000000000000000000000000000000000000000000000000000000000000000000000000000000000000000000000000000

0000000000000000000000000000000000000000000000000000000000000000000000000000000000000000000000000000000000000000000000000000000000000000000000000000000000000000000000000000000000000000000000000000000000000000000000000000000000000011111000000000000000000000000000000000000000000000000000000000000000000000000000000000000000000000000000000000000000000000000000000000000000000000000000000000000000000000000000000000000000000

0000000000000000000000000000000000000000000000000000000000000000000000000000000000000000000000000000000000000000000000000000000000000000000000000000000000000000000000000000000000000000000000000000000000000000000000000000000000000000000000000000000000000000000000000000000000000000000000000000000000000000000000000000000000000000000000000000000000000000000000000000000000000000000000000000000000000000000000000000000000000

0000000000000000000000000000000000000000000000000000000000000000000000000000000000000000000000000000000000000000000000000000000000000000000000000000000000000000000000000000000000000000000000000000000000000000000000000000000000000000000000000000000000000000000000000000000000000000000000000000000000000000000000000000000000000000000000000000000000000000000000000000000000000000000000000000000000000000000000000000000000000

>DP01241

MVLRSGICGLSPHRIFPSLLVVVALVGLLPVLRSHGLQLSPTASTIRSSEPPRERSIGDVTTAPPEVTPESRPVNHSVTDHGMKPRKAFPVLGIDYTHVRTPFEISLWILLACLMKIGFHVIPTISSIVPESCLLIVVGLLVGGLIKGVGETPPFLQSDVFFLFLLPPIILDAGYFLPLRQFTENLGTILIFAVVGTLWNAFFLGGLMYAVCLVGGEQINNIGLLDNLLFGSIISAVDPVAVLAVFEEIHINELLHILVFGESLLNDAVTVVLYHLFEEFANYEHVGIVDIFLGFLSFFVVALGGVLVGVVYGVIAAFTSRFTSHIRVIEPLFVFLYSYMAYLSAELFHLSGIMALIASGVVMRPYVEANISHKSHTTIKYFLKMWSSVSETLIFIFLGVSTVAGSHHWNWTFVISTLLFCLIARVLGVLGLTWFINKFRIVKLTPKDQFIIAYGGLRGAIAFSLGYLLDKKHFPMCDLFLTAIITVIFFTVFVQGMTIRPLVDLLAVKKKQETKRSINEEIHTQFLDHLLTGIEDICGHYGHHHWKDKLNRFNKKYVKKCLIAGERSKEPQLIAFYHKMEMKQAIELVESGGMGKIPSAVSTVSMQNIHPKSLPSERILPALSKDKEEEIRKILRNNLQKTRQRLRSYNRHTLVADPYEEAWNQMLLRRQKARQLEQKINNYLTVPAHKLDSPTMSRARIGSDPLAYEPKEDLPVITIDPASPQSPESVDLVNEELKGKVLGLSRDPAKVAEEDEDDDGGIMMRSKETSSPGTDDVFTPAPSDSPSSQRIQRCLSDPGPHPEPGEGEPFFPKGQ

00000000000000000000000000000000000000000000000000000000000000000000000000000000000000000000000000000000000000000000000000000000000000000000000000000000000000000000000000000000000000000000000000000000000000000000000000000000000000000000000000000000000000000000000000000000000000000000000000000000000000000000000000000000000000000000000000000000000000000000000000000000000000000000000000000000000000000000000000000000000000000000000000000000000000000000000000000000000000000000000000000000000000000000000000000000000000000000000000000000000000000000000000000000000000000000000000000000000000000000000000000000000000000000000000000000000000000000000000000000000000000000000000000000000000000000000000000000000000000000000000000000000000000000000000000000000000000000000000000000000000000000000000000000000000000000000

00000000000000000000000000000000000000000000000000000000000000000000000000000000000000000000000000000000000000000000000000000000000000000000000000000000000000000000000000000000000000000000000000000000000000000000000000000000000000000000000000000000000000000000000000000000000000000000000000000000000000000000000000000000000000000000000000000000000000000000000000000000000000000000000000000000000000000000000000000000000000000000000000000000000000000000000000000000000000000000000000000000000000000000000000000000000000000000000000000000000000000000000000000000000000000000000000000000000000000000000000000000000000000000000000000000000000000000000000000000000000000000000000000000000000000000000000000000000000000000000000000000000000000000000000000000000000000000000000000000000000000000000000000000000000000000000

00000000000000000000000000000000000000000000000000000000000000000000000000000000000000000000000000000000000000000000000000000000000000000000000000000000000000000000000000000000000000000000000000000000000000000000000000000000000000000000000000000000000000000000000000000000000000000000000000000000000000000000000000000000000000000000000000000000000000000000000000000000000000000000000000000000000000000000000000000000000000000000000000000000000000000000000000000000000000000000000000000000000000000000000000000000000000000000000000000000000000000000000000000000000000000000000000000000000000000000000000000000000000000000000000000000000000000000000000000000000000000000000000000000000000000000000000000000000000000000000000000000000000000000000000000000000000000000000000000000000000000111111111100000000000000000000

00000000000000000000000000000000000000000000000000000000000000000000000000000000000000000000000000000000000000000000000000000000000000000000000000000000000000000000000000000000000000000000000000000000000000000000000000000000000000000000000000000000000000000000000000000000000000000000000000000000000000000000000000000000000000000000000000000000000000000000000000000000000000000000000000000000000000000000000000000000000000000000000000000000000000000000000000000000000000000000000000000000000000000000000000000000000000000000000000000000000000000000000000000000000000000000000000000000000000000000000000000000000000000000000000000000000000000000000000000000000000000000000000000000000000000000000000000000000000000000000000000000000000000000000000000000000000000000000000000000000000000000000000000000000000000000000
[truncated: 1,290,680 more chars]
