# Supplementary material for: DMFpred: Predicting protein disorder molecular functions based on protein cubic language model
Source: PLoS Comput Biol. 2022 Oct 31;18(10):e1010668. doi: 10.1371/journal.pcbi.1010668 (PMC9674156; doi:10.1371/journal.pcbi.1010668)
Supplement: S2 Data — (DOCX) [file pcbi.1010668.s007.docx]

# The IDRs pre-training dataset.

Line 1: >Protein ID

Line 2: Protein sequence in one-letter amino acid encoding

Line 3: Annotations of disordered residues

# Training set (2639 proteins)

>DM_train26

GLVPRGSHMMEILRGSPALSAFRINKLLARFQAANLQVHNIYAEYVHFADLNAPLNDSEQAQLTRLLQYGPALSSHTPAGKLLLVTPRPGTISPWSSKATDIAHNCGLQQVDRLERGVAYYIEASTLTAEQWRQVAAELHDRMMETVFSSLTDAEKLFIHHQPAPVSSVDLLGEGRQALIDANLRLGLALAEDEIDYLQEAFTKLGRNPNDIELYMFAQANSEHCRHKIFNADWIIDGKPQPKSLFKMIKNTFETTPDYVLSAYKDNAAVMEGSAVGRYFADHNTGRYDFHQEPAHILMKVETHNHPTAISPWPGAATGSGGEIRDEGATGRGAKPKAGLVGFSVSNLRIPGFEQPWEEDFGKPERIVTALDIMTEGPLGGAAFNNEFGRPALTGYFRTYEEKVNSHNGEELRGYHKPIMLAGGIGNIRADHVQKGEIVVGAKLIVLGGPAMNIGLGGGAASSMASGQSDADLDFASVQRDNPEMERRCQEVIDRCWQLGDANPILFIHDVGAGGLSNAMPELVSDGGRGGKFELRDILSDEPGMSPLEIWCNESQERYVLAVAADQLPLFDELCKRERAPYAVIGDATEEQHLSLHDNHFDNQPIDLPLDVLLGKTPKMTRDVQTLKAKGDALNRADITIADAVKRVLHLPTVAEKTFLVTIGDRTVTGMVARDQMVGPWQVPVADCAVTTASLDSYYGEAMSIGERAPVALLDFAASARLAVGEALTNIAATQIGDIKRIKLSANWMAAAGHPGEDAGLYDAVKAVGEELCPQLGLTIPVGKDSMSMKTRWQEGNEQREMTSPLSLVISAFARVEDVRHTLTPQLSTEDNALLLIDLGKGHNALGATALAQVYRQLGDKPADVRDVAQLKGFYDAMQALVAARKLLAWHDRSDGGLLVTLAEMAFAGHCGVQVDIAALGDDHLAALFNEELGGVIQVRAEDRDAVEALLAQYGLADCVHYLGQALAGDRFVITANDQTVFSESRTTLRVWWAETTWQMQRLRDNPQCADQEHEAKANDTDPGLNVKLSFDINEDIAAPYIATGARPKVAVLREQGVNSHVEMAAAFHRAGFDAIDVHMSDLLGGRIGLGNFHALVACGGFSYGDVLGAGEGWAKSILFNHRVRDEFETFFHRPQTLALGVCNGCQMMSNLRELIPGSELWPRFVRNHSDRFEARFSLVEVTQSPSLLLQGMVGSQMPIAVSHGEGRVEVRDDAHLAALESKGLVALRYVDNFGKVTETYPANPNGSPNGITAVTTENGRVTIMMPHPERVFRTVANSWHPENWGEDSPWMRIFRNARKQLG

0000000000000000000000000000000000000000000000000000000000000000000000000000000000000000000000000000000000000000000000000000000000000000000000000000000000000000000000000000000000000000000000000000000000000000000000000000000000000000000000000000000000000000000000000000000000000000000000000000000000000000000000000000000000000000000000000000000000000000000000000000000000000000000000000000000000000000000000000000000000000000000000000000000000000000000000011111111111111111110000000000000000000000000000000000000000000000000000000000000000000000000000000000000000000000000000000000000000000000000000000000000000000000000000000000000000000000000000000000000000000000000000000000000000000000000000000000000000000000000000000000000000000000000000000000000000000000000000000000000000000000000000000000000000000000000000000000000000000000000000000000000000000000000000000000000000000000000000000000000000000000000000000000000000000000000000000000000000000000000000000000000000000000000000000000000000000000000000000000000000000000000000000000000000000000000000000000000000000000000000000000000000000000000000000000000000000000000000000000000000000000000000000000000000000000000000000000000000000000000000000000000000000000000000000000000000000000000000000000000000000000000000000000000000000000000000000000000

>DM_train27

MANVWGVRLADSLSSPTIETRTRQYTLHDLCSDLDANPGREPWKPLRNQRTNNIVAVQLFRPLQGLVLDTQLYGFPGAFDDWERFMREKLRVLKYEVLRIYPISNYSNEHVNVFVANALVGAFLSNQAFYDLLPLLIINDTMIGDLLGTGASLSQFFQSHGDVLEVAAGRKYLQMENYSNDDDDPPLFAKDLSDYAKAFYSDTYEVLDRFFWTHDSSAGVLVHYDKPTNGHHYLLGTLTQMVSAPPYIINATDAMLLESCLEQFSANVRARPAQPVTRLDQCYHLRWGAQYVGEDSLTYRLGVLSLLATNGYQLARPIPRQLTNRWLSSFVSQIMSDGVNETPLWPQERYVQIAYDSPSVVDGATQYGYVRKNQLRLGMRISALQSLSDTPSPVQWLPQYTIDQAAMDEGDLMVSRLTQLPLRPDYGNIWVGDALSYYVDYNRSHRVVLSSELPQLPDTYFDGDEQYGRSLFSLARKIGDRSLVKDTAVLKHAYQAIDPNTGKEYLRSRQSVAYFGASAGHSGADQPLVIEPWIQGKISGVPPPSSVRQFGYDVARGAIVDLARPFPSGDYQFVYSDVDQVVDGHDDLSISSGLVESLLSSCMHATAPGGSFVVKINFPTRPVWHYIEQKILPNITSYMLIKPFVTNNVELFFVAFGVHQHSSLTWTSGVYFFLVDHFYRYETLSTISRQLPSFGYVDDGSSVTGIETISIENPGFSNMTQAARIGISGLCANVGNARKSIAIYESHGARVLTITSRRSPASARRKSRLRYLPLIDPRSLEVQARTILPADPVLFENVSGASPHVCLTMMYNFEVSSAVYDGDVVLDLGTGPEAKILELIPATSPVTCVDIRPTAQPSGCWNVRTTFLELDYLSDGWITGVRGDIVTCMLSLGAAAAGKSMTFDAAFQQLIKVLSKSTANVVLVQVNCPTDVVRSIKGYLEIDSTNKRYRFPKFGRDEPYSDMDALEKICRTAWPNCSITWVPLSYDLRWTRLALLESTTLSSASIRIAELMYKYMPIMRIDIHGLPMEKRGNFIVGQNCSLVIPGFNAQDVFNCYFNSALAFSTEDVNAAMIPQVSAQFDATKGEWTLDMVFSDAGIYTMQALVGSNANPVSLGSFVVDSPDVDITDAWPAQLDFTIAGTDVDITVNPYYRLMTFVRIDGQWQIANPDKFQFFSSASGTLVMNVKLDIADKYLLYYIRDVQSRDVGFYIQHPLQLLNTITLPTNEDLFLSAPDMREWAVKESGNTICILNSQGFVLPQDWDVLTDTISWSPSIPTYIVPPGDYTLTPL

00000000000000000000000000000000000000000000000000000000000000000000000000000000000000000000000000000000000000000000000000000000000000000000000000000000000000000000000000000000000000000000000000000000000000000000000000000000000000000000000000000000000000000000000000000000000000000000000000000000000000000000000000000000000000000000000000000000000000000000000000000000000000000000000000000000000000000000000000000000000000000000000000000000000000000000000000000000000000000000000000000000000000000000000000000000000000000000000000000000000000000000000000000000000000000000000000000000000000000000000000000000000000000000000000000000000000000000000000000000000000000000000000000000000000000000000000000000000000000000000000000000000000000000000000000000000000000000000000000000000000000000000000000000000000000000000000000000000000000000000000000000000000000000000000000000000000000000000000000000000000000000000000000000000000000000000000000000000000000000000000000000000000000000000000000000000000000000000000000000000000000000000000000000000000000000000000000000000000000000000000000000000000000000000000000000000000000000000000000000000000000000000000000000000000000000000111100000000000000000000000000000000000000000000000000000000000000000000000000000000000000000000000000000000000000

>DM_train29

MSSMILTQFGPFIESISGITDQSNDVFEDAAKAFSMFTRSDVYKALDEIPFSDDAMLPIPPTIYTKPSHDSYYYIDALNRVRRKTYQGPDDVYVPNCSIVELLEPHETLTSYGRLSEAIENRAKDGDSQARIATTYGRIAESQARQIKAPLEKFVLALLVAEAGGSLYDPVLQKYDEIPDLSHNCPLWCFREICRHISGPLPDRAPYLYLSAGVFWLMSPRMTSAIPPLLSDLVNLAILQQTAGLDPSLVKLGVQICLHAAASSSYSWFILKTKSIFPQNTLHSMYESLEGGYCPNLEWLEPRSDYKFMYMGVMPLSAKYARSAPSNDKKARELGEKYGLSSVVGELRKRTKTYVKHDFASVRYIRDAMACTSGIFLVRTPTETVLQEYTQSPEIKVPIPQKDWTGPIGEIRILKDTTSSIARYLYRTWYLAAARMAAQPRTWDPLFQAIMRSQYVTARGGSGAALRESLYAINVSLPDFKGLPVKAATKIFQAAQLANLPFSHTSVAILADTSMGLRNQVQRRPRSIMPLNVPQQQVSAPHTLTADYINYHMNLSPTSGSAVIEKVIPLGVYASSPPNQSINIDISACDASITWDFFLSVIMAAIHEGVASSSIGKPFMGVPASIVNDESVVGVRAARPISGMQNMIQHLSKLYKRGFSYRVNDSFSPGNDFTHMTTTFPSGSTATSTEHTANNSTMMETFLTVWGPEHTDDPDVLRLMKSLTIQRNYVCQGDDGLMIIDGTTAGKVNSETIQNDLELISKYGEEFGWKYDIAYDGTAEYLKLYFIFGCRIPNLSRHPIVGKERANSSAEEPWPAILDQIMGVFFNGVHDGLQWQRWIRYSWALCCAFSRQRTMIGESVGYLQYPMWSFVYWGLPLVKAFGSDPWIFSWYMPTGDLGMYSWISLIRPLMTRWMVANGYVTDRCSTVFGNADYRRCFNELKLYQGYYMAQLPRNPKKSGRAASREVREQFTQALSDYLMQNPELKSRVLRGRSEWEKYGAGIIHNPPSLFDVPHKWYQGAQEAAIATREELAEMDETLMRARRHSYSSFSKLLEAYLLVKWRMCEAREPSVDLRLPLCAGIDPLNSDPFLKMVSVGPMLQSTRKYFAQTLFMAKTVSGLDVNAIDSALLRLRTLGADKKALTAQLLMVGLQESEADALAGKIMLQDVNTVQLARVVNLAVPDTWMSLDFDSMFKHHVKLLPKDGRHLNTDIPPRMGWLRAILRFLGAGMVMTATGVAVDIYLEDIHGGGRSLGQRFMTWMRQEGRSA

0000000000000000000000000000000000000000000000000000000000000000000000000000000000000000000000000000000000000000000000000000000000000000000000000000000000000000000000000000000000000000000000000000000000000000000000000000000000000000000000000000000000000000000000000000000000000000000000000000000000000000000000000000000000000000000000000000000000000000000000000000000000000000000000000000000000000000000000000000000000000000000000000000000000000000000000000000000000000000000000000000000000000000000000000000000000000000000000000000000000000000000000000000000000000000000000000000000000000000000000000000000000000000000000000000000000000000000000000000000000000000000000000000000000000000000000000000000000000000000000000000000000000000000000000000000000000000000000000000000000000000000000000000000000000000000000000000000000000000000000000000000000000000000000000000000000000000000000000000000000000000000000000000000000000000000000000000000000000000000011111111000000000000000000000000000000000000000000000000000000000000000000000000000000000000000000000000000000000000000000000000000000000000000000000000000000000000000000000000000000000000000000000000000000000000000000000000000000000000000000000000000000000000000000000000000000000000000000000000000000000000000

>DM_train31

ESTVTEELKEGIDAVYPSLVGTADSKAEGIKNYFKLSFTLPEEQKSRTVGSEAPLKDVAQALSSRARYELFTEKETANPAFNGEVIKRYKELMEHGEGIADILRSRLAKFLNTKDVGKRFAQGTEANRWVGGKLLNIVEQDGDTFKYNEQLLQTAVLAGLQWRLTATSNTAIKDAKDVAAITGIDQALLPEGLVEQFDTGMTLTEAVSSLAQKIESYWGLSRNPNAPLGYTKGIPTAMAAEILAAFVESTDVVENIVDMSEIDPDNKKTIGLYTITELDSFDPINSFPTAIEEAVLVNPTEKMFFGDDIPPVANTQLRNPAVRNTPEQKAALKAEQATEFYVHTPMVQFYETLGKDRILELMGAGTLNKELLNDNHAKSLEGKNRSVEDSYNQLFSVIEQVRAQSEDISTVPIHYAYNMTRVGRMQMLGKYNPQSAKLVREAILPTKATLDLSNQNNEDFSAFQLGLAQALDIKVHTMTREVMSDELTKLLEGNLKPAIDMMVEFNTTGSLPENAVDVLNTALGDRKSFVALMALMEYSRYLVAEDKSAFVTPLYVEADGVTNGPINAMMLMTGGLFTPDWIRNIAKGGLFIGSPNKTMNEHRSTADNNDLYQASTNALMESLGKLRSNYASNMPIQSQIDSLLSLMDLFLPDINLGENGALELKRGIAKNPLTITIYGSGARGIAGKLVSSVTDAIYERMSDVLKARAKDPNISAAMAMFGKQAASEAHAEELLARFLKDMETLTSTVPVKRKGVLELQSTGTGAKGKINPKTYTIKGEQLKALQENMLHFFVEPLRNGITQTVGESLVYSTEQLQKATQIQSVVLEDMFKQRVQEKLAEKAKDPTWKKGDFLTQKELNDIQASLNNLAPMIETGSQTFYIAGSENAEVANQVLATNLDDRMRVPMSIYAPAQAGVAGIPFMTIGTGDGMMMQTLSTMKGAPKNTLKIFDGMNIGLNDITDASRKANEAVYTSWQGNPIKNVYESYAKFMKNVDFSKLSPEALEAIGKSALEYDQRENATVDDIANAASLIERNLRNIALGVDIRHKVLDKVNLSIDQMAAVGAPYQNNGKIDLSNMTPEQQADELNKLFREELEARKQKVAKA

1111111111000000000000000000000000000000000000000000000000000000000000000000000000000000000000000000000000000000000000000000000000000000000000000000000000000000000000000000000000000000000000000000000000000000000000000000000000000000000000000000000000000000000000000000000000000000000000000000000000000000000000000000000000000000000000000000000000000000000000000000000000000000000000000000000000000000000000000000000000000000000000000000000000000000000000000000000000000000000000000000000000000000000000000000000000000000000000000000000000000000000000000000000000000000000000000000000000000000000000000000000000000000000000000000000000000000000000000000000000000000000000000000000000000000000000000000000000000000000000000000000000000000000000000000000000000000000000000000000000000000000000000000000000000000000000000000000000000000000000000000000000000000000000000000000000000000000000000000000000000000000000000000000000000000000000000000000000000000000000000000000000000000000000000000000000000000000000000000000000000000000000000000000000000000000000000000000000000000000000000000000000000000000000011

>DM_train33

MPNFFIDRPIFAWVIAIIIMLAGGLAILKLPVAQYPTIAPPAVTISASYPGADAKTVQDTVTQVIEQNMNGIDNLMYMSSNSDSTGTVQITLTFESGTDADIAQVQVQNKLQLAMPLLPQEVQQQGVSVEKSSSSFLMVVGVINTDGTMTQEDISDYVAANMKDAISRTSGVGDVQLFGSQYAMRIWMNPNELNKFQLTPVDVITAIKAQNAQVAAGQLGGTPPVKGQQLNASIIAQTRLTSTEEFGKILLKVNQDGSRVLLRDVAKIELGGENYDIIAEFNGQPASGLGIKLATGANALDTAAAIRAELAKMEPFFPSGLKIVYPYDTTPFVKISIHEVVKTLVEAIILVFLVMYLFLQNFRATLIPTIAVPVVLLGTFAVLAAFGFSINTLTMFGMVLAIGLLVDDAIVVVENVERVMAEEGLPPKEATRKSMGQIQGALVGIAMVLSAVFVPMAFFGGSTGAIYRQFSITIVSAMALSVLVALILTPALCATMLKPIAKGDHGEGKKGFFGWFNRMFEKSTHHYTDSVGGILRSTGRYLVLYLIIVVGMAYLFVRLPSSFLPDEDQGVFMTMVQLPAGATQERTQKVLNEVTHYYLTKEKNNVESVFAVNGFGFAGRGQNTGIAFVSLKDWADRPGEENKVEAITMRATRAFSQIKDAMVFAFNLPAIVELGTATGFDFELIDQAGLGHEKLTQARNQLLAEAAKHPDMLTSVRPNGLEDTPQFKIDIDQEKAQALGVSINDINTTLGAAWGGSYVNDFIDRGRVKKVYVMSEAKYRMLPDDIGDWYVRAADGQMVPFSAFSSSRWEYGSPRLERYNGLPSMEILGQAAPGKSTGEAMELMEQLASKLPTGVGYDWTGMSYQERLSGNQAPSLYAISLIVVFLCLAALYESWSIPFSVMLVVPLGVIGALLAATFRGLTNDVYFQVGLLTTIGLSAKNAILIVEFAKDLMDKEGKGLIEATLDAVRMRLRPILMTSLAFILGVMPLVISTGAGSGAQNAVGTGVMGGMVTATVLAIFFVPVFFVVVRRRFSRKNEDIEHSHTVDHHHHHH

111111000000000000000000000000000000000000000000000000000000000000000000000000000000000000000000000000000000000000000000000000000000000000000000000000000000000000000000000000000000000000000000000000000000000000000000000000000000000000000000000000000000000000000000000000000000000000000000000000000000000000000000000000000000000000000000000000000000000000000000000000000000000000000000000000000000000000000000000000000000000000000000000000000000000000000000000000000000000000000000000000000000000000111111111111110000000000000000000000000000000000000000000000000000000000000000000000000000000000000000000000000000000000000000000000000000000000000000000000000000000000000000000000000000000000000000000000000000000000000000000000000000000000000000000000000000000000000000000000000000000000000000000000000000000000000000000000000000000000000000000000000000000000011111111100000000000000000000000000000000000000000000000000000000000000000000000000000000000000000000000000000000000000000000000000000000000000000000000000000000000000000000000011111111111111111

>DM_train34

DDPIRPPLKVARSPRPGQCQDVVQDVPNVDVQMLELYDRMSFKDIDGGVWKQGWNIKYDPLKYNAHHKLKVFVVPHSHNDPGWIQTFEEYYQHDTKHILSNALRHLHDNPEMKFIWAEISYFARFYHDLGENKKLQMKSIVKNGQLEFVTGGWVMPDEANSHWRNVLLQLTEGQTWLKQFMNVTPTASWAIAPFGHSPTMPYILQKSGFKNMLIQRTHYSVKKELAQQRQLEFLWRQIWDNKGDTALFTHMMPFYSYDIPHTCGPDPKVCCQFDFKRMGSFGLSCPWKVPPRTISDQNVAARSDLLVDQWKKKAELYRTNVLLIPLGDDFRFKQNTEWDVQRVNYERLFEHINSQAHFNVQAQFGTLQEYFDAVHQAERAGQAEFPTLSGDFFTYADRSDNYWSGYYTSRPYHKRMDRVLMHYVRAAEMLSAWHSWDGMARIEERLEQARRELSLFQHHDGITGTAKTHVVVDYEQRMQEALKACQMVMQQSVYRLLTKPSIYSPDFSFSYFTLDDSRWPGSGVEDSRTTIILGEDILPSKHVVMHNTLPHWREQLVDFYVSSPFVSVTDLANNPVEAQVSPVWSWHHDTLTKTIHPQGSTTKYRIIFKARVPPMGLATYVLTISDSKPEHTSYASNLLLRKNPTSLPLGQYPEDVKFGDPREISLRVGNGPTLAFSEQGLLKSIQLTQDSPHVPVHFKFLKYGVRSHGDRSGAYLFLPNGPASPVELGQPVVLVTKGKLESSVSVGLPSVVHQTIMRGGAPEIRNLVDIGSLDNTEIVMRLETHIDSGDIFYTDLNGLQFIKRRRLDKLPLQANYYPIPSGMFIEDANTRLTLLTGQPLGGSSLASGELEIMQDRRLASDDERGLGQGVLDNKPVLHIYRLVLEKVNNCVRPSKLHPAGYLTSAAHKASQSLLDPLDKFIFAENEWIGAQGQFGGDHPSAREDLDVSVMRRLTKSSAKTQRVGYVLHRTNLMQCGTPEEHTQKLDVCHLLPNVARCERTTLTFLQNLEHLDGMVAPEVCPMETAAYVSSHSS

1111111111111111110000000000000000000000000000000000000000000000000000000000000000000000000000000000000000000000000000000000000000000000000000000000000000000000000000000000000000000000000000000000000000000000000000000000000000000000000000000000000000000000000000000000000000000000000000000000000000000000000000000000000000000000000000000000000000000000000000000000000000000000000000000000000000000000000000000000000000000000000000000000000000000000000000000000000000000000000000000000000000000000000000000000000000000000000000000000000000000000000000000000000000000000000000000000000000000000000000000000000000000000000000000000000000000000000000000000000000000000000000000000000000000000000000000000000000000000000000000000000000000000000000000000000000000000000000000000000000000000000000000000000000000000000000000000000000000000000000000000000000000000000000000000000000000000000000000000000000000000000000000000000000000000000000000000000000000000000000000000000000000000000000000000000000000000000000000000000000000000000000000

>DM_train35

ALVRDDVDYQIFRDFAENKGRFSVGATNVEVRDKNNHSLGNVLPNGIPMIDFSVVDVDKRIATLINPQYVVGVKHVSNGVSELHFGNLNGNMNNGNAKSHRDVSSEENRYFSVEKNEYPTKLNGKAVTTEDQTQKRREDYYMPRLDKFVTEVAPIEASTASSDAGTYNDQNKYPAFVRLGSGSQFIYKKGDNYSLILNNHEVGGNNLKLVGDAYTYGIAGTPYKVNHENNGLIGFGNSKEEHSDPKGILSQDPLTNYAVLGDSGSPLFVYDREKGKWLFLGSYDFWAGYNKKSWQEWNIYKPEFAKTVLDKDTAGSLTGSNTQYNWNPTGKTSVISNGSESLNVDLFDSSQDTDSKKNNHGKSVTLRGSGTLTLNNNIDQGAGGLFFEGDYEVKGTSDSTTWKGAGVSVADGKTVTWKVHNPKSDRLAKIGKGTLIVEGKGENKGSLKVGDGTVILKQQADANNKVKAFSQVGIVSGRSTVVLNDDKQVDPNSIYFGFRGGRLDANGNNLTFEHIRNIDDGARLVNHNTSKTSTVTITGESLITDPNTITPYNIDAPDEDNPYAFRRIKDGGQLYLNLENYTYYALRKGASTRSELPKNSGESNENWLYMGKTSDEAKRNVMNHINNERMNGFNGYFGEEEGKNNGNLNVTFKGKSEQNRFLLTGGTNLNGDLKVEKGTLFLSGRPTPHARDIAGISSTKKDQHFAENNEVVVEDDWINRNFKATNINVTNNATLYSGRNVANITSNITASDNAKVHIGYKAGDTVCVRSDYTGYVTCTTDKLSDKALNSFNATNVSGNVNLSGNANFVLGKANLFGTISGTGNSQVRLTENSHWHLTGDSNVNQLNLDKGHIHLNAQNDANKVTTYNTLTVNSLSGNGSFYYLTDLSNKQGDKVVVTKSATGNFTLQVADKTGEPTKNELTLFDASNATRNNLNVSLVGNTVDLGAWKYKLRNVNGRYDLYNPEVEKRNQTVDTTNITTPHHHHHHVP

00000000000000000000000000000000000000000000000000000000000000000000000000000000000000000000000000000000000000000000000000000000000000000000000000000000000000000000000000000000000000000000000000000000000000000000000000000000000000000000000000000000000000000000000000000000000000000000000000000000000000000000000000000000000000000000000000000000000000000000000000000000000000000000000000000000000000000000000000000000000000000000000000000000000000000000000000000000000000000000000000000000000000000000000000000000000000000000000000000000000000000000000000000000000000000000000000000000000000000000000000000000000000000000000000000000000000000000000000000000000000000000000000000000000000000000000000000000000000000000000000000000000000000000000000000000000000000000000000000000000000000000000000000000000000000000000000000000000000000000000000000000000000000000000000000000000000000000000000000000000000000000000000000000000000000000000000000111000000000000000000001111111111111111111111111

>DM_train39

MSDLETVAKFLAESVIASTAKTSERNLRQLETQDGFGLTLLHVIASTNLPLSTRLAGALFFKNFIKRKWVDENGNHLLPANNVELIKKEIVPLMISLPNNLQVQIGEAISSIADSDFPDRWPTLLSDLASRLSNDDMVTNKGVLTVAHSIFKRWRPLFRSDELFLEIKLVLDVFTAPFLNLLKTVDEQITANENNKASLNILFDVLLVLIKLYYDFNCQDIPEFFEDNIQVGMGIFHKYLSYSNPLLEDPDETEHASVLIKVKSSIQELVQLYTTRYEDVFGPMINEFIQITWNLLTSISNQPKYDILVSKSLSFLTAVTRIPKYFEIFNNESAMNNITEQIILPNVTLREEDVELFEDDPIEYIRRDLEGSDTDTRRRACTDFLKELKEKNEVLVTNIFLAHMKGFVDQYMSDPSKNWKFKDLYIYLFTALAINGNITNAGVSSTNNLLNVVDFFTKEIAPDLTSNNIPHIILRVDAIKYIYTFRNQLTKAQLIELMPILATFLQTDEYVVYTYAAITIEKILTIRESNTSPAFIFHKEDISNSTEILLKNLIALILKHGSSPEKLAENEFLMRSIFRVLQTSEDSIQPLFPQLLAQFIEIVTIMAKNPSNPRFTHYTFESIGAILNYTQRQNLPLLVDSMMPTFLTVFSEDIQEFIPYVFQIIAFVVEQSATIPESIKPLAQPLLAPNVWELKGNIPAVTRLLKSFIKTDSSIFPDLVPVLGIFQRLIASKAYEVHGFDLLEHIMLLIDMNRLRPYIKQIAVLLLQRLQNSKTERYVKKLTVFFGLISNKLGSDFLIHFIDEVQDGLFQQIWGNFIITTLPTIGNLLDRKIALIGVLNMVINGQFFQSKYPTLISSTMNSIIETASSQSIANLKNDYVDLDNLEEISTFGSHFSKLVSISEKPFDPLPEIDVNNGVRLYVAEALNKYNAISGNTFLNTILPQLTQENQVKLNQLLVGN

000000000000000000000000000000000000000000000000000000000000000000000000000000000000000000000000000000000000000000000000000000000000000000000000000000000000000000000000000000000000000000000000011000000000000000000000000000000000000000000000000000001111110000000000000000000000000000000000000000000000000000000000000000000000000000000000000000000000000000000000000000000001111000000000000000000000000000000000000001111000000000000000000000000000000000000000000000000000000000000000000000000000000000000000000000000000000000000000000000000000000000000000000000000000000000000000000000000000000000000000000000000000000000000000000000000000000000000000000000000000000000000000000000000000000000000000000000000000000000000000000000000000000000000000000000000000000000000000000000000000000000000000000000000000000000000000000000000000000000000000000000000000000000000000000000000000000011111000000000000000000000000000000000000000000000000000000000000000000000000001

>DM_train40

ATPSMMPQWSYMHISGQDASEYLSPGLVQFARATETYFSLNNKFRNPTVAPTHDVTTDRSQRLTLRFIPVDREDTAYSYKARFTLAVGDNRVLDMASTYFDIRGVLDRGPTFKPYSGTAYNALAPKGAPNPCEWDEAATALEINLEEEDDDNEDEVDEQAEQQKTHVFGQAPYSGINITKEGIQIGVEGQTPKYADKTFQPEPQIGESQWYETEINHAAGRVLKKTTPMKPCYGSYAKPTNENGGQGILVKQQNGKLESQVEMQFFSTTEATAGNGDNLTPKVVLYSEDVDIETPDTHISYMPTIKEGNSRELMGQQSMPNRPNYIAFRDNFIGLMYYNSTGNMGVLAGQASQLNAVVDLQDRNTELSYQLLLDSIGDRTRYFSMWNQAVDSYDPDVRIIENHGTEDELPNYCFPLGGVINTETLTKVKPKTGQENGWEKDATEFSDKNEIRVGNNFAMEINLNANLWRNFLYSNIALYLPDKLKYSPSNVKISDNPNTYDYMNKRVVAPGLVDCYINLGARWSLDYMDNVNPFNHHRNAGLRYRSMLLGNGRYVPFHIQVPQKFFAIKNLLLLPGSYTYEWNFRKDVNMVLQSSLGNDLRVDGASIKFDSICLYATFFPMAHNTASTLEAMLRNDTNDQSFNDYLSAANMLYPIPANATNVPISIPSRNWAAFRGWAFTRLKTKETPSLGSGYDPYYTYSGSIPYLDGTFYLNHTFKKVAITFDSSVSWPGNDRLLTPNEFEIKRSVDGEGYNVAQCNMTKDWFLVQMLANYNIGYQGFYIPESYKDRMYSFFRNFQPMSRQVVDDTKYKDYQQVGILHQHNNSGFVGYLAPTMREGQAYPANFPYPLIGKTAVDSITQKKFLCDRTLWRIPFSSNFMSMGALTDLGQNLLYANSAHALDMTFEVDPMDEPTLLYVLFEVFDVVRVHRPHRGVIETVYLRTPFSAGNATT

111100000000000000000000000000000000000000000000000000000000000000000000000000000000000000000000000000000000000000000000000000000000000111111111111111111111111111110000000000000000000000111111000000000000000000000000000000000000000000000000000000000011111110000000000001111111100000000000000000000000000000000000000000000000000000000000000000000000000000000000000000000000000000000000000000000000000000000000000000000000000000000011111100000000000000000000000000000000000000000000000000000000000000000000000000000000000000000000000000000000000000000000000000000000000000000000000000000000000000000000000000000000000000000000000000000000000000000000000000000000000000000000000000000000000000000000000000000000000000000000000000000000000000000000000000000000000000000000000000000000000000000000000000000000000000000000000000000000000000000000000000000000000000000000000000000000000000000000000000000000000000000000000000000000000000000000000000000011111

>DM_train43

MVAKKWVYYFGGGNADGNKNMKELLGGKGANLAEMVNLGIPVPPGFTITTEACKTYQETETIPQEVADQVRENVSRVEKEMGAKFGDPANPLLFSVRSGAAASMPGMMDTVLNLGLNKVTVDAWVRRAPRLERFVYDSYRRFITMYADIVMQVGREDFEEALSRMKERRGTKFDTDLTASDLKELCDGYLELFELKTGCSFPQDPVMQLFAAIKAVFRSWGNPRATIYRRMNNITGLLGTAVNVQAMVFGNINDRSATGVAFSRSPSTGENFFFGEYLVNAQGEDVVAGIRTPQQINHSLSLRWAKAHGVGEEERRKRYPSMEEAMPENYRLLCDVRKRLENHYRDMQDLEFTVQDGRLWLLQCRNGKRTIHAAVRIAIDMVNEGLISREEAVLRIDPYQVDHLMHPNLEPGAEKANKPIGRGLAASPGAAVGQVVFDAESAKEWSGRGKKVIMVRLETSPEDLAGMDAACGILTARGGMTSHAAVVARGMGKCCVSGCGDMVIRGKSFKLNGSVFREGDYITIDGSKGLIYAGKLKLRSPDLKGSFQTILQWCQEMKRLGVRTNADTPADAAKARSFGAEGVGLCRTEHMFFEGSRINFIREMILADSASGRKAALDKLLPIQRADFVGILRAMRGLPVTIRLLDPPLHEFVPHDAAAQFELAQKLGMPAEKVRNRVNALHELNPMLGHRGCRLGITYPEIYNMQVRAIIEAAIAVSEEGSSVIPEIMVPLVGKKEELSLIREEVVKTAEAVITKSGKRVHYTVGTMIEVPRAAVTADSIAQKADFFSFGTNDLTQMGCGFSRDDAGPFLRHYGNLGIYAQDPFQSIDQEGIGELVRIAVTKGRRVKPMLKMGICGEHGGDPATIGFCHKVGLDYVSCSPFRVPVAIVAAAHASIKDRRAAMKARKGFAAKL

0000000000000000000000000000000000000000000000000000000000000000000000000000000000000000000000000000000111110000000000000000000000000000000000000000000000000000000000000000000000000000000000000000000000000000000000000000000000000000000000000000000000000000000000000000000000000000000000000000000000000000000000000000000000000000000000000000000000000000000000000000000000000000000000000000000000000000000000000000000000000000000000000000000000000000000000000000000000000000000000000000000000000000000000000000000000000000000000000000000000000000000000000000000000000000000000000000000000000000000000000000000000000000000000000000000000000000000000000000000000000000000000000000000000000000000000000000000000000000000000000000000000000000000000000000000000000000000000000000000000000000000000000000000000000000000000000000000000000000000000000000000000000000000000000000000000000000000000000000000000000000111111111

>DM_train45

MVIASVEDGGDGDTSKDDWLWYKQPASQTDATATAGGNYGNPDNNRWQQTTLPFGNGKIGGTVWGEVSRERVTFNEETLWTGGPGSSTSYNGGNNETKGQNGATLRALNKQLANGAETVNPGNLTGGENAAEQGNYLNWGDIYLDYGFNDTTVTEYRRDLNLSKGKADVTFKHDGVTYTREYFASNPDNVMVARLTASKAGKLNFNVSMPTNTNYSKTGETTTVKGDTLTVKGALGNNGLLYNSQIKVVLDNGEGTLSEGSDGASLKVSDAKAVTLYIAAATDYKQKYPSYRTGETAAEVNTRVAKVVQDAANKGYTAVKKAHIDDHSAIYDRVKIDLGQSGHSSDGAVATDALLKAYQRGSATTAQKRELETLVYKYGRYLTIGSSRENSQLPSNLQGIWSVTAGDNAHGNTPWGSDFHMNVNLQMNYWPTYSANMGELAEPLIEYVEGLVKPGRVTAKVYAGAETTNPETTPIGEGEGYMAHTENTAYGWTAPGQSFSWGWSPAAVPWILQNVYEAYEYSGDPALLDRVYALLKEESHFYVNYMLHKAGSSSGDRLTTGVAYSPEQGPLGTDGNTYESSLVWQMLNDAIEAAKAKGDPDGLVGNTTDCSADNWAKNDSGNFTDANANRSWSCAKSLLKPIEVGDSGQIKEWYFEGALGKKKDGSTISGYQADNQHRHMSHLLGLFPGDLITIDNSEYMDAAKTSLRYRCFKGNVLQSNTGWAIGQRINSWARTGDGNTTYQLVELQLKNAMYANLFDYHAPFQIDGNFGNTSGVDEMLLQSNSTFTDTAGKKYVNYTNILPALPDAWAGGSVSGLVARGNFTVGTTWKNGKATEVRLTSNKGKQAAVKITAGGAQNYEVKNGDTAVNAKVVTNADGASLLVFDTTAGTTYTITKKAS

11111111100000000000000000000000000000000000000000000000000000000000000000000000000000000000000000000000000000000000000000000000000000000000000000000000000000000000000000000000000000000000000000000000000000000000000000000000000000000000000000000000000111000000000000000000000000000000000000000000000000000000000000000000000000000000000000000000000000000000000000000000000000000000000000000000000000000000000000000000000000000000000000000000000000000000000000000000000000000000000000000000000000000000000000000000000000000000000000000000000000000000000000000000000000000000000000000000000000000000000000000000000000000000000000000000000000000000000000000000000000000000000000000000000000000000000000000000000000000000000000000000000000000000000000000000000000000000000000000000000000000000000000000000000000000000000000000000000000000000000000000000000000000000000111100000000000000000000000000000011

>DM_train47

MTQQPQAKYRHDYRAPDYQITDIDLTFDLDAQKTVVTAVSQAVRHGASDAPLRLNGEDLKLVSVHINDEPWTAWKEEEGALVISNLPERFTLKIINEISPAANTALEGLYQSGDALCTQCEAEGFRHITYYLDRPDVLARFTTKIIADKIKYPFLLSNGNRVAQGELENGRHWVQWQDPFPKPCYLFALVAGDFDVLRDTFTTRSGREVALELYVDRGNLDRAPWAMTSLKNSMKWDEERFGLEYDLDIYMIVAVDFFNMGAMENKGLNIFNSKYVLARTDTATDKDYLDIERVIGHEYFHNWTGNRVTCRDWFQLSLKEGLTVFRDQEFSSDLGSRAVNRINNVRTMRGLQFAEDASPMAHPIRPDMVIEMNNFYTLTVYEKGAEVIRMIHTLLGEENFQKGMQLYFERHDGSAATCDDFVQAMEDASNVDLSHFRRWYSQSGTPIVTVKDDYNPETEQYTLTISQRTPATPDQAEKQPLHIPFAIELYDNEGKVIPLQKGGHPVNSVLNVTQAEQTFVFDNVYFQPVPALLCEFSAPVKLEYKWSDQQLTFLMRHARNDFSRWDAAQSLLATYIKLNVARHQQGQPLSLPVHVADAFRAVLLDEKIDPALAAEILTLPSVNEMAELFDIIDPIAIAEVREALTRTLATELADELLAIYNANYQSEYRVEHEDIAKRTLRNACLRFLAFGETHLADVLVSKQFHEANNMTDALAALSAAVAAQLPCRDALMQEYDDKWHQNGLVMDKWFILQATSPAANVLETVRGLLQHRSFTMSNPNRIRSLIGAFAGSNPAAFHAEDGSGYLFLVEMLTDLNSRNPQVASRLIEPLIRLKRYDAKRQEKMRAALEQLKGLENLSGDLYEKITKALA

111100000000000000000000000000000000000000000000000000000000000000000000000000000000000000000000000000000000000000000000000000000000000000000000000000000000000000000000000000000000000000000000000000000000000000000000000000000000000000000000000000000000000000000000000000000000000000000000000000000000000000000000000000000000000000000000000000000000000000000000000000000000000000000000000000000000000000000000000000000000000000000000000000000000000000000000000000000000000000000000000000000000000000000000000000000000000000000000000000000000000000000000000000000000000000000000000000000000000000000000000000000000000000000000000000000000000000000000000000000000000000000000000000000000000000000000000000000000000000000000000000000000000000000000000000000000000000000000000000000000000000000000000000000000000000000000000000000000000000000000000000000000000000000000000000

>DM_train48

SAECPVVNELERINCIPDQPPTKATCDQRGCCWNPQGAVSVPWCYYSKNHSYHVEGNLVNTNAGFTARLKNLPSSPVFGSNVDNVLLTAEYQTSNRFHFKLTDQTNNRFEVPHEHVQSFSGNAAASLTYQVEISRQPFSIKVTRRSNNRVLFDSSIGPLLFADQFLQLSTRLPSTNVYGLGEHVHQQYRHDMNWKTWPIFNRDTTPNGNGTNLYGAQTFFLCLEDASGLSFGVFLMNSNAMEVVLQPAPAITYRTIGGILDFYVFLGNTPEQVVQEYLELIGRPALPSYWALGFHLSRYEYGTLDNMREVVERNRAAQLPYDVQHADIDYMDERRDFTYDSVDFKGFPEFVNELHNNGQKLVIIVDPAISNNSSSSKPYGPYDRGSDMKIWVNSSDGVTPLIGEVWPGQTVFPDYTNPNCAVWWTKEFELFHNQVEFDGIWIDMNEVSNFVDGSVSGCSTNNLNNPPFTPRILDGYLFCKTLCMDAVQHWGKQYDIHNLYGYSMAVATAEAAKTVFPNKRSFILTRSTFAGSGKFAAHWLGDNTATWDDLRWSIPGVLEFNLFGIPMVGPDICGFALDTPEELCRRWMQLGAFYPFSRNHNGQGYKDQDPASFGADSLLLNSSRHYLNIRYTLLPYLYTLFFRAHSRGDTVARPLLHEFYEDNSTWDVHQQFLWGPGLLITPVLDEGAEKVMAYVPDAVWYDYETGSQVRWRKQKVEMELPGDKIGLHLRGGYIFPTQQPNTTTLASRKNPLGLIIALDENKEAKGELFWDDGETKDTVANKVYLLCEFSVTQNRLEVNISQSTYKDPNNLAFNEIKILGTEEPSNVTVKHNGVPSQTSPTVTYDSNLKVAIITDIDLLLGEAYTVEW

1111110000000000000000000000000000000000000000000000000000000000000000000000000000000000000000000000000000000000000000000000000000000000000000000000000000000000000000000000000000000000000000000000000000000000000000000000000000000000000000000000000000000000000000000000000000000000000000000000000000000000000000000000000000000000000000000000000000000000000000000000000000000000000000000000000000000000000000000000000000000000000000000000000000000000000000000000000000000000000000000000000000000000000000000000000000000000000000000000000000000000000000000000000000000000000000000000000000000000000000000000000000000000000000000000000000000000000000000000000000000000000000000000000000000000000000000000000000000000000000000000000000000000000000000000000000000000000000000000000000000000000000000000000000000000000000000000000000000000000010000000000000000000000000000000

>DM_train50

MAKPLTDQEKRRQISIRGIVGVENVAELKKSFNRHLHFTLVKDRNVATTRDYYFALAHTVRDHLVGRWIRTQQHYYDKCPKRVYYLSLEFYMGRTLQNTMINLGLQNACDEAIYQLGLDIEELEEIEEDAGLGNGGLGRLAACFLDSMATLGLAAYGYGIRYEYGIFNQKIRDGWQVEEADDWLRYGNPWEKSRPEFMLPVHFYGKVEHTNTGTKWIDTQVVLALPYDTPVPGYMNNTVNTMRLWSARAPNDFNLRDFNVGDYIQAVLDRNLAENISRVLYPNDNFFEGKELRLKQEYFVVAATLQDIIRRFKASKFGSTRGAGTVFDAFPDQVAIQLNDTHPALAIPELMRIFVDIEKLPWSKAWELTQKTFAYTNHTVLPEALERWPVDLVEKLLPRHLEIIYEINQKHLDRIVALFPKDVDRLRRMSLIEEEGSKRINMAHLCIVGSHAVNGVAKIHSDIVKTKVFKDFSELEPDKFQNKTNGITPRRWLLLCNPGLAELIAEKIGEDYVKDLSQLTKLHSFLGDDVFLRELAKVKQENKLKFSQFLETEYKVKINPSSMFDVQVKRIHEYKRQLLNCLHVITMYNRIKKDPKKLFVPRTVIIGGKAAPGYHMAKMIIKLITSVADVVNNDPMVGSKLKVIFLENYRVSLAEKVIPATDLSEQISTAGTEASGTGNMKFMLNGALTIGTMDGANVEMAEEAGEENLFIFGMRIDDVAALDKKGYEAKEYYEALPELKLVIDQIDNGFFSPKQPDLFKDIINMLFYHDRFKVFADYEAYVKCQDKVSQLYMNPKAWNTMVLKNIAASGKFSSDRTIKEYAQNIWNVEPSDLKISLSNESNKVNGN

1111111111111111111111100000000000000000000000000000000000000000000000000000000000000000000000000000000000000000000000000000000000000000000000000000000000000000000000000000000000000000000000000000000000000000000000000000000000000000000000000000000000111111111100000000000000000000000000000000000000000000000000000011111111111100000000000000000000000000000000000000000000000000000000000000000000000000000000000000000000000000000000000000000000000000000000000000000000000000000000000000000000000000000000000000000000000000000000000000000000000000000000000000000000000000000000000000000000000000000000000000000000000000000000000000000000000000000000000000000000000000000000000000000000000000000000000000000000000000000000000000000000000000000000000000000000000000000000000000000000000000000000000000000000000000000000000000000000000001111111111111111

>DM_train51

MFSAGHKIKGTVVLMPKNELEVNPDGSAVDNLNAFLGRSVSLQLISATKADAHGKGKVGKDTFLEGINTSLPTLGAGESAFNIHFEWDGSMGIPGAFYIKNYMQVEFFLKSLTLEAISNQGTIRFVCNSWVYNTKLYKSVRIFFANHTYVPSETPAPLVSYREEELKSLRGNGTGERKEYDRIYDYDVYNDLGNPDKSEKLARPVLGGSSTFPYPRRGRTGRGPTVTDPNTEKQGEVFYVPRDENLGHLKSKDALEIGTKSLSQIVQPAFESAFDLKSTPIEFHSFQDVHDLYEGGIKLPRDVISTIIPLPVIKELYRTDGQHILKFPQPHVVQVSQSAWMTDEEFAREMIAGVNPCVIRGLEEFPPKSNLDPAIYGDQSSKITADSLDLDGYTMDEALGSRRLFMLDYHDIFMPYVRQINQLNSAKTYATRTILFLREDGTLKPVAIELSLPHSAGDLSAAVSQVVLPAKEGVESTIWLLAKAYVIVNDSCYHQLMSHWLNTHAAMEPFVIATHRHLSVLHPIYKLLTPHYRNNMNINALARQSLINANGIIETTFLPSKYSVEMSSAVYKNWVFTDQALPADLIKRGVAIKDPSTPHGVRLLIEDYPYAADGLEIWAAIKTWVQEYVPLYYARDDDVKNDSELQHWWKEAVEKGHGDLKDKPWWPKLQTLEDLVEVCLIIIWIASALHAAVNFGNYPYGGLIMNRPTASRRLLPEKGTPEYEEMINNHEKAYLRTITSKLPTLISLSVIEILSTHASDEVYLGQRDNPHWTSDSKALQAFQKFGNKLKEIEEKLVRRNNDPSLQGNRLGPVQLPYTLLYPSSEEGLTFRGIPNSISI

11111100000000000000111111111110000000000000000000000000000000000000000000000000000000000000000000000000000000000001111110000000000000000000000000000000000000000000000000000000000000000000000000000000000000000000000000000000000000000000000000000000000000000000000000000000000000000000000000000000000000000000000000000000000000000000000000000000000000000000000000000000000000000000000000000000000000000000000000000000000000000000000000000000000000000000000000000000000000000000000000000000000000000000000000000000000000000000000000000000000000000000000000000000000000000000000000000000000000000000000000000000000000000000000000000000000000000000000000000000000000000000000000000000000000000000000000000000000000000000000000000000000000000000000000000000000000000000000000000000000000000000000000000000000000000000000000000000000000000000000

>DM_train52

GCPNDRITLPPANAQRTNMTCHFCIVGCGYHVYKWPELEEGGRAPEQNALGLDFRKQLPPLASTLTPAMTNVVTEHDGARYDIMVVPDKACVVNSGLSSTRGGKMASYMYTPTGDGKERLSAPRLYAADEWVDTTWDHAMALYAGLIKKTLDSDGPQGVFFSCFDHGGAGGGFENTWGTGKLMFSAIQTPMVRIHNRPAYNSECHATREMGIGELNNAYEDAQLADVIWSIGNNPYESQTNYFLNHWLPNLQGATTSKKKERFPNENFPQARIIFVDPRETPSVAIARHVAGNDRVLHLAIEPGTDTALFNGLFTYVVEQGWIDKPFIEAHTKGFDDAVKTNRLSLDECSNITGVPVDMLKRAAEWSYKPKASGQAPRTMHAYEKGIIWGNDNYVIQSALLDLVIATHNVGRRGTGCVRMGGHQEGYTRPPYPGDKKIYIDQELIKGKGRIMTWWGCNNFQTSNNAQALREAILQRSAIVKQAMQKARGATTEEMVDVIYEATQNGGLFVTSINLYPTKLAEAAHLMLPAAHPGEMNLTSMNGERRIRLSEKFMDPPGTAMADCLIAARIANALRDMYQKDGKAEMAAQFEGFDWKTEEDAFNDGFRRAGQPGAPAIDSQGGSTGHLVTYDRLRKSGNNGVQLPVVSWDESKGLVGTEMLYTEGKFDTDDGKAHFKPAPWNGLPATVQQQKDKYRFWLNNGRNNEVWQTAYHDQYNSLMQERYPMAYIEMNPDDCKQLDVTGGDIVEVYNDFGSTFAMVYPVAEIKRGQTFMLFGYVNGIQGDVTTDWTDRDIIPYYKGTWGDIRKVGSMSEFKRTVSFKSRRFG

111110000000000000000000000000000000000000000000000000000000000000000000000000000000000000000000000000000000000000000000000000000000000000000000000000000000000000000000000000000000000000000000000000000000000000000000000000000000000000000000000000000000000000000000000000000000000000000000000000000000000000000000000000000000000000000000000000000000000000000000000000000000000000000000000000000000000000000000000000000000000000000000000000000000000000000000000000000000000000000000000000000000000000000000000000000000000000000000000000000000000000000000000000000000000000000000000000000000000000000000000000000000000000000000000000000000000000000000000000000000000000000000000000000000000000000000000000000000000000000000000000000000000000000000000000000000000000000000000000000000000000000000000000000000000000000000000000000

>DM_train54

SEHPQPVTTQIEKSVNTALNKNYVFNKADYQYTLTNPSLGKIVGGILYPNATGSTTVKISDKSGKIIKEVPLSVTASTEDNFTKLLDKWNDVTIGNYVYDTNDSNMQKLNQKLDETNAKNIEAIKLDSNRTFLWKDLDNLNNSAQLTATYRRLEDLAKQITNPHSTIYKNEKAIRTVKESLAWLHQNFYNVNKDIEGSANWWDFEIGVPRSITGTLSLMNNYFTDAEIKTYTDPIEHFVPDAEYFRKTLVNPFKALGGNLVDMGRVKIIEGLLRKDNTIIEKTSHSLKNLFTTATKAEGFYADGSYIDHTNVAYTGAYGNVLIDGLTQLLPIIQETDYKISNQELDMVYKWINQSFLPLIVKGELMDMSRGRSISREAASSHAAAVEVLRGFLRLANMSNEERNLDLKSTIKTIITSNKFYNVFNNLKSYSDIANMNKLLNDSTVATKPLKSNLSTFNSMDRLAYYNAKKDFGFALSLHSKRTLNYEGMNDENTRGWYTGDGMFYIYNSDQSHYSNHFWPTVNPYKMAGTTEKDAKREDTTKEFMSKHSKDAKEKTGQVTGTSDFVGSVKLNDHFALAAMDFTNWDRTLTAQKGWVILNDKIVFLGSNIKNTNGIGNVSTTIDQRKDDSKTPYTTYVNGKTIDLKQASSQQFTDTKSVFLESKEPGRNIGYIFFKNSTIDIERKEQTGTWNSINRTSKNTSIVSNPFITISQKHDNKGDSYGYMMVPNIDRTSFDKLANSKEVELLENSSKQQVIYDKNSQTWAVIKHDNQESLINNQFKMNKAGLYLVQKVGNDYQNVYYQPQTMTKTDQLAI

0000000000000000000000000000000000000000000000000000000000000000000000000000000000000000000000000000000000000000000000000000000000000000000000000000000000000000000000000000000000000000000000000000000000000000000000000000000000000000000000000000000000000000000000000000000000000000000000000000000000000000000000000000000000000000000000000000000000000000000000000000000000000000000000000000000000000000000000000000000000000000000000000000000000000000000000000000000000000000000000000000000000000000000000000000000000000000000000000000000000000001111111100000000000000000000000000000000000000000000000000000000000000000000000000000000000000000001111110000000000000000000000000000000000000000000000000000000000000000000111100000000000000000000000000000000000000000000000000000000000000000000000000000000000000000000000

>DM_train56

MKYGYFDNDNREYVITRPDVPAPWTNYLGTEKFCTVISHNAGGYSFYNSPEYNRVTKFRPNATFDRPGHYVYLRDDDSGDYWSISWQPVAKSLDEAQYQIRHGLSYSKFQCDYNGIHARKTLFVPKGEDAEIWDVVIKNTSDQVRTISAFSFVEFSFSHIQSDNQNHQMSLYSAGTAYRPGLIEYDLYYNTDDFEGFYYLASTFDPDSYDGQRDRFLGLYRDEANPLAVEQGRCSNSAQTCYNHCGSLHKQFTLQPGEEIRFAYILGIGKGNGERLREHYQDVANIDAAFAAIKAHWDERCAKFQVKSPNQGLDTMINAWTLYQAETCVVWSRFASFIEVGGRTGLGYRDTAQDAISVPHANPEMTRKRIVDLLRGQVKAGYGLHLFDPDWFDPEKEDVAPSKSPTVVPTPSDEDKIHGIKDTCSDDHLWLIPTICKYVMETGETSFFDQMIPYADGGEASVYEHMKAALDFSAEYVGQTGICKGLRADWNDCLNLGGGESSMVSFLHFWALQEFIDLAKFLGKDQDVNTYTEMAANVREACETHLWDDEGGWYIRGLTKNGDKIGTAQQQEGRVHLESNTLAVLSGLASQERGEQAMDAVDEHLFSPYGLHLNAPSFSTPNDDIGFVTRVYQGVKENGAIFSHPNPWAWVAETKLGRGDRAMKFYDALNPYNQNDIIEKRIAEPYSYVQFIMGRDHQDHGRANHPWLTGTSGWAYFAVTNYILGVQSGFTGLSVDPCIPSDWPGFEVTRQWRGATYHIQVENPDHVSKGVKSITLNGAPIQGRIPPQAQGSDNQVVVVLG

000000000000000000000000000000000000000000000000000000000000000000000000000000000000000000000000000000000000000000000000000000000000000000000000000000000000000000000000000000000000000000000000000000000000000000000000000000000000000000000000000000000000000000000000000000000000000000000000000000000000000000000000000000000000000000000000000000000000000000000000000000000000000000000000000000000011111111111111111111110000000000000000000000000000000000000000000000000000000000000000000000000000000000000000000000000000000000000000000000000000000000000000000000000000000000000000000000000000000000000000000000000000000000000000000000000000000000000000000000000000000000000000000000000000000000000000000000000000000000000000000000000000000000000000000000000000000000000000000000000000000000000000000000000

>DM_train58

MRVPFSWLKAYVPELESPEVLEERLAGLGFETDRIERVFPIPRGVVFARVLEAHPIPGTRLKRLVLDAGRTVEVVSGAENARKGIGVALALPGTELPGLGQKVGERVIQGVRSFGMALSPRELGVGEYGGGLLEFPEDALPPGTPLSEAWPEEVVLDLEVTPNRPDALGLLGLARDLHALGYALVEPEAALKAEALPLPFALKVEDPEGAPHFTLGYAFGLRVAPSPLWMQRALFAAGMRPINNVVDVTNYVMLERAQPMHAFDLRFVGEGIAVRRAREGERLKTLDGVERTLHPEDLVIAGWRGEESFPLGLAGVMGGAESEVREDTEAIALEVACFDPVSIRKTARRHGLRTEASHRFERGVDPLGQVPAQRRALSLLQALAGARVAEALLEAGSPKPPEAIPFRPEYANRLLGTSYPEAEQIAILKRLGCRVEGEGPTYRVTPPSHRLDLRLEEDLVEEVARIQGYETIPLALPAFFPAPDNRGVEAPYRKEQRLREVLSGLGFQEVYTYSFMDPEDARRFRLDPPRLLLLNPLAPEKAALRTHLFPGLVRVLKENLDLDRPERALLFEVGRVFREREETHLAGLLFGEGVGLPWAKERLSGYFLLKGYLEALFARLGLAFRVEAQAFPFLHPGVSGRVLVEGEEVGFLGALHPEIAQELELPPVHLFELRLPLPDKPLAFQDPSRHPAAFRDLAVVVPAPTPYGEVEALVREAAGPYLESLALFDLYQGPPLPEGHKSLAFHLRFRHPKRTLRDEEVEEAVSRVAEALRARGFGLRGLDTP

00000000000000000000000000000000000000000000000000000000000000000000000000000000000000000000000000000000000000000000000000000000000000000000000000000000000000000000000000000000000000000000000000000000000000000000000000000000000000000000000000000000000000000000000000000000000000000000000000000000000000000000000000000000000000000000000000000000000000000000000000000000000000000000000000000000000000000000000000000000000000000000000000000000000000000000000000000000000000000000000000000000000000000000000000000000000000000000000000000000000000000000000000000000000000000000000000000000000000000000000000000000000000000000000000000000000000000000000000000000000000000000000000000000000000000000000000000000000000000000000000000000000000000000000000000000000000000000000000000001111111111

>DM_train59

MRFKNVKKTALMLAMFGMATSSNAALFDYNATGDTEFDSPAKQGWMQDNTNNGSGVLTNADGMPAWLVQGIGGRAQWTYSLSTNQHAQASSFGWRMTTEMKVLSGGMITNYYANGTQRVLPIISLDSSGNLVVEFEGQTGRTVLATGTAATEYHKFELVFLPGSNPSASFYFDGKLIRDNIQPTASKQNMIVWGNGSSNTDGVAAYRDIKFEIQGDVIFRGPDRIPSIVASSVTPGVVTAFAEKRVGGGDPGALSNTNDIITRTSRDGGITWDTELNLTEQINVSDEFDFSDPRPIYDPSSNTVLVSYARWPTDAAQNGDRIKPWMPNGIFYSVYDVASGNWQAPIDVTDQVKERSFQIAGWGGSELYRRNTSLNSQQDWQSNAKIRIVDGAANQIQVADGSRKYVVTLSIDESGGLVANLNGVSAPIILQSEHAKVHSFHDYELQYSALNHTTTLFVDGQQITTWAGEVSQENNIQFGNADAQIDGRLHVQKIVLTQQGHNLVEFDAFYLAQQTPEVEKDLEKLGWTKIKTGNTMSLYGNASVNPGPGHGITLTRQQNISGSQNGRLIYPAIVLDRFFLNVMSIYSDDGGSNWQTGSTLPIPFRWKSSSILETLEPSEADMVELQNGDLLLTARLDFNQIVNGVNYSPRQQFLSKDGGITWSLLEANNANVFSNISTGTVDASITRFEQSDGSHFLLFTNPQGNPAGTNGRQNLGLWFSFDEGVTWKGPIQLVNGASAYSDIYQLDSENAIVIVETDNSNMRILRMPITLLKQKLTLSQN

1111111111111111111111110000000000000000000000000000000000000000000000000000000000000000000000000000000000000000000000000000000000000000000000000000000000000000000000000000000000000000000000000000000000000000000000000000000000000000000000000000000000000000000000000000000000000000000000000000000000000000000000000000000000000000000000000000000000000000000000000000000000000000000000000000000000000000000000000000000000000000000000000000000000000000000000000000000000000000000000000000000000000000000000000000000000000000000000000000000000000000000000000000000000000000000000000000000000000000000000000000000000000000000000000000000000000000000000000000000000000000000000000000000000000000000000000000000000000000000000000000000000000000000000000000000000000000000000000000000001111

>DM_train60

AGGHGDVGMHVKEKEKNKDENKRKDEERNKTQEEHLKEIMKHIVKIEVKGEEAVKKEAAEKLLEKVPSDVLEMYKAIGGKIYIVDGDITKHISLEALSEDKKKIKDIYGKDALLHEHYVYAKEGYEPVLVIQSSEDYVENTEKALNVYYEIGKILSRDILSKINQPYQKFLDVLNTIKNASDSDGQDLLFTNQLKEHPTDFSVEFLEQNSNEVQEVFAKAFAYYIEPQHRDVLQLYAPEAFNYMDKFNEQEINLSLEELKDQRMLSRYEKWEKIKQHYQHWSDSLSEEGRGLLKKLQIPIEPKKDDIIHSLSQEEKELLKRIQIDSSDFLSTEEKEFLKKLQIDIRDSLSEEEKELLNRIQVDSSNPLSEKEKEFLKKLKLDIQPYDINQRLQDTGGLIDSPSINLDVRKQYKRDIQNIDALLHQSIGSTLYNKIYLYENMNINNLTATLGADLVDSTDNTKINRGIFNEFKKNFKYSISSNYMIVDINERPALDNERLKWRIQLSPDTRAGYLENGKLILQRNIGLEIKDVQIIKQSEKEYIRIDAKVVPKSKIDTKIQEAQLNINQEWNKALGLPKYTKLITFNVHNRYASNIVESAYLILNEWKNNIQSDLIKKVTNYLVDGNGRFVFTDITLPNIAEQYTHQDEIYEQVHSKGLYVPESRSILLHGPSKGVELRNDSEGFIHEFGHAVDDYAGYLLDKNQSDLVTNSKKFIDIFKEEGSNLTSYGRTNEAEFFAEAFRLMHSTDHAERLKVQKNAPKTFQFINDQIKFIINS

11111111111111111111111111000000000000000000000000000000000000000000000000000000000000000000000000000000000000000000000000000000000000000000000000000000000000000000000000000000000000000000000000000000000000000000000000000000000000000000000000000000000000000000000000000000000000000000000000000000000000000000000000000000000000000000000000000000011111111111111111111110000000000000000000000000000000000000000000000000000000000000000000000000000000000000000000000000000000000000000000000000000000000000000000000000000000000000000000000000000000000000000000000000000000000000000000000000000000000000000000000000000000000000000000000000000000000000000000000000000000000000000000000000000000000000000000000000000000000000000000000000000000000000000000000000000000000000000000000111

>DM_train62

ANADNYKNVINRTGAPQYMKDYDYDDHQRFNPFFDLGAWHGHLLPDGPNTMGGFPGVALLTEEYINFMASNFDRLTVWQDGKKVDFTLEAYSIPGALVQKLTAKDVQVEMTLRFATPRTSLLETKITSNKPLDLVWDGELLEKLEAKEGKPLSDKTIAGEYPDYQRKISATRDGLKVTFGKVRATWDLLTSGESEYQVHKSLPVQTEINGNRFTSKAHINGSTTLYTTYSHLLTAQEVSKEQMQIRDILARPAFYLTASQQRWEEYLKKGLTNPDATPEQTRVAVKAIETLNGNWRSPGGAVKFNTVTPSVTGRWFSGNQTWPWDTWKQAFAMAHFNPDIAKENIRAVFSWQIQPGDSVRPQDVGFVPDLIAWNLSPERGGDGGNWNERNTKPSLAAWSVMEVYNVTQDKTWVAEMYPKLVAYHDWWLRNRDHNGNGVPEYGATRDKAHNTESGEMLFTVKKGDKEETQSGLNNYARVVEKGQYDSLEIPAQVAASWESGRDDAAVFGFIDKEQLDKYVANGGKRSDWTVKFAENRSQDGTLLGYSLLQESVDQASYMYSDNHYLAEMATILGKPEEAKRYRQLAQQLADYINTCMFDPTTQFYYDVRIEDKPLANGCAGKPIVERGKGPEGWSPLFNGAATQANADAVVKVMLDPKEFNTFVPLGTAALTNPAFGADIYWRGRVWVDQFWFGLKGMERYGYRDDALKLADTFFRHAKGLTADGPIQENYNPLTGAQQGAPNFSWSAAHLYMLYNDFFRKQ

11000000000000000000000000000000000000000000000000000000000000000000000000000000000000000000000000000000000000000000000000000000000000000000000000000000000000000000000000000000000000000000000000000000000000000000000000000000000000000000000000000000000000000000000000000000000000000000000000000000000000000000000000000000000000000000000000000000000000000000000000000000000000000000000000000000000000000000000000000000000000000000000000000000000000000000000000000000000000000000000000000000000000000000000000000000000000000000000000000000000000000000000000000000000000000000000000000000000000000000000000000000000000000000000000000000000000000000000000000000000000000000000000000000000000000000000000000000000000000000000000000000000000000000000000000000000000001

>DM_train63

MLNQELELSLNMAFARAREHRHEFMTVEHLLLALLSNPSAREALEACSVDLVALRQELEAFIEQTTPVLPASEEERDTQPTLSFQRVLQRAVFHVQSSGRNEVTGANVLVAIFSEQESQAAYLLRKHEVSRLDVVNFISHGTRKDEPTQSSDPGSQPNSEEQAGGEERLENFTTNLNQLARVGGIDPLIGREKELERAIQVLCRRRKNNPLLVGESGVGKTAIAEGLAWRIVQGDVPEVMADCTIYSLDIGSLLAGTKYRGDFEKRFKALLKQLEQDTNSILFIDEIHTIIGAGAASGGQVDAANLIKPLLSSGKIRVIGSTTYQEFSNIFEKDRALARRFQKIDITEPSIEETVQIINGLKPKYEAHHDVRYTAKAVRAAVELAVKYINDRHLPDKAIDVIDEAGARARLMPVSKRKKTVNVADIESVVARIARIPEKSVSQSDRDTLKNLGDRLKMLVFGQDKAIEALTEAIKMARAGLGHEHKPVGSFLFAGPTGVGKTEVTVQLSKALGIELLRFDMSEYMERHTVSRLIGAPPGYVGFDQGGLLTDAVIKHPHAVLLLDEIEKAHPDVFNILLQVMDNGTLTDNNGRKADFRNVVLVMTTNAGVRETERKSIGLIHQDNSTDAMEEIKKIFTPEFRNRLDNIIWFDHLSTDVIHQVVDKFIVELQVQLDQKGVSLEVSQEARNWLAEKGYDRAMGARPMARVIQDNLKKPLANELLFGSLVDGGQVTVALDKEKNELTYGFQSAQKHKAEAAH

00000000000000000000000000000000000000000000000000000000000000000000000000000000000000000000000000000000000000000000000000000000000000000000001111111111111111111111111000000000000000000000000000000000000000000000000000000000000000000000000000000000000111000000000000000000000000000000000000000000000000000000000000000000000000000000000000000000000000000000000000000000000000000000000000000000000000000000000000000000000000000000000000000000000000000000000000000000000000000000000000000000000000000000000000000000000000000000000000000000000000000000000000000000000000000000000000000000000000000000000000000000001111111111111000000000000000000000000000000000000000000000000000000000000000000000000000000000000000000000000000000000000000000000000000000000000111

>DM_train66

KDWTQYVNPLMGSQSTFELSTGNTYPAIARPWGMNFWTPQTGKMGDGWQYTYTANKIRGFKQTHQPSPWINDYGQFSIMPIVGQPVFDEEKRASWFAHKGEVATPYYYKVYLAEHDIVTEMTPTERAVLFRFTFPENDHSYVVVDAFDKGSYIKIIPEENKIIGYTTRNSGGVPENFKNYFIIEFDKPFTYKATVENGNLQENVAEQTTDHAGAIIGFKTRKGEQVNARIASSFISFEQAAANMNELGKDNIEQLAQKGKDAWNQVLGKIEVEGGNLDQYRTFYSCLYRSLLFPRKFYELDANGQPIHYSPYNGQVLPGYMFTDTGFWDTFRCLFPLLNLMYPSVNKEMQEGLINTYLESGFFPEWASPGHRGCMVGNNSASILVDAYMKGVKVDDIKTLYEGLIHGTENVHPEVSSTGRLGYEYYNKLGYVPYDVKINENAARTLEYAYDDWCIYRLAKELKRPKKEISLFAKRAMNYKNLFDKESKLMRGRNEDGTFQSPFSPLKWGDAFTEGNSWHYTWSVFHDPQGLIDLMGGKEMFVTMMDSVFAVPPIFDDSYYGQVIHEIREMTVMNMGNYAHGNQPIQHMIYLYDYAGQPWKAQYWLRQVMDRMYTPGPDGYCGDEDNGQTSAWYVFSALGFYPVCPGTDEYVMGTPLFKKATLHFENGNSLVIDAPNNSTENFYIDSMSFNGADHTKNYLRHEDLFKGGTIKVDMSNRPNLNRGTKEEDMPYSFSKELEHHHHHH

000000000000000000000000000000000000000000000000000000000000000000000000000000000000000000000000000000000000000000000000000000000000000000000000000000000000000000000000000000000000000000000000000000000000000000000000000000000000000000000000000000000000000000000000000000000000000000000000000000000000000000000000000000000000000000000000000000000000000000000000000000000000000000000000000000000000000000000000000000000000000000000000000000000000000000000000000000000000000000000000000000000000000000000000000000000000000000000000000000000000000000000000000000000000000000000000000000000000000000000000000000000000000000000000000000000000000000000000000000000000000000000000000000000000000000000000000000000000000000000000000000000000000011111111

>DM_train67

MTDRVSVGNLRIARVLYDFVNNEALPGTDIDPDSFWAGVDKVVADLTPQNQALLNARDELQAQIDKWHRRRVIEPIDMDAYRQFLTEIGYLLPEPDDFTITTSGVDAEITTTAGPQLVVPVLNARFALNAANARWGSLYDALYGTDVIPETDGAEKGPTYNKVRGDKVIAYARKFLDDSVPLSSGSFGDATGFTVQDGQLVVALPDKSTGLANPGQFAGYTGAAESPTSVLLINHGLHIEILIDPESQVGTTDRAGVKDVILESAITTIMDFEDSVAAVDAADKVLGYRNWLGLNKGDLAAAVDKDGTAFLRVLNRDRNYTAPGGGQFTLPGRSLMFVRNVGHLMTNDAIVDTDGSEVFEGIMDALFTGLIAIHGLKASDVNGPLINSRTGSIYIVKPKMHGPAEVAFTCELFSRVEDVLGLPQNTMKIGIMDEERRTTVNLKACIKAAADRVVFINTGFLDRTGDEIHTSMEAGPMVRKGTMKSQPWILAYEDHNVDAGLAAGFSGRAQVGKGMWTMTELMADMVETKIAQPRAGASTAWVPSPTAATLHALHYHQVDVAAVQQGLAGKRRATIEQLLTIPLAKELAWAPDEIREEVDNNCQSILGYVVRWVDQGVGCSKVPDIHDVALMEDRATLRISSQLLANWLRHGVITSADVRASLERMAPLVDRQNAGDVAYRPMAPNFDDSIAFLAAQELILSGAQQPNGYTEPILHRRRREFKARAAEKPAPSDRAGDDAAR

000000000000000000000000000000000000000000000000000000000000000000000011111100000000000000000000000000000000000000000000000000000000000000000000000000000000000000000000000000000000000000000000000000000000000000000000000000000000000000000000000000000000000000000000000000000000000000000000000000000000011111111110000000000000000000000000000000000000000000000000000000000000000000001111100000000000000000000000000000000000000000000000000000000000000000000000000000000000000000000000000000000000000000000000000000000000000000000000000000000000000000000000000000000000000000000000000000000000000000000000000000000000000000000000000000000000000000000000000000000000000000000000011110000000000000000000000000000000000000000000000000011111111111111

>DM_train68

MSEEISLSAEFIDRVKASVKPHWGKLGWVTYKRTYARWLPEKGRSENWDETVKRVVEGNINLDPRLQDSPSLELKQSLTEEAERLYKLIYGLGATPSGRNLWISGTDYQRRTGDSLNNCWFVAIRPQKYGDSKIVPSYLGKQEKAVSMPFSFLFDELMKGGGVGFSVARSNISQIPRVDFAIDLQLVVDETSESYDASVKVGAVGKNELVQDADSIYYRLPDTREGWVLANALLIDLHFAQTNPDRKQKLILDLSDIRPYGAEIHGFGGTASGPMPLISMLLDVNEVLNNKAGGRLTAVDAADICNLIGKAVVAGNVRRSAELALGSNDDQDFISMKQDQEKLMHHRWASNNSVAVDSAFSGYQPIAAGIRENGEPGIVNLDLSKNYGRIVDGYQAGIDGDVEGTNPCGEISLANGEPCNLFEVFPLIAEEQGWDLQEVFALAARYAKRVTFSPYDWEISREIIQKNRRIGISMSGIQDWLLTRLGNRVVTGFKDDFDPETHEAIKVPVYDKRAIKMVDQLYKAVVKADQDYSKTLGCNESIKHTTVKPSGTVAKLAGASEGMHFHYGAYLIQRIRFQDSDPLLPALKACGYRTEADIYTENTTCVEFPIKAVGADNPNFASAGTVSIAEQFATQAFLQTYWSDNAVSCTITFQDSEGDQVESLLRQYRFITKSTSLLPYFGGSLQQAPKEPIDKETYEKRSQEITGNVEEVFSQLNSDVKDLELVDQTDCEGGACPIK

1110000000000000000000000000000000000000000000000000000000000000000000000000000000000000000000000000000000000000000000000000000000000000000000000000000000000000000000000000000000000000000000000000000000000000000000000000000000000000000000000000000000000000000000000000000000000000000000000000000000000000000000000000111100000000000000000000000000000000000000000000000000000000000000000000000000000000000000000000000000000000000000000000000000000000000000000000000000000000000000000000000000000000000000000000000000000000000000000000000000000000000000000000000000000000000000000000000000000000000000000000000000000000000000000000000000000000000000000000000000000000000000000000000000000000000000000000000000000000000000000000111111111111111

>DM_train69

EVKQENRLLNESESSSQGLLGYYFSDLNFQAPMVVTSSTTGDLSIPSSELENIPSENQYFQSAIWSGFIKVKKSDEYTFATSADNHVTMWVDDQEVINKASNSNKIRLEKGRLYQIKIQYQRENPTEKGLDFKLYWTDSQNKKEVISSDNLQLPELKQKSSNSRKKRSTSAGPTVPDRDNDGIPDSLEVEGYTVDVKNKRTFLSPWISNIHEKKGLTKYKSSPEKWSTASDPYSDFEKVTGRIDKNVSPEARHPLVAAYPIVHVDMENIILSKNEDQSTQNTDSETRTISKNTSTSRTHTSEVHGNAEVHASFFDIGGSVSAGFSNSNSSTVAIDHSLSLAGERTWAETMGLNTADTARLNANIRYVNTGTAPIYNVLPTTSLVLGKNQTLATIKAKENQLSQILAPNNYYPSKNLAPIALNAQDDFSSTPITMNYNQFLELEKTKQLRLDTDQVYGNIATYNFENGRVRVDTGSNWSEVLPQIQETTARIIFNGKDLNLVERRIAAVNPSDPLETTKPDMTLKEALKIAFGFNEPNGNLQYQGKDITEFDFNFDQQTSQNIKNQLAELNATNIYTVLDKIKLNAKMNILIRDKRFHYDRNNIAVGADESVVKEAHREVINSSTEGLLLNIDKDIRKILSGYIVEIEDTEGLKEVINDRYDMLNISSLRQDGKTFIDFKKYNDKLPLYISNPNYKVNVYAVTKENTIINPSENGDTSTNGIKKILIFSKKGYEIG

111111111111100000000000000000000000000000000000000000000000000000000000000000000000000000000000001111000000000000000000000000000000000000000000000000000000000001111111111111000000000000000000000000000000000000000000000000000000000000000000000000000000000000000000000000000001111111111110000000000000000111111111111111100000000000000000000000111111110000000000000000000000000000000000000000000000000000000000000000000000000000000000000000000000000000000000000000000000000000000000000000000000000000000000000000011110000000000000000000000000000000000000000000000000000000000000000000000000000000000000000000000000000000000000000000000000000000000000000000000000000000000000000000000000000000000000000000000000000000000000000000000000000

>DM_train73

MAPSLDSISHSFANGVASAKQAVNGASTNLAVAGSHLPTTQVTQVDIVEKMLAAPTDSTLELDGYSLNLGDVVSAARKGRPVRVKDSDEIRSKIDKSVEFLRSQLSMSVYGVTTGFGGSADTRTEDAISLQKALLEHQLCGVLPSSFDSFRLGRGLENSLPLEVVRGAMTIRVNSLTRGHSAVRLVVLEALTNFLNHGITPIVPLRGTISASGDLSPLSYIAAAISGHPDSKVHVVHEGKEKILYAREAMALFNLEPVVLGPKEGLGLVNGTAVSASMATLALHDAHMLSLLSQSLTAMTVEAMVGHAGSFHPFLHDVTRPHPTQIEVAGNIRKLLEGSRFAVHHEEEVKVKDDEGILRQDRYPLRTSPQWLGPLVSDLIHAHAVLTIEAGQSTTDNPLIDVENKTSHHGGNFQAAAVANTMEKTRLGLAQIGKLNFTQLTEMLNAGMNRGLPSCLAAEDPSLSYHCKGLDIAAAAYTSELGHLANPVTTHVQPAEMANQAVNSLALISARRTTESNDVLSLLLATHLYCVLQAIDLRAIEFEFKKQFGPAIVSLIDQHFGSAMTGSNLRDELVEKVNKTLAKRLEQTNSYDLVPRWHDAFSFAAGTVVEVLSSTSLSLAAVNAWKVAAAESAISLTRQVRETFWSAASTSSPALSYLSPRTQILYAFVREELGVKARRGDVFLGKQEVTIGSNVSKIYEAIKSGRINNVLLKMLA

11111111111111111111111110000000111111000000000000000000000000000000000000000000000000000000000000000001111111111111111111100000000000000000000000000000000000000000000000000000000000000000000000000000000000000000000000000000000000000000000000000000000000000000000000000000000000000000000000000000000000000000000000000000000000000000000000000000000001111000000000000000000000000000000000000000000000000000000000000000000000000000000000000000000000000000000000000000000000000000000000000000000000000000000000000000000000000000000000000000000000000000000000000000000000000000000000000000000000000000000000000000000000000000000000000000000000000000000000000000000000000000000000000000000000000000000000000000000000000000

>DM_train74

QNVSLQPPPQQLIVQNKTIDLPAVYQLNGGEEANPHAVKVLKELLSGKQSSKKGMLISIGEKGDKSVRKYSRQIPDHKEGYYLSVNEKEIVLAGNDERGTYYALQTFAQLLKDGKLPEVEIKDYPSVRYRGVVEGFYGTPWSHQARLSQLKFYGKNKMNTYIYGPKDDPYHSAPNWRLPYPDKEAAQLQELVAVANENEVDFVWAIHPGQDIKWNKEDRDLLLAKFEKMYQLGVRSFAVFFDDISGEGTNPQKQAELLNYIDEKFAQVKPDINQLVMCPTEYNKSWSNPNGNYLTTLGDKLNPSIQIMWTGDRVISDITRDGISWINERIKRPAYIWWNFPVSDYVRDHLLLGPVYGNDTTIAKEMSGFVTNPMEHAESSKIAIYSVASYAWNPAKYDTWQTWKDAIRTILPSAAEELECFAMHNSDLGPNGHGYRREESMDIQPAAERFLKAFKEGKNYDKADFETLQYTFERMKESADILLMNTENKPLIVEITPWVHQFKLTAEMGEEVLKMVEGRNESYFLRKYNHVKALQQQMFYIDQTSNQNPYQPGVKTATRVIKPLIDRTFATVVKFFNQKFNAHLDATTDYMPHKMISNVEQIKNLPLQVKANRVLISPANEVVKWAAGNSVEIELDAIYPGENIQINFGKDAPCTWGRLEISTDAKEWKTVDLKQKESRLSAGLQKAPVKFVRFTNVSDEEQQVYLRQFVLTIEKK

11110000000000000000000000000000000000000000011111111000000000000000000000000000000000000000000000000000000000000000000000000000000000000000000000000000000000000000000000000000000000000000000000000000000000000000000000000000000000000000000000000000000000000000000000000000000000000000000000000000000000000000000000000000000000000000000000000000000000000000000000000000000000000000000000000000000000000000000000000000000000000000000000000000000000000000000000000000000000000000000000000000000000000000000000000000000000000000000000000000000000000000000000000000000000000000000000000000000000000001111111100000000000000011111111111100000000000000000011111111111111111111111111111100000000000000001111111111111100000001

>DM_train75

GVTVIPRLLGLKDEKKIATTVGEARLSGINYRHPDSALVSYPVAAAAPLGRLPAGNYRIAIVGGGAGGIAALYELGRLAATLPAGSGIDVQIYEADPDSFLHDRPGIKAIKVRGLKAGRVSAALVHNGDPASGDTIYEVGAMRFPEIAGLTWHYASAAFGDAAPIKVFPNPGKVPTEFVFGNRVDRYVGSDPKDWEDPDSPTLKVLGVVAGGLVGNPQGENVAMYPIANVDPAKIAAILNAATPPADALERIQTKYWPEFIAQYDGLTLGAAVREIVTVAFEKGTLPPVDGVLDVDESISYYVELFGRFGFGTGGFKPLYNISLVEMMRLILWDYSNEYTLPVTENVEFIRNLFLKAQNVGAGKLVVQVRQERVANACHSGTASARAQLLSYDSHNAVHSEAYDFVILAVPHDQLTPIVSRSGFEHAASQNLGDAGLGLETHTYNQVYPPLLLSDSSPAANARIVTAIGQLHMARSSKVFATVKTAALDQPWVPQWRGEPIKAVVSDSGLAASYVVPSPIVEDGQAPEYSSLLASYTWEDDSTRLRHDFGLYPQNPATETGTADGMYRTMVNRAYRYVKYAGASNAQPWWFYQLLAEARTADRFVFDWTTNKTAGGFKLDMTGDHHQSNLCFRYHTHALAASLDNRFFIASDSYSHLGGWLEGAFMSALNAVAGLIVRANRGDVSALSTEARPLVIGLRPVVKVPAAELATSQ

11111111111111100000000000000000000000000000000000000000000000000000000000000000000000000000000000000000111100000000000000000000000000000000000000000000000000000000000000000000000000000000000000000000000000000000000000000000000000000000000000000000000000000000000000000000000000000000000000000000000000000000000000000000000000000000000000000000000000000000000000000000000000000000000000000000000000000000000000000000000000000000000000000000000000000000000000000000000000000000000000000000000000000000000000000000000000000111100000000000000000000000000000000000000000000000000000000000000000000000000000000000000000000000000000000000000000000000000000000000000000000000000000000000000000000000000000000000000111111

>DM_train77

MKKLFVTCIVFFSILSPALLIAQQTGTAELIMKRVMLDLKKPLRNMDKVAEKNLNTLQPDGSWKDVPYKDDAMTNWLPNNHLLQLETIIQAYIEKDSHYYGDDKVFDQISKAFKYWYDSDPKSRNWWHNEIATPQALGEMLILMRYGKKPLDEALVHKLTERMKRGEPEKKTGANKTDIALHYFYRALLTSDEALLSFAVKELFYPVQFVHYEEGLQYDYSYLQHGPQLQISSYGAVFITGVLKLANYVRDTPYALSTEKLAIFSKYYRDSYLKAIRGSYMDFNVEGRGVSRPDILNKKAEKKRLLVAKMIDLKHTEEWADAIARTDSTVAAGYKIEPYHHQFWNGDYVQHLRPAYSFNVRMVSKRTRRSESGNKENLLGRYLSDGATNIQLRGPEYYNIMPVWEWDKIPGITSRDYLTDRPLTKLWGEQGSNDFAGGVSDGVYGASAYALDYDSLQAKKAWFFFDKEIVCLGAGINSNAPENITTTLNQSWLNGPVISTAGKTGRGKITTFKAQGQFWLLHDAIGYYFPEGANLSLSTQSQKGNWFHINNSHSKDEVSGDVFKLWINHGARPENAQYAYIVLPGINKPEEIKKYNGTAPKVLANTNQLQAVYHQQLDMVQAIFYTAGKLSVAGIEIETDKPCAVLIKHINGKQVIWAADPLQKEKTAVLSIRDLKTGKTNRVKIDFPQQEFAGATVELK

1111111111111111111111111000000000000000000000000000000000000000000000000000000000000000000000000000000000000000000000000000000000000000000000000000000000000000000000000000000000000000000000000000000000000000000000000000000000000000000000000000000000000000000000000000000000000000000000000000000000000000000000000000000000000000000000000000000000000000000000000000000000000000000000000000000000000000000000000000000000000000000000000000000000000000000000000000000000000000000000000000000000000000000000000000000000000000000000000000000000000000000000000000000000000000000000000000000000000000000000000000000000000000000000000000000000000000000000000000000000000000000000000000000000000000000000000000

>DM_train79

LMPGGKEFYNFYPEYVVGLQWMGDNYVFIEGDDLVFNKANGKSAQTTRFSAADLNALMPEGCKFQTTDAFPSFRTLDAGRGLVVLFTQGGLVGFDMLARKVTYLFDTNEETASLDFSPVGDRVAYVRNHNLYIARGGKLGEGMSRAIAVTIDGTETLVYGQAVHQREFGIEKGTFWSPKGSCLAFYRMDQSMVKPTPIVDYHPLEAESKPLYYPMAGTPSHHVTVGIYHLATGKTVYLQTGEPKEKFLTNLSWSPDENILYVAEVNRAQNECKVNAYDAETGRFVRTLFVETDKHYVEPLHPLTFLPGSNNQFIWQSRRDGWNHLYLYDTTGRLIRQVTKGEWEVTNFAGFDPKGTRLYFESTEASPLERHFYCIDIKGGKTKDLTPESGMHRTQLSPDGSAIIDIFQSPTVPRKVTVTNIGKGSHTLLEAKNPDTGYAMPEIRTGTIMAADGQTPLYYKLTMPLHFDPAKKYPVIVYVYGGPHAQLVTKTWRSSVGGWDIYMAQKGYAVFTVDSRGSANRGAAFEQVIHRRLGQTEMADQMCGVDFLKSQSWVDADRIGVHGWSYGGFMTTNLMLTHGDVFKVGVAGGPVIDWNRYAIMYGERYFDAPQENPEGYDAANLLKRAGDLKGRLMLIHGAIDPVVVWQHSLLFLDACVKARTYPDYYVYPSHEHNVMGPDRVHLYETITRYFTDHL

1111111111111110000000000000000000000011111110000000000000111111111110000000000000000000000000000000000000000000000000000000000000000000000000000000000000000000000000000000000000000000000000000000000000000000000000000000000000000000000000000000000000000000000000000000000000000000000000000000000000000000000000000000000000000000000000000000000000000000000000000000000000000000000000000000000000000000000000000000000000000000000000001111110000000000000000000000000000000000000000000000000000001111000000000000000000000000000000000000000000000000000000000000000000000000000000000000000000000000000000000000000000000000000000000000000000000000000000000000000000000000000000000000000000000000000000

>DM_train84

VLSGCSNNVSSIKIDRFNNISAVNGPGEEDTWASAQKQGVGTANNYVSKVWFTLANGAISEVYYPTIDTADVKEIKFIVTDGKSFVPDETKDAISKVEKFTDKSLGYKLVNTDKKGRYRITKDIFTDVKRNSLIMKAKFEALEGSIHDYKLYLAYDPHIKNQGSYNEGYVIKANNNEMLMAKRDNVYTALSSNIGWKGYSIGYYKVNDIMTDLDENKQMTKHYDSARGNIIEGAEIDLTKNSEFEIVLSFGQSDSEAAKTALETLGEDYNNLKNNYIDEWTKYCNTLNNFNGKANSLYYNSMMILKASEDKTNKGAYIASLSIPWGDGQRDDNTGGYHLVWSRDLYHVANAFIAAGDVDSANRSLDYLAKVVKDNGMIPQNTWISGKPYWTGIQLDEQADPIILSYRLKRYDLYDSLVKPLADFIIKIGPKTGQERWEEIGGYSPATMAAEVAGLTCAAYIAEQNKDYESAQKYQEKADNWQKLIDNLTYTENGPLGNGQYYIRIAGLSDPDADFMINIANGGGVYDQKEIVDPSFLELVRLGVKSADDPKILNTLKVVDSTIKVDTPKGPSWYRYNHDGYGEPSKTELYHGAGKGRLWPLLTGERGMYEIAAGKDATPYVKAMEKFANEGGIISEQVWEDTGLPTDSASPLNWAHAEYVILFASNIEHKVLDMPDIVYKRYVA

111111111100000000000000000000000000000000000000000000000000000000000000000000000000000000000000000000000000000000000000000000000000000000000000000000000000000000000000000000000000000000000000000000000000000000000000000000000000000000000000000000000000000000000000000000000000000000000000000000000000000000000000000000000000000000000000000000000000000000000000000000000000000000000000000000000000000000000000000000000000000000000000000000000000000000000000000000000000000000000000000000000000000000000000000000000000000000000000000000000000000000000000000000000000000000000000000000000000000000000000000000000000000000000000000000000000000000000000000000000000000000000000000000000000

>DM_train85

TDAKNNLLYFFDRPNEPCFMQKGEDKVVFEIPDHYYPDKYKSLSNTLSNRFGNEATKRIPIRNITLPNLEVPMQLPYNDQFSLFVPKHRTMAAKLIDIFMGMRDVEDLQSVCSYCQLRINPYMFNYCLSVAILHRPDTKGLSIPTFAETFPDKFMDSKVFLRAREVSNVVISGSRMPVNVPINYTANTTEPEQRVAYFREDIGINLHHWHWHLVYPFDSADRSIVNKDRRGELFYYMHQQIIGRYNVERMCNGLPQVKPFSDFSAPIEEGYFPKLDSQVASRTWPPRFAGSVFRNLDRTVDQVKIDVRKLFTWRDQFLEAIQKMAIKMPNGRELPLDEVTGIDMLGNLMESSIISPNRGYYGDLHNMGHVFAAYTHDPDHRHLEQFGVMGDSATAMRDPFFYRWHRFVDDVFNIYKEKLTPYTNERLDFPGVRVSSVGIEGARPNTLRTLWQQSTVELGRGLDFTPRGSVLARFTHLQHDEFQYVIEVNNTTGGNLMGTVRIFMAPKVDDNGQPMSFNKQRRLMIELDKFSQALRPGTNTIRRRSVDSSVTIPYERTFRNQSERPGDPGTAGAAEFDFCGCGWPHHMLIPKGTAQGYPVVLFVMISNWNNDRIEQDLVGSCNDAASYCGIRDRKYPDKQAMGYPFDRKMANDAATLSDFLRPNMAVRDCSIQFSDTTVERGQQG

100000000000000000000000000000000000000000000000000000000000000000000000000000000000000000000000000000000000000000000000000000000000000000000000000000000000000000000000000000000000000000000000000000000000000000000000000000000000000000000000000000000000000000000000000000000000000000000000000000000000000000000000000000000000000000000000000000000000000000000000000000000000000000000000000000000000000000000000000000000000000000000000000000000100000000000000000000000000000000000000000000000000000000000000000000000000000000000000000000000000000000000000000001111111111111111111000000000000000000000000000000000000000011000000000000000000000000000000000000000000000000000000000000011111

>DM_train86

MLRFVTKNSQDKSSDLFSICSDRGTFVAHNRVRTDFKFDNLVFNRVYGVSQKFTLVGNPTVCFNEGSSYLEGIAKKYLTLDGGLAIDNVLNELRSTCGIPGNAVASHAYNITSWRWYDNHVALLMNMLRAYHLQVLTEQGQYSAGDIPMYHDGHVKIKLPVTIDDTAGPTQFAWPSDRSTDSYPDWAQFSESFPSIDVPYLDVRPLTVTEVNFVLMMMSKWHRRTNLAIDYEAPQLADKFAYRHALTVQDADEWIEGDRTDDQFRPPSSKVMLSALRKYVNHNRLYNQFYTAAQLLAQIMMKPVPNCAEGYAWLMHDALVNIPKFGSIRGRYPFLLSGDAALIQATALEDWSAIMAKPELVFTYAMQVSVALNTGLYLRRVKKTGFGTTIDDSYEDGAFLQPETFVQAALACCTGQDAPLNGMSDVYVTYPDLLEFDAVTQVPITVIEPAGYNIVDDHLVVVGVPVACSPYMIFPVAAFDTANPYCGNFVIKAANKYLRKGAVYDKLEAWKLAWALRVAGYDTHFKVYGDTHGLTKFYADNGDTWTHIPEFVTDGDVMEVFVTAIERRARHFVELPRLNSPAFFRSVEVSTTIYDTHVQAGAHAVYHASRINLDYVKPVSTGIQVINAGELKNYWGSVRRTQQGLGVVGLTMPAVMPTGEPTAGAAHEELIEQADNVLVE

00000000000000000000000000000000000000000000000000000000000000000000000000000000000000000000000000000000000000000000000000000000000000000000000000000000000000000000000000000000000000000000000000000000000000000000000000000000000000000000000000000000000000000000000000000000000000000000000000000000000000000000000000000000000000000000000000000000000000000000000000000000000000000000000000000000000000000000000000000000000000000000000000000000000000000000000000000000000000000000000000000000000000000000000000000000000000000000000000000000000000000000000000000000000000000000000000000000000000000000000000000000000000000000000000000000000000000000000000011111111111111111111111111111

>DM_train87

MTAGYEPCWLRYERKDQYSRLRFEEIVAKRTSPIFQAAVEELQKGLRSMMEIEPQVVQEVNETANSIWLGTLEDEEFERPLEGTLVHPEGYVIRSDVDDGPFRIYIIGKTDAGVLYGVFHFLRLLQMGENIAQLSIIEQPKNRLRMINHWDNMDGSIERGYAGRSIFFVDDQFVKQNQRIKDYARLLASVGINAISINNVNVHKTETKLITDHFLPDVAEVADIFRTYGIKTFLSINYASPIEIGGLPTADPLDPEVRWWWKETAKRIYQYIPDFGGFVVKADSEFRPGPFTYGRDHAEGANMLAEALAPFGGLVIWRCFVYNCQQDWRDRTTDRAKAAYDHFKPLDGQFRENVILQIKNGPMDFQVREPVSPLFGAMPKTNQMMEVQITQEYTGQQKHLCFLIPQWKEVLDFDTYAKGKGSEVKKVIDGSLFDYRYSGIAGVSNIGSDPNWTGHTLAQANLYGFGRLAWNPDLSAEEIANEWVVQTFGDDSQVVETISWMLLSSWRIYENYTSPLGVGWMVNPGHHYGPNVDGYEYSHWGTYHYADRDGIGVDRTVATGTGYTAQYFPENAAMYESLDTCPDELLLFFHHVPYTHRLHSGETVIQHIYNTHFEGVEQAKQLRKRWEQLKGKIDEKRYHDVLERLTIQVEHAKEWRDVINTYFYRKSGIDDQYGRKIYR

1111000000000000000000000000000000000000000000000000000000000000000000000000000000000000000000000000000000000000000000000000000000000000000000000000000000000000000000000000000000000000000000000000000000000000000000000000000000000000000000000000000000000000000000000000000000000000001111100000000000000000000000000000000000000000000000000000000000000000000000000000000000000000000000000000000000000000000000000000000000000000000000000000000000000000000000000000000000000000000000000000000000000000000000000000000000000000000000000000000000000000000000000000000000000000000000000000000000000000000000000000000000000000000000000000000000000000000000000000000000000000000000000000000

>DM_train88

SAPGEDEECGRVRDFVAKLANNTHQHVFDDLRGSVSLSWVGDSTGVILVLTTFHVPLVIMTFGQSKLYRSEDYGKNFKDITDLINNTFIRTEFGMAIGPENSGKVVLTAEVSGGSRGGRIFRSSDFAKNFVQTDLPFHPLTQMMYSPQNSDYLLALSTENGLWVSKNFGGKWEEIHKAVCLAKWGSDNTIFFTTYANGSCKADLGALELWRTSDLGKSFKTIGVKIYSFGLGGRFLFASVMADKDTTRRIHVSTDQGDTWSMAQLPSVGQEQFYSILAANDDMVFMHVDEPGDTGFGTIFTSDDRGIVYSKSLDRHLYTTTGGETDFTNVTSLRGVYITSVLSEDNSIQTMITFDQGGRWTHLRKPENSECDATAKNKNECSLHIHASYSISQKLNVPMAPLSEPNAVGIVIAHGSVGDAISVMVPDVYISDDGGYSWTKMLEGPHYYTILDSGGIIVAIEHSSRPINVIKFSTDEGQCWQTYTFTRDPIYFTGLASEPGARSMNISIWGFTESFLTSQWVSYTIDFKDILERNCEEKDYTIWLAHSTDPEDYEDGCILGYKEQFLRLRKSSMCQNGRDYVVTKQPSICLCSLEDFLCDFGYYRPENDSKCVEQPELKGHDLEFCLYGREEHLTTNGYRKIPGDKCQGGVNPVREVKDLKKKCTSNFLSPEKQNSKSNS

1111111111000000000000000000000000000000000000000000000001111100000000000000000000000000000000000000000000000000000000000000000000000000000000000000000000000000000000000000000000000000000000000000000000000000000000000000000000000000000000000000000000000000000000000000000000000000000000000000000000000000000000000000000000000000000000000000000000000000000000000000000000000000000000000000000000000000000000000000000000000000000000000000000000000000000000000000000000000000000000000000000000000000000000000000000000000000000000000000000000000000000000000000000000000000000000000000000000000000000000000000000000000000000000000000000000000000000000000000000000000000000000001111111

>DM_train89

MVKSRKISILLAVAMLVSIMIPTTAFAGPTKAPTKDGTSYKDLFLELYGKIKDPKNGYFSPDEGIPYHSIETLIVEAPDYGHVTTSEAFSYYVWLEAMYGNLTGNWSGVETAWKVMEDWIIPDSTEQPGMSSYNPNSPATYADEYEDPSYYPSELKFDTVRVGSDPVHNDLVSAYGPNMYLMHWLMDVDNWYGFGTGTRATFINTFQRGEQESTWETIPHPSIEEFKYGGPNGFLDLFTKDRSYAKQWRYTNAPDAEGRAIQAVYWANKWAKEQGKGSAVASVVSKAAKMGDFLRNDMFDKYFMKIGAQDKTPATGYDSAHYLMAWYTAWGGGIGASWAWKIGCSHAHFGYQNPFQGWVSATQSDFAPKSSNGKRDWTTSYKRQLEFYQWLQSAEGGIAGGATNSWNGRYEKYPAGTSTFYGMAYVPHPVYADPGSNQWFGFQAWSMQRVMEYYLETGDSSVKNLIKKWVDWVMSEIKLYDDGTFAIPSDLEWSGQPDTWTGTYTGNPNLHVRVTSYGTDLGVAGSLANALATYAAATERWEGKLDTKARDMAAELVNRAWYNFYCSEGKGVVTEEARADYKRFFEQEVYVPAGWSGTMPNGDKIQPGIKFIDIRTKYRQDPYYDIVYQAYLRGEAPVLNYHRFWHEVDLAVAMGVLATYFPDMTYKVPGTPSTKLYG

111111111111111111111111111000000000000000000000000000000000000000000000000000000000000000000000000000000000000000000000000000000000000000000000000000000000000000000000000000000000000000000000000000000000000000000000000000000000000000000000000000000000000000000000000000000000000000000000000000000000000000000000000000000000000000000000000000000000000000000000000000000000000000000000000000000000000000000000000000000000000000000000000000000000000000000000000000000000000000000000000000000000000000000000000000000000000000000000000000000000000000000000000000000000000000000000000000000000000000000000000000000000000000000000000000000000000000000000000000000000000000000111111111

>DM_train90

SPCSVVNDLRWDLSAQQIEERTRELIEQTKRVYDQVGTQEFEDVSYESTLKALADVEVTYTVQRNILDFPQHVSPSKDIRTASTEADKKLSEFDVEMSMREDVYQRIVWLQEKVQKDSLRPEAARYLERLIKLGRRNGLHLPRETQENIKRIKKKLSLLCIDFNKNLNEDTTFLPFTLQELGGLPEDFLNSLEKMEDGKLKVTLKYPHYFPLLKKCHVPETRRKVEEAFNSRCKEENSAILKELVTLRAQKSRLLGFHTHADYVLEMNMAKTSQTVATFLDELAQKLKPLGEQERAVILELKRAECERRGLPFDGRIRAWDMRYYMNQVEETRYCVDQNLLKEYFPVQVVTHGLLGIYQELLGLAFHHEEGASAWHEDVRLYTARDAASGEVVGKFYLDLYPREGKYGHAACFGLQPGCLRQDGSRQIAIAAMVANFTKPTADAPSLLQHDEVRTYFHEFGHVMHQLCSQAEFAMFSGTHVETDFVEAPSQMLENWVWEQEPLLRMSRHYRTGSAVPRELLEKLIESRQANTGLFNLRQIVLAKVDQALHTQTDADPAEEYARLCQEILGVPATPGTNMPATFGHLAGGYDAQYYGYLWSEVYSMDMFHTRFKQEGVLNSKVGMDYRSCILRPGGSEDASAMLRRFLGRDPKQDAFLLSKGLQVGGCEPEPQVC

11111111000000000000000000000000000000000000000000000000000000000000000000000000000000000000000000000000000000000000000000000000000000000000000000000000000000000000000000000000000000000000000000000000000000000000000000000000000000000000000000000000000000000000000000000000000000000000000000000000000000000000000000000000000000000000000000000000000000000000000000000000000000000000000000000000000000000000000000000000000000000000000000000000000000000000000000000000000000000000000000000000000000000000000000000000000000000000000000000000000000000000000000000000000000000000000000000000000000000000000000000000000000000000000000000000000000000000000000000000000000111111111111

>DM_train91

AGVDEAAIRATEQAGGEWLSHGRTYAEQRFSPLKQIDASNVRSLGLAWYMDLDNTRGLEATPLFHDGVIYTSMSWSRVIAVDAASGKELWRYDPEVAKVKARTSCCDAVNRGVALWGDKVYVGTLDGRLIALDAKTGKAIWSQQTTDPAKPYSITGAPRVVKGKVIIGNGGAEYGVRGFVSAYDADTGKLAWRFYTVPGDPALPYEHPELREAAKTWQGDQYWKLGGGGTVWDSMAYDPELDLLYVGTGNGSPWNREVRSPGGGDNLYLSSILAIRPDTGKLAWHYQVTPGDSWDFTATQQITLAELNIDGKPRKVLMQAPKNGFFYVLDRTNGKLISAEKFGKVTWAEKVDLATGRPVEAPGVRYEKEPIVMWPSPFGAHNWHSMSFNPGTGLVYIPYQEVPGVYRNEGKDFVTRKAFNTAAGFADATDVPAAVVSGALLAWDPVKQKAAWKVPYPTHWNGGTLSTAGNLVFQGTAAGQMHAYSADKGEALWQFEAQSGIVAAPMTFELAGRQYVAIMAGWGGVATLTGGESMNLPGMKNRSRLLVFALDGKAQLPPPAPAPAKVERVPQPVTAAPEQVQAGKQLYGQFCSVCHGMGTISGGLIPDLRQSSDATREHFQQIVLQGALKPLGMPSFDDSLKPEEVEQIKLYVMSREYEDYMARHKAAP

00000000000000000000000000000000000000000000000000000000000000000000000000000000000000000000000000000000000000000000000000000000000000000000000000000000000000000000000000000000000000000000000000000000000000000000000000000000000000000000000000000000000000000000000000000000000000000000000000000000000000000000000000000000000000000000000000000000000000000000000000000000000000000000000000000000000000000000000000000000000000000000000000000000000000000000000000000000000000000000000000000000000000000000000000000000000000000000000000000000000000000000000000000000000000000000000000000000000000000000000000000000000000000000000000000000000000000000000000000000000000001111

>DM_train96

MSLSIPREFSNAIRFLSIDATLKAKSGHPGMPMGMADIATVLWTKFLKHNPNNPHWINRDRFVLSNGHGSMLLYSLLHLTGYDLSIEDIKNFRQLHSKTPGHPEYGYTPGVETTTGPLGQGVANAVGMALGEKLLSDRYNTPDLKVIDHHTYVFLGDGCLMEGVSHEACSLAGTLGLNKLVAFWDDNNISIDGDTKGWFSDNTPERFRAYGWHVIENVDGHDFVAIEKAINEAHSQQQKPTLICCKTVIGFGSPEKAGTASVHGSPLSDQERASAAKELNWDYQAFEIPQDVYKYWDAREKGQALEANWQGQRNLFKDSPKFDEFERVLSKELPVGLESAINDYIASQLSNPVKVATRKASQMVLEVLCKNMPEMFGGSADLTGSNNTNWSGSVWLNNTQEGANYLSYGVREFGMAAIMNGLSLYGGIKPYGGTFLVFSDYSRNAIRMSALMKQPVVHVMSHDSIGLGEDGPTHQPIEHVPSLRLIPNLSVWRPADTIETMIAWKEAVKSKDTPSVMVLTRQNLMPVVQTQHQVANIARGGYLVKDNPDAKLTIVATGSEVELAVKVANEFEKKGIKLNVASIPCVEVFATQAHEYKKTVIKDDIPAVFVEMAQPDMWYKYMPKAGGEVKGIYSFGESAPAEDLFKRFGFTVENISNIVAKYV

111000000000000000000000000000000000000000000000000000000000000000000000000000000000000000000000000000000000000000000000000000000000000000000000000000000000000000000000000000000000000000001111110000000000000000000000000000000000000000000000000000000000000000111110000000000000000000000000000000000000000000000000000000000000000000000000000000000000000000000000000000000000000000000001000000000000000000000000000000000000000000000000000000000000000000000000000000000000000000000000000000000000000000000000000000000000000000000000000000000000000000000000000000000000000000000000000000000000000000000000000000000000000000000000000000000000000000000000000000000000000

>DM_train97

MERINFIFGIHNHQPLGNFGWVFEEAYNRSYRPFMEILEEFPEMKVNVHFSGPLLEWIEENKPDYLDLLRSLIKRGQLEIVVAGFYEPVLAAIPKEDRLVQIEMLKDYARKLGYDAKGVWLTERVWQPELVKSLREAGIEYVVVDDYHFMSAGLSKEELFWPYYTEDGGEVITVFPIDEKLRYLIPFRPVKKTIEYLESLTSDDPSKVAVFHDDGEKFGVWPGTYEWVYEKGWLREFFDAITSNEKINLMTYSEYLSKFTPRGLVYLPIASYFEMSEWSLPAKQAKLFVEFVEQLKEEGKFEKYRVFVRGGIWKNFFFKYPESNFMHKRMLMVSKAVRDNPEARKYILKAQCNDAYWHGVFGGIYLPHLRRTVWENIIKAQRYLKPENKILDVDFDGRAEIMVENDGFIATIKPHYGGSIFELSSKRKAVNYNDVLPRRWEHYHEVPEATKPEKESEEGIASIHELGKQIPEEIRRELAYDWQLRAILQDHFIKPEETLDNYRLVKYHELGDFVNQPYEYEMIENGVKLWREGGVYAEEKIPARVEKKIELTEDGFIAKYRVLLEKPYKALFGVEINLAVHSVMEKPEEFEAKEFEVNDPYGIGKVRIELDKAAKVWKFPIKTLSQSEAGWDFIQQGVSYTMLFPIEKELEFTVRFREL

00000000000000000000000000000000000000000000000000000000000000000000000000000000000000000000000000000000000000000000000000000000000000000000000000000000000000000000000000000000000000000000000000000000000000000000000000000000000000000000000000000000000000000000000000000000000000000000000000000000000000000000000000000000000000000000000000000000000000000000000000000000000000000000000000000000000000000000000000000000000000000000000000000000000001111111111111111111111100000000000000000000000000000000000000000000000000000000000000000000000000000000000000000000000000000000000000000000000000000000000000000000000000000000000000000000000000000000000000000000000

>DM_train98

MSRLLALLALAPLLAGAAETTAPKPPSAFTVEAQRRVEAELPFADRADFERADRGLIRRPERLLIRNPDGSVAWQLGGYDFLLDGKPRDSINPSLQRQALLNLKYGLFEVAEGIYQVRGFDLANITFIRGDSGWIVVDTLTTPATARAAYELVSRELGERPIRTVIYSHAHADHFGGVRGLVEPQQVASGAVQIIAPAGFMEAAIKENVLAGNAMMRRATYQYGTQLPKGPQGQVDMAIGKGLARGPLSLLAPTRLIEGEGEDLVLDGVPFTFQNTPGTESPAEMNIWLPRQKALLMAENVVGTLHNLYTLRGAEVRDALGWSKYINQALHRFGRQAEVMFAVHNWPRWGNAEIVEVLEKQRDLYGYLHDQTLHLANQGVTIGQVHNRLRLPPSLDQEWYDRGYHGSVSHNARAVLNRYLGYYDGNPATLDPLSPEDSAGRYVEYMGGAERLLEQARASYARGEYRWVVEVVNRLVFAEPDNRAARELQADALEQLGYQAENAGWRNSYLSAAYELRHGVPRDQPTMKAGSADALAAMDTGLLFDYLGVRLDAGAAEGKALSINLRLPDIGENYLLELKNSHLNNLRGVQSEDAGQTVSIDRADLNRLLLKEVSAVRLVFEGKLKSSGNPLLLGQLFGMLGDFDFWFDIVTPAAKSEG

1111111111111111111000000000000000000000000000000000000000000000000000000000000000000000000000000000000000000000000000000000000000000000000000000000000000000000000000000000000000000000000000000000000000000111000000000000000000000000000000000000000000000000000000000000000000000000000000000000000000000000000000000000000000000000000000000000000000000000000000000000000000000000000000000000000000000000000000000000000000000000000000000000000000000000000000000000000000000000000000000000000000000000000000000000000000000000000011111000000000000000000000000000000000000000000000000000000000000000000000000000000000000000000000000000000000000000000000000000000111

>DM_train100

MNFLRGVMGGQSAGPQHTEAETIQKLCDRVASSTLLDDRRNAVRALKSLSKKYRLEVGIQAMEHLIHVLQTDRSDSEIIGYALDTLYNIISNDEEEEVEENSTRQSEDLGSQFTEIFIKQQENVTLLLSLLEEFDFHVRWPGVKLLTSLLKQLGPQVQQIILVSPMGVSRLMDLLADSREVIRNDGVLLLQALTRSNGAIQKIVAFENAFERLLDIITEEGNSDGGIVVEDCLILLQNLLKNNNSNQNFFKEGSYIQRMKPWFEVGDENSGWSAQKVTNLHLMLQLVRVLVSPNNPPGATSSCQKAMFQCGLLQQLCTILMATGVPADILTETINTVSEVIRGCQVNQDYFASVNAPSNPPRPAIVVLLMSMVNERQPFVLRCAVLYCFQCFLYKNQKGQGEIVSTLLPSTIDATGNTVSAGQLLCGGLFSTDSLSNWCAAVALAHALQENATQKEQLLRVQLATSIGNPPVSLLQQCTNILSQGSKIQTRVGLLMLLCTWLSNCPIAVTHFLHNSANVPFLTGQIAENLGEEEQLVQGLCALLLGISIYFNDNSLETYMKEKLKQLIEKRIGKENFIEKLGFISKHELYSRASQKPQPNFPSPEYMIFDHEFTKLVKELEGVITKAIYKSSEEDKKEEEVKKTLEQHDSI

111111111111111110000000000000000000000000000000000000000000000000000000000000000000000000000111111111111110000000000000000000000000000000000000000000000000000000000000000000000000000000000000000000000000000000000000000000000000000000000000000000000000000000000000000000000000000000000000000000000000000000000000000000000000000000000000000000000000000000000000000000000000000000000000000000000000000000000000000000000000000000000000000000000000000000000000000000000000000000000000000000000000000000000000000000000000000000000000000000000000000000000000000000000000000000000000000000000000000000000000000000000000000000000000000000001111111111111111111

>DM_train101

MGGSHHHHHHGMASLAPGSSRVELFKRQSSKVPFEKDGKVTERVVHSFRLPALVNVDGVMVAIADARYETSFDNSLIDTVAKYSVDDGETWETQIAIKNSRASSVSRVVDPTVIVKGNKLYVLVGSYNSSRSYWTSHGDARDWDILLAVGEVTKSTAGGKITASIKWGSPVSLKEFFPAEMEGMHTNQFLGGAGVAIVASNGNLVYPVQVTNKKKQVFSKIFYSEDEGKTWKFGKGRSAFGCSEPVALEWEGKLIINTRVDYRRRLVYESSDMGNTWLEAVGTLSRVWGPSPKSNQPGSQSSFTAVTIEGMRVMLFTHPLNFKGRWLRDRLNLWLTDNQRIYNVGQVSIGDENSAYSSVLYKDDKLYCLHEINSNEVYSLVFARLVGELRIIKSVLQSWKNWDSHLSSICTPADPAASSSERGCGPAVTTVGLVGFLSHSATKTEWEDAYRCVNASTANAERVPNGLKFAGVGGGALWPVSQQGQNQRYHFANHAFTLVASVTIHEVPKGASPLLGASLDSSGGKKLLGLSYDKRHQWQPIYGSTPVTPTGSWEMGKRYHVVLTMANKIGSVYIDGEPLEGSGQTVVPDERTPDISHFYVGGYKRSGMPTDSRVTVNNVLLYNRQLNAEEIRTLFLSQDLIGTEAHMD

111111111111111110000000000000000000000000000000000000000000000000000000000000000000000000000000000000000000000000000000000000000000000000000000000000000000000000000000000000000000000000000000000000000000000000000000000000000000000000000000000000000000000000000000000000000000000000000000000000000000000000000000000000000000000000000000000000000000000000000000000000000000000000000000000000000000000000000000000001111111110000000000000000000000000000000000000000000000000000000000000000000000000000000000000000000000000000000000000000000000000000000000000000000000000000000000000000000000000000000000000000000000000000000000000000000000000000000000

>DM_train104

MTHHDCAHCSSDACATEMLNLAEANSIETAWHRYEKQQPQCGFGSAGLCCRICLKGPCRIDPFGEGPKYGVCGADRDTIVARHLVRMIAAGTAAHSEHGRHIALAMQHISQGELHDYSIRDEAKLYAIAKTLGVATEGRGLLAIVGDLAAITLGDFQNQDYDKPCAWLAASLTPRRVKRLGDLGLLPHNIDASVAQTMSRTHVGCDADPTNLILGGLRVAMADLDGSMLATELSDALFGTPQPVVSAANLGVMKRGAVNIAVNGHNPMLSDIICDVAADLRDEAIAAGAAEGINIIGICCTGHEVMMRHGVPLATNYLSQELPILTGALEAMVVDVQCIMPSLPRIAECFHTQIITTDKHNKISGATHVPFDEHKAVETAKTIIRMAIAAFGRRDPNRVAIPAFKQKSIVGFSAEAVVAALAKVNADDPLKPLVDNVVNGNIQGIVLFVGCNTTKVQQDSAYVDLAKSLAKRNVLVLATGCAAGAFAKAGLMTSEATTQYAGEGLKGVLSAIGTAAGLGGPLPLVMHMGSCVDNSRAVALATALANKLGVDLSDLPLVASAPECMSEKALAIGSWAVTIGLPTHVGSVPPVIGSQIVTKLVTETAKDLVGGYFIVDTDPKSAGDKLYAAIQERRAGLGL

111111111111111111111111111000000000000000000000000000000000000000000000000000000000000000000000000000000000000000000000000000000000000000000000000000000000000000000000000000000000000000000000000000000000000000000000000000000000000000000000000000000000000000000000000000000000000000000000000000000000000000000000000000000000000000000000000000000000000000000000000000000000000000000000000000000000000000000000000000000000000000000000000000000000000000000000000000000000000000000000000000000000000000000000000000000000000000000000000000000000000000000000000000000000000000000000000000000000000000000000000000000000000000000000000000000000000

>DM_train105

PILEKAPQKMPVKASSWEELDLPKLPVPPLQQTLATYLQCMQHLVPEEQFRKSQAIVKRFGAPGGLGETLQEKLLERQEKTANWVSEYWLNDMYLNNRLALPVNSSPAVIFARQHFQDTNDQLRFAACLISGVLSYKTLLDSHSLPTDWAKGQLSGQPLCMKQYYRLFSSYRLPGHTQDTLVAQKSSIMPEPEHVIVACCNQFFVLDVVINFRRLSEGDLFTQLRKIVKMASNEDERLPPIGLLTSDGRSEWAKARTVLLKDSTNRDSLDMIERCICLVCLDGPGTGELSDTHRALQLLHGGGCSLNGANRWYDKSLQFVVGRDGTCGVVCEHSPFDGIVLVQCTEHLLKHMMTSNKKLVRADSVSELPAPRRLRWKCSPETQGHLASSAEKLQRIVKNLDFIVYKFDNYGKTFIKKQKYSPDGFIQVALQLAYYRLYQRLVPTYESASIRRFQEGRVDNIRSATPEALAFVQAMTDHKAAMPASEKLQLLQTAMQAQTEYTVMAITGMAIDNHLLALRELARDLCKEPPEMFMDETYLMSNRFVLSTSQVPTTMEMFCCYGPVVPNGYGACYNPQPEAITFCISSFHSCKETSSVEFAEAVGASLVDMRDLCSSRQPADSKPPAPKEKARGPSQAKQS

111111111111111111000000000000000000000000000000000000000000000000000000000000000000000000000000000000000000000000000000000000000000000000000000000000000000000000000000000000000000000000000000000000000000000000000000000000000000000000000000000000000000000000000000000000000000000000000000000000000000000000000000000000000000000000000000000000000000000000000000000000000000000000000000000000000000000000000000000000000000000000000000000000000000000000000000000000000000000000000000000000000000000000000000000000000000000000000000000000000000000000000000000000000000000000000000000000000000000000000000000000000000000111111111111111111111111

>DM_train106

APAADAAQAHDPLSVQTGSDIPASVHMPTDQQRDYIKREVMVPMRDGVKLYTVIVIPKNARNAPILLTRTPYNAKGRANRVPNALTMREVLPQGDDVFVEGGYIRVFQDIRGKYGSQGDYVMTRPPHGPLNPTKTDETTDAWDTVDWLVHNVPESNGRVGMTGSSYEGFTVVMALLDPHPALKVAAPESPMVDGWMGDDWFHYGAFRQGAFDYFVSQMTARGGGNDIPRRDADDYTNFLKAGSAGSFATQAGLDQYPFWQRMHAHPAYDAFWQGQALDKILAQRKPTVPMLWEQGLWDQEDMWGAIHAWQALKDADVKAPNTLVMGPWRHSGVNYNGSTLGPLEFEGDTAHQYRRDVFRPFFDEYLKPGSASVHLPDAIIYNTGDQKWDYYRSWPSVCESNCTGGLTPLYLADGHGLSFTHPAADGADSYVSDPAHPVPFISRPFAFAQSSRWKPWLVQDQREAESRPDVVTYETEVLDEPVRVSGVPVADLFAATSGTDSDWVVKLIDVQPAMTPDDPKMGGYELPVSMDIFRGRYRKDFAKPEALQPDATLHYHFTLPAVNHVFAKGHRIMVQIQSSWFPLYDRNPQKFVPNIFDAKPADYTVATQSIHHGGKEATSILLPVVKQEQKLISEEDL

1111111110000000000000111111111000000000000000000000000000000000000000000000000000000000000000000000000000000000000000000000000000000000000000000000000000000000000000000000000000000000000000000000000000000000000000000000000000000000000000000000000000000000000000000000000000000000000000000000000000000000000000000000000000000000000000000000000000000000000000000000000000000000000000000000000000000000000000000000000000000000000000000000000000000000000000000000000000000000000000000000000000000000000000000000000000000000000000000000000000000000000000000000000000000000000000000000000000000000000000000000000000000000000000000011111111111

>DM_train107

GSHMLDNMDVIGERIKRIKEEHNSTWHYDDENPYKTWAYHGSYEVKATGSASSMINGVVKLLTKPWDVVPMVTQMAMTDTTPFGQQRVFKEKVDTRTPRPLPGTRKVMEITAEWLWRTLGRNKRPRLCTREEFTKKVRTNAAMGAVFTEENQWDSAKAAVEDEEFWKLVDRERELHKLGKCGSCVYNMMGKREKKLGEFGKAKGSRAIWYMWLGARYLEFEALGFLNEDHWFSRENSYSGVEGEGLHKLGYILRDISKIPGGAMYADDTAGWDTRITEDDLHNEEKIIQQMDPEHRQLANAIFKLTYQNKVVKVQRPTPTGTVMDIISRKDQRGSGQVGTYGLNTFTNMEAQLVRQMEGEGVLTKADLENPHLLEKKITQWLETKGVERLKRMAISGDDCVVKPIDDRFANALLALNDMGKVRKDIPQWQPSKGWHDWQQVPFCSHHFHELIMKDGRKLVVPCRPQDELIGRARISQGAGWSLRETACLGKAYAQMWSLMYFHRRDLRLASNAICSAVPVHWVPTSRTTWSIHAHHQWMTTEDMLTVWNRVWIEENPWMEDKTPVTTWENVPYLGKREDQWCGSLIGLTSRATWAQNIPTAIQQVRSLIGNEEFLDYMPSMKRFRKEEESEGAIW

11111110000000000000000000000000000000000000011111100000000000000000000000000000000000000000000000000000000000000000000000000000000000000000111111111111100000000000000000000000000000000111111111111111111100000000000000000000000000000000000000000000000000000000000000000000000000000000000000000000000000000000000000000000000000000000000000000000000000000000000000000000000000000000000000000000000000000000000000000000000000000000000000000000000000000000000000000000000000000000000000000000000000000000000000000000000000000000000000000000000000000000000000000000000000000000000000000000000000000000000000000000000000000011111111111111111

>DM_train108

MAEQLVEAPAYARTLDRAVEYLLSCQKDEGYWWGPLLSNVTMEAEYVLLCHILDRVDRDRMEKIRRYLLHEQREDGTWALYPGGPPDLDTTIEAYVALKYIGMSRDEEPMQKALRFIQSQGGIESSRVFTRMWLALVGEYPWEKVPMVPPEIMFLGKRMPLNIYEFGSWARATVVALSIVMSRQPVFPLPERARVPELYETDVPPRRRGAKGGGGWIFDALDRALHGYQKLSVHPFRRAAEIRALDWLLERQAGDGSWGGIQPPWFYALIALKILDMTQHPAFIKGWEGLELYGVELDYGGWMFQASISPVWDTGLAVLALRAAGLPADHDRLVKAGEWLLDRQITVPGDWAVKRPNLKPGGFAFQFDNVYYPDVCDTAVVVWALNTLRLPDERRRRDAMTKGFRWIVGMQSSNGGWGAYDVDNTSDLPNHIPFCDFGEVTDPPSEDVTAHVLECFGSFGYDDAWKVIRRAVEYLKREQKPDGSWFGRWGVNYLYGTGAVVSALKAVGIDTREPYIQKALDWVEQHQNPDGGWGEDCRSYEDPAYAGKGASTPSQTAWALMALIAGGRAESEAARRGVQYLVETQRPDGGWDEPYYTGTGFPGDFYLGYTMYRHVFPTLALGRYKQAIERR

1111111110000000000000000000000000000000000000000000000000000000000000000000000000000000000000000000000000000000000000000000000000000000000000000000000000000000000000000000000000000000000000000000000000000000000000000000000000000000000000000000000000000000000000000000000000000000000000000000000000000000000000000000000000000000000000000000000000000000000000000000000000000000000000000000000000000000000000000000000000000000000000000000000000000000000000000000000000000000000000000000000000000000000000000000000000000000000000000000000000000000000000000000000000000000000000000000000000000000000000000000000000000000000000000000111

>DM_train110

GELMPPQLQNGLNLSAKVVQGSLDSLPQAVREFLENNAELCQPDHIHICDGSEEENGRLLGQMEEEGILRRLKKYDNCWLALTDPRDVARIESKTVIVTQEQRDTVPIPKTGLSQLGRWMSEEDFEKAFNARFPGCMKGRTMYVIPFSMGPLGSPLSKIGIELTDSPYVVASMRIMTRMGTPVLEALGDGEFVKCLHSVGCPLPLQKPLVNNWPCNPELTLIAHLPDRREIISFGSGYGGNSLLGKKCFALRMASRLAKEEGWLAEHMLVLGITNPEGEKKYLAAAFPSACGKTNLAMMNPSLPGWKVECVGDDIAWMKFDAQGHLRAINPENGFFGVAPGTSVKTNPNAIKTIQKNTIFTNVAETSDGGVYWEGIDEPLASGVTITSWKNKEWSSEDGEPCAHPNSRFCTPASQCPIIDAAWESPEGVPIEGIIFGGRRPAGVPLVYEALSWQHGVFVGAAMRSEATAAAEHKGKIIMHDPFAMRPFFGYNFGKYLAHWLSMAQHPAAKLPKIFHVNWFRKDKEGKFLWPGFGENSRVLEWMFNRIDGKASTKLTPIGYIPKEDALNLKGLGHINMMELFSISKEFWDKEVEDIEKYLVDQVNADLPCEIEREILALKQRISQM

1111111111110000000000000000000000000000000000000000000000000000000000000000000000000000000000000000000000000000000000000000000000000000000000000000000000000000000000000000000000000000000000000000000000000000000000000000000000000000000000000000000000000000000000000000000000000000000000000000000000000000000000000000000000000000000000000000000000000000000000000000000000000000000000000000000000000000000000000000000000000000000000000000000000000000000000000000000000011111111000000000000000000000000000000000000000000000000000000000000000000000000000000000000000000000000000000000000000000000000000000000000000000000000000000

>DM_train112

STIRIGGAAVNQTPIDWENNVKNILDAIEEAKNANVEILCLPELCITGYGCEDLFLTDWVAETAIEYCFEIAASCTDITVSLGLPMRIAGITYNCVCLVENGIVKGFSAKQFLANEGVHYETRWFTAWPRNHTTTFLYNDVKYPFGDVLYNVKDARIGFEICEDAWRTDRVGIRHYEKGATLVLNPSASHFAFGKSAIRYDLVIGGSERFDCTYVYANLLGNEAGRMIYDGEVLIAHKGKLIQRNDRLSFKNVNLIYADIATDSAETPETVLTQDDLEKEFEFWEATSLGLFDYMRKSRSKGFVLSLSGGADSSACAIMVAEMIRKGLKELGLTAFLQKSNMETLFDLPALQHLPFEEQAKKITAVFLTTAYQSTRNSGDETYTSAKTLAESIGATFYNWSVDEEIEQYKATIENVIERPLTWEKDDITLQNIQARGRAPIIWMLTNVKQALLITTSNRSEGDVGYATMDGDTAGGIAPIAGVDKDFIRSWLRWAEKNRNQHGLHIVNKLAPTAELRPSEYTQTDERDLMPYDVLARIERKAIKERLSPVQVYTALLTEGPYTKNEFKYWVKKFFRLWSINQWKRERLAPSFHMDDFNIDPRSWYRFPILSSGFAKELNDLDQ

00000000000000000000000000000000000000000000000000000000000000000000000000000000000000000000000000000000000000000000000000000000000000000000000000000000000000000000111111111100000000000000000000000000000000000000000000000000000000000000000000000000000000000000001111111111111100000000000000000000000000000000000000000000000000000000000000000000000000000000000000000000000000000000000000000000000000000000000000000000000000000000000000000000000000000000000000000000000000000000000000000000000000000000000000001111111111111111111000000000000000000000000000000000000000000000000000000000000000000000000000000000000000000000000

>DM_train115

MYGWWGRILRVNLTTGEVKVQEYPEEVAKKFIGGRGLAAWILWNEARGVEPLSPENKLIFAAGPFNGLPTPSGGKLVVAAKSPLTGGYGDGNLGTMASVHLRRAGYDALVVEGKAKKPVYIYIEDDNVSILSAEGLWGKTTFETERELKEIHGKNVGVLTIGPAGENLVKYAVVISQEGRAAGRPGMGAVMGSKKLKAVVIRGTKEIPVADKEELKKLSQEAYNEILNSPGYPFWKRQGTMAAVEWCNTNYALPTRNFSDGYFEFARSIDGYTMEGMKVQQRGCPYCNMPCGNVVLDAEGQESELDYENVALLGSNLGIGKLNEVSVLNRIADEMGMDTISLGVSIAHVMEAVERGILKEGPTFGDFKGAKQLALDIAYRKGELGNLAAEGVKAMAEKLGTHDFAMHVKGLEVSGYNCYIYPAMALAYGTSAIGAHHKEAWVIAWEIGTAPIEGEKAEKVEYKISYDPIKAQKVVELQRLRGGLFEMLTACRLPWVEVGLSLDYYPKLLKAITGVTYTWDDLYKAADRVYSLIRAYWVREFNGKWDRKMDYPPKRWFTEGLKSGPHKGEHLDEKKYDELLSEYYRIRGWDERGIPKKETLKELDLDFVIPELEKVTNLE

0000000000000000000000000000000000000000000000000000000000000000000000000000000000000000000000000000000000000000000000000000000000000000000000000000000000000000000000000000000000000000000000000000000000000000000000000000000000000000000000000000000000000000000000000000000000000000000000000000000000000000000000000000000000000000000000000000000000000000000000000000000000000000000000000000000000000000000000000000000000000000000000000000000000000000000001111111100000000000000000000000000000000000000000000000000000000000000000000000000000000000000000000000000000000000000000000000000000000000000000000000000000000000000

>DM_train116

MKPKKRQMEYLTRGLIAVQTEQGVFVSWRFLGTDHETTAFHLYRDGKRITRDPIAESTNFLDQNGTADSVYQVAAVNKGREEKLSKKARVWQENVLEVPLAKPEGGVTPDGKPYTYSANDASVGDIDGDGEYEMILKWDPSNSKDNAHDGYTGEVLIDAYKLDGTFLWRINLGRNIRAGAHYTQFMVYDLDGDGKAEIAMKTADGTTDGKGHIIGDEQADFRNEQGRILSGPEYLTVFKGETGEALTTVEYEPPRGKLEDWGDGYGNRMDRFLAGTAYLDGERPSLVMARGYYTRTVLVAYDFRNGRLKKRWVFDSNQPGHEAYAGQGNHSLSVADVDGDGKDEIIYGAMAVDHDGTGLYSTGLGHGDAMHVGDLDPSRKGLEVFQVHEDATKPYGLSLRDAGTGEILWGVHAGTDVGRGMAAHIDPSYKGSLVWGIDPPGNDGMSYGLFTSKGEKISDKAPSSANFAIWWDGDLVRELLDHDWDGTIGRPKIEKWDAENGCLKTIFQPAGVLSNNGTKGNPVLQANLFGDWREEVIWRTEDSSALRIYTTTHLTRHCFYTLMHDPVYRLGIAWQNTAYNQPPHTSFYLGTGMKKPPKPALYIAGSKAEAPL

110000000000000000000000000000000000000000000000000000000000000000000000000000000000000000000000000000000000000000000000000000000000000000000000000000000000000000000000000000000000000000000000000000000000000000000000000000000000000000000000000000000000000000000000000000000000000000000000000000000000000000000000000000000000000000000000000000000000000000000000000000000000000000000000000000000000000000000000000000000000000000000000000000000000000000000000000000000000000000000000000000000000000000000000000000000000000000000000000000000000000000000000000000000000000000000000000000000000000000000000000000111111

>DM_train117

MRYIAGIDIGNSSTEVALATLDEAGALTITHSALAETTGIKGTLRNVFGIQEALALVARGAGIAVSDISLIRINEATPVIGDVAMETITETIITESTMIGHNPKTPGGAGLGTGITITPQELLTRPADAPYILVVSSAFDFADIASVINASLRAGYQITGVILQRDDGVLVSNRLEKPLPIVDEVLYIDRIPLGMLAAIEVAVPGKVIETLSNPYGIATVFNLSPEETKNIVPMARALIGNRSAVVVKTPSGDVKARAIPAGNLELLAQGRSVRVDVAAGAEAIMKAVDGCGRLDNVTGESGTNIGGMLEHVRQTMAELTNKPSSEIFIQDLLAVDTSVPVSVTGGLAGEFSLEQAVGIASMVKSDRLQMAMIAREIEQKLNIDVQIGGAEAEAAILGALTTPGTTRPLAILDLGAGSTDASIINPKGDIIATHLAGAGDMVTMIIARELGLEDRYLAEEIKKYPLAKVESLFHLRHEDGSVQFFSTPLPPAVFARVCVVKADELVPLPGDLALEKVRAIRRSAKERVFVTNALRALRQVSPTGNIRDIPFVVLVGGSSLDFEVPQLVTDALAHYRLVAGRGNIRGSEGPRNAVATGLILSWHKEFAHER

0000000000000000000000000000000000000000000000000000000000000000000000000000000000000000000000000000000000000000000000000000000000000000000000000000000000000000000000000000000000000000000000000000000000000000000000000000000000000000000000000000000000000000000000000000000000000000000000000000000000000000000000000000000000000000000000000000000000000000000000000000000000000000000000000000000000000000000000000000000000000000000000000000000000000000000000000000000000000000000000000000000000000000000000000000000000000000000000000000000000000000000000000000000000000000000000000000000000000000000000000000111111

>DM_train120

MAKKVNWYVSCSPRSPEKIQPELKVLANFEGSYWKGVKGYKAQEAFAKELAALPQFLGTTYKKEAAFSTRDRVAPMKTYGFVFVDEEGYLRITEAGKMLANNRRPKDVFLKQLVKWQYPSFQHKGKEYPEEEWSINPLVFVLSLLKKVGGLSKLDIAMFCLTATNNNQVDEIAEEIMQFRNEREKIKGQNKKLEFTENYFFKRFEKIYGNVGKIREGKSDSSHKSKIETKMRNARDVADATTRYFRYTGLFVARGNQLVLNPEKSDLIDEIISSSKVVKNYTRVEEFHEYYGNPSLPQFSFETKEQLLDLAHRIRDENTRLAEQLVEHFPNVKVEIQVLEDIYNSLNKKVDVETLKDVIYHAKELQLELKKKKLQADFNDPRQLEEVIDLLEVYHEKKNVIEEKIKARFIANKNTVFEWLTWNGFIILGNALEYKNNFVIDEELQPVTHAAGNQPDMEIIYEDFIVLGEVTTSKGATQFKMESEPVTRHYLNKKKELEKQGVEKELYCLFIAPEINKNTFEEFMKYNIVQNTRIIPLSLKQFNMLLMVQKKLIEKGRRLSSYDIKNLMVSLYRTTIECERKYTQIKAGLEETLNNWVVDKEVRF

0000000000000000000000000000000000000000000000000000000000111111100000000000000000000000000000000000000000000000000000000000000000000000000000000000000000000000000000000000000000000000000000000000000000000000000111111111100000000000000000000000000000000000000000000000000000000000000000000000000000000000000000000000000000000000000000000000000000000000000000000000000000000000000000000000000000000000000000000000000000000000000000000000000000000000000000000000000000000000000000000000000000000000000000000000000000000000000000000000000000000000000000000000000000000000000000000000000000000000000000000000

>DM_train121

MASEIHMTGPMCLIENTNGRLMANPEALKILSAITQPMVVVAIVGLYRTGKSYLMNKLAGKKKGFSLGSTVQSHTKGIWMWCVPHPKKPGHILVLLDTEGLGDVEKGDNQNDSWIFALAVLLSSTFVYNSIGTINQQAMDQLYYVTELTHRIRSKSSPDENENEVEDSADFVSFFPDFVWTLRDFSLDLEADGQPLTPDEYLTYSLKLKKGTSQKDETFNLPRLCIRKFFPKKKCFVFDRPVHRRKLAQLEKLQDEELDPEFVQQVADFCSYIFSNSKTKTLSGGIQVNGPRLESLVLTYVNAISSGDLPCMENAVLALAQIENSAAVQKAIAHYEQQMGQKVQLPTESLQELLDLHRDSEREAIEVFIRSSFKDVDHLFQKELAAQLEKKRDDFCKQNQEASSDRCSGLLQVIFSPLEEEVKAGIYSKPGGYRLFVQKLQDLKKKYYEEPRKGIQAEEILQTYLKSKESMTDAILQTDQTLTEKEKEIEVERVKAESAQASAKMLHEMQRKNEQMMEQKERSYQEHLKQLTEKMENDRVQLLKEQERTLALKLQEQEQLLKEGFQKESRIMKNEIQDLQTKMRRRKACTIS

1111100000000000000000000000000000000000000000000000000000000011111111111000000000000000000000000000000000000000000000000000000000000000000000000000000000001111111111000000000000000000000001111000000000000000000000000000000000000000000000000001111111111111000000000000000000000000000000000000000000000000000000000000000000000000000000000000000000000000000000000000000000000000000000000000000000000000000000000000000000000000000000000000000000000000000000000000000000000000000000000000000000000000000000000000000000000000000000000000000000000000000000000000000000000000000000000000000111111111

>DM_train123

IETGYTPIDISLSLTQFLLSEFVPGAGFVLGLVDIIWGIFGPSQWDAFLVQIEQLINQRIEEFARNQAISRLEGLSNLYQIYAESFREWEADPTNPALREEMRIQFNDMNSALTTAIPLLAVQNYQVPLLSVYVQAANLHLSVLRDVSVFGQRWGFDAATINSRYNDLTRLIGNYTDYAVRWYNTGLERVWGPDSRDWVRYNQFRRELTLTVLDIVALFSNYDSRRYPIRTVSQLTREIYTNPVLENFDGSFRGMAQRIEQNIRQPHLMDILNSITIYTDVHRGFNYWSGHQITASPVGFSGPEFAFPLFGNAGNAAPPVLVSLTGLGIFRTLSSPLYRRIILGSGPNNQELFVLDGTEFSFASLTTNLPSTIYRQRGTVDSLDVIPPQDNSVPPRAGFSHRLSHVTMLSQAAGAVYTLRAPTFSWQHRSAEFNNIIPSSQITQIPLTKSTNLGSGTSVVKGPGFTGGDILRRTSPGQISTLRVNITAPLSQRYRVRIRYASTTNLQFHTSIDGRPINQGNFSATMSSGSNLQSGSFRTVGFTTPFNFSNGSSVFTLSAHVFNSGNEVYIDRIEFVPAEVTFEAEYDLER

11110000000000000000000000000000000000000000000000000000000000000000000000000000000000000000000000000000000000000000000000000000000000000000000000000000000000000000000000000000000000000000000000000000000000000000000000000000000000000000000000000000000000000000000000000000000000000000000000000000000000000000000000000000000000000000000000000000000000000000000000000000000000000000000000000000000000000000000000000000000000000000000000000000000000000000000000000000000000000000000000000000000000000000000000000000000000000000000000000000000000000000000000000000000000000000000000000111111111

>DM_train124

LVTDEAEASKFVEEYDRTSQVVWNEYAEANWNYNTNITTETSKILLQKNMQIANHTLKYGTQARKFDVNQLQNTTIKRIIKKVQDLERAALPAQELEEYNKILLDMETTYSVATVCHPNGSCLQLEPDLTNVMATSRKYEDLLWAWEGWRDKAGRAILQFYPKYVELINQAARLNGYVDAGDSWRSMYETPSLEQDLERLFQELQPLYLNLHAYVRRALHRHYGAQHINLEGPIPAHLLGNMWAQTWSNIYDLVVPFPSAPSMDTTEAMLKQGWTPRRMFKEADDFFTSLGLLPVPPEFWNKSMLEKPTDGREVVCHASAWDFYNGKDFRIKQCTTVNLEDLVVAHHEMGHIQYFMQYKDLPVALREGANPGFHEAIGDVLALSVSTPKHLHSLNLLSSEGGSDEHDINFLMKMALDKIAFIPFSYLVDQWRWRVFDGSITKENYNQEWWSLRLKYQGLCPPVPRTQGDFDPGAKFHIPSSVPYIRYFVSFIIQFQFHEALCQAAGHTGPLHKCDIYQSKEAGQRLATAMKLGFSRPWPEAMQLITGQPNMSASAMLSYFKPLLDWLRTENELHGEKLGWPQYNWTPNS

1110000000000000000000000000000000000000000000000000000000000000000000000000000000000000000000000000000000000000000000000000000000000000000000000000000000000000000000000000000000000000000000000000000000000000000000000000000000000000000000000000000000000000000000000000000000000000000000000000000000000000000000000000000000000000000000000000000000000000000000000000000000000000000000000000000000000011110000000000000000000000000000000000000000000000000000000000000000000000000000000000000000000000000000000000000000000000000000000000000000000000000000000000000000000000000000000000011111111

>DM_train125

DAHKSEVAHRFKDLGEENFKALVLIAFAQYLQQCPFEDHVKLVNEVTEFAKTCVADESAENCDKSLHTLFGDKLCTVATLRETYGEMADCCAKQEPERNECFLQHKDDNPNLPRLVRPEVDVMCTAFHDNEETFLKKYLYEIARRHPYFYAPELLFFAKRYKAAFTECCQAADKAACLLPKLDELRDEGKASSAKQRLKCASLQKFGERAFKAWAVARLSQRFPKAEFAEVSKLVTDLTKVHTECCHGDLLECADDRADLAKYICENQDSISSKLKECCEKPLLEKSHCIAEVENDEMPADLPSLAADFVESKDVCKNYAEAKDVFLGMFLYEYARRHPDYSVVLLLRLAKTYETTLEKCCAAADPHECYAKVFDEFKPLVEEPQNLIKQNCELFEQLGEYKFQNALLVRYTKKVPQVSTPTLVEVSRNLGKVGSKCCKHPEAKRMPCAEDYLSVVLNQLCVLHEKTPVSDRVTKCCTESLVNRRPCFSALEVDETYVPKEFNAETFTFHADICTLSEKERQIKKQTALVELVKHKPKATKEQLKAVMDDFAAFVEKCCKADDKETCFAEEGKKLVAASQAALGL

111100000000000000000000000000000000000000000000000000000000000000000000000011111111111100000000000000000000000000000000000000000000000000000000000000000000000000000000000000000000000000000000000000000000000000000000000000000000000000000000000000000000000000000000000000000000000000000000000000000000000000000000000000000000000000000000000000000000000000000000000000000000000000000000000000000000000000000000000000000000000000000000000000000000000000000000000000000000000000000000000000000000000000000000000000000000000000000000000000000000000000000000000000000000000001111111111111111

>DM_train127

GAMGSPKEHIDLYQQIKWNGWGDTRKFLHQLKPSGTIAMTTPEVSSVPLPSLRGFIKKELTLPGEEDKPFVLDETPALQIENIHVDPPKQYPEFVRELKAFFLPDQLKDDKLARITHTFGKSLRDLIRVRIGQVKNAPDLIVLPHSHEEVERLVQLAHKYNVVIIPMGGGSNIVGAIEPVSNERFTVSIDMRRMNKVLWVDRREMTACIQVGIMGPELEKQLHKQGVSLGHDPDSFEFSTLGGWLATCSSGHQSDKYGDIEDMAVSFRTVTPTGTLELRNGARSGAGINYKHIILGSEGTLGIITEAVMKVHAVPQAVEYYGFLFPTFAHAVSALQQIRSSEVIPTMIRVYDPEETQLSFAWKPSKGAVSEFTSAMVKKYLHYIRSFDFKNVCLSIIGFEGPKKVVDFHRTSVFDILSKNAAFGLGSAPGKTWAEKRYDLPYIRDFLLDHNMWVDVAETTVSYANLQTLWKDAKQTFVKHFKDQGIPAWICAHISHTYTNGVCLYFIFASKQNENKDMAQYIEAKKLMTDIIFKYGGSLSHHHGVGYEHVPWMTRYATRGWINVYRSLKETIDPKDICNPRKLI

00000000000000000000000000000000000000000000000000000000000111111111100000000000000000000000000000000000000000000000000000000000000000000000000000000000000000000000000000000000000000000000000000000000000000000000000000000000000000000000000000000000000000000000000000000000000000011111100000000000000000000000000000000000000000000000000000000000000000000000000000000111100000000000000000000000000000000000000000000000000000000000000000000000000000000000000000000000000000000000000000000000000000000000000000000000011111000000000000000000000111111111111111111110000000000000000000000011

>DM_train128

PVFSADPGAPAPVNPCCYYPCQHQGICVRFGLDRYQCDCTRTGYSGPNCTIPEIWTWLRTTLRPSPSFIHFLLTHGRWLWDFVNATFIRDTLMRLVLTVRSNLIPSPPTYNIAHDYISWESFSNVSYYTRILPSVPRDCPTPMGTKGKKQLPDAEFLSRRFLLRRKFIPDPQGTNLMFAFFAQHFTHQFFKTSGKMGPGFTKALGHGVDLGHIYGDNLERQYQLRLFKDGKLKYQMLNGEVYPPSVEEAPVLMHYPRGIPPQSQMAVGQEVFGLLPGLMLYATIWLREHNRVCDLLKAEHPTWGDEQLFQTARLILIGETIKIVIEEYVQQLSGYFLQLKFDPELLFGAQFQYRNRIAMEFNQLYHWHPLMPDSFRVGPQDYSYEQFLFNTSMLVDYGVEALVDAFSRQPAGRIGGGRNIDHHILHVAVDVIKESRVLRLQPFNEYRKRFGMKPYTSFQELTGEKEMAAELEELYGDIDALEFYPGLLLEKCHPNSIFGESMIEMGAPFSLKGLLGNPICSPEYWKASTFGGEVGFNLVKTATLKKLVCLNTKTCPYVSFHVPDPRQEDRPGVERPPTEL

1111111111110000000000000000000000000000000000000000000000000000000000000000000000000000000000000000000000000000000000000000000000000000000000000000000000000000000000000000000000000000000000000000000000000000000000000000000000000000000000000000000000000000000000000000000000000000000000000000000000000000000000000000000000000000000000000000000000000000000000000000000000000000000000000000000000000000000000000000000000000000000000000000000000000000000000000000000000000000000000000000000000000000000000000000000000000000000000000000000000000000000000000000000000011111111111111111

>DM_train129

MVSKIRTFGWVQNPGKFENLKRVVQVFDRNSKVHNEVKNIKIPTLVKESKIQKELVAIMNQHDLIYTYKELVGTGTSIRSEAPCDAIIQATIADQGNKKGYIDNWSSDGFLRWAHALGFIEYINKSDSFVITDVGLAYSKSADGSAIEKEILIEAISSYPPAIRILTLLEDGQHLTKFDLGKNLGFSGESGFTSLPEGILLDTLANAMPKDKGEIRNNWEGSSDKYARMIGGWLDKLGLVKQGKKEFIIPTLGKPDNKEFISHAFKITGEGLKVLRRAKGSTKFTRVPKRVYWEMLATNLTDKEYVRTRRALILEILIKAGSLKIEQIQDNLKKLGFDEVIETIENDIKGLINTGIFIEIKGRFYQLKDHILQFVIPNRLGKPDLVKSELEEKKSELRHKLKYVPHEYIELIEIARNSTQDRILEMKVMEFFMKVYGYRGKHLGGSRKPDGAIYTVGSPIDYGVIVDTKAYSGGYNLPIGQADEMQRYVEENQTRNKHINPNEWWKVYPSSVTEFKFLFVSGHFKGNYKAQLTRLNHITNCNGAVLSVEELLIGGEMIKAGTLTLEEVRRKFNNGEINF

111100000000000000000000000000000000000000000000000000000000000000000000000011111000000000000000000000000000000000000000000000000000000000000000000000000000000000000000000000000000000000000000000000000000000000000000000000000000000000000000000000000001111100000000000000000000000000000000000000000000000000000000000000000000000000000000000000000000000000000000000000000000000000011111000000000000000000000000000000000000000000000000000000000000000000000000000000000000000000000000000000000000000000000000000000000000000000000000000000000000000000000000000000000000000000000000000

>DM_train130

SMSYTWTGALITPCAAEESKLPINALSNSLLRHHNMVYATTSRSAGLRQKKVTFDRLQVLDDHYRDVLKEMKAKASTVKAKLLSVEEACKLTPPHSAKSKFGYGAKDVRNLSSKAVNHIHSVWKDLLEDTVTPIDTTIMAKNEVFCVQPEKGGRKPARLIVFPDLGVRVCEKMALYDVVSTLPQVVMGSSYGFQYSPGQRVEFLVNTWKSKKNPMGFSYDTRCFDSTVTENDIRVEESIYQCCDLAPEARQAIKSLTERLYIGGPLTNSKGQNCGYRRCRASGVLTTSCGNTLTCYLKASAACRAAKLQDCTMLVNGDDLVVICESAGTQEDAASLRVFTEAMTRYSAPPGDPPQPEYDLELITSCSSNVSVAHDASGKRVYYLTRDPTTPLARAAWETARHTPVNSWLGNIIMYAPTLWARMILMTHFFSILLAQEQLEKALDCQIYGACYSIEPLDLPQIIERLHGLSAFSLHSYSPGEINRVASCLRKLGVPPLRVWRHRARSVRARLLSQGGRAATCGKYLFNWAVKTKLKLTPIPAASQLDLSGWFVAGYSGGDIYHSLSRARPRGSHHHHHH

00000000000000000000000000000000000000000000000000000000000000000000000000000000000000000000000000000000000000000000000000000000000000000000000000000000000000000000000000000000000000000000000000000000000000000000000000000000000000000000000000000000000000000000000000000000000000000000000000000000000000000000000000000000000000000000000000000000000000000000000000000000000000000000000000000000000000000000000000000000000000000000000000000000000000000000000000000000000000000000000000000000000000000000000000000000000000000000000000000000000000011100000000001111111111111111111111

>DM_train131

GSNQSSSTSTKKLKAGNFDVAYQNPDKAIKGGNLKVAYQSDSPMKAQWLSGLSNDATFATMSGPGGGQDGLFFTDSGFKFIKGGAADVALDKESKTATITLRKDLKWSDGSEVTAKDYEFTYETIANPAYGSDRWTDSLANIVGLSDYHTGKAKTISGITFPDGENGKVIKVQFKEMKPGMTQSGNGYFLETVAPYQYLKDVAPKDLASSPKTTTKPLVTGPFKPENVVAGESIKYVPNPYYWGEKPKLNSITYEVVSTAKSVAALSSSKYDIINGMVSSQYKQVKNLKGYKVLGQQAMYISLMYYNLGHYDAKNSINVQDRKTPLQDQNVRQAIGYARNVAEVDNKFSNGLSTPANSLIPPIFKQFTSSSVKGYEKQDLDKANKLLDEDGWKLNKSTGYREKDGKELSLVYAARVGDANAETIAQNYIQQWKKIGVKVSLYNGKLMEFNSWVDHMTTPPGANDWDITDGSWSLASEPSQQDLFSAAAPYNFGHFNDSEITKDLNDIDSAKSENPTYRKAAFVKYQEDMNKKAYVIPTNFMLNYTPVNKRVVGMTLDYGAMNTWSEIGVSSAKLATK

1111111111111111111111111111110000000000000000000000000000000000000000000000000000000000000000000000000000000000000000000000000000000000000000000000000000000000000000000000000000000000000000000000000000000000000000000000000000000000000000000000000000000000000000000000000000000000000000000000000000000000000000000000000000000000000000000000000000000000000000000000000000000000000000000000000000000000000000000000000000000000000000000000000000000000000000000000000000000000000000000000000000000000000000000000000000000000000000000000000000000000000000000000000000000000011111111

>DM_train132

MKHMPRKMYSCAFETTTKVEDCRVWAYGYMNIEDHSEYKIGNSLDEFMAWVLKVQADLYFHNLKFAGAFIINWLERNGFKWSADGLPNTYNTIISRMGQWYMIDICLGYKGKRKIHTVIYDSLKKLPFPVKKIAKDFKLTVLKGDIDYHKERPVGYKITPEEYAYIKNDIQIIAEALLIQFKQGLDRMTAGSDSLKGFKDIITTKKFKKVFPTLSLGLDKEVRYAYRGGFTWLNDRFKEKEIGEGMVFDVNSLYPAQMYSRLLPYGEPIVFEGKYVWDEDYPLHIQHIRCEFELKEGYIPTIQIKRSRFYKGNEYLKSSGGEIADLWLSNVDLELMKEHYDLYNVEYISGLKFKATTGLFKDFIDKWTYIKTTSEGAIKQLAKLMLNSLYGKFASNPDVTGKVPYLKENGALGFRLGEEETKDPVYTPMGVFITAWARYTTITAAQACYDRIIYCDTDSIHLTGTEIPDVIKDIVDPKKLGYWAHESTFKRAKYLRQKTYIQDIYMKEVDGKLVEGSPDDYTDIKFSVKCAGMTDKIKKEVTFENFKVGFSRKMKPKPVQVPGGVVLVDDTFTIK

11110000000000000000000000000000000000000000000000000000000000000000000000000000000000000000000000000000000000000000000000000000000000000000000000000000000000000000000000000000000000000000000000000000000000000000000000000000000000000000000000000000000000000000000000000000000000000000000000000000000000001111111110000000000000000000000000000000000000000000000000000000000000000000000000000000000000000000000000000000000000000000000000000000000000000000000000000000000000000000000000000000000000000000000000000000000000000000000000000000000000000000000000000000000000000000000

>DM_train133

HGTTANTHCGADFCTWWHDSGEINTQTPVQPGNVRQSHKYSVQVSLAGTNNFHDSFVYESIPRNGNGRIYAPTDPPNSNTLDSSVDDGISIEPSIGLNMAWSQFEYSHDVDVKILATDGSSLGSPSDVVIRPVSISYAISQSDDGGIVIRVPADANGRKFSVEFKTDLYTFLSDGNEYVTSGGSVVGVEPTNALVIFASPFLPSGMIPHMTPDNTQTMTPGPINNGDWGAKSILYFPPGVYWMNQDQSGNSGKLGSNHIRLNSNTYWVYLAPGAYVKGAIEYFTKQNFYATGHGILSGENYVYQANAGDNYIAVKSDSTSLRMWWHNNLGGGQTWYCVGPTINAPPFNTMDFNGNSGISSQISDYKQVGAFFFQTDGPEIYPNSVVHDVFWHVNDDAIKIYYSGASVSRATIWKCHNDPIIQMGWTSRDISGVTIDTLNVIHTRYIKSETVVPSAIIGASPFYASGMSPDSRKSISMTVSNVVCEGLCPSLFRITPLQNYKNFVVKNVAFPDGLQTNSIGTGESIIPAASGLTMGLAISAWTIGGQKVTMENFQANSLGQFNIDGSYWGEWQIS

1100000000000000000000000000000000000000000000000000000000000000000000000000000000000000000000000000000000000000000000000000000000000000000000000000000000000000000000000000000000000000000000000000000000000000000000000000000000000000000000000000000000000000000000000000000000000000000000000000000000000000000000000000000000000000000000000000000000000000000000000000000000000000000000000000000000000000000000000000000000000000000000000000000000000000000000000000000000000000000000000000000000000000000000000000000000000000000000000000000000000000000000000000000000000000000000

>DM_train137

SNISRQAYADMFGPTVGDKVRLADTELWIEVEDDLTTYGEEVKFGGGKVIRDGMGQGQMLAADCVDLVLTNALIVDHWGIVKADIGVKDGRIFAIGKAGNPDIQPNVTIPIGAATEVIAAEGKIVTAGGIDTHIHWICPQQAEEALVSGVTTMVGGGTGPAAGTHATTCTPGPWYISRMLQAADSLPVNIGLLGKGNVSQPDALREQVAAGVIGLCIHEDWGATPAAIDCALTVADEMDIQVALHSDTLNESGFVEDTLAAIGGRTIHTFHTEGAGGGHAPDIITACAHPNILPSSTNPTLPYTLNTIDEHLDMLMVAHHLDPDIAEDVAFAESRIRRETIAAEDVLHDLGAFSLTSSDSQAMGRVGEVILRTWQVAHRMKVQRGALAEETGDNDNFRVKRYIAKYTINPALTHGIAHEVGSIEVGKLADLVVWSPAFFGVKPATVIKGGMIAIAPMGDINASIPTPQPVHYRPMFGALGSARHHCRLTFLSQAAAANGVAERLNLRSAIAVVKGCRTVQKADMVHNSLQPNITVDAQTYEVRVDGELITSEPADVLPMAQRYFLF

00000000000000000000000000000000000000000000000000000000000000000000000000000000000000000000000000000000000000000000000000000000000000000000000000000000000000000000000000000000000000000000000000000000000000000000000000000000000000000000000000000000000000000000000000000000000000000000000000000000000000000001111111111111111111111111111110000000000000000000000000000000000000000000000000000000000000000000000000000000000000000000000000000000000000000000000000000000000000000000000000000000000000000000000000000000000000000000000000000000000000000000000000000000000000

>DM_train138

GHMNIESTLNSVASVKDLANEASKYEIILQKGINQVGLKQYTQVVHKLDDMLEDIQSGQANREENSEFHGILTHLEQLIKRSEAQLRVYFISILNSIKPFDPQINITKKMPFPYYEDQQLGALSWILDYFHGNSEGSIIQDILVGERSKLILKCMAFLEPFAKEISTAKNAPYEKGSSGMNSYTEALLGFIANEKSLVDDLYSQYTESKPHVLSQILSPLISAYAKLFGANLKIVRSNLENFGFFSFELVESINDVKKSLRGKELQNYNLLQDCTQEVRQVTQSLFRDAIDRIIKKANSISTIPSNNGVTEATVDTMSRLRKFSEYKNGCLGAMDNITRENWLPSNYKEKEYTLQNEALNWEDHNVLLSCFISDCIDTLAVNLERKAQIALMPNQEPDVANPNSSKNKHKQRIGFFILMNLTLVEQIVEKSELNLMLAGEGHSRLERLKKRYISYMVSDWRDLTANLMDSVFIDSSGKKSKDKEQIKEKFRKFNEGFEDLVSKTKQYKLSDPSLKVTLKSEIISLVMPMYERFYSRYKDSFKNPRKHIKYTPDELTTVLNQLVR

111111100000000000000000000000000000000000000000000000000111100000000000000000000000000000000000000000000000000000000000000000000000000000000000000000000000000000111111111111000000000000000000000000000000000000000000000000000000000000001111100000000000000000000000000000000000000000000000000000000000000000000000000000000000000000000000000000000000000000011110000000000000000000000000000000000000000000000000000000000000000000000000000000000000000000000000000000000000000000000000000000000000000000000000000000000000000000000000000000000000000000000000000000000000

>DM_train140

MTTAARRPAPTTAGAGWDAGVGALVNPSRRRGGTLRLVSSADVDSLDPARTYYVWVWLLQRLLNRTLMAYPTDPGPAGLVPAPDLAEGPGEVSDGGRTWTYRLRRGLRYDDGTPITSDDVRHAVQRVFAQDVLPGGPTYLIPLLDDPERPYPGPYRTDEPLRSVLTPDEHTIVFRLTRPFSDFDHLMAQPCAAPVPRRSDTGADYGRDPRSSGPYRVARHEPDTLLHLERNPHWDRATDPIRPALPDRVELTIGLDVDVLDARLIAGEFDINLEGRGLQHAAQRRATADEVLRSHTDNPRTSFLHFVAMQPHIPPFDNVHVRRAVQYAADKILLQDARGGPVNGGDLTTALFPPTLPAHQDLDLYPTGPDLRGDLDAARAELAAAGLPDGFRAVIGTQRGKFRLVADAVVESLARVGIELTVKELDVATYFSLGAGHPETVREHGLGLLVTDWGADFPTEYGFLAPLVDGRQIKRNGGNWNLPELDDPEVNALIDETLHTTDPAARAELWRAVERRVMEHAVLLPLVHDKTLHFRNPWVTNVYVHPAFGLYDIQAMGLAEED

1111111000000000000000000000000000000000000000000000000000000000000000000000000000000000000000000000000000000000000000000000000000000000000000000000000000000000000000000000000000000000000000000000000000000000000000000000000000000000000000000000000000000000000000000000000000000000000000000000000000000000000000000000000000000000000000000000000000000000000000000000000000000000000000000000000000000000000000000000000000000000000000000000000000000000000000000000000000000000000000000000000000000000000000000000000000000000000000000000000000000000000000000000000011

>DM_train141

STGPVAPLPTPPNFPNDIALFQQAYQNWSKEIMLDATWVCSPKTPQDVVRLANWAHEHDYKIRPRGAMAGWTPLTVEKGANVEKVILADTMTHLNGITVNTGGPVATVTAGAGASIEAIVTELQKHDLGWANLPAPGVLSIGGALAVNAHGAALPAVGQTTLPGHTYGSLSNLVTELTAVVWNGTTYALETYQRNDPRITPLLTNLGRCFLTSVTMQAGPNFRQRCQSYTDIPWRELFAPKGADGRTFEKFVAESGGAEAIWYPFTEKPWMKVWTVSPTKPDSSNEVGSLGSAGSLVGKPPQAREVSGPYNYIFSDNLPEPITDMIGAINAGNPGIAPLFGPAMYEITKLGLAATNANDIWGWSKDVQFYIKATTLRLTEGGGAVVTSRANIATVINDFTEWFHERIEFYRAKGEFPLNGPVEIRCCGLDQAADVKVPSVGPPTISATRPRPDHPDWDVAIWLNVLGVPGTPGMFEFYREMEQWMRSHYNNDDATFRPEWSKGWAFGPDPYTDNDIVTNKMRATYIEGVPTTENWDTARARYNQIDPHRVFTNGFMDKLLP

111110000000000000000000000000000000000000000000000000000000000000000000000000000000000000000000000000000000000000000000000000000000000000000000000000000000000000000000000000000000000000000000000000000000000000000000000000000000000000000000000000000000000000000000000000000000011111111111111111111000000000000000000000000000000000000000000000000000000000000000000000000000000000000000000000000000000000000000000000000000000000000000000000000000000000000000000000000000000000000000000000000000000000000000000000000000000000000000000000000000000000000000000000000

>DM_train142

YDPDQYSIEADKKFKYSVKLSDYPTLQDAASAAVDGLLIDRDYNFYGGETVDFGGKVLTIECKAKFIGDGNLIFTKLGKGSRIAGVFMESTTTPWVIKPWTDDNQWLTDAAAVVATLKQSKTDGYQPTVSDYVKFPGIETLLPPNAKGQNITSTLEIRECIGVEVHRASGLMAGFLFRGCHFCKMVDANNPSGGKDGIITFENLSGDWGKGNYVIGGRTSYGSVSSAQFLRNNGGFERDGGVIGFTSYRAGGSGVKTWQGTVGSTTSRNYNLQFRDSVVIYPVWDGFDLGADTDMNPELDRPGDYPITQYPLHQLPLNHLIDNLLVRGALGVGFGMDGKGMYVSNITVEDCAGSGAYLLTHESVFTNIAIIDTNTKDFQANQIYISGACRVNGLRLIGIRSTDGQSLTIDAPNSTVSGITGMVDPSRINVANLAEEGLGNIRANSFGYDSAAIKLRIHKLSKTLDSGALYSHINGGAGSGSAYTQLTAISGSTPDAVSLKVNHKDCRGAEIPFVPDIASDDFIKDSSCFLPYWENNSTSLKALVKKPNGELVRLTLATL

1111100000000000000000000000000000000000000000000000000000000000000000000000000000000000000000000000000000000000000000000000000000000000000000000000000000000000000000000000000000000000000000000000000000000000000000000000000000000000000000000000000000000000000000000000000000000000000000000000011111100000000000000000000000000000000000000000000000000000000000000000000000000000000000000000000000000000111110000000000000000000000000000000000000000000000000000000000000000000000000000000000000000000000000000000000000000000000000000000000000000000000000000000000

>DM_train143

MTTANTPVRPKSAIDAVADAYTEKLIELNPSFATTLGLPGHETEYQDYSPAGAAAHAEATRLALEALAGLEPSDDVDAVTLDAMRERLGLELEIHQSGWDAADLNNIASPAQDIRAIFDLMPTDTVEHWEHIAGRAANVPGAIEGYIASLRAAKDDRKVAAARQIRIVIEQTGRYAAEDGFFAKMAADASLGDAPLPAEVQDKLDAGTSAARSAYSALGAFLRDELLPVAPEKDAVGRERYSLASRSFIGAEVDLEETYAWGVQELERLISEQEKVAGQIKPGASIEEAKSILNNDPARQIKGTDALKAWMQELSDRAVSELADVHFDIPDVMKTLECMIAPTDEGGIYYTGPSDDFSRPGRMWWSVPAGEDTFTTWSETTTVFHEGVPGHHLQVATATYRRELLNNWRRNVCWVSGHGEGWALYAEQLMLELGYLKDPGDHMGMLDGQRMRAARVVFDIGVHLELPVPERWGTGTWTPEKGFDFLKANLDISEGQLQFEFTRYLGWPGQAPSYKVGQRLWEQIRAELESREGFDLKSFHSKALNIGSVGLDVLRRALL

1111111000000000000000000000000000000000000000000000000000000000000000000000000000000000000000000000000000000000000000000000000000000000000000000000000000000000000000000000000000000000000000000000000000000000000000000000000000000000000000000000000000000000000000000000000000000000000000000000000000000000000000000000000000000000000000000000000011000000000000000000000000000000000000000000000000000000000000000000000000000000000000000000000000000000000000000000000000000000000000000000000000000000000000000000000000000000000000000000000000000000000000000000000

>DM_train144

MSKVPSDIEIAQAAKMKPVMELARGLGIQEDEVELYGKYKAKISLDVYRRLKDKPDGKLILVTAITPTPAGEGKTTTSVGLTDALARLGKRVMVCLREPSLGPSFGIKGGAAGGGYAQVVPMEDINLHFTGDIHAVTYAHNLLAAMVDNHLQQGNVLNIDPRTITWRRVIDLNERALRNIVIGLGGKANGVPRETGFDISVASEVMACLCLASDLMDLKERFSRKVVGYTYDGKPVTAGDLEAQGSMALLMKDAIKPNLVQTLENTPAFIHGGPFANIAHGCNSIIATKTALKLADYVVTEAGFGADLGAEKFYDVKCRYAGFKPDATVIVATVRALKMHGGVPKSDLATENLEALREGFANLEKHIENIGKFGVPAVVAINAFPTDTEAELNLLYELCAKAGAEVALSWAKGGEGGLELARKVLQTLESRPSNFHVLYNLDLSIKDKIAKIATEIYGADGVNYTAEADKAIQRYESLGYGNLPVVMAKTQYSFSDDMTKLGRPRNFTITVREVRLSAGGRLIVPITGAIMTMPGLPKRPAACNIDIDADGVITGLF

11111110000000000000000000000000000000000000000000000000000000000000000000000000000000000000000000000000000000000000000000000000000000000000000000000000000000000000000000000000000000000000000000000000000000000000000000000000000000000000000000000000000000000000000000000000000000000000000000000000000000000000000000000000000000000000000000000000000000000000000000000000000000000000000000000000000000000000000000000000000000000000000000000000000000000000000000000000000000000000000000000000000000000000000000000000000000000000000000000000000000000000000000000

>DM_train145

MSVSAFNRRWAAVILEALTRHGVRHICIAPGSRSTLLTLAAAENSAFIHHTHFDERGLGHLALGLAKVSKQPVAVIVTSGTAVANLYPALIEAGLTGEKLILLTADRPPELIDCGANQAIRQPGMFASHPTHSISLPRPTQDIPARWLVSTIDHALGTLHAGGVHINCPFAEPLYGEMDDTGLSWQQRLGDWWQDDKPWLREAPRLESEKQRDWFFWRQKRGVVVAGRMSAEEGKKVALWAQTLGWPLIGDVLSQTGQPLPCADLWLGNAKATSELQQAQIVVQLGSSLTGKRLLQWQASCEPEEYWIVDDIEGRLDPAHHRGRRLIANIADWLELHPAEKRQPWCVEIPRLAEQAMQAVIARRDAFGEAQLAHRICDYLPEQGQLFVGNSLVVRLIDALSQLPAGYPVYSNRGASGIDGLLSTAAGVQRASGKPTLAIVGDLSALYDLNALALLRQVSAPLVLIVVNNNGGQIFSLLPTPQSERERFYLMPQNVHFEHAAAMFELKYHRPQNWQELETAFADAWRTPTTTVIEMVVNDTDGAQTLQQLLAQVSHL

1000000000000000000000000000000000000000000000000000000000000000000000000000000000000000000000000000000000000000000000000000000000000000000000000000000000000000000000000000001100000000000000000000000000000000000000000000000000000000000000000000000000000000000000000000000000000000000000000000000000000000000000000000000000000000000000000000000000000000000000000000000000000000000000000000000000000000000000000000000000000000000000000000000000000000000000000000000000000001111111111111111111110000000000000000000000000000000000000000000000000000000000000000

>DM_train146

MKRSKRFAVLAQRPVNQDGLIGEWPEEGLIAMDSPFDPVSSVKVDNGLIVELDGKRRDQFDMIDRFIADYAINVERTEQAMRLEAVEIARMLVDIHVSREEIIAITTAITPAKAVEVMAQMNVVEMMMALQKMRARRTPSNQCHVTNLKDNPVQIAADAAEAGIRGFSEQETTVGIARYAPFNALALLVGSQCGRPGVLTQCSVEEATELELGMRGLTSYAETVSVYGTEAVFTDGDDTPWSKAFLASAYASRGLKMRYTSGTGSEALMGYSESKSMLYLESRCIFITKGAGVQGLQNGAVSCIGMTGAVPSGIRAVLAENLIASMLDLEVASANDQTFSHSDIRRTARTLMQMLPGTDFIFSGYSAVPNYDNMFAGSNFDAEDFDDYNILQRDLMVDGGLRPVTEAETIAIRQKAARAIQAVFRELGLPPIADEEVEAATYAHGSNEMPPRNVVEDLSAVEEMMKRNITGLDIVGALSRSGFEDIASNILNMLRQRVTGDYLQTSAILDRQFEVVSAVNDINDYQGPGTGYRISAERWAEIKNIPGVVQPDTIE

000000000000000000000000000000000000000000000000000000000000000000000000000000000000000000000000000000000000000000000000000000000000000000000000000000000000000000000000000000000000000000000000000000000000000000000000000000000000000000000000000000000000000000000000000000000000000000000000000000000000000000000000000000000000000000000000000000000000000000000000000000000000000000000000000000000000000000000000000000000000000000000000000000000000000000000000000000000000000000000000000000000000000000000000000000000000000000000000000000000000000000000000111

>DM_train147

ANFNVPKLGVFPVAAVFDIDNVPEDSSATGSRWLPSIYQGGNYWGGGPQALHAQVSNFDSSNRLPYNPRTENNPAGNCAFAFNPFGQYISNISSAQSVHRRIYGIDLNDEPLFSPNAASITNGGNPTMSQDTGYHNIGPINTAYKAEIFRPVNPLPMSDTAPDPETLEPGQTEPLIKSDGVYSNSGIASFIFDRPVTEPNPNWPPLPPPVIPIIYPTPALGIGAAAAYGFGYQVTVYRWEEIPVEFIADPETCPAQPTTDKVIIRTTDLNPEGSPCAYEAGIILVRQTSNPMNAVAGRLVPYVEDIAVDIFLTGKFFTLNPPLRITNNYFADDEVKENTVTIGNYTTTLSSAYYAVYKTDGYGGATCFIASGGAGISALVQLQDNSVLDVLYYSLPLSLGGSKAAIDEWVANNCGLFPMSGGLDKTTLLEIPRRQLEAINPQDGPGQYDLFILDDSGAYASFSSFIGYPEAAYYVAGAATFMDVENPDEIIFILRNGAGWYACEIGDALKIADDEFDSVDYFAYRGGVMFIGSARYTEGGDPLPIKYRAIIPGLP

000000000000000000000000000000000000000000000000000000000000000000000000000000000000000000000000000000000000000000000000000000000000000000000000000000000000000000000000000000000000000000000000000000000000000000000000000000000000000000000000000000111111111111111111111100000000000000000000000000000000000000000000000000000000000000000000000000000000000000000000000000000000000000000000000000000000000000000000000000000000000000000000000000000000000000000000000000000000000000000000000000000000000000000000000000000000000000000000000000000000000000000000000

>DM_train148

MNTSLFKQERQKYIPKLPNILKKDFNNISLVYGENTEAIQDRQALKEFFKNTYGLPIISFTEGESSLSFSKALNIGIILSGGPAPGGHNVISGVFDAIKKFNPNSKLFGFKGGPLGLLENDKIELTESLINSYRNTGGFDIVSSGRTKIETEEHYNKALFVAKENNLNAIIIIGGDDSNTNAAILAEYFKKNGENIQVIGVPKTIDADLRNDHIEISFGFDSATKIYSELIGNLCRDAMSTKKYWHFVKLMGRSASHVALECALKTHPNICIVSEEVLAKKKTLSEIIDEMVSVILKRSLNGDNFGVVIVPEGLIEFIPEVKSLMLELCDIFDKNEGEFKGLNIEKMKEIFVAKLSDYMKGVYLSLPLFIQFELIKSILERDPHGNFNVSRVPTEKLFIEMIQSRLNDMKKRGEYKGSFTPVDHFFGYEGRSAFPSNFDSDYCYSLGYNAVVLILNGLTGYMSCIKNLNLKPTDWIAGGVPLTMLMNMEERYGEKKPVIKKALVDLEGRPFKEFVKNRDKWALNNLYLYPGPVQYFGSSEIVDEITETLKLELFK

000000000000000000000000000000000000000000000000000000000000000000000000000000000000000000000000000000000000000000000000000000000000000000000000000000000000000000000000000000000000000000000000000000000000000000000000000000000000000000000000000000000000000000000000000000000000000000000000000000000000000000000000000000000000000000001111111111100000000000000000000000000000000000001111111111000000000000000000000000000000000000000000000000000000000000000000000000000000000000000000000000000000000000000000000000000000000000000000000000000000000000000000000

>DM_train149

GSHMPVVHVIDVESGNLQSLTNAIEHLGYEVQLVKSPKDFNISGTSRLILPGVGNYGHFVDNLFNRGFEKPIREYIESGKPIMGICVGLQALFAGSVESPKSTGLNYIDFKLSRFDDSEKPVPEIGWNSCIPSENLFFGLDPYKRYYFVHSFAAILNSEKKKNLENDGWKIAKAKYGSEEFIAAVNKNNIFATQFHPEKSGKAGLNVIENFLKQQSPPIPNYSAEEKELLMNDYSNYGLTRRIIACLDVRTNDQGDLVVTKGDQYDVREKSDGKGVRNLGKPVQLAQKYYQQGADEVTFLNITSFRDCPLKDTPMLEVLKQAAKTVFVPLTVGGGIKDIVDVDGTKIPALEVASLYFRSGADKVSIGTDAVYAAEKYYELGNRGDGTSPIETISKAYGAQAVVISVDPKRVYVNSQADTKNKVFETEYPGPNGEKYCWYQCTIKGGRESRDLGVWELTRACEALGAGEILLNCIDKDGSNSGYDLELIEHVKDAVKIPVIASSGAGVPEHFEEAFLKTRADACLGAGMFHRGEFTVNDVKEYLLEHGLKVRMDEE

000000000000000000000000000000000000000000000000000000000000000000000000000000000000000000000000000000000000000000000000000000000000000000000000000000000000000000000000000000000000000000000000000000000000000000000000000000000000000000000000000000000000000000011111111111111111110000000000000000000000000111000000000000000000000000000000000000000000000000000000000000000000000000000000000000000000000000000000000000000000000000000000000000000000000000000000000000000000000000000000000000000000000000000000000000000000000000000000000000000000000000000000000

>DM_train151

MGDYDIPTTENLYFQGMTDLNKLVKELNDLGLTDVKEIVYNPSYEQLFEEETKPGLEGFDKGTLTTLGAVAVDTGIFTGRSPKDKYIVCDETTKDTVWWNSEAAKNDNKPMTQETWKSLRELVAKQLSGKRLFVVEGYCGASEKHRIGVRMVTEVAWQAHFVKNMFIRPTDEELKNFKADFTVLNGAKCTNPNWKEQGLNSENFVAFNITEGIQLIGGTWYGGEMKKGMFSMMNYFLPLKGVASMHCSANVGKDGDVAIFFGLSGTGKTTLSTDPKRQLIGDDEHGWDESGVFNFEGGCYAKTINLSQENEPDIYGAIRRDALLENVVVRADGSVDFDDGSKTENTRVSYPIYHIDNIVRPVSKAGHATKVIFLTADAFGVLPPVSKLTPEQTEYYFLSGFTAKLAGTERGVTEPTPTFSACFGAAFLSLHPIQYADVLVERMKASGAEAYLVNTGWNGTGKRISIKDTRGIIDAILDGSIEKAEMGELPIFNLAIPKALPGVDPAILDPRDTYADKAQWQVKAEDLANRFVKNFVKYTANPEAAKLVGAGPKA

11111111111111111100000000000000000000000000000000000000000000000000000000000000000000000000000000000000000000000000000000000000000000000000000000000000000000000000000000000000000000000000000000000000000000000000000000000000000000000000000000000000000000000000000000000000000000000000000000000000000000000000000000000000000000000000000000000000000000000000000000000000000000000000000000000000000000000001111111111100000000000000000000000000000000000000000000000000000000000000000000000000000000000000000000000000000000000000000000000000000000000000000001

>DM_train152

MSALLLKPHKDLPRRTVLIVVMDGLGIGPEDDYDAVHMASTPFMDAHRRDNRHFRCVRAHGTAVGLPTDADMGNSEVGHNALGAGRVALQGASLVDDAIKSGEIYTGEGYRYLHGAFSKEGSTLHLIGLLSDGGVHSRDNQIYSIIEHAVKDGAKRIRVHALYDGRDVPDGSSFRFTDELEAVLAKVRQNGCDAAIASGGGRMFVTMDRYDADWSIVERGWRAQVLGDARHFHSAKEAITTFREEDPKVTDQYYPPFIVVDEQDKPLGTIEDGDAVLCVNFRGDRVIEMTRAFEDEDFNKFDRVRVPKVRYAGMMRYDGDLGIPNNFLVPPPKLTRVSEEYLCGSGLNIFACSETQKFGHVTYFWNGNRSGKIDEKHETFKEVPSDRVQFNEKPRMQSAAITEAAIEALKSGMYNVVRINFPNGDMVGHTGDLKATITGVEAVDESLAKLKDAVDSVNGVYIVTADHGNSDDMAQRDKKGKPMKDGNGNVLPLTSHTLSPVPVFIGGAGLDPRVAMRTDLPAAGLANVTATFINLLGFEAPEDYEPSLIYVEK

1110000000000000000000000000000000000000000000000000000000000000000000000000000000000000000000000000000000000000000000000000000000000000000000000000000000000000000000000000000000000000000000000000000000000000000000000000000000000000000000000000000000000000000000000000000000000000000000000000000000000000000000000000000000000000000000000000000000000000000000000000000000000000000000000000000000000000000000000000000000000000000000000000000000000000000000000000000000000000000000000000000000000000000000000000000000000000000000000000000000000000000000001

>DM_train153

MSDMEKPWKEEEKREVLAGHARRQAPQAVDKGPVTGDQRISVTVVLRRQRGDELEAHVERQAALAPHARVHLEREAFAASHGASLDDFAEIRKFAEAHGLTLDRAHVAAGTAVLSGPVDAVNQAFGVELRHFDHPDGSYRSYVGDVRVPASIAPLIEAVLGLDTRPVARPHFRLRRRAEGEFEARSQSAAPTAYTPLDVAQAYQFPEGLDGQGQCIAIIELGGGYDETSLAQYFASLGVSAPQVVSVSVDGATNQPTGDPNGPDGEVELDIEVAGALAPGAKIAVYFAPNTDAGFLNAITTAVHDPTHKPSIVSISWGGPEDSWAPASIAAMNRAFLDAAALGVTVLAAAGDSGSTDGEQDGLYHVDFPAASPYVLACGGTRLVASAGRIERETVWNDGPDGGSTGGGVSRIFPLPSWQERANVPPSANPGAGSGRGVPDVAGNADPATGYEVVIDGETTVIGGTAAVAPLFAALVARINQKLGKPVGYLNPTLYQLPPEVFHDITEGNNDIANRARIYQAGPGWDPCTGLGSPIGIRLLQALLPSASQAQP

111111111110000000000000000000000000000000000000000000000000000000000000000000000000000000000000000000000000000000000000000000000000000000000000000000000000000000000000000000000000000000000000000000000000000000000000000000000000000000000000000000000000000000000000000000000000000000000000000000000000000000000000000000000000000000000000000000000000000000000000000000000000000000000000000000000000000000000000000000000000000000000000000000000000000000000000000000000000000000000000000000000000000000000000000000000000000000000000000000000000000001111111

>DM_train154

MKRREVLQAGMALGGLAGLGRALAQGGFTLTLVHTNDTHAHLEPVELTLSGEKTPVGGVARRVALFDRVWARAKNPLFLDAGDVFQGTLYFNQYRGLADRYFMHRLRYRAMALGNHEFDLGPGPLADFLKGARFKVVSANVDASREPRLKGLFAPYAVVVVGGERVGIIGLTTPDTREISNPGPTVAFLDPYESAQKAVYELLAKGVNKIVVLSHLGYGEDLKLARRLVGVQVIVGGHSHTLLGSFPHKELSPAGPYPTVVKNPEGKDVLVVQAWEWGKVVGLLEVTFDAKGELLAYKGEALLMTPEAAPEDFFAKEALLAYAQPVMALMQQVIAEAKVDLVGERAVVRRRESNLGNLITDGMLWKTRNAGTQIALQNGGGIRASIPKGPITVGKVYEVLPFGNTLVVMDLKGKEILAALENGVSQWENTAGRFLQVSGLRYAFDLSRPAGSRVVRVEVKTEKGYVPLDLEATYRVVVNNFIANGGDGFTVLKEAQGYRVDTGFSDAESFMDYLKELKVVEAGLEGRIEVLNEPKGERPAYFAYRVPGLVGV

111111111111111111111111111000000000000000000000000000000000000000000000000000000000000000000000000000000000000000000000000000000000000000000000000000000000000000000000000000000000000000000000000000000000000000000000000000000000000000000000000000000000000000000000000000000000000000000000000000000000000000000000000000000000000000000000000000000000000000000000000000000000000000000000000000000000000000000000000000000000000000000000000000000000000000000000000000000000000000000000000000000000000000000000000000000000000000000000000000111111111111111111

>DM_train155

MTQVAKKILVTCALPYANGSIHLGHMLEHIQADVWVRYQRMRGHEVNFICADDAHGTPIMLKAQQLGITPEQMIGEMSQEHQTDFAGFNISYDNYHSTHSEENRQLSELIYSRLKENGFIKNRTISQLYDPEKGMFLPDRFVKGTCPKCKSPDQYGDNCEVCGATYSPTELIEPKSVVSGATPVMRDSEHFFFDLPSFSEMLQAWTRSGALQEQVANKMQEWFESGLQQWDISRDAPYFGFEIPNAPGKYFYVWLDAPIGYMGSFKNLCDKRGDSVSFDEYWKKDSTAELYHFIGKDIVYFHSLFWPAMLEGSNFRKPSNLFVHGYVTVNGAKMSKSRGTFIKASTWLNHFDADSLRYYYTAKLSSRIDDIDLNLEDFVQRVNADIVNKVVNLASRNAGFINKRFDGVLASELADPQLYKTFTDAAEVIGEAWESREFGKAVREIMALADLANRYVDEQAPWVVAKQEGRDADLQAICSMGINLFRVLMTYLKPVLPKLTERAEAFLNTELTWDGIQQPLLGHKVNPFKALYNRIDMRQVEALVEASKEEV

11110000000000000000000000000000000000000000000000000000000000000000000000000000000000000000000000000000000000000000000000000000000000000000000000000000000000000000000000000000000000000000000000000000000000000000000000000000000000000000000000000000000000000000000000000000000000000000000000000000000000000000000000000000000000000000000000000000000000000000000000000000000000000000000000000000000000000000000000000000000000000000000000000000000000000000000000000000000000000000000000000000000000000000000000000000000000000000000000000000000000000000000

>DM_train158

PTVSATPYDYIIVGAGPGGIIAADRLSEAGKKVLLLERGGPSTKQTGGTYVAPWATSSGLTKFDIPGLFESLFTDSNPFWWCKDITVFAGCLVGGGTSVNGALYWYPNDGDFSSSVGWPSSWTNHAPYTSKLSSRLPSTDHPSTDGQRYLEQSFNVVSQLLKGQGYNQATINDNPNYKDHVFGYSAFDFLNGKRAGPVATYLQTALARPNFTFKTNVMVSNVVRNGSQILGVQTNDPTLGPNGFIPVTPKGRVILSAGAFGTSRILFQSGIGPTDMIQTVQSNPTAAAALPPQNQWINLPVGMNAQDNPSINLVFTHPSIDAYENWADVWSNPRPADAAQYLANQSGVFAGASPKLNFWRAYSGSDGFTRYAQGTVRPGAASVNSSLPYNASQIFTITVYLSTGIQSRGRIGIDAALRGTVLTPPWLVNPVDKTVLLQALHDVVSNIGSIPGLTMITPDVTQTLEEYVDAYDPATMNSNHWVSSTTIGSSPQSAVVDSNVKVFGTNNLFIVDAGIIPHLPTGNPQGTLMSAAEQAAAKILALAGGP

111110000000000000000000000000000000000000000000000000000000000000000000000000000000000000000000000000000000000000000000000000000000000000000000000000000000000000000000000000000000000000000000000000000000000000000000000000000000000000000000000000000000000000000000000000000000000000000000000000000000000000000000000000000000000000000000000000000000000000000000000000000000000000000000000000000000000000000000000000000000000000000000000000000000000000000000000000000000000000000000000000000000000000000000000000000000000000000000000000000000000000

>DM_train159

MHIDNIENLSDREFDYIVVGGGSAGAAVAARLSEDPAVSVALVEAGPDDRGVPEVLQLDRWMELLESGYDWDYPIEPQENGNSFMRHARAKVMGGCSSHNSCIAFWAPREDLDEWEAKYGATGWNAEAAWPLYKRLETNEDAGPDAPHHGDSGPVHLMNVPPKDPTGVALLDACEQAGIPRAKFNTGTTVVNGANFFQINRRADGTRSSSSVSYIHPIVEQENFTLLTGLRARQLVFDADRRCTGVDIVDSAFGHTHRLTARNEVVLSTGAIDTPKLLMLSGIGPAAHLAEHGIEVLVDSPGVGEHLQDHPEGVVQFEAKQPMVAESTQWWEIGIFTPTEDGLDRPDLMMHYGSVPFDMNTLRHGYPTTENGFSLTPNVTHARSRGTVRLRSRDFRDKPMVDPRYFTDPEGHDMRVMVAGIRKAREIAAQPAMAEWTGRELSPGVEAQTDEELQDYIRKTHNTVYHPVGTVRMGAVEDEMSPLDPELRVKGVTGLRVADASVMPEHVTVNPNITVMMIGERCADLIRSARAGETTTADAELSAALA

000000000000000000000000000000000000000000000000000000000000000000000000000000000000000000000000000000000000000000000000000000000000000000000000000000000000000000000000000000000000000000000000000000000000000000000000000000000000000000000000000000000000000000000000000000000000000000000000000000000000000000000000000000000000000000000000000000000000000000000000000000000000000000000000000000000000000000000000000000000000000000000000000000000000000000000000000000000000000000000000000000000000000000000000000000000000000000000001111111111111111111

>DM_train160

ASFPHRNLTWNDINFVHTTDTHGWYSGHINQPLYHANWGDFISFTTHMRRIAHSRNQDLLLIDSGDRHDGNGLSDITSPNGLKSTPIFIKQDYDLLTIGNHELYLWENSKQEYETVVNHFQDKYVCSNVDIRLDNGLFVPLGLKYKYFTTPIRGIRVMAFGFLFDFKRFNSGTRVTPMAETIHEPWFQEALKHEVDLIIIVGHTPISHNWGEFYQVHQYLRQFFPDTIIQYFGGHSHIRDFTVFDSLSTGLQSGRYCETVGWTSVNLDKADLNLPVRQRFSRSYIDFNTDSFKYHTNLDKEFDTAKGKLVSKLIRETRKELKLDTLIGYVKTNYYVDYVPIDHPKSIFNLLALKILKTLPKSKHEERITIINTGSIRYDLYKGPYTIDSKFIVSPFENIWVNITVPKSVATKVAAKLNDADYISASRLKPPHQYDLQVQDLSTSPHQAHFEMQEKLPKGYVTHDDFGADGDDTLHRAVVNFPVPNVIQSVEINDEVDSPVNLVFYSFITPNIIWALKELNFSTEQVPTPYSDIYLGTLLNEFVANN

100000000000000000000000000000000000000000000000000000000000000000000000000000000000000000000000000000000000000000000000000000000000000000000000000000000000000000000000000000000000000000000000000000000000000000000000000000000000000000000000000000000000000000000000000000000000000000000000000000000000000000000000000000000000000000000000000000000000000000000000000000000000000000000000000000000000000000000000000000000001100000000000000000011111111111110000000000000000000000000000000000000000000000000000000000000000000000000000000000000000000000

>DM_train161

MAAKEVKFNSDARDRMLKGVNILADAVKVTLGPKGRNVVIDKSFGAPRITKDGVSVAKEIELSDKFENMGAQMVREVASRTNDEAGDGTTTATVLAQAIVREGLKAVAAGMNPMDLKRGIDVATAKVVEAIKSAARPVNDSSEVAQVGTISANGESFIGQQIAEAMQRVGNEGVITVEENKGMETEVEVVEGMQFDRGYLSPYFVTNADKMIAELEDAYILLHEKKLSSLQPMVPLLESVIQSQKPLLIVAEDVEGEALATLVVNKLRGGLKIAAVKAPGFGDRRKAMLQDIAILTGGQVISEDLGMKLENVTIDMLGRAKKVSINKDNTTIVDGAGEKAEIEARVSQIRQQIEETTSDYDREKLQERVAKLAGGVAVIRVGGMTEIEVKERKDRVDDALNATRAAVQEGIVVGGGVALVQGAKVLEGLSGANSDQDAGIAIIRRALEAPMRQIAENAGVDGAVVAGKVRESSDKAFGFNAQTEEYGDMFKFGVIDPAKVVRTALEDAASVAGLLITTEAMIAEKPEPKAPAGGMPDMGGMGGMM

00000000000000000000000000000000000000000000000000000000000000000000000000000000000000000000000000000000000000000000000000000000000000000000000000000000000000000000000000000000000000000000000000000000000000000000000000000000000000001111111111100000000000011111111111111111000000000000000000000000001111111111111110000000000000000000000000000000000000000000000000000000000000000000000000000000000000000000000000000000000000000000000000000000000000000000000000000000000000000000000000000000000000000000000000000000000000000000001111111111111111111

>DM_train163

ASYFIGVDVGTGSARAGVFDLQGRMVGQASREITMFKPKADFVEQSSENIWQAVCNAVRDAVNQADINPIQVKGLGFDATCSLVVLDKEGNPLTVSPSGRNEQNVIVWMDHRAITQAERINATKHPVLEFVGGVISPEMQTPKLLWLKQHMPNTWSNVGHLFDLPDFLTWRATKDETRSLCSTVCKWTYLGHEDRWDPSYFKLVGLADLLDNNAAKIGATVKPMGAPLGHGLSQRAASEMGLIPGTAVSVSIIDAHAGTIGILGASGVTGENANFDRRIALIGGTSTAHMAMSRSAHFISGIWGPYYSAILPEYWLNEGGQSATGALIDHIIQSHPCYPALLEQAKNKGETIYEALNYILRQMAGEPENIAFLTNDIHMLPYFHGNRSPRANPNLTGIITGLKLSTTPEDMALRYLATIQALALGTRHIIETMNQNGYNIDTMMASGGGTKNPIFVQEHANATGCAMLLPEESEAMLLGSAMMGTVAAGVFESLPEAMAAMSRIGKTVTPQTNKIKAYYDRKYRVFHQMYHDHMRYQALMQEG

000000000000000000000000000000000000000000000000000000000000000000000000000000000000000000000000000000000000000000000000000000000000000000000000000000000000000000000000000000000000000000000000000000000000000000000000000000000000000000000000000000000000000000000000000000000000000000000000000000000000000000000000000000000000000000000000000000000000000000000000000000000000000000000000000000000000000000000000000000000000000000000000000000000000000000000000000000000000000000000000000000000000000000000000000000000000000000000000000000000000011

>DM_train164

MASWSHPQFEKGAETAVPNSSSVPGDPSSMRKKNVLLIVVDQWRADFVPHVLRADGKIDFLKTPNLDRLCREGVTFRNHVTTCVPAGPARASLLTGLYLMNHRAVQNTVPLDQRHLNLGKALRGVGYDPALIGYTTTVPDPRTTSPNDPRFRVLGDLMDGFHPVGAFEPNMEGYFGWVAQNGFDLPEHRPDIWLPEGEDAVAGATDRPSRIPKEFSDSTFFTERALTYLKGRDGKPFFLHLGYYRPHPPFVASAPYHAMYRPEDMPAPIRAANPDIEAAQHPLMKFYVDSIRRGSFFQGAEGSGATLDEAELRQMRATYCGLITEVDDCLGRVFSYLDETGQWDDTLIIFTSDHGEQLGDHHLLGKIGYNDPSFRIPLVIKDAGENARAGAIESGFTESIDVMPTILDWLGGKIPHACDGLSLLPFLSEGRPQDWRTELHYEYDFRDVYYSEPQSFLGLGMNDCSLCVIQDERYKYVHFAALPPLFFDLRHDPNEFTNLADDPAYAALVRDYAQKALSWRLKHADRTLTHYRSGPEGLSERSH

111111111111111111111111111111000000000000000000000000000000000000000000000000000000000000000000000000000000000000000000000000000000000000000000000000000000000000000000000000000000000000000000000000000000000000000000000000000000000000000000000000000000000000000000000000000000000000000000000000000000000000000000000000000000000000000000000000000000000000000000000000000000000000000000000000000000000000000000000000000000000000000000000000000000000000000000000000000000000000000000000000000000000000000000000000000000000000000000000000000000000

>DM_train165

MAGQTTVDSRRQPPEEVDVLVVGAGFSGLYALYRLRELGRSVHVIETAGDVGGVWYWNRYPGARCDIESIEYCYSFSEEVLQEWNWTERYASQPEILRYINFVADKFDLRSGITFHTTVTAAAFDEATNTWTVDTNHGDRIRARYLIMASGQLSVPQLPNFPGLKDFAGNLYHTGNWPHEPVDFSGQRVGVIGTGSSGIQVSPQIAKQAAELFVFQRTPHFAVPARNAPLDPEFLADLKKRYAEFREESRNTPGGTHRYQGPKSALEVSDEELVETLERYWQEGGPDILAAYRDILRDRDANERVAEFIRNKIRNTVRDPEVAERLVPKGYPFGTKRLILEIDYYEMFNRDNVHLVDTLSAPIETITPRGVRTSEREYELDSLVLATGFDALTGALFKIDIRGVGNVALKEKWAAGPRTYLGLSTAGFPNLFFIAGPGSPSALSNMLVSIEQHVEWVTDHIAYMFKNGLTRSEAVLEKEDEWVEHVNEIADETLYPMTASWYTGANVPGKPRVFMLYVGGFHRYRQICDEVAAKGYEGFVLT

11111111100000000000000000000000000000000000000000000000000000000000000000000000000000000000000000000000000000000000000000000000000000000000000000000000000000000000000000000000000000000000000000000000000000000000000000000000000000000000000000000000000000000000000000000000000000000000000000000000000000000000000000000000000000000000000000000000000000000000000000000000000000000000000000000000000000000000000000000000000000000000000000000000000000000000000000000000000000000000000000000000000000000000000000000000000000000000000000000000000000

>DM_train166

ALEEAPWPPPEGAFVGFVLSRKEPMWADLLALAAARGGRVHRAPEPYKALRDLKEARGLLAKDLSVLALREGLGLPPGDDPMLLAYLLDPSNTTPEGVARRYGGEWTEEAGERAALSERLFANLWGRLEGEERLLWLYREVERPLSAVLAHMEATGVRLDVAYLRALSLEVAEEIARLEAEVFRLAGHPFNLNSRDQLERVLFDELGLPAIGKTEKTGKRSTSAAVLEALREAHPIVEKILQYRELTKLKSTYIDPLPDLIHPRTGRLHTRFNQTATATGRLSSSDPNLQNIPVRTPLGQRIRRAFIAEEGWLLVALDYSQIELRVLAHLSGDENLIRVFQEGRDIHTETASWMFGVPREAVDPLMRRAAKTINFGVLYGMSAHRLSQELAIPYEEAQAFIERYFQSFPKVRAWIEKTLEEGRRRGYVETLFGRRRYVPDLEARVKSVREAAERMAFNMPVQGTAADLMKLAMVKLFPRLEEMGARMLLQVHDELVLEAPKERAEAVARLAKEVMEGVYPLAVPLEVEVGIGEDWLSAKE

000000000000000000000000000000000000000000000000000000000000000000000000000000000000000000000000000000000000000000000000000000000000000000000000000000000000000000000000000000000000000000000000000000000000000011111111111111100000000000000000000000000000000000000000000000000000000000000000000000000000000000000000000000000000000000000000000000000000000000000000000000000000000000000000000000000000000000000000000000000000000000000000000000000000000000000000000000000000000000000000000000000000000000000000000000000000000000000000000000000000

>DM_train168

MNKPIKNIVIVGGGTAGWMAASYLVRALQQQANITLIESAAIPRIGVGEATIPSLQKVFFDFLGIPEREWMPQVNGAFKAAIKFVNWRKSPDPSRDDHFYHLFGNVPNCDGVPLTHYWLRKREQGFQQPMEYACYPQPGALDGKLAPCLSDGTRQMSHAWHFDAHLVADFLKRWAVERGVNRVVDEVVDVRLNNRGYISNLLTKEGRTLEADLFIDCSGMRGLLINQALKEPFIDMSDYLLCDSAVASAVPNDDARDGVEPYTSSIAMNSGWTWKIPMLGRFGSGYVFSSHFTSRDQATADFLKLWGLSDNQPLNQIKFRVGRNKRAWVNNCVSIGLSSCFLEPLESTGIYFIYAALYQLVKHFPDTSFDPRLSDAFNAEIVHMFDDCRDFVQAHYFTTSRDDTPFWLANRHDLRLSDAIKEKVQRYKAGLPLTTTSFDDSTYYETFDYEFKNFWLNGNYYCIFAGLGMLPDRSLPLLQHRPESIEKAEAMFASIRREAERLRTSLPTNYDYLRSLRDGDAGLSRGQRGPKLAAQESL

1000000000000000000000000000000000000000000000000000000000000000000000000000000000000000000000000000000000000000000000000000000000000000000000000000000000000000000000000000000000000000000000000000000000000000000000000000000000000000000000000000000000000000000000000000000000000000000000000000000000000000000000000000000000000000000000000000000000000000000000000000000000000000000000000000000000000000000000000000000000000000000000000000000000000000000000000000000000000000000000000000000000000000000000000000000000000011111111111111111111

>DM_train169

MSSQKVFGITGPVSTVGATAAENKLNDSLIQELKKEGSFETEQETANRVQVLKILQELAQRFVYEVSKKKNMSDGMARDAGGKIFTYGSYRLGVHGPGSDIDTLVVVPKHVTREDFFTVFDSLLRERKELDEIAPVPDAFVPIIKIKFSGISIDLICARLDQPQVPLSLTLSDKNLLRNLDEKDLRALNGTRVTDEILELVPKPNVFRIALRAIKLWAQRRAVYANIFGFPGGVAWAMLVARICQLYPNACSAVILNRFFIILSEWNWPQPVILKPIEDGPLQVRVWNPKIYAQDRSHRMPVITPAYPSMCATHNITESTKKVILQEFVRGVQITNDIFSNKKSWANLFEKNDFFFRYKFYLEITAYTRGSDEQHLKWSGLVESKVRLLVMKLEVLAGIKIAHPFTKPFESSYCCPTEDDYEMIQDKYGSHKTETALNALKLVTDENKEEESIKDAPKAYLSTMYIGLDFNIENKKEKVDIHIPCTEFVNLCRSFNEDYGDHKVFNLALRFVKGYDLPDEVFDENEKRPSKKSKRKN

000000000000000000000000000000000000000000000000000000000000000000000000000000000000000000000000000000000000000000000000000000000000000000000000000000000000000000000000000000000000000000000000000000000000000000000000000000000000000000000000000000000000000000000000000000000000000000000000000000000000000000000000000000000000000000000000000000000000000000000000000000000000000000000000000000000000000000000000000000000000000000011111111111100000000000000000000000000000000000000000000000000000000000000000000000000000000000011111111111111

>DM_train170

MPSRVPKSIFYNQVGYLISGDKRFWIQAHEPQPFALRTPEGQAVFAGMTKPVGGNWYVGDFTALRVPGTYTLTVGTLEARVVIHRRAYRDVLEAMLRFFDYQLCGVVLPEDEAGPWAHGACHTSDAKVFGTERALACPGGWHDAGDYGKYTVPAAKAVADLLLAHEYFPAALAHVRPMRSVHRAPHLPPALEVAREEIAWLLTMQDPATGGVYHKVTTPSFPPLDTRPEDDDAPLVLSPISYAATATFCAAMAHAALVYRPFDPALSSCCADAARRAYAWLGAHEMQPFHNPDGILTGEYGDAELRDELLWASCALLRMTGDSAWARVCEPLLDLDLPWELGWADVALYGVMDYLRTPRAAVSDDVRNKVKSRLLRELDALAAMAESHPFGIPMRDDDFIWGSNMVLLNRAMAFLLAEGVGVLHPAAHTVAQRAADYLFGANPLGQCYVTGFGQRPVRHPHHRPSVADDVDHPVPGMVVGGPNRHLQDEIARAQLAGRPAMEAYIDHQDSYSTNEVAVYWNSPAVFVIAALLEARGR

111111000000000000000000000000000000000000000000000000000000000000000000000000000000000000000000000000000000000000000000000000000000000000000000000000000000000000000000000000000000000000000000000000000000000000000000000000000000000000000000000000000000000000000000000000000000000000000000000000000000000000000000000000000000000000000000000000000000000000000000000000000000000000000000000000000000000000000000000000000000000000000000000000000000000000000000000000000000000000000000000000000000000000000000000000000000000000000000000000000

>DM_train171

GADDVVDSSKSFVMENFSSYHGTKPGYVDSIQKGIQKPKSGTQGNYDDDWKGFYSTDNKYDAAGYSVDNENPLSGKAGGVVKVTYPGLTKVLALKVDNAETIKKELGLSLTEPLMEQVGTEEFIKRFGDGASRVVLSLPFAEGSSSVEYINNWEQAKALSVELEINFETRGKRGQDAMYEYMAQACAGNRVRRSVGSSLSCINLDWDVIRDKTKTKIESLKEHGPIKNKMSESPNKTVSEEKAKQYLEEFHQTALEHPELSELKTVTGTNPVFAGANYAAWAVNVAQVIDSETADNLEKTTAALSILPGIGSVMGIADGAVHHNTEEIVAQSIALSSLMVAQAIPLVGELVDIGFAAYNFVESIINLFQVVHNSYNRPAYSPGHKTQPFLHDGYAVSWNTVEDSIIRTGFQGESGHDIKITAENTPLPIAGVLLPTIPGKLDVNKSKTHISVNGRKIRMRCRAIDGDVTFCRPKSPVYVGNGVHANLHVAFHRSSSEKIHSNEISSDSIGVLGYQKTVDHTKVNSKLSLFFEIKS

0000000000000000000000000000000000000111111111100000000000000000000000000000000000000000000000000000000000000000000000000000000000000000000000000000000000000000000000000000000000000000000111111111111000000000000000000000000000000000000000000000000000000000000000000000000000000000000000000000000000000000000000000000000000000000000000000000000000000000000000000000000000000000000000000000000000000000000000000000000000000000000000000000000000000000000000000000000000000000000000000000000000000000000000000000000000000000000000000000000

>DM_train172

EETPVTPQPPDILLGPLFNDVQNAKLFPDQKTFADAVPNSDPLMILADYRMQQNQSGFDLRHFVNVNFTLPKEGEKYVPPEGQSLREHIDGLWPVLTRSTENTEKWDSLLPLPEPYVVPGGRFREVYYWDSYFTMLGLAESGHWDKVADMVANFAHEIDTYGHIPNGNRSYYLSRSQPPFFALMVELLAQHEGDAALKQYLPQMQKEYAYWMDGVENLQAGQQEKRVVKLQDGTLLNRYWDDRDTPRPESWVEDIATAKSNPNRPATEIYRDLRSAAASGWDFSSRWMDNPQQLNTLRTTSIVPVDLNSLMFKMEKILARASKAAGDNAMANQYETLANARQKGIEKYLWNDQQGWYADYDLKSHKVRNQLTAAALFPLYVNAAAKDRANKMATATKTHLLQPGGLNTTSVKSGQQWDAPNGWAPLQWVATEGLQNYGQKEVAMDISWHFLTNVQHTYDREKKLVEKYDVSTTGTGGGGGEYPLQDGFGWTNGVTLKMLDLICPKEQPCDNVPATRPTVKSATTQPSTKEAQPTP

1111110000000000000000000000000000000000000000000000000000000000000000001111000000000000000000000000000000000000000000000000000000000000000000000000000000000000000000000000000000000000000000000000000000000000000000000000000000000000000000000000000000000000000000000000000000000000000000000000000000000000000000000000000000000000000000000000000000000000000000000000000000000000000000000000000000000000000000000000000000000000000000000000000000000000000000000000000000000000000000000000000000000000000000000000000000000111111111111111111

>DM_train174

MAQLGKLLKEQKYDRQLRLWGDHGQEALESAHVCLINATATGTEILKNLVLPGIGSFTIIDGNQVSGEDAGNNFFLQRSSIGKNRAEAAMEFLQELNSDVSGSFVEESPENLLDNDPSFFCRFTVVVATQLPESTSLRLADVLWNSQIPLLICRTYGLVGYMRIIIKEHPVIESHPDNALEDLRLDKPFPELREHFQSYDLDHMEKKDHSHTPWIVIIAKYLAQWYSETNGRIPKTYKEKEDFRDLIRQGILKNENGAPEDEENFEEAIKNVNTALNTTQIPSSIEDIFNDDRCINITKQTPSFWILARALKEFVAKEGQGNLPVRGTIPDMIADSGKYIKLQNVYREKAKKDAAAVGNHVAKLLQSIGQAPESISEKELKLLCSNSAFLRVVRCRSLAEEYGLDTINKDEIISSMDNPDNEIVLYLMLRAVDRFHKQQGRYPGVSNYQVEEDIGKLKSCLTGFLQEYGLSVMVKDDYVHEFCRYGAAEPHTIAAFLGGAAAQEVIKIITKQFVIFNNTYIYSGMSQTSATFQL

111111110000000000000000000000000000000000000000000000000000000000000000000000000000000000000000000000000000000000000000000000000000000000000000000000000000000000000000000000000000000000000000000000000000000000000000000000000000000000000000000000000000000000000000000000000000000000000000000000000000000000000000000000000000000000000000000000000000000000000000000000000000000000000000000000000000000000000000000000000000000000000000000000000000000000000000000000000000000000000000000000000000000000000000000000000000000000000000000000

>DM_train175

SHMQSRELKTVSADCKKEAIEKCAQWVVRDCRPFSAVSGSGFIDMIKFFIKVKAEYGEHVNVEELLPSPITLSRKVTSDAKEKKALIGREIKSAVEKDGASATIDLWTDNYIKRNFLGVTLHYHENNELRDLILGLKSLDFERSTAENIYKKLKAIFSQFNVEDLSSIKFVTDRGANVVKSLANNIRINCSSHLLSNVLENSFEETPELNMPILACKNIVKYFKKANLQHRLRSSLKSECPTRWNSTYTMLRSILDNWESVIQILSEAGETQRIVHINKSIIQTMVNILDGFERIFKELQTCSSPSLCFVVPSILKVKEICSPDVGDVADIAKLKVNIIKNVRIIWEENLSIWHYTAFFFYPPALHMQQEKVAQIKEFCLSKMEDLELINRMSSFNELSATQLNQSDSNSHNSIDLTSHSKDISTTSFFFPQLTQNNSREPPVCPSDEFEFYRKEIVILSEDFKVMEWWNLNSKKYPKLSKLALSLLSIPASSAASERTFSLAGNIITEKRNRIGQQTVDSLLFLNSFYKNFCK

000000000000000000000000000000000000000000000000000000000000000000000000000000000000000000000000000000000000000000000000000000000000000000000000000000000000000000000000000000000000000000000000000000000000000000000000000000000000000000000000000000000000000000000000000000000000000000000000000000000000000000000000000000000000000000000000000000000000000000000000000000000000000000000000000000000000000000000111111111111111100000000000000000000000000000000000000000000000000000000000000000000000000000000000000000000000000000000000000000

>DM_train177

MSSQVEHPAGGYKKLFETVEELSSPLTAHVTGRIPLWLTGSLLRCGPGLFEVGSEPFYHLFDGQALLHKFDFKEGHVTYHRRFIRTDAYVRAMTEKRIVITEFGTCAFPDPCKNIFSRFFSYFRGVEVTDNALVNIYPVGEDYYACTETNFITKVNPETLETIKQVDLCNYVSVNGATAHPHIENDGTVYNIGNCFGKNFSIAYNIVKIPPLQADKEDPISKSEIVVQFPCSDRFKPSYVHSFGLTPNYIVFVETPVKINLFKFLSSWSLWGANYMDCFESNETMGVWLHIADKKRKKYINNKYRTSPFNLFHHINTYEDHEFLIVDLCCWKGFEFVYNYLYLANLRENWEEVKKNARKAPQPEVRRYVLPLNIDKADTGKNLVTLPNTTATAILCSDETIWLEPEVLFSGPRQAFEFPQINYQKYGGKPYTYAYGLGLNHFVPDRLCKLNVKTKETWVWQEPDSYPSEPIFVSHPDALEEDDGVVLSVVVSPGAGQKPAYLLILNAKDLSEVARAEVEINIPVTFHGLFKKS

11000000000000000000000000000000000000000000000000000000000000000000000000000000000000000000000000000000000111111111111111111100000000000000000000000000000000000000000000000000000000000000000000000111000000000000000000000000000000000000000000000000000000000000111111111111000000000000000000000000000000000000000000000000000000000000000000000000000000000000000000000000000000000000000000000000000000000000000000000000000000000000000000000000000000000000000000000000000000000000000000000000000000000000000000000000000000000000000000000

>DM_train179

MKPVKPPRINGRVPVLSAQEAVNYIPDEATLCVLGAGGGILEATTLITALADKYKQTQTPRNLSIISPTGLGDRADRGISPLAQEGLVKWALCGHWGQSPRISDLAEQNKIIAYNYPQGVLTQTLRAAAAHQPGIISDIGIGTFVDPRQQGGKLNEVTKEDLIKLVEFDNKEYLYYKAIAPDIAFIRATTCDSEGYATFEDEVMYLDALVIAQAVHNNGGIVMMQVQKMVKKATLHPKSVRIPGYLVDIVVVDPDQSQLYGGAPVNRFISGDFTLDDSTKLSLPLNQRKLVARRALFEMRKGAVGNVGVGIADGIGLVAREEGCADDFILTVETGPIGGITSQGIAFGANVNTRAILDMTSQFDFYHGGGLDVCYLSFAEVDQHGNVGVHKFNGKIMGTGGFIDISATSKKIIFCGTLTAGSLKTEIADGKLNIVQEGRVKKFIRELPEITFSGKIALERGLDVRYITERAVFTLKEDGLHLIEIAPGVDLQKDILDKMDFTPVISPELKLMDERLFIDAAMGFVLPEAAH

111000000000000000000000000000000000000000000000000000000000000000000000000000000000000000000000000000000000000000000000000000000000000000000000000000000000000000000000000000000000000000000000000000000000000000000000000000000000000000000000000000000000000000000000000000000000111111000000000000000000000000000000000000000000000000000000000000111110000000000000000000000000000000000000000000000000000000000000000000000000000000000000000000000000000000000000000000000000000000000000000000000000000000000000000000000000000000000000011

>DM_train181

MDNGTDSSTSKFVPEYRRTNFKNKGRFSADELRRRRDTQQVELRKAKRDEALAKRRNFIPPTDGADSDEEDESSVSADQQFYSQLQQELPQMTQQLNSDDMQEQLSATVKFRQILSREHRPPIDVVIQAGVVPRLVEFMRENQPEMLQLEAAWALTNIASGTSAQTKVVVDADAVPLFIQLLYTGSVEVKEQAIWALGNVAGDSTDYRDYVLQCNAMEPILGLFNSNKPSLIRTATWTLSNLCRGKKPQPDWSVVSQALPTLAKLIYSMDTETLVDACWAISYLSDGPQEAIQAVIDVRIPKRLVELLSHESTLVQTPALRAVGNIVTGNDLQTQVVINAGVLPALRLLLSSPKENIKKEACWTISNITAGNTEQIQAVIDANLIPPLVKLLEVAEYKTKKEACWAISNASSGGLQRPDIIRYLVSQGCIKPLCDLLEIADNRIIEVTLDALENILKMGEADKEARGLNINENADFIEKAGGMEKIFNCQQNENDKIYEKAYKIIETYFGEEEDAVDETMAPQNAGNTFG

11111111111000000001111111111100000000000000000000000000001111111111111111111111111111000000000000000000000000000000000000000000000000000000000000000000000000000000000000000000000000000000000000000000000000000000000000000000000000000000000000000000000000000000000000000000000000000000000000000000000000000000000000000000000000000000000000000000000000000000000000000000000000000000000000000000000000000000000000000000000000000000000000000000000000000000000000000000000000000000000000000000000000000000000000000000001111111111111111

>DM_train183

STQTSRVTLVGEMLPAYNEILTPEALSFLKELHENFNERRIELLQKRMKKQQKIDAGEFPKFLEETKRIREADWTIAKLPKDLEDRRVEITGPVDRKMVINALNSGAHLFMADFEDSNSPTWENAIEGQINLRDAVKGTISHKNENGKEYRLNSKTAVLIVRPRGWHLEEKHMQVDGKNMSGSLVDFGLYFFHNAKALLEKGSGPYFYLPKMESYLEARLWNDVFVFAQKYIGIPNGTIKATVLLETIHASFEMDEILYELKDHSAGLNCGRWDYIFSFLKAFRNHNEFLLPDRAQVTMTAPFMRAYSLKVIQTCHRRNAPAIGGMAAQIPIKNNPEANEAAFEKVRADKEREALDGHDGTWVAHPGLVPVAMEVFNHIMKTPNQIFRKREEIHVTEKDLLEVPVGTITEEGLRMNISVGIQYIASWLSGRGAAPIYNLMEDAATAEISRAQVWQWIRHEGGKLNDGRNITLELMEELKEEELAKIEREIGKEAFKKGRFQEATTLFTNLVRNDEFVPFLTLPGYEIL

111110000000011000000000000000000000000000000000000000000000000000000000000000000000000000000000000000000000000000000000000000000000000000000000000000000000000000000000000000000000000000000000000000000000000000000000000000000000000000000000000000000000000000000000000000000000000000000000000000000000000000000000000000000000111111111111111111100000000000000000000000000000000000000000000000000000000000000000000000000000000000000000000000000000000000000000000000000000000000000000000000000000001000000000000000000000000000000000

>DM_train184

GCDLERYPLTDLSEETFWNSESNAELALTSLYRGSLTDGVEYNPSDWWSYHGMIMMEHLSDNAFDRRGENNPFFKISSGNLTADNAFIKRYWETSYKRIGYCNRFLVGIQNSSESEKKTRMIAEARFLRATQYFYLASYFKNVPLVENVLTGEEANNVTKTSQADILKWCVTEFTAAAADLPRFSAIPAGEAGRACKQAALAFLGRTCMLQKDWKSGAKAFHDIMELGDNAINANYQELFYPSTGTSNKENIFYIQYLENYLGTGLPQHALSAKDGGWSLVNPAADLYESYEFKDGTPFSYDDPRYDPSNLGKDRDPRLDYTIYYNGAIFMGTEYKMSPDYSAAKKEKLDYTSEASRTGFMMRKYFEESTPINDVQSANGLTPVIRYAEVLLGYLECLVEDNQTITQGILDETINAVRGRASVNMPPVTEVTPAKLREIVRHERRIELAMEGIRYWDIMRWGIAHEVLSQKIWGAPYPGSTQYATTTKEVDPTGNYRWYVGKRAFRNPTDYTWPIPQSEQNINPNLRD

111111111111110000000000000000000000000000000000000000000000000000000000000000000000000000000000000000000000000000000000000000000000000000000000000000000000000000000000000000000000000000000000000000000000000000000000000000000000000000000000000000000000000000000000000000000000000000000000000000000000000000000000000000000000000000000000000000000000000000000000000000000000000000000000000000000000000000000000000000000000000000000000000000000000000000000000000000000000000000000000000000000000000000000000000000000000000000000001

>DM_train185

MADSRDPASDQMQHWKEQRAAQKADVLTTGAGNPVGDKLNVITVGPRGPLLVQDVVFTDEMAHFDRERIPERVVHAKGAGAFGYFEVTHDITKYSKAKVFEHIGKKTPIAVRFSTVAGESGSADTVRDPRGFAVKFYTEDGNWDLVGNNTPIFFIRDPILFPSFIHSQKRNPQTHLKDPDMVWDFWSLRPESLHQVSFLFSDRGIPDGHRHMNGYGSHTFKLVNANGEAVYCKFHYKTDQGIKNLSVEDAARLSQEDPDYGIRDLFNAIATGKYPSWTFYIQVMTFNQAETFPFNPFDLTKVWPHKDYPLIPVGKLVLNRNPVNYFAEVEQIAFDPSNMPPGIEASPDKMLQGRLFAYPDTHRHRLGPNYLHIPVNCPYRARVANYQRDGPMCMQDNQGGAPNYYPNSFGAPEQQPSALEHSIQYSGEVRRFNTANDDNVTQVRAFYVNVLNEEQRKRLCENIAGHLKDAQIFIQKKAVKNFTEVHPDYGSHIQALLDKYNAEKPKNAIHTFVQSGSHLAAREKANL

11111111111111111111111000000000000000000000000000000000000000000000000000000000000000000000000000000000000000000000000000000000000000000000000000000000000000000000000000000000000000000000000000000000000000000000000000000000000000000000000000000000000000000000000000000000000000000000000000000000000000000000000000000000000000000000000000000000000000000000000000000000000000000000000000000000000000000000000000000000000000000000000000000000000000000000000000000000000000000000000000000000000000000000001111111111111111111111111

>DM_train187

SQIAVRTFHDIRAALLARRELALLDVREEDPFAQAHPLFAANLPLSRLELEIHARVPRRDTPITVYDDGEGLAPVAAQRLHDLGYSDVALLDGGLSGWRNAGGELFRDVNVPSKAFGELVEAERHTPSLAAEEVQALLDARAEAVILDARRFDEYQTMSIPGGISVPGAELVLRVAELAPDPRTRVIVNCAGRTRSIIGTQSLLNAGIPNPVAALRNGTIGWTLAGQQLEHGQTRRFGAISQDTRKAAAQRARAVADRAGVERLDLAGLAQWQDEHDRTTYLLDVRTPEEYEAGHLPGSRSTPGGQLVQETDHVASVRGARLVLVDDDGVRANMSASWLAQMGWQVAVLDGLSEADFSERGAWSAPLPRQPRADTIDPTTLADWLGEPGTRVLDFTASANYAKRHIPGAAWVLRSQLKQALERLGTAERYVLTCGSSLLARFAVAEVQALSGKPVFLLDGGTSAWVAAGLPTEDGESLLASPRIDRYRRPYEGTDNPREAMQGYLDWEFGLVEQLGRDGTHGFFVI

1100000000000000000000000000000000000000000000000000000000000000000000000000000000000000000000000000000000000000000000000000000000000000000000000000000000000000000000000000000000000000000000000000000000000000000000000000000000000000000000000000000000000000000000000000000000000000000000000000000000000000000000000000000000000000000000000000000000000000000000000000000000000000000000000000000000000000000000000000000000000000000000000000000000000000000000000000000000000000000000000000000000000000000000000000000000000000000000

>DM_train188

MVAAPPAVGAAMPSLDFDTSVFNKEKVSLAGHEEYIVRGGRNLFPLLPEAFKGIKQIGVIGWGSQGPAQAQNLRDSLAEAKSDIVVKIGLRKGSKSFDEARAAGFTEESGTLGDIWETVSGSDLVLLLISDAAQADNYEKIFSHMKPNSILGLSHGFLLGHLQSAGLDFPKNISVIAVCPKGMGPSVRRLYVQGKEINGAGINSSFAVHQDVDGRATDVALGWSVALGSPFTFATTLEQEYKSDIFGERGILLGAVHGIVEALFRRYTEQGMDEEMAYKNTVEGITGIISKTISKKGMLEVYNSLTEEGKKEFNKAYSASFYPCMDILYECYEDVASGSEIRSVVLAGRRFYEKEGLPAFPMGNIDQTRMWKVGEKVRSTRPENDLGPLHPFTAGVYVALMMAQIEVLRKKGHSYSEIINESVIESVDSLNPFMHARGVAFMVDNCSTTARLGSRKWAPRFDYILTQQAFVTVDKDAPINQDLISNFMSDPVHGAIEVCAELRPTVDISVPANADFVRPELRQSS

111111111111110000000000000000000000000000000000000000000000000000000000000000000000000000000000000000000000000000000000000000000000000000000000000000000000000000000000000000000000000000000000000000000000000000000000000000000000000000000000000000000000000000000000000000000000000000000000000000000000000000000000000000000000000000000000000000000000000000000000000000000000000000000000000000000000000000000000000000000000000000000000000000000000000000000000000000000000000000000000000000000000000000000000111111111111111111111

>DM_train189

AVPAVGEDFPIDYADWLPKRDPNDRRRAGILLHPTSFPGPYGIGDLGPQAFKFLDWLHLAGCSLWQVLPLVPPGKRGNEDGSPYSGQDANCGNTLLISLEELVDDGLLKMEELPEPLPTDRVNYSTISEIKDPLITKAAKRLLSSEGELKDQLENFRRDPNISSWLEDAAYFAAIDNSVNTISWYDWPEPLKNRHLAALEEVYQSEKDFIDIFIAQQFLFQRQWKKVRDYARSKGISIMGDMPIYVGYHSADVWANKKQFLLNRKGFPLIVSGVPPDAFSETGQLWGSPLYDWKAMEKDGFSWWVRRIQRATDLFDEFRIDHFRGFAGFWAVPSEEKIAILGRWKVGPGKPLFDAILQAVGKINIIAEDLGVITEDVVQLRKSIEAPGMAVLQFAFGSDAENPHLPHNHEQNQVVYTGTHDNDTIRGWWDTLPQEEKSNVLKYLSNIEEEEISRGLIEGAVSSVARIAIIPMQDVLGLGSDSRMNIPATQFGNWSWRIPSSTSFDNLDAEAKKLRDILATYGRL

10000000000000000000000000000000000000000000000000000000000000000000000000000000000000000000000000000000000000000000000000000000000000000000000000000000000000000000000000000000000000000000000000000000000000000000000000000000000000000000000000000000000000000000000000000000000000000000000000000000000000000000000000000000000000000000000000000000000000000000000000000000000000000000000000000000000000000000000000000000000000000000000000000000000000000000000000000000000000000000000000000000000000000000000000000000000000000000

>DM_train191

MASEVPQVVSLDPTSIPIEYNTPIHDIKVQVYDIKGGCNVEEGLTIFLVNNPGKENGPVKISSKVNDKQVSEFLKDENMEKFNVKLGTSKHFYMFNDNKNSVAVGYVGCGSVADLSEADMKRVVLSLVTMLHDNKLSKLTVVFEINVDKNLFRFFLETLFYEYMTDERFKSTDKNVNMEYIKHLGVYINNADTYKEEVEKARVYYFGTYYASQLIAAPSNYCNPVSLSNAAVELAQKLNLEYKILGVKELEELKMGAYLSVGKGSMYPNKFIHLTYKSKGDVKKKIALVGKGITFDSGGYNLKAAPGSMIDLMKFDMSGCAAVLGCAYCVGTLKPENVEIHFLSAVCENMVSKNSYRPGDIITASNGKTIEVGNTDAEGRLTLADALVYAEKLGVDYIVDIATLTGAMLYSLGTSYAGVFGNNEELINKILQSSKTSNEPVWWLPIINEYRATLNSKYADINQISSSVKASSIVASLFLKEFVQNTAWAHIDIAGVSWNFKARKPKGFGVRLLTEFVLNDAL

110000000000000000000000000000000000000000000000000010000000000000000000000000000000000000000000000000000000000000000000000000000000000000000000000000000000000000000000000111111110000000000000000000000000000000000000000000000000000000000000000000000000000000000000000000000000000000000000000000000000000000000000000000000000000000000000000000000000000000000000000000000000000000000000000000000000000000000000000000000000000000000000000000000000000000000000000000000000000000000000000000000000000000000000000000000000000011

>DM_train193

MPSFASLKSLVVLSLTSLSLAATVALDLHILNANLDPDGTGARSAVTAEGTTIAPLITGNIDDRFQINVIDQLTDANMRRATSIHWHGFFQAGTTEMDGPAFVNQCPIIPNESFVYDFVVPGQAGTYWYHSHLSTQYCDGLRGAFVVYDPNDPHLSLYDVDDASTVITIADWYHSLSTVLFPNPNKAPPAPDTTLINGLGRNSANPSAGQLAVVSVQSGKRYRFRIVSTSCFPNYAFSIDGHRMTVIEVDGVSHQPLTVDSLTIFAGQRYSVVVEANQAVGNYWIRANPSNGRNGFTGGINSAIFRYQGAAVAEPTTSQNSGTALNEANLIPLINPGAPGNPVPGGADINLNLRIGRNATTADFTINGAPFIPPTVPVLLQILSGVTNPNDLLPGGAVISLPANQVIEISIPGGGNHPFHLHGHNFDVVRTPGSSVYNYVNPVRRDVVSIGGGGDNVTFRFVTDNPGPWFLHCHIDWHLEAGLAVVFAEDIPNIPIANAISPAWDDLCPKYNANNPDSGLA

11111111111111111111100000000000000000000000000000000000000000000000000000000000000000000000000000000000000000000000000000000000000000000000000000000000000000000000000000000000001111111000000000000000000000000000000000000000000000000000000000000000000000000000000000000000000000000000000000000000000000000000000000000000000000000000000000000000000000000000000000000000000000000000000000000000000000000000000000000000000000000000000000000000000000000000000000000000000000000000000000000000000000000000000000000000000111111

>DM_train194

MSASSSSALPPLVPALYRWKSTGSSGRQVQRRCVGAEAIVGLEEKNRRALYDLYIATSLRNIAPASTLLTLQNLKEMFELALLDARFEHPECACTVSWDDEVPAIITYESPESNESARDWARGCIHVQPTAKSALDLWSEMEEGRAAANDNTPSKSIELFLLSDVSTDSTPIPQDATVEILFHSNHLFWDGIGCRKFVGDLFRLVGSYIGRSDSREMKKIQWGQEIKNLSPPVVDSLKLDINTLGSEFDDKCTEYTSALVANYKSRGMKFQPGLALPRCVIHKLSADESIDIVKAVKTRLGPGFTISHLTQAAIVLALLDHLKPNDLSDDEVFISPTSVDGRRWLREDIASNFYAMCQTAAVVRIENLKSITVSHKDEKELQVRALESACRNIKKSYRQWLENPFLQALGLRVHNFEASYLHAKPIPFEGEANPLFISDGINERFIPHEIKQTATGENVLSVESIDFVVNQSLPYLAIRLDSWRDASTLNIIYNDANYTEAEVQKYLQSIVEFMLAFRL

111111110000000000000000000000000000000000000000000000000000000000000000000000000000000000000000000000000000000000000000000000000000000000000000000001000000000000000000000000000000000000000000000000000000000000000000000000000000000000000000000000000000000000000000000000000000000000000000000000000000000000000000000000000001110000000000000000000000000000000000000000000000000000000000000000000000000000000000000000000000000000000000000000000000000000000000000000000000000000000000000000000000000000000000000000000000000

>DM_train195

MEVKREHWATRLGLILAMAGNAVGLGNFLRFPVQAAENGGGAFMIPYIIAFLLVGIPLMWIEWAMGRYGGAQGHGTTPAIFYLLWRNRFAKILGVFGLWIPLVVAIYYVYIESWTLGFAIKFLVGLVPEPPPNATDPDSILRPFKEFLYSYIGVPKGDEPILKPSLFAYIVFLITMFINVSILIRGISKGIERFAKIAMPTLFILAVFLVIRVFLLETPNGTAADGLNFLWTPDFEKLKDPGVWIAAVGQIFFTLSLGFGAIITYASYVRKDQDIVLSGLTAATLNEKAEVILGGSISIPAAVAFFGVANAVAIAKAGAFNLGFITLPAIFSQTAGGTFLGFLWFFLLFFAGLTSSIAIMQPMIAFLEDELKLSRKHAVLWTAAIVFFSAHLVMFLNKSLDEMDFWAGTIGVVFFGLTELIIFFWIFGADKAWEEINRGGIIKVPRIYYYVMRYITPAFLAVLLVVWAREYIPKIMEETHWTVWITRFYIIGLFLFLTFLVFLAERRRNHESAGTLVPR

111100000000000000000000000000000000000000000000000000000000000000000000000000000000000000000000000000000000000000000000000000000000110000000000000000000000000000000000000000000000000000000000000000000000000000000000000000000000000000000000000000000000000000000000000000000000000000000000000000000000000000000000000000000000000000000000000000000000000000000000000000000000000000000000000000000000000000000000000000000000000000000000000000000000000000000000000000000000000000000000000000000000000000000000000000000001111

>DM_train196

FNYDQPYRGQYHFSPQKNWMNDPNGLLYHNGTYHLFFQYNPGGIEWGNISWGHAISEDLTHWEEKPVALLARGFGSDVTEMYFSGSAVADVNNTSGFGKDGKTPLVAMYTSYYPVAQTLPSGQTVQEDQQSQSIAYSLDDGLTWTTYDAANPVIPNPPSPYEAEYQNFRDPFVFWHDESQKWVVVTSIAELHKLAIYTSDNLKDWKLVSEFGPYNAQGGVWECPGLVKLPLDSGNSTKWVITSGLNPGGPPGTVGSGTQYFVGEFDGTTFTPDADTVYPGNSTANWMDWGPDFYAAAGYNGLSLNDHVHIGWMNNWQYGANIPTYPWRSAMAIPRHMALKTIGSKATLVQQPQEAWSSISNKRPIYSRTFKTLSEGSTNTTTTGETFKVDLSFSAKSKASTFAIALRASANFTEQTLVGYDFAKQQIFLDRTHSGDVSFDETFASVYHGPLTPDSTGVVKLSIFVDRSSVEVFGGQGETTLTAQIFPSSDAVHARLASTGGTTEDVRADIYKIASTWN

00000000000000000000000000000000000000000000000000000000000000000000000000000000000000000000000000000000000000000000000000000000000000000000000000000000000000000000000000000000000000000000000000000000000000000000000000000000000000000000000000000000000000000000000000000000000000000000000000000000000000000000000000000000000000000000000000000000000000000000000000000000000000000000000000000000000000000000000000000000000000000000000000000000000000000000000000000000000000000000000000000000000000000000000000000000000001

>DM_train197

EPAKVVDIRIDTSAERKPISPYIYGSNQELDATVTAKRFGGNRTTGYNWENNFSNAGSDWLHYSDTYLLEDGGVPKGEWSTPASVVTTFHDKALSKNVPYTLITLQAAGYVSADGNGPVSQEETAPSSRWKEVKFEKGAPFSLTPDTEDDYVYMDEFVNYLVNKYGNASTPTGIKGYSIDNEPALWSHTHPRIHPDNVTAKELIEKSVALSKAVKKVDPYAEIFGPALYGFAAYETLQSAPDWGTEGEGYRWFIDYYLDKMKKASDEEGKRLLDVLDVHWYPEARGGGERICFGADPRNIETNKARLQAPRTLWDPTYIEDSWIGQWKKDFLPILPNLLDSIEKYYPGTKLAITEYDYGGGNHITGGIAQADVLGIFGKYGVYLATFWGDASNNYTEAGINLYTNYDGKGGKFGDTSVKCETSDIEVSSAYASIVGEDDSKLHIILLNKNYDQPTTFNFSIDSSKNYTIGNVWAFDRGSSNITQRTPIVNIKDNTFTYTVPALTACHIVLEAAEP

11000000000000000000000000000000000000000000000000000000000000000000000000000000000000000000000000000000000000000000000000000000000000000000000000000000000000000000000000000000000000000000000000000000000000000000000000000000000000000000000000000000000000000000000000000000000000000000000000000000000000000000000000000000000000000000000000000000000000000000000000000000000000000000000000000000000000000000000000000000000000000000000000000000000000000000000000000000000000000000000000000000000000000000000000000001111

>DM_train198

AEQVALSRTHVCGILREELFQGDAFHQSDTHIFIIMGASGDLAKKKIYPTIWWLFRDGLLPENTFIVGYARSRLTVADIRKQSEPFFKATPEEKLKLEDFFARNSYVAGQYDDAASYQRLNSHMNALHLGSQANRLFYLALPPTVYEAVTKNIHESCMSQIGWNRIIVEKPFGRDLQSSDRLSNHISSLFREDQIYRIDHYLGKEMVQNLMVLRFANRIFGPIWNRDNIACVILTFKEPFGTEGRGGYFDEFGIIRDVMQNHLLQMLCLVAMEKPASTNSDDVRDEKVKVLKCISEVQANNVVLGQYVGNPDGEGEATKGYLDDPTVPRGSTTATFAAVVLYVENERWDGVPFILRCGKALNERKAEVRLQFHDVAGDIFHQQCKRNELVIRVQPNEAVYTKMMTKKPGMFFNPEESELDLTYGNRYKNVKLPDAYERLILDVFCGSQMHFVRSDELLEAWRIFTPLLHQIELEKPKPIPYIYGSRGPTEADELMKRVGFQYEGTYKWVNPHKL

1111111100001111111111111110000000000000000000000000000000000000000000000000000000000000000000000000000000000000000000000000000000000000000000000000000000000000000000000000000000000000000000000000000000000000000000000000000000000000000000000000000000000000000000000000000000000000000000000000000000000000000000000000000000000000000000000000000000000000000000000000000000000000000000000000000000000000000000000000000000000000000000000000000000000000000000000000000000000000000000000000000000000000000000000000001111

>DM_train199

DPDQFGPDLIEQLAQSGKYSQDNTKGDAMIGVKQPLPKAVLRTQHDKNKEAISILDFGVIDDGVTDNYQAIQNAIDAVASLPSGGELFIPASNQAVGYIVGSTLLIPGGVNIRGVGKASQLRAKSGLTGSVLRLSYDSDTIGRYLRNIRVTGNNTCNGIDTNITAEDSVIRQVYGWVFDNVMVNEVETAYLMQGLWHSKFIACQAGTCRVGLHFLGQCVSVSVSSCHFSRGNYSADESFGIRIQPQTYAWSSEAVRSEAIILDSETMCIGFKNAVYVHDCLDLHMEQLDLDYCGSTGVVIENVNGGFSFSNSWIAADADGTEQFTGIYFRTPTSTQSHKIVSGVHINTANKNTAANNQSIAIEQSAIFVFVSGCTLTGDEWAVNIVDINECVSFDKCIFNKPLRYLRSGGVSVTDCYLAGITEVQKPEGRYNTYRGCSGVPSVNGIINVPVAVGATSGSAAIPNPGNLTYRVRSLFGDPASSGDKVSVSGVTINVTRPSPVGVALPSMVEYLAI

1110000000000000000000000000000000000000000000000000000000000000000000000000000000000000000000000000000000000000000000000000000000000000000000000000000000000000000000000000000000000000000000000000000000000000000000000000000000000000000000000000000000000000000000000000000000000000000000000000000000000000000000000000000000000000000000000000000000000000000000000000000000000000000000000000000000000000000000000000000000000000000000000000000000000000000000000000000000000000000000000000000000000000000000000000000000

>DM_train200

MPIHDKSPRPQEFAAVDLGSNSFHMVIARVVDGAMQIIGRLKQRVHLADGLGPDNMLSEEAMTRGLNCLSLFAERLQGFSPASVCIVGTHTLRQALNATDFLKRAEKVIPYPIEIISGNEEARLIFMGVEHTQPEKGRKLVIDIGGGSTELVIGENFEPILVESRRMGCVSFAQLYFPGGVINKENFQRARMAAAQKLETLTWQFRIQGWNVAMGASGTIKAAHEVLMEMGEKDGIITPERLEKLVKEVLRHRNFASLSLPGLSEERKTVFVPGLAILCGVFDALAIRELRLSDGALREGVLYEMEGRFRHQDVRSRTASSLANQYHIDSEQARRVLDTTMQMYEQWREQQPKLAHPQLEALLRWAAMLHEVGLNINHSGLHRHSAYILQNSDLPGFNQEQQLMMATLVRYHRKAIKLDDLPRFTLFKKKQFLPLIQLLRLGVLLNNQRQATTTPPTLTLITDDSHWTLRFPHDWFSQNALVLLDLEKEQEYWEGVAGWRLKIEEESTPEIAA

111111111110000000000000000000000000000000000000000000000000000000000000000000000000000000000000000000000000000000000000000000000000000000000000000000000000000000000000000000000000000000000000000000000000000000000000000000000000000000000000000000000000000000000000000000000000000000000000000000000000000000000000000000000000000000000000000000000000000000000000000000000000000000000000000000000000000000000000000000000000000000000000000000000000000000000000000000000000000000000000000000000000000000000000000001111

>DM_train201

GSPEFMHTINEEERREFIKHINSVLAGDPDVGSRVPINTETFEFFDQCKDGLILSKLINDSVPDTIDERVLNKQRNNKPLDNFKCIENNNVVINSAKAMGGISITNIGAGDILEGREHLILGLVWQIIRRGLLGKIDITLHPELYRLLEEDETLDQFLRLPPEKILLRWFNYHLKAANWPRTVSNFSKDVSDGENYTVLLNQLAPELCSRAPLQTTDVLQRAEQVLQNAEKLDCRKYLTPTAMVAGNPKLNLAFVAHLFNTHPGLEPLNEEEKPEIEPFDAEGEREARVFTLWLNSLDVTPSIHDFFNNLRDGLILLQAYDKITPNTVNWKKVNKAPASGDEMMRFKAVENCNYAVDLGKNQGFSLVGIQGADITDGSRTLTLALVWQMMRMNITKTLHSLSRGGKTLSDSDMVAWANSMAAKGGKGSQIRSFRDPSISTGVFVLDVLHGIKSEYVDYNLVTDGSTEELAIQNARLAISIARKLGAVIFILPEDIVAVRPRLVLHFIGSLMAV

111111110000000000000000000000000000000000000000000000000000000000000000000111000000000000000000000000000000000000000000000000000000000011111111111111110000000000000000000000000000000000000000000000000000000000000000000000000000000000000000000000000000000000000000000111111111111100000000000000000000000000000000000000000000000000000000000000000000000000000000000000000000000000000000000000000000000011111100000000000000000000000000000000000000000000000000000000000000000000000000000000000000000000000000000000000

>DM_train202

GMPEIHQSIAQHYHERTKYDPETIASKSQRLDWAKQPVPFKEYKIGSAIDLKPYLQETPEVFVNDTNGQWWQRLSRLLFRSYGLTARMPSMGNTVYLRAAPSAGGLYPAEVYVVSRGTPLLSPGLYNYQCRTHSLIHYWESDVWQSLQEACFWHPALESTQLAIIVTAVFYRSAWRYEDRAYRRICLDTGHLLGNIELSAAITDYRPHLIGGFIDEAVNDLLYIDPLQEGAIAVLPLADLLDIQQNISPGCTALPSATETNYPQVPDGELLKYFHHHTQISASITGKLNLPTVIQEKSLEDKYNFPFCLKISTVSAPIYWGENLSDLEITMHKRRSTRAYNGEELTFDELKALLDFTYQPQNYIDQSLDNSPDYFDLNLIETFIAVCGVQGLEAGCYYYAPKAQELRQIRFKNFRRELHFLCLGQELGRDAAAVIFHTSDLKSAIAQYGDRVYRYLHMDAGHLGQRLNLAAIQLNLGVSGIGGFFDDQVNEVLGIPNDEAVIYITTLGRPR

1111100000000000000000000001100000000000000000000000000000011111000000000000000000000000000000000000000000000000000000000000000000000000000000000000000000000000000000000000000000000000000000000000000000000000000000000000000000000000000000000000000000000000000000000000000000000000000000111111111111000000000000000000000000000000000000000000000000000000000000000000000000000000000000000000000000000000000000000000000000000000000000000000000000000000000000000000000000000000000000000000000000000000000000000000000

>DM_train204

MSNDKTGKSLEQENSERDVEIRDRNYFRKLSLFDDTVIAGAEMIGTSYDVFGKYCNVGSCMNSLFDERKINASEDNFKKVTILGKTLKVPYYIDCYSVGDLKYTNASGESIESYQSNISSKSRIKGNYLFFSASLKVDFDTDSLTDFENAFSRIQYTYDLYILKSSAEALKEFLKESVKTALDKADTEEDMNDLFNTWGSHFLSGVVMGGCAQYSSSTNKYTSNLTNSFDVVAAASFAGFIGLSARTGNSFMEDIKKFRSASNIKTHAIGGDLSRFDPFGGATSADQPSAEEIAAAKKAFEDWKASVPNAPELVNFADSNPLTGIWELCSDRTQKAKLKKHFETVWAPAESAKRRVHADYIDEIIIGINNTNTPPEGYIGLKSTKDENLNSKGNICLFMHKAKYDPNIDNKDCITELKFITVRDKSPEGDWVKIPQDIYISPNQYLYLCYLPAKYSAEKAIKDIQLLCSSCGSSMILPYGYNDVLDERGERANATEDDNVHYLIYSAGWK

111111100000000000000000000000000000000000000000000000000000000000000000000000000000000000000000000000000000000000000000000000000000000000000000000000000000000000000000000000000000000000000000000000000000000000000000000000000000000000000000000000000000000000000000000000000000000000000000000000000000000000000000000000000000000000000000000000000000000000000000000000000000000000000000000000000000000000000000000000000000000000000000000000000000000000000000000000000000011110000000000000000000000000000000000001

>DM_train206

GVSKEEYKDVETAKKEKEQLGELMEPALGYVVKVPVSSFENKKVDISDIEVITNGNLDDVPYKANSSKYNYPDIKTKDSSLQYVRSGYVIDGEHSGSNEKGYVYYKGNSPAKELPVNQLLTYTGSWDFTSNANLNNEEGRPNYLNDDYYTKFIGKRVGLVSGDAKPAKHKYTSQFEVDFATKKMTGKLSDKEKTIYTVNADIRGNRFTGAATASDKNKGKGESYNFFSADSQSLEGGFYGPKAEEMAGKFVANDKSLFAVFSAKHNGSNVDTVRIIDASKIDLTNFSISELNNFGDASVLIIDGKKIKLAGSGFTNKHTIEINGKTMVAVACCSNLEYMKFGQLWQQAEGGKPENNSLFLQGERTATDKMPKGGNYKYIGTWDAQVSKENNWVATADDDRKAGYRTEFDVDFGNKNLSGKLFDKNGVNPVFTVDPKIDGNGFTGKAKTSDAGFALDSGSSRYENVKFNDVAVSGGFYGPTAAELGGQFHHKSENGSVGAVFGAKQQVKK

11110000000000000000000000000000000000000000000000000000000000000000000000000000000000000000000000000000000000000000000000000000000000000000000000000000000000000000000000000000000000000000000000000000000000000000000000000000000000000000000000000000000000000000000000000000000000000000000000000000000000000000000000000000000000000000000000000000000000000000000000000000000000000000000000000000000000000000000000000000000000000000000000000000000000000000000000000000000000000000000000000000000000000000000000000

>DM_train207

MFRLGTTPRATTYDPDARIGEVASRFGLPTRVLIEIVRTESFQRSLARVTSGKPVVLDLRELDSDLASWIATHARLVEPALRELVRTVAPDVEPRVRFRGLPHRFRRVERIRPMDGALISIEGVVREVRGAERLEHAIVDTGSELVAVRLHGHRLGPGLRVEILGIVRSATLDALEVHKKDPIPEVHPDPAELEEFRELADKDPLTTFARAIAPLPGAEEVGKMLALQLFSCVGKNSERLHVLLAGYPVVCSEILHHVLDHLAPRGVYVDLRRTELTDLTAVLKEDRGWALRAGAAVLADGGILAVDHLEGAPEPHRWALMEAMDKGTVTVDGIALNARCAVLAAINPGEQWPSDPPIARIDLDQDFLSHFDLIAFLGVDPRPGEPEEQDTEVPSYTLLRRYLLYAIREHPAPELTEEARKRLEHWYETRREEVEERLGMGLPTLPVTRRQLESVERLAKAHARMRLSDDVEPEDVDIAAELVDWYLETAMQIPGGDEIRISSLKP

11111111111111111000000000000000000000000000000000000000000000000000000000000000000000000000000000000000000000000000000000000000000000000000000000000000000000000000000000000000000000000000000000000000000000000000000000000000000000000000000000000000000000000000000000000000000000000000000000000000000000000000000000000000000000000000000000000000000000110000000000000000000000000001111111111111100000000000000000000000000000000000000000000000000000000000000000000000000000000000000000000000000011111111111111

>DM_train208

GAASEAEYGKVSKAWTLHADGSQEYRSSMELTLFTHTAMNSTYGESFIVYNPDFQTLKIHSSYTRQKDGTIVKTPDNAFVEVLPRFAADAPAYNQLKEMVVVHTGLELGATIYLDYSIITKPGYYPALDINERLQETSPVKECKVSISVPEGTPLACGLYGSPVKAVEESHDGIKEVHWTLRNIPASSREAFQPKNREASPHLVASTYPSGKAALATLDKRLKESQGYESKTFAQFLTDKSGNEQEKVNIIRDHILNNLSTCPIPMAMTGYTVRDIDTVLRSAYGTPLEIAQLLNVMLNAAGIPSEVLAVYPGHLDTDACGLAAIQTLAVKATVDGKDQYLSASPLTNRGGLDKVVSLSGTSIEIETTPIQIKESRSVAISADQAKDGFAICVLPAISAGIDSWGMSALNSKRSNLFELPSLIREEVTYTVTPAEGMKLQTSTQEQVISKPFGKVTRTITPRGNTIEVVRTIELNKQQFTPAEYSDVRSLIHEWTNPDNRVLLFSL

10000000000000000000000000000000000000000000000000000000000000000000000000000000000000000000000000000000000000000000000000000000000000000000000000000000000000000000000000000000000000000000000000000000000000000000000000000000000000000000000000000000000000000000000000000000000000000000000000000000000000000000000000000000000000000000000000000000000000000000000000000000000000000000000000000000000000000000000000000000000000000000000000000000000000000000000000000000000000000000000000000000000000000000000000

>DM_train211

CGIVGIAGVMPVNQSIYDALTVLQHRGQDAAGIITIDANNCFRLRKANGLVSDVFEARHMQRLQGNMGIGHVRYPTAGSSSASEAQPFYVNSPYGITLAHNGNLTNAHELRKKLFEEKRRHINTTSDSEILLNIFASELDNFRHYPLEADNIFAAIAATNRLIRGAYACVAMIIGHGMVAFRDPNGIRPLVLGKRDIDENRTEYMVASESVALDTLGFDFLRDVAPGEAIYITEEGQLFTRQCADNPVSNPCLFEYVYFARPDSFIDKISVYSARVNMGTKLGEKIAREWEDLDIDVVIPIPETSCDIALEIARILGKPYRQGFVKNRYVGRTFIMPGQQLRRKSVRRKLNANRAEFRDKNVLLVDDSIVRGTTSEQIIEMAREAGAKKVYLASAAPEIRFPNVYGIDMPSATELIAHGREVDEIRQIIGADGLIFQDLNDLIDAVRAENPDIQQFECSVFNGVYVTKDVDQGYLDFLDTLRNDDAKAVQRQNEVENLEMHNEG

000000000000000000000000000000000000000000000000000000000000000000000000000000000000000000000000000000000000000000000000000000000000000000000000000000000000000000000000000000000000000000000000000000000000000000000000000000000000000000000000000000000000000000000000000000000000000000000000000000000000000000000000000000000000000000000000111111110000000000000000000000000000000000000000000000000000000000000000000000000000000000000000000000000000000000000000000000000000000000000011111111111111111111111111

>DM_train212

MATKKATMIIEKDFKIAEIDKRIYGSFIEHLGRAVYGGIYEPGHPQADENGFRQDVIELVKELQVPIIRYPGGNFVSGYNWEDGVGPKEQRPRRLDLAWKSVETNEIGLNEFMDWAKMVGAEVNMAVNLGTRGIDAARNLVEYCNHPSGSYYSDLRIAHGYKEPHKIKTWCLGNAMDGPWQIGHKTAVEYGRIACEAAKVMKWVDPTIELVVCGSSNRNMPTFAEWEATVLDHTYDHVDYISLHQYYGNRDNDTANYLALSLEMDDFIRSVVAIADYVKAKKRSKKTIHLSFDEWNVWYHSNEADKLIEPWTVAPPLLEDIYNFEDALLVGCMLITLMKHADRVKIACLAQLVNVIAPIMTEKNGPAWKQTIYYPFMHASVYGRGVALHPVISSPKYDSKDFTDVPYLESIAVYNEEKEEVTIFAVNRDMEDALLLECDVRSFEDYRVIEHIVLEHDNVKQTNSAQSSPVVPHRNGDAQLSDRKVSATLPKLSWNVIRLGKR

1111000000000000000000000000000000000000000000000000000000000000000000000000000000000000000000000000000000000000000000000000000000000000000000000000000000000000000000000000000000000000000000000000000000000000000000000000000000000000000000000000000000000000000000000000000000000000000000000000000000000000000000000000000000000000000000000000000000000000000000000000000000000000000000000000000000000000000000000000000000000000000000000000000000000000000000000000000000000000000000000000000000000000000001

>DM_train214

METKDLIVIGGGINGAGIAADAAGRGLSVLMLEAQDLACATSSASSKLIHGGLRYLEHYEFRLVSEALAEREVLLKMAPHIAFPMRFRLPHRPHLRPAWMIRIGLFMYDHLGKRTSLPGSTGLRFGANSVLKPEIKRGFEYSDCWVDDARLVLANAQMVVRKGGEVLTRTRATSARRENGLWIVEAEDIDTGKKYSWQARGLVNATGPWVKQFFDDGMHLPSPYGIRLIKGSHIVVPRVHTQKQAYILQNEDKRIVFVIPWMDEFSIIGTTDVEYKGDPKAVKIEESEINYLLNVYNTHFKKQLSRDDIVWTYSGVRPLCDDESDSPQAITRDYTLDIHDENGKAPLLSVFGGKLTTYRKLAEHALEKLTPYYQGIGPAWTKESVLPGGAIEGDRDDYAARLRRRYPFLTESLARHYARTYGSNSELLLGNAGTVSDLGEDFGHEFYEAELKYLVDHEWVRRADDALWRRTKQGMWLNADQQSRVSQWLVEYTQQRLSLAS

110000000000000000000000000000000000000000000000000000000000000000000000000000000000000000000000000000000000000000000000000000000000000000000000000000000000000000000000000000000000000000000000000000000000000000000000000000000000000000000000000000000000000000000000000000000000000000000000000000000000000000000000000000000000000000000000000000000000000000000000000000000000000000000000000000000000000000000000000000000000000000000000000000000000000000000000000000000000000000000000000000000000000000000

>DM_train215

MVYDLIVIGGGSGGMAAARRAARHNAKVALVEKSRLGGTCVNVGCVPKKIMFNAASVHDILENSRHYGFDTKFSFNLPLLVERRDKYIQRLNNIYRQNLSKDKVDLYEGTASFLSENRILIKGTKDNNNKDNGPLNEEILEGRNILIAVGNKPVFPPVKGIENTISSDEFFNIKESKKIGIVGSGYIAVELINVIKRLGIDSYIFARGNRILRKFDESVINVLENDMKKNNINIVTFADVVEIKKVSDKNLSIHLSDGRIYEHFDHVIYCVGRSPDTENLKLEKLNVETNNNYIVVDENQRTSVNNIYAVGDCCMVKKSKEIEDLNLLKLYNEERYLNKKENVTEDIFYNVQLTPVAINAGRLLADRLFLKKTRKTNYKLIPTVIFSHPPIGTIGLSEEAAIQIYGKENVKIYESKFTNLFFSVYDIEPELKEKTYLKLVCVGKDELIKGLHIIGLNADEIVQGFAVALKMNATKKDFDETIPIHPTAAEEFLTLQPWMK

00000000000000000000000000000000000000000000000000000000000000000000000000000000000000000000000000000000000000000011111111111111111111111111000000000000000000000000000000000000000000000000000000000000000000000000000000000000000000000000000000000000000000000000000000000000000000000000000000000000000000000000000000000111111111111111111111111111111000000000000000000000000000000000000000000000000000000000000000000000000000000000000000000000000000000000000000000000000000000000000000000000000000001111

>DM_train216

TEKKYIVALDQGTTSSRAVVMDHDANIISVSQREFEQIYPKPGWVEHDPMEIWATQSSTLVEVLAKADISSDQIAAIGITNQRETTIVWEKETGKPIYNAIVWQCRRTAEICEHLKRDGLEDYIRSNTGLVIDPYFSGTKVKWILDHVEGSRERARRGELLFGTVDTWLIWKMTQGRVHVTDYTNASRTMLFNIHTLDWDDKMLEVLDIPREMLPEVRRSSEVYGQTNIDGKGGTRIPISGIAGDQQAALFGQLCVKEGMAKNTYGTGCFMLMNTGEKAVKSENGLLTTIACGPTGEVNYALEGAVFMAGASIQWLRDEMKLINDAYDSEYFATKVQNTNGVYVVPAFTGLGAPYWDPYARGAIFGLTRGVNANHIIRATLESIAYQTRDVLEAMQADSGIRLHALRVDGGAVANNFLMQFQSDILGTRVERPEVREVTALGAAYLAGLAVGFWQNLDELQEKAVIEREFRPGIETTERNYRYAGWKKAVKRAMAWEEHD

10000000000000000000000000000000000000000000000000000000000000000000000000000000000000000000000000000000000000000000000000000000000000000000000000000000000000000000000000000000000000000000000000000000000000000000000000000000000011111110000000000000000000000000000000000000000000000000000000000000000000000000000000000000000111100000000000000000000000000000000000000000000000000000000000000000000000000000000000000000000000000000000001110000000000000000000000000011100000000000000000000000000000000000

>DM_train217

AFYIATFDIGTTEVKAALADRDGGLHFQRSIALETYGDGNGPVEQDAGDWYDAVQRIASSWWQSGVDARRVSAIVLSGQMQNFLPLDQDHEPLHRAVLYSDKRPLKEAEEINARHGADNLWSALENPMTAASILPKLVFWRASFPQAFGRLRHVVLGAKDYVVLRLTGRHATDRTNASTTGLYRPKDDAWHVELLADYGFSLDLMPRLLEPGEQVGGVSALAARQTGFVSGTPVLCGLGDAGAATLGVGVLDDEDAYLHLGTTGWLARLTQTDPVGDMPVGTIFRLAGIIAGKTLQVAPVLNAGNILQWALTLVGHRPGEDCAEYFHMAAAEVQGVTVPDGLLFVPYLHAERCPVELPAPRGALLGVTGATTRAQILLAVLEGAALSLRWCAELLGMEKVGLLKVVGGGARSEAWLRMIADNLNVSLLVKPDAHLHPLRGLAALAAVELEWSHSIQDFLREADLREPASNILHPQPCDEGRRRRKFERFKQCVETLGRLD

00000000000000000000000000000000000000000000000000000000000000000000000000000000000000000000000000000000000000000000000000000000000000000000000000000000000000000000000000000000000000000000000000000000000000000000000000000000000000000000000000000000000000000000000000000000000000000000000000000000000000000000000000000000000000000000000000000000000000000000000000000000000000000000000000000000000000000000000000000000000000000000000000000000000000000000000000000000011100000000000000000000000000000011

>DM_train218

MASLQLLPSPTPNLEIKYTKIFINNEWQNSESGRVFPVCNPATGEQVCEVQEADKVDIDKAVQAARLAFSLGSVWRRMDASERGRLLDKLADLVERDRATLATMESLNGGKPFLQAFYIDLQGVIKTLRYYAGWADKIHGMTIPVDGDYFTFTRHEPIGVCGQIIPWNFPLLMFTWKIAPALCCGNTVVIKPAEQTPLSALYMGALIKEAGFPPGVVNILPGYGPTAGAAIASHIGIDKIAFTGSTEVGKLIQEAAGRSNLKRVTLELGGKSPNIIFADADLDYAVEQAHQGVFFNQGQCCTAGSRIFVEESIYEEFVKRSVERAKRRIVGSPFDPTTEQGPQIDKKQYNKILELIQSGVAEGAKLECGGKGLGRKGFFIEPTVFSNVTDDMRIAKEEIFGPVQEILRFKTMDEVIERANNSDFGLVAAVFTNDINKALMVSSAMQAGTVWINCYNALNAQSPFGGFKMSGNGREMGEFGLREYSEVKTVTVKIPQKNS

0000000000000000000000000000000000000000000000000000000000000000000000000000000000000000000000000000000000000000000000000000000000000000000000000000000000000000000000000000000000000000000000000000000000000000000000000000000000000000000000000000000000000000000000000000000000000000000000000000000000000000000000000000000000000000000000000000000000000000000000000000000000000000000000000000000000000000000000000000000000000000000000000000000000000000000000011111111111111111111100000000000000000000000

>DM_train220

LEPRHAMLLRCRLPGGVITTKQWQAIDKFAGENTIYGSIRLTNRQTFQFHGILKKNVKPVHQMLHSVGLDALATANDMNRNVLCTSNPYESQLHAEAYEWAKKISEHLLPRTRAYAEIWLDQEKVATTDEEPILGQTYLPRKFKTTVVIPPQNDIDLHANDMNFVAIAENGKLVGFNLLVGGGLSIEHGNKKTYARTASEFGYLPLEHTLAVAEAVVTTQRDWGNRTDRKNAKTKYTLERVGVETFKAEVERRAGIKFEPIRPYEFTGRGDRIGWVKGIDDNWHLTLFIENGRILDYPARPLKTGLLEIAKIHKGDFRITANQNLIIAGVPESEKAKIEKIAKESGLMNAVTPQRENSMACVSFPTCPLAMAEAERFLPSFIDNIDNLMAKHGVSDEHIVMRVTGCPNGCGRAMLAEVGLVGKAPGRYNLHLGGNRIGTRIPRMYKENITEPEILASLDELIGRWAKEREAGEGFGDFTVRAGIIRPVLDPARDLWD

11111111000000000000000000000000000000000000000000000111110000000000000011100000000000000000000000000000000000111111111111111111111111111100000000000000000000000000000000000000000000000000000000000000000000000000000000000000000000000000000000000000000000000000000000000000000000000000000000000000000000000000000000000000000000000000000000000000000000000000000000000000000000000000000000000000000000000000000000000000000000000000000000000000000000000000000000000000000000000000000000000000000000000

>DM_train221

MAHHHHHHMASPASTNPAHDHFETFVQAQLCQDVLSSFQGLCRALGVESGGGLSQYHKIKAQLNYWSAKSLWAKLDKRASQPVYQQGQACTNTKCLVVGAGPCGLRAAVELALLGARVVLVEKRIKFSRHNVLHLWPFTIHDLRALGAKKFYGRFCTGTLDHISIRQLQLLLLKVALLLGVEIHWGVKFTGLQPPPRKGSGWRAQLQPNPPAQLASYEFDVLISAAGGKFVPEGFTIREMRGKLAIGITANFVNGRTVEETQVPEISGVARIYNQKFFQSLLKATGIDLENIVYYKDETHYFVMTAKKQCLLRLGVLRQDLSETDQLLGKANVVPEALQRFARAAADFATHGKLGKLEFAQDARGRPDVAAFDFTSMMRAESSARVQEKHGARLLLGLVGDCLVEPFWPLGTGVARGFLAAFDAAWMVKRWAEGAGPLEVLAERESLYQLLSQTSPENMHRNVAQYGLDPATRYPNLNLRAVTPNQVQDLYDMMDKE

11111111111111000000000000000000000000000000000000000000000000000000000000000000000000000000000000000000000000000000000000000000000000000000000000000000000000000000000000000000000000000000000000000000000000000000000000000000000000000000000000000000000000000000000000001111000000000000000000000000000000000000000000000000000000000000000000000000000000000000000000000000000000000000000000000000000000000000000000000000000000000000000000000000000000000000000000000000000000000000000000000000000000000

>DM_train222

GKDDDNVETGAFDPSKPVAISDFTPKEGGAYQKLLIYGENFGTDVSKVKVKIGGKDAIVINVKSTYVYCFVPSGAFSGEIEITVGEGENAVTTTASTTFSYEKKMVVGTLCGYRNNRDDQGWRDGPFDGPEGVKCCGFSDNGRLAFDPLNKDHLYICYDGHKAIQLIDLKNRMLSSPLNINTIPTNRIRSIAFNKKIEGYADEAEYMIVAIDYDGKGDESPSVYIIKRNADGTFDDRSDIQLIAAYKQCNGATIHPINGELYFNSYEKGQVFRLDLVDYFKTIKNGGSWDPIVKNNPNTFKQLFTIADPSWEFQIFIHPTGKYAYFGVINNHYFMRSDYDEIKKEFITPYNFVGGYKQSGYRDDVGTEARMNNPCQGVFVKNPDYTGEEEYDFYFVDRLNFCVRKVTPEGIVSTYAGRGASTSLADGNQWGTDDGDLREVARFRDVSGLVYDDVKEMFYVHDQVGHTIRTISMEQEENVAGDENIPEDESTVESNE

1111110000000000000000000000000000000000000000000000000000000000000000000000000000000000000000000000000000000000000000000000000000000000000000000000000000000000000000000000000000000000000000000000000000000000000000000000000000000000000000000000000000000000000000000000000000000000000000000000000000000000000000000000000000000000000000000000000000000000000000000000000000000000000000000000000000000000000000000000000000000000000000000000000000000000000000000000000000000000000001111111111111111111

>DM_train223

ATSDSNMLLNYVPVYVMLPLGVVNVDNVFEDPDGLKEQLLQLRAAGVDGVMVDVWWGIIELKGPKQYDWRAYRSLLQLVQECGLTLQAIMSFHQCGGNVGDIVNIPIPQWVLDIGESNHDIFYTNRSGTRNKEYLTVGVDNEPIFHGRTAIEIYSDYMKSFRENMSDFLESGLIIDIEVGLGPAGELRYPSYPQSQGWEFPGIGEFQCYDKYLKADFKAAVARAGHPEWELPDDAGKYNDVPESTGFFKSNGTYVTEKGKFFLTWYSNKLLNHGDQILDEANKAFLGCKVKLAIKVSGIHWWYKVENHAAELTAGYYNLNDRDGYRPIARMLSRHHAILNFTCLEMRDSEQPSDAKSGPQELVQQVLSGGWREDIRVAGENALPRYDATAYNQIILNARPQGVNNNGPPKLSMFGVTYLRLSDDLLQKSNFNIFKKFVLKMHADQDYCANPQKYNHAITPLKPSAPKIPIEVLLEATKPTLPFPWLPETDMKVDG

111110000000000000000000000000000000000000000000000000000000000000000000000000000000000000000000000000000000000000000000000000000000000000000000000000000000000000000000000000000000000000000000000000000000000000000000000000000000000000000000000000000000000000000000000000000000000000000000000000000000000000000000000000000000000000000000000000000000000000000000000000000000000000000000000000000000000000000000000000000000000000000000000000000000000000000000000000000000000000000000000000000000000

>DM_train224

MTSRDGYQWTPETGLTQGVPSLGVISPPTNIEDTDKDGPWDVIVIGGGYCGLTATRDLTVAGFKTLLLEARDRIGGRSWSSNIDGYPYEMGGTWVHWHQSHVWREITRYKMHNALSPSFNFSRGVNHFQLRTNPTTSTYMTHEAEDELLRSALHKFTNVDGTNGRTVLPFPHDMFYVPEFRKYDEMSYSERIDQIRDELSLNERSSLEAFILLCSGGTLENSSFGEFLHWWAMSGYTYQGCMDCLMSYKFKDGQSAFARRFWEEAAGTGRLGYVFGCPVRSVVNERDAARVTARDGREFVAKRVVCTIPLNVLSTIQFSPALSTERISAMQAGHVSMCTKVHAEVDNKDMRSWTGIAYPFNKLCYAIGDGTTPAGNTHLVCFGNSANHIQPDEDVRETLKAVGQLAPGTFGVKRLVFHNWVKDEFAKGAWFFSRPGMVSECLQGLREKHGGVVFANSDWALGWRSFIDGAIEEGTRAARVVLEELGTKREVKARL

000000000000000000000000000000011111111000000000000000000000000000000000000000000000000000000000000000000000000000000000000000000000000000000000000000000000000000000000000000000000000000000000000000000000000000000000000000000000000000000000000000000000000000000000000000000000000000000000000000000000000000000000000000000000000000000000000000000000000000000000000000000000000000000000000000000000000000000000000000000000000000000000000000000000000000000000000000000000000000000000000000111111111

>DM_train225

MAQILPIRFQEHLQLQNLGINPANIGFSTLTMESDKFICIREKVGEQAQVVIIDMNDPSNPIRRPISADSAIMNPASKVIALKAGKTLQIFNIEMKSKMKAHTMTDDVTFWKWISLNTVALVTDNAVYHWSMEGESQPVKMFDRHSSLAGCQIINYRTDAKQKWLLLTGISAQQNRVVGAMQLYSVDRKVSQPIEGHAASFAQFKMEGNAEESTLFCFAVRGQAGGKLHIIEVGTPPTGNQPFPKKAVDVFFPPEAQNDFPVAMQISEKHDVVFLITKYGYIHLYDLETGTCIYMNRISGETIFVTAPHEATAGIIGVNRKGQVLSVCVEEENIIPYITNVLQNPDLALRMAVRNNLAGAEELFARKFNALFAQGNYSEAAKVAANAPKGILRTPDTIRRFQSVPAQPGQTSPLLQYFGILLDQGQLNKYESLELCRPVLQQGRKQLLEKWLKEDKLECSEELGDLVKSVDPTLALSVYLRANVPNKVIQCFAE

00000000000000000000000000000000000000000000000000000000000000000000000000000000000000000000000000000000000000000000000000000000000000000000000000000000000000000000000000000000000000000000000000000000000000000000000000000000000000000000000000000000000000000000000000000000000000000000000000000000000000000000000000000000000000000000000000000000000000000000000000000000000000000000000000000000000000000000000000000000000000000000000000000000000000000000000000000000000000000000000000000001111111

>DM_train226

MDYTYADDSLTLHTDMYQINMMQTYWELGRADLHAVFECYFREMPFNHGYAIFAGLERLVNYLENLTFTESDIAYLREVEEYPEDFLTYLANFEFKCTVRSALEGDLVFNNEPLIQIEGPLAQCQLVETALLNMVNFQTLIATKAARIKSVIGDDPLLEFGTRRAQELDAAIWGTRAAYIGGADATSNVRAGKIFGIPVSGTHAHSLVQSYGNDYEAFMAYAKTHRDCVFLVDTYDTLKAGVPSAIRVAREMGDKINFLGVRIDSGDMAYISKRVREQLDEAGFTEAKIYASNDLDENTILNLKMQKSKIDVWGVGTKLITAYDQPALGAVFKLVSIEGEDGQMKDTIKLSSNAEKVTTPGKKQVWRITRKSDKKSEGDYVTLWNEDPRQEEEIYMFHPVHTFINKYVRDFEARPVLQDIFVEGKRVYELPTLDEIKQYAKENLDSLHEEYKRDLNPQKYPVDLSTDCWNHKMNLLEKVRKDVKHLTETVNKEA

11100000000000000000000000000000000000000000000000000000000000000000000000000000000000000000000000000000000000000000000000000000000000000000000000000000000000000000000000000000000000000000000000000000000000000000000000000000000000000000000000000000000000000000000000000000000000000000000000000000000000000000000000000000000000000000000000000000000000000000000000000000000000000000000000000000000000000000000000000000000000000000000000000000000000000000000000000000000000000000000000000111111111

>DM_train227

TGNLVTKNSLTPDVRNGIDFKIADLSLADFGRKELRIAEHEMPGLMSLRREYAEVQPLKGARISGSLHMTVQTAVLIETLTALGAEVRWASCNIFSTQDHAAAAVVVGPHGTPDEPKGVPVFAWKGETLEEYWWAAEQMLTWPDPDKPANMILDDGGDATMLVLRGMQYEKAGVVPPAEEDDPAEWKVFLNLLRTRFETDKDKWTKIAESVKGVTEETTTGVLRLYQFAAAGDLAFPAINVNDSVTKSKFDNKYGTRHSLIDGINRGTDALIGGKKVLICGYGDVGKGCAEAMKGQGARVSVTEIDPINALQAMMEGFDVVTVEEAIGDADIVVTATGNKDIIMLEHIKAMKDHAILGNIGHFDNEIDMAGLERSGATRVNVKPQVDLWTFGDTGRSIIVLSEGRLLNLGNATGHPSFVMSNSFANQTIAQIELWTKNDEYDNEVYRLPKHLDEKVARIHVEALGGHLTKLTKEQAEYLGVDVEGPYKPDHYRY

11111111100000000000000000000000000000000000000000000000000000000000000000000000000000000000000000000000000000000000000000000000000000000000000000000000000000000000000000000000000000000000000000000000000000000000000000000000000000000000000000000000000000000000000000000000000000000000000000000000000000000000000000000000000000000000000000000000000000000000000000000000000000000000000000000000000000000000000000000000000000000000000000000000000000000000000000000000000000000000000000000000000000

>DM_train228

MTANGTAEAVQIQFGLINCGNKYLTAEAFGFKVNASASSLKKKQIWTLEQPPDEAGSAAVCLRSHLGRYLAADKDGNVTCEREVPGPDCRFLIVAHDDGRWSLQSEAHRRYFGGTEDRLSCFAQTVSPAEKWSVHIAMHPQVNIYSVTRKRYAHLSARPADEIAVDRDVPWGVDSLITLAFQDQRYSVQTADHRFLRHDGRLVARPEPATGYTLEFRSGKVAFRDCEGRYLAPSGPSGTLKAGKATKVGKDELFALEQSCAQVVLQAANERNVSTRQGMDLSANQDEETDQETFQLEIDRDTKKCAFRTHTGKYWTLTATGGVQSTASSKNASCYFDIEWRDRRITLRASNGKFVTSKKNGQLAASVETAGDSELFLMKLINRPIIVFRGEHGFIGCRKVTGTLDANRSSYDVFQLEFNDGAYNIKDSTGKYWTVGSDSAVTSSGDTPVDFFFEFCDYNKVAIKVGGRYLKGDHAGVLKASAETVDPASLWEY

1111111000000000000000000000000000000000000000000111111110000000000000000000000000000000000000000000000000000000000000000000000000000000000000000000000000000000000000000000000000000000000000000000000000000000000000000000000000000000000000000000000000000000000000000000000000011110000000000000000000000000000000000000000000000000000000000000000000000000000000000000000000000000000000000000000000000000000000000000000000000000000000000000000000000000000000000000000000000000000000000000000000000

>DM_train229

GGTAYWKNPDQFTAFNTGLHALLREKSYNFFLLGEPRADIYGDNPIGGEASQGMERLPFNTINKENVGISNYGDMYKIINQINQMIAKTTETTILTEATQNYYLGEAYGMRAYLYFHLLRSWGDVVLYLDYTEGQNLDLSNITKGVSPATEVMEQIKKDIQASENAFGSDYSFKLGRHFWSAAATQMLKGEAYLWSGRQMNGGNSDYTIAKNAFENVKKADVGLVTSSFKDIFSFENKKNKEMIFTIHNGKDEYEMWGGYYRMRLIPAQDKMVKIYCDENGNSFVGTPDAQLNGLTQLQVRREFYFKGFRNNDTRWTTSLKAVYKKDAQGVVSYFGPITYKFQGTMLEGGSTRSFLDDFPIYRYADCLLQLAMAKVLLGEDPTEEINAVRERAYGSKYFNEHKAEIAYPNDNDPEFYTDNKWMKPDNAGALEAILKERLREFMFEGKRWYDIRLLGWDYVHQYSSAEQSRLLWPIDAGTLTNNSALKQTPGYE

1111100000000000000000000000000000000000000000000000000000000000000000000000000000000000000000000000000000000000000000000000000000000111111111100000000000000000000000000000000000000000000000000000000000000000000000000000000000000000000000000000000000000000000000000000000000000000000000000000000000000000000000000000000000000000000000000000000000000000000000000000000000000000000000000000000000000000000000000000000000000000000000000000000000000000000000000000000000000000000000000000000000000

>DM_train231

SVTVQQPQLTLTAAVIGDGAPANGKTAITVEFTVADFEGKPLAGQEVVITTNNGALPNKITEKTDANGVARIALTNTTDGVTVVTAEVEGQRQSVDTHFVKGTIAADKSTLAAVPTSIIADGLMASTITLELKDTYGDPQAGANVAFDTTLGNMGVITDHNDGTYSAPLTSTTLGVATVTVKVDGAAFSVPSVTVNFTADPIPDAGRSSFTVSTPDILADGTMSSTLSFVPVDKNGHFISGMQGLSFTQNGVPVSISPITEQPDSYTATVVGNSVGDVTITPQVDTLILSTLQKKISLFPVPTLTGILVNGQNFATDKGFPKTIFKNATFQLQMDNDVANNTQYEWSSSFTPNVSVNDQGQVTITYQTYSEVAVTAKSKKFPSYSVSYRFYPNRWIYDGGRSLVSSLEASRQCQGSDMSAVLESSRATNGTRAPDGTLWGEWGSLTAYSSDWQSGEYWVKKTSTDFETMNMDTGALQPGPAYLAFPLCALSI

111111110000000000000000000000000000000000000000000000000000000000000000000000000000000000000000000000000000000000000000000000000000000000000000000000000000000000000000000000000000000000000000000000000000000000000000000000000000000000000000000000000000000000000000000000000000000000000000000000000000000000000000000000000000000000000000000000000000000000000000000000000000000000000000000000000000000000000000000000000000000000000000000000000000000000000000000000000000000000000000000000000000

>DM_train234

LLAGASSQRSVARMDGDVIIGALFSVHHQPPAEKVPERKCGEIREQYGIQRVEAMFHTLDKINADPVLLPNITLGSEIRDSCWHSSVALEQSIEFIRDSLISIRDEKDGINRCLPDGQSLPPGRTKKPIAGVIGPGSSSVAIQVQNLLQLFDIPQIAYSATSIDLSDKTLYKYFLRVVPSDTLQARAMLDIVKRYNWTYVSAVHTEGNYGESGMDAFKELAAQEGLSIAHSDKIYSNAGEKSFDRLLRKLRERLPKARVVVCFCEGMTVRGLLSAMRRLGVVGEFSLIGSDGWADRDEVIEGYEVEANGGITIKLQSPEVRSFDDYFLKLRLDTNTRNPWFPEFWQHRFQCRLPGHLLENPNFKRICTGNESLEENYVQDSKMGFVINAIYAMAHGLQNMHHALCPGHVGLCDAMKPIDGSKLLDFLIKSSFIGVSGEEVWFDEKGDAPGRYDIMNLQYTEANRYDYVHVGTWHEGVLNIDDYKIQMNKSG

11111110000000000000000000000000000000000000000000000000000000000000000000000000000000000000000000001111111111111111111111111100000000000000000000000000000000000000000000000000000000000000000000000000000000000000000000000000000000000000000000000000000000000000000000000000000000000000000000000000000000000000000000000000000000000000000000000000000000000111111100000000000000000000000000000000000000000000000000000000000000000000000000000000000000000000000000000000000000000000000000111111111

>DM_train235

MKKRILAVAVTSMLLSASVFAQETVVPSRVGDLKFESDFPTQETMKNMLNEMDFQRATQAYLWGIPASSIMEWLNVSRNDFKFEEGQMGFFNTLKQKQGIITANFTTPYVIGTWNLEKTGPLIINLPEAKMAGMMLDVHQRVLSDLSLLGPDKGKGGKYLIVPPGEKYKDLNPKGYYVIRPKTNVVYGGIRILEPDVDRVVKQVVPNITTQPYADGKLGRKIPVAQVPEIDWTHIPKDGLEYWKTIHQIIQENPVEERDRFVMAQLKFLGIEKGKPFNPTEEQKKILLEASKVGRAMAQSNDYTKRFTQPYWKGTNWKDAISVSLDQRSENYDELDERAAWFYEAITVSRGMKSTIPGFGQRYLVTYQDSDGNWLSGEHTYKLHVPANVPASNFWSTTVYDENNRLMIINDAGSPDISSRKNLKVNSDGSIDVYYGPKPVKGYENNWVQTNPGEGWFTYFRFYGPTEKMFDKSWTMGDIELVKLEHHHHHH

11111111111111111111100000000000000000000000000000000000000000000000000000000000000000000000000000000000000000000000000000000000000000000000000000000000000000000000000000000000000000000000000000000000000000000000000000000000000000000000000000000000000000000000000000000000000000000000000000000000000000000000000000000000000000000000000000000000000000000000000000000000000000000000000000000000000000000000000000000000000000000000000000000000000000000000000000000000000000000000000000111111111

>DM_train236

RLTELREDIDAILEDPALEGAVSGVVVVDTATGEELYSRDGGEQLLPASNMKLFTAAAALEVLGADHSFGTEVAAESAPGRRGEVQDLYLVGRGDPTLSAEDLDAMAAEVAASGVRTVRGDLYADDTWFDSERLVDDWWPEDEPYAYSAQISALTVAHGERFDTGVTEVSVTPAAEGEPADVDLGAAEGYAELDNRAVTGAAGSANTLVIDRPVGTNTIAVTGSLPADAAPVTALRTVDEPAALAGHLFEEALESNGVTVKGDVGLGGVPADWQDAEVLADHTSAELSEILVPFMKFSNNGHAEMLVKSIGQETAGAGTWDAGLVGVEEALSGLGVDTAGLVLNDGSGLSRGNLVTADTVVDLLGQAGSAPWAQTWSASLPVAGESDPFVGGTLANRMRGTAAEGVVEAKTGTMSGVSALSGYVPGPEGELAFSIVNNGHSGPAPLAVQDAIAVRLAEYAGHQAPEGARMMRGPVQGSGELECSWVQAC

000000000000000000000000000000000000000000000000000000000000000000000000000000000000000000000000000000000000000000000000000000000000000000000000000000000000000000000000000000000000000000000000000000000000000000000000000000000000000000000000000000000000000000000000000000000000000000000000000000000000000000000000000000000000000000000000000000000000000000000000000000000000000000000000000000000000000000000000000000000000000000000000000000000000000000000000000000000011111111111111111111111

>DM_train237

MNSTNSGPPDSGSATGVVPTPDEIASLLQVEHLLDQRWPETRIDPSLTRISALMDLLGSPQRSYPSIHIAGTNGKTSVARMVDALVTALHRRTGRTTSPHLQSPVERISIDGKPISPAQYVATYREIEPLVALIDQQSQASAGKGGPAMSKFEVLTAMAFAAFADAPVDVAVVEVGMGGRWDATNVINAPVAVITPISIDHVDYLGADIAGIAGEKAGIITRAPDGSPDTVAVIGRQVPKVMEVLLAESVRADASVAREDSEFAVLRRQIAVGGQVLQLQGLGGVYSDIYLPLHGEHQAHNAVLALASVEAFFGAGAQRQLDGDAVRAGFAAVTSPGRLERMRSAPTVFIDAAHNPAGASALAQTLAHEFDFRFLVGVLSVLGDKDVDGILAALEPVFDSVVVTHNGSPRALDVEALALAAGERFGPDRVRTAENLRDAIDVATSLVDDAAADPDVAGDAFSRTGIVITGSVVTAGAARTLFGRDPQ

1111111111111111111110000000000000000011100000000000000000000000000000000000000000000000000000000000000000000000000000000000000000000000000001111100000000000000000000000000000000000000000000000000000000000000000000000000000011000000000000000000000000000000000000000000000000000000000000000000000000000000000000000001111000000000000000000000000000000000000000000000000000000000000000000000000000000000000000000000000000000000000000000000000000000000000000000011110000000000000000000000000

>DM_train239

MSQPDATPFDYILSGGTVIDGTNAPGRLADVGVRGDRIAAVGDLSASSARRRIDVAGKVVSPGFIDSHTHDDNYLLKHRDMTPKISQGVTTVVTGNCGISLAPLAHANPPAPLDLLDEGGSFRFARFSDYLEALRAAPPAVNAACMVGHSTLRAAVMPDLRREATADEIQAMQALADDALASGAIGISTGAFYPPAAHASTEEIIEVCRPLITHGGVYATHMRDEGEHIVQALEETFRIGRELDVPVVISHHKVMGKLNFGRSKETLALIEAAMASQDVSLDAYPYVAGSTMLKQDRVLLAGRTLITWCKPYPELSGRDLEEIAAERGKSKYDVVPELQPAGAIYFMMDEPDVQRILAFGPTMIGSDGLPHDERPHPRLWGTFPRVLGHYSRDLGLFPLETAVWKMTGLTAAKFGLAERGQVQPGYYADLVVFDPATVADSATFEHPTERAAGIHSVYVNGAAVWEDQSFTGQHAGRVLNRAGA

1111111000000000000000000000000000000000000000000000000000000000000000000000000000000000000000000000000000000000000000000000000000000000000000000000000000000000000000000000000000000000000000000000000000000000000000000000000000000000000000000000000000000000000000000000000000000000000000000000000000000000000000000000000000000000000000000000000000000000000000000000000000000000000000000000000000000000000000000000000000000000000000000000000000000000000000000000000000000000000000000111

>DM_train241

LLSRRRTRRPGEPPLDLGSIPWLGYALDFGKDAASFLTRMKEKHGDIFTILVGGRYVTVLLDPHSYDAVVWEPRTRLDFHAYAIFLMERIFDVQLPHYSPSDEKARMKLTLLHRELQALTEAMYTNLHAVLLGDATEAGSGWHEMGLLDFSYSFLLRAGYLTLYGIEALPRTHESQAQDRVHSADVFHTFRQLDRLLPKLARGSLSVGDKDHMCSVKSRLWKLLSPARLARRAHRSKWLESYLLHLEEMGVSEEMQARALVLQLWATQGNMGPAAFWLLLFLLKNPEALAAVRGELESILWQAEQPVSQTTTLPQKVLDSTPVLDSVLSESLRLTAAPFITREVVVDLAMPMADGREFNLRRGDRLLLFPFLSPQRDPEIYTDPEVFKYNRFLNPDGSEKKDFYKDGKRLKNYNMPWGAGHNHCLGRSYAVNSIKQFVFLVLVHLDLELINADVEIPEFDLSRYGFGLMQPEHDVPVRYRIRP

111110000000000000000000000000000000000000000000000000000000000000000000000000000000000000000000000000000000000000000000000000000000000000000000000000000000000000000000000000000000000000000000000000000000000000000000000000000000000000000000000000000000000000000000000000000000000000000000000000000000001111111111000000000000000000000000000000000000000000000000000000000000000000000000000000000000000000000000000000000000000000000000000000000000000000000000000000000000000000000000000

>DM_train242

MMNDGKQQSTFLFHDYETFGTHPALDRPAQFAAIRTDSEFNVIGEPEVFYCKPADDYLPQPGAVLITGITPQEARAKGENEAAFAARIHSLFTVPKTCILGYNNVRFDDEVTRNIFYRNFYDPYAWSWQHDNSRWDLLDVMRACYALRPEGINWPENDDGLPSFRLEHLTKANGIEHSNAHDAMADVYATIAMAKLVKTRQPRLFDYLFTHRNKHKLMALIDVPQMKPLVHVSGMFGAWRGNTSWVAPLAWHPENRNAVIMVDLAGDISPLLELDSDTLRERLYTAKTDLGDNAAVPVKLVHINKCPVLAQANTLRPEDADRLGINRQHCLDNLKILRENPQVREKVVAIFAEAEPFTPSDNVDAQLYNGFFSDADRAAMKIVLETEPRNLPALDITFVDKRIEKLLFNYRARNFPGTLDYAEQQRWLEHRRQVFTPEFLQGYADELQMLVQQYADDKEKVALLKALWQYADEIVEHHHHHH

11111110000000000000000000000000000000000000000000000000000000000000000000000000000000000000000000000000000000000000000000000000000000000000000000000000000011100000000000000000000000000000000000000000000000000000000000000000000000000000000000000000000000000000000000000000000000000000000000111100000000000000000000000000000000000000000000000000000000000011110000000000000000000000000000000000000000000000000000000000000000000000000000000000000000000000000000000000000000000000011111

>DM_train243

MSRLVVVSNRIAPPDEHAASAGGLAVGILGALKAAGGLWFGWSGETGNEDQPLKKVKKGNITWASFNLSEQDLDEYYNQFSNAVLWPAFHYRLDLVQFQRPAWDGYLRVNALLADKLLPLLQDDDIIWIHDYHLLPFAHELRKRGVNNRIGFFLHIPFPTPEIFNALPTYDTLLEQLCDYDLLGFQTENDRLAFLDCLSNLTRVTTRSAKSHTAWGKAFRTEVYPIGIEPKEIAKQAAGPLPPKLAQLKAELKNVQNIFSVERLDYSKGLPERFLAYEALLEKYPQHHGKIRYTQIAPTSRGDVQAYQDIRHQLENEAGRINGKYGQLGWTPLYYLNQHFDRKLLMKIFRYSDVGLVTPLRDGMNLVAKEYVAAQDPANPGVLVLSQFAGAANELTSALIVNPYDRDEVAAALDRALTMSLAERISRHAEMLDVIVKNDINHWQECFISDLKQIVPRSAESQQRDKVATFPKLALEHHHHHH

10000000000000111111000000000000000000000000000000000000000000000000000000000000000000000000000000000000000000000000000000000000000000000000000000000000000000000000000000000000000000000000000000000000000000000000000000000000000000000000000000000000000000000000000000000000000000000000000000000000000000000000000000000000000000000000000000000000000000000000000000000000000000000000000000000000000000000000000000000000000000000000000000000000000000000000000001111111111111111111111111

>DM_train244

TKFYTDAVEAVKDIPNGATVLVGGFGLCGIPENLIGALLKTGVKELTAVSNNAGVDNFGLGLLLQSKQIKRMISSYVGENAEFERQYLAGELEVELTPQGTLAERIRAGGAGVPAFYTSTGYGTLVQEGGSPIKYNKDGSIAIASKPREVREFNGQHFILEEAIRGDFALVKAWKADQAGNVTFRKSARNFNLPMCKAAETTVVEVEEIVDIGSFAPEDIHIPKIYVHRLVKGEKYEKRIERLSVRKEEDVKTRSGKLGDNVRERIIKRAALEFEDGMYANLGIGIPLLASNFISPNMTVHLQSENGILGLGPYPLQNEVDADLINAGKETVTVLPGASYFSSDESFAMIRGGHVNLTMLGAMQVSKYGDLANWMIPGKLVKGMGGAMDLVSSAKTKVVVTMEHSAKGNAHKIMEKCTLPLTGKQCVNRIITEKAVFDVDRKKGLTLIELWEGLTVDDIKKSTGCDFAVSPKLIPMQQVTT

0000000000000000000000000000000000000000000000000000000000000000000000000000000000000000000000000000000000000000000000000000000000000000000000000000000000000000000000000000000000000000000000000000000000000000000000000000000000000000000000000000000111111111111100000000000000000000000000000000000000000000000000000000000000000000000000000000000000000000000000000111111111111111111100000000000000000000011111111111111111111111100000000000000000000000000000000000000000000000000000000

>DM_train246

MARTGAEYIEALKTRPPNLWYKGEKVEDPTTHPVFRGIVRTMAALYDLQHDPRYREVLTYEEEGKRHGMSFLIPKTKEDLKRRGQAYKLWADQNLGMMGRSPDYLNAVVMAYAASADYFGEFAENVRNYYRYLRDQDLATTHALTNPQVNRARPPSGQPDPYIPVGVVKQTEKGIVVRGARMTATFPLADEVLIFPSTLLQAGSEKYALAFALPTSTPGLHFVCREALVGGDSPFDHPLSSRVEEMDCLVIFDDVLVPWERVFILGNVELCNNAYAATGALNHMAHQVVALKTAKTEAFLGVAALMAEGIGADVYGHVQEKIAEIIVYLEAMRAFWTRAEEEAKENAYGLLVPDRGALDGARNLYPRLYPRIREILEQIGASGLITLPSEKDFKGPLGPFLEKFLQGAALEAKERVALFRLAWDMTLSGFGARQELYERFFFGDPVRMYQTLYNVYNKEPYKERIRAFLKESLKVFEEVQA

1000000000000000000000000000000000000000000000000000000000000000000000000000000000000000000000000000000000000000000000000000000000000000000000000000000000000000000000000000000000000000000000000000000000000000000000000000000000000000000000000000000000000000000000000000000000000000000000000000000000000000000000000000000000000000000000000000000000000000000000000000000000000000000000000000000000000000000000000000000000000000000000000000000000000000000000000000000000000000000001111

>DM_train247

GSMGATKILMDSTHFNEIRSIIRSRSVAWDALARSEELSEIDASTAKALESILVKKNIGDGLSSSNNAHSGFKVNGKTLIPLIHLLSTSDNEDCKKSVQNLIAELLSSDKYGDDTVKFFQEDPKQLEQLFDVSLKGDFQTVLISGFNVVSLLVQNGLHNVKLVEKLLKNNNLINILQNIEQMDTCYVCIRLLQELAVIPEYRDVIWLHEKKFMPTLFKILQRATDSQLATRIVATNSNHLGIQLQYHSLLLIWLLTFNPVFANELVQKYLSDFLDLLKLVKITIKEKVSRLCISIILQCCSTRVKQHKKVIKQLLLLGNALPTVQSLSERKYSDEELRQDISNLKEILENEYQELTSFDEYVAELDSKLLCWSPPHVDNGFWSDNIDEFKKDNYKIFRQLIELLQAKVRNGDVNAKQEKIIIQVALNDITHVVELLPESIDVLDKTGGKADIMELLNHSDSRVKYEALKATQAIIGYTFK

111000000000000000000000000000000000000000000000000000011111111111111111000000000000000000000000000000000000000000000000000000000000000000000000000000000000000000000000000000000000000000000000000000000000000000000000000000000111111111111100000000000000000000000000000000000000000000000000000000000000000000000000000000000000000000000000000000000000000000000000000000000000000000000000000000000000000000000000000000000000000000000000000000000000000000000000000000000000000000000000

>DM_train249

GASETQQWKTLEDTRSALMGVYGLTRAALADNNTHWICGDLRKGDFTVYKRSDLQAVSDNELNKPYDLLKKVSNWRRFYAVINAASVFMEKAPRTVELDRSYSEQNLKYDIAQVRALRAFAYFYMVRIWGDVPLVTYSYDNGTFPSMPRTDAQTVLSYAKAELLTAIEDLPYQYGTQTNLYYGSYGAQWQGKLFNKLSAYSVLAHICAWQGNYAEAETYSAFIIDHASEINAKYTSIADLTSETGLFYSNASVKGSRILGFNFAHNDNEATQSGHLEQLTLAYPLVQKSYPEIYISKDSLFSIFTNFDDLRFGIIDTIKYSSYYVQNLNEETPVFSKIKIIQDGSAKDNDFGVFGSSIVFTRLEDITLLRAEALCALNRSTEAVSYLNMIRTNRGLREVSFKKDFGNNRESLIAEIFEERRRELMGEGWRWYDLVRRQKLMKDNEAFLRLISSGGIYWPVSEDIITANSQIEQNEFWK

1111000000000000000000000000000000000000000000000000000000000000000000000000000000000000000000000000000000000000000000000000000000000000000000000000000000000000000000000000000000000000000000000000000000000000000000000000000000000000000000000000000000000000000000001111111110000000000000000000000000000000000000000000000000000000000000000000001111111110000000000000000000000000000000000000000000000000000000000000000000000000000000000000000000000000000000000000000000000000000000

>DM_train250

ARTDNFKLSSLANGLKVATSNTPGHFSALGLYIDAGSRFEGRNLKGCTHILDRLAFKSTEHVEGRAMAETLELLGGNYQCTSSRENLMYQASVFNQDVGKMLQLMSETVRFPKITEQELQEQKLSAEYEIDEVWMKPELVLPELLHTAAYSGETLGSPLICPRGLIPSISKYYLLDYRNKFYTPENTVAAFVGVPHEKALELTGKYLGDWQSTHPPITKKVAQYTGGESCIPPAPVFGNLPELFHIQIGFEGLPIDHPDIYALATLQTLLGGGGSFSAGGPGKGMYSRLYTHVLNQYYFVENCVAFNHSYSDSGIFGISLSCIPQAAPQAVEVIAQQMYNTFANKDLRLTEDEVSRAKNQLKSSLLMNLESKLVELEDMGRQVLMHGRKIPVNEMISKIEDLKPDDISRVAEMIFTGNVNNAGNGKGRATVVMQGDRGSFGDVENVLKAYGLGNSSSSKNDSPKKKGWFHHHHHH

0000000000000000000000000000000000000000000000000000000000000000000000000000000000000000000000000000000000000000000000000000000000000000000000000000000000000000000000000000000000000000000000000000000000000000000000000000000000000000000000000000000000000000000000000000000000111111111100000000000000000000000000000000000000000000000000000000000000000000000000000000000000000000000000000000000000000000000000000000000000000000000000000000000000000000000000011111111111111111111

>DM_train251

GGNLEEMNIDPDNATQTHPKLLLTQICMNAFKRGTDGMYATKKVIQADGESADQYYKWTRGSFGYYDNLRNVQKMGEEAERVNAPVYTALTKFFRAYYFYELTLRFGDIPYSQALKGEKEEIYTPEYDAQEDVFAGILQELREADEILANDASVIDGDIIYNGNSTQWRKLINSFRLKVLMTLSNHTTVGNINIASEFKNIATNSPLMNSLADNGQLVYLDQQGNRYPQFNAQWSGYYMDDTFIQRMRERRDPRLFIFSAQTNKGKTEGKPIDDFSSYEGGDPAAPYSDAIIKVSEGTISPINDRFRTDPIVEPTMLMGYAELQQILAEAVVRGWISGNAQTYYEKGIRASFSFYETHAKDYAGYLNENAVAQYLKEPLVDFTQASGTEEQIERIIMQKYLVTFYQGNWDSFYEQLRTGYPDFRRPAGTEIPKRWMYPQGEYDNNGTNVETAITRQFGAGNDKINQATWWQKKS

111111111111111000000000000000000000000000000000000000000000000000000000000000000000000000000000000000000000000000001111110000000000000000000000000000000000000000000000000000000000000000000000000000000000000000000000000000000000000000000000000000000000000000000000000000000000000000000000000000011100000000000000000000000000000000000000000000000000000000000000000000000000000000000000000000000000000000000000000000000000000000000000000000000000000000000000000000000000000000

>DM_train252

GALRSLVLIGHGSHHHGESARATQQVAEALRGRGLAGHLPYDEVLEGYWQQEPGLRQVLRTVAYSDVTVVPVFLSEGYVTETVLPRELGLGHQGPVPTGGVVRVLGGRRVRYTRPLGAHPGMADAIAAQARDTLPEGTDPADVTLLLLAARPGNAALETHAQALRERGQFAGVEVVLESREALTPESHAASAVPLSEWPSRVEAGQAVLVPFLTHLGKHAAERLQQALAQAAERFPQAPPLHVGGPVGEHPAVAEVVLALAAEGREDERGGDIDQAHAEAWAALRHLAERGGRLGEVLLTPYGGLFELRHTLDEGRATLDLQTVVTPEGLRDLTARDEAGRWRPIRTWRTLPRGWRAVLSPADLRLGLELLYPAVIEESYAHEHRRLHWTPWMSTARRQTGTLARVQRATPDQVDTVAAQVCASCLRTRLWAGHTLGQTIFSGVPGGLPCAEACTVLLAAVRDEVGREAMGSGD

000000000000000000000000000000000000000000000000000000000000000000000000000000000000000000000000000000000000000000000000000000000000000000000000000000000000000000000000000000000001111111111110000000000000000000000000000000000000000000000000000000000000000000000000000000000000000000000000000000000000000000000000000000000000000000000000000000000000000000000000000000000000000000000000000000000000000000000000000000000000000000000000000000000000000000000000000000000000111111

>DM_train253

SKDVSSTITTVSASPDGTLNLPAAAPLSIASGRLNQTILETGSQFGGVARWGQESHEFGMRRLAGTALDGAMRDWFTNECESLGCKVKVDKIGNMFAVYPGKNGGKPTATGSHLDTQPEAGKYDGILGVLAGLEVLRTFKDNNYVPNYDVCVVVWFNAEGARFARSCTGSSVWSHDLSLEEAYGLMSVGEDKPESVYDSLKNIGYIGDTPASYKENEIDAHFELHIEQGPILEDENKAIGIVTGVQAYNWQKVTVHGVGAHAGTTPWRLRKDALLMSSKMIVAASEIAQRHNGLFTCGIIDAKPYSVNIIPGEVSFTLDFRHPSDDVLATMLKEAAAEFDRLIKINDGGALSYESETLQVSPAVNFHEVCIECVSRSAFAQFKKDQVRQIWSGAGHDSCQTAPHVPTSMIFIPSKDGLSHNYYEYSSPEEIENGFKVLLQAIINYDNYRVIRGHQFPGDDDDKHHHHHHHHSGD

111111111111111111111111110000000000000000000000000000000000000000000000000000000000000000000000000000000000000000000000000000000000000000000000000000000000000000000000000000000000000000000000000000000000000000000000000000000000000000000000000000000000000000000000000000000000000000000000000000000000000000000000000000000000000000000000000000000000000000000000000000000000000000000000000000000000000000000000000000000000000000000000000000000000000000000000011111111111111111

>DM_train254

MKTDTSTFLAQQIVRLRRRDQIRRLLQRDKTPLAILFMAAVVGTLTGLVGVAFEKAVSWVQNMRIGALVQVADHAFLLWPLAFILSALLAMVGYFLVRKFAPEAGGSGIPEIEGALEELRPVRWWRVLPVKFIGGMGTLGAGMVLGREGPTVQIGGNLGRMVLDVFRMRSAEARHTLLATGAAAGLSAAFNAPLAGILFIIEEMRPQFRYNLISIKAVFTGVIMSSIVFRIFNGEAPIIEVGKLSDAPVNTLWLYLILGIIFGVVGPVFNSLVLRTQDMFQRFHGGEIKKWVLMGGAIGGLCGILGLIEPAAAGGGFNLIPIAAAGNFSVGLLLFIFITRVVTTLLCFSSGAPGGIFAPMLALGTLLGTAFGMAAAVLFPQYHLEAGTFAIAGMGALMAASVRAPLTGIVLVLEMTDNYQLILPMIITCLGATLLAQFLGGKPLYSTILARTLAKQDAEQAEKNQNAPADENT

11111111111111111111111111111100000000000000000000000000000000000000000000000000000000000000000000000000000000000000000000000000000000000000000000000000000000000000000000000000000000000000000000000000000000000000000000000000000000000000000000000000000000000000000000000000000000000000000000000000000000000000000000000000000000000000000000000000000000000000000000000000000000000000000000000000000000000000000000000000000000000000000000000000000000000000000000001111111111111

>DM_train256

MGSSSSENLYFQGHMATESPATRRVQVAEHPRLLKLKEMFNSKFGSIPKFYVRAPGRVNIIGEHIDYCGYSVLPMAVEQDVLIAVEPVKTYALQLANTNPLYPDFSTSANNIQIDKTKPLWHNYFLCGLKGIQEHFGLSNLTGMNCLVDGNIPPSSGLSSSSALVCCAGLVTLTVLGRNLSKVELAEICAKSERYIGTEGGGMDQSISFLAEEGTAKLIEFSPLRATDVKLPSGAVFVIANSCVEMNKAATSHFNIRVMECRLAAKLLAKYKSLQWDKVLRLEEVQAKLGISLEEMLLVTEDALHPEPYNPEEICRCLGISLEELRTQILSPNTQDVLIFKLYQRAKHVYSEAARVLQFKKICEEAPENMVQLLGELMNQSHMSCRDMYECSCPELDQLVDICRKFGAQGSRLTGAGWGGCTVSMVPADKLPSFLANVHKAYYQRSDGSLAPEKQSLFATKPGGGALVLLEA

1111111111111110000000000000000000000000000000000000000000000000000000000000000000000000000000000000000000000001100000000000000000000000000000000000000000000000000000000000000000000000000000000000000000000000000000000000000000000000000000000000000000000000000000000000000000000000000000000000000000000000000000000000000000000000000000000000000000000000000000000000000000000000000000000000000000000000000000000000000000000000000000000000000000001111111110000000000000000000

>DM_train257

ATLDSWLSNEATVARTAILNNIGADGAWVSGADSGIVVASPSTDNPDYFYTWTRDSGLVLKTLVDLFRNGDTSLLSTIENYISAQAIVQGISNPSGDLSSGAGLGEPKFNVDETAYTGSWGRPQRDGPALRATAMIGFGQWLLDNGYTSTATDIVWPLVRNDLSYVAQYWNQTGYDLWEEVNGSSFFTIAVQHRALVEGSAFATAVGSSCSWCDSQAPEILCYLQSFWTGSFILANFDSSRSGKDANTLLGSIHTFDPEAACDDSTFQPCSPRALANHKEVVDSFRSIYTLNDGLSDSEAVAVGRYPEDTYYNGNPWFLCTLAAAEQLYDALYQWDKQGSLEVTDVSLDFFKALYSDAATGTYSSSSSTYSSIVDAVKTFADGFVSIVETHAASNGSMSEQYDKSDGEQLSARDLTWSYAALLTANNRRNSVVPASWGETSASSVPGTCAATSAIGTYSSVTVTSWPSIV

11111000000000000000000000000000000000000000000000000000000000000000000000000000000000000000000000000000000000000000000000000000000000000000000000000000000000000000000000000000000000000000000000000000000000000000000000000000000000000000000000000000000000000000000000000000000000000000000000000000000000000000000000000000000000000000000000000000000000000000000000000000000000000000000000000000000000000000000000000000000000000000000000000000000000000000000000000001111111

>DM_train258

MPEQYRYTLPVKAGEQRLLGELTGAACATLVAEIAERHAGPVVLIAPDMQNALRLHDEISQFTDQMVMNLADWETLPYDSFSPHQDIISSRLSTLYQLPTMQRGVLIVPVNTLMQRVCPHSFLHGHALVMKKGQRLSRDALRTQLDSAGYRHVDQVMEHGEYATRGALLDLFPMGSELPYRLDFFDDEIDSLRVFDVDSQRTLEEVEAINLLPAHEFPTDKAAIELFRSQWRDTFEVKRDPEHIYQQVSKGTLPAGIEYWQPLFFSEPLPPLFSYFPANTLLVNTGDLETSAERFQADTLARFENRGVDPMRPLLPPQSLWLRVDELFSELKNWPRVQLKTEHLPTKAANANLGFQKLPDLAVQAQQKAPLDALRKFLETFDGPVVFSVESEGRREALGELLARIKIAPQRIMRLDEASDRGRYLMIGAAEHGFVDTVRNLALICESDLLGERVARRRQDSRRTINPDTL

11110000000000000000000000000000000000000000000000000000000000000000000000000000000000000000000000000000000000000000000000000000000000000000000000000000000000000000000000000000000000000000000000000000000000000000000000000000000000000000000000000000000000000000000000000000000000000000000000000000000000000000000000000000000000000000000000000000000000000000000000000000000000000000000000000000000000000000000000000000000000000000000000000000000000000011111111111111111111

>DM_train259

MLSNELRQTLQKGLHDVNSDWTVPAAIINDPEVHDVERERIFGHAWVFLAHESEIPERGDYVVRYISEDQFIVCRDEGGEIRGHLNACRHRGMQVCRAEMGNTSHFRCPYHGWTYSNTGSLVGVPAGKDAYGNQLKKSDWNLRPMPNLASYKGLIFGSLDPHADSLEDYLGDLKFYLDIVLDRSDAGLQVVGAPQRWVIDANWKLGADNFVGDAYHTMMTHRSMVELGLAPPDPQFALYGEHIHTGHGHGLGIIGPPPGMPLPEFMGLPENIVEELERRLTPEQVEIFRPTAFIHGTVFPNLSIGNFLMGKDHLSAPTAFLTLRLWHPLGPDKMEVMSFFLVEKDAPDWFKDESYKSYLRTFGISGGFEQDDAENWRSITRVMGGQFAKTGELNYQMGRGVLEPDPNWTGPGEAYPLDYAEANQRNFLEYWMQLMLAESPLRDGNSNGSGTADASTPAAAKSKSPAKAEA

00000000000000000000000000000000000000000000000000000000000000000000000000000000000000000000000000000000000000000000000000000000000000000000000000000000000000000000000000000000000000000000000000000000000000000000000000000000000000000000000000000000000000000000000000000000000000000000000000000000000000000000000000000000000000000000000000000000000000000000000000000000000000000000000000000000000000000000000000000000000000000000000000000000011111111111111111111111111111

>DM_train260

GSPGIPMVAAHAAHSQSSAEWIACLDKRPLERSSEDVDIIFTRLKGVKAFEKFHPNLLRQICLCGYYENLEKGITLFRQGDIGTNWYAVLAGSLDVKVSETSSHQDAVTICTLGIGTAFGESILDNTPRHATIVTRESSELLRIEQEDFKALWEKYRQYMAGLLAPPYGVMETGSNNDRIPDKENVPSEKILRAGKILRIAILSRAPHMIRDRKYHLKTYRQCCVGTELVDWMIQQTSCVHSRTQAVGMWQVLLEDGVLNHVDQERHFQDKYLFYRFLDDEREDAPLPTEEEKKECDEELQDTMLLLSQMGPDAHMRMILRKPPGQRTVDDLEIIYDELLHIKALSHLSTTVKRELAGVLIFESHAKGGTVLFNQGEEGTSWYIILKGSVNVVIYGKGVVCTLHEGDDFGKLALVNDAPRAASIVLREDNCHFLRVDKEDFNRILRDVEANTVRLKEHDQDVLVLEKVP

1111111111111111110000000000000000000000000000000000000000000000000000000000000000000000000000000000000000000000000000000000000000000000000000000000000000000000000000000000011111111111100000000000000000000000000000000000000000000000000000000000000000000000000000000000000000000000000000000000000000000000000000000000000000000000000000000000000000000000000000000000000000000000000000000000000000000000000000000000000000000000000000000000000000000000000111111111111111111

>DM_train261

MRGSHHHHHHSNFAIILAAGKGTRMKSDLPKVLHKVAGISMLEHVFRSVGAIQPEKTVTVVGHKAELVEEVLAGQTEFVTQSEQLGTGHAVMMTEPILEGLSGHTLVIAGDTPLITGESLKNLIDFHINHKNVATILTAETDNPFGYGRIVRNDNAEVLRIVEQKDATDFEKQIKEINTGTYVFDNERLFEALKNINTNNAQGEYYITDVIGIFRETGEKVGAYTLKDFDESLGVNDRVALATAESVMRRRINHKHMVNGVSFVNPEATYIDIDVEIAPEVQIEANVILKGQTKIGAETVLTNGTYVVDSTIGAGAVITNSMIEESSVADGVTVGPYAHIRPNSSLGAQVHIGNFVEVKGSSIGENTKAGHLTYIGNCEVGSNVNFGAGTITVNYDGKNKYKTVIGDNVFVGSNSTIIAPVELGDNSLVGAGSTITKDVPADAIAIGRGRQINKDEYATRLPHHPKNQ

111111111100000000000000000000000000000000000000000000000000000000000000000000000000000000000000000000000000000000000000000000000000000000000000000000011111100000000000000000000000000000000000000000000000000000000000000000000000000000000000000000000000000000000000000000000000000000000000000000000000000000000000000000000000000000000000000000000000000000000000000000000000000000000000000000000000000000000000000000000000000000000000000000000000000000000000111111111111

>DM_train264

MEELNIDFDVFKKRIELLYSKYNEFEGSPNSLLFVLGSSNAENPYQKTTILHNWLLSYEFPATLIALVPGKVIIITSSAKAKHLQKAIDLFKDPESKITLELWQRNNKEPELNKKLFDDVIALINSAGKTVGIPEKDSYQGKFMTEWNPVWEAAVKENEFNVIDISLGLSKVWEVKDVNEQAFLSVSSKGSDKFMDLLSNEMVRAVDEELKITNAKLSDKIENKIDDVKFLKQLSPDLSALCPPNYKFNFDLLDWTYSPIIQSGKKFDLRVSARSTNDQLYGNGCILASCGIRYNNYCSNITRTFLIDPSEEMANNYDFLLTLQKEIVTNILKPGRTPKEVYESVIEYIEKTKPELVPNFTKNIGSLIGLEFRDSNFILNVKNDYRKIQRGDCFNISFGFNNLKDSQSANNYALQLADTVQIPLDETEPPRFLTNYTKAKSQISFYFNNEEEDNNKKKSSPATKV

111100000000000000000000000000000000000000000000000000000000000000000000000000000000000000000110000000000000000000000000000000000000000000000000000000000000000000000000000000000000000000000000000000000000000000000000000000000000000000000000000000000000000000000000000000000000000000000000000000000000000000000000000000000000000000000000000000000000000000000000000000000000000000000000000000000000000000000000000000000000000000000000000000000000000111111111111111111

>DM_train265

VSPKTYKDADFYVAPTQQDVNYDLVDDFGANGNDTSDDSNALQRAINAISRKPNGGTLLIPNGTYHFLGIQMKSNVHIRVESDVIIKPTWNGDGKNHRLFEVGVNNIVRNFSFQGLGNGFLVDFKDSRDKNLAVFKLGDVRNYKISNFTIDDNKTIFASILVDVTERNGRLHWSRNGIIERIKQNNALFGYGLIQTYGADNILFRNLHSEGGIALRMETDNLLMKNYKQGGIRNIFADNIRCSKGLAAVMFGPHFMKNGDVQVTNVSSVSCGSAVRSDSGFVELFSPTDEVHTRQSWKQAVESKLGRGCAQTPYARGNGGTRWAARVTQKDACLDKAKLEYGIEPGSFGTVKVFDVTARFGYNADLKQDQLDYFSTSNPMCKRVCLPTKEQWSKQGQIYIGPSLAAVIDTTPETSKYDYDVKTFNVKRINFPVNSHKTIDTNTESSRVCNYYGMSECSSSRWER

00000000000000000000000000000000000000000000000000000000000000000000000000000000000000000000000000000000000000000000000000000000000000000000000000000000000000000000000000000000000000000000000000000000000000000000000000000000000000000000000000000000000000000000000000000000000000000000011111111111111111111110000011111111111100000000000000000000000000000000000000000000000000000000000000000000000000000000000000000000000000000000000000000000000000000000000000000000

>DM_train266

MCYASMEMSAPIRFGTEGFRGVIAREFTFATLHRLAEAYGRHLLERGGGLVVVGHDTRFLADAFARALSGHLAGMGLKVVLLKGPVPTPLLSFAVRHLKAAGGAMLTASHNPPQYLGVKFKDATGGPIAQEEAKAIEALVPEEARALEGAYETLDLREAYFEALKAHLDLKALSGFSGVLYHDSMGGAGAGFLKGFLRHVGLEIPVRPIREEPHPLFHGVNPEPIPKNLGVTLAVLGPETPPSFAVATDGDADRVGVVLPGGVFFNPHQVLTTLALYRFRKGHRGRAVKNFAVTWLLDRLGERLGFGVTTTPVGFKWIKEEFLKGDCFIGGEESGGVGYPEHLPERDGILTSLLLLESVAATGKDLAEQFKEVEALTGLTHAYDRLDLPLKAPLDLTPFREPRPLAGLTPKGVDTLDGVKWLYEEAWVLFRASGTEPVVRIYVEAQSPELVRALLEEARKLVEG

11111110000000000000000000000000000000000000000000000000000000000000000000000000000000000000000000000000000000000000000000000000000000000000000000000000000000000000000000000000000000000000000000000000000000000000000000000000000000000000000000000000000000000000000000000000000000000000000000000000000000000000000000000000000000000000000000000000000000000000000000000000000000000000000000011111111111111100000000000000000000000000000001111100000000000000000000000000

>DM_train267

GRPANANSGFYVSGTTLYDANGNPFVMRGINHGHAWYKDQATTAIEGIANTGANTVRIVLSDGGQWTKDDIQTVRNLISLAEDNNLVAVLEVHDATGYDSIASLNRAVDYWIEMRSALIGKEDTVIINIANEWFGSWDGAAWADGYKQAIPRLRNAGLNNTLMIDAAGWGQFPQSIHDYGREVFNADPQRNTMFSIHMYEYAGGNASQVRTNIDRVLNQDLALVIGEFGHRHTNGDVDESTIMSYSEQRGVGWLAWSWKGNGPEWEYLDLSNDWAGNNLTAWGNTIVNGPYGLRETSKLSTVFTGGGSDGRTSPTTLYDFEESTQGWTGSSLSRGPWTVTEWSSKGNHSLKADIQMSSNSQHYLHVIQNRSLQQNSRIQATVKHANWGSVGNGMTARLYVKTGHGYTWYSGSFVPINGSSGTTLSLDLSNVQNLSQVREIGVQFQSESNSSGQTSIYIDNVIVE

00000110000000000000000000000000000000000000000000000000000000000000000000000000000000000000000000000000000000000000000000000000000000000000000000000000000000000000000000000000000000000000000000000000000000000000000000000000000000000000000000000000000000000000000000000000000000000000000000000000000000001111111110000000000000000000000000000000000000000000000000000000000000000000000001111111000000000000000000000000000000000000000000000000000000000000000000000000

>DM_train268

MGSDKIHHHHHHMFLGEDYLLTNRAAVRLFNEVKDLPIVDPHNHLDAKDIVENKPWNDIWEVEGATDHYVWELMRRCGVSEEYITGSRSNKEKWLALAKVFPRFVGNPTYEWIHLDLWRRFNIKKVISEETAEEIWEETKKKLPEMTPQKLLRDMKVEILCTTDDPVSTLEHHRKAKEAVEGVTILPTWRPDRAMNVDKEGWREYVEKMGERYGEDTSTLDGFLNALWKSHEHFKEHGCVASDHALLEPSVYYVDENRARAVHEKAFSGEKLTQDEINDYKAFMMVQFGKMNQETNWVTQLHIGALRDYRDSLFKTLGPDSGGDISTNFLRIAEGLRYFLNEFDGKLKIVLYVLDPTHLPTISTIARAFPNVYVGAPWWFNDSPFGMEMHLKYLASVDLLYNLAGMVTDSRKLLSFGSRTEMFRRVLSNVVGEMVEKGQIPIKEARELVKHVSYDGPKALFFG

1111111111110000000000000000000000000000000000000000000000000000000000000000000000000000000000000000000000000000000000000000000000000000000000000000000000000000000000000000000000000000000000000000000000000000000000000000000000000000000000000000000000000000000000000000000000000000000000000000000000000000000000000000000000000000000000000000000000000000000000000000000000000000000000000000000000000000000000000000000000000000000000000000000000000000000000000000000

>DM_train270

MELITILEKTVSPDRLELEAAQKFLERAAVENLPTFLVELSRVLANPGNSQVARVAAGLQIKNSLTSKDPDIKAQYQQRWLAIDANARREVKNYVLQTLGTETYRPSSASQCVAGIACAEIPVNQWPELIPQLVANVTNPNSTEHMKESTLEAIGYICQDIDPEQLQDKSNEILTAIIQGMRKEEPSNNVKLAATNALLNSLEFTKANFDKESERHFIMQVVCEATQCPDTRVRVAALQNLVKIMSLYYQYMETYMGPALFAITIEAMKSDIDEVALQGIEFWSNVCDEEMDLAIEASEAAEQGRPPEHTSKFYAKGALQYLVPILTQTLTKQDENDDDDDWNPCKAAGVCLMLLATCCEDDIVPHVLPFIKEHIKNPDWRYRDAAVMAFGCILEGPEPSQLKPLVIQAMPTLIELMKDPSVVVRDTAAWTVGRICELLPEAAINDVYLAPLLQCLIEGLSA

000000000000000000000000000000000000000000000000000000000000000000000000000000000000000000000000000000000000000000000000000000000000000000000000000000000000000000000000000000000000000000000000000000000000000000000000000000000000000000000000000000000000000000000000000000000000000000000000000000000000000000000000000000000000000000000000000000000000000000000000000000000000000000000000000000000000000000000000000000000000000000000000000000011111111111111111111111

>DM_train271

MAIVPFKRDPTFYPSPKMAMKAPPEDLAYVACLYTGTGINRADFIAVVDVNPKSETYSKIVHKVELPYINDELHHFGWNACSSALCPNGKPNIERRFLIVPGLRSSRIYIIDTKPNPREPKIIKVIEPEEVKKVSGYSRLHTVHCGPDAIYISALGNEEGEGPGGILMLDHYSFEPLGKWEIDRGDQYLAYDFWWNLPNEVLVSSEWAVPNTIEDGLKLEHLKDRYGNRIHFWDLRKRKRIHSLTLGEENRMALELRPLHDPTKLMGFINMVVSLKDLSSSIWLWFYEDGKWNAEKVIEIPAEPLEGNLPEILKPFKAVPPLVTDIDISLDDKFLYLSLWGIGEVRQYDISNPFKPVLTGKVKLGGIFHRADHPAGHKLTGAPQMLEISRDGRRVYVTNSLYSTWDNQFYPEGLKGWMVKLNANPSGGLEIDKEFFVDFGEARSHQVRLSGGDASSDSYCYP

111111100000000000000000000000000000000000000000000000000000000000000000000000000000000000000000000000000000000000000000000000000000000000000000000000000000000000000000000000000000000000000000000000000000000000000000000000000000000000000000000000000000000000000000000000000000000000000000000000000000000000000000000000000000000000000000000000000000000000000000000000000000000000000000000000000000000000000000000000000000000000000000000000000000000000000000000000

>DM_train274

MSLDIQSLDIQCEELSDARWAELLPLLQQCQVVRLDDCGLTEARCKDISSALRVNPALAELNLRSNELGDVGVHCVLQGLQTPSCKIQKLSLQNCCLTGAGCGVLSSTLRTLPTLQELHLSDNLLGDAGLQLLCEGLLDPQCRLEKLQLEYCSLSAASCEPLASVLRAKPDFKELTVSNNDINEAGVRVLCQGLKDSPCQLEALKLESCGVTSDNCRDLCGIVASKASLRELALGSNKLGDVGMAELCPGLLHPSSRLRTLWIWECGITAKGCGDLCRVLRAKESLKELSLAGNELGDEGARLLCETLLEPGCQLESLWVKSCSFTAACCSHFSSVLAQNRFLLELQISNNRLEDAGVRELCQGLGQPGSVLRVLWLADCDVSDSSCSSLAATLLANHSLRELDLSNNCLGDAGILQLVESVRQPGCLLEQLVLYDIYWSEEMEDRLQALEKDKPSLRVIS

10000000000000000000000000000000000000000000000000000000000000000000000000000000000000000000000000000000000000000000000000000000000000000000000000000000000000000000000000000000000000000000000000000000000000000000000000000000000000000000000000000000000000000000000000000000000000000000000000000000000000000000000000000000000000000000000000000000000000000000000000000000000000000000000000000000000000000000000000000000000000000000000000000000000000000000000000000

>DM_train275

MTQPELTPAQRTEVELLARGRADKSRVLRDLKLPETPEAAHALLLRLGVWDEARTPYADRLRAALNAVELPVPDFDPAEERLDLTHLPTFAIDDEGNQDPDDAVGVEDLGGGLTRLWVHVADVAALVAPDSPLDLEARARGATLYLPDRTIGMLPDELVAKAGLGLHEVSPALSICLDLDPDGNAEAVDVLLTRVKVQRLAYQEAQARLEAGEEPFVTLARLARASRRLREGEGALSIDLPEVRVKADETGASVFPLPKPEMRTVVQECMTLAGWGTAIFADDNEIPLPFATQDYPTREVAGDTLPAMWARRKTLARTRFQPSPGPHHGMGLDLYAQATSPMRRYLDLVVHQQLRAFLAGRDPLSSKVMAAHIAESQMNADATRQAERLSRRHHTLRFIAAQPERVWDAVVVDRRGAQATLLIPDLAFDVQVNTPAAPGTALQVQFADIDLPQMRVRARSV

11000000000000000000000000000000000000000000000000000000000000000000000000000000000000000000000000000000000000000000000000000000000000000000000000000000000000000000000000000000000000000000000000000000000000000000000000000000000000000000000000000000000000000000000000000000000000000000000000000000000000000000000000000000000000000000000000000000000000000000000000000000000000000000000000000000000000000000000000000000000000000000000000000000000000000000000000000

>DM_train276

FSYGAAIPQSTQEKQFSQEFRDGYSILKHYGGNGPYSERVSYGIARDPPTSCEVDQVIMVKRHGERYPSPSAGKDIEEALAKVYSINTTEYKGDLAFLNDWTYYVPNECYYNAETTSGPYAGLLDAYNHGNDYKARYGHLWNGETVVPFFSSGYGRVIETARKFGEGFFGYNYSTNAALNIISESEVMGADSLTPTCDTDNDQTTCDNLTYQLPQFKVAAARLNSQNPGMNLTASDVYNLMVMASFELNARPFSNWINAFTQDEWVSFGYVEDLNYYYCAGPGDKNMAAVGAVYANASLTLLNQGPKEAGSLFFNFAHDTNITPILAALGVLIPNEDLPLDRVAFGNPYSIGNIVPMGGHLTIERLSCQATALSDEGTYVRLVLNEAVLPFNDCTSGPGYSCPLANYTSILNKNLPDYTTTCNVSASYPQYLSFWWNYNTTTELNYRSSPIACQEGDAMD

1111111111111000000000000000000000000000000000000000000000000000000000000000000000000000000000000000000000000000000000000000000000000000000000000000000000000000000000000000000000000000000000000000000111100000000000000000000000000000000000000000000000000000000000000000000000000000000000000000000000000000000000000000000000000000000000000000000000000000000000000000000000000000000000000000000000000000000000000000000000000000000000000000000000000000000000000000

>DM_train279

LERGRDYEKNKVCKEFSHLGKEDFTSLSLVLYSRKFPSGTFEQVSQLVKEVVSLTEACCAEGADPDCYDTRTSALSAKSCESNSPFPVHPGTAECCTKEGLERKLCMAALKHQPQEFPTYVEPTNDEICEAFRKDPKEYANQFMWEYSTNYGQAPLSLLVSYTKSYLSMVGSCCTSASPTVCFLKERLQLKHLSLLTTLSNRVCSQYAAYGEKKSRLSNLIKLAQKVPTADLEDVLPLAEDITNILSKCCESASEDCMAKELPEHTVKLCDNLSTKNSKFEDCCQEKTAMDVFVCTYFMPAAQLPELPDVELPTNKDVCDPGNTKVMDKYTFELSRRTHLPEVFLSKVLEPTLKSLGECCDVEDSTTCFNAKGPLLKKELSSFIDKGQELCADYSENTFTEYKKKLAERLKAKLPDATPTELAKLVNKRSDFASNCCSINSPPLYCDSEIDAELKNIL

11111111111100000000000000000000000000000000000000000000000111111110000000000000000000000000000000111100000000000000000000000000000000000000000000000000000000000000000000000000000000000000000000000000000000000000000000000000000000000000000000000000000000000000000000000000000000000000000000000000000000000000000000000000000000000000000000000000000000000000000000000000000000000000000000000000000000000000000000000000000000000000000000000000000000000000000000

>DM_train280

MSLMLSHNPIVEPFALAHATIVTGDKAGTILRNMTIVVGADGRIEQVAPSIETSIPAEYHYLDGTGKIVMPGLINAHTHLFSQGKPLNPKLATPKGQRMVATFAHSPLGKPYMAATVKHNATTLLESGVTTIRTLGDVGYEVVTLRDQIDAGQILGPRILASGPLMAIPEGHGAPLIALTSGTPEEARTAVAQNLKAGVNAIKIAATGGVTDAQEIGEAGSPQMSVEQMRAICDEAHQYGVIVGAHAQSPEGVRRSLLAGVDTIEHGSVLDDELIGMFRHNPNALRGYSALIPTLSAGLPLTLLGQDVTGITDIQLENSKNVVGGMVSGARQAHEAGLMIGVGTDTGMTFVPQYATWRELELLVAYAGFSPAEALHAATAVNASILGVDAETGSLEVGKSADLLVLNANPLDDLRALEHPALVIAAGHPVWRPGPKRFADIDALLDEAYAEGHHHHHH

11111111000000000000000000000000000000000000000000000000000000000000000000000000001111111111111111111111111111100000000000000000000000000000000000000000000000000000000000000000000000000000000000000000000000000000000011111100000000000000000000000000000000000000000000000000000000000000000000000000000000000000000000000000000000000000000000000000000000000000000000000000000000000000000000000000000000000000000000000000000000000000000000000000000000000011111111

>DM_train282

MGSSGHIEGRHMGKNVLLLGSGFVAQPVIDTLAANDDINVTVACRTLANAQALAKPSGSKAISLDVTDDSALDKVLADNDVVISLIPYTFHPNVVKSAIRTKTDVVTSSYISPALRELEPEIVKAGITVMNEIGLDPGIDHLYAVKTIDEVHRAGGKLKSFLSYCGGLPAPEDSDNPLGYKFSWSSRGVLLALRNSAKYWKDGKIETVSSEDLMATAKPYFIYPGYAFVCYPNRDSTLFKDLYHIPEAETVIRGTLRYQGFPEFVKALVDMGMLKDDANEIFSKPIAWNEALKQYLGAKSTSKEDLIASIDSKATWKDDEDRERILSGFAWLGLFSDAKITPRGNALDTLCARLEELMQYEDNERDMVVLQHKFGIEWADGTTETRTSTLVDYGKVGGYSSMAATVGYPVAIATKFVLDGTIKGPGLLAPYSPEINDPIMKELKDKYGIYLKEKTVA

1111111111110000000000000000000000000000000000000000000000000000000000000000000000000000000000000000000000000000000000000000000000000000000000000000000000000000000000000000000000000000000000000000000000000000000000000000000000000000000000000000000000000000000000000000000000000000000000000000000000000000000000000000000000000000000000000000000000000000000000000000000000000000000000000000000000000000000000000000000000000000000000000000000000000000000000000

>DM_train283

MSQTTTNPHVAVLAFPFSTHAAPLLAVVRRLAAAAPHAVFSFFSTSQSNASIFHDSMHTMQCNIKSYDISDGVPEGYVFAGRPQEDIELFTRAAPESFRQGMVMAVAETGRPVSCLVADAFIWFAADMAAEMGVAWLPFWTAGPNSLSTHVYIDEIREKIGVSGIQGREDELLNFIPGMSKVRFRDLQEGIVFGNLNSLFSRMLHRMGQVLPKATAVFINSFEELDDSLTNDLKSKLKTYLNIGPFNLITPPPVVPNTTGCLQWLKERKPTSVVYISFGTVTTPPPAEVVALSEALEASRVPFIWSLRDKARVHLPEGFLEKTRGYGMVVPWAPQAEVLAHEAVGAFVTHCGWNSLWESVAGGVPLICRPFFGDQRLNGRMVEDVLEIGVRIEGGVFTKSGLMSCFDQILSQEKGKKLRENLRALRETADRAVGPKGSSTENFITLVDLVSKPKDV

111111000000000000000000000000000000000000000000000001111111000000000000000000000000000000000000000000000000000000000000000000000000000000000000000000000000000000000000000000000000000000000000000000000000000000000000000000000000000000000000000000000011111111100000000000000000000000000000000000000000000000000000000000000000000000000000000000000000000000000000000000000000000000000000000000000000000000000000000000000000000000000000000000000000000000000000

>DM_train284

MNYPAEPFRIKSVETVSMIPRDERLKKMQEAGYNTFLLNSKDIYIDLLTDSGTNAMSDKQWAGMMMGDEAYAGSENFYHLERTVQELFGFKHIVPTHQGRGAENLLSQLAIKPGQYVAGNMYFTTTRYHQEKNGAVFVDIVRDEAHDAGLNIAFKGDIDLKKLQKLIDEKGAENIAYICLAVTVNLAGGQPVSMANMRAVRELTEAHGIKVFYDATRCVENAYFIKEQEQGFENKSIAEIVHEMFSYADGCTMSGKKDCLVNIGGFLCMNDDEMFSSAKELVVVYEGMPSYGGLAGRDMEAMAIGLREAMQYEYIEHRVKQVRYLGDKLKAAGVPIVEPVGGHAVFLDARRFCEHLTQDEFPAQSLAASIYVETGVRSMERGIISAGRNNVTGEHHRPKLETVRLTIPRRVYTYAHMDVVADGIIKLYQHKEDIRGLKFIYEPKQLRFFTARFDYI

100000000000000000000000000000000000000000000000000000000000000000000000000000000000000000000000000000000000000000000000000000000000000000000000000000000000000000000000000000000000000000000000000000000000000000000000000000000000000000000000000000000000000000000000000000000000000000000000000000000000000000000000000000000000000000000000000000000000000000000000000000000000000000000000000000000000000000000000000000000000000000000000000000000000000000000000

>DM_train286

CFAKGTNVLMADGSIECIENIEVGNKVMGKDGRPREVIKLPRGRETMYSVVQKSQHRAHKSDSSREVPELLKFTCNATHELVVRTPRSVRRLSRTIKGVEYFEVITFEMGQKKAPDGRIVELVKEVSKSYPISEGPERANELVESYRKASNKAYFEWTIEARDLSLLGSHVRKATYQTYAPILYENDHFFDYMQKSKFHLTIEGPKVLAYLLGLWIGDGLSDRATFSVDSRDTSLMERVTEYAEKLNLCAEYKDRKEPQVAKTVNLYSKVVRGNGIRNNLNTENPLWDAIVGLGFLKDGVKNIPSFLSTDNIGTRETFLAGLIDSDGYVTDEHGIKATIKTIHTSVRDGLVSLARSLGLVVSVNAEPAKVDMNGTKHKISYAIYMSGGDVLLNVLSKCAGSKKFRPAPAAAFARECRGFYFELQELKEDDYYGITLSDDSDHQFLLANQVVVHN

0000000000000000000000000000000000000000000000000000000000000000000000000000000000000000000000000000000000000000000000000000000000000011111111111111111000000000000000000000000000000000000000000011111000000000000000000000000000000000000000000000000000000111111111111111111111111111000000000000000000000000000000000000000000000000000000000000000000000000000000000000000000000000000000000000000000000000000000000000000000000000000000000000000000000000000000

>DM_train287

GAPIHDPDFIGGIGKELIVDNASDVTSFYPSAFQEHLNFIPAPTTGSGCTRIPSFDMSATHYCYTHNVILSGCRDHSHSHQYLALGVLRTTATGRIFFSTLRSISLDDTQNRKSCSVSATPLGCDMLCSKVTETEEEDYNSAVPTLMAHGRLGFDGQYHEKDLDVTTLFEDWVANYPGVGGGSFIDGRVWFSVYGGLKPNSPSDTVQEGKYVIYKRYNDTCPDEQDYQIRMAKSSYKPGRFGGKRIQQAILSIKVSTSLGEDPVLTVPPNTVTLMGAEGRILTVGTSHFLYQRGSSYFSPALLYPMTVSNKTATLHSPYTFNAFTRPGSIPCQASARCPNSCVTGVYTDPYPLIFYRNHTLRGVFGTMLDSEQARLNPASAVFDSTSRSRITRVSSSSTKAAYTTSTCFKVVKTNKTYCLSIAEISNTLFGEFRIVPLLVEILKNDGVREARSG

0000000000000000000000000000000000000000000000000000000000000000000000000000000000000000000000000000000000000000000000000000000000000000000000000000000000000000000000000000000000000000000000000000000000000000000000000000000000000000000000000000000000000000000000000000000000000000000000000000000000000000000000000000000000000000000000000000000000000000000000000000000000000000000000000000000000000000000000000000000000000000000000000000000000000011111111

>DM_train289

LEDKDLRSIQEVRNLIESANKAQKELAAMSQQQIDTIVKAIADAGYGAREKLAKMAHEETGFGIWQDKVIKNVFASKHVYNYIKDMKTIGMLKEDNEKKVMEVAVPLGVVAGLIPSTNPTSTVIYKTLISIKAGNSIVFSPHPNALKAILETVRIISEAAEKAGCPKGAISCMTVPTIQGTDQLMKHKDTAVILATGGSAMVKAAYSSGTPAIGVGPGNGPAFIERSANIPRAVKHILDSKTFDNGTICASEQSVVVERVNKEAVIAEFRKQGAHFLSDAEAVQLGKFILRPNGSMNPAIVGKSVQHIANLAGLTVPADARVLIAEETKVGAKIPYSREKLAPILAFYTAETWQEACELSMDILYHEGAGHTLIIHSEDKEIIREFALKKPVSRLLVNTPGALGGIGATTNLVPALTLGCGAVGGSSSSDNIGPENLFNIRRIATGVLELEDIR

1000000000000000000000000000000000000000000000000000000000000000000000000000000000000000000000000000000000000000000000000000000000000000000000000000000000000000000000000000000000000000000000000000000000000000000000000000000000000000000000000000000000000000000000000000000000000000000000000000000000000000000000000000000000000000000000000000000000000000000000000000000000000000000000000000000000000000000000000000000000000000000000000000000000000000000000

>DM_train290

MASLPILTVLEQSQVSPPPDTLGDKSLQLTFFDFFWLRSPPINNLFFYELPITRSQFTETVVPNIKHSLSITLKHFYPFVGKLVVYPAPTKKPEICYVEGDSVAVTFAECNLDLNELTGNHPRNCDKFYDLVPILGESTRLSDCIKIPLFSVQVTLFPNQGIAIGITNHHCLGDASTRFCFLKAWTSIARSGNNDESFLANGTRPLYDRIIKYPMLDEAYLKRAKVESFNEDYVTQSLAGPSDKLRATFILTRAVINQLKDRVLAQLPTLEYVSSFTVACAYIWSCIAKSRNDKLQLFGFPIDRRARMKPPIPTAYFGNCVGGCAAIAKTNLLIGKEGFITAAKLIGENLHKTLTDYKDGVLKDDMESFNDLVSEGMPTTMTWVSGTPKLRFYDMDFGWGKPKKLETVSIDHNGAISINSCKESNEDLEIGVCISATQMEDFVHIFDDGLKAYL

1111100000000000000000000000000000000000000000000000000000000000000000000000000000000000000000000000000000000000000000000000000000000000000000000000000000000000000000000000000000000000000000000000000000000000000000000000000000000000000000000000000000000000000000000000000000000000000000000000000000000000000000000000000000000000000000000000000000000000000000000000111110000000000000000000000000000000000000000000000000000000000000000000000000000000001111

>DM_train291

MKTLSPAVITLLWRQDAAEFYFSRLSHLPWAMLLHSGYADHPYSRFDIVVAEPICTLTTFGKETVVSESEKRTTTTDDPLQVLQQVLDRADIRPTHNEDLPFQGGALGLFGYDLGRRFESLPEIAEQDIVLPDMAVGIYDWALIVDHQRHTVSLLSHNDVNARRAWLESQQFSPQEDFTLTSDWQSNMTREQYGEKFRQVQEYLHSGDCYQVNLAQRFHATYSGDEWQAFLQLNQANRAPFSAFLRLEQGAILSLSPERFILCDNSEIQTRPIKGTLPRLPDPQEDSKQAVKLANSAKDRAENLMIVDLMRNDIGRVAVAGSVKVPELFVVEPFPAVHHLVSTITAQLPEQLHASDLLRAAFPGGSITGAPKVRAMEIIDELEPQRRNAWCGSIGYLSFCGNMDTSITIRTLTAINGQIFCSAGGGIVADSQEEAEYQETFDKVNRILKQLEK

000000000000000000000000000000000000000000000000000000000000000000000000000000000000000000000000000000000000000000000000000000000000000000000000000000000000000000000000000000000000000000000000000000000000000000000000000000000000000000000000000000000000000000000000000000011111111111111111111111110000000000000000000000000000000111111111111110000000000000000000000000000000000000000000000000000000000000000000000000000000000000000000000000000000000000000

>DM_train293

MEALNQSQAATGAPVITDLKVVPVAGHDSMLLNLSGAHGPLFTRNILILTDSSGHVGVGEVPGGEGIRKTLEDARHLLINQSIGNYQSLLNKVRNAFADRDVGGRGLQTFDLRIAVHAVTAVESALLDLLGQHLQVPVAALLGEGQQRDAVEMLGYLFYVGDRNKTDLGYRSEHEADNEWFRLRNKEALTPESVVALAEAAYDRYGFKDFKLKGGVLRGEDEIAAVTALSERFPDARITLDPNGAWSLKEAVALCRDQHHVLAYAEDPCGAENGYSGREVMAEFRRSTGLRTATNMIATDWRQMGHAIQLQSVDIPLADPHFWTMQGSVRVAQMCNEWGLTWGSHSNNHFDISLAMFTHVAAAAPGNITAIDTHWIWQDGQRLTKEPLQIKGGLVEVPKKPGLGVELDWDALMKAHEVYKSMGLGARDDATAMRYLVSGWEFNNKRPCMVR

1111111111100000000000000000000000000000000000000000000000000000000000000000000000000000000000000011111111111100000000000000000000000000000000000000000000000000000000000000000000000000000000000000000000000000000000000000000000000000000000000000000000000000000000000000000000000000000000000000000000000000000000000000000000000000000000000000000000000000000000000000000000000000000000000000000000000000000000000000000000000011111111111111111111111111111

>DM_train294

IIQVSPAGSMDLLSQLEVERLKKTASSDLYQLYRNCSLAVLNSGSHTDNSKELLDKYKNFDITVMRRERGIKLELANPPEHAFVDGQIIKGIQEHLFSVLRDIVYVNMHLADSQRLNLTNATHITNLVFGILRNAGALIPGATPNLVVCWGGHSINEVEYQYTREVGHELGLRELNICTGCGPGAMEGPMKGAAVGHAKQRYSEYRYLGLTEPSIIAAEPPNPIVNELVIMPDIEKRLEAFVRMAHGIIIFPGGPGTAEELLYILGIMMHPENADQPMPIVLTGPKQSEAYFRSLDKFITDTLGEAARKHYSIAIDNPAEAARIMSNAMPLVRQHRKDKEDAYSFNWSLKIEPEFQLPFEPNHESMANLDLHLNQRPEVLAANLRRAFSGVVAGNVKAEGIREIERHGPFEMHGDPVLMKKMDQLLNDFVAQNRMKLPGGSAYEPCYKIVT

0000000000000000000000000000000000000000000000110000000000000000000000000000000000000000000000000000000000000011111111100000000000000000000000000000000000000000000000000000000000000000000000000000000000000000000000000000000000000000000000000000000000000000000000000000000000000000000000000000000000000000000000000000000000000000000000000000000000000000000000000000000000000000000000000000000000000000000000000000000000000000000000000000000000000000001

>DM_train295

MKTWPAPTAPTPVRATVTVPGSKSQTNRALVLAALAAAQGRGASTISGALRSRDTELMLDALQTLGLRVDGVGSELTVSGRIEPGPGARVDCGLAGTVLRFVPPLAALGSVPVTFDGDQQARGRPIAPLLDALRELGVAVDGTGLPFRVRGNGSLAGGTVAIDASASSQFVSGLLLSAASFTDGLTVQHTGSSLPSAPHIAMTAAMLRQAGVDIDDSTPNRWQVRPGPVAARRWDIEPDLTNAVAFLSAAVVSGGTVRITGWPRVSVQPADHILAILRQLNAVVIHADSSLEVRGPTGYDGFDVDLRAVGELTPSVAALAALASPGSVSRLSGIAHLRGHETDRLAALSTEINRLGGTCRETPDGLVITATPLRPGIWRAYADHRMAMAGAIIGLRVAGVEVDDIAATTKTLPEFPRLWAEMVGPGQGWGYPQPRSGQRARRATGQGSGG

000000000000000000000000000000000000000000000000000000000000000000000000000000000000000000000000000000000000000000000000000000000000000000000000000000000000000000000000000000000000000000000000000000000000000000000000000000000000000000000000000000000000000000000000000000000000000000000000000000000000000000000000000000000000000000000000000000000000000000000000000000000000000000000000000000000000000000000000000000000000000011111111111111111111111111

>DM_train296

RQGCEARRAHEHLIRLLLEQGKCPEDGWDESTLELFLHELAVMDSNNFLGNCGVGEREGRVASALVARRHYRFIHGIGRSGDISAVQPKAAGSSLLNKITNSLVLNVIKLAGVHSVASCFVVPMATGMSLTLCFLTLRHKRPKAKYIIWPRIDQKSCFKSMVTAGFEPVVIENVLEGDELRTDLKAVEAKIQELGPEHILCLHSTTACFAPRVPDRLEELAVICANYDIPHVVNNAYGLQSSKCMHLIQQGARVGRIDAFVQSLDKNFMVPVGGAIIAGFNEPFIQDISKMYPGRASASPSLDVLITLLSLGCSGYRKLLKERKEMFVYLSTQLKKLAEAHNERLLQTPHNPISLAMTLKTIDGHHDKAVTQLGSMLFTRQVSGARAVPLGNVQTVSGHTFRGFMSHADNYPCAYLNAAAAIGMKMQDVDLFIKRLDKCLNIVRKEQTRA

111100000000000000000000000000000000000000000000000000000000000000000000000000011111110000000000000000000000000000000000000000000000000000000000000000000000000000000000000000000000000000000000000000000000000000000000000000000000000000000000000000000000000000000000000000000000000000000000000000000000000000000000000000000000000000000000000000000000000000000000000000000000000000000000000000000000000000000000000000000000000000000000000000000000000001

>DM_train297

SKYVDRVIAEVEKKYADEPEFVQTVEEVLSSLGPVVDAHPEYEEVALLERMVIPERVIEFRVPWEDDNGKVHVNTGYRVQFNGAIGPYLGGLRFAPSVNLSIMKFLGFEQAFKDSLTTLPMGGAKGGSDFDPNGKSDREVMRFCQAFMTELYRHIGPDIDVPAGDLGVGAREIGYMYGQYRKIVGGFYNGVLTGKARSFGGSLVRPEATGYGSVYYVEAVMKHENDTLVGKTVALAGFGNVAWGAAKKLAELGAKAVTLSGPDGYIYDPEGITTEEKINYMLEMRASGRNKVQDYADKFGVQFFPGEKPWGQKVDIIMPCATQNDVDLEQAKKIVANNVKYYIEVANMPTTNEALRFLMQQPNMVVAPSKAVNAGGVLVVGFEMSQNSERLSWTAEEVDSKLHQVMTDIHDGSAAAAERYGLGYNLVAGANIVGFQKIADAMMAQGIAW

00000000000000000000000000000000000000000000000000000000000000000000000000000000000000000000000000000000000000000000000000000000000000000000000000000000000000000000000000000000000000000000000011111111111100000000000000000000000000000000000000000000000000000000000000000000000000000000000000000000000000000000000000000000000000000000000000000000000000000000000000000000000000000000000111111111100000000000000000000000000000000000000000000000000000000

>DM_train298

GAADPVVVPANMEPLTIEGNRFVTLCIMIRTTPWEVSRDVKLHPRDEVDWHTLEGVRALREAFATNNPNGRLTWGFTMNALEDGRKNYREIRDYVVECQKKYGDEVTYFPGYFPAMYLPRERVNREMSEAIEIISKMVGNGYRPQSIMGGFLSADNLRYLAEKENIHVAHAVIWSQHNIDGGGADGSPSYPFYPSTEHFCKPAQGKSDFIDCVNLDGWTMDFICARRSGQTGHGIDGYNSRRGVGPIETYKGWGLDLGHREVMHTEAIHFDKGLELNGFGWVANIWEAQMVHEFGKDLICDAMKMWVTGTKERWPDTHFVTFGEFGELWRKQYKSNDDWNYRFVERGSGLGDSYNNLEIKWFMNKEFRLALLRDWHTKNSPAYVIDFTRYDLQAHEPADPSPEKPAKDWSLINKINQKALRPQDKPVLIDKLEKEDQDLIRKYYPELLK

11111111111100000000000000000000000000000000000000000000000000000000000000000000000000000000000000000000000000000000000000000000000000000000000000000000000000000000000000000000001100000000000000000000000000000000000000000000000000000000000000000000000000000000000000000000000000000000000000000000000000000000000000000000000000000000000000000000000000000000000000000000000000000000000000000000000000000000000000000000000000000000000000000000000000001

>DM_train300

MGSDKIHHHHHHMTFEEFKDRLFALAKKNGVEVQISFLETREFSLRLANGDLDQYTDAGKFNVEIKVLKDGKTGTFRTQVLENPEKCFEEALSNLQVKDSEEKEYFFEGGKEYREMETYVGRFEKLSVKEKMDMAKKAHESAAKDERVVMVPTVMYKDMVIKKIITNTLGLDVESQMDGGFLFAMAIARDANPRSGSWYELARTPEDLNPEEIGKRAAEEAISLIGSKTIPSGKYPVLMRNTALLDLMEMFIPMISAENVQKNLSPLKGKLGEQVGNPAVSIKDLPYHPKGLSSTPFDDEGVPTTEKFVLENGVLKTFLHNLKTARKEGVEPTGNGFVGGIRPVNLMLMPGEKSFEELLKEMDRGVVITEVEGMHAGANSISGEFSLFAKGYWVENGEIAHGVEDITISGNFLDLLRKIVLVGNDVKVSQHTIAPSVLVEVLDVAGK

111111111111000000000000000000000000000000000000000000000000000000000000000000000000000000000000001111000000000000000000000000000000000000000000000000000000000000000000000000000000000000000000000000000000000000000000000000000000000000000000000000000000000000000000000000000000000000000000000000000000000000000000000000000000000000000000000000000000000000000000000000000000000000000000000000000000000000000000000000000000000000000000000000000000011

>DM_train301

ASMKETNQKPYKETYGISHITRHDMLQIPEQQKNEKYQVPEFDSSTIKNISSAKGLDVWDSWPLQNADGTVANYHGYHIVFALAGDPKNADDTSIYMFYQKVGETSIDSWKNAGRVFKDSDKFDANDSILKDQTQEWSGSATFTSDGKIRLFYTDFSGKHYGKQTLTTAQVNVSASDSSLNINGVEDYKSIFDGDGKTYQNVQQFIDEGNYSSGDNHTLRDPHYVEDKGHKYLVFEANTGTEDGYQGEESLFNKAYYGKSTSFFRQESQKLLQSDKKRTAELANGALGMIELNDDYTLKKVMKPLIASNTVTDEIERANVFKMNGKWYLFTDSRGSKMTIDGITSNDIYMLGYVSNSLTGPYKPLNKTGLVLKMDLDPNDVTFTYSHFAVPQAKGNNVVITSYMTNRGFYADKQSTFAPSFLLNIKGKKTSVVKDSILEQGQLTVNK

111111100000000000000000000000000000000000000000000000000000000000000000000000000000000000000000000000000000000000000000000000000000000000000000000000000000000000000000000000000000000000000000000000000000000000000000000000000000000000000000000000000000000000000000000000000000000000000000000000000000000000000000000000000000000000000000000000000000000000000000000000000000000000000000000000000000000000000000000000000000000000000000000000000000000

>DM_train302

MKGRLISSDPYRQQFLVERAVSFSHRQRDCSELISVLPRHALQQIDGFGGSFTEGAGVVFNSMSEKTKAQFLSLYFSAQEHNYTLARMPIQSCDFSLGNYAYVDSSADLQQGRLSFSRDEAHLIPLISGALRLNPHMKLMASPWSPPAFMKTNNDMNGGGKLRRECYADWADIIINYLLEYRRHGINVQALSVQNEPVAVKTWDSCLYSVEEETAFAVQYLRPRLARQGMDEMEIYIWDHDKDGLVDWAELAFADEANYKGINGLAFHWYTGDHFSQIQYLAQCLPDKKLLFSEGCVPMESDAGSQIRHWHTYLHDMIGNFKSGCSGFIDWNLLLNSEGGPNHQGNLCEAPIQYDAQNDVLRRNHSWYGIGHFCRYVRPGARVMLSSSYDNLLEEVGFVNPDGERVLVVYNRDVQERRCRVLDGDKEIALTLPPSGASTLLWRQESI

000000000000000000000000000000000000000000000000000000000000000000000000000000000000000000000000000000000000000000000000000000000000000000000000000000000000000000000000000000000000000000000000000000000000000000000000000000000000000000000000000000000000000000000000000000000000000000000000000000000000000000000000000000000000000000000000000000000000000000000000000000000000000000000000000000000000000000000000000000000000000000000000000000000000001

>DM_train304

MSDQPIIRRRQVKTGISDARANNAKTQSQYQPYKDAAWGFINHWYPALFTHELEEDQVQGIQICGVPIVLRRVNGKVFALKDQCLHRGVRLSEKPTCFTKSTISCWYHGFTFDLETGKLVTIVANPEDKLIGTTGVTTYPVHEVNGMIFVFVREDDFPDEDVPPLAHDLPFRFPERSEQFPHPLWPSSPSVLDDNAVVHGMHRTGFGNWRIACENGFDNAHILVHKDNTIVHAMDWVLPLGLLPTSDDCIAVVEDDDGPKGMMQWLFTDKWAPVLENQELGLKVEGLKGRHYRTSVVLPGVLMVENWPEEHVVQYEWYVPITDDTHEYWEILVRVCPTDEDRKKFQYRYDHMYKPLCLHGFNDSDLYAREAMQNFYYDGTGWDDEQLVATDISPITWRKLASRWNRGIAKPGRGVAGAVKDTSLIFKQTADGKRPGYKVEQIKEDH

11111111111111100000000000000000000000000000000000000000000000000000000000000000000000000000000000000000000000000000000000000000000000000000000000000000000000000000000000000000000000000000000000000000000000000000000000000000000000000000000000000000000000000000000000000000000000000000000000000000000000000000000000000000000000000000000000000000000000000000000000000000000000000000000000000000000000000000000000000000000000000000000000000000001111

>DM_train306

GKKAVINDSNTPLHLLQPAYQGTYGDLTPEQVKKDIDRVFAYIDKETPARVVDKNTGKVITDYTAMGDEAQLERGAFRLASYEWGVTYSALIAAAETTGDKRYTDYVQNRFRFLAEVAPHFKRVYEEKGKTDSQLLQILTPHALDDAGAVCTAMIKLRLKDESLPVDGLIQNYFDFIINKEYRLADGTFARNRPQRNTLWLDDMFMGIPAVAQMSRYDKEAKNKYLAEAVKQFLQFADRMFIPEKGLYRHGWVESSTDHPAFCWARANGWALLTACELLDVLPEDYPQRPKVMDYFRAHVRGVTALQSGEGFWHQLLDCNDSYLETSATAIYVYCLAHAINKGWIDAIAYGPVAQLGWHAVAGKINEEGQVEGTCVGTGMAFDPAFYYYRPVNVYAAHGYGPVLWAGAEMIRLLNTQHPQMNDSAVQYYQEKQKTTAPIFAVDSE

1111100000000000000000000000000000000000000000000000000000000000000000000000000000000000000000000000000000000000000000000000000000000000000000000000000000000000000000000000000000000000000000000000000000000000000000000000000000000000000000000000000000000000000000000000000000000000000000000000000000000000000000000000000000000000000000000000000000000000000000000000000000000000000000000000000000000000000000000000000000000000000000000000000000001

>DM_train311

MERVNVVGAGLAGSEAAWTLLRLGVPVRLFEMRPKRMTPAHGTDRFAEIVCSNSLGGEGETNAKGLLQAEMRRAGSLVMEAADLARVPAGGALAVDREEFSGYITERLTGHPLLEVVREEVREIPPGITVLATGPLTSEALAEALKRRFGDHFLAYYDAASPIVLYESIDLTKCFRAGRYGQSADYLNCPMTEEEYRRFHQALLEAQRHTPHDWEKLEFFEACVPVEELARRGYQTLLFGPMKPVGLVDPRTGKEPFAVVQLRQEDKAGRMWSLVGFQTGLKWPEQKRLIQMIPGLENAEIVRYGVMHRNTYLNAPRLLGETLEFREAEGLYAAGVLAGVEGYLESAATGFLAGLNAARKALGLPPVAPPEESMLGGLVRYLATANPEGFQPMYANWGLVPPVEGRMGKKEKRQAMYRRGLEAFSAWLSGLNPPLPRPEAALV

00000000000000000000000000000000000000000000000000000000000000000000000000000000000000000000000000000000000000000000000000000000000000000000000000000000000000000000000000000000000011111000000000000000000000000000111111100000000000000000000000000000000000000000000000000000000000000000000000000000000000000000000000000000000000000000000000000000000000000000000000000000000000000000000000000000000000000000000000000000000000000000000000001111111

>DM_train312

MRIDPFKLAHWMNARKYTAAQTADLAGLPLDDLRRLLGDEANEPDPAAATALAEALSVEPSQLAADAHRNLTVVHKSAEEMHASRRPIQRDGIHFYNYYTLAAPEGRVAPVVLDILCPSDRLPALNNGHLEPAITVNLGPGDINGRWGEEITPQTWRVLHANHGGDRWITGDSYVEPSYCPHSYSLAGDAPARIVSYTAQSNISPLMTEANNWSTGAFEEALKALSGKVSAGSVLDLFLARRAHTRTSAAEAAGVPPADLEAALRSPASETGLTVLRTLGRALGFDYRVLLPADDQHDGVGKTWTTIEDSRRSRRTFGTYEAASMASAAHLPDLVGSFLRVDADGRGADLIDHAENHYVVTEGRLTLEWDGPDGPASVELEPDGSAWTGPFVRHRWHGTGTVLKFGSGAHLGYQDWLELTNTFEPAATLRRGRRDLAGWGYDN

00000000000000000000000000000000000001111111100000000000000000000000000000000000000000000000000000000000000000000000000000000000000000000000000000000000000000000000000000000000000000000000000000000000000000000000000000000000000000000000000000000000000000000000000000000000000000000000000000000000110000000000000000000000000000000000000000000011111100000000000000000000000000000000000000000000000000000000000000000000000000000000000000111111111

>DM_train313

MWEANELSSTNTFSHQAEMDWPSANWWQRYQDAQLNHLIEEALQHSPSLCMAMARLKGAQGFARQAGAIRSFDLGLAASATESKVSERYQSATPPDGWNDYGTLTLNFQYDFDFWGKNRAAVVAATSELAAAEAESVAARLMISTSIANAYAELARLYANQETVHAALQVRNKTVELLEKRYANGLETLGSVSQAKAVAASVEAELLGIQESIQLQKNALAALVGQGPDRAASIEEPHITLTSRYGLPSEAGVGLLGHRADITAARWRAEAAAQQVGIAQAQFYPDVTLSAFIGYQAFGLDHLFDSGNDAGAIGPAIYLPLFTGGRLEGQLTSAEARYQEAVAQYNGTLVQALHEIADVVTSSQALQARINKTEQAVQQAEQALHIATNRYQGGLATYLDVLVAEESLLNNQRALVNLQSRAFSLDLALIHALGGGFETTES

1111111111111111111100000000000000000000000000000000000000000000000000000000000000000000000000000000000000000000000000000000000000000000000000000000000000000000000000000000000000000000000000000000000000000000000000000000000000000000000000000000011111110000000000000000000000000000000000000000000000000000000000000000000000000000000000000000000000000000000000000000000000000000000000000000000000000000000000000000000000000000000000000000001111

>DM_train315

AGTHDYSTALKDSIIFFDANKCGPQAGENNVFDWRGACHTTDGSDVGVDLTGGYHDAGDHVKFGLPQGYSAAILGWSLYEFKESFDATGNTTKMLQQLKYFTDYFLKSHPNSTTFYYQVGEGNADHTYWGAPEEQTGQRPSLYKADPSSPASDILSETSAALTLMYLNYKNIDSAYATKCLNAAKELYAMGKANQGVGNGQSFYQATSFGDDLAWAATWLYTATNDSTYITDAEQFITLGNTMNENKMQDKWTMCWDDMYVPAALRLAQITGKQIYKDAIEFNFNYWKTQVTTTPGGLKWLSNWGVLRYAAAESMVMLVYCKQNPDQSLLDLAKKQVDYILGDNPANMSYIIGYGSNWCIHPHHRAANGYTYANGDNAKPAKHLLTGALVGGPDQNDKFLDDANQYQYTEVALDYNAGLVGVLAGAIKFFGGTIVNPPVKK

000000000000000000000000000000000000000000000000000000000000000000000000000000000000000000000000000000000000000000000000000000000000000000000000000000000000000000000000000000000000000000000000000000000000000000000000000000000000000000000001111110000000000000000000000000000000000000000000000000000000000000000000000000000000000000000000000000000000000000000000000000000000000000000000000000000000000000000000000000000000000000000001111111111

>DM_train316

PYFDNISTIAYEGPASKNPLAFKFYNPEEKVGDKTMEEHLRFSVAYWHTFTGDGSDPFGAGNMIRPWNKYSGMDLAKARVEAAFEFFEKLNIPFFCFHDVDIAPEGETLKETYKNLDIIVDMIEEYMKTSKTKLLWNTANLFTHPRFVHGAATSCNADVFAYAAAKVKKGLEIAKRLGAENYVFWGGREGYETLLNTDMKLELDNLARFLHMAVDYAKEIGFDGQFLIEPKPKEPTKHQYDFDVATALAFLQTYGLKDYFKFNIEANHATLAGHTFEHELRVARIHGMLGSVDANQGDMLLGWDTDEFPTDLYSTTLAMYEILKNGGLGRGGLNFDAKVRRGSFEPEDLFYAHIAGMDSFAVGLKVAHRLIEDRVFDEFIEERYKSYTEGIGREIVEGTADFHKLEAHALQLGEIQNQSGRQERLKTLLNQYLLEVCAAR

00000000000000000000000000000000000000000000000000000000000000000000000000000000000000000000000000000000000000000000000000000000000000000000000000000000000000000000000000000000000000000000000000000000000000000000000000000000000000000000000000000000000000000000000000000000000000000000000000000000000000000000000000000000000000000000000000000000000000000000000000000000000000000000000000000000000000000000000000000000000000000000000000000111

>DM_train317

MLSTVFRRTMATGRHFIAVCQMTSDNDLEKNFQAAKNMIERAGEKKCEMVFLPECFDFIGLNKNEQIDLAMATDCEYMEKYRELARKHNIWLSLGGLHHKDPSDAAHPWNTHLIIDSDGVTRAEYNKLHLFDLEIPGKVRLMESEFSKAGTEMIPPVDTPIGRLGLSICYDVRFPELSLWNRKRGAQLLSFPSAFTLNTGLAHWETLLRARAIENQCYVVAAAQTGAHNPKRQSYGHSMVVDPWGAVVAQCSERVDMCFAEIDLSYVDTLREMQPVFSHRRSDLYTLHINEKSSETGGLKFARFNIPADHIFYSTPHSFVFVNLKPVTDGHVLVSPKRVVPRLTDLTDAETADLFIVAKKVQAMLEKHHNVTSTTICVQDGKDAGQTVPHVHIHILPRRAGDFGDNEIYQKLASHDKEPERKPRSNEQMAEEAVVYRNLM

11111111100000000000000000000000000000000000000000000000000000000000000000000000000000000000000000000000000000000000000000000000000000000000000000000000000000000000000000000000000000000000000000000000000000000000000000000000000000000000000000000000000000000000000000000000000000000000000000000000000000000000000000000000000000000000000000000000000000000000000000000000000000000000000000000000000000000011111111111111111111100000000000000000

>DM_train319

MPKLVTWMNNQRVGELTKLANGAHTFKYAPEWLASRYARPLSLSLPLQRGNITSDAVFNFFDNLLPDSPIVRDRIVKRYHAKSRQPFDLLSEIGRDSVGAVTLIPEDETVTHPIMAWEKLTEARLEEVLTAYKADIPLGMIREENDFRISVAGAQEKTALLRIGNDWCIPKGITPTTHIIKLPIGEIRQPNATLDLSQSVDNEYYCLLLAKELGLNVPDAEIIKAGNVRALAVERFDRRWNAERTVLLRLPQEDMCQTFGLPSSVKYESDGGPGIARIMAFLMGSSEALKDRYDFMKFQVFQWLIGATQGHAKNFSVFIQAGGSYRLTPFYDIISAFPVLGGTGIHISDLKLAMGLNASKGKKTAIDKIYPRHFLATAKVLRFPEVQMHEILSDFARMIPAALDNVKTSLPTDFPENVVTAVESNVLRLHGRLSREYGSK

10000000000000000000000000000000000000000000000000000000000000000000000000000000000000000000000000000000000001111000000000000000000000111111111110000000000000000000000000000000000000001111111111100000000000000000000000000000000000000000000000000000000000000000000000000000000000000000000000000000000000000000000000000000000000000000000000000000000000000000000000000000000000000000000000000000000000000000000000000000000000000000000000000111

>DM_train320

MKPLSSPLQQYWQTVVERLPEPLAEESLSAQAKSVLTFSDFVQDSVIAHPEWLTELESQPPQADEWQHYAAWLQEALCNVSDEAGLMRELRLFRRRIMVRIAWAQTLALVTEESILQQLSYLAETLIVAARDWLYDACCREWGTPCNAQGEAQPLLILGMGKLGGGELNFSSDIDLIFAWPEHGCTQGGRRELDNAQFFTRMGQRLIKVLDQPTQDGFVYRVDMRLRPFGESGPLVLSFAALEDYYQEQGRDWERYAMVKARIMGDSEGVYANELRAMLRPFVFRRYIDFSVIQSLRNMKGMIAREVRRRGLTDNIKLGAGGIREIEFIVQVFQLIRGGREPSLQSRSLLPTLSAIAELHLLSENDAEQLRVAYLFLRRLENLLQSINDEQTQTLPSDELNRARLAWAMDFADWPQLTGALTAHMTNVRRVFNELIGDDE

00000000000000000000000000000000000000000000000000000000000000000000000000000000000000000000000000000000000000000000000000000000000000000000000000000000000000000000000000000000000000111111110000000000000000000000000000000000000000000000000000000000000000000000000000000000000000000000000000000000000000000000000000000000000000000000000000000000000000000000000000000000000000000000000000000000000000000000000000000000000000000000000000000111

>DM_train322

DIAVVGIGYVGLVSATCFAELGANVRCIDTDRNKIEQLNSGTIPIYEPGLEKMIARNVKAGRLRFGTEIEQAVPEADIIFIAVGTPAGEDGSADMSYVLDAARSIGRAMSRYILIVTKSTVPVGSYRLIRKAIQEELDKREVLIDFDIASNPEFLKEGNAIDDFMKPDRVVVGVDSDRARELITSLYKPMLLNNFRVLFMDIASAEMTKYAANAMLATRISFMNDVANLCERVGADVSMVRLGIGSDSRIGSKFLYPGCGYGGSCFPKDVKALIRTAEDNGYRMEVLEAVERVNEKQKSILFDKFSTYYKGNVQGRCVAIWGLSFKPGTDDMREAPSLVLIEKLLEVGCRVRVYDPVAMKEAQKRLGDKVEYTTDMYDAVRGAEALFHVTEWKEFRMPDWSALSQAMAASLVIDGRNVYELPADSDFTLLNIGNSAIES

0000000000000000000000000000000000000000111100000000000000000000000000000000000000000001100000000000000000000000000000000000000000000000000000000000000000000000000000000000000000000000000000111110000000000000000000000000000000000000000000000000000000000000000000000000000000000000000000000000000000000000000000000000000000000000000000000000000000000000000000000000000000000000000000000000000000000000000000000000000000000011100000000111111

>DM_train323

GRDAPAPAASQPSGCGKHNSPERKVYMDYNATTPLEPEVIQAMTKAMWEAWGNPSSPYSAGRKAKDIINAARESLAKMIGGKPQDIIFTSGGTESNNLVIHSVVKHFHANQTSKGHTGGHHSPVKGAKPHFITSSVEHDSIRLPLEHLVEEQVAAVTFVPVSKVSGQTEVDDILAAVRPTTRLVTIMLANNETGIVMPVPEISQRIKALNQERVAAGLPPILVHTDAAQALGKQRVDVEDLGVDFLTIVGHKFYGPRIGALYIRGLGEFTPLYPMLFGGGQERNFRPGTENTPMIAGLGKAAELVTQNCEAYEAHMRDVRDYLEERLEAEFGQKRIHLNSQFPGTQRLPNTCNFSIRGPRLQGHVVLAQCRVLMASVGAACHSDHGDQPSPVLLSYGVPFDVARNALRLSVGRSTTRAEVDLVVQDLKQAVAQLEDQA

111111111111111111111110000000000000000000000000000000000000000000000000000000000000000000000000000000000000000001111111111110000000000000000000000000000000000000000000000000000000000000000000000000000000000000000000000000000000000000000000000000000000000000000000000000000000000000000000000000000000000000000000000000000000000000000000000000000000000000000000000000000000000000111111111000000000000000000000000000000000000000000000000011

>DM_train324

MAAGTFTAQNKVRPGVYINFKSEPQAAGTLGERGIVSMPLILSWGEPGKMITIEAGDDVFPKLGYSIMDAQLRLINEALKRAKTLLLYRLNAGTKAAVTVGNLTVTAKWGGARGNDITLVIQENIDDETKFDVSTLVDGAELDKQTVSDIAGLAANDWVIFSGTGALTETAGAPLINGSDGAVTNQAYIDYLAAVEIFDFNTIALPSTDDALKATFTAFAKRLRDDEGKKIQVVLENYPAADYEGVISVKNGVVLADGTILTAAQATAWVAGATAGARVNESLTYQGYDEAVDVAPRYTNAQIIAALQAGEFLFTASDNQALVEQDINTLTSFTADKGKQFAKNRVIRVLDGINNDFVRIFSKFYIGKVSNNADGRNLLKSECINYMNTLQDIDAIKNFDGQTDLTVQSGNDVDAVYIEAYAWPVDSIEKIYVRVRIK

111111111111110000000000011110000000000000000000000000000000000000000000000000000000000000000000000000000000000000000000000000000000000000000000000000000000000000000000000000000000000000000000000000000000000000000000000000000000000000000000000000000000000000000000000000000000000000000000000000000000000000000000000000000000000000000000000000000000000000000000000001111000000000000000000000000000000000000000011000000000000000000000000000

>DM_train325

GAMDPSQYASSSSWTSFLKSIASFNGDLSSLSAPPFILSPISLTEFSQYWAEHPELFLEPSFINDDNYKEHCLIDPEVESPELARMLAVTKWFISTLKSQYCSRNESLGSEKKPLNPFLGELFVGKWENKEHPEFGETVLLSEQVSHHPPVTAFSIFNDKNKVKLQGYNQIKASFTKSLMLTVKQFGHTMLDIKDESYLVTPPPLHIEGILVASPFVELEGKSYIQSSTGLLCVIEFSGRGYFSGKKNSFKARIYKDSKDSKDKEKALYTISGQWSGSSKIIKANKKEESRLFYDAARIPAEHLNVKPLEEQHPLESRKAWYDVAGAIKLGDFNLIAKTKTELEETQRELRKEEEAKGISWQRRWFKDFDYSVTPEEGALVPEKDDTFLKLASALNLSTKNAPSGTLVGDKEDRKEDLSSIHWRFQRELWDEEKEIVL

110000000000000000000000000000000000000000000000000000000000000000000000000000000000000000000000000000000000000000000000000000000000000000000000000000000000000000000000000000000000000000000000000000000000000000000000000000000000000000000000000000000000000000000000000000000000000000000000000000000000000000000000000000000000000000000000000000000000000000000000000000000000000000000000000000000000000000000000000000000000000000000000000000

>DM_train326

STLHGISHIFSYERLSLKRVVWALCFMGSLALLALVCTNRIQYYFLYPHVTKLDEVAATRLTFPAVTFCNLNEFRFSRVTKNDLYHAGELLALLNNRYEIPDTQTADEKQLEILQDKANFRNFKPKPFNMLEFYDRAGHDIREMLLSCFFRGEQCSPEDFKVVFTRYGKCYTFNAGQDGKPRLITMKGGTGNGLEIMLDIQQDEYLPVWGETDETSFEAGIKVQIHSQDEPPLIDQLGFGVAPGFQTFVSCQEQRLIYLPPPWGDCKATTGDSEFYDTYSITACRIDCETRYLVENCNCRMVHMPGDAPYCTPEQYKECADPALDFLVEKDNEYCVCEMPCNVTRYGKELSMVKIPSKASAKYLAKKYNKSEQYIGENILVLDIFFEALNYETIEQKKAYEVAGLLGDIGGQMGLFIGASILTVLELFDYAYEVIKHR

111111111111111100000000000000000000000000000000000000000000000000000000000000000000000000000000000000000000000000000000000000000000000000000000000000000000000000000000000000000000000000000000000000000000000000000000000000000000000000000000000000000000000000000000000000000000000000000000000000000000000000000000000000000000000000000000000000000000000000000000000000000000000000000000000000000000000000000000000000000000000000001111111111

>DM_train327

MSLPGTGSGTSSGGGNTQGQDVYIIPRPFSNFGKKLSTYTKSHKFMIFGLANNVIGPTGTGTTAVNRLLTTCLAEIPWQKLPLYMNQSEFDLLPPGSRVVECNVKVIFRTNRIAFETSSTVTKQATLNQISNVQTAIGLNKLGWGINRAFTAFQSDQPMIPTATTAPKYEPVTGDTGYRGMIADYYGADSTNDTAFGNAGNYPHHQVSSFTFLQNYYCMYQQTNQGTGGWPCLAEHLQQFDSKTVNNQCLIDVTYKPKMGLIKSPLNYKIIGQPTVKGTISVGDNLVNMRGAVVTNPPEATQNVAESTHNLTRNFPADLFNIYSDIEKSQVLHKGPWGHENPQIQPSVHIGIQAVPALTTGALLINSSPLNSWTDSMGYIDVMSSCTVMEAQPTHFPFSTEANTNPGNTIYRINLTPNSLTSAFNGLYGNGATLGNV

11111111111111111111100000000000000000000000000000000000000000000000000000000000000000000000000000000000000000000000000000000000000000000000000000000000000000000000000000000000000000000000000000000000000000000000000000000000000000000000000000000000000000000000000000000000000000000000000000000000000000000000000000000000000000000000000000000000000000000000000000000000000000000000000000000000000000000000000000000000000000000000000000000

>DM_train328

ADYQGKNVVIIGLGLTGLSCVDFFLARGVTPRVMDTRMTPPGLDKLPEAVERHTGSLNDEWLMAADLIVASPGIALAHPSLSAAADAGIEIVGDIELFCREAQAPIVAITGSNGKSTVTTLVGEMAKAAGVNVGVGGNIGLPALMLLDDECELYVLELSSFQLETTSSLQAVAATILNVTEDHMDRYPFGLQQYRAAKLRIYENAKVCVVNADDALTMPIRGADERCVSFGVNMGDYHLNHQQGETWLRVKGEKVLNVKEMKLSGQHNYTNALAALALADAAGLPRASSLKALTTFTGLPHRFEVVLEHNGVRWINDSKATNVGSTEAALNGLHVDGTLHLLLGGDGKSADFSPLARYLNGDNVRLYCFGRDGAQLAALRPEVAEQTETMEQAMRLLAPRVQPGDMVLLSPACASLDQFKNFEQRGNEFARLAKELG

00000000000000000000000000000000000000000000000000000000000000000000000000000000000000000000000000000000000000000000000000000000000000000000000000000000000000000000000000000000000000111111000000000000000000000000000000000000000000000000000000000000000000000000000000000000000000000000000000000000000000000000000000000000000000000000000000000000000000000000000000000000000000000000000000000000000000000000000000000000000000000000000000000

>DM_train329

LNSVGQGEFGGAPFKRFLRGTRIVSGGKLKRMTREKAKQVTVAGVPMPRDAEPRHLLVNGATGTGKSVLLRELAYTGLLRGDRMVIVDPNGDMLSKFGRDKDIILNPYDQRTKGWSFFNEIRNDYDWQRYALSVVPRGKTDEAEEWASYGRLLLRETAKKLALIGTPSMRELFHWTTIATFDDLRGFLEGTLAESLFAGSNEASKALTSARFVLSDKLPEHVTMPDGDFSIRSWLEDPNGGNLFITWREDMGPALRPLISAWVDVVCTSILSLPEEPKRRLWLFIDELASLEKLASLADALTKGRKAGLRVVAGLQSTSQLDDVYGVKEAQTLRASFRSLVVLGGSRTDPKTNEDMSLSLGEHEVERDRYSKNTGKHHSTGRALERVRERVVMPAEIANLPDLTAYVGFAGNRPIAKVPLEIKQFANRQPAFVEGTI

11111110000000000000000000000000000000000000000000000000000000000000000000000000000000000000000000000000000000000000000000000000000000000000000000000000000000000000000000000000000000000000000000000000000000000000000000000000000000000000000000000000000000000000000000000000000000000000000000000000000000000000000000000000000000000000000000000000000000000000000000000000111111111111111100000000000000000000000000000000000000000000000000111

>DM_train330

MASSSSFTYYCPPSSSPVWSEPLYSLRPEHARERLQDDSVETVTSIEQAKVEEKIQEVFSSYKFNHLVPRLVLQREKHFHYLKRGLRQLTDAYECLDASRPWLCYWILHSLELLDEPIPQIVATDVCQFLELCQSPDGGFGGGPGQYPHLAPTYAAVNALCIIGTEEAYNVINREKLLQYLYSLKQPDGSFLMHVGGEVDVRSAYCAASVASLTNIITPDLFEGTAEWIARCQNWEGGIGGVPGMEAHGGYTFCGLAALVILKKERSLNLKSLLQWVTSRQMRFEGGFQGRCNKLVDGCYSFWQAGLLPLLHRALHAQGDPALSMSHWMFHQQALQEYILMCCQCPAGGLLDKPGKSRDFYHTCYCLSGLSIAQHFGSGAMLHDVVMGVPENVLQPTHPVYNIGPDKVIQATTHFLQKPVPGFEECEDAVTSDPATD

11111111111111111111100000000000000000000000000000000000000000000000000000000000000000000000000000000000000000000000000000000000000000000000000000000000000000000000000000000000000000000000000000000000000000000000000000000000000000000000000000000000000000000000000000000000000000000000000000000000000000000000000000000000000000000000000000000000000000000000000000000000000000000000000000000000000000000000000000000000000001111111111111111

>DM_train331

MAGGLSQLVAYGAQDVYLTGNPQITFFKTVYRRYTNFAIESIQQTINGSVGFGNKVSTQISRNGDLITDIVVEFVLTKGGNGGTTYYPAEELLQDVELEIGGQRIDKHYNDWFRTYDALFRMNDDRYNYRRMTDWVNNELVGAQKRFYVPLIFFFNQTPGLALPLIALQYHEVKLYFTLASQVQGVNYNGSSAIAGAAQPTMSVWVDYIFLDTQERTRFAQLPHEYLIEQLQFTGSETATPSATTQASQNIRLNFNHPTKYLAWNFNNPTNYGQYTALANIPGACSGAGTAAATVTTPDYGNTGTYNEQLAVLDSAKIQLNGQDRFATRKGSYFNKVQPYQSIGGVTPAGVYLYSFALKPAGRQPSGTCNFSRIDNATLSLTYKTCSIDATSPAAVLGNTETVTANTATLLTALNIYAKNYNVLRIMSGMGGLAYAN

11111111111111111111111100000000000000000000000000000000000000000000000000000000000000000000000000000000000000000000000000000000000000000000000000000000000000000000000000000000000000000000000000000000000000000000000000000000000000000000000000000000000000000000000000000000000000000000000000000000000000000000000000000000000000000000000000000000000000000000000000000000000000000000000000000000000000000000000000000000000000000000000000000

>DM_train332

MSLSINSREVLAEKVKNAVNNQPVTDMHTHLFSPNFGEILLWDIDELLTYHYLVAEVMRWTDVSIEAFWAMSKREQADLIWEELFIKRSPVSEACRGVLTCLQGLGLDPATRDLQVYREYFAKKTSEEQVDTVLQLANVSDVVMTNDPFDDNERISWLEGKQPDSRFHAALRLDPLLNEYEQTKHRLRDWGYKVNDEWNEGSIQEVKRFLTDWIERMDPVYMAVSLPPTFSFPEESNRGRIIRDCLLPVAEKHNIPFAMMIGVKKRVHPALGDAGDFVGKASMDGVEHLLREYPNNKFLVTMLSRENQHELVVLARKFSNLMIFGCWWFMNNPEIINEMTRMRMEMLGTSFIPQHSDARVLEQLIYKWHHSKSIIAEVLIDKYDDILQAGWEVTEEEIKRDVADLFSRNFWRFVGRNDHVTSVKVEQQTEGHHHHHH

11000000000000000000000000000000000000000000000000000000000000000000000000000000000000000000000000000000000000000000000000000000000000000000000000000000000000000000000000000000000000000000000000000000000000000000000000000000000000000000000000000000000000000000000000000000000000000000000000000000000000000000000000000000000000000000000000000000000000000000000000000000000000000000000000000000000000000000000000000000111111111111111111111

>DM_train333

MLLAQKPFWQRHLAYPHINLDTVAHSLRLTGPLDTTLLLRALHLTVSEIDLFRARFSAQGELYWHPFSPPIDYQDLSIHLEAEPLAWRQIEQDLQRSSTLIDAPITSHQVYRLSHSEHLIYTRAHHIVLDGYGMMLFEQRLSQHYQSLLSGQTPTAAFKPYQSYLEEEAAYLTSHRYWQDKQFWQGYLREAPDLTLTSATYDPQLSHAVSLSYTLNSQLNHLLLKLANANQIGWPDALVALCALYLESAEPDAPWLWLPFMNRWGSVAANVPGLMVNSLPLLRLSAQQTSLGNYLKQSGQAIRSLYLHGRYRIEQIEQDQGLNAEQSYFMSPFINILPFESPHFADCQTELKVLASGSAEGINFTFRGSPQHELCLDITADLASYPQSHWQSHCERFPRFFEQLLARFQQVEQDVARLLAEPAALAATTSTRAIAS

0000000000000000000000000000000000000000000000000000000000000000000000000000000000000000000000000000000000000000000000000000000000000000000000000000000000000000000000000000000000000000000000000000000000000000000000000000000000000000000000000000000000000000000000000000000000000000000000000000000000000000000000000000000000000000000000000000000000000000000000000000000000000000000000000000000000000000000000000000000000000000111111111111

>DM_train334

MSASQKEGKLSTATISVDGKSAEMPVLSGTLGPDVIDIRKLPAQLGVFTFDPGYGETAACNSKITFIDGDKGVLLHRGYPIAQLAENASYEEVIYLLLNGELPNKAQYDTFTNTLTNHTLLHEQIRNFFNGFRRDAHPMAILCGTVGALSAFYPDANDIAIPANRDLAAMRLIAKIPTIAAWAYKYTQGEAFIYPRNDLNYAENFLSMMFARMSEPYKVNPVLARAMNRILILHADHEQNASTSTVRLAGSTGANPFACIAAGIAALWGPAHGGANEAVLKMLARIGKKENIPAFIAQVKDKNSGVKLMGFGHRVYKNFDPRAKIMQQTCHEVLTELGIKDDPLLDLAVELEKIALSDDYFVQRKLYPNVDFYSGIILKAMGIPTSMFTVLFAVARTTGWVSQWKEMIEEPGQRISRPRQLYIGAPQRDYVPLAKR

1111111111000000000000000000000000000000000000000000000000000000000000000000000000000000000000000000000000000000000000000000000000000000000000000000000000111111111000000000000000000000000000000000000000000000000000000000000000000000000000000000000000000000000000000000000000000000000000000000000000000000000000000000000000000000000000000000000000000000000000000000000000000000000000000000000000000000000000000000000000000000000000000000

>DM_train336

YTPVEEKENGRMIVIVAKKYEGDIKDFVDWKNQRGLRTEVKVAEDIASPVTANAIQQFVKQEYEKEGNDLTYVLLVGDHKDIPAKITPGIKSDQVYGQIVGNDHYNEVFIGRFSCESKEDLKTQIDRTIHYERNITTEDKWLGQALCIASAEGGPSADNGESDIQHENVIANLLTQYGYTKIIKCYDPGVTPKNIIDAFNGGISLVNYTGHGSETAWGTSHFGTTHVKQLTNSNQLPFIFDVACVNGDFLFSMPCFAEALMRAQKDGKPTGTVAIIASTIDQYWAPPMRGQDEMNEILCEKHPNNIKRTFGGVTMNGMFAMVEKYKKDGENMLDTWTVFGDPSLLVRTLVPTEMQVTAPANISASAQTFEVACDYNGAIATLSDDGDMVGTAIVKDGKAIIKLNESIADETNLTLTVVGYNKVTVIKDVKVEGTS

000000000000000000000000000000000000000000000000000000000000000000000000000000000000000000000000000000000000000000000000000000000000000000000000000000000000000000000000000000000000000000000000000000000000000000000000000000000000000000000000000000000000000000000000000000000000000000000000000000000000000000000000000000000000000000000000000000000000000000000000000000000000000000000000000000000000000000000000000000000000000000000000111

>DM_train337

SLMAGLPNSSNALQQWHHLFEAEGTKRSPQAQQHLQQLLRTGLPTRKHENWKYTPLEGLINSQFVSIAGEISPQQRDALALTLDSVRLVFVDGRYVPALSDATEGSGYEVSINDDRQGLPDAIQAEVFLHLTESLAQSVTHIAVKRGQRPAKPLLLMHITQGVAGEEVNTAHYRHHLDLAEGAEATVIEHFVSLNDARHFTGARFTINVAANAHLQHIKLAFENPLSHHFAHNDLLLAEDATAFSHSFLLGGAVLRHNTSTQLNGENSTLRINSLAMPVKNEVCDTRTWLEHNKGFCNSRQLHKTIVSDKGRAVFNGLINVAQHAIKTDGQMTNNNLLMGKLAEVDTKPQLEIYADDVKCSHGATVGRIDDEQIFYLRSRGINQQDAQQMIIYAFAAELTEALRDEGLKQQVLARIGQRLPGGAREGGSHHHHHH

111111111100000000000011100000000000000000000000000000000000000000000000000000000000000000000000000000000000000000000000000000000000000000000000000000000000000000000000000000000000000000000000000000000000000000000000000000000000000000000000000000000000000000000000000000000000000000000000000000000000000000000000000000000000000000000000000000000000000000000000000000000000000000000000000000000000000000000000000000000000000111111111111

>DM_train338

ERTSIAVHALMGLPTGQPANGTKLDSIGLPKVDGMSFTLYRVNEIDLTTQAGWDAASKIKLEELYTNGHPTDKVTKVATKKTEGGVAKFDNLTPALYLVVQELNGAEAVVRSQPFLVAAPQTNPTGDGWLQDVHVYPKHQALSEPVKTAVDPDATQPGFSVGENVKYRVATKIPEIASNTKFEGFTVADKLPAELGKPDTNKITVTLGGKPINSTDVSVQTYQVGDRTVLSVQLAGATLQSLDQHKDQELVVEFEAPVTKQPENGQLDNQAWVLPSNPTAQWDPEESGDAALRGMPSSRVSSKFGQITIEKSFDGNTPGADRTATFQLHRCEADGSLVKSDPPISLDGKQEFVTGQDGKAVLSGIHLGTLQLESNVMKYTDAWAGKGTEFCLVETATASGYELLPKPVIVKLEANESTNVLVEQKVKIDNKKKN

10000000000000001111111111100000000000000000000000000000000000000000000000000000000000000000000000000000000000000000000000000000000000000000000000000000000000000000000000000000000000000000000000000000000000000000000000000000000000000000000000000000000000000000000000000000000000000000000000000000000000000000000000000000000000000000000000000000000000000000000000000000000000000000000000000000000000000000000000000000000000000000000011

>DM_train339

MTQDVREPPALKYGIVLDAGSSHTSMFVYKWPADKENDTGIVGQHSSCDVQGGGISSYANDPSKAGQSLVRCLEQALRDVPRDRHASTPLYLGATAGMRLLNLTSPEATARVLEAVTQTLTQYPFDFRGARILSGQDEGVFGWVTANYLLENFIKYGWVGRWIRPRKGTLGAMDLGGASTQITFETTSPSEDPGNEVHLRLYGQHYRVYTHSFLCYGRDQILLRLLASALQIHRFHPCWPKGYSTQVLLQEVYQSPCTMGQRPRAFNGSAIVSLSGTSNATLCRDLVSRLFNISSCPFSQCSFNGVFQPPVAGNFIAFSAFYYTVDFLTTVMGLPVGTLKQLEEATEITCNQTWTELQARVPGQKTRLADYCAVAMFIHQLLSRGYHFDERSFREVVFQKKAADTAVGWALGYMLNLTNLIPADLPGLRKGTHF

11111111000000000000000000000000000000000000000000000000000000000000000000000000000000000000000000000000000000000000000000000000000000000000000000000000000000000000000000000000000000000000000000000000000000000000000000000000000000000000000000000000000000000000011111110000000000000000000000000000000000000000000000000000000000000000000000000000000000000000000000000000000000000000000000000000000000000000000000000000000000000000000000

>DM_train343

MSNVFYRSSKPYPVAVRGEGVFLYDDAGRRYLDGSSGALVANIGHGRAEVGERMAAQAARLPFVHGSQFSSDVLEEYAGRLARFVGLPTFRFWAVSGGSEATESAVKLARQYHVERGEPGRFKVITRVPSYHGASLGSLAASGMGARRELYTPLMRPEAWPKLPKPDPARNGAEDAEGLRALLEREGPETVAAFMAEPVVGASDAALAPAPGYYERVRDICDEAGIIFIADEVMSGMGRCGSPLALSRWSGVTPDIAVLGKGLAAGYAPLAGLLAAPQVYETVMGGSGAFMHGFTYAGHPVSVAAGLSVLDIVEREDLTGAAKERGAQLLAGLQALQARFPQMMQVRGTGLLLGVVLGDLATGQAFETPGIASRIGAAALKRGLITYPGSGAEPNGRGDHLLLGPPLSITAAEVDGLLALLAGALEDVLG

1100000000000000000000000000000000000000000000000000000000000000000000000000000000000000000000000000000000000000000000000000000000111111111111111111111111100000000000000000000000000000000000000000000000000000000000000000000000000000000000000000000000000000000000000000000000000000000000000000000000000000000000000000000000000000000000000000000000000000000000111111111111000000000000000001111111111100000000000000000000000000000001

>DM_train344

MAEFRIAQDVVARENDRRASALKEDYEALGANLARRGVDIEAVTAKVEKFFVAVPSWGVGTGGTRFARFPGTGEPRGIFDKLDDCAVIQQLTRATPNVSLHIPWDKADPKELKARGDALGLGFDAMNSNTFSDAPGQAHSYKYGSLSHTNAATRAQAVEHNLECIEIGKAIGSKALTVWIGDGSNFPGQSNFTRAFERYLSAMAEIYKGLPDDWKLFSEHKMYEPAFYSTVVQDWGTNYLIAQTLGPKAQCLVDLGHHAPNTNIEMIVARLIQFGKLGGFHFNDSKYGDDDLDAGAIEPYRLFLVFNELVDAEARGVKGFHPAHMIDQKHNVTDPIESLINSANEIRRAYAQALLVDRAALSGYQEDNDALMATETLKRAYRTDVEPILAEARRRTGGAVDPVATYRASGYRARVAAERPASVAGGGGII

1110000000000000000000000000000000000000000000000000000000000000000000000000000000000000000000000000000000000000000000000000000000000000000000000000000000000000000000000000000000000000000000000000000000000000000000000000000000000000000000000000000000000000000000000000000000000000000000000000000000000000000000000000000000000000000000000000000000000000000000000000000000000000000000000000000000000000000000000000000000000111111111

>DM_train345

MTTQTTPAHIAMFSIAAHGHVNPSLEVIRELVARGHRVTYAIPPVFADKVAATGPRPVLYHSTLPGPDADPEAWGSTLLDNVEPFLNDAIQALPQLADAYADDIPDLVLHDITSYPARVLARRWGVPAVSLSPNLVAWKGYEEEVAEPMWREPRQTERGRAYYARFEAWLKENGITEHPDTFASHPPRSLVLIPKALQPHADRVDEDVYTFVGACQGDRAEEGGWQRPAGAEKVVLVSLGSAFTKQPAFYRECVRAFGNLPGWHLVLQIGRKVTPAELGELPDNVEVHDWVPQLAILRQADLFVTHAGAGGSQEGLATATPMIAVPQAVDQFGNADMLQGLGVARKLATEEATADLLRETALALVDDPEVARRLRRIQAEMAQEGGTRRAADLIEAELPARHERQEPVGDRPNVGDRPAGVRSDRQRSAL

1111111000000000000000000000000000000000000000000000000000000000000000000000000000000000000000000000000000000000000000000000000000000000000000000000000000000000000000000000000000000000000000000000000000000000000000001111100000000000000000000000100000000000000000000000001110000000000000000000000000000000000000000000000000000000000000000000000000000000000000000000000000000000000000000000000000000000111111111111111111111111111111

>DM_train346

MNVLVIGRGGREHAIAWKAAQSPLVGKLYVAPGNPGIADVAELVHIDELDIEALVQFAKQQAIDLTIVGPEAPLASGIVDRFMAEGLRIFGPSQRAALIEGSKAFAKELMKKYGIPTADHAAFTSYEEAKAYIEQKGAPIVIKADGLAAGKGVTVAQTVEEALAAAKAALVDGQFGTAGSQVVIEEYLEGEEFSFMAFVNGEKVYPLAIAQDHKRAYDGDEGPNTGGMGAYSPVPQISDEMMDAALEAILRPAAKALAAEGRPFLGVLYAGLMATANGPKVIEFNARFGDPEAQVVLPRLKTDLVEAVLAVMDGKELELEWTDEAVLGVVLAAKGYPGAYERGAEIRGLDRISPDALLFHAGTKREGGAWYTNGGRVLLLAAKGETLAKAKEKAYEQLAAIDCDGLFYRRDIGRRAIERASAAYTRMKGR

0000000000000000000000000000000000000000000000000000000000000000000000000000000000000000000000000000000000000000000000000000000000000000000000000011110000000000000000000000000000000000000000000000000000000000000000000000000000000000000000000000000000000000000000000000000000000000000000000000000000000000000000000000000000000000000000000000000000000000000000000000000000000000000000000000000000000000000000000000000001111111111111

>DM_train347

MGSDKIHHHHHHMFDPESLKKLAIEIVKKSIEAVFPDRAVKETLPKLNLDRVILVAVGKAAWRMAKAAYEVLGKKIRKGVVVTKYGHSEGPIDDFEIYEAGHPVPDENTIKTTRRVLELVDQLNENDTVLFLLSGGGSSLFELPLEGVSLEEIQKLTSALLKSGASIEEINTVRKHLSQVKGGRFAERVFPAKVVALVLSDVLGDRLDVIASGPAWPDSSTSEDALKVLEKYGIETSESVKRAILQETPKHLSNVEIHLIGNVQKVCDEAKSLAKEKGFNAEIITTSLDCEAREAGRFIASIMKEVKFKDRPLKKPAALIFGGETVVHVKGNGIGGRNQELALSAAIALEGIEGVILCSAGTDGTDGPTDAAGGIVDGSTAKTLKAMGEDPYQYLKNNDSYNALKKSGALLITGPTGTNVNDLIIGLIV

111111111111111000000000000000000000000000000000000000000000000000000000000000000000000000000000000000000000000000000000000000000000000000000000000000000000000000000000000000000000000000000000000000000000000000000000000000000000000000000000000000000000000000000000000000000000000000000000000000000000000000000000000000000000000000000000000000000000000000000000000000000000000000000000000000000000000000000000000000000000000000000

>DM_train348

MAAVVLENGVLSRKLSDFGQETSYIEDNSNQNGAISLIFSLKEEVGALAKVLRLFEENDINLTHIESRPSRLNKDEYEFFTYLDKRTKPVLGSIIKSLRNDIGATVHELSRDKEKNTVPWFPRTIQELDRFANQILSYGAELDADHPGFKDPVYRARRKQFADIAYNYRHGQPIPRVEYTEEEKQTWGTVFRTLKALYKTHACYEHNHIFPLLEKYCGFREDNIPQLEDVSQFLQTCTGFRLRPVAGLLSSRDFLGGLAFRVFHCTQYIRHGSKPMYTPEPDICHELLGHVPLFSDRSFAQFSQEIGLASLGAPDEYIEKLATIYWFTVEFGLCKEGDSIKAYGAGLLSSFGELQYCLSDKPKLLPLELEKTACQEYSVTEFQPLYYVAESFSDAKEKVRTFAATIPRPFSVRYDPYTQRVEVLDNTQQ

111111111111111111000000000000000000000000000000000000000000000000000000000000000000000000000000000000000000000000000000000000000000000011111100000000000000000000000000000000000000000000000000000000000000000000000000000000000000000000000000000000000000000000000000000000000000000000000000000000000000000000000000000000000000000000000000000000000000000000000000000000000000000000000000000000000000000000000000000000000000000000000

>DM_train349

SQFDILCKTPPKVLVRQFVERFERPSGEKIASCAAELTYLCWMITHNGTAIKRATFMSYNTIISNSLSFDIVNKSLQFKYKTQKATILEASLKKLIPAWEFTIIPYNGQKHQSDITDIVSSLQLQFESSEEADKGNSHSKKMLKALLSEGESIWEITEKILNSFEYTSRFTKTKTLYQFLFLATFINCGRFSDIKNVDPKSFKLVQNKYLGVIIQCLVTETKTSVSRHIYFFSARGRIDPLVYLDEFLRNSEPVLKRVNRTGNSSSNKQEYQLLKDNLVRSYNKALKKNAPYPIFAIKNGPKSHIGRHLMTSFLSMKGLTELTNVVGNFSDKRASAVARTTYTHQITAIPDHYFALVSRYYAYDPISKEMIALKDETNPIEEWQHIEQLKGSAEGSIRYPAWNGIISQEVLDYLSSYINRRIGHHHHHH

000000000000000000000000000000000000000000000000000000000000000000000000000000000000000000000000000000000000011100000000000000001111110000000000000000000000000000000000000000000000000000000000000000000000000000000000000000000000000000000000000000000000000000000000000000000000000000000000000000000000000000000000000000000000000000001111111111110000000000000000000000000000000000000000000000000000000000000000000000000000011111111

>DM_train350

MTDFYSLIPSAPKGRFDGIERAHTAEDVKRLRGSVEIKYSLAEMGANRLWKLIHEEDFVNALGALSGNQAMQMVRAGLKAIYLSGWQVAADANTASAMYPDQSLYPANAGPELAKRINRTLQRADQIETAEGKGLSVDTWFAPIVADAEAGFGDPLDAFEIMKAYIEAGAAGVHFEDQLASEKKCGHLGGKVLIPTAAHIRNLNAARLAADVMGTPTLIVARTDAEAAKLLTSDIDERDQPFVDYEAGRTAEGFYQVKNGIEPCIARAIAYAPYCDLIWMETSKPDLAQARRFAEAVHKAHPGKLLAYNCSPSFNWKKNLDDATIAKFQRELGAMGYKFQFITLAGFHQLNYGMFELARGYKDRQMAAYSELQQAEFAAEADGYTATKHQREVGTGYFDAVSLAITGGQSSTTAMKESTETAQFKPAAE

110000000000000000000000000000000000000000000000000000000000000000000000000000000000111111111111111111111110000000000000000000000000000000000000000001110000000000000000000000000000111111111000000000000000000000000000000000000000000000000000000000000000000000000000000000000000000000000000000000000000000000000000000000000000000000000000000000000000000000000000000000000000000000000000000111111110000000000001111111111111111111111

>DM_train352

VDFHGYARSGIGWTGSGGEQQCFQATGAQSKYRLGNECETYAELKLGQEVWKEGDKSFYFDTNVAYSVNQQNDWESTDPAFREANVQGKNLIEWLPGSTIWAGKRFYQRHDVHMIDFYYWDISGPGAGIENIDLGFGKLSLAATRSTEAGGSYTFSSQNIYDEVKDTANDVFDVRLAGLQTNPDGVLELGVDYGRANTTDGYKLADGASKDGWMFTAEHTQSMLKGYNKFVVQYATDAMTTQGKGQARGSDGSSSFTEELSDGTKINYANKVINNNGNMWRILDHGAISLGDKWDLMYVGMYQNIDWDNNLGTEWWTVGVRPMYKWTPIMSTLLEVGYDNVKSQQTGDRNNQYKITLAQQWQAGDSIWSRPAIRIFATYAKWDEKWGYIKDGDNISRYAAATNSGISTNSRGDSDEWTFGAQMEIWW

0000000000000000000000000000000000000000000000000000000000000000000000000000000000000000000000000000000000000000000000000000000000000000000000000000000000000000000000000000000000000000000000000000000000000000000000000000000000000000000000000000000000000000001111110000000000000000000000000000000000000000000000000000000000000000000000000000000000000000000000000000000000000000000000000000000000000000000000000000000000000000000

>DM_train353

ELDGKAPSHRNLNVQTWSTAEGAKVLFVEARELPMFDLRLIFAAGSSQDGNAPGVALLTNAMLNEGVAGKDVGAIAQGFEGLGADFGNGAYKDMAVASLRSLSAVDKREPALKLFAEVVGKPTFPADSLARIKNQMLAGFEYQKQNPGKLASLELMKRLYGTHPYAHASDGDAKSIPPITLAQLKAFHAKAYAAGNVVIALVGDLSRSDAEAIAAQVSAALPKGPALAKIEQPAEPKASIGHIEFPSSQTSLMLAQLGIDRDDPDYAAVSLGNQILGGGGFGTRLMSEVREKRGLTYGVYSGFTPMQARGPFMINLQTRAEMSEGTLKLVQDVFAEYLKNGPTQKELDDAKRELAGSFPLSTASNADIVGQLGAMGFYNLPLSYLEDFMRQSQELTVEQVKAAMNKHLNVDKMVIVSAGPTVAQKPL

1111111111100000000000000000000000000000000000000000000000000000000000000000000000000000000000000000000000000000000000000000000000000000000000000000000000000000000000000000000000000000000000000000000000000000000000000000000000000000000000000000000000000000000000000000000000000000000000000000000000000000000000000000000000000000000000000000000000000000000001111100000000000000000000000000000000000000000000000000000000000000000

>DM_train354

MMEYKIVENGLTYRIGNGASVPISNTGELIKGLRNYGPYEVPSLKYNQIALIHNNQFSSLINQLKSQISSKIDEVWHIHNINISEFIYDSPHFDSIKSQVDNAIDTGVDGIMLVLPEYNTPLYYKLKSYLINSIPSQFMRYDILSNRNLTFYVDNLLVQFVSKLGGKPWILNVDPEKGSDIIIGTGATRIDNVNLFCFAMVFKKDGTMLWNEISPIVTSSEYLTYLKSTIKKVVYGFKKSNPDWDVEKLTLHVSGKRPKMKDGETKILKETVEELKKQEMVSRDVKYAILHLNETHPFWVMGDPNNRFHPYEGTKVKLSSKRYLLTLLQPYLKRNGLEMVTPIKPLSVEIVSDNWTSEEYYHNVHEILDEIYYLSKMNWRGFRSRNLPVTVNYPKLVAGIIANVNRYGGYPINPEGNRSLQTNPWFL

1111111111000000000000000000000000000000000000000000000000000000000000000000000000000000000000000000000000000000000000000000000000000000000000011110000000000000000000000000000000000000000000000000000000000000000000000000000000000000000000000000000000000000000000000000000000000000000000000000000000000011111111000000000000000000011111111110000000000000000000000000000000000000000000000000000000000000000000000000111111000000000

>DM_train355

TEAAAQPHALPADAPDIAPERDLLSKFDGLIAERQKLLDSGVTDPFAIVMEQVKSPTEAVIRGKDTILLGTYNYMGMTFDPDVIAAGKEALEKFGSGTCGSRMLNGTFHDHMEVEQALRDFYGTTGAIVFSTGYMANLGIISTLAGKGEYVILDADSHASIYDGCQQGNAEIVRFRHNSVEDLDKRLGRLPKEPAKLVVLEGVYSMLGDIAPLKEMVAVAKKHGAMVLVDEAHSMGFFGPNGRGVYEAQGLEGQIDFVVGTFSKSVGTVGGFVVSNHPKFEAVRLACRPYIFTASLPPSVVATATTSIRKLMTAHEKRERLWSNARALHGGLKAMGFRLGTETCDSAIVAVMLEDQEQAAMMWQALLDGGLYVNMARPPATPAGTFLLRCSICAEHTPAQIQTVLGMFQAAGRAVGVIGLEHHHHHH

1111111111111111111110000000000000000000000000000000000000000000000000000000000000000000000000000000000000000000000000000000000000000000000000000000000000000000000000000000000000000000000000000000000000000000000000000000000000000000000000000000000000000000000000000000000000000000000000000000000000000000000000000000000000000000000000000000000000000000000000000000000000000000000000000000000000000000000000000000000000011111111

>DM_train356

GHHHHHHMTTQLEQAWELAKQRFAAVGIDVEEALRQLDRLPVSMHCWQGDDVSGFENPEGSLTGGIQATGNYPGKARNASELRADLEQAMRLIPGPKRLNLHAIYLESDTPVSRDQIKPEHFKNWVEWAKANQLGLDFNPSCFSHPLSADGFTLSHADDSIRQFWIDHCKASRRVSAYFGEQLGTPSVMNIWIPDGMKDITVDRLAPRQRLLAALDEVISEKLNPAHHIDAVESKLFGIGAESYTVGSNEFYMGYATSRQTALCLDAGHFHPTEVISDKISAAMLYVPQLLLHVSRPVRWDSDHVVLLDDETQAIASEIVRHDLFDRVHIGLDFFDASINRIAAWVIGTRNMKKALLRALLEPTAELRKLEAPGDYTARLALLEEQKSLPWQAVWEMYCQRHDTPAGSEWLESVRAYEKEILSRRG

111111111000000000000000000000000000000000000000000000000000000000000000000000000000000000000000000000000000000000000000000000000000000000000000000000000000000000000000000000000000000000000000000000000000000000000000000000000000000000000000000000000000000000000000000000000000000000000000000000000000000000000000000000000000000000000000000000000000000000000000000000000000000000000000000000000000000000000000000000000000000000

>DM_train357

GMKVTNYQGATIDPYSKGLGMVPGTSIQLTDAARLEWNLLNEDVSLPAAVLYADRVEHNLKWMQAFVAEYGVKLAPHGKTTMAPQLFRRQLETGAWGITLATAHQVRAAYHGGVSRVLMANQLVGRRNMMMVAELLSDPEFEFFCLVDSVEGVEQLGEFFKSVNKQLQVLLELGVPGGRTGVRDAAQRNAVLEAITRYPDTLKLAGVELYEGVLKEEHEVREFLQSAVAVTRELVEQERFARAPAVLSGAGSAWYDVVAEEFVKASETGKVEVVLRPGCYLTHDVGIYRKAQTDIFARNPVAKKMGEGLLPALQLWAYVQSIPEPDRAIIGLGKRDSAFDAGMPEPARHYRPGNEAPRDIAASEGWEIFGLMDQHAYLRIPAGADLKVGDMIAFDISHPCLTFDKWRQVLVVDPAYRVTEVIETFF

111111110000000000000000000000000000000000000000000000000000000000000000000000000000000000000000000000000000000000000000000000000000000000000000000000000000000000000000000000000000000000000000000000000000000000000000000000000000000000000000000000000000000000000000000000000000000000111111111111111111111100000000000000000000000000000000000000000000000000000000000000000000000000000000000000000000000000000000000000000000000000

>DM_train358

MKNTTLYTYRDYFVIRGGKPLTGKVKISGAKNAALPIMFATILTEEPCTITNVPDLLDVRNTLLLLRELGAELEFLNNTVFINPSINSFITNQEIIRRMRASVLSLGPLLGRFGRAVVGLPGGCSIGARPIDQHLKFFKEAGADVEVREGYVYVNLKEKRRVHFKFDLVTVTGTENALLYLASVPEESILENIALEPEVMDLIEVLKKMGAHVKVEGRSAYVKGSENLKGFTHSVIPDRIEAGTFMVGAVLTDGEILLENARINHLRAVVEKLKLIGGEVVEENGNLRVFRKESLRACDIETQVYPGFPTDMQAQFMALLSVAKGKSRIKENIFEHRFHHAQELNRLGANITVRGNTAYVEGVERLYGSEVYSTDLRASASLVLAGLVAQGETVVRDVYHLDRGYEKLEEKLKKLGADIERVSEL

11111111000000000000000000000000000000000000000000000000000000000000000000000000000000000000000000000000000000000000000000000000000000000000000000000000000000000000000000000000000000000000000000000000000000000000000000000000000000000000000000000000000000000000000000000000000000000000000000000000000000000000000000000000000000000000000000000000000000000000000000000000000000000000000000000000000000000000000000000000000000001

>DM_train359

GSMSFIPVAEDSDFPIQNLPYGVFSTQSNPKPRIGVAIGDQILDLSVIKHLFTGPALSKHQHVFDETTLNNFMGLGQAAWKEARASLQNLLSASQARLRDDKELRQRAFTSQASATMHLPATIGDYTDFYSSRQHATNVGIMFRGKENALLPNWLHLPVGYHGRASSIVVSGTPIRRPMGQMRPDNSKPPVYGACRLLDMELEMAFFVGPGNRFGEPIPISKAHEHIFGMVLMNDWSARDIQQWEYVPLGPFLGKSFGTTISPWVVPMDALMPFVVPNPKQDPKPLPYLCHSQPYTFDINLSVSLKGEGMSQAATICRSNFKHMYWTMLQQLTHHSVNGCNLRPGDLLASGTISGSDPESFGSMLELSWKGTKAIDVGQGQTRTFLLDGDEVIITGHCQGDGYRVGFGQCAGKVLPALSPAGS

000000000000000000000000000000000000000000000000000000000000000000000000000000000000000000000000000000000000000000000000000000000000000000000000000000000000000000000000000000000000000000000000000000000000000000000000000000000000000000000000000000000000000000000000000000000000000000000000000000000000000000000000000000000000000000000000000000000000000000000000000000000000000000000000000000000000000000000000000000000011111

>DM_train360

MSSGVDLGTENLYFQSNAMYMAQPGHIDHIKQINAGRVYKLIDQKGPISRIDLSKESELAPASITKITRELIDAHLIHETTVQEAISRGRPAVGLQTNNLGWQFLSMRLGRGYLTIALHELGGEVLIDTKIDIHEIDQDDVLARLLFEIEEFFQTYAAQLDRVTSIAITLPGLVNSEQGIVLQMPHYNVKNLALGPEIYKATGLPVFVANDTRAWALAEKLFGHSQDVDNSVLISIHHGLGAGIVLDGRVLQGRHGNIGELGHIQIDPQGKRCHCGNYGCLETVASSQAIRDQVTARIQAGEPSCLATVEEISIEDICAAAADGDPLAVDVIQQLGRYLGAAIAIVINLFNPEKILIGGVINQAKSILYPSIEQCIREQSLPVYHQDLKLVESRFYKQATMPGAALIKQALYDGLLLMKVVEG

111111111111111111111111111000000000000000000000000000000000000000000000000000000000000000000000000000000000000000000000000000000000000000000000000000000000000000000000000000000000000000000000000000000000000000000000000000000000000000000000000000000000000000000000000000000000000000000000000000000000000000000000000000000000000000000000000000000000000000000000000000000000000000000000000000000000000000000000000000000000000

>DM_train361

SGDFSLLSSPINREKNGTEIVKFSIHPYKGTVIRLGEEILPFKVLEMDKNIALVEMAIPVYKDEKEIELKLSSPGFQNSSYRIRKPEELNEKLIALDKEGITHRFISRFKTGFQPKSVRFIDNTRLAIPLLEDEGMDVLDINSGQTVRLSPPEKYKKKLGFVETISIPEHNELWVSQMQANAVHVFDLKTLAYKATVDLTGKWSKILLYDPIRDLVYCSNWISEDISVIDRKTKLEIRKTDKIGLPRGLLLSKDGKELYIAQFSASNQESGGGRLGIYSMDKEKLIDTIGPPGNKRHIVSGNTENKIYVSDMCCSKIEVYDLKEKKVQKSIPVFDKPNTIALSPDGKYLYVSCRGPNHPTEGYLKKGLVLGKVYVIDTTTDTVKEFWEAGNQPTGLDVSPDNRYLVISDFLDHQIRVYRRDGF

111111111111111100000000000000000000000000000000000000000000000000000000000000000000000000000000000000000000000000000000000000000000000000000000000000000000000000000000000000000000000000000000000000000000000000000000000000000000000000000000000000000000000000000000000000000000000000000000000000000000000000000000000000000000000000000000000000000000000000000000000000000000000000000000000000000000000000000000000000000000000

>DM_train362

MRVLVRDLKAHVGQEVELLGFLHWRRDLGRIQFLLLRDRSGVVQVVTGGLKLPLPESALRVRGLVVENAKAPGGLEVQAKEVEVLSPALEPTPVEIPKEEWRANPDTLLEYRYVTLRGEKARAPLKVQAALVRGFRRYLDRQDFTEIFTPKVVRAGAEGGSGLFGVDYFEKRAYLAQSPQLYKQIMVGVFERVYEVAPVWRMEEHHTSRHLNEYLSLDVEMGFIADEEDLMRLEEALLAEMLEEALNTAGDEIRLLGATWPSFPQDIPRLTHAEAKRILKEELGYPVGQDLSEEAERLLGEYAKERWGSDWLFVTRYPRSVRPFYTYPEEDGTTRSFDLLFRGLEITSGGQRIHRYEELLESLKAKGMDPEAFHGYLEVFKYGMPPHGGFAIGAERLTQKLLGLPNVRYARAFPRDRHRLTP

00000000000000000000000000000000000000000000000000000000000000000000000000000000000000000000011111111111111111000000000000000000000000000000000000000011111111111111111111111111110000000000000000000001111111111111000000000000000000000000000000000000000000000000000000000000000000000000000000000000000000000000000000000000000000000000000000000000000000000000000000000000000000000000000000000000000000000000000000000011111111

>DM_train363

GSQSRSIQTFPQPDTSVINGPDRPAGIPDPAGTTVAGGGAVYTVVPHLSMPHWAAQDFAKSLQSFRLGCANLKNRQGWQDVCAQAFQTPIHSFQAKRFFERYFTPWQVAGNGSLAGTVTGYYEPVLKGDGRRTERARFPIYGIPDDFISVPLPAGLRGGKNLVRIRQTGKNSGTIDNAGGTHTADLSRFPITARTTAIKGRFEGSRFLPYHTRNQINGGALDGKAPILGYAEDPVELFFMHIQGSGRLKTPSGKYIRIGYADKNEHPYVSIGRYMADKGYLKLGQTSMQGIKAYMRQNPQRLAEVLGQNPSYIFFRELAGSGGDGPVGALGTPLMGEYAGAIDRHYITLGAPLFVATAHPVTRKALNRLIMAQDTGSAIKGAVRVDYFWGYGDEAGELAGKQKTTGYVWQLLPNGMKPEYRP

11111111111111111111000000000000000000000000000000000000000000000000000000000000000000000000000000000000000000000000000000000000000000000000000000000000011111111000000000000111111100000000000000000000000000000000000000000000000000000000000000000000000000000000000000000000000000000000000000000000000000000000000000000011111000000000000000000000000000000000000000000000000000000000000000000000000000000000000000000000000000

>DM_train365

MGLPPLLSLPSNSAPRSLGRVETPPEVVDFMVSLAEAPRGGRVLEPACAHGPFLRAFREAHGTGYRFVGVEIDPKALDLPPWAEGILADFLLWEPGEAFDLILGNPPYGIVGEASKYPIHVFKAVKDLYKKAFSTWKGKYNLYGAFLEKAVRLLKPGGVLVFVVPATWLVLEDFALLREFLAREGKTSVYYLGEVFPQKKVSAVVIRFQKSGKGLSLWDTQESESGFTPILWAEYPHWEGEIIRFETEETRKLEISGMPLGDLFHIRFAARSPEFKKHPAVRKEPGPGLVPVLTGRNLKPGWVDYEKNHSGLWMPKERAKELRDFYATPHLVVAHTKGTRVVAAWDERAYPWREEFHLLPKEGVRLDPSSLVQWLNSEAMQKHVRTLYRDFVPHLTLRMLERLPVRREYGFHTSPESARNF

1111111111111111111100000000000000000000000000000000000000000000000000000000000000000000000000000000000000000001111111111110000000000000000000000000000000000000000000000000000000000000000000000000000000000000000000000000000000000000000000000000000000000000000000000000000000000000000000000000000000000000000000000000000000000000000000000000000000000000000000000000000000000000000000000000000000000000000000000000011111111

>DM_train366

MSRTVMERIEYEMHTPDPKADPDKLHFVQIDEAKCIGCDTCSQYCPTAAIFGEMGEPHSIPHIEACINCGQCLTHCPENAIYEAQSWVPEVEKKLKDGKVKCIAMPAPAVRYALGDAFGMPVGSVTTGKMLAALQKLGFAHCWDTEFTADVTIWEEGSEFVERLTKKSDMPLPQFTSCCPGWQKYAETYYPELLPHFSTCKSPIGMNGALAKTYGAERMKYDPKQVYTVSIMPCIAKKYEGLRPELKSSGMRDIDATLTTRELAYMIKKAGIDFAKLPDGKRDSLMGESTGGATIFGVTGGVMEAALRFAYEAVTGKKPDSWDFKAVRGLDGIKEATVNVGGTDVKVAVVHGAKRFKQVCDDVKAGKSPYHFIEYMACPGGCVCGGGQPVMPGVLEAMDRTTTRLYAGLKKRLAMASANKA

0000000000000000000000000000000000000000000000000000000000000000000000000000000000000000000000000000000000000000000000000000000000000000000000000000000000000000000000000000000000000000000000000000000000000000000000000000000000000000000000000000000000000000000000000000000000000000000000000000000000000000000000000000000000000000000000000000000000000000000000000000000000000000000000000000000000000111111111111111111111111

>DM_train367

MGSDKIHHHHHHMKEKVVLAYSGGLDTSVILKWLCEKGFDVIAYVANVGQKDDFVAIKEKALKTGASKVYVEDLRREFVTDYIFTALLGNAMYEGRYLLGTAIARPLIAKRQVEIAEKEGAQYVAHGATGKGNDQVRFELTYAALNPNLKVISPWKDPEFLAKFKGRTDLINYAMEKGIPIKVSKKRPYSEDENLMHISHEAGKLEDPAHIPDEDVFTWTVSPKDAPDEETLLEIHFENGIPVKVVNLKDGTEKTDPLELFEYLNEVGAKNGVGRLDMVENRFIGIKSRGVYETPGATILWIAHRDLEGITMDKEVMHLRDMLAPKFAELIYNGFWFSPEMEFLLAAFRKAQENVTGKVTVSIYKGNVMPVARYSPYSLYNPELSSMDVEGGFDATDSKGFINIHALRLKVHQLVKKGYQR

1111111111111000000000000000000000000000000000000000000000000000000000000000000000000000000000000000000000000000000000000000000000000000000000000000000000000000000000000000000000000111100000000000000000000000000000000000000000000000000000000000000000000000000000000000000000000000000000000000000000000000000000000000000000000000000000000000000000000000000000000000000000000000000001111111111000000000000000000000000011111

>DM_train368

MSRNPSNSDAAHAFWSTQPVPQTEDETEKIVFAGPMDEPKTVADIPEEPYPIASTFEWWTPNMEAADDIHAIYELLRDNYVEDDDSMFRFNYSEEFLQWALCPPSYIPDWHVAVRRKADKKLLAFIAGVPVTLRMGTPKYMKVKAQEKGQEEEAAKYDAPRHICEINFLCVHKQLREKRLAPILIKEVTRRVNRTNVWQAVYTAGVLLPTPYASGQYFHRSLNPEKLVEIRFSGIPAQYQKFQNPMAMLKRNYQLPNAPKNSGLREMKPSDVPQVRRILMNYLDNFDVGPVFSDAEISHYLLPRDGVVFTYVVENDKKVTDFFSFYRIPSTVIGNSNYNILNAAYVHYYAATSMPLHQLILDLLIVAHSRGFDVCNMVEILDNRSFVEQLKFGAGDGHLRYYFYNWAYPKIKPSQVALVML

1111111111000000000000000000000000000000000000000000000000000000000000000000000000111000000000000000000000000000000000000000000000000000000000000000000000000000000000000000000000000000000000000000000000000000000000000000000000000000000000000000000000000000000000000000000000000000000000000000000000000000000000000000000000000000000001111110000000000000000000000000000000000000000000000000000000000000000000000000000000000

>DM_train371

MFVFMLLLLLPFTISKAKDFPANPIEKAGYKLDFSDEFNGPTLDREKWTDYYLPHWCKDPESAKANYRFENGSLVEYITEDQKPWCPEHDGTVRSSAIMSFDKSWIHNFSGTTDNHERNEWRGYTTKYGYFEIRAKLSNTGGGGHQAWWMVGMQDDTNDWFNSKQTGEIDILETFFSKKDTWRIAAYGWNDPNFQTSWTISEDKVPSGDPTSEYHIYAMEWTPTALKFYYDNELFKVIYGSPDYEMGTILNIYTDAGSGAHNDVWPKEWAIDYMRVWKPVDGYKESESLNNYLIRNRQTGKFLYIEENNDKVSYGDITLKNEKNAKWSKEYRDGYTLLKNNETGEYLNIENQTGYIEHGKVPKTWWSAQWSEVPVDGYTRFVNRWKPNMSIHTESYEGVLQYGNVPNTYWTSQWQLIPVE

111111111111111111000000000000000000000000000000000000000000000000000000000000000000000000000000000000000000000000000000000000000000000000000000000000000000000000000000000000000000000000000000000000000000000000000000000000000000000000000000000000000000000000000000000000000000000000001111100000000000000000000000000000000000000000000000000000000000000000000000000000000000000000000000000000000000000000000000000000000000

>DM_train373

MIDLSKTVFYTSIDIGSRYIKGLVLGKRDQEWEALAFSSVKSRGLDEGEIKDAIAFKESVNTLLKELEEQLQKSLRSDFVISFSSVSFEREDTVIERDFGEEKRSITLDILSEMQSEALEKLKENGKTPLHIFSKRYLLDDERIVFNPLDMKASKIAIEYTSIVVPLKVYEMFYNFLQDTVKSPFQLKSSLVSTAEGVLTTPEKDRGVVVVNLGYNFTGLIAYKNGVPIKISYVPVGMKHVIKDVSAVLDTSFEESERLIITHGNAVYNDLKEEEIQYRGLDGNTIKTTTAKKLSVIIHARLREIMSKSKKFFREVEAKIVEEGEIGIPGGVVLTGGGAKIPRINELATEVFKSPVRTGCYANSDRPSIINADEVANDPSFAAAFGNVFAVSENPYEETPVKSENPLKKIFRLFKELME

11111100000000000000000000000000000000000000000000000000000000000000000000000000000000000000000000000000000000000000000000000000000000000000000000000000000000000000000000000000000000000000000000000000000000000000000000000000000000000000000000000000000000000000000000000000000000000000000000000000000000000000000000000000111111000000000000000000000000000000000000000000000000000000000000000011111111111111111111111111111

>DM_train375

MAAKNAFYAQSGGVTAVINASAAGVIEAARKQSGKIGRIYAGRNGIIGALTEDLIDTGQESDAAISALRYTPSGAFGSCRYKLKSLEQNRREYERLIEVFKAHDIGYFFYNGGGDSADTCLKVSQLSGTLGYPIQAIHVPKTVDNDLPITDCCPGFGSVAKYIAVSTLEASFDVASMSATSTKVFVLEVMGRHAGWIAAAGGLASSPEREIPVVILFPEISFDKQKFLAKVDSCVKKFGYCSVVVSEGVKGDDGKFLSDQGVRDAFGHAQLGGVAPVVASMVKEGLGLKYHWGVADYLQRAARHIASKTDVEQAYAMGQAAVEFAVQGHNSVMPTIERISARPYQWKVGMAQLSQVANVEKMMPENFITEDGFGITDLCREYLAPLIEGEDYPPYKDGLPDYVRLKNVAVPKKLSGFTL

10000000000000000000000000000000000000000000000000000000000000000000000000000000001111110000000000000000000000000000000000000000000000000000000000000000000000000000000000000000000000000000000000000000000000000000000000000000000000000000000000000000000000001111111111111111000000000000000000000000000000000000000000000000000000000000000000000100000000000000000000000000000000000000000000000000000000000000000000000000001

>DM_train377

GSHMTNYKEKLQQYAELLVKVGMNVQPKQPVFIRSSVETLELTHLIVEEAYHCGASDVRVVYSDPTLKRLKFENESVEHFANHEIKSYDVEARMDYVKRGAANLALISEDPDLMDGIDSQKLQAFQQQNARAFKGYMESVQKNQFPWVVAAFPSKAWAKRVYPELSVEEAYIKFIDEVFDIVRIDGNDPVENWRQHIANLSVYAQKLQQKNYHALHYVSEGTDLTVGLAKNHIWEDATSYVNGKEQAFIANIPTEEVFTAPDRNRVDGYVTNKLPLSYNGTIIDQFKLMFKDGEIIDFSAEKGEAVLKDLINTDEGSRRLGEVALVPDDSPISNRNTIFYNTLFDENAACHLAIGSAYAFNIQGGTEMTVEEKIASGLNDSNVHVDFMIGSSDLTIYGIFEDGSKELVFENGNWASTF

1111100000000000000000000000000000000000000000000000000000000000000000000000000000000000000000000000000000000000000000000000000000000000000000000000000000000000000000000000000000000000000000000000000000000000000000000000000000000000000000000000000000000000000000000000000000000000000000000000000000000000000000000000000000000000000000000000000000000000000000000000000000000000000000000000000000000000000000000000000000

>DM_train378

MSLSTPLQGIKVLDFTGVQSGPSCTQMLAWFGADVIKIERPGVGDVTRHQLRDIPDIDALYFTMLNSNKRSIELNTKTAEGKEVMEKLIREADILVENFHPGAIDHMGFTWEHIQEINPRLIFGSIKGFDECSPYVNVKAYENVAQAAGGAASTTGFWDGPPLVSAAALGDSNTGMHLLIGLLAALLHREKTGRGQRVTMSMQDAVLNLCRVKLRDQQRLDKLGYLEEYPQYPNGTFGDAVPRGGNAGGGGQPGWILKCKGWETDPNAYIYFTIQEQNWENTCKAIGKPEWITDPAYSTAHARQPHIFDIFAEIEKYTVTIDKHEAVAYLTQFDIPCAPVLSMKEISLDPSLRQSGSVVEVEQPLRGKYLTVGCPMKFSAFTPDIKAAPLLGEHTAAVLQELGYSDDEIAAMKQNHAI

1100000000000000000000000000000000000000000000000000000000000000000000000000000000000000000000000000011111110000000000000000000000000000000000000000000000000000000000000000000000000000000000000000000000000000000000000000000000000000000000000000000000000000000000000000000000000000000000000000000000000000000000000000000000000000000000000000000000000000000000000000000000000000000000000000000000000000000000000000000000

>DM_train379

SESPWIAEDTSQIQTKLLEFAKSPEVFDWMVKIRRKIHENPELGYEELETSKLIRSELELIGIKYRYPVAITGVIGYIGTGEPPFVALRADMDALPIQEGVEWEHKSKIAGKMHACGHDGHVTMLLGAAKILHEHRHHLQGTVVLIFQPAEEGLSGAKKMREEGALKNVEAIFGIHLSARIPFGKAASRAGSFLAGAGVFEAVITGKGGHAAIPQHTIDPVVAASSIVLSLQQLVSRETDPLDSKVVTVSKVNGGNAFNVIPDSITIGGTLRAFTGFTQLQQRVKEVITKQAAVHRCNASVNLTPNGREPMPPTVNNKDLYKQFKKVVRDLLGQEAFVEAAPVMGSEDFSYFAETIPGHFSLLGMQDETNGYASSHSPLYRINEDVLPYGAAIHASMAVQYLKEKASKGSVSGFHEEL

1111111111111110000000000000000000000000000000000000000000000000000000000000000000000000000000000000000000000000000000000000000000000000000000000000000000000000000000000000000000000000000000000000000000000001111111110000000000000000000000000000000000000111111110000000000000000000000000000000000000000000000000000000000000000000000000000000000000000000000000000000000000000000000000000000000000000000000000011111111111

>DM_train381

TSPTTTSTGTGTGSSTDQAISPLVEGENAPEVTTAKLGFIALTDAAPLIIAKEKGFYAKYGMPDVEVLKQASWGTTRDNLVLGSASGGIDGAHILTPMPYLITMGTVTDGKPTPMYILARLNVNGQGIQLGNNYKDLKVGTDAAPLKEAFAKVTDPKVAMTFPGGTHDMWIRYWLAAGGMEPGKDFSTIVVPPAQMVANVKVNAMESFCVGEPWPLQTVNQGVGYQALTTGQLWKDHPEKAFGMRADWVDQNPKAAKALLMAVMEAQQWCDQAENKEEMCQILSKREWFKVPFEDIIDRSKGIYNFGNGQETFEDQEIMQKYWVDNASYPYKSHDQWFLTENIRWGYLPASTDTKAIVDKVNREDLWREAAQALEVPADQIPSSPSRGIETFFDGITFDPENPQAYLDSLKIKSIKA

111111111111111111111111111000000000000000000000000000000000000000000000000000000000000000000000000000000000000000000000000000000000000000000000000000000000000000000000000000000000000000000000000000000000000000000000000000000000000000000000000000000000000000000000000000000000000000000000000000000000000000000000000000000000000000000000000000000000000000000000000000000000000000000000000000000000000000000000000011111

>DM_train382

MSLSNKLTLDKLDVKGKRVVMRVDFNVPMKNNQITNNQRIKAAVPSIKFCLDNGAKSVVLMSHLGRPDGVPMPDKYSLEPVAVELKSLLGKDVLFLKDCVGPEVEKACANPAAGSVILLENLRFHVEEEGKGKDASGNKVKAEPAKIEAFRASLSKLGDVYVNDAFGTAHRAHSSMVGVNLPQKAGGFLMKKELNYFAKALESPERPFLAILGGAKVADKIQLINNMLDKVNEMIIGGGMAFTFLKVLNNMEIGTSLFDEEGAKIVKDLMSKAEKNGVKITLPVDFVTADKFDENAKTGQATVASGIPAGWMGLDCGPESSKKYAEAVTRAKQIVWNGPVGVFEWEAFARGTKALMDEVVKATSRGCITIIGGGDTATCCAKWNTEDKVSHVSTGGGASLELLEGKVLPGVDALSNI

110000000000000000000000000000000000000000000000000000000000000000000000000000000000000000000000000011110000000000000000000000000000000000000000000000000000000000000000000000000000000000000000000000000000000000000000000000000000000000000000000000000000000000000000000000000000000000000000000000000000000000000000000000000000000000000000000000000000000000000000000000000000011111111111110000000000000000000000000000000

>DM_train385

CGSSSHETSYGYATLSYADYWAGELGQSRDVLLAGNAEADRAGDLDAGMFDAVSRATHGHGAFRQQFQYAVEVLGEKVLSKQETEDSRGRKKWEYETDPSVTKMVRASASFQDLGEDGEIKFEAVEGAVALADRASSFMVDSEEYKITNVKVHGMKFVPVAVPHELKGIAKEKFHFVEDSRVTENTNGLKTMLTEDSFSARKVSSMESPHDLVVDTVGTGYHSRFGSDAEASVMLKRADGSELSHREFIDYVMNFNTVRYDYYGDDASYTNLMASYGTKHSADSWWKTGRVPRISCGINYGFDRFKGSGPGYYRLTLIANGYRDVVADVRFLPKYEGNIDIGLKGKVLTIGGADAETLMDAAVDVFADGQPKLVSDQAVSLGQNVLSADFTPGTEYTVEVRFKEFGSVRAKVVAQ

1111110000000000000000000000000000111111111000000000000000000000000000000000000000000000000000000000000000000000000000000000000000000000000000000000000000000000000000000000000000000000000000000000000000000000000000000000000000000000000000000000000000000000000000000000000000000000000000000000000000000000000000000000000000000000000000000000000000000000000000000000000000000000000000000000000000000000000000000000000

>DM_train386

MGSDKIHHHHHHPEQQVQQFAQLPVGFKNMGNTCYLNATLQALYRVNDLRDMILNYNPSQGVSNSGAQDEEIHKQIVIEMKRCFENLQNKSFKSVLPVVLLNTLRKCYPQFAERDSQGGFYKQQDAEELFTQLFHSMSIVFGDKFSEDFRIQFKTTIKDTANDNDITVKENESDSKLQCHISGTTNFMRNGLLEGLNEKIEKRSDLTGANSIYSVEKKISRLPKFLTVQYVRFFWKRSTNKKSKILRKVVFPFQLDVADMLTPEYAAEKVKVRDELRKVEKEKNEKEREIKRRKFDPSSSENVMTPREQYETQVALNESEKDQWLEEYKKHFPPNLEKGENPSCVYNLIGVITHQGANSESGHYQAFIRDELDENKWYKFNDDKVSVVEKEKIESLAGGGESDSALILMYKGFGL

1111111111111111110000000000000000000000000000000000000000000000000000000000000000000000001000000000000000000000000111100000000000000000000000000000000000000000000000000000000000000000000000000000000111111111000000000000000000000000000000000000000000000000000000000000000000000000000000111111111111111100000000000000000000000000000000000000000000000000000000000000000000000000000000000000000000000000000000000000000

>DM_train387

MSSKPKYVQDQEMIPGVYWVGIVDWMVRIFHGYHTDEGSSYNSYFIDDECPTVIDSVKYPFAEEWLSRIAACCPLDKIKYVVMNHAEGDHASSLKDHYHKFTNATFVCTKKCQEHLKILYGMEKATWLIVDDKYTLKIGKRTLKFIPVPLLHWPDSTFTYCPEDKILFSNDGFGQHYATSRRWADECDVSHVMHLFKEYTANILGLFSAQMRKALEVASTVEIKYILSAHGVSWRGDAMGLAIAEYDRWSKGQHCQKKVTVVLDSMYGTTHRMALALLDGARSTGCETVLLEMTSSDITKVALHTYDSGAVAFASPTLNNTMMPSVAAALNYVRGLTLIKGKPAFAFGAFGWSNRAVPDIVAELRDGCKADVYDEKGITFKFNYTEELLEQAYNAGVDLGKRAIAYCEKNAPKQ

111110000000000000000000000000000000000000000000000000000000000000000000000000000000000000000000000000000000000000000000000000000000000000000000000000000000000000000000000000000000000000000000000000000000000000000000000000000000000000000000000000000000000000000000000000000000000000000000000000000000000000000000000000000000000000000000000000000000000000000000000000000000000000000000000000000000000000000000000011

>DM_train388

MGQLFSSPKSDENNDLPSSFTGYFKKFNTGRKIISQEILNLIELRMRAGNIQLTNSAISDALKEIDSSVLNVAVTGETGSGKSSFINTLRGIGNEEEGAAKTGVVEVTMERHPYKHPNIPNVVFWDLPGIGSTNFPPDTYLEKMKFYEYDFFIIISATRFKKNDIDIAKAISMMKKEFYFVRTKVDSDITNEADGEPQTFDKEKVLQDIRLNCVNTFRENGIAEPPIFLLSNKNVCHYDFPVLMDKLISDLPIYKRHNFMVSLPNITDSVIEKKRQFLKQRIWLEGFAADLVNIIPSLTFLLDSDLETLKKSMKFYRTVFGVDETSLQRLARDWEIEVDQVEAMIKSPAVFKPTDEETIQERLSRYIQEFCLANGYLLPKNSFLKEIFYLKYYFLDMVTEDAKTLLKEICLRN

11111111111110000000000000000000000000000000000000000000000000000000000000000000000000000000000000000000111100000000000000000000000000000000000000000000000000000000000000000000000000000000000000000000000000000000000000000000000000000000000000000000000000000000000000000000000000000000000000000000000000000000000000000000000000000000000000000000000000000000000000000000000000000000000000000000000000000000000000000

>DM_train389

KIQGGSVVEMQGDEMTRIIWELIKEKLILPYVELDLHSYDLGIENRDATNDQVTKDAAEAIKKYNVGVKCATITPDEKRVEEFKLKQMWKSPNGTIRNILGGTVFREAIICKNIPRLVTGWVKPIIIGRHAYGDQYRATDFVVPGPGKVEITYTPKDGTQKVTYMVHDFEEGGGVAMGMYNQDKSIEDFAHSSFQMALSKGWPLYLSTKNTILKKYDGRFKDIFQEIYDKKYKSQFEAQNICYEHRLIDDMVAQAMKSEGGFIWACKNYDGDVQSDSVAQGYGSLGMMTSVLICPDGKTVEAEAAHGTVTRHYRMYQKGQETSTNPIASIFAWSRGLAHRAKLDNNTELSFFAKALEDVCIETIEAGFMTKDLAACIKGLPNVQRSDYLNTFEFMDKLGENLKAKLAQAK

00000000000000000000000000000000000000000000000000000000000000000000000000000000000000000000000000000000000000000000000000000000000000000000000000000000000000000000000000000000000000000000000000000000000000000000000000000000000000000000000000000000000000000000000000000000000000000000000000000000000000000000000000000000000000000000000000000000000000000000000000000000000000000000000000000000000000000000000001

>DM_train390

ADGQQGAPLNSPNTYDVTTWRIKAHPEVTAQSDIGAVINDIIADIKQRQTSPDARPGAAIIIPPGDYDLHTQVVVDVSYLTIAGFGHGFFSRSILDNSNPTGWQNLQPGASHIRVLTSPSAPQAFLVKRAGDPRLSGIVFRDFCLDGVGFTPGKNSYHNGKTGIEVASDNDSFHITGMGFVYLEHALIVRGADALRVNDNMIAECGNCVELTGAGQATIVSGNHMGAGPDGVTLLAENHEGLLVTGNNLFPRGRSLIEFTGCNRCSVTSNRLQGFYPGMLRLLNGCKENLITANHIRRTNEGYPPFIGRGNGLDDLYGVVHIAGDNNLISDNLFAYNVPPANIAPAGAQPTQILIAGGDANVVALNHVVSDVASQHVVLDASTTHSKVLDSGTASQITSYSSDTAIRPTP

11111111111000000000000000000000000000000000000000000000000000000000000000000000000000000000000000000000000000000000000000000000000000000000000000000000000000000000000000000000000000000000000000000000000000000000000000000000000000000000000000000000000000000000000000000000000000000000000000000000000000000000000000000000000000000000000000000000000000000000000000000000000000000000000000000000000000000000000000

>DM_train391

MSVFDSKFKGIHVYSEIGELESVLVHEPGREIDYITPARLDELLFSAILESHDARKEHKQFVAELKANDINVVELIDLVAETYDLASQEAKDKLIEEFLEDSEPVLSEEHKVVVRNFLKAKKTSRELVEIMMAGITKYDLGIEADHELIVDPMPNLYFTRDPFASVGNGVTIHYMRYKVRQRETLFSRFVFSNHPKLINTPWYYDPSLKLSIEGGDVFIYNNDTLVVGVSERTDLQTVTLLAKNIVANKECEFKRIVAINVPKWTNLMHLDTWLTMLDKDKFLYSPIANDVFKFWDYDLVNGGAEPQPVENGLPLEGLLQSIINKKPVLIPIAGEGASQMEIERETHFDGTNYLAIRPGVVIGYSRNEKTNAALEAAGIKVLPFHGNQLSLGMGNARCMSMPLSRKDVKW

10000000000000000000000000000000000000000000000000000000000000000000000000000000000000000000000000000000000000000000000000000000000000000000000000000000000000000000000000000000000000000000000000000000000000000000000000000000000000000000000000000000000000000000000000000000000000000000000000000000000000000000000000000000000000000000000000000000000000000000000000000000000000000000000000000000000000000000000000

>DM_train392

GMNNIQAIRKKVETQIDDLQNKTDEIAEFNQAKVLDAFQENKVSDFHFHPSTGYGYDDEGRDTLERVYATVFKTEAALVRPQIISGTHAISTVLFGILRPDDELLYITGQPYDTLEEIVGIRKQGQGSLKDFHIGYSSVPLLENGDVDFPRIAKKMTPKTKMIGIQRSRGYADRPSFTIEKIKEMIVFVKNINPEVIVFVDNCYGEFVEYQEPPEVGADIIAGSLIKNPGGGLAKTGGYIAGKEALVDLCGYRLTTPGIGREAGASLYSLLEMYQGFFLAPHVTAQAIKGARFTAAMLAEFGVEADPVWDAPRTDLIQSVSFHNKEKMVAFAQAIQAASPVNAHVLPIGAYMPGYEDDVIMAAGTFIQGASLELTADGPIREPYQLYVQGGLTYEHIKIAVTRAIQKIV

1111000000000000000000000000000000000000000000000000000000000000000000000000000000000000000000000000000000000000000000000000000000000000000000000000000000000000000000000000000000000000000000000000000000000000000000000000000000000000000000000000000000000000000000000000000000000000000000000000000000000000000000000000000000011000000000000000000000000000000001100000000000000000001111110000000000000000000000001

>DM_train393

APQVVDKVAAVVNNGVVLESDVDGLMQSVKLNAAQARQQLPDDATLRHQIMERLIMDQIILQMGQKMGVKISDEQLDQAIANIAKQNNMTLDQMRSRLAYDGLNYNTYRNQIRKEMIISEVRNNEVRRRITILPQEVESLAQQVGNQNDASTELNLSHILIPLPENPTSDQVNEAESQARAIVDQARNGADFGKLAIAHSADQQALNGGQMGWGRIQELPGIFAQALSTAKKGDIVGPIRSGVGFHILKVNDLRGESKNISVTEVHARHILLKPSPIMTDEQARVKLEQIAADIKSGKTTFAAAAKEFSQDPGSANQGGDLGWATPDIFDPAFRDALTRLNKGQMSAPVHSSFGWHLIELLDTRNVDKTDAAQKDRAYRMLMNRKFSEEAASWMQEQRASAYVKILSN

111100000000000000000000000000000000000000000000000000000000000000000000000000000000000000000000000000000000000000000000000000000000000000000001111111100000000000000000000000000000000000000000000000000000000000000000000000000000000000000000000000000000011111111100000000000000000000000000000000000000000000000000000000000000000000000000000000000000000000000000000001111111111000000000000000000000000000000000

>DM_train394

MLFDEKEILAITRWAKLYANQSPDRILLGSNDIPPEYRAAVATQIELWPRLRNKLPQWAGISSLYIPSRLSLEQSSGAVTSSYKSRFIREGTKVVDLTGGLGIDFIALMSKASQGIYIERNDETAVAARHNIPLLLNEGKDVNILTGDFKEYLPLIKTFHPDYIYVDPARRSGADKRVYAIADCEPDLIPLATELLPFCSSILAKLSPMIDLWDTLQSLLHVQELHVVAAHGEVKELLVRMSLNEATIPPEKVPIHAINLLLEDTVIPFIFTMEEERSISIPYTDSIDKYVYEPHTALLKAGAFKTVAYRLGLRKLHPNSHLYTSEAYESAFPGRTFVLEEIIPFSTSVLKQLRKVVPQASISCRNFPLSPIELRQRSKMADGGEKTLMGTTMADGKKVLLLLRKAE

00000000000000000000000000000000000000000000000000000000000000000000000000000000000000000000000000000000000000000000000000000000000000000000000000000000000000000000000000011111000000000000000000000000000000000000000000000000000000000000000000000000000000000000000100000000000000000000000000000000000000000000000000000000000000000000000000000000000000000000000000000000000000000000000000000000000000000000000

>DM_train395

PGSSVSGTFGCLGGRLTIPGTGVSLLVPNGAIPQGKFYDLYLRINKTESTLPLSEGSQTVLSPSVTCGPTGLLLCRPVVLTVPHCAEVIAGDWIFQLKTQAHQGHWEEVVTLDEETLNTPCYCQLEAKSCHILLDQLGTYVFTGESYSRSAVKRLQLAIFAPALCTSLEYSLRVYCLEDTPAALKEVLELERTLGGYLVEEPKPLLFKDSYHNLRLSLHDIPHAHWRSKLLAKYQEIPFYHVWNGSQKALHCTFTLERHSLASTEFTCKVCVRQVEGEGQIFQLHTTLAETPAGSLDALCSAPGNAATTQLGPYAFKIPLSIRQKICNSLDAPNSRGNDWRLLAQKLSMDRYLNYFATKASPTGVILDLWEARQQDDGDLNSLASALEEMGKSEMLVAMTTDGDC

000000000000000000000000000000000000000000000000000000000000000000000000000000000000000000000000000000000000000000000000000000000000000000000000000000000000000000000000000000000000000000000000000000000000000000000000000000000000000000000000000000000000000000000000000000000000000000000000111111111111111111100000000000000000000000000000000000000000000000000000000000000000000000000000000000000000000000111

>DM_train396

MADLLPEHPEFLWANPEPKKSYDAIIVGGGGHGLATAYFLAKNHGITNVAVLEKGWLAGGNMARNTTIIRSNYLWDESAGIYEKSLKLWEQLPEDLEYDFLFSQRGVLNLAHTLGDVRESVRRVEANKLNGVDAEWLDPSQVKEACPIINTSDDIRYPVMGATWQPRAGIAKHDHVAWAFARKANEMGVDIIQNCEVTGFIKDGEKVTGVKTTRGTIHAGKVALAGAGHSSVLAEMAGFELPIQSHPLQALVSELFEPVHPTVVMSNHIHVYVSQAHKGELVMGAGIDSYNGYGQRGAFHVIQEQMAAAVELFPIFARAHVLRTWGGIVDTTMDASPIISKTPIQNLYVNCGWGTGGFKGTPGAGFTLAHTIANDEPHELNKPFSLERFETGHLIDEHGAAAVAH

110000000000000000000000000000000000000000000000000000000000000000000000000000000000000000000000000000000000000000000000000000000000000000000000000000000000000000000000000000000000000000000000000000000000000000000000000000000000000000000000000000000000000000000000000000000000000000000000000000000000000000000000000000000000000000000000000000000000000000000000000000000000000000000000000000000000000000000

>DM_train398

LHLDQTPSRQPIPSEGLQLHLPQVLADAVSRLVLGKFGDLTDNFSSPHARRKVLAGVVMTTGTDVKDAKVISVSTGTKCINGEYMSDRGLALNDCHAEIISRRSLLRFLYTQLELYLNNKDDQKRSIFQKSERGGFRLKENVQFHLYISTSPCGDARIFSPHEPILEEPADRHPNRKARGQLRTKIESGEGTIPVRSNASIQTWDGVLQGERLLTMSCSDKIARWNVVGIQGSLLSIFVEPIYFSSIILGSLYHGDHLSRAMYQRISNIEDLPPLYTLNKPLLSGISNAEARQPGKAPNFSVNWTVGDSAIEVINATTGKDELGRASRLCKHALYCRWMRVHGKVPSHLLRSKITKPNVYHESKLAAKEYQAAKARLFTAFIKAGLGAWVEKPTEQDQFSLTP

1111111000000011110000000000000000000000000000000000000000000000000000000000000000000000000000000000000000000000000000000000000000000000000000000000000000000000000111111111111000000000000000000001111111111111111100000000000000000000000000000000000000000000000000000000000000000000000000000000000000000000000000000000000000000000000000000000000000000000000000000000000000000000000000000000000000000000001

>DM_train399

MSTFNRIHLVVLDSVGIGAAPDANNFSNAGVPDGASDTLGHISKTVGLNVPNMAKIGLGNIPRDTPLKTVPAENHPTGYVTKLEEVSLGKDTMTGHWEIMGLNITEPFDTFWNGFPEEIISKIEKFSGRKVIREANKPYSGTAVIDDFGPRQMETGELIIYTSADPVLQIAAHEDVIPLDELYRICEYARSITLERPALLGRIIARPYVGKPRNFTRTANRHDYALSPFAPTVLNKLADAGVSTYAVGKINDIFNGSGITNDMGHNKSNSHGVDTLIKTMGLSAFTKGFSFTNLVDFDALYGHRRNAHGYRDCLHEFDERLPEIIAAMKVDDLLLITADHGNDPTYAGTDHTREYVPLLAYSPSFTGNGVLPVGHYADISATIADNFGVDTAMIGESFLDKLI

1000000000000000000000000000000000000000000000000000000000000000000000000000000000000000000000000000000000000000000000000000000000000000000001111111111111000000000000000000000000000000000000000000000000000001111111111000000000000000000000000000000000000000000000000000000000000000000000000000000000000000000000000000000000000000000000000000000000000000000000000000000000000000000000000000000000000000000

>DM_train400

SKKVKVSHRSHSTEPGLVLTLGQGDVGQLGLGENVMERKKPALVSIPEDVVQAEAGGMHTVCLSKSGQVYSFGCNDEGALGRDTSVEGSEMVPGKVELQEKVVQVSAGDSHTAALTDDGRVFLWGSFRDNNGVIGLLEPMKKSMVPVQVQLDVPVVKVASGNDHLVMLTADGDLYTLGCGEQGQLGRVPELFANRGGRQGLERLLVPKCVMLKSRGSRGHVRFQDAFCGAYFTFAISHEGHVYGFGLSNYHQLGTPGTESCFIPQNLTSFKNSTKSWVGFSGGQHHTVCMDSEGKAYSLGRAEYGRLGLGEGAEEKSIPTLISRLPAVSSVACGASVGYAVTKDGRVFAWGMGTNYQLGTGQDEDAWSPVEMMGKQLENRVVLSVSSGGQHTVLLVKDKEQS

111100000000000000000000000000000000000000000000000000000000000000000000000000000000000000000000000000000000000000000000000000000000000000000000000000000000000000000000000000000000000000000000000000000000000000000111111000000000000000000000000000000000000000000000000000000000000000000000000000000000000000000000000000000000000000000000000000000000000000000000000000000000000000000000000000000000001111

>DM_train401

EKPGETKEVHPQLTTFRCTKRGGCKPATNFIVLDSLSHPIHRAEGLGPGGCGDWGNPPPKDVCPDVESCAKNCIMEGIPDYSQYGVTTNGTSLRLQHILPDGRVPSPRVYLLDKTKRRYEMLHLTGFEFTFDVDATKLPCGMNSALYLSEMHPTGAKSKYNPGGAYYGTGYCDAQCFVTPFINGLGNIEGKGSCCNSMDIWEANSRASHVAPHTCNKKGLYLCEGEECAFEGVCDKNGCGWNNYRVNVTDYYGRGEEFKVNTLKPFTVVTQFLANRRGKLEKIHRFYVQDGKVIESFYTNKEGVPYTNMIDDEFCEATGSRKYMELGATQGMGEALTRGMVLAMSIWWDQGGNMEWLDHGEAGPCAKGEGAPSNIVQVEPFPEVTYTNLRWGEIGSTYQELQ

000000000000000000000000000000000000000000000000000000000000000000000000000000000000000000000000000000000000000000000000000000000000000000000000000000000000000000000000000000000000000000000000000000000000000000000000000000000000000000000000000000000000000000000000000000000000000000000000000000000000000000000000000000000000000000000000000000000000000000000000000000000000000000000000000000000000000111

>DM_train402

GSMRICIFMARGLEGCGVTKFSLEQRDWFIKNGHEVTLVYAKDKSFTRTSSHDHKSFSIPVILAKEYDKALKLVNDCDILIINSVPATSVQEATINNYKKLLDNIKPSIRVVVYQHDHSVLSLRRNLGLEETVRRADVIFSHSDNGDFNKVLMKEWYPETVSLFDDIEEAPTVYNFQPPMDIVKVRSTYWKDVSEINMNINRWIGRTTTWKGFYQMFDFHEKFLKPAGKSTVMEGLERSPAFIAIKEKGIPYEYYGNREIDKMNLAPNQPAQILDCYINSEMLERMSKSGFGYQLSKLNQKYLQRSLEYTHLELGACGTIPVFWKSTGENLKFRVDNTPLTSHDSGIIWFDENDMESTFERIKELSSDRALYDREREKAYEFLYQHQDSSFCFKEQFDIITK

000000000000000000000000000000000000000000000000000000000000000000000000000000000000000000000000000000000000000000000000000000000000000000000001111111111111111111111111111100000000000000000000000000000000000000000000000000000000000000000000000000000000000000000000000000000000000000000000000000000000000000000000000000000000000000000000000000000000000000000000000000000000000000000000000000000000000000

>DM_train403

MSEPIDILIAGAGIGGLSCALALHQAGIGKVTLLESSSEIRPLGVGINIQPAAVEALAELGLGPALAATAIPTHELRYIDQSGATVWSEPRGVEAGNAYPQYSIHRGELQMILLAAVRERLGQQAVRTGLGVERIEERDGRVLIGARDGHGKPQALGADVLVGADGIHSAVRAHLHPDQRPLSHGGITMWRGVTEFDRFLDGKTMIVANDEHWSRLVAYPISARHAAEGKSLVNWVCMVPSAAVGQLDNEADWNRDGRLEDVLPFFADWDLGWFDIRDLLTRNQLILQYPMVDRDPLPHWGRGRITLLGDAAHLMYPMGANGASQAILDGIELAAALARNADVAAALREYEEARRPTANKIILANREREKEEWAAASRPKTEKSAALEAITGSYRNQVERPR

111000000000000000000000000000000000000000000000000000000000000000000000000000000000000000000000000000000000000000000000000000000000000000000000000000000000000000000000000000000000000000000000000000000000000000000000000000000000000000000000000000000000000000000000000000000000000000000000000000000000000000000000000000000000000000000000000000000000000000000000000000000000000000011111111111111111110000

>DM_train404

MQRPSDQTAPGTSSRPILSAKEAQNFDAQHYFASLTPGAAAWNPSPITLPAQPDFVVGPAGTQGVTHTTIQAAVDAAIIKRTNKRQYIAVMPGEYQGTVYVPAAPGGITLYGTGEKPIDVKIGLSLDGGMSPADWRHDVNPRGKYMPGKPAWYMYDSCQSKRSDSIGVLCSAVFWSQNNGLQLQNLTIENTLGDSVDAGNHPAVALRTDGDQVQINNVNILGRQNTFFVTNSGVQNRLETNRQPRTLVTNSYIEGDVDIVSGRGAVVFDNTEFRVVNSRTQQEAYVFAPATLSNIYYGFLAVNSRFNAFGDGVAQLGRSLDVDANTNGQVVIRDSAINEGFNTAKPWADAVISNRPFAGNTGSVDDNDEIQRNLNDTNYNRMWEYNNRGVGSKVVAEAKK

0000000000000000000000000000000000000000000000000000000000000000000000000000000000000000000000000000000000000000000000000000000000000000000000000000000000000000000000000000000000000000000000000000000000000000000000000000000000000000000000000000000000000000000000000000000000000000000000000000000000000000000000000000000000000000000000000000000000000000000000000000000000000000000000000000000001111111

>DM_train405

SSLDDKPQFPGASAEFIDKLEFIQPNVISGIPIYRVMDRQGQIINPSEDPHLPKEKVLKLYKSMTLLNTMDRILYESQRQGRISFYMTNYGEEGTHVGSAAALDNTDLVFGQAREAGVLMYRDYPLELFMAQCYGNISDLGKGRQMPVHYGCKERHFVTISSPLATQIPQAVGAAYAAKRANANRVVICYFGEGAASEGDAHAGFNFAATLECPIIFFCRNNGYAISTPTSEQYRGDGIAARGPGYGIMSIRVDGNDVFAVYNATKEARRRAVAENQPFLIEAMTYRIGHASTSDDSSAFRSVDEVNYWDKQDHPISRLRHYLLSQGWWDEEQEKAWRKQSRRKVMEAFEQAERKPKPNPNLLFSDVYQEMPAQLRKQQESLARHLQTYGEHYPLDHFDK

1111100000000000000000000000000000000000000000000000000000000000000000000000000000000000000000000000000000000000000000000000000000000000000000000000000000000000000000000000000000000000000000000000000000000000000000000000000000000000000000000000000000000000000000000000000000000000000000000110011111111111111111110000000000000000000000000000000000000000000000000000000000000000000000000000000000000000

>DM_train406

MGVSPAAPASGIRRPCYLVLSSHDFRTPRRANIHFITDQLALRGTTRFFSLRYSRLSRMKGDMRLPLDDTANTVVSHNGVDCYLWRTTVHPFNTRRSWLRPVEDAMFRWYAAHPPKQLLDWMRESDVIVFESGIAVAFIELAKRVNPAAKLVYRASDGLSTINVASYIEREFDRVAPTLDVIALVSPAMAAEVVSRDNVFHVGHGVDHNLDQLGDPSPYAEGIHAVAVGSMLFDPEFFVVASKAFPQVTFHVIGSGMGRHPGYGDNVIVYGEMKHAQTIGYIKHARFGIAPYASEQVPVYLADSSMKLLQYDFFGLPAVCPNAVVGPYKSRFGYTPGNADSVIAAITQALEAPRVRYRQCLNWSDTTDRVLDPRAYPETRLYPHPPTAAPQLSSEAALSH

1111111111110000000000000000000000000000000000000000000000000000000000000000000000000000000000000000000000000000000000000000000000000000000000000000000000000000000000000000000000000000000000000000000000000000000000000000000000000000000000000000000000000000000000000000000000000000000000000000000000000000000000000000000000000000000000000000000000000000000000000000000000000000000000000111111111111111

>DM_train407

MTALKVGSESWWQSKHGPEWQRLNDEMFEVTFWWRDPQGSEEYSTIKRVWVYITGVTDHHQNSQPQSMQRIAGTDVWQWTTQLNANWRGSYCFIPTERDDIFSAPSPDRLELREGWRKLLPQAIADPLNPQSWKGGLGHAVSALEMPQAPLQPGWDCPQAPEIPAKEIIWKSERLKNSRRVWIFTTGDVTAEERPLAVLLDGEFWAQSMPVWPVLTSLTHRQQLPPAVYVLIDAIDTTHRAHELPCNADFWLAVQQELLPLVKVIAPFSDRADRTVVAGQSFGGLSALYAGLHWPERFGCVLSQSGSYWWPHRGGQQEGVLLEKLKAGEVSAEGLRIVLEAGIREPMIMRANQALYAQLHPIKESIFWRQVDGGHDALCWRGGLMQGLIDLWQPLFHDRS

1111100000000000000000000000000000000000000000000000000001111111000000000000000000000000000000000000000001100000000000000000000000000000000000000000000000000000000000000000000000000000001111100000000000000000000000000000000000000000000000000000000000000000000000000000000000000000000000000000000000000000000000000000000000000000000000000000000000000000000000000000000000000000000000000000000000000111

>DM_train408

MKTITLYLDPASLPALNQLMDFTQNNEDKTHPRIFGLSRFKIPDNIITQYQNIHFVELKDNRPTEALFTILDQYPGNIELNIHLNIAHSVQLIRPILAYRFKHLDRVSIQQLNLYDDGSMEYVDLEKEENKDISAEIKQAEKQLSHYLLTGKIKFDNPTIARYVWQSAFPVKYHFLSTDYFEKAEFLQPLKEYLAENYQKMDWTAYQQLTPEQQAFYLTLVGFNDEVKQSLEVQQAKFIFTGTTTWEGNTDVREYYAQQQLNLLNHFTQAEGDLFIGDHYKIYFKGHPRGGEINDYILNNAKNITNIPANISFEVLMMTGLLPDKVGGVASSLYFSLPKEKISHIIFTSNKQVKSKEDALNNPYVKVMRRLGIIDESQVIFWDSLKQLGGGLEHHHHHH

000000000000000000000000000000000000000000000000000000000000000000000000000000000000000000000000000000000000000000000000000000000000000000000000000000000000000000000000000000000000000000000000000000000000000000000000000000000000000000000000000000000000000000000000000000000000000000000000000000000000000000000000000000000000000000000000000000000000000000000000000000000000000000000000000000111111111

>DM_train409

TMAHHHHHHVTNSTDGRADGRLRVVVLGSTGSIGTQALQVIADNPDRFEVVGLAAGGAHLDTLLRQRAQTGVTNIAVADEHAAQRVGDIPYHGSDAATRLVEQTEADVVLNALVGALGLRPTLAALKTGARLALANKESLVAGGSLVLRAARPGQIVPVDSEHSALAQCLRGGTPDEVAKLVLTASGGPFRGWSAADLEHVTPEQAGAHPTWSMGPMNTLNSASLVNKGLEVIETHLLFGIPYDRIDVVVHPQSIIHSMVTFIDGSTIAQASPPDMKLPISLALGWPRRVSGAAAACDFHTASSWEFEPLDTDVFPAVELARQAGVAGGCMTAVYNAANEEAAAAFLAGRIGFPAIVGIIADVLHAADQWAVEPATVDDVLDAQRWARERAQRAVSGM

11111111111111111110000000000000000000000000000000000000000000000000000000000000000000000000000000000000000000000000000000000000000000000000000000000000000000000000000000000000000000000000000000000000000000000000000000000000000000000000000000000000000000000000000000000000000000000000000000000000000000000000000000000000000000000000000000000000000000000000000000000000000000000000000000000000000000

>DM_train410

MAYDVARVRGLHPSLGDGWVHFDAPAGMLIPDSVATTVSTAFRRSGASTVGAHPSARRSAAVLDAAREAVADLVNADPGGVVLGADRAVLLSLLAEASSSRAGLGYEVIVSRLDDEANIAPWLRAAHRYGAKVKWAEVDIETGELPTWQWESLISKSTRLVAVNSASGTLGGVTDLRAMTKLVHDVGALVVVDHSAAAPYRLLDIRETDADVVTVNAHAWGGPPIGAMVFRDPSVMNSFGSVSTNPYATGPARLEIGVHQFGLLAGVVASIEYLAALDESARGSRRERLAVSMQSADAYLNRVFDYLMVSLRSLPLVMLIGRPEAQIPVVSFAVHKVPADRVVQRLADNGILAIANTGSRVLDVLGVNDVGGAVTVGLAHYSTMAEVDQLVRALASLG

11000000000000000000000000000000000000000000000000000000000000000000000000000000000000000000000000000000000000000000000000000000000000000000000000000000000000000000000000000000000000000000000000000000000000000000000000000000000000000000000000000000000000000000000000000000000000000000000000000000000000000000000000000000000000000000000000000000000000000000000000000000000000000000000000000000000000

>DM_train411

MSKVFIATANAGKAHDADIFSVSACNSFTVSCSGDGYLKVWDNKLLDNENPKDKSYSHFVHKSGLHHVDVLQAIERDAFELCLVATTSFSGDLLFYRITREDETKKVIFEKLDLLDSDMKKHSFWALKWGASNDRLLSHRLVATDVKGTTYIWKFHPFADESNSLTLNWSPTLELQGTVESPMTPSQFATSVDISERGLIATGFNNGTVQISELSTLRPLYNFESQHSMINNSNSIRSVKFSPQGSLLAIAHDSNSFGCITLYETEFGERIGSLSVPTHSSQASLGEFAHSSWVMSLSFNDSGETLCSAGWDGKLRFWDVKTKERITTLNMHCDDIEIEEDILAVDEHGDSLAEPGVFDVKFLKKGWRSGMGADLNESLCCVCLDRSIRWFREAGGK

1100000000000000000000000000000000000000000000000000000000000000000000000000000000000000000000000000000000000000000000000000000000000111100000000000000000000000000000000000000000000000000000000000000000000000000000000000000000011100000000000000000000000000000000000000000000000111111110000000000000000000000000000000000000000000000000000000000000000000000000000000000000000000000000000000000000011

>DM_train412

APARGTLLTSNFLTSYTRDAISAMLASGSQPASGSQPEQAKCNVRVAEFTYATIGVEGEPATASGVLLIPGGERCSGPYPLLGWGHPTEALRAQEQAKEIRDAKGDDPLVTRLASQGYVVVGSDYLGLGKSNYAYHPYLHSASEASATIDAMRAARSVLQHLKTPLSGKVMLSGYSQGGHTAMATQREIEAHLSKEFHLVASAPISGPYALEQTFLDSWSGSNAVGENTFGILLGSYAIVAMQHTYKNIYLEPGQVFQDPWAAKVEPLFPGKQSLTDMFLNDTLPSIDKVKSYFQPGFYSDFPSNPANPFRQDLARNNLLEWAPQTPTLLCGSSNDATVPLKNAQTAIASFQQRGSNQVALVDTGTGNASDNSAFAHMLTKESCIVVVRDQLLDKQR

1000000000000000000000000001111111110000000000000000000000000000000000000000000000000000000000000000000000000000000000000000000000000000000000000000000000000000000000000000000000000000000000000000000000000000000000000000000000000000000000000000000000000000000000000000000000000000000000000000000000000000000000000000000000000000000000000000000000000000000000000000000000000000000000000000000000000

>DM_train413

MSLVVFPFKHEHPEVLLHNVRVAAAHPRVHEVLCIGYERDQTYEAVERAAPEISRATGTPVSVRLQERLGTLRPGKGDGMNTALRYFLEETQWERIHFYDADITSFGPDWITKAEEAADFGYGLVRHYFPRASTDAMITWMITRTGFALLWPHTELSWIEQPLGGELLMRREVAAMLYEDERVRRRSDWGIDTLYTFVTVQQGVSIYECYIPEGKAHRLYGGLDDLRTMLVECFAAIQSLQHEVVGQPAIHRQEHPHRVPVHIAERVGYDVEATLHRLMQHWTPRQVELLELFTTPVREGLRTCQRRPAFNFMDEMAWAATYHVLLEHFQPGDPDWEELLFKLWTTRVLNYTMTVALRGYDYAQQYLYRMLGRYRYQAALENGRGHPVPPRAALSTA

1000000000000000000000000000000000000000000000000000000000000000000000000000000000000000000000000000000000000000000000000000000000000000000000000000000000000000000000000000000000000000000000000000000000000000000000000000000000000000000000000000000000000000000000000000000000000000000000000000000000000000000000000000000000000000000000000000000000000000000000000000000000000000000001111111111111111

>DM_train414

MGVTKTPLYETLNESSAVALAVKLGLFPSKSTLTCQEIGDGNLNYVFHIYDQEHDRALIIKQAVPYAKVVGESWPLTIDRARIESSALIRQGEHVPHLVPRVFYSDTEMAVTVMEDLSHLKIARKGLIEGENYPHLSQHIGEFLGKTLFYSSDYALEPKVKKQLVKQFTNPELCDITERLVFTDPFFDHDTNDFEEELRPFVEKLWNNDSVKIEAAKLKKSFLTSAETLIHGDLHTGSIFASEHETKVIDPEFAFYGPIGFDVGQFIANLFLNALSRDGADREPLYEHVNQVWETFEETFSEAWQKDSLDVYANIDGYLTDTLSHIFEEAIGFAGCELIRRTIGLAHVADLDTIVPFDKRIGRKRLALETGTAFIEKRSEFKTITDVIELFKLLVKE

1111110000000000000000000011111110000000000000000011111000000000011111111100000000000000000000000000000000000000000000000000000000000000000000000000000000000000000000000000000000000000000000000000000000000000000000000000000000000000000000000000000000000000000000000000000000000000000000000000000000000000000000000000000000000000000000000000000000000000000000000000000000000000000000000000000000001

>DM_train416

MSPLRKTVPEFLAHLKSLPISKIASNDVLTICVGNESADMDSIASAITYSYCQYIYNEGTYSEEKKKGSFIVPIIDIPREDLSLRRDVMYVLEKLKIKEEELFFIEDLKSLKQNVSQGTELNSYLVDNNDTPKNLKNYIDNVVGIIDHHFDLQKHLDAEPRIVKVSGSCSSLVFNYWYEKLQGDREVVMNIAPLLMGAILIDTSNMRRKVEESDKLAIERCQAVLSGAVNEVSAQGLEDSSEFYKEIKSRKNDIKGFSVSDILKKDYKQFNFQGKGHKGLEIGLSSIVKRMSWLFNEHGGEADFVNQCRRFQAERGLDVLVLLTSWRKAGDSHRELVILGDSNVVRELIERVSDKLQLQLFGGNLDGGVAMFKQLNVEATRKQVVPYLEEAYSNLEE

1110000000000000000000000000000000000000000000000000000000000000000000000000000000000000000000000000000000000000000000000000000000000000000000000000000000000000000000000000000000000000000000000000000000000000000000000000000000000000000000000000000000000000000000000000000001111100000000000000000000000000000000000000000000000000000000000000000000000000000000000000000000000000000000000000000000000

>DM_train417

GMETYDVLVVGGGPGGSTAARYAAKYGLKTLMIEKRPEIGSPVRCGEGLSKGILNEADIKADRSFIANEVKGARIYGPSEKRPIILQSEKAGNEVGYVLERDKFDKHLAALAAKAGADVWVKSPALGVIKENGKVAGAKIRHNNEIVDVRAKMVIAADGFESEFGRWAGLKSVILARNDIISALQYRMINVDVDPDYTDFYLGSIAPAGYIWVFPKGEGMANVGIGSSINWIHNRFELKNYLDRFIENHPGLKKGQDIQLVTGGVSVSKVKMPITMPGLMLVGDAARLIDPITGGGIANAIVSGMYAAQVTKEAIESNDYSPQMMQKYEKLIKERFERKHLRNWVAKEKLAMLSDDTLDKLVDIVSEQVLTTISVEAILKAIAEKYPEVVKELEDLI

0000000000000000000000000000000000000000000000000000000000000000000000000000000000000001111100000000000000000000000000000000000000000000000000000000000000000000000000000000000000000000000000000000000000000000000000000000000000000000000000000000000000000000000000000000000000000000000000000000000000000000000000000000000000000000000000000000000000000000000000000000000000000000000000000001111111111

>DM_train418

TTLLNPYFGEFGGMYVPQILMPALNQLEEAFVRAQKDPEFQAQFADLLKNYAGRPTALTKCQNITAGTRTTLYLKREDLLHGGAHKTNQVLGQALLAKRMGKSEIIAETGAGQHGVASALASALLGLKCRIYMGAKDVERQSPNVFRMRLMGAEVIPVHSGSATLKDACNEALRDWSGSYETAHYMLGTAAGPHPYPTIVREFQRMIGEETKAQILDKEGRLPDAVIACVGGGSNAIGMFADFINDTSVGLIGVEPGGHGIETGEHGAPLKHGRVGIYFGMKAPMMQTADGQIEESYSISAGLDFPSVGPQHAYLNSIGRADYVSITDDEALEAFKTLCRHEGIIPALESSHALAHALKMMREQPEKEQLLVVNLSGRGDKDIFTVHDILKARGEI

000000000000000000000000000000000000000000000000000000000000000000000000000000000000000000000000000000000000000000000000000000000000000001111000000000000000111111100000000000000000000000000000000000000000000000000000000000000000000000000000000000000000000000000000000000000000000000000000000000000000000000000000000000000000000000000000000000000000000000000000000000000000000000000000000111111111

>DM_train419

PQDLMVTCTAPVNIAVIKYWGKRDEALILPINSSLSVTLHQDQLKTTTTVAISKDFTEDRIWLNGREEDVGQPRLQACLREIRRLARKRRSTEDGDTLPLSLSYKVHVASVNNFPTAAGLASSAAGYACLAYTLAQVYGVEGDLSEVARRGSGSACRSLYGGFVEWQMGEQADGKDSIARQIAPEWHWPQLRILILVVSADKKQTGSTVGMQTSVETSTLLKFRAESVVPERMKEMTRCIQEQDFQGFAQLTMKDSNQFHATCLDTFPPISYLNDTSRRIIQLVHRFNTHHGQTKVAYTFDAGPNAVIFTLEDTVAEFVAAVRHSFPPAANGDKFLKGLQVAPVLLSDELKAALVVEPSPGGVQYIIATQVGPGPQVLDDTHDHLLGQDGLPQRDL

110000000000000000000000000000000000000000000000000000000000000000000000000000000000000000111111111000000000000000011111000000000000000000000000000000000000000000000000000000000000000000000000000000000000000000000000000000000000000000000000000000000000000000000000000000000000000000000000000000000000000000000000000000000000000000011000000000000000000000011000000000000000000000000000000000000111

>DM_train420

MFENITAAPADPILGLADLFRADERPGKINLGIGVYKDETGKTPVLTSVKKAEQYLLENETTKNYLGIDGIPEFGRCTQELLFGKGSALINDKRARTAQTPGGTGALRVAADFLAKNTSVKRVWVSNPSWPNHKSVFNSAGLEVREYAYYDAENHTLDFDALINSLNEAQAGDVVLFHGCCHNPTGIDPTLEQWQTLAQLSVEKGWLPLFDFAYQGFARGLEEDAEGLRAFAAMHKELIVASSYSKNFGLYNERVGACTLVAADSETVDRAFSQMKAAIRANYSNPPAHGASVVATILSNDALRAIWEQELTDMRQRIQRMRQLFVNTLQEKGANRDFSFIIKQNGMFSFSGLTKEQVLRLREEFGVYAVASGRVNVAGMTPDNMAPLCEAIVAVL

000000000011111111111111111000000000000000000000000000000000000000000000000000000000000000000000000000000000000000000000000000000000000000000000000000000000000000000000000000000000000000000000000000000000000000000000000000000000000000000000000000000000000000000000000000000000000000000000000000000000000000000000000000000000000000000000000000000000000000000000000000000000000000000000000000000000

>DM_train421

MSQPQSSQVTKRGLTDPERAAIIAAAVPDHALDTQRKYHYFIQPRWKPLSEYEQLSCYAQPNPDWIAGGLDWGDWTQKFHGGRPSWGNESTELRTTDWYRHRDPARRWHHPYVKDKSEEARYTQRFLAAYSSEGSIRTIDPYWRDEILNKYFGALLYSEYGLFNAHSSVGRDCLSDTIRQTAVFAALDKVDNAQMIQMERLFIAKLVPGFDASTDVPKKIWTTDPIYSGARATVQEIWQGVQDWNEILWAGHAVYDATFGQFARREFFQRLATVYGDTLTPFFTAQSQTYFQTTRGAIDDLFVYCLANDSEFGAHNRTFLNAWTEHYLASSVAALKDFVGLYAKVEKVAGATDSAGVSEALQRVFGDWKIDYADKIGFRVDVDQKVDAVLAGYKN

11111111110000000000000000000000000000000000000000000000000000000000000000000000000000000000000000000000000000000000000000000000000000000000000000000000000000000000000000000000000000000000000000000000000000000000000000000000000000000000000000000000000000000000000000000000000000000000000000000000000000000000000000000000000000000000000000000000000000000000000000000000000000000000000000000000000

>DM_train422

MFAKFPSSASISPNPFTVSIPDEQLDDLKTLVRLSKIAPPTYESLQADGRFGITSEWLTTMREKWLSEFDWRPFEARLNSFPQFTTEIEGLTIHFAALFSEREDAVPIALLHGWPGSFVEFYPILQLFREEYTPETLPFHLVVPSLPGYTFSSGPPLDKDFGLMDNARVVDQLMKDLGFGSGYIIQGGDIGSFVGRLLGVGFDACKAVHLNFCNMSAPPEGPSIESLSAAEKEGIARMEKFMTDGYAYAMEHSTRPSTIGHVLSSSPIALLAWIGEKYLQWVDKPLPSETILEMVSLYWLTESFPRAIHTYREWVPTASAPNGATPYQKELYIHKPFGFSFFPKDLVPVPRSWIATTGNLVFFRDHAEGGHFAALERPRELKTDLTAFVEQVWQK

10000000000000000000000000000000000000000000000000000000000000000000000000000000000000000000000000000000000000000000000000000000000000000000000000000000000000000000000000000000000000000000000000000000000000000000000000011111100000000000000000000000000000000000000000000000000000000000000000000000000000000000000000011111111000000000000000000000000000000000000000000000000000000000000000000000011

>DM_train424

AQVQQLTPAQQAALRNQQAMAANLQARQIVLQQSYPVIQQVETQTFDPANRSVFDVTPANVGIVKGFLVKVTAAITNNHATEAVALTDFGPANLVQRVIYYDPDNQRHTETSGWHLHFVNTAKQGAPFLSSMVTDSPIKYGDVMNVIDAPATIAAGATGELTMYYWVPLAYSETDLTGAVLANVPQSKQRLKLEFANNNTAFAAVGANPLEAIYQGAGAADCEFEEISYTVYQSYLDQLPVGQNGYILPLIDLSTLYNLENSAQAGLTPNVDFVVQYANLYRYLSTIAVFDNGGSFNAGTDINYLSQRTANFSDTRKLDPKTWAAQTRRRIATDFPKGVYYCDNRDKPIYTLQYGNVGFVVNPKTVNQNARLLMGYEYFTSRTELVNAGTISTT

1111111111111000000000000000000000000000000000000000000000000000000000000000000000000000000000000000000000000000000000000000000000000000000000000000000000000000000000000000000000000000000000000000000000000000000000000000000000000000000000000000000000000000000000000000000000000000000000000000000000000000000000000000000000000000000000000000000000000000000000000000000000000000000000111111111111

>DM_train425

APLASVPTPAFPFPVPTMVHGPCVAESEPALLTGSKQFGLSRNSHIAIAFDDTKVKNRLTIELEVRTEAESGLLFYMARINHADFATVQLRNGFPYFSYDLGSGDTSTMIPTKINDGQWHKIKIVRVKQEGILYVDDASSQTISPKKADILDVVGILYVGGLPINYTTRRIGPVTYSLDGCVRNLHMEQAPVDLDQPTSSFHVGTCFANAESGTYFDGTGFAKAVGGFKVGLDLLVEFEFRTTRPTGVLLGVSSQKMDGMGIEMIDEKLMFHVDNGAGRFTAIYDAEIPGHMCNGQWHKVTAKKIKNRLELVVDGNQVDAQSPNSASTSADTNDPVFVGGFPGGLNQFGLTTNIRFRGCIRSLKLTKGTGKPLEVNFAKALELRGVQPVSCPTT

1111111111111111111000000000000000000000000000000000000000000000000000000000000000000000000000000000000000000000000000000000000000000000000000000000000000000000000000000000000000000000000000000000000000000000000000000000000000000000000000000000000000000000000000000000000000000000000000000000000000000000000000000000000000000000000000000000000000000000000000000000000000000000000000000000000000

>DM_train426

MKTQVAIIGAGPSGLLLGQLLHKAGIDNVILERQTPDYVLGKIRAGVLEQGMVDLLREAGVDRRMARDGLVHEGVEIAFAGQRRRIDLKRLSGGKTVTVYGQTEVTRDLMEAREASGATTVYQAAEVRLHDLQGERPYVTFERDGERLRLDCDYIAGCDGFHGISRQSIPAERLKVFERVYPFGWLGLLADTPPVSHELIYANHPRGFALCSQRSATRSRYYVQVPLTEKVEDWSDERFWTELKARLPAEVAEKLVTGPSLEKSIAPLRSFVVEPMQHGRLFLAGDAAHIVPPTGAKGLNLAASDVSTLYRLLLKAYREGRGELLERYSAICLRRIWKAERFSWWMTSVLHRFPDTDAFSQRIQQTELEYYLGSEAGLATIAENYVGLPYEEIE

0000000000000000000000000000000000000000000000000000000000000000000000000000000000000000000000000000000000000000000000000000000000000000000000000000000000000000000000000000000000000000000000000000000000000000000000000000000000000000000000000000000000000000000000000000000000000000000000000000000000000000000000000000000000000000000000000000000000000000000000000000000000000000000000000000000111

>DM_train428

GMSSSDRYMQRGVSSQKEDVHKAIKSIDKGIYPRAFCKIIPDILGGDPEYCNIMHADGAGTKSSLAYVYWKETGDISVWKGIAQDAVIMNIDDLICVGAVDNILLSSTIGRNKNLIPGEVLAAIINGTEEVLQMLRDNGIGIYSTGGETADVGDLVRTIIVDSTVTCRMKRQDVISNENIKAGNVIVGFASYGQTSYETEYNGGMGSNGLTSARHDVFNNVLASKYPESFDPKVPENLVYSGEMNLTDPYLNVPLDAGKLVLSPTRTYAPLMKEIIHQYKGKLDGVVHCSGGGQTKVLHFTDATTHIIKDNLFDVPPLFQLIQGQSNTPWEEMYKVFNMGHRLEIYTDAAHAEGMIAIAKKFNIEAKIIGRVEAPVAGKRLTITGPQGTEYTYA

1111111111111111111111111110000000000000000000000000000000000000000000000000000000000000000000000000000000000000000000000000000000000000000000000000000000000000000000000000000000000000000000000000000000000000000000000000000000000000000000000000000000000000000000000000000000000000000000000000000000000000000000000000000000000000000000000000000000000000000000000000000000000000000000000000000000

>DM_train429

MRCIGISNRDFVEGVSGGSWVDIVLEHGSCVTTMAKNKPTLDFELIKTEAKQPATLRKYCIEAKLTNTTTESRCPTQGEPTLNEEQDKRFVCKHSMVDRGWGNGCGLFGKGGIVTCAMFTCKKNMEGKIVQPENLEYTVVITPHSGEEHAVGNDTGKHGKEVKITPQSSITEAELTGYGTVTMECSPRTGLDFNEMVLLQMKDKAWLVHRQWFLDLPLPWLPGADTQGSNWIQKETLVTFKNPHAKKQDVVVLGSQEGAMHTALTGATEIQMSSGNLLFTGHLKCRLRMDKLQLKGMSYSMCTGKFKVVKEIAETQHGTIVIRVQYEGDGSPCKIPFEIMDLEKRHVLGRLITVNPIVTEKDSPVNIEAEPPFGDSYIIIGVEPGQLKLNWFKK

0000000000000000000000000000000000000000000000000000000000000000000000000000000000000000000000000000000000000000000000000000000000000000000000001111111111111100000000000000000000000000000000000000000000000000000000000000000000000000000000000000000000000000000000000000000000000000000000000000000000000000000000000000000000000000000000000000000000000000000000000000000000000000000000000000000000

>DM_train430

MKLEIFSWWAGDEGPALEALIRLYKQKYPGVEVINATVTGGAGVNARAVLKTRMLGGDPPDTFQVHAGMELIGTWVVANRMEDLSALFRQEGWLQAFPKGLIDLISYKGGIWSVPVNIHRSNVMWYLPAKLKGWGVNPPRTWDKFLATCQTLKQKGLEAPLALGENWTQQHLWESVALAVLGPDDWNNLWNGKLKFTDPKAVRAWEVFGRVLDCANKDAAGLSWQQAVDRVVQGKAAFNIMGDWAAGYMTTTLKLKPGTDFAWAPSPGTQGVFMMLSDSFGLPKGAKNRQNAINWLRLVGSKEGQDTSNPLKGSIAARLDSDPSKYNAYGQSAMRDWRSNRIVGSLVHGAVAPESFMSQFGTVMEIFLQTRNPQAAANAAQAIADQVGLGRLGQ

0000000000000000000000000000000000000000000000000000000000000000000000000000000000000000000000000000000000000000000000000000000000000000000000000000000000000000000000000000000000000000000000000000000000000000000000000000000000000000000000000000000000000000000000000000000000000000000000000000000000000000000000000000000000000000000000000000000000000000000000000000000000000000000000000000000111

>DM_train431

MGSSSSGRENLYFQGMASIVFSTIGNPKGYQKVTYEIDGEKFESNVSVLALRDLLKVDKTVVILGISVADVYNCKYADYRSCKECIIQNSKNDLGISESYVVAPNVYQKFKGKPDHYFTYIYYHSLRILEKEGINEVFIDTTHGINYMGVLAKEAIQLAVSAYAAKSEKEVKVSLYNSDPVGKDVSDTVKLHEIEAIKISPLSGLKYVTYQILNKDKNFFNKIFSDSVNAIPRFATALDNGLFIYLSEKDSSLHLKRLEDDLSKDPLLTPSENEINVVYKDMKYALSHALFYVISRFSGNVDLDTLRHYAETYADKVTRAIIENEVDKIEKYQMGSERKLLGEYMKVEGKGFDKRILYAHGGLPYAGTYVYKEKDKVYVTYGDKIDEIERQIGS

1111111111110000000000000000000000000000000000000000000000000000000000000000000000000000000000000000000000000000000000000000000000000000000000000000000000000000000000000000000000000000000000000000000000000000000000000000000000000000000000000000000000000000000000000000000000000000000000000000000000000000000000000000000000000000000000000000000001111111110000000000000000000000000000000000000011

>DM_train432

IESLCMNCYRNGTTRLLLTKIPFFREIIVSSFSCEHCGWNNTEIQSAGRIQDQGVRYTLTVRSQEDMNREVVKTDSATTRIPELDFEIPAFSQKGALTTVEGLISRAISGLEQDQPTRRAVEGAIAERIDEFIGKLKDLKQMASPFTLVIDDPSGNSFVENPHAPQKDNALVITYYDRTPQQAEMLGLQAEAPEEKAEEEDLRNEVLQFNTNCPECNAPAQTNMKLVQIPHFKEVIIMATNCENCGHRTNEVKSGGAVEPLGTRITLHITDPSDMTRDLLKSETCSVEIPELEFELGMAVLGGKFTTLEGLLKDIRELVTKNPFTLGDSSNPDQSEKLQEFSQKLGQIIEGKMKAHFIMNDPAGNSYLQNVYAPEDDPEMKVERYKRTFDQNEE

0000000000000000000000000000000000000000000000000000000000000000000000000000000000000000000000000000000000000000000000000000000000000000000000000000000000000000000000000000000000000000000011111111110000000000000000000000000000000000000000000000000000000000000000000000000000000000000000000000000000000000000000000000000000000000000000000000000000000000000000000000000000000000000000000000000000

>DM_train433

MASMQKLINSVQNYAWGSKTALTELYGIANPQQQPMAELWMGAHPKSSSRITTANGETVSLRDAIEKNKTAMLGEAVANRFGELPFLFKVLCAAQPLSIQVHPNKRNSEIGFAKENAAGIPMDAAERNYKDPNHKPELVFALTPFLAMNAFREFSDIVSLLQPVAGAHSAIAHFLQVPNAERLSQLFASLLNMQGEEKSRALAVLKAALNSQQGEPWQTIRVISEYYPDDSGLFSPLLLNVVKLNPGEAMFLFAETPHAYLQGVALEVMANSDNVLRAGLTPKYIDIPELVANVKFEPKPAGELLTAPVKSGAELDFPIPVDDFAFSLHDLALQETSIGQHSAAILFCVEGEAVLRKDEQRLVLKPGESAFIGADESPVNASGTGRLARVYNKL

0000000000000000000000000000000000000000000000000000111110000000000000000000000000000000000000000000000000000000000000000000000000000000000000000000000000000000000000000000000000000000000000000000000000000000000000000000000000000000000000000000000000000000000000000000000000000000000000000000000000000000000000000000000000000000000000000000000000000000000000000000000000000000000000000000000000

>DM_train434

MLLEAPVYKEIFGAVTIHEVQKVIKMDTETEEVPIYTISNIPREKIYDLLGKMAVIVPMKNEKLHLVDGVLKAIPHKCPIIIVSNSKREGPNRYKLEVDLIRHFYNLTHSKIIMIHQKDPGLAKAFKEVGYTDILDENGMIRSGKGEGMLVGLLLAKAIGAEYVGFVDADNYIPGAVNEYVKDYAAGFLMSESEYTMVRLHWRHKPKVTKGTLYFKKWGRVSEITNHYLNLLVSEHTAFETTIMVTGNAGEHAMTMKLAEILPFSTGYSIEPYEIVYILERFGKWENVEEFKDVFDQGIEIFQIETLNPHFHEDKGKEHVKEMLLLSLATIYHSKLATDNLRKRILKDLRDHGILGENEEPPKPLVMRPIKEIPIKEWMDIVEGNSETLLRFEL

0000000000000000000000000000111111110000000000000000000000000000000000000000000000000000000000000000000000000000000000000000000000000000000000000000000000000000000000000000000000000000000000000000000000111111111111111111000000000000000000000000000000000000000000000000000000000000000000000000000000000000000000000000000000000000000000000000000000000111111111000000000000000000000000000000000000

>DM_train435

MSNPTKISILGRESIIADFGLWRNYVAKDLISDCSSTTYVLVTDTNIGSIYTPSFEEAFRKRAAEITPSPRLLIYNRPPGEVSKSRQTKADIEDWMLSQNPPCGRDTVVIALGGGVIGDLTGFVASTYMRGVRYVQVPTTLLAMVDSSIGGKTAIDTPLGKNLIGAIWQPTKIYIDLEFLETLPVREFINGMAEVIKTAAISSEEEFTALEENAETILKAVRREVTPGEHRFEGTEEILKARILASARHKAYVVSADEREGGLRNLLNWGHSIGHAIEAILTPQILHGECVAIGMVKEAELARHLGILKGVAVSRIVKCLAAYGLPTSLKDARIRKLTAGKHCSVDQLMFNMALDKKNDGPKKKIVLLSAIGTPYETRASVVANEDIRVVLAP

111000000000000000000000000000000000000000000000000000000000000000000000000000000000000000000000000000000000000000000000000000000000000000000000000000000000000000000000000000000000000000000000000000000000000000000000000000000000000000000000000000000000000000000000000000000000000000000000000000000000000000000000000000000000000000000000000000000000000011111111111110000000000000000000000000000

>DM_train436

MAEGKMMSDFLPFSRPAMGAEELAAVKTVLDSGWITTGPKNQELEAAFCRLTGNQYAVAVSSATAGMHIALMALGIGEGDEVITPSMTWVSTLNMIVLLGANPVMVDVDRDTLMVTPEHIEAAITPQTKAIIPVHYAGAPADLDAIYALGERYGIPVIEDAAHATGTSYKGRHIGARGTAIFSFHAIKNITCAEGGIVVTDNPQFADKLRSLKFHGLGVDAWDRQSGGRAPQAEVLAPGYKYNLPDLNAAIALAQLQKLDALNARRAAIAAQYHQAMADLPFQPLSLPSWEHIHAWHLFIIRVDEARCGITRDALMASLKTKGIGTGLHFRAAHTQKYYRERFPTLTLPDTEWNSERICSLPLFPDMTESDFDRVITALHQIAGQGSHHHHHH

111111110000000000000000000000000000000000000000000000000000000000000000000000000000000000000000000000000000000000000000000000000000000000000000000000000000000000000000000000000000000000000000000000000000000000000000000011111111111000000000000000000000000000000000000000000000000000000000000000000000000000000000000000000000000000000000000000000000000000000000000000000000000000000001111111111

>DM_train437

GAKSEMAFAGKGEISPRAITMWDFSWLERRWPGAGYEDWDQVLDELSERGYNAIRIDAYPHLIAENPMKKWLLKEVWNQQDWGSPDMNEVQVQPNLNLFLSKCKERDIKVGLSSWYRLDVDEVCLKLDTPEKLADCWLTILRSIEEDGLLDTILYVDLCNEWPGDSWAPFFAKTYPNVGWGNWYKEESLRWMKTSLEKMRQVYPDMPFLYSFDHGDVKKYEEVDCSFLDLYEHHIWMAQQNGGEFYKLVGYGYNRFLPDDYKNVVKNAERVYRERPGYWQKLLTDKIELMASVARKNRRPLVTTECWGLVDYKDWPLLKWDWVKDLCELGTITAARTGMWVGVATSNFCGPQFAGMWRDVEWHKRLTSIIRSSPLDESLTKNNEVAAKLLKRL

111111111111000000000000000000000000000000000000000000000000000000000000000000000000000000000000000000000000000000000000000000000000000000000000000000000000000000000000000000000000000000000000000000000000000000000000000000000000000000000000000000000000000000000000000000000000000000000000000000000000000000000000000000000000000000000000000000000000000000000000000000000000000000000000000000000

>DM_train438

RIGIPRERLTNETRVAATPKTVEQLLKLGFTVAVESGAGQLASFDDKAFVQAGAEIVEGNSVWQSEIILKVNAPLDDEIALLNPGTTLVSFIWPAQNPELMQKLAERNVTVMAMDSVPRISRAQSLDALSSMANIAGYRAIVEAAHEFGRFFTGQITAAGKVPPAKVMVIGAGVAGLAAIGAANSLGAIVRAFDTRPEVKEQVQSMGAEFLELDFKEEAGSGDGYAKVMSDAFIKAEMELFAAQAKEVDIIVTTALIPGKPAPKLITREMVDSMKAGSVIVDLAAQNGGNCEYTVPGEIFTTENGVKVIGYTDLPGRLPTQSSQLYGTNLVNLLKLLCKEKDGNITVDFDDVVIRGVTVIRAGEITWPAPPIQVSAQPQAAQKAAPEVKTEEK

000000000000000000000000000000000000000000000000000000000000000000000000000000000000000000000000000000000000000000000000000000000000000000000000000000000000000000000000000000000000000000000000000000000000000000000011111111111111100000000000000000000000000000000000000000000000000000000000000000000000000000000000000000000000000000000000000000000000000000000000000000000000000011111111111111111

>DM_train439

ASASTPHALLLISIDGLRADMLDRGITPNLSHLAREGVRARWMAPSYPSLTFPNHYTLVTGLRPDHHGIVHNSMRDPTLGGFWLSKSEAVGDARWWGGEPVWVGVENTGQHAATWSWPGSEAAIKGVRPSQWRHYQKGVRLDTRVDAVRGWLATDGAQRNRLVTLYFEHVDEAGHDHGPESRQYADAVRAVDAAIGRLLAGMQRDGTRARTNIIVVSDHGMAEVAPGHAISVEDIAPPQIATAITDGQVIGFEPLPGQQAAAEASVLGAHDHYDCWRKAELPARWQYGSHPRIPSLVCQMHEGWDALFPDKLAKRAQRGTRGSHGYDPALPSMRAVFLAQGPDLAQGKTLPGFDNVDVYALMSRLLGIPAAPNDGNPATLLPALRMPPAPDAR

111100000000000000000000000000000000000000000000000000000000000000000000000000000000000000000000000000000000000000000000000000000000000000000000000000000000000000000000000000000000000000000000000000000000000000000000000000000000000000000000000000000000000000000000000000000000000000000000000000000000000000000000000000000000000000000000000000000000000000000000000000000000000000000000001111111

>DM_train441

MTAASLDPTAFSLDAASLAARLDAVFDQALRERRLVGAVAIVARHGEILYRRAQGLADREAGRPMREDTLFRLASVTKPIVALAVLRLVARGELALDAPVTRWLPEFRPRLADGSEPLVTIHHLLTHTSGLGYWLLEGAGSVYDRLGISDGIDLRDFDLDENLRRLASAPLSFAPGSGWQYSLALDVLGAVVERATGQPLAAAVDALVAQPLGMRDCGFVSAEPERFAVPYHDGQPEPVRMRDGIEVPLPEGHGAAVRFAPSRVFEPGAYPSGGAGMYGSADDVLRALEAIRANPGFLPETLADAARRDQAGVGAETRGPGWGFGYLSAVLDDPAAAGTPQHAGTLQWGGVYGHSWFVDRALGLSVLLLTNTAYEGMSGPLTIALRDAVYAR

11111111111111000000000000000000000000000000000000000000000000000000000000000000000000000000000000000000000000000000000000000000000000000000000000000000000000000000000000000000000000000000000000000000000000000000000000000000000000000000000000000000000000000000000000000000000000000000000000000000000000000000000000000000000000000000000000000000000000000000000000000000000000000000000000000000

>DM_train442

MATALYTANDFILISLPQNAQPVTAPGSKTDSWFNETLIGGRAFVSDFKIPEFKIGSLDTLIVESEELSKVDNQIGASIGKIIEILQGLNETSTNAYRTLPINNMPVPEYLENFQWQTRKFKLDKSIKDLITLISNESSQLDADVRATYANYNSAKTNLAAAERKKTGDLSVRSLHDIVKPEDFVLNSEHLTTVLVAVPKSLKSDFEKSYETLSKNVVPASASVIAEDAEYVLFNVHLFKKNVQEFTTAAREKKFIPREFNYSEELIDQLKKEHDSAASLEQSLRVQLVRLAKTAYVDVFINWFHIKALRVYVESVLRYGLPPHFNIKIIAVPPKNLSKCKSELIDAFGFLGGNAFMKDKKGKINKQDTSLHQYASLVDTEYEPFVMYIINL

11110000000000000000000000000000000000000000000000000000000000000000000000000000000000000000000000000000000000000000000000000000000000000000000000000000000000000000000000000000000000000000000000000000000000000000000000000000000000000000000000000000000000000000000000000000000000000000000000000000000000000000000000000000000000000000000000000000000000000000011111111111111111111111100000000000

>DM_train443

GAMGSMKPYLFDLKLKDTEKLDWKKGLSSYLKKSYGSSQWRTFYDEKATSELDHLRNNANGELAPSSLSEQNLKYYSFLEHLYFRLGSKGSRLKMDFTWYDAEYSSAQKGLKYTQHTLAFEKSCTLFNIAVIFTQIARENINEDYKNSIANLTKAFSCFEYLSENFLNSPSVDLQSENTRFLANICHAEAQELFVLKLLNDQISSKQYTLISKLSRATCNLFQKCHDFMKEIDDDVAIYGEPKWKTTVTCKLHFYKSLSAYYHGLHLEEENRVGEAIAFLDFSMQQLISSLPFKTWLVEFIDFDGFKETLEKKQKELIKDNDFIYHESVPAVVQVDSIKALDAIKSPTWEKILEPYMQDVANKYDSLYRGIIPLDVYEKESIYSEEKATLLR

11111000000000000000000000000000000000000000000000000000000000000000000000000000000000000000000000000000000000000000000000000000000000000000000000000000000000000000000000000000000000000000000000000000000000000000000000000000000000000000000000000000000000000000000000000000000000000000000000000000000000000000000000000000000000000000000000000000000000000000000000000000000011111111111111111111

>DM_train444

MNARSTGQHPARYPGAAAGEPTLDSWQEPPHNRWAFAHLGEMVPSAAVSRRPVNAPGHALARLGAIAAQLPDLEQRLEQTYTDAFLVLRGTEVVAEYYRAGFAPDDRHLLMAVSKSLCGTVVGALVDEGRIDPAQPVTEYVPELAGSVYDGPSVLQVLDMQISIDYNEDYVDPASEVQTHDRSAGWRTRRHGDPADTYEFLTTLRGDGSTGEFQYCSANTDVLAWIVERVTGLRYVEALSTYLWAKLDADRDATITVDTTGFGFANGGVSCTARDLARVGRMMLDGGVAPGGRVVSEDWVRRVLAGGSHEAMTDKGFTNTFPDGSYTRQWWCTGNERGNVSGIGIHGQNLWLDPLTDSVIVKLSSWPDPDTEHWHRLQNGILLDVSRALDAV

11110000000000000000000000000000000000000000000000001111000000000000000000000000000000000000000000000000000000000000000000000000000000000000000000000000000000000000000000000000000000000000000000000000000000000000000000000000000000000000000000000000000000000000000000000000000000000000000000000000000000000000000000000000000000000000000000000000000000000000000000000000000000000000000000000000

>DM_train445

TSTATDSPLKYYDIGLNLTDPMFHGIYNGKQYHPADYVKLLERAAQRHVKNALVTGSSIAESQSAIELVSSVKDLSPLKLYHTIGVHPCCVNEFADASQGDKASASIDNPSMDEAYNESLYAKVISNPSFAQGKLKELYDLMNQQAKPHDTSFRSIGEIGLDYDRFHYSSKEMQKVFFEEQLKISCLNDKLSSYPLFLHMRSACDDFVQILERFVVGFTDEKDTFQLQKLGASSSSGFYKFHPDRKLVVHSFTGSAIDLQKLLNLSPNIFIGVNGCSLRTEENLAVVKQIPTERLLLETDAPWCEIKRTHASFQYLAKYQEVRDFEYPAFKSVKKNKLADKLNAEELYMVKGRNEPCNMEQVAIVVSEVKDVDLATLIDTTWKTTCKIFGE

1111111000000000000000000000000000000000000000000000000000000000000000000000000000000000000000011111111111111111100000000000000000000000000000000000000000000000000000000000000000000000000000000000000000000000000000000000000000000011000000000000000000000000000000000000000000000000000000000000000000000000000000000000000000000000000000000000000000000000000000000000000000000000000000000000011

>DM_train446

TSKNQFYKPGMLVPVFSAAGLLKNGRYQFLLQQIETLSLLPTEQYAQLYEALVYRFVEFVQVLPIRLDEPLCSLMNEGLLRGVNSLNHYIQNHPEATPLERYALFSAGLLLEVAHAVVNQKIFITDEEGNFIKQWNPFSGPLIDDVETKHYKIMPLSSYYQRNIPSITPILVRQLLPDEGFLWLTSDMRVFSDWMQALRDDEGEGGGRFEHVLQLFKHKNIDGLFNTLPALPVNLQDSPATAHADAFLNWLKEALATNQIKVNTSDAGVHVIPEGVFLEKTGIFKQYIDLHVNVPVNLFTVYQQFGNLFGLTKLSGIDYRFEQLFSEYPDALKRKSKMGFAGLIGIRRASPTREGVLIADPNLIFTRGEIPSATSYLKLSSAQKSQNIPMT

1110000000000000000000000000000000000000000000000000000000000000000000000000000000000000000000000000000000000000000000000000000000000000000000000000000000000000000000000000000000000000000000000000000001111100000000000000000000000000000000000000000000000000000000000000000000000000000000000000000000000000000000000000000000000111100000000000000111111000000000000000000000000010000111111111111

>DM_train447

PEFFSVRHLELAGDDPYSNVNCTKILQGDPEEIQKVKLEILTVQFKKRPRWTPHDYINMTRDCASFIRTRKYIVEPLTKEEVGFPIAYSIVVHHKIEMLDRLLRAIYMPQNFYCIHVDRKAEESFLAAVQGIASCFDNVFVASQLESVVYASWTRVKADLNCMKDLYRMNANWKYLINLCGMDFPIKTNLEIVRKLKCSTGENNLETEKMPPNKEERWKKRYAVVDGKLTNTGIVKAPPPLKTPLFSGSAYFVVTREYVGYVLENENIQKLMEWAQDTYSPDEFLWATIQRIPEVPGSFPSSNKYDLSDMNAIARFVKWQYFEGDVSNGAPYPPCSGVHVRSVCVFGAGDLSWMLRQHHLFANKFDMDVDPFAIQCLDEHLRRKALENLEH

1111110000011111110000000000000000000111111111100000000000000000000000000000000000000000000000000000000000000000000000000000000000000000000000000000000000000000000000000000000000000000000000000000000000000000000000000000000000000000000000000000000000000000000000000000000000000000000000000000000000000000000000000000000000000000000000000000000000000000000000000000000000000000000000000001111

>DM_train448

YEHSTVMPNVVGFPYKAHIERPGYSPLTLQMQVVETSLEPTLNLEYITCEYKTVVPSPYVKCCGASECSTKEKPDYQCKVYTGVYPFMWGGAYCFCDSENTQLSEAYVDRSDVCRHDHASAYKAHTASLKAKVRVMYGNVNQTVDVYVNGDHAVTIGGTQFIFGPLSSAWTPFDNKIVVYKDEVFNQDFPPYGSGQPGRFGDIQSRTVESNDLYANTALKLARPSPGMVHVPYTQTPSGFKYWLKEKGTALNTKAPFGCQIKTNPVRAMNCAVGNIPVSMNLPDSAFTRIVEAPTIIDLTCTVATCTHSSDFGGVLTLTYKTNKNGDCSVHSHSNVATLQEATAKVKTAGKVTLHFSTASASPSFVVSLCSARATCSASCEPPKDHIVPY

000000000000000000000000000000000000000000000000000000000000000000000000000000000000000000000000000000000000000000000000000000000000000000000000000000000000000000000000000000000000000000000000000000000000000000000000000000000000000000000000000000000000000000000000000000000000000000000000000000000000000000000111110000000000000000000000000000000111111000000000000000000000000000001111111111

>DM_train449

MIDYTAAGFTLLQGAHLYAPEDRGICDVLVANGKIIAVASNIPSDIVPNCTVVDLSGQILCPGFIDQHVHLIGGGGQAGPTTRTPEVALSRLTEAGVTSVVGLLGTDSISRHPESLLAKTRALNEEGISAWMLTGAYHVPSRTITGSVEKDVAIIDRVIGVKCAISDHRSAAPDVYHLANMAAESRVGGLLGGKPGVTVFHMGDSKKALQPIYDLLENCDVPISKLLPTHVNRNVPLFEQALEFARKGGTIDITSSIDEPVAPAEGIARAVQAGIPLARVTLSSDGNGSQPFFDDEGNLTHIGVAGFETLLETVQVLVKDYDFSISDALRPLTSSVAGFLNLTGKGEILPGNDADLLVMTPELRIEQVYARGKLMVKDGKACVKGTFETA

000000000000000000000000000000000000000000000000000000000000000000000000000000000000000000000000000000000000000000000000000000000000000000000000000000000000000000000000000000000000000000000000000000000000000000000000000000000000000000000000000000000000000000000000000000000000000000000000011111111111111000000000000000000000000000000000000000000000000000000000000000000000000000000000000001

>DM_train450

MKSKFKLTTAAAMLGLMVLAGGAQAQDKPREVLTGGHSVSAPQENRIYVMDSVFMHLTESRVHVYDYTNGKFLGMVPTAFNGHVQVSNDGKKIYTMTTYHERITRGKRSDVVEVWDADKLTFEKEISLPPKRVQGLNYDGLFRQTTDGKFIVLQNASPATSIGIVDVAKGDYVEDVTAAAGCWSVIPQPNRPRSFMTICGDGGLLTINLGEDGKVASQSRSKQMFSVKDDPIFIAPALDKDKAHFVSYYGNVYSADFSGDEVKVDGPWSLLNDEDKAKNWVPGGYNLVGLHRASGRMYVFMHPDGKEGTHKFPAAEIWVMDTKTKQRVARIPGRDALSMTIDQQRNLMLTLDGGNVNVYDISQPEPKLLRTIEGAAEASLQVQFHPVGGV

111111111111111111111111111100000000000000000000000000000000000000000000000000000000000000000000000000000000000000000000000000000000000000000000000000000000000000000000000000000000000000000000000000000000000000000000000000000000000000000000000000000000000000000000000000000000000000000000000000000000000000000000000000000000000000000000000000000000000000000000000000000000000000000000000111

>DM_train451

MTESYDVVVVGGGPVGLATAWQVAERGHRVLVLERHTFFNENGGTSGAERHWRLQYTQEDLFRLTLETLPLWRALESRCERRLIHEIGSLWFGDTDVVTNEGQISGTAAMMDKLSVRYEWLKATDIERRFGFRGLPRDYEGFLQPDGGTIDVRGTLAALFTLAQAAGATLRAGETVTELVPDADGVSVTTDRGTYRAGKVVLACGPYTNDLLEPLGARLAYSVYEMAIAAYRQATPVTEAPFWFAFQQPTPQDTNLFYGFGHNPWAPGEFVRCGPDFEVDPLDHPSAATGVADRRQMDRLSGWLRDHLPTVDPDPVRTSTCLAVLPTDPERQFFLGTARDLMTHGEKLVVYGAGWAFKFVPLFGRICADLAVEDSTAYDISRLAPQSAL

11000000000000000000000000000000000000000000000000000000000000000000000000000000000000000000000000000000000000000000000000000000000000000000000000000000000000000000000000000000000000000000000000000000000000000000000000000000000000000000000000000000000000000000000000000000000000000000000000000000000000000000000000000000000000000000000000000000000000000000000000000000000000000000000000011

>DM_train452

ASPRANDAPIVLLHGFTGWGREEMLGFKYWGGVRGDIEQWLNDNGYRTYTLAVGPLSSNWDRACEAYAQLVGGTVDYGAAHAAKHGHARFGRTYPGLLPELKRGGRVHIIAHSQGGQTARMLVSLLENGSQEEREYAKEHNVSLSPLFEGGHRFVLSVTTIATPHDGTTLVNMVDFTDRFFDLQKAVLEAAAVASNAPYTSEIYDFKLDQWGLRREPGESFDHYFERLKRSPVWTSTDTARYDLSVPGAETLNRWVKASPNTYYLSFSTERTYRGALTGNYYPELGMNAFSAIVCAPFLGSYRNAALGIDSHWLGNDGIVNTISMNGPKRGSNDRIVPYDGTLKKGVWNDMGTYNVDHLEVIGVDPNPSFNIRAFYLRLAEQLASLRP

1110000000000000000000000000000000000000000000000000000000000000000000000000000000000000000000000000000000000000000000000000000000000000000000000000000000000000000000000000000000000000000000000000000000000000000000000000000000000000000000000000000000000000000000000000000000000000000000000000000000000000000000000000000000000000000000000000000000000000000000000000000000000000000000000000

>DM_train453

MNLHEYQAKQLFARYGLPAPVGYACTTPREAEEAASKIGAGPWVVKCQVHAGGRGKAGGVKVVNSKEDIRAFAENWLGKRLVTYQTDANGQPVNQILVEAATDIAKELYLGAVVDRSSRRVVFMASTEGGVEIEKVAEETPHLIHKVALDPLTGPMPYQGRELAFKLGLEGKLVQQFTKIFMGLATIFLERDLALIEINPLVITKQGDLICLDGKLGADGNALFRQPDLREMRDQSQEDPREAQAAQWELNYVALDGNIGCMVNGAGLAMGTMDIVKLHGGEPANFLDVGGGATKERVTEAFKIILSDDKVKAVLVNIFGGIVRCDLIADGIIGAVAEVGVNVPVVVRLEGNNAELGAKKLADSGLNIIAAKGLTDAAQQVVAAVEGK

0000000000000000000000000000000000000000000000000000000000000000000000000000000000000000000000000000000000000000000000000000000000000000000000000000000000000000000000000000000000000000000000000000000000000000000000000000000000000000000000000000000000000000000000000000000000000000000000000000000000000000000000000000000000000000000000000000000000000000000000000000000000000000000000000111

>DM_train455

MEKTTEGAFYTREYRNLFKEFGYSEAEIQERVKDTWEQLFGDNPETKIYYEVGDDLGYLLDTGNLDVRTEGMSYGMMMAVQMDRKDIFDRIWNWTMKNMYMTEGVHAGYFAWSCQPDGTKNSWGPAPDGEEYFALALFFASHRWGDGDEQPFNYSEQARKLLHTCVHNGEGGPGHPMWNRDNKLIKFIPEVEFSDPSYHLPHFYELFSLWANEEDRVFWKEAAEASREYLKIACHPETGLAPEYAYYDGTPNDEKGYGHFFSDSYRVAANIGLDAEWFGGSEWSAEEINKIQAFFADKEPEDYRRYKIDGEPFEEKSLHPVGLIATNAMGSLASVDGPYAKANVDLFWNTPVRTGNRRYYDNCLYLFAMLALSGNFKIWFPEGQEEEH

1111100000000000000000000000000000000000000011000000000000000000000000000000000000000000000000000000000000000000000000000000000000000000000000000000000000000000000000000000000000000000000000000000000000000000000000000000000000000000000000000000000000000000000000000000000000000000000000000000000000000000000000000000000000000000000000000000000000000000000000000000000000000000000001111111

>DM_train456

MAGSATVEKRLDFGLLGPLQMTIDGTPVPSGTPKQRAVLAMLVINRNRPVGVDALITALWEEWPPSGARASIHSYVSNLRKLLGGAGIDPRVVLAAAPPGYRLSIPDNTCDLGRFVAEKTAGVHAAAAGRFEQASRHLSAALREWRGPVLDDLRDFQFVEPFATALVEDKVLAHTAKAEAEIACGRASAVIAELEALTFEHPYREPLWTQLITAYYLSDRQSDALGAYRRVKTTLADDLGIDPGPTLRALNERILRQQPLDAKKSAKTTAAGTVTVLDQRTMASGQQAVAYLHDIASGRGYPLQAAATRIGRLHDNDIVLDSANVSRHHAVIVDTGTNYVINDLRSSNGVHVQHERIRSAVTLNDGDHIRICDHEFTFQISAGTHGGT

1111111000000000000000000000000000000000000000000000000000000000000000000000000000000000000000000000000000000000000000000000000000000000000000000000000000000000000000000000000000000000000000000000000000000000000000000000000000000000000000000000000000000000000000000000000000000000000000000000000000000000000000000000000000000000000000000000000000000000000000000000000000000000000011111111

>DM_train457

MGKNLQALAQLYKNALLNDVLPFWENHSLDSEGGYFTCLDRQGKVYDTDKFIWLQNRQVWTFSMLCNQLEKRENWLKIARNGAKFLAQHGRDDEGNWYFALTRGGEPLVQPYNIFSDCFAAMAFSQYALASGEEWAKDVAMQAYNNVLRRKDNPKGKYTKTYPGTRPMKALAVPMILANLTLEMEWLLPQETLENVLAATVQEVMGDFLDQEQGLMYENVAPDGSHIDCFEGRLINPGHGIEAMWFIMDIARRKNDSKTINQAVDVVLNILNFAWDNEYGGLYYFMDAAGHPPQQLEWDQKLWWVHLESLVALAMGYRLTGRDACWAWYQKMHDYSWQHFADPEYGEWFGYLNRRGEVLLNLKGGKWKGCFHVPRAMYLCWQQFEALS

1100000000000000000000000000000000000000000000000000000000000000000000000000000000000000000000000000000000000000000000000000000000000000000000000000001111111111111100000000000000000000000000000000000000000000000000000000000000000000000000000000000000000000000000000000000000000000000000000000000000000000000000000000000000000000000000000000000000000000000000000000000000000000000000000000

>DM_train458

NSIQIGGLFPRGADQEYSAFRVGMVQFSTSEFRLTPHIDNLEVANSFAVTNAFCSQFSRGVYAIFGFYDKKSVNTITSFCGTLHVSFITPSFPTDGTHPFVIQMRPDLKGALLSLIEYYQWDKFAYLYDSDRGLSTLQAVLDSAAEKKWQVTAINVGNINNDKKDEMYRSLFQDLELKKERRVILDCERDKVNDIVDQVITIGKHVKGYHYIIANLGFTDGDLLKIQFGGANVSGFQIVDYDDSLVSKFIERWSTLEEKEYPGAHTTTIKYTSALTYDAVQVMTEAFRNLRKQRIEISRRGNAGDCLANPAVPWGQGVEIERALKQVQVEGLSGNIKFDQNGKRINYTINIMELKTNGPRKIGYWSEVDKMVVTLTELPSGNDTSGLE

0000000000000000000000000000000000000000000000000000000000000000000000000000000000000000000000000000000000000000000000000000000000000000000000000000000000000000000000000000000000000000000000000000000000000000000000000000000000000000000000000000000000000000000000000000000000000000000000000000000001111110000000000000000000000000000000000000000000000000000000000000000000000011111111111111

>DM_train459

QKTQSAPGTLSPDARNEKQPFYGEHQAGILTPQQAAMMLVAFDVLASDKADLERLFRLLTQRFAFLTQGGAAPETPNPRLPPLDSGILGGYIAPDNLTITLSVGHSLFDERFGLAPQMPKKLQKMTRFPNDSLDAALCHGDVLLQICANTQDTVIHALRDIIKHTPDLLSVRWKREGFISDHAARSKGKETPINLLGFKDGTANPDSQNDKLMQKVVWVTADQQEPAWTIGGSYQAVRLIQFRVEFWDRTPLKEQQTIFGRDKQTGAPLGMQHEHDVPDYASDPEGKVIALDSHIRLANPRTAESESSLMLRRGYSYSLGVTNSGQLDMGLLFVCYQHDLEKGFLTVQKRLNGEALEEYVKPIGGGYFFALPGVKDANDYFGSALLRV

1111111111110000000000000000000000000000000000000000000000000000000000000000000000000000000000000000000000000000000000000000000000000000000000000000000000000000000000000000000000000000000000000000000000000000000000000000000000000000000000000000000000000000000000000000000000000000000000000000000000011111111000000000000000000000000000000000000000000000000000000000000000000000000000000000

>DM_train460

MEQVVIVDAIRTPMGRSKGGAFRNVRAEDLSAHLMRSLLARNPSLTAATLDDIYWGCVQQTLEQGFNIARNAALLAEIPHSVPAVTVNRLCGSSMQALHDAARMIMTGDAQVCLVGGVEHMGHVPMSHGVDFHPGLSRNVAKAAGMMGLTAEMLSRLHGISREMQDQFAARSHARAWAATQSGAFKTEIIPTGGHDADGVLKQFNYDEVIRPETTVEALSTLRPAFDPVSGTVTAGTSSALSDGAAAMLVMSESRARELGLKPRARIRSMAVVGCDPSIMGYGPVPASKLALKKAGLSASDIDVFEMNEAFAAQILPCIKDLGLMEQIDEKINLNGGAIALGHPLGCSGARISTTLINLMERKDAQFGLATMCIGLGQGIATVFERV

000000000000000000000000000000000000000000000000000000000000000000000000000000000000000000000000000000000000000000000000000000000000000001111111000000000000000000000000000000000000000000000000000000000000000000000000000000000000000000000000000000000000000000000000000000000000000000000000000000000000000000000000000000000000000000000000000000000000000000000000000000000000000000000000000

>DM_train461

MSKTIIKNIGKIVSGDIKSPVLQADTIVVEDGLIAAIGGEELMKDAGDATIIDAAGSTVTPGLLDTHVHVSGGDYAPRQKTMDFISSALHGGVTTMISAGSPHFPGRPKDAAGTKALAITLSKSYYNARPAGVKVHGGAVILEKGLTEEDFIEMKKEGVWIVGEVGLGTIKNPEDAAPMVEWAHKHGFKVQMHTGGTSIPGSSTVTADDVIKTKPDVVSHINGGPTAISVQEVDRIMDETDFAMEIVQCGNPKIADYVARRAAEKGQLGRVIFGNDAPSGTGLIPLGILRNMCQIASMSDIDPEVAVCMATGNSTAVYGLNTGVIAPGKEADLIIMDTPLGSVAEDAMGAIAAGDIPGISVVLIDGEAVVTKSRNTPPAKRAAKIL

10000000000000000000000000000000000000000000000000000000000000000000000000000000000000000000000000000000000000000000000000000000000000000000000000000000000000000000000000000000000000000000000000000000000000000000000000000000000000000000000000000000000000000000000000000000000000000000000000000000000000000000000000000000000000000000000000000000000000000000000000000000000000000000000000

>DM_train462

MKKQNDIPQPIRGDKGATVKIPRNIERDRQNPDMLVPPETDHGTVSNMKFSFSDTHNRLEKGGYAREVTVRELPISENLASVNMRLKPGAIRELHWHKEAEWAYMIYGSARVTIVDEKGRSFIDDVGEGDLWYFPSGLPHSIQALEEGAEFLLVFDDGSFSENSTFQLTDWLAHTPKEVIAANFGVTKEEISNLPGKEKYIFENQLPGSLKDDIVEGPNGEVPYPFTYRLLEQEPIESEGGKVYIADSTNFKVSKTIASALVTVEPGAMRELHWHPNTHEWQYYISGKARMTVFASDGHARTFNYQAGDVGYVPFAMGHYVENIGDEPLVFLEIFKDDHYADVSLNQWLAMLPETFVQAHLDLGKDFTDVLSKEKHPVVKKKCSK

1111111000000000000000000000000000000000000000000000000000000000000000000000000000000000000000000000000000000000000000000000000000000000000000000000000000000000000000000000000000000000000000000000000000000000000000000000000000000000000000000000000000000000000000000000000000000000000000000000000000000000000000000000000000000000000000000000000000000000000000000000000000000000000111111

>DM_train466

MTPISQTPGLLAEAMVDLGAIEHNVRVLREHAGHAQLMAVVKADGYGHGATRVAQTALGAGAAELGVATVDEALALRADGITAPVLAWLHPPGIDFGPALLADVQVAVSSLRQLDELLHAVRRTGRTATVTVKVDTGLNRNGVGPAQFPAMLTALRQAMAEDAVRLRGLMSHMVYADKPDDSINDVQAQRFTAFLAQAREQGVRFEVAHLSNSSATMARPDLTFDLVRPGIAVYGLSPVPALGDMGLVPAMTVKCAVALVKSIRAGEGVSYGHTWIAPRDTNLALLPIGYADGVFRSLGGRLEVLINGRRCPGVGRICMDQFMVDLGPGPLDVAEGDEAILFGPGIRGEPTAQDWADLVGTIHYEVVTSPRGRITRTYREAENR

111111111100000000000000000000000000000000000000000000000000000000000000000000000000000000000000000000000000000000000000000000000000000000000000000000000000000000000000000000000000000000000000000000000000000000000000000000000000000000000000000000000000000000000001111111111111110000000000000000000000000000000000000000000000000000000000000000000000000000000000000000000000000000000111

>DM_train467

MKSKKILIVGAGFSGAVIGRQLAEKGHQVHIIDQRDHIGGNSYDARDSETNVMVHVYGPHIFHTDNETVWNYVNKHAEMMPYVNRVKATVNGQVFSLPINLHTINQFFSKTCSPDEARALIAEKGDSTIADPQTFEEEALRFIGKELYEAFFKGYTIKQWGMQPSELPASILKRLPVRFNYDDNYFNHKFQGMPKCGYTQMIKSILNHENIKVDLQREFIVEERTHYDHVFYSGPLDAFYGYQYGRLGYRTLDFKKFTYQGDYQGCAVMNYCSVDVPYTRITEHKYFSPWEQHDGSVCYKEYSRACEENDIPYYPIRQMGEMALLEKYLSLAENETNITFVGRLGTYRYLDMDVTIAEALKTAEVYLNSLTENQPMPVFTVSVR

100000000000000000000000000000000000000000000000000000000000000000000000000000000000000000000000000000000000000000000000000000000000000000000000000000000000000000000000000000000000000000000000000000000000000000000000000000000000000000000000000000000000000000000000000000000000000000000000000000000000000000000000000000000000000000000000000000000000000000000000000000000000000000000000

>DM_train468

MELQEVLHMNGGEGDTSYAKNSSYNLFLIRVKPVLEQCIQELLRANLPNINKCFKVGDLGCASGPNTFSTVRDIVQSIDKVGQEKKNELERPTIQIFLNDLFQNDFNSVFKLLPSFYRNLEKENGRKIGSCLIGAMPGSFYSRLFPEESMHFLHSCYCLHWLSQVPSGLVTELGISVNKGCIYSSKASRPPIQKAYLDQFTKDFTTFLRIHSEELISRGRMLLTFICKEDEFDHPNSMDLLEMSINDLVIEGHLEEEKLDSFNVPIYAPSTEEVKRIVEEEGSFEILYLETFNAPYDAGFSIDDDYQGRSHSPVSCDEHARAAHVASVVRSIYEPILASHFGEAILPDLSHRIAKNAAKVLRSGKGFYDSVIISLAKKPEKADM

110000000001111000000000000000000000000000000000000000000000000000000000000000000111111111100000000000000000000000000000000000000000000000000000000000000000000000000000111111000000000000000000000000000000000000000000000000000000000000000000000000000000000000000000000000000000000000000000000000000000001111111110000000000000000000000000000000000000000000000000000000000000000000011111

>DM_train469

MRILFVAAGSPATVFALAPLATAARNAGHQVVMAANQDMGPVVTGVGLPAVATTDLPIRHFITTDREGRPEAIPSDPVAQARFTGRWFARMAASSLPRMLDFSRAWRPDLIVGGTMSYVAPLLALHLGVPHARQTWDAVDADGIHPGADAELRPELSELGLERLPAPDLFIDICPPSLRPANAAPARMMRHVATSRQCPLEPWMYTRDTRQRVLVTSGSRVAKESYDRNFDFLRGLAKDLVRWDVELIVAAPDTVAEALRAEVPQARVGWTPLDVVAPTCDLLVHHAGGVSTLTGLSAGVPQLLIPKGSVLEAPARRVADYGAAIALLPGEDSTEAIADSCQELQAKDTYARRAQDLSREISGMPLPATVVTALEQLAHHHHHH

000000000000000000000000000000000000000000000000000000000000000000000000000000000000000000000000000000000000000000000000000000000000000000000000000000000000000000000000000000000000000000000000000000000000000000000000001111111100000000000000000000000000000000000000000000000000000000000000000000000000000000000000000000000000000000000000000000000000000000000000000000000000000000000000

>DM_train471

NDVGAESKQPLLDIALKGLKRTLPQLEQMDGNSLRENFQEMASGNGPLRSLMTNLQNLNKIPEAKQLNDYVTTLTNIQVGVARFSQWGTCGGEVERWVDKASTHELTQAVKKIHVIAKELKNVTAELEKIEAGAPMPQTMSGPTLGLARFAVSSIPINQQTQVKLSDGMPVPVNTLTFDGKPVALAGSYPKNTPDALEAHMKMLLEKECSCLVVLTSEDQMQAKQLPPYFRGSYTFGEVHTNSQKVSSASQGEAIDQYNMQLSCGEKRYTIPVLHVKNWPDHQPLPSTDQLEYLADRVKNSNQNGAPGRSSSDKHLPMIHCLGGVGRTGTMAAALVLKDNPHSNLEQVRADFRDSRNNRMLEDASQFVQLKAMQAQLLMTTAS

11111111110000000000000000000000000111111111110000000000000111110000000000000000000000011111000000000000000000000000000000000000001111110000000000000000000000000000000000000000000000000000000000000000000000000000000000000000000000000000000000000000000000000000000000000000000000000000000000000000000000000000000000000000000000000000000000000000000000000000000000000000000000000001111

>DM_train472

AAASFGQTKIPRGNGPYSVGCTDLMFDHTNKGTFLRLYYPSQDNDRLDTLWIPNKEYFWGLSKFLGTHWLMGNILRLLFGSMTTPANWNSPLRPGEKYPLVVFSHGLGAFRTLYSAIGIDLASHGFIVAAVEHRDRSASATYYFKDQSAAEIGDKSWLYLRTLKQEEETHIRNEQVRQRAKECSQALSLILDIDHGKPVKNALDLKFDMEQLKDSIDREKIAVIGHSFGGATVIQTLSEDQRFRCGIALDAWMFPLGDEVYSRIPQPLFFINSEYFQYPANIIKMKKCYSPDKERKMITIRGSVHQNFADFTFATGKIIGHMLKLKGDIDSNVAIDLSNKASLAFLQKHLGLHKDFDQWDCLIEGDDENLIPGTNINTTNQHI

11111110000000000000000000000000000000000000000000000000000000000000000000000000000000000000000000000000000000000000000000000000000000000000000000000000000000000000000000000000000000000000000000000000000000000000000000000000000000000000000000000000000000000000000000000000000000000000000000000000000000000000000000000000000000000000000000000000000000000000000000000000000000000001111

>DM_train473

PQPPADEQPEPRTRRRAYLWCKEFLPGAWRGLREDEFHISVIRGGLSNMLFQCSLPDTTATLGDEPRKVLLRLYGAILQMRSCNKEGSEQAQKENEFQGAEAMVLESVMFAILAERSLGPKLYGIFPQGRLEQFIPSRRLDTEELSLPDISAEIAEKMATFHGMKMPFNKEPKWLFGTMEKYLKEVLRIKFTEESRIKKLHKLLSYNLPLELENLRSLLESTPSPVVFCHNDCQEGNILLLEGRENSEKQKLMLIDFEYSSYNYRGFDIGNHFCEWMYDYSYEKYPFFRANIRKYPTKKQQLHFISSYLPAFQNDFENLSTEEKSIIKEEMLLEVNRFALASHFLWGLWSIVQAKISSIEFGYMDYAQARFDAYFHQKRKLGV

11111111111110000000000000100111111111111111111100000111111111111111000001111111111111111111111111111100000000000000000000000000000000000000000000000000000000000000000000000000000000000000000000000000000000000000000000000000000000000000000000000000000000000000000000000000000000000000000000000000000000000000000000000000000000000000000000000000000000000000000000000000000000000000000

>DM_train476

MKALHFGAGNIGRGFIGKLLADAGIQLTFADVNQVVLDALNARHSYQVHVVGETEQVDTVSGVNAVSSIGDDVVDLIAQVDLVTTAVGPVVLERIAPAIAKGLVKRKEQGNESPLNIIACENMVRGTTQLKGHVMNALPEDAKAWVEEHVGFVDSAVDRIVPPSASATNDPLEVTVETFSEWIVDKTQFKGALPNIPGMELTDNLMAFVERKLFTLNTGHAITAYLGKLAGHQTIRDAILDEKIRAVVKGAMEESGAVLIKRYGFDADKHAAYIQKILGRFENPYLKDDVERVGRQPLRKLSAGDRLIKPLLGTLEYSLPHKNLIQGIAGAMHFRSEDDPQAQELAALIADKGPQAALAQISGLDANSEVVSEAVTAYKAMQ

0000000000000000000000000000000000000000000000000000000000000000000000000000000000000000000000000000000000000000000000000000000000000000000000000000000000000000000111110000000000000000000000000000000000000000000000000000000000000000000000000000000000000000000000000000000000000000000000000000000000000000000000000000000000000000000000000000000000000000000000000000000000000000000000

>DM_train477

EESRESPAEHGYYMPAEWDSHAQTWIGWPERQDNWRHNALPAQRVFAGVAKAISKFEPVTVCASPAQWENARKQLPEDIRVVEMSMNDSWFRDSGPTFIVRKRPVKLSSLNRNIAGIDWNFNAWGGANDGCYNDWSHDLLVSRKILALERIPRFQHSMILEGGSIHVDGEGTCLVTEECLLNKNRNPHMSKEQIEEELKKYLGVQSFIWLPRGLYGDEDTNGHIDNMCCFARPGVVLLSWTDDETDPQYERSVEALSVLSNSIDARGRKIQVIKLYIPEPLYMTEEESSGITQDGEAIPRLAGTRLAASYVNFYIANGGIIAPQFGDPIRDKEAIRVLSDTFPHHSVVGIENAREIVLAGGNIHCITQQQPAEPTSVAENGH

0000000000000000000000000000000000000000000000000000000000000000000000000000000000000000000000000000000111111100000000000000000000000000000000000000000000000000000000000000000000000000000000000000000000000000000000000000000000000000000000000000000000000000000000000000000000000000000000000000000000000000000000000000000000000000000000000000000000000000000000000000000000000001111111

>DM_train478

MKSFQLVATAASGLEAIVGKEVARLGYDPKVENGKVYFEGDLSAIARANLWLRVADRVKIVVGVFKATTFDELFEKTKALPWEDYLPLDAQFPVAGKSVKSTLYSVPDCQAIVKKAIVNRVSEKYRRSGRLMETGALFKLEVSILKDEVTLTIDTSGAGLHKRGYRLAQGSAPIKETMAAALVLLTSWHPDRPFYDPVCGSGTIPIEAALIGQNIAPGFNREFVSETWDWMPKQVWADARQEAEDLANYDQPLNIIGGDIDARLIEIAKQNAVEAGLGDLITFRQLQVADFQTEDEYGVVVANPPYGERLEDEEAVRQLYREMGIVYKRMPTWSVYVLTSYELFEEVYGKKATKKRKLYNGYLRTDLYQYWGPRKPRPKKED

0000000000000000000000000000000000000000000000000000000000000000000000000000000000000000000000000000000000000000000000000000000000000000000000000000000000000000000000000000000000000000000000000000000000000000000000000000000000000000000000000000000000000000000000000000000000000000000000000000000000000000000000000000000000000000000000000000000000000000000000011110000000000111111111

>DM_train480

MQRLDALLPTLRERAQETEDLRRIPDDSMKALQETGFFRLLQPEQWGGYQADPVLFYSAVRKIASACGSTGWVSSIIGVHNWHLALFSQQAQEDVWGNDTDVRISSSYAPMGAGQVVDGGYTVNGAWAWSSGCDHASWAVLGGPVIKDGRPVDFVSFLIPREDYRIDDVWNVVGLRGTGSNTVVVEDVFVPTHRVLSFKAMSNLTAPGLERNTAPVYKMPWGTIHPTTISAPIVGMAYGAYDAHVEHQGKRVRAAFAGEKAKDDPFAKVRIAEASSDIDAAWRQLSGNVADEYALLVAGEEVPFELRLRARRDQVRATGRAISSIDKLFESSGATALANGTPLQRFWRDAHAGRVHAANDPERAYVMYGTGEFGLPITDTMV

0000000000000000000000000000000000000000000000000000000000000000000000000000000000000000000000000000000000000000000000000000000000000000000000000000000000000000000000000000000000000000000000000000000000000000000000000000000000000000000000000000000000001111111111000000000000000000000000000000000000000000000000000000000000000000000000000000000000000000000000000000000000000000000000

>DM_train482

MDINGGGATLPQALYQTSGVLTAGFAQYIGVGSGNGKAAFLNNDYTKFQAGVTNKNVHWAGSDSKLSATELSTYASAKQPTWGKLIQVPSVGTSVAIPFNKSGSAAVNLSVQELCGVFSGRINTWDGISGSGRTGPIVVVYRSESSGTTELFTRFLNAKCNAETGNFAVTTTFGTSFSGGLPAGAVAATGSQGVMTALAAGDGRITYMSPDFAAPTLAGLDDATKVARVGKNVATNTQGVSPAAANVSAAIGAVPVPAAADRSNPDAWVPVFGPDNTAGVQPYPTSGYPILGFTNLIFSQCYADATQTTQVRDFFTKHYGASNNNDAAITANAFVPLPTAWKATVRASFLTASNALSIGNTNVCNGIGRPLLEAAHHHHHH

000000000000000000000000000000000000000000000000000000000000000000000000000000000000000000000000000000000000000000000000000000000000000000000000000000000000000000000000000000000000000000000000000000000000000000000000000000000000000000000000000000000000000000000000000000000000000000000000000000000000000000000000000000000000000000000000000000000000000000000000000000000000111111111

>DM_train483

HHHHHHTDPALRAAQQTPLYEQHTLCGARMVDFHGWMMPLHYGSQIDEHHAVRTDAGMFDVSHMTIVDLRGSRTREFLRYLLANDVAKLTKSGKALYSGMLNASGGVIDDLIVYYFTEDFFRLVVNSATREKDLSWITQHAEPFGIEITVRDDLSMIAVQGPNAQAKAATLFNDAQRQAVEGMKPFFGVQAGDLFIATTGYTGEAGYEIALPNEKAADFWRALVEAGVKPCGLGARDTLRLEAGMNLYGQEMDETISPLAANMGWTIAWEPADRDFIGREALEVQREHGTEKLVGLVMTEKGVLRNELPVRFTDAQGNQHEGIITSGTFSPTLGYSIALARVPEGIGETAIVQIRNREMPVKVTKPVFVRNGKAVAGLCGR

111111111111111000000000000000000000000000000000000000000000000000000000000000000000000000000000000000000000000000000000000000000000000000000000000000000000000000000000000000000000000000000000000000000000000000000000000000000000000000000000000000000000000000000000000000000000000000000000000000000000000000000000000000000000000000000000000000000000000000000000000000000000000000011

>DM_train484

MSTAVQFRGGTTAQHATFTGAAREITVDTDKNTVVVHDGATAGGFPLARHDLVKTAFIKADKSAVAFTRTGNATASIKAGTIVEVNGKLVQFTADTAITMPALTAGTDYAIYVCDDGTVRADSNFSAPTGYTSTTARKVGGFHYAPGSNAAAQAGGNTTAQINEYSLWDIKFRPAALDPRGMTLVAGAFWADIYLLGVNHLTDGTSKYNVTIADGSASPKKSTKFGGDGSAAYSDGAWYNFAEVMTHHGKRLPNYNEFQALAFGTTEATSSGGTDVPTTGVNGTGATSAWNIFTSKWGVVQASGCLWTWGNEFGGVNGASEYTANTGGRGSVYAQPAAALFGGAWNGTSLSGSRAALWYSGPSFSFAFFGARGVCDHLILE

111100000000000000000000000000000000000000000000000000000000000000000000000000000000000000000000000000000000000000000000000000000000000000000000000000000000000000000000000000000000000000000000000000000000000000000000000000000000000000000000000000000000000000000000000000000000000000000000000000000000000000000000000000000000000000000000000000000000000000000000000000000000000000001

>DM_train488

MIQFSINRTLFIHALNTTKRAISTKNAIPILSSIKIEVTSTGVTLTGSNGQISIENTIPVSNENAGLLITSPGAILLEASFFINIISSLPDISINVKEIEQHQVVLTSGKSEITLKGKDVDQYPRLQEVSTENPLILKTKLLKSIIAETAFAASLQESRPILTGVHIVLSNHKDFKAVATDSHRMSQRLITLDNTSADFMVVLPSKSLREFSAVFTDDIETVEVFFSPSQILFRSEHISFYTRLLEGNYPDTDRLLMTEFETEVVFNTQSLRHAMERAFLISNATQNGTVKLEITQNHISAHVNSPEVGKVNEDLDIVSQSGSDLTISFNPTYLIESLKAIKSETVKIHFLSPVRPFTLTPGDEEESFIQLITPVRTN

100000000000000000000000000000000000000000000000000000000000111110000000000000000000000000000000000000000000000000000000000000000000000000000000000000000000000000000000000000000000000000000000000000000000000000000000000000000000000000000000000000000000000000000000000000000000000000000000000000000000000000000000000000000000000000000000000000000000000000000000000000000000000001

>DM_train489

MELIEVQEGKAKILIPKAESIYDSPVFYNPRMALNRDIVVVLLNILNPKIVLDALSATGIRGIRFALETPAEEVWLNDISEDAYELMKRNVMLNFDGELRESKGRAILKGEKTIVINHDDANRLMAERHRYFHFIDLDPFGSPMEFLDTALRSAKRRGILGVTATDGAPLCGAHPRACLRKYLAVPLRGELCHEVGTRILVGVIARYAAKYDLGIDVILAYYKDHYFRAFVKLKDGARKGDETLEKLGYIYFDDKTGKFELEQGFLPTRPNAYGPVWLGPLKDEKIVSKMVKEAESLSLARKKQALKLLKMIDQELDIPLFYDTHAIGRRLKIETKKVEEIISALREQGYEATRTHFSPTGIKTSAPYEVFIETIKRI

110000000000000011111111000000000000000000000000000000000000000000000000000000000000000000000000000000000000000000000000000000000000000000000000000000000000000000000000000000000000000000000000000000000000000000000000000000000000000000000000000000000000000000000000000000000000000000000000000000000000000000000000000000000000000000000000000000000000000000000000000000000000000001

>DM_train490

MTKKRVALIFGGNSSEHDVSKRSAQNFYNAIEATGKYEIIVFAIAQNGFFLDTESSKKILALEDEQPIVDAFMKTVDASDPLARIHALKSAGDFDIFFPVVHGNLGEDGTLQGLFKLLDKPYVGAPLRGHAVSFDKALTKELLTVNGIRNTKYIVVDPESANNWSWDKIVAELGNIVFVKAANQGSSVGISRVTNAEEYTEALSDSFQYDYKVLIEEAVNGARELEVGVIGNDQPLVSEIGAHTVPNQGSGDGWYDYNNKFVDNSAVHFQIPAQLSPEVTKEVKQMALDAYKVLNLRGEARMDFLLDENNVPYLGEPNTLPGFTNMSLFKRLWDYSDINNAKLVDMLIDYGFEDFAQNKKLSYSFVSLGEEKIGKFN

00000000000000000000000000000000000000000000000000000000000000000000000000000000000000000000000000000000000000000000000000000000000000000000000000000000000000000000000000000000000000000000000000000000000000000000000000000000000000000000000000001111111111111111111111000000000000000000000000000000000000000000000000000000000000000000000000000000000000000000000000000000001111111

>DM_train491

GLKEHDFNPEEAEETKQVSWKLVTEYAMETKCDDVLLLLGMYLEFQYSFEMCLKCIKKEQPSHYKYHEKHYANAAIFADSKNQKTICQQAVDTVLAKKRVDSLQLTREQMLTNRFNDLLDRMDIMFGSTGSADIEEWMAGVAWLHCLLPKMDSVVYDFLKCMVYNIPKKRYWLFKGPIDSGKTTLAAALLELCGGKALNVNLPLDRLNFELGVAIDQFLVVFEDVKGTGGESRDLPSGQGINNLDNLRDYLDGSVKVNLEKKHLNKRTQIFPPGIVTMNEYSVPKTLQARFVKQIDFRPKDYLKHCLERSEFLLEKRIIQSGIALLLMLIWYRPVAEFAQSIQSRIVEWKERLDKEFSLSVYQKMKFNVAMGIGVLD

11111111111111100000000000000000000000000000000000000000000000000000000000000000000000000000000000000000000000000000000000000000000000000000000000000000000000000000000000000000000000000000000000000000000000000000000000000000000000000000000000000000000000000000000000000000000000000000000000000000000000000000000000000000000000000000000000000000000000000000000000000000000000000

>DM_train492

GMKSAPDWIAHPDTPYLLIDEAKLKSNINYLKQRVESLGSHLRPHLKTLRTLEAAGYLLDSKSAPATVSTLAEAEAYAKAGYTDLLYAVGIAPAKLKRVAALRQQGINLHILLDNITQAQAVVDYAAEFGQDFSVFIEIDSDDHRGGIKPSDSKLLTIAKTLGEHFTGLMTHAGGSYACNTEQGLKNFAKQECDAVRIARNNLETAGIHCAITSVGSTPTAHFGEDFSDISEVRAGVYTTFDLVMKNIGVCDFSHIAMSVVTTVIGHNKEKNWLLTDSGWMALSRDSGTAGQNRDFGYGQVCKIDGSVLDGLCVNSTSQEHGVIELSDAYQLEDFPVGHQLRIMPNHACATAAMHPVYHVLMSDGSHNTWQRITGW

1110000000000000000000000000000000000000000000000000000000000000000000000000000000000000000000000000000000000000000000000000000000000000000000000000000000000000000000000000000000000000000000000000000000000000000000000000000000000000000000000000000000000000000000000000000000000000000000000000000000000000000000000000000000000000000000000000000000000000000000000000000000000000

>DM_train493

MREWKIIDSTLREGEQFEKANFSTQDKVEIAKALDEFGIEYIEVTTPVASPQSRKDAEVLASLGLKAKVVTHIQCRLDAAKVAVETGVQGIDLLFGTSKYLRAPHGRDIPRIIEEAKEVIAYIREAAPHVEVRFSAEDTFRSEEQDLLAVYEAVAPYVDRVGLADTVGVATPRQVYALVREVRRVVGPRVDIEFHGHNDTGCAIANAYEAIEAGATHVDTTILGIGERNGITPLGGFLARMYTLQPEYVRRKYKLEMLPELDRMVARMVGVEIPFNNYITGETAFSHKAGMHLKAIYINPEAYEPYPPEVFGVKRKLIIASRLTGRHAIKARAEELGLHYGEEELHRVTQHIKALADRGQLTLEELDRILREWITA

1000000000000000000000000000000000000000000000000000000000000000000000000000000000000000000000000000011111100000000000000000000000000000000000000000000000000000000000000000000000000000000000000000000000000000000000000000000000000000000000000000000000000000000000000000000000000000000000000000000000000000000000000011111111111111111111110000000000000000000000000000000000000000

>DM_train494

MTSQTPTRKPRARDLGLPFTGVTGPYNAITDVDGVGVGFQTIIENEPRPGRKRPARSGVTAILPHMQSETPVPVYAGVHRFNGNGEMTGTHWIEDGGYFLGPVVITNTHGIGMAHHATVRWMVDRYASTYQTDDFLWIMPVVAETYDGALNDINGFPVTEADVRKALDNVASGPVQEGNCGGGTGMITYGFKGGTGTASRVVEFGGRSFTIGALVQANHGQRDWLTIAGVPVGQHMRDGTPQSQLQERGSIIVVLATDLPLMPHQLKRLARRASIGIGRNGTPGGNNSGDIFIAFSTANQRPMQHRSAPFLDVEMVNDEPLDTVYLAAVDSVEEAVVNAMIAAEDMGGTPFDRLLVQAIDHERLRAVLRQYGRLA

111111110000000000000000000000000000000000000000000000000000000000000000000000000000000000000000000000000000000000000000000000000000000000000000000000000000000000000000000000000000000000000000000000000000000000000000000000000000000000000000000001111000000000000000000000000000000000000000000000000000000000000000000000000000000000000000000000000000000000000000000000000000000

>DM_train495

MSLTREAYHRLTPLPHPGGRLFIKPGARGYRDPVHDLLQKTVEPFGERALDLNPGVGWGSLPLEGRMAVERLETSRAAFRCLTASGLQARLALPWEAAAGAYDLVVLALPAGRGTAYVQASLVAAARALRMGGRLYLAGDKNKGFERYFKEARALLGYGVVVRREGPYRVALLEKEKEAPPLPSLWRAFSARILGAEYTFHHLPGVFSAGKVDPASLLLLEALQERLGPEGVRGRQVLDLGAGYGALTLPLARMGAEVVGVEDDLASVLSLQKGLEANALKAQALHSDVDEALTEEARFDIIVTNPPFHVGGAVILDVAQAFVNVAAARLRPGGVFFLVSNPFLKYEPLLEEKFGAFQTLKVAEYKVLFAEKRGR

110000000000000000000000000000000000000000000000000000000000000000000000000000000000000000000000000000000000000000000000000000000000000000000000000000000000000000000000000000000000000000000000000000000000000000000000000000000000000000000000000000000000000000000000000000000000000000000000000000000000000000000000000000000000000000000000000000000000000000000000000000000000011

>DM_train497

MENFPTEYFLNTTVRLLEYIRYRDSNYTREERIENLHYAYNKAAHHFAQPRQQQLLKVDPKRLQASLQTIVGMVVYSWAKVSKECMADLSIHYTYTLVLDDSKDDPYPTMVNYFDDLQAGREQAHPWWALVNEHFPNVLRHFGPFCSLNLIRSTLDFFEGCWIEQYNFGGFPGSHDYPQFLRRMNGLGHCVGASLWPKEQFNERSLFLEITSAIAQMENWMVWVNDLMSFYKEFDDERDQISLVKNYVVSDEISLHEALEKLTQDTLHSSKQMVAVFSDKDPQVMDTIECFMHGYVTWHLCDRRYRLSEIYEKVKEEKTEDAQKFCKFYEQAANVGAVSPSEWAYPPVAQLANVRSKDVKEVQKPFLSSIELVE

11110000000000000000000000000000000000000000000000000000000000000000000000000000000000000000000000000000000000000000000000000000000000000000000000000000000000000000000000000000000000000000000000000000000000000000000000000000000000000000000000000000000000000000000000000000000000000000000000000000000000000000000000000000000000000000000000000000000000000011111111111111111111

>DM_train498

MTWKNFGFEIFGEKYGQEELEKRIKDEHTPPPDSPVFGGLKLKLKKEKFKTLFTLGTTLKGFRRATHTVGTGGIGEITIVNDPKFPEHEFFTAGRTFPARLRHANLKYPDDAGADARSFSIKFADSDSDGPLDIVMNTGEANIFWNSPSLEDFVPVEEGDAAEEYVYKNPYYYYNLVEALRRAPDTFAHLYYYSQVTMPFKAKDGKVRYCRYRALPGDVDIKEEDESGRLTEEEQRKIWIFSRHENEKRPDDYLRKEYVERLQKGPVNYRLQIQIHEASPDDTATIFHAGILWDKETHPWFDLAKVSIKTPLSPDVLEKTAFNIANQPASLGLLEAKSPEDYNSIGELRVAVYTWVQHLRKLKIGSLVPAGQNA

11000000000000000000000000000000000000000000000000000000000000000000000000000000000000000000000000000000000000000000000000000000000000000000000000000000000000000000000000000000000000000000000000000000000000000000000000000000000000000000000000000000000000000000000000000000000000000000000000000000000000000000000000000000000000000000000000000000000000000000000000000000111111

>DM_train499

MGSDKIHHHHHHMTSSPTSTQESSTSWYLLLQQLIDGESLSRSQAAELMQGWLSEAVPPELSGAILTALNFKGVSADELTGMAEVLQSQSKMGTGENYSQLPITNSPFSIIDTCGTGGDGSSTFNISTAVAFVAAAYGVPVAKHGNRSASSLTGSADVLEALGVNLGASPEKVQAALQEVGITFLFAPGWHPALKAVATLRRTLRIRTVFNLLGPLVNPLRPTGQVVGLFTPKLLTTVAQALDNLGKQKAIVLHGRERLDEAGLGDLTDLAVLSDGELQLTTINPQEVGVTPAPIGALRGGDVQENAEILKAVLQGKGTQAQQDAVALNAALALQVAGAVPLLDHAQGVSVAKEILQTGTAWAKLAQLVYFLGN

11111111111111111111111110000000000000000000000000000000000000000000000000000000000000000000111111111111000000000000011100000000000000000000000000000010000000000000000000000000000000000000111000000000000000000000000000000000000000000000000000000000000000000000000000000000000000000000000000000000000000000000000000000000000000000000000000000000000000000000000000000000000000

>DM_train500

MIVAFCLYKYFPFGGLQRDFMRIASTVAARGHHVRVYTQSWEGDCPKAFELIQVPVKSHTNHGRNAEYYAWVQNHLKEHPADRVVGFNKMPGLDVYFAADVCYAEKVAQEKGFLYRLTSRYRHYAAFERATFEQGKSTKLMMLTDKQIADFQKHYQTEPERFQILPPGIYPDRKYSEQIPNSREIYRQKNGIKEQQNLLLQVGSDFGRKGVDRSIEALASLPESLRHNTLLFVVGQDKPRKFEALAEKLGVRSNVHFFSGRNDVSELMAAADLLLHPAYQEAAGIVLLEAITAGLPVLTTAVCGYAHYIADANCGTVIAEPFSQEQLNEVLRKALTQSPLRMAWAENARHYADTQDLYSLPEKAADIITGGLDG

10000000000000000000000000000000000000000000000000000000000000000000000000000000000000000000000000000000000000000000000000000000000000000000000000000000000000000000000000000000000000000000000000000000000000000000000000000000000000000000000000000000000000000000000000000000000000000000000000000000000000000000000000000000000000000000000000000000000000000000000000000000000111

>DM_train501

GHEIKDIREDTMHAEFNALRAQVAINDGNPDEAERLAKLALEELPPGWFYSRIVATSVLGEVLHCKGELTRSLALMQQTEQMARQHDVWHYALWSLIQQSEILFAQGFLQTAWETQEKAFQLINEQHLEQLPMHEFLVRIRAQLLWAWARLDEAEASARSGIEVLSSYQPQQQLQCLAMLIQCSLARGDLDNARSQLNRLENLLGNGKYHSDWISNANKVRVIYWQMTGDKAAAANWLRHTAKPEFANNHFLQGQWRNIARAQILLGEFEPAEIVLEELNENARSLRLMSDLNRNLLLLNQLYWQAGRKSDAQRVLLDALKLANRTGFISHFVIEGEAMAQQLRQLIQLNTLPELEQHRAQRILREINQHHGA

0000000000000000000000000000000000000000000000000000000000000000000000000000000000000000000000000000000000000000000000000000000000000000000000000000000000000000000000000000000000000000000000000000000000000000000000000000000000000000000000000000000000000000000000000000000000000000000000000000000000000000000000000000000000000000000000000000000000000000000000000000000011111

>DM_train502

MGSMDQSIAVKSPLTYAEALANTIMNTYTVEELPPANRWHYHQGVFLCGVLRLWEATGEKRYFEYAKAYADLLIDDNGNLLFRRDELDAIQAGLILFPLYEQTKDERYVKAAKRLRSLYGTLNRTSEGGFWHKDGYPYQMWLDGLYMGGPFALKYANLKQETELFDQVVLQESLMRKHTKDAKTGLFYHAWDEAKKMPWANEETGCSPEFWARSIGWYVMSLADMIEELPKKHPNRHVWKNTLQDMIKSICRYQDKETGLWYQIVDKGDRSDNWLESSGSCLYMYAIAKGINKGYLDRAYETTLLKAYQGLIQHKTETSEDGAFLVKDICVGTSAGFYDYYVSRERSTNDLHGAGAFILAMTELEPLFRSAGK

1111111111000000000000000000000000000000000000000000000000000000000000000000000000000000000000000000000000000000000000000000000000000000000000000000000000000000000000000000000000000000000000000000000000000000000000000000000000000000000000000000000000000000000000000000000000000000000000000000000000000000000000000000000000000000000000000000000000000000000000000000000000000

>DM_train503

MAAVTLHLRAETKPLEARAALTPTTVKKLIAKGFKIYVEDSPQSTFNINEYRQAGAIIVPAGSWKTAPRDRIIIGLKEMPETDTFPLVHEHIQFAHCYKDQAGWQNVLMRFIKGHGTLYDLEFLENDQGRRVAAFGFYAGFAGAALGVRDWAFKQTHSDDEDLPAVSPYPNEKALVKDVTKDYKEALATGARKPTVLIIGALGRCGSGAIDLLHKVGIPDANILKWDIKETSRGGPFDEIPQADIFINCIYLSKPIAPFTNMEKLNNPNRRLRTVVDVSADTTNPHNPIPIYTVATVFNKPTVLVPTTAGPKLSVISIDHLPSLLPREASEFFSHDLLPSLELLPQRKTAPVWVRAKKLFDRHCARVKRSSRL

1000000000000000000000000000000000000000000000000000000000000000000000000000000000000000000000000000000000000000000000000000000000000000000000000000000000000000000000000000000000000000000000000000000000000000000000000000000000000000000000000000000000000000000000000000000000000000000000000000000000000000000000000000000000000000000000000000000000000000000000000000000000000

>DM_train504

MGNSYITKEDNQISATSEQTEDSACLSAMVLTTNLVYPAVLNAAIDLNLFEIIAKATPPGAFMSPSEIASKLPASTQHSDLPNRLDRMLRLLASYSVLTSTTRTIEDGGAERVYGLSMVGKYLVPDESRGYLASFTTFLCYPALLQVWMNFKEAVVDEDIDLFKNVHGVTKYEFMGKDKKMNQIFNKSMVDVCATEMKRMLEIYTGFEGISTLVDVGGGSGRNLELIISKYPLIKGINFDLPQVIENAPPLSGIEHVGGDMFASVPQGDAMILKAVCHNWSDEKCIEFLSNCHKALSPNGKVIIVEFILPEEPNTSEESKLVSTLDNLMFITVGGRERTEKQYEKLSKLSGFSKFQVACRAFNSLGVMEFYK

111111111111111111000000000000000000000000000000000000000000000000000000000000000000000000000000000000000000000000000000000000000000000000000000000000000000000111111111111110000000000000000000000000000000000000000000000000000000000000000000000000000000000000000000000000000000000000000000000000000000000000000000000000000000000000000000000000000000000000000000000000000000

>DM_train505

DPFAGDPPRHPGLRVNSQKPFNAEPPAELLAERFLTPNELFFTRNHLPVPAVEPSSYRLRVDGPGGRTLSLSLAELRSRFPKHEVTATLQCAGNRRSEMSRVRPVKGLPWDIGAISTARWGGARLRDVLLHAGFPEELQGEWHVCFEGLDADPGGAPYGASIPYGRALSPAADVLLAYEMNGTELPRDHGFPVRVVVPGVVGARSVKWLRRVAVSPDESPSHWQQNDYKGFSPCVDWDTVDYRTAPAIQELPVQSAVTQPRPGAAVPPGELTVKGYAWSGGGREVVRVDVSLDGGRTWKVARLMGDKAPPGRAWAWALWELTVPVEAGTELEIVCKAVDSSYNVQPDSVAPIWNLRGVLSTAWHRVRVSVQD

111111111111100000000000000000000000000000000000000000000000000000000000000000000000000000000000000000000000000000000000000000000000000000000000000000000000000000000000000000000000000000000000000000000000000000000000000000000000000000000000000000000000000000000000000000000000000000000000000000000000000000000000000000000000000000000000000000000000000000000000000000000000

>DM_train507

SPEVTMNISQMITYWGYPNEEYEVVTEDGYILEVNRIPYGKKNSGNTGQRPVVFLQHGLLASATNWISNLPNNSLAFILADAGYDVWLGNSRGNTWARRNLYYSPDSVEFWAFSFDEMAKYDLPATIDFIVKKTGQKQLHYVGHSQGTTIGFIAFSTNPSLAKRIKTFYALAPVATVKYTKSLINKLRFVPQSLFKFIFGDKIFYPHNFFDQFLATEVCSREMLNLLCSNALFIICGFDSKNFNTSRLDVYLSHNPAGTSVQNMFHWTQAVKSGKFQAYDWGSPVQNRMHYDQSQPPYYNVTAMNVPIAVWNGGKDLLADPQDVGLLLPKLPNLIYHKEIPFYNHLDFIWAMDAPQEVYNDIVSMISEDKK

00000000000000000000000000000000000000000000011100000000000000000000000000000000000000000000000000000000000000000000000000000000000000000000000000000000000000000000000000000000000000000000000000000000000000000000000000000000000000000000000000000000000000000000000000000000000000000000000000000000000000000000000000000000000000000000000000000000000000000000000000000000000

>DM_train509

GSSSSGLVPRGSHMSVQNVVLFDTQPLTLMLGGKLSYINVAYQTYGTLNDEKNNAVLICHALTGDAEPYFDDGRDGWWQNFMGAGLALDTDRYFFISSNVLGGCKGTTGPSSINPQTGKPYGSQFPNIVVQDIVKVQKALLEHLGISHLKAIIGGSFGGMQANQWAIDYPDFMDNIVNLCSSIYFSAEAIGFNHVMRQAVINDPNFNGGDYYEGTPPDQGLSIARMLGMLTYRTDLQLAKAFGRATKSDGSFWGDYFQVESYLSYQGKKFLERFDANSYLHLLRALDMYDPSLGYENVKEALSRIKARYTLVSVTTDQLFKPIDLYKSKQLLEQSGVDLHFYEFPSDYGHDAFLVDYDQFEKRIRDGLAGN

11111111111111000000000000000000000000000000000000000000000000000000000000000000000000000000000000000000000000000000000000000000000000000000000000000000000000000000000000000000000000000000000000000000000000000000000000000000000000000000000000000000000000000000000000000000000000000000000000000000000000000000000000000000000000000000000000000000000000000000000000000000000

>DM_train510

MAHHHHHHMNILQEPIDFLKKEELKNIDLSQMSKKERYKIWKRIPKCELHCHLDLCFSADFFVSCIRKYNLQPNLSDEEVLDYYLFAKGGKSLGEFVEKAIKVADIFHDYEVIEDLAKHAVFNKYKEGVVLMEFRYSPTFVAFKYNLDIELIHQAIVKGIKEVVELLDHKIHVALMCIGDTGHEAANIKASADFCLKHKADFVGFDHGGHEVDLKEYKEIFDYVRESGVPLSVHAGEDVTLPNLNTLYSAIQVLKVERIGHGIRVAESQELIDMVKEKNILLEVCPISNVLLKNAKSMDTHPIRQLYDAGVKVSVNSDDPGMFLTNINDDYEELYTHLNFTLEDFMKMNEWALEKSFMDSNIKDKIKNLYF

11111111111100000000000000000000000000000000000000000000000000000000000000000000000000000000000000000000000000000000000000000000000000000000000000000000000000000000000000000000000000000000000000000000000000000000000000000000000000000000000000000000000000000000000000000000000000000000000000000000000000000000000000000000000000000000000000000000000000000000000000000000000

>DM_train511

WDGKIDGTGTHAMIVTQGVSILENDLSKNEPESVRKNLEILKENMHELQLGSTYPDYDKNAYDLYQDHFWDPDIDNNFSKDNSWYLAYSIPDTGESQIRKFSALARYEWQRGNYKQATFYLGEAMHYFGDIDTPYHPANVTAVDSAGHVKFETFAEERKEQYKINTAGCKTNEAFYTDILKNKDFNAWSKEYARGFAKTGKSIYYSHASMSHSWDDWDYAAKVTLANSQKGTAGYIYRFLHDVSEGNDPSVGKNVKELVAYISTSGEKDAGTDDYMYFGIKTKDGKTQEWEMDNPGNDFMTGSKDTYTFKLKDENLKIDDIQNMWIRKRKYTAFPDAYKPENIKIIANGKVVVDKDINEWISGNSTYNIK

0000000000000000000000000000000000000000000000000000000000000000000000000011111111100000000000000000000000000000000000000000000000000000000000000000000000000000000000000000000000000000000000000000000000000000000000000000000000000000000000000000000000000000000000000000000000000000000000000000000000000000000000000000000000000000000000000000000000000000000000000000000000

>DM_train512

MSTDIDCIVIGAGVVGLAIARALAAGGHEVLVAEAAEGIGTGTSSRNSEVIHAGIYYPADSLKARLCVRGKHLLYEYCAARGVPHQRLGKLIVATSDAEASQLDSIARRAGANGVDDLQHIDGAAARRLEPALHCTAALVSPSTGIVDSHALMLAYQGDAESDGAQLVFHTPLIAGRVRPEGGFELDFGGAEPMTLSCRVLINAAGLHAPGLARRIEGIPRDSIPPEYLCKGSYFTLAGRAPFSRLIYPVPQHAGLGVHLTLDLGGQAKFGPDTEWIATEDYTLDPRRADVFYAAVRSYWPALPDGALAPGYTGIRPKISGPHEPAADFAIAGPASHGVAGLVNLYGIESPGLTASLAIAEETLARLAA

110000000000000000000000000000000000000000000000000000000000000000000000000000000000000000000000000000000000000000000000000000000000000000000000000000000000000000000000000000000000000000000000000000000000000000000000000000000000000000000000000000000000000000000000000000000000000000000000000000000000000000000000000000000000000000000000000000000000000000000000000000001

>DM_train513

KERRRAVLELLQRPGNARCADCGAPDPDWASYTLGVFICLSCSGIHRNIPQVSKVKSVRLDAWEEAQVEFMASHGNDAARARFESKVPSFYYRPTPSDCQLLREQWIRAKYERQEFIYPEKQEPYSAGYREGFLWKRGRDNGQFLSRKFVLTEREGALKYFNRNDAKEPKAVMKIEHLNATFQPAKIGHPHGLQVTYLKDNSTRNIFIYHEDGKEIVDWFNALRAARFHYLQVAFPGASDADLVPKLSRNYLKEGYMEKTGPKQTEGFRKRWFTMDDRRLMYFKDPLDAFARGEVFIGSKESGYTVLHGFPPSTQGHHWPHGITIVTPDRKFLFACETESDQREWVAAFQKAVDRPMLPQEYAVEAHF

00000000000000000000000000000000000000000000000000000000000000000000000000000000000000000000000000000000000000000000000000000000000000000000000000000000000000000000000000000000000000000000000000000000000000000000000000000000000000000000000000000000000000000000111111000000000000000000000000000000000000000000000000000000000000000000000000000000000000000000011111111111

>DM_train514

GMRVDKHEVRVGELAAGQPLSLPVYRFKGKGAGPSVYIQANVHGAEVQGNAVIYQLMKLLEHYELLGDISLVPLANPLGINQKSGEFTLGRFDPITGVNWNREYLDHGFNIEVWYQEHSHLDDDTLITAFRATLVEECARRLNNPWGVTTGHRLAVTLQSMAHRADIVLDLHTGPKSCKHLYCPEYERSAAQYFSIPYTLLIPNSFGGAMDEAAFVPWWTLAEVASSHGRELGVRVSALTLELGSQERIDLDDALEDAEGILAYLSHRGVIAETVLPKPMKRYGCFLKNYRKFHAPKAGMVEYLGKVGVPMKATDPLVNLLRLDLYGTGEELTVLRLPEDGVPILHFASASVHQGTELYKVMTKVFEL

11100000000000000000000000000000000000000000000000000000000000000000000000000000000000000000000000000000000000000000000000000000000000000000000000000000000000000000000000000000000000000000000000000000000000000000000000000000000000000000000000000000000000000000000000000000000000000000000000000000000000000000000000000000000000000000000000000000000000000000000000000000

>DM_train517

MPNPPAKEDTWAFGPIGSPFPDNPVKALGQQNMYVALWYKNGRPMHGRAWNNGGVIECSFPYNKSELTGVKDLGGQIQVLQYKGNHLSLGYWYNWIKYSDRFDKMDKGAEMLRCGDSFPILWSERPGGALLGYADNKTEIARFSHDGKVDEVSGSALANMLIIARELKGGPPYCECEECKSEPPKPIVRVTLNEWADFRCGDPWPTVGTPVRALGRSLDTLPGENPDQYVALWYQSGEPVMGRIWNDGGKIAACFGWGGHEYRQKIGSIQILYELPEAIRGFDYDWKPFPEAAQFGAKEWIPVHVDHHKGNISPAVLIVDGKEILGKADIRNERATIGYGGTEKVLVGPAVHSCMVLCRKAKPGCTID

11111000000000000000000000000000000000000000000000000000000000000000000000000000000000000000000000000000000000000000000000000000000000000000000000000000000000000000000000000000000000000110000000000000000000000000000000000000000000000000000000000000000000000000000000000000000000000000000000000011110000000000000000000000000000000000000000000000000000000000000000000000

>DM_train518

ASHSRKFLDVRSEEELLSCIKKETEAGKLPPNVAAGMEELYQNYRNAVIESGNPKADEIVLSNMTVALDRILLDVEDPFVFSSHHKAIREPFDYYIFGQNYIRPLIDFGNSFVGNLSLFKDIEEKLQQGHNVVLISNHQTEADPAIISLLLEKTNPYIAENTIFVAGDRVLADPLCKPFSIGRNLICVYSKKHMFDIPELTETKRKANTRSLKEMALLLRGGSQLIWIAPSGGRDRPDPSTGEWYPAPFDASSVDNMRRLIQHSDVPGHLFPLALLCHDIMPPPSQVEIEIGEKRVIAFNGAGLSVAPEISFEEIAATHKNPEEVREAYSKALFDSVAMQYNVLKTAISGKQGLGASTADVSLSQPW

0000000000000000000000000000000000000000000000000000000000000000000000000000000000000000000000000000000000000000000000000000000000000000000000000000000000000000000000000000000000000000000000000000000000000000000000000000000000000000000000000000000000000000000000000000000000000000000011111111110000000000000000000000000000000000000000000000000000000000000000000000000

>DM_train520

MKMKLAVLPGDGIGPEVMDAAIRVLKTVLDNDGHEAVFENALIGGAAIDEAGTPLPEETLDICRRSDAILLGAVGGPKWDHNPASLRPEKGLLGLRKEMGLFANLRPVKAYATLLNASPLKRERVENVDLVIVRELTGGLYFGRPSERRGPGENEVVDTLAYTREEIERIIEKAFQLAQIRRKKLASVDKANVLESSRMWREIAEETAKKYPDVELSHMLVDSTSMQLIANPGQFDVIVTENMFGDILSDEASVITGSLGMLPSASLRSDRFGMYEPVHGSAPDIAGQGKANPLGTVLSAALMLRYSFGLEKEAAAIEKAVDDVLQDGYCTGDLQVANGKVVSTIELTDRLIEKLNNSAARPRIFQ

000000000000000000000000000000000000000000000000000000000000000000000000000000000000000000000000000000000000000000000000000000000000000000000000000000000000000000000000000000000000000000000000000000000000000000000000000000000000000000000000000000000000000000000000000000000000000000000000000000000000000000000000000000000000000000000000000000000000000000001111111111

>DM_train522

MGSSHHHHHHSSGLVSRRHMNIDMAALHAIEVDRGISVNELLETIKSALLTAYRHTQGHQTDARIEIDRKTGVVRVIARETDEAGNLISEWDDTPEGFGRIAATTARQVMLQRFRDAENERTYGEFSTREGEIVAGVIQRDSRANARGLVVVRIGTETKASEGVIPAAEQVPGESYEHGNRLRCYVVGVTRGAREPLITLSRTHPNLVRKLFSLEVPEIADGSVEIVAVAREAGHRSKIAVRSNVAGLNAKGACIGPMGQRVRNVMSELSGEKIDIIDYDDDPARFVANALSPAKVVSVSVIDQTARAARVVVPDFQLSLAIGKEGQNARLAARLTGWRIDIRGDAPPPPPGQPEPGVSRGMAHDR

111111111111110000000000000000000000000000000000000000000000000000000000000000000000000000000000000000000000000000000011111111000000000000000000000000000000000000000000000000000000000000000000000000000000000000000000000000000000000000000000000000000000000000000000000000000000000000000000000000000000000000000000000000000000000000000000000000000000111111111111111111

>DM_train523

MPYRNCWSKIMKKLINDVQDVLDEQLAGLAKAHPSLTLHQDPVYVTRADAPVAGKVALLSGGGSGHEPMHCGYIGQGMLSGACPGEIFTSPTPDKIFECAMQVDGGEGVLLIIKNYTGDILNFETATELLHDSGVKVTTVVIDDDVAVKDSLYTAGRRGVANTVLIEKLVGAAAERGDSLDACAELGRKLNNQGHSIGIALGACTVPAAGKPSFTLADNEMEFGVGIHGEPGIDRRPFSSLDQTVDEMFDTLLVNGSYHRTLRFWDYQQGSWQEEQQTKQPLQSGDRVIALVNNLGATPLSELYGVYNRLTTRCQQAGLTIERNLIGAYCTSLDMTGFSITLLKVDDETLALWDAPVHTPALNWGK

111111111111111111100000000000000000000000000000000000000000000000000000000000000000000000000000000000000000000000000000000000000000000000000000000000000000000000000000000000000000000000000000000000000000111111111110000000000000000000000000000000000000000000000000000000000000000000000000000000000000000000000000000000000000000000000000000000000000000000000000000000

>DM_train524

AVPATIPLTITNNSGRAEQIHIYNLGTELSSGRQGWADASGAFHPWPAGGNPPTPAPDASIPGPAPGRSTTIQIPKFSGRIYFSYGRKMEFRLTTGGLVQPAVQNPTDPNRDILFNWSEYTLNDSGLWINSTQVDMFSAPYTVGVRRGDGTTLSTGKLRPGGYNGVFNALRGQSGGWANLIQTRSDGTVLRALSPLYGVETGALPASVMDDYINRVWNKYTGTDLIVTPFADRPDVRYTGRVSGGVLRFTDGSGAVVTTFQKPDASSVFGCHRLLDAPNDQVRGPISRTLCAGFNRTTLLANPHQPDRSAAGFYQEPVTNHYARIIHAHMADGKAYGFAFDDVGHHESLVHDGDPRGASLTLDPFD

100000000000000000000000000000000000000000000000000000000000000000000000000000000000000000000000000000000000000000000000000000000000000000000000000000000000000000000000000000000000000000000000000000000000000000000000000000000000000000000000000000000000000000000000000000000000001110000000000000000000000000000000000000000000000000000000000000000000000000000000000000

>DM_train526

KKCKYSFKCVNSLKEDHNQPLFGVQFNWHSKEGDPLVFATVGSNRVTLYECHSQGEIRLLQSYVDADADENFYTCAWTYDSNTSHPLLAVAGSRGIIRIINPITMQCIKHYVGHGNAINELKFHPRDPNLLLSVSKDHALRLWNIQTDTLVAIFGGVEGHRDEVLSADYDLLGEKIMSCGMDHSLKLWRINSKRMMNAIKESYDYNPNKTNRPFISQKIHFPDFSTRDIHRNYVDCVRWLGDLILSKSCENAIVCWKPGKMEDDIDKIKPSESNVTILGRFDYSQCDIWYMRFSMDFWQKMLALGNQVGKLYVWDLEVEDPHKAKCTTLTHHKCGAAIRQTSFSRDSSILIAVCDDASIWRWDRLR

111100000000000000000000000000000000000000000000000000000000000000000000000000000000000000000000000000000000000000000000000000000000000000000000000000000000000000000000000000000000000000000000000000000000000000000000000000000000000000000000000000000000000000000000000000000000000000000000000000000000000000000000000000000000000000000000000000000000000000000000000011

>DM_train528

ESDSEMVPFPQLPMPIENNYRACTIPYRFPSDDPKKATPNEISWINVFANSIPSFKKRAESDITVPDAPARAEKFAERYAGILEDLKKDPESHGGPPDGILLCRLREQVLRELGFRDIFKKVKDEENAKAISLFPQVVSLSDAIEDDGKRLENLVRGIFAGNIFDLGSAQLAEVFSRDGMSFLASCQNLVPRPWVIDDLENFQAKWINKSWKKAVIFVDNSGADIILGILPFARELLRRGAQVVLAANELPSINDITCTELTEILSQLKDENGQLLGVDTSKLLIANSGNDLPVIDLSRVSQELAYLSSDADLVIVEGMGRGIETNLYAQFKCDSLKIGMVKHLEVAEFLGGRLYDCVFKFNEVQS

111100000000000000000000000000000000000000000000000000000000000000000000000000000000000000000000000000000000000000000000000000000000000000000000000000000000000000001111111111111110000000000000000000000000000000000000000000000000000000000000000000000000000000000000000001100000000000000000000000000000000000000000000000000000000000000000000000000000000000000000000011

>DM_train529

MKPHALIGLLAGMLLSSSLYAADSTKPLPLHIGGRVLVESPANQPVSYTYSWPAVYFETAFKGQSLTLKFDDDQNIFRLIVDDKAPVVINKPGKVDYPVESLAPGKHRVRLEKLTETQSTSGRFLGFYTDPSAKPLALPKRKRQIEFIGDSFTVGYGNTSPSRECTDEELFKTTNSQMAFGPLTAKAFDADYQINASSGFGIVRNYNGTSPDKSLLSLYPYTLNNPDQLYHNKHWKPQVIVIGLGTNDFSTALNDNERWKTREALHADYVANYVKFVKQLHSNNARAQFILMNSDQSNGEIAEQVGKVVAQLKGGGLHQVEQIVFKGLDYSGCHWHPSANDDQLLANLLITHLQQKKGIWLEHHHH

111111111111111111111111110000000000000001100000000000000000010000000000000000000110000000000000001111111110000000000000000000011111111100000000000000000000000000000000000000000000000000000000000000000000000000000000000000000000000000000000000000000000000000000000000000000000000000000000000000000000000000000000000000000000000000000000000000000000000000000000011111

>DM_train530

MVVEGVKTDFGPPYFRDLLHPVIAKNYGKWKYHEVVKPGVIKRVAESGDVIYVVRFGTPRLLSIYTVRELCDIADKYSDGYLRWTSRNNVEFFVTDESKIDDLINEVQERVGFPCGGTWDAVKGEYGLSNIVHTQGWIHCHTPAIDASGIVKAVMDELYEYFTDHKLPAMCRISLACCANMCGAVHASDIAIVGIHRTPPIPNDEAIRKTCEIPSTVAACPTGALKPDMKNKTIKVDVEKCMYCGNCYTMCPGMPLFDPENDGAAIMVGGKLSEARRMPELSKVVVPWVPNEPPRWPTLVKYVKQILEAWAANANKHERLIEWVDRIGWERFFELTGLEFTQHLIDDYRITPYFYSEFRASTQFKW

111000000000000000000000000000000000000000000000000000000000000000000000000000000000000000000000000000000000000000000000000000000000000000000000000000000000000000000000000000000000000000000000000000000000000000000000000000000000000000000000000000000000000000000000000000000000000000000000000000000000000000000000000000000000000000000000000000000000000000000000000000

>DM_train533

NVIDLFSGVGGLSLGAARAGFDVKMAVEIDQHAINTHAINFPRSLHVQEDVSLLNAEIIKGFFKNDMPIDGIIGGPPCQGFSSIGKGNPDDSRNQLYMHFYRLVSELQPLFFLAENVPGIMQEKYSGIRNKAFNLVSGDYDILDPIKVKASDYGAPTIRTRYFFIGVKKSLKLDISDEVFMPKMIDPVTVKDALYGLPDIIDANWQSDSESWRTIKKDRKGGFYEKLWGQIPRNVGDTESIAKLKNNIISGCTGTLHSKIVQERYASLSFGETDKISRSTRLDPNGFCPTLRAGTARDKGSFQAVRPIHPYHPRVITPREAARLQGFPDWFRFHVTKWHSFRQIGNSVSPIVAEYILKGLYNLLN

00000000000000000000000000000000000000000000000000000000000000000000000000000000001111111000000000000000000000000000000000000000000000000000000000000000000000000000000000000000000000000000000000000000000000000000000000000000000000000000000000000000000000000000000000000000000000000000000000011111111111110000000000000000000000000000000000000000000000000000000000000

>DM_train534

MTSRVDPANPGSELDSAIRDTLTYSNSPVPNALLTASESGFLDAAGIELDVLSGQQGTVHFTYDQPAYTRFGGEIPPLLSEGLRAPGRTRLLGITPLLGRQGFFVRDDSPITAAADLAGRRIGVSASAIRILRGQLGDYLELDPWRQTLVALGSWEARALLHTLEHGELGVDDVELVPISSPGVDVPAEQLEESATVKGADLFPDVARGQAAVLASGDVDALYSWLPWAGELQATGARPVVDLGLDERNAYASVWTVSSGLVRQRPGLVQRLVDAAVDAGLWARDHSDAVTSLHAANLGVSTGAVGQGFGADFQQRLVPRLDHDALALLERTQQFLLTNNLLQEPVALDQWAAPEFLNNSLNRHR

11111111111111111110000000000000000000000000000000000000000000000000000000000000000000000000000000000000000000000000000000000000000000000000000000000000000000000000000000000000000000000000000000000000000000000000000000000000000000000000000000000000000000000000000000000000000000000000000000000000000000000000000000000000000000000000000000000000000000000000000000011

>DM_train536

MMSGQGKRLMVMAGGTGGHVFPGLAVAHHLMAQGWQVRWLGTADRMEADLVPKHGIEIDFIRISGLRGKGIKALIAAPLRIFNAWRQARAIMKAYKPDVVLGMGGYVSGPGGLAAWSLGIPVVLHEQNGIAGLTNKWLAKIATKVMQAFPGAFPNAEVVGNPVRTDVLALPLPQQRLAGREGPVRVLVVGGSQGARILNQTMPQVAAKLGDSVTIWHQSGKGSQQSVEQAYAEAGQPQHKVTEFIDDMAAAYAWADVVVCRSGALTVSEIAAAGLPALFVPFQHKDRQQYWNALPLEKAGAAKIIEQPQLSVDAVANTLAGWSRETLLTMAERARAASIPDATERVANEVSRVARALEHHHHHH

1111110000000000000000000000000000000000000000000000000000000000000000000000000000000000000000000000000000000000000000000000000000000000000000000000000000000000000000000000000000000000000000000000000000000000000000000000000000000000000000000000000000000000000000000000000000000000000000000000000000000000000000000000000000000000000000000000000000000000000011111111

>DM_train537

MGFKCGIVGLPNVGKSTLFNALTKAGIEAANYPFCTIEPNTGVVPMPDPRLDALAEIVKPERILPTTMEFVDIAGLVAGASKGEGLGNKFLANIRETDAIGHVVRCFENDDIVHVAGKIDPLDDIDTINTELALADLDSCERAIQRLQKRAKGGDKEAKFELSVMEKILPVLENAGMIRSVGLDKEELQAIKSYNFLTLKPTMYIANVNEDGFENNPYLDRVREIAAKEGAVVVPVCAAIESEIAELDDEEKVEFLQDLGIEEPGLNRVIRAGYALLNLQTYFTAGVKEVRAWTVSVGATAPKAAAVIHTDFEKGFIRAEVIAYEDFIQFNGENGAKEAGKWRLEGKDYIVQDGDVMHFRFNV

000000000000000000000000011111111111111100000000000000000000000000000000000000000000000000000000000000000000111111111111100000000000000000000000000000000000000000000000000000000000000000000000000000000000000000000000000000000000000000000000000000000000000000000000000000000000000000000000000000000000000000000000000000000000000000000000000000000000000000000000000

>DM_train538

ASTLKSGSKEVENLKKPFMPPREVHVQVTHSMPPQKIEIFKSLDNWAEENILVHLKPVEKCWQPQDFLPDPASDGFDEQVRELRERAKEIPDDYFVVLVGDMITEEALPTYQTMLNTLDGVRDETGASPTSWAIWTRAWTAEENRHGDLLNKYLYLSGRVDMRQIEKTIQYLIGSGMDPRTENSPYLGFIYTSFQERATFISHGNTARQAKEHGDIKLAQICGTIAADEKRHETAYTKIVEKLFEIDPDGTVLAFADMMRKKISMPAHLMYDGRDDNLFDHFSAVAQRLGVYTAKDYADILEFLVGRWKVDKLTGLSAEGQKAQDYVCRLPPRIRRLEERAQGRAKEAPTMPFSWIFDRQVKL

111111111111111110000000000000000000000000000000000000000000000000000000000000000000000000000000000000000000000000000000000000000000000000000000000000000000000000000000000000000000000000000000000000000000000000000000000000000000000000000000000000000000000000000000000000000000000000000000000000000000000000000000000000000000000000000000011111111000000000000000000

>DM_train539

MFQPLLDAYVESASIEKMASKSPPPLKIAVANWWGDEEIKEFKNSVLYFILSQRYTITLHQNPNEFSDLVFGNPLGSARKILSYQNAKRVFYTGENESPNFNLFDYAIGFDELDFNDRYLRMPLYYDRLHHKAESVNDTTAPYKLKDNSLYALKKPSHCFKEKHPNLCAVVNDESDPLKRGFASFVASNPNAPIRNAFYDALNSIEPVTGGGSVRNTLGYNVKNKNEFLSQYKFNLCFENTQGYGYVTEKIIDAYFSHTIPIYWGSPSVAKDFNPKSFVNVHDFKNFDEAIDYIKYLHTHKNAYLDMLYENPLNTLDGKAYFYQNLSFKKILAFFKTILENDTIYHDNPFIFCRDLNEPLVTI

000000000000000000000000000000000000000000000000000000000000000000000000001111111111000000000000000000000000000000000000000000000000000000000000000000000000000000000000000000000000000000000000000000000000000000000000000000000000000000000000000000000000000000000000000000000000000000000000000000000000000000000000000000000000000000000000000000000000111111111111111

>DM_train540

MHSFASLLAYGLVAGATFASASPIEARDSCTFTTAAAAKAGKAKCSTITLNNIEVPAGTTLDLTGLTSGTKVIFEGTTTFQYEEWAGPLISMSGEHITVTGASGHLINCDGARWWDGKGTSGKKKPKFFYAHGLDSSSITGLNIKNTPLMAFSVQANDITFTDVTINNADGDTQGGHNTDAFDVGNSVGVNIIKPWVHNQDDCLAVNSGENIWFTGGTCIGGHGLSIGSVGDRSNNVVKNVTIEHSTVSNSENAVRIKTISGATGSVSEITYSNIVMSGISDYGVVIQQDYEDGKPTGKPTNGVTIQDVKLESVTGSVDSGATEIYLLCGSGSCSDWTWDDVKVTGGKKSTACKNFPSVASC

11111111111111111111111111100000000000000000000000000000000000000000000000000000000000000000000000000000000000000000000000000000000000000000000000000000000000000000000000000000000000000000000000000000000000000000000000000000000000000000000000000000000000000000000000000000000000000000000000000000000000000000000000000000000000000000000000000000000000000000000000

>DM_train541

SVNNSQLVVSVAGTVEGTNQDISLKFFEIDLTSRPAHGGKTEQGLSPKSKPFATDSGAMPHKLEKADLLKAIQEQLIANVHSNDDYFEVIDFASDATITDRNGKVYFADKDGSVTLPTQPVQEFLLSGHVRVRPYKEKPIQNQAKSVDVEYTVQFTPLNPDDDFRPGLKDTKLLKTLAIGDTITSQELLAQAQSILNKTHPGYTIYERDSSIVTHDNDIFRTILPMDQEFTYHVKNREQAYEINKKSGLNEEINNTDLISEKYYVLKKGEKPYDPFDRSHLKLFTIKYVDVNTNELLKSEQLLTASERNLDFRDLYDPRDKAKLLYNNLDAFGIMDYTLTGKVEDNHDDTNRIITVYMGKRP

00000000000000000000000000000000001111111111111111111111111000000000000000000000000000000000000000000000000000000000000000000000000000000000000000000000000000000001111111000000000000000000000000000000000000000000000000000000000000000000000011111111111000000000000000000000000000000000000000000000000000000000000000000000000000000000000000000000000000000000000000

>DM_train542

ALTVSAGTKSKLAKPIQDLIKMIFDVESMKKAMVEFEIDLQKMPLGKLSKRQIQSAYSILNEVQQAVSDGGSESQILDLSNRFYTLIPHDFGMKKPPLLSNLEYIQAKVQMLDNLLDIEVAYSLLRGGNEDGDKDPIDINYEKLRTDIKVVDKDSEEAKIIKQYVKNTHAATHNAYDLKVVEIFRIEREGESQRYKPFKQLHNRQLLWHGSRTTNFAGILSQGLRIAPPEAPVTGYMFGKGIYFADMVSKSANYCHTSQADPIGLILLGEVALGNMYELKNASHITKLPKGKHSVKGLGKTAPDPTATTTLDGVEVPLGNGISTGINDTCLLYNEYIVYDVAQVNLKYLLKLKFNYKTSLW

1111111100000000000000000000000000000000000000000000000000000000000000000000000000000000000000000000000000000000000000000000000000000000000000000000000000000000000000000000000000000000000000000000000000000000000000000000000000000000000000000000000000000000000000000000000000000000000000000000000000000000000000000000000000000000000000000000000000000000000011111

>DM_train544

VKQVFNFNAGPSALPKPALERAQKELLNFNDTQMSVMELSHRSQSYEEVHEQAQNLLRELLQIPNDYQILFLQGGASLQFTMLPMNLLTKGTIGNYVLTGSWSEKALKEAKLLGETHIAASTKANSYQSIPDFSEFQLNENDAYLHITSNNTIYGTQYQNFPEINHAPLIADMSSDILSRPLKVNQFGMIYAGAQKNLGPSGVTVVIVKKDLLNTKVEQVPTMLQYATHIKSDSLYNTPPTFSIYMLRNVLDWIKDLGGAEAIAKQNEEKAKIIYDTIDESNGFYVGHAEKGSRSLMNVTFNLRNEELNQQFLAKAKEQGFVGLNGHRSVGGCRASIYNAVPIDACIALRELMIQFKENA

000000000000000000000000000000000000000000000000000000000000000000000000000000000000000000000000000000000000000000000000000000000000000000000000000000000000000000000000000000000000000000000000000000000000000000000111000000000000000000000000000000000000000000000000000000000000000000000000000000000000000000000000000000000000000000000000000000000000000000000000

>DM_train545

PPFPWFGMDIGGTLVKLVYFEPKDITAEEEQEEVENLKSIRKYLTSNTAYGKTGIRDVHLELKNLTMCGRKGNLHFIRFPSCAMHRFIQMGSEKNFSSLHTTLCATGGGAFKFEEDFRMIADLQLHKLDELDCLIQGLLYVDSVGFNGKPECYYFENPTNPELCQKKPYCLDNPYPMLLVNMGSGVSILAVYSKDNYKRVTGTSLGGGTFLGLCCLLTGCETFEEALEMAAKGDSTNVDKLVKDIYGGDYERFGLQGSAVASSFGNMMSKEKRDSISKEDLARATLVTITNNIGSIARMCALNENIDRVVFVGNFLRINMVSMKLLAYAMDFWSKGQLKALFLEHEGYFGAVGALLELFK

110000000000000000000000111111111111000000000000000000000000000000011000000000000000000000011111111111100000000000000111111100000000000000000000000000000000000000000000000000000000000000000000000000000000000000000000000000000000000000000000000000000000000000000000000000000000000000000000000000000000000000000000000000000000000000000000000000000000000000000000

>DM_train546

SCDLNATNYIRGCQSKTYDGKIFPGKGGEKQWICKDTIIHGDTNGACIPPRTQNLCVGELWDKSYGGRSNIKNDTKELLKEKIKNAIHKETELLYEYHDTGTAIISKNDKKGQKGKNDPNGLPKGFCHAVQRSFIDYKNMILGTSVNIYEHIGKLQEDIKKIIEKGTPQQKDKIGGVGSSTENVNAWWKGIEREMWDAVRCAITKINKKNNNSIFNGDECGVSPPTGNDEDQSVSWFKEWGEQFCIERLRYEQNIREACTINGKNEKKCINSKSGQGDKIQGACKRKCEKYKKYISEKKQEWDKQKTKYENKYVGKSASDLLKENYPECISANFDFIFNDNIEYKTYYPYGDYSSICSCE

000000000000000000000000000000000000000000000000000000000000000000000000000000000000000000000000000000000000000000000000000000000000000000000000000000000000000000000000001111111110000000000000000000000000000000000000000000000011110000000000000000000000000000001111100000001111000000000000000000000000000000000000000000000000000000000000000000000000000000000000

>DM_train547

MRGSHHHHHHGSLVPRGSMVAADAPAAGTDITNQLTNVTVGIDSGTTVYPHQAGYVKLNYGFSVPNSAVKGDTFKITVPKELNLNGVTSTAKVPPIMAGDQVLANGVIDSDGNVIYTFTDYVNTKDDVKATLTMPAYIDPENVKKTGNVTLATGIGSTTANKTVLVDYEKYGKFYNLSIKGTIDQIDKTNNTYRQTIYVNPSGDNVIAPVLTGNLKPNTDSNALIDQQNTSIKVYKVDNAADLSESYFVNPENFEDVTNSVNITFPNPNQYKVEFNTPDDQITTPYIVVVNGHIDPNSKGDLALRSTLYGYNSNIIWRSMSWDNEVAFNNGSGSGDGIDKPVVPEQPDEPGEIEPIPEK

11111111111111111111111111100000000000000000000000000000000000000000000000000000000000000000000000000000000000000000000000000000000000000000000000000000000000000000000000000000000000000000000000000000000000000000000000000000000000000000000000000000000000000000000000000000000000000000000000000000000000000000000000000000000000000000000000000000000000000000000

>DM_train548

MIESKTYAVLGLGNGGHAFAAYLALKGQSVLAWDIDAQRIKEIQDRGAIIAEGPGLAGTAHPDLLTSDIGLAVKDADVILIVVPAIHHASIAANIASYISEGQLIILNPGATGGALEFRKILRENGAPEVTIGETSSMLFTCRSERPGQVTVNAIKGAMDFACLPAAKAGWALEQIGSVLPQYVAVENVLHTSLTNVNAVMHPLPTLLNAARCESGTPFQYYLEGITPSVGSLAEKVDAERIAIAKAFDLNVPSVCEWYKESYGQSPATIYEAVQGNPAYRGIAGPINLNTRYFFEDVSTGLVPLSELGRAVNVPTPLIDAVLDLISSLIDTDFRKEGRTLEKLGLSGLTAAGIRSAVE

11100000000000000000000000000000000000000000000000000000000000000000000000000000000000000000000000000000000000000000000000000000000000000000000000000000000000000000000000000000000000000000000000000000000000000000000000000000000000000000000000000000000000000001111111000000000000000000000000000000000000000000000000000000000000000000000000000000000000000000000

>DM_train549

MGDVRLSALSTLNYRNLAPGTLNFPEGVTGIYGENGAGKTNLLEAAYLALTGQTDAPRIEQLIQAGETEAYVRADLQQGGSLSIQEVGLGRGRRQLKVDGVRARTGDLPRGGAVWIRPEDSELVFGPPSGRRAYLDSLLSRLSARYGEQLSRYERTVSQRNAALRGGEEWAMHVWDDVLLKLGTEIMLFRRRALTRLDELAREANAQLGSRKTLALTLTESTSPETYAADLRGRRAEELARGSTVTGPHRDDLLLTLGDFPASDYASRGEGRTVALALRRAELELLREKFGEDPVLLLDDFTAELDPHRRQYLLDLAASVPQAIVTGTELAPGAALTLRAQAGRFTPVADEEMQAEGTA

10000000000000000000000000000000000000000000000000000000000000000000000000000000000000000000000000000000000000000000000000000000000000000000000000000000000000000000000000000000000000000000000000000000000000000000000000000000000000000000000000000000000000000000000000000000000000000000000000000000000000000000000000000000000000000000000000000000000000000000000

>DM_train550

PGDVVIEELFNRIPQANVRTTSEYMQSAADSLVSTSLWNTGQPFRVESELGERPRTLVRGTVLGQEDPYAYLEATDQETGESFEVHVPYFTERPPSNAIKQMKEEVLRLRLLRGIKNQKQAKVHLRFIFPFDLVKDPQKKKMIRVRLDERDMWVLSRFFLYPRMQSNLQTFGEVLLSHSSTHKSLVHHARLQLTLQVIRLLASLHHYGLVHTYLRPVDIVLDQRGGVFLTGFEHLVRDGARVVSSVSRGFEPPELEARRATISYHRDRRTLMTFSFDAWALGLVIYWIWCADLPITKDAALGGSEWIFRSCKNIPQPVRALLEGFLRYPKEDRLLPLQAMETPEYEQLRTELSAALPLY

00000000000000000000000100000000000000010000000000000000000000000000000000001000000000000000100000000000000000000000000000000000000000000000000001111110000000000000000000000000000000000000000000000000000000000000000000000000000000000000000000000000000000000000000000000000000000000000000000000001111110000000000000000000000000000000000000000000000000000000000

>DM_train551

MKIAILGAGCYRTHAAAGITNFMRACEVAKEVGKPEIALTHSSITYGAELLHLVPDVKEVIVSDPCFAEEPGLVVIDEFDPKEVMEAHLSGNPESIMPKIREVVKAKAKELPKPPKACIHLVHPEDVGLKVTSDDREAVEGADIVITWLPKGNKQPDIIKKFADAIPEGAIVTHACTIPTTKFAKIFKDLGREDLNITSYHPGCVPEMKGQVYIAEGYASEEAVNKLYEIGKIARGKAFKMPANLIGPVCDMCSAVTATVYAGLLAYRDAVTKILGAPADFAQMMADEALTQIHNLMKEKGIANMEEALDPAALLGTADSMCFGPLAEILPTALKVLEKHKVVEEEGKTKCEIMSQKE

0000000000000000000000000000000000000000000000000000000000000000000000000000000000000000000000000000000000000000000000000000000000000000000000000000000000000000000000000000000000000000000000000000000000000000000000000000000000000000000000000000000000000000000000000000000000000000000000000000000000000000000000000000000000000000000000000000000011111111111111

>DM_train552

MAVKEATSETKKRSGYEIITLTSWLLQQEQKGIIDAELTIVLSSISMACKQIASLVQRANISNLTGTQGAVNIQGEDQKKLDVISNEVFSNCLRSSGRTGIIASEEEDVPVAVEESYSGNYIVVFDPLDGSSNLDAAVSTGSIFGIYSPNDESLPDFGDDSDDNTLGTEEQRCIVNVCQPGSNLLAAGYCMYSSSVIFVLTIGKGVFVFTLDPLYGEFVLTQENLQIPKSGKIYSFNEGNYKLWDENLKKYIDDLKEPGPSGKPYSARYIGSLVGDFHRTLLYGGIYGYPRDKKSKNGKLRLLYECAPMSFIVEQAGGKGSDGHQRVLDIQPTEIHQRVPLYIGSTEEVEKVEKYLA

111111111111111110000000000000000000000000000000000000000000000001111111111000000000000000000000000000000000000000000000000000000000000000000000000000111111111111100000000000000000000000000000000000000000000000000000000000000000000000000000000000000000000000000000000000000000000000000000000000000000000000000000000000000000000000000000000000000000000000000

>DM_train553

MSAFEKPQIIAHIQKGFNYTVFDCKWVPCSAKFVTMGNFARGTGVIQLYEIQHGDLKLLREIEKAKPIKCGTFGATSLQQRYLATGDFGGNLHIWNLEAPEMPVYSVKGHKEIINAIDGIGGLGIGEGAPEIVTGSRDGTVKVWDPRQKDDPVANMEPVQGENKRDCWTVAFGNAYNQEERVVCAGYDNGDIKLFDLRNMALRWETNIKNGVCSLEFDRKDISMNKLVATSLEGKFHVFDMRTQHPTKGFASVSEKAHKSTVWQVRHLPQNRELFLTAGGAGGLHLWKYEYPIQRSKKDSEGIEMGVAGSVSLLQNVTLSTQPISSLDWSPDKRGLCVCSSFDQTVRVLIVTKLNKI

111100000000000000000000000000000000001100000000000000000000000000000000000000000000000000000000000000000000000000000000000000000000000000000000000000000000000000000000000000000000000000000000000000000000000000000000000000000000000000000000000000000000000000000000000000000000000000000000000000011000000000000000000000000000000000000000000000000000000001111

>DM_train554

GMNRLRTSFQQTTGQISGHGKRNVGVLKTAFAAVADEMASDQYGTGAIIEPFEQKFADVLGMDDAVFFPSGTMAQQVALRIWSDETDNRTVAYHPLCHLEIHEQDGLKELHPIETILVGAADRLMTLDEIKALPDIACLLLELPQREIGGVAPAFSELETISRYCRERGIRLHLDGARLFEMLPYYEKTAAEIAGLFDSIYISFYKGLGGIAGAILAGPAAFCQTARIWKRRYGGDLISLYPYIVSADYYYELRKDRMGQYYEQAKQLAEQFNALPGVHTTPEVPVSNMFHLHFDGQAADISPKLEQVQEETGLGFVGYLVDKDGYCSTEISVGDAYGELDQQTRDAGFARLRQAFS

110000000000000000000000000000000000000000000000000000000000000000000000000000000000000000000000000000000000000000000000000000000000000000000000000000000000000000000000000000000000000000000000000000000000000000000000000000000000000000000000000000000000000000000000000000000000000000000000000000000000000000000000000000000000000000000000000000000000000000001

>DM_train555

MLHILCQGTPFEIGYEHGSAAKAVIARSIDFAVDLIRGKTKKTDEELKQVLSQLGRVIEERWPKYYEEIRGIAKGAERDVSEIVMLNTRTEFAYGLKAARDGCTTAYCQLPNGALQGQNWDFFSATKENLIRLTIRQAGLPTIKFITEAGIIGKVGFNSAGVAVNYNALHLQGLRPTGVPSHIALRIALESTSPSQAYDRIVEQGGMAASAFIMVGNGHEAFGLEFSPTSIRKQVLDANGRMVHTNHCLLQHGKNEKELDPLPDSWNRHQRMEFLLDGFDGTKQAFAQLWADEDNYPFSICRAYEEGKSRGATLFNIIYDHARREATVRLGRPTNPDEMFVMRFDEEDERSALNARL

000000000000000000000000000000000000000000000000000000000000000000000000000000000000000000000000011111000000000000000000000000000000000000000000000000000000000000000000000000000000000000000000000000000000000000000000000000000000000000000000000000000000000000000000000000000000000000000000000000000000000000000000000000000000000000000000000000000000000000001

>DM_train558

MQTLHALLRDIPAPDAEAMARTQQHIDGLLKPPGSLGRLETLAVQLAGMPGLNGTPQVGEKAVLVMCADHGVWDEGVAVSPKIVTAIQAANMTRGTTGVCVLAAQAGAKVHVIDVGIDAEPIPGVVNMRVARGCGNIAVGPAMSRLQAEALLLEVSRYTCDLAQRGVTLFGVGELGMANTTPAAAMVSVFTGSDAKEVVGIGANLPPSRIDNKVDVVRRAIAINQPNPRDGIDVLSKVGGFDLVGMTGVMLGAARCGLPVLLDGFLSYSAALAACQIAPAVRPYLIPSHFSAEKGARIALAHLSMEPYLHMAMRLGEGSGAALAMPIVEAACAMFHNMGELAASNIVLPEGNANAT

11100000000000000000000000000000000000000000000000000000000000000000000000000000000000000000000000000000000000000000000000000000000000000000000000000000000000000000000000000000000000000000000000000000000000000000000000000000000000000000000000000000000000000000000000000000000000000000000000000000000000000000000000000000000000000000000000000001111111111111

>DM_train559

EIKTTTTLHRVVEETTKPLGATLVVETDISRKDVNGLARGHLVDGIPLCTPSFYADIAMQVGQYSMQRLRAGHPGAGAIDGLVDVSDMVVDKALVPHGKGPQLLRTTLTMEWPPKAAATTRSAKVKFATYFADGKLDTEHASCTVRFTSDAQLKSLRRSVSEYKTHIRQLHDGHAKGQFMRYNRKTGYKLMSSMARFNPDYMLLDYLVLNEAENEAASGVDFSLGSSEGTFAAHPAHVDAITQVAGFAMNANDNVDIEKQVYVNHGWDSFQIYQPLDNSKSYQVYTKMGQAKENDLVHGDVVVLDGEQIVAFFRGLTLRSVPRGALRVVLQTTVKKADRQLGFKTMPSPPPPTTTM

00000000000000000000000000000000000000000000000000000000000000000000001111111111000000000000000000000000000000000000000000000000000111100000000000000000000000000000000000000000000000000000000000000000000000000000000000000000000000000000000000000000000000000000000000000000000000000000000000011000000000000000000000000000000000000000111111111111111111111111

>DM_train560

MKLTLKNLSMAIMMSTIVMGSSAMAADSNEKIVIAHRGASGYLPEHTLPAKAMAYAQGADYLEQDLVMTKDDNLVVLHDHYLDRVTDVADRFPDRARKDGRYYAIDFTLDEIKSLKFTEGFDIENGKKVQTYPGRFPMGKSDFRVHTFEEEIEFVQGLNHSTGKNIGIYPEIKAPWFHHQEGKDIAAKTLEVLKKYGYTGKDDKVYLQCFDADELKRIKNELEPKMGMELNLVQLIAYTDWNETQQKQPDGSWVNYNYDWMFKPGAMKQVAEYADGIGPDYHMLIEETSQPGNIKLTGMVQDAQQNKLVVHPYTVRSDKLPEYTPDVNQLYDALYNKAGVNGLFTDFPDKAVKFLN

11111111111111111111111111110000000000000000000000000000000000000000000000000000000000000000000000000000000000000000000000000000000000000000000000000000000000000000000000000000000000000000000000000000000000000000000000000000000000000000000000000000000000000000000000000000000000000000000000000000000000000000000000000000000000000000000000000000000000000000

>DM_train561

HHHHHHPMSNTNIPTTENLYPEGAMVNKNKEGLNIDGKEVLAGSTNYYELTWDLDQYKGDKSSKEAIQNGFYYVDDYPEEALDVRPDLVKVADEKGNQVSGVSVQQYDSLEAAPKKVQDLLKKANITVKGAFQLFSADNPEEFYKQYVATGTSLVITDPMTVKSEFGKTGGKYENKAYQIDFGNGYATEVVVNNVPKITPKKDVTVSLDPTSENLDGQTVQLYQTFNYRLIGGLIPQNHSEELEDYSFVDDYDQAGDQYTGNYKTFSSLNLTMKDGSVIKAGTDLTSQTTAETDATNGIVTVRFKEDFLQKISLDSPFQAETYLQMRRIAIGTFENTYVNTVNKVAYASNTVRTTT

11111111111111111111111000000000000000000000000000000000000000000000000000000000000000000000000000000000000000000000000000000000000000000000000000000000000000000000000000000000000000000000000000000000000000000000000000000000000000000000000000000000000000000000000000000000000000000000000000000000000000000000000000000000000000000000000000000000000000000000

>DM_train563

MRICLFLCLCLCMASPALAQVAVFPALSGKTDAQTLVVYSSLDEPLATPMIEGFQKANPDIAVHYEDMLTGEIYDRIVKETDAGKKTADFAFSSAMDLQVKLSNDGYAQRSDLAMSARWPAWANWRNTAYALTFEPAVFVYHKPSFTTEKPPATRAEFVDYLERHAKEVHGRIATYDIERSGVGFLFMSRDQEQFGDIWSVIKAMGAAGVKVYSTSSAILERVSDGRFVLGYNILGSYAADWASRHPDVGIVLPKDYTVVMSRIGLVPEAAANPELGRRYLEFFMSKEGQTIMARQLQIPAVSPEVAGENTANTMQAIHGAQLRPVPVSPGLMVYLDQVKRSRLIERWNEALRSQ

1111111111111110000000000000011110000000000000000000000000000000000000000000000000000000000000000000000000000000000000000000000000000000000000000000000000000000000000000000000000001000000000000000000000000000000000000000000000000000000000000000000000000000000000000000000000000000000000000000000000000000000000000000000000000000000000000000000000000000001

>DM_train565

METMKSKANCAQNPNCNIMIFHPTKEEFNDFDKYIAYMESQGAHRAGLAKIIPPKEWKARETYDNISEILIATPLQQVASGRAGVFTQYHKKKKAMTVGEYRHLANSKKYQTPPHQNFEDLERKYWKNRIYNSPIYGADISGSLFDENTKQWNLGHLGTIQDLLEKECGVVIEGVNTPYLYFGMWKTTFAWHTEDMDLYSINYLHLGEPKTWYVVPPEHGQRLERLARELFPGSSRGCGAFLRHKVALISPTVLKENGIPFNRITQEAGEFMVTFPYGYHAGFNHGFNCAEAINFATPRWIDYGKMASQCSCGEARVTFSMDAFVRILQPERYDLWKRGQDRAVVDHMEPRVPA

111111111100000000000000000000000000000000000000000000000000000000000000000000000000000000000000000000000000000000000000000000000000000000000000000000000000000000000000000000000000000000000000000000000000000000000000000000000000000000000000000000000000000000000000000000000000000000000000000000000000000000000000000000000000000000000000000001111111111111

>DM_train566

MKRVFVFQDFKSQKFWSIDVRGTDVIVNYGKLGTDGQTQVKNFSSAGEAEKAAGKLIAEKTKKGYVETLEEVAKEMKVEAKKYALSYDEAEEGVNLMDKILKDKKLPSLKQITIGCWGYEGEDCSDIADGIVENKEKFAHFEGLFWGDIDFEEQEISWIEQVDLSPVLDAMPLLNNLKIKGTNNLSIGKKPRPNLKSLEIISGGLPDSVVEDILGSDLPNLEKLVLYVGVEDYGFDGDMNVFRPLFSKDRFPNLKWLGIVDAEEQNVVVEMFLESDILPQLETMDISAGVLTDEGARLLLDHVDKIKHLKFINMKYNYLSDEMKKELQKSLPMKIDVSDSQEYDDDYSYPMITE

000000000000000000000000000000000000000000000000000000000000000000000000000000000000000000000000000000000000000000000000000000000000000000000000000000000000000000000000000000000000000000000000000000000000000000000000000000000000000000000000000000000000000000000000000000000000000000000000000000000000000000000000000000000000000000000000000000011111100000

>DM_train567

GMYEEWKIVKREAPILGNDQLIENIWKMKREDSPYDIISLHKVNLIGGGNDAVLILPGTWSSGEQLVTISWNGVHYTIPDYRKSIVLYLARNGFNVYTIDYRTHYVPPFLKDRQLSFTANWGWSTWISDIKEVVSFIKRDSGQERIYLAGESFGGIAALNYSSLYWKNDIKGLILLDGGPTKHGIRPKFYTPEVNSIEEMEAKGIYVIPSRGGPNNPIWSYALANPDMPSPDPKYKSISDFLMDSLYVTGSANPYDYPYSKKEDMFPILASFDPYWPYRLSLERDLKFDYEGILVPTIAFVSERFGIQIFDSKILPSNSEIILLKGYGHLDVYTGENSEKDVNSVVLKWLSQQR

110000000000000000000000000000000000000000000000000000000000000000000000000000000000000000000000000000000000000000000000000000000000000000000000000000000000000000000000000000000000000000110000000000000000000000000000000000000000000000000000000000000000000000000000000000000000000000000000000000000000000000000000000000000000000000000000000000000000000000

>DM_train568

MGGSHHHHHHMASSVPIPGIKDISKLKFFYGFKYLWNPTVYNKIFDKLDLTKTYKHPEELKVLDLYPGVGIQSAIFYNKYCPRQYSLLEKRSSLYKFLNAKFEGSPLQILKRDPYDWSTYSNLIDEERIFVPEVQSSDHINDKFLTVANVTGEGSEGLIMQWLSCIGNKNWLYRFGKVKMLLWMPSTTARKLLARPGMHSRSKCSVVREAFTDTKLIAISDANELKGFDSQCIEEWDPILFSAAEIWPTKGKPIALVEMDPIDFDFDVDNWDYVTRHLMILKRTPLNTVMDSLGHGGQQYFNSRITDKDLLKKCPIDLTNDEFIYLTKLFMEWPFKPDILMDFVDMYQTEHSG

11111111111111100000000000000000000000000000000000000000000000000000000000000000000000000000000000000000000000000000000000000000000000000000000000000000000000000000000000000000000000000000000000000000000000000000000000000000000000000000000000000000000000000000000000000000000000000000000000000000000000000000000000000000000000000000000001111111111111111

>DM_train569

AGSDEVNRNECKTVVPLHTWVLISNFKLSYNILRRADGTFERDLGEYLDRRVPANARPLEGVSSFDHIIDQSVGLEVRIYRAAAEGDAEEGAAAVTRPILEFLTDAPAAEPFPVIIFFHGGSFVHSSASSTIYDSLCRRFVKLSKGVVVSVNYRRAPEHRYPCAYDDGWTALKWVMSQPFMRSGGDAQARVFLSGDSSGGNIAHHVAVRAADEGVKVCGNILLNAMFGGTERTESERRLDGKYFVTLQDRDWYWKAYLPEDADRDHPACNPFGPNGRRLGGLPFAKSLIIVSGLDLTCDRQLAYADALREDGHHVKVVQCENATVGFYLLPNTVHYHEVMEEISDFLNANLYY

11111111111111000000000000000000000000000000000000000000000000000000000000000000001111111111111111111111111111100000000000000000000000000000000000000000000000000000000000000000000000111111000000000000000000000000000000000000000000000000000000000000000000000000000000000000000000000000000000000000000000000000000000000000000000000000000000000000000000111

>DM_train572
[truncated: 1,397,063 more chars]
